# Comparative Proteomic Analysis of Experimental Evolution of the *Bacillus cereus-Ketogulonicigenium vulgare* Co-Culture

**DOI:** 10.1371/journal.pone.0091789

**Published:** 2014-03-11

**Authors:** Qian Ma, Yang Zou, Yajin Lv, Hao Song, Ying-Jin Yuan

**Affiliations:** Key Laboratory of Systems Bioengineering, Ministry of Education and Department of Pharmaceutical Engineering, Collaborative Innovation Center of Chemical Science and Engineering, School of Chemical Engineering and Technology, Tianjin University, Tianjin, P. R. China; University of Florida, United States of America

## Abstract

The microbial co-culture system composing of *Ketogulonicigenium vulgare* and *Bacillus cereus* was widely adopted in industry for the production of 2-keto-gulonic acid (2-KGA), the precursor of vitamin C. We found serial subcultivation of the co-culture could enhance the yield of 2-KGA by 16% in comparison to that of the ancestral co-culture. To elucidate the evolutionary dynamics and interaction mechanisms of the two microbes, we performed iTRAQ-based quantitative proteomic analyses of the pure cultures of *K. vulgare*, *B. cereus* and their co-culture during serial subcultivation. Hierarchy cluster analyses of the proteomic data showed that the expression level of a number of crucial proteins associated with sorbose conversion and oligopeptide transport was significantly enhanced by the experimental evolution. In particular, the expression level of sorbose/sorbosone dehydrogenase was enhanced in the evolved *K. vulgare*, while the expression level of InhA and the transport efficiency of oligopeptides were increased in the evolved *B. cereus.* The decreased sporulating protein expression and increased peptide transporter expression observed in evolved *B. cereus*, together with the increased amino acids synthesis in evolved *K. vulgare* suggested that serial subcultivation result in enhanced synergistic cooperation between *K. vulgare* and *B. cereus,* enabling an increased production of 2-KGA.

## Introduction

Social interactions and evolution are ubiquitous in microbial communities. Many studies have investigated the change of interactions in microbial communities during evolution processes. Experimental evolution is an efficient approach to emulate naturally occurring evolutionary processes [1]–[5]. For example, two mutualistic strains composing of *Methanogenic maripaludis* and *Desulfovibrio vulgaris* acquired a more stable and efficient mutualistic interaction *via* experimental evolution [5].

The co-culture consisting of *Ketogulonicigenium vulgare* and *Bacillus cereus* is widely adopted in the industrial production of vitamin C. In the co-culture, *K. vulgare* is responsible for the production of 2-keto-gulonic acid (2-KGA, the precursor of vitamin C), while *B. cereus*, as a companion bacterium, is able to accelerate the growth of *K. vulgare* and its 2-KGA production [6], [7]. A serial subcultivation-based experimental evolution (over 150 days) was conducted on this co-culture in our recent research, enabling an increased yield of 2-KGA from 77% (by the original co-culture) to 93% (by the evolved co-culture) [8]. However, the underlying mechanism of such yield enhancement by experimental evolution remained vague, which impeded further engineering efforts to increase its yield. We thus employed proteomic profiling to systematically study the characteristic of the two strains and their interactions in the co-culture during the experimental evolution process.

High-throughput omics-based systems biology methods were widely applied in the investigation of microbial communities [9]–[11]. Among these methods, proteomics has the advantage of bridging the gap between the upstream genome and the downstream metabolome, thus enabling a functional understanding of microbial communities [12]–[14]. In our previous study of the vitamin C fermentation process, proteomics had provided important insights into the purine flow between *K. vulgare* and *B. megaterium* [15] and the glutathione requirement of *K. vulgare* [16]. Given the powerful capability of proteomics in exploring the mutual relationship in microbial communities, we applied the iTRAQ based quantitative proteomics [16] to investigate the experimental evolution of the *K. vulgare*-*B. cereus* co-culture, and to elucidate the enhancement mechanism of the 2-KGA yield during experimental evolution.

Proteome variations of monoculture of *K. vulgare*, *B. cereus*, and their co-culture at the 0^th^, 50^th^, 100^th^, and 150^th^ day of subculture transfer were compared. Enhanced expression of proteins involved in purine, pyrimidine and amino acid biosynthesis was observed in the evolved *K. vulgare* strain, while changes in protein expression suggesting stronger peptide uptake and resistance to severe environmental conditions were observed in the evolved *B. cereus* strain. As opposed to other bacillus such as *B. subtilis*, *B. cereus* prefers a more carnivorous diet of proteins and amino acids [17]. Meanwhile, sporulation of *B. cereus* is usually resulted from nutrient limitation [18]. The decreased sporulating protein expression and increased peptide transporter expression observed in evolved *B. cereus*, together with the increased amino acids supply in evolved *K. vulgare* suggested a better synergistic cooperation of the consortium, which was resulted from the experimental evolution, enabling an increased yield of 2-KGA.

## Results and Discussion

### Proteomic Profiling of the Evolved *K. vulgare*

After 150 serial subcultivation transfers of the co-culture of *K. vulgare* and *B. cereus*, *K. vulgare* showed higher growth rate and yield of 2-KGA, from 77% (original co-culture) to 93% (evolved co-culture) [8]. However, the mechanism underlying the yield enhancement of 2-KGA by serial subcultivation remained unclear. To elucidate the systematic mechanism of the experimental evolution process, *K. vulgare* from the 0^th^, 50^th^, 100^th^ and 150^th^ transfers were thus compared via iTRAQ-based proteomic quantification.

**Figure 1:**
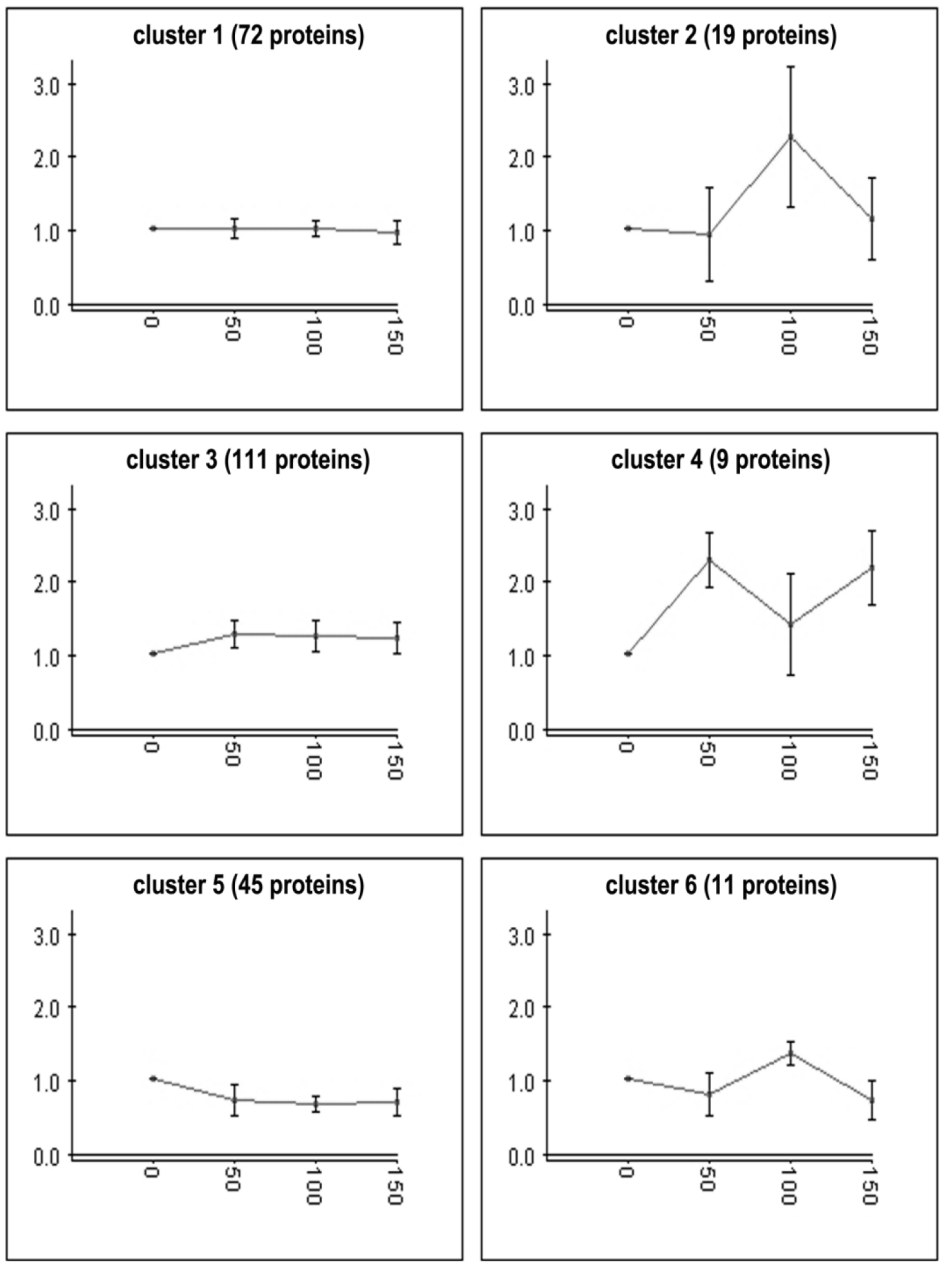
Hierarchy cluster analysis of *K. vulgare* proteins: 267 proteins were categorized into 6 clusters based on expression levels using K-means algorithm with the software Expander 4.1.

**Figure 2:**
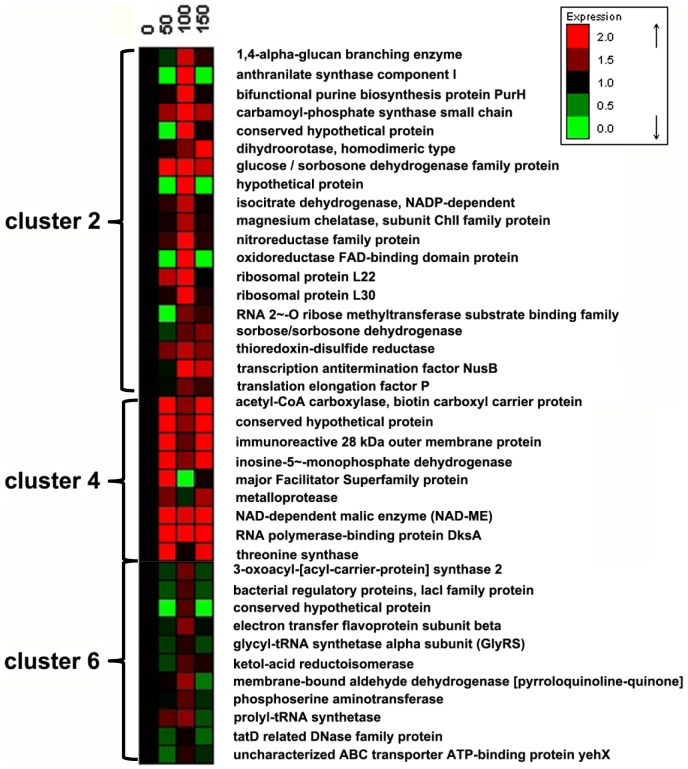
Proteins with significant expression variations of *K. vulgare* during the subcultivation: corresponding to clusters 2, 4 and 6 in Figure 1.

267 proteins were identified and categorized into 6 clusters by the K-means algorithm using Expander 4.1 (Fig. 1). Among these proteins, more than two thirds (clusters 1, 3 and 5) showed insignificant changes in the four transfers, while the other one third (clusters 2, 4 and 6) showed significantly increased protein level upon experimental evolution (*i.e.,* in the 50^th^, 100^th^ and 150^th^ transfers). The proteins with increased expression level (in clusters 2, 4 and 6, Fig. 2) include several dehydrogenases associated with the biosynthesis of 2-KGA (glucose/sorbosone dehydrogenase, sorbose/sorbosone dehydrogenase, membrane-bound aldehyde dehydrogenase), proteins for purine and pyrimidine biosynthesis (bifunctional purine biosynthesis protein PurH, inosine-5-monophosphate dehydrogenase, carbamoyl-phosphate synthase small chain, dihydroorotase), and proteins for amino acids biosynthesis (threonine synthase, glycyl-tRNA synthetase, phosphoserine aminotransferase, prolyl-tRNA synthetase).

Sorbose/sorbosone dehydrogenase was found to be an enzyme responsible for the conversion of L-sorbose to 2-KGA [19], [20]. Based on our previous researches, the expression of sorbose/sorbosone dehydrogenase was limited owing to the poor growth of *K. vulgare*. However, the expression of this dehydrogenase could be activated either by the existence of *B. megaterium* [15] or by the addition of glutathione (GSH) [16]. Here, we further found that upon experimental evolution, the expression of sorbose/sorbosone dehydrogenase increased, suggesting the reduction of sorbose and sorbosone was enhanced in the evolved *K. vulgare*.

According to our previous study, the purine biosynthesis was weak in *K. vulgare,* but it could be enhanced by the co-culturing with *B. megaterium* [15]. From the proteomic study of experimental evolution in this study, we found the biosynthesis of purine and pyrimidine was improved in the evolved *K. vulgare* in the co-culture. Meanwhile, the amino acid biosynthesis in the evolved *K. vulgare* was also enhanced, which subsequently facilitated the growth of *K. vulgare*. Our previous study showed that *K. vulgare* could efficiently degrade proteins and peptides in the medium into amino acids. However, *K. vulgare* was not able to efficiently utilize these amino acids for its growth, leading to its poor growth rate (Zhou et al., 2011). In this study, the improvement of amino acids biosynthesis in the evolved *K. vulgare* (as a result of serial subcultivation with *B. cereus*) could enhance the growth of *K. vulgare*, which ultimately led to the increased production of 2-KGA.

### Proteomic Profiling of the Evolved *B. cereus*

**Figure 3:**
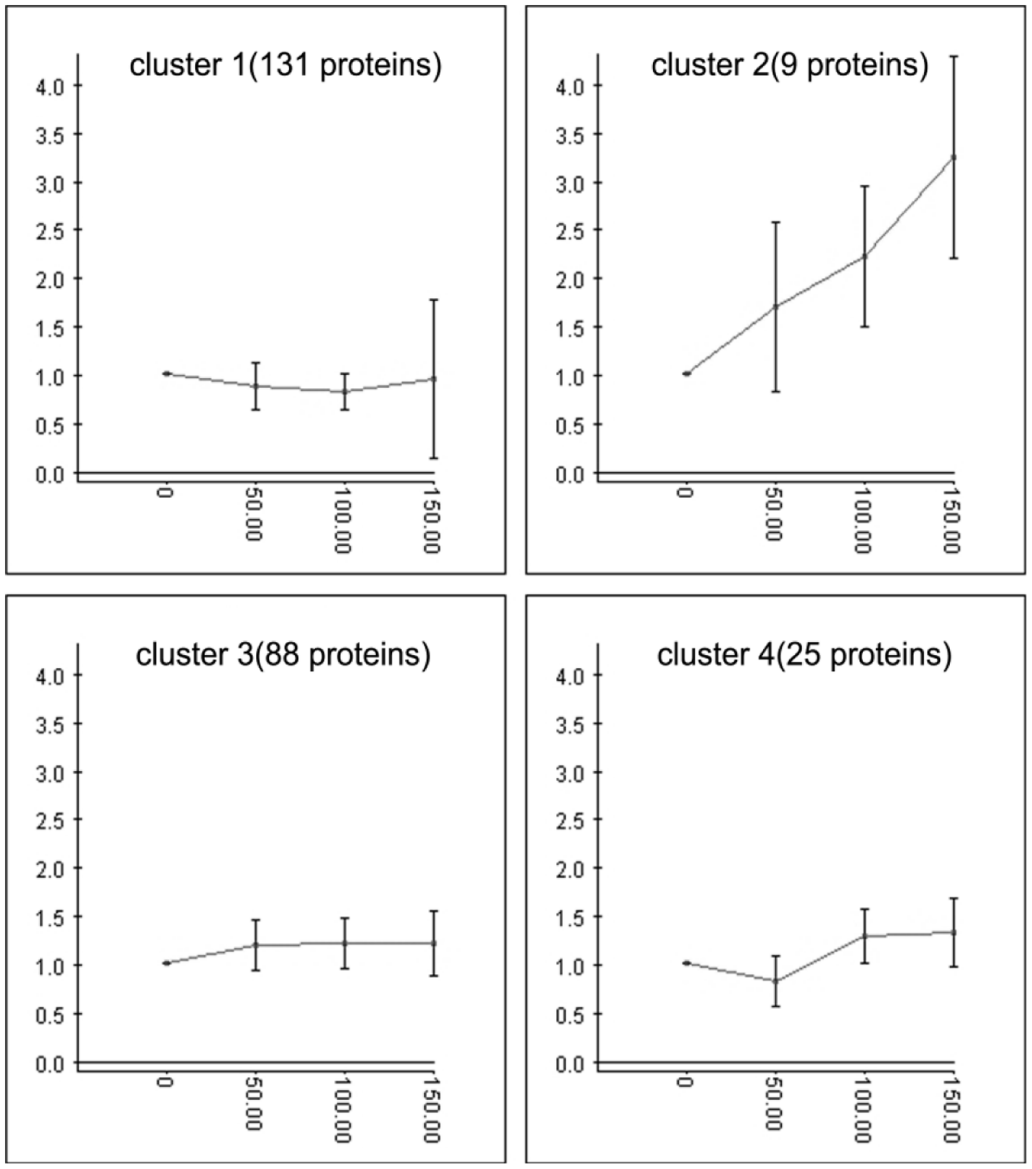
Hierarchy cluster analysis of *B. cereus* proteins: 253 proteins were categorized into 4 clusters based on expression levels using K-means algorithm with the software Expander 4.1.

**Figure 4:**
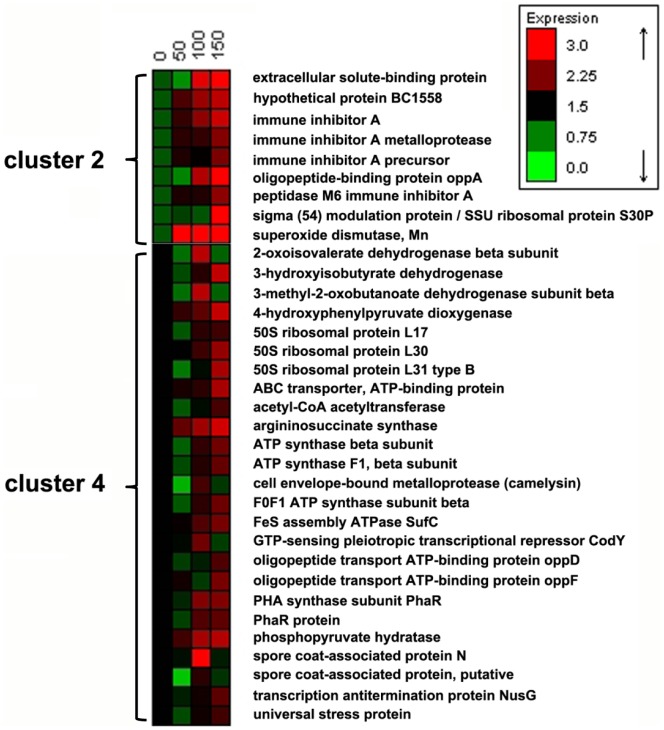
Heat map of significantly up-regulated protein expressions in *B. cereus* during the subcultivation: corresponding to clusters 2 and 4 in Figure 3.

253 proteins in *B. cereus* were quantified and further analyzed by the HCA clustering (Fig. 3). As transfers progressed, proteins in clusters 1 and 3 showed insignificant changes, while proteins in clusters 2 and 4 exhibited significant up-regulation. A heat map of protein expression levels in clusters 2 and 4 is presented in Figure 4.

**Figure 5:**
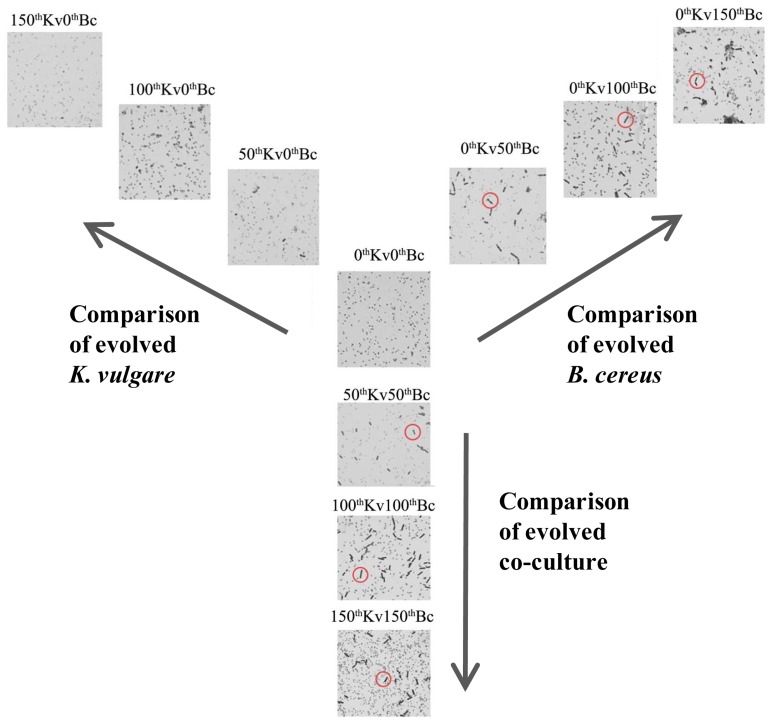
Microscopic images of the evolved strains, ancestral strains, and fully evolved strains in the industrial fermentation medium at 96 h. Vegetative *B. cereus* cells were indicted by red circles.

Intriguingly, we found that 4 proteins (among the 9 proteins in cluster 2) were significantly up-regulated, which were related with immune inhibitor A (InhA). InhA is a zinc metalloprotease, which is usually found in pathogenic *Bacillus cereus*, including *B. cereus, B. thuringiensis* and *B. anthracis* [21]. InhA degrades proteins of insect immune system and is a major component of exosporium [21], [22], a balloon-like structure notably existed in *Bacillus cereus*. InhA is both associated with the exosporium and secreted throughout the growth cycle of *Bacillus* [22], [23]. It remains unclear whether InhA is absorbed onto the surface of spores after its secretion, or assembled intracellularly into the exosporium of the developing spores [22]. Exosporium controls the attachment and colonization of *Bacillus* [22], [24]–[27]. We found the sporulation of *B. cereus* was weakened upon serial subcultivation, and the vegetative *B. cereus* existed even after 96 hours subcultivation (Fig. 5). Such weakened sporulation resulted in less total exosporium content. However, the InhA content was increased with the progression of serial subcultivation, suggesting the portion of InhA in exosporuium got increased. Considering the external immune inhibition function of InhA as a protease, together with the significant increase of InhA during the experimental evolution, evolved *B. cereus* is believed to be capable of resisting more severe conditions.

**Figure 6:**
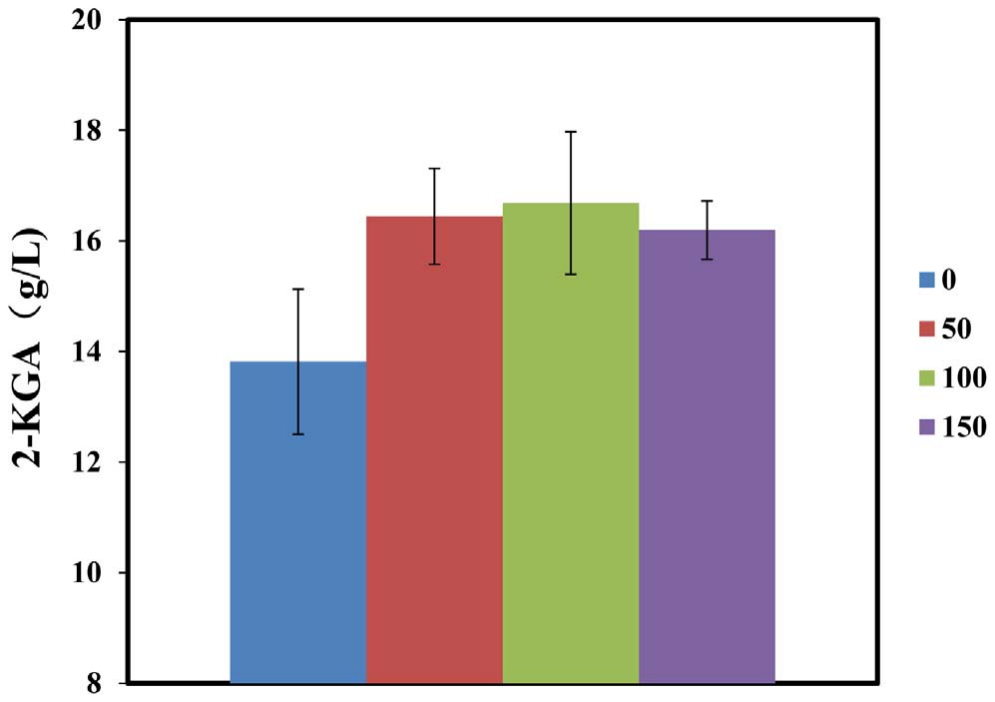
2-KGA yield of original *K. vulgare* respectively co-cultured with 0^th^, 50^th^, 100^th^ and 150^th^ *B. cereus*.

In our proteomic analyses, two spore coat-associated proteins were down-regulated at the 150^th^ transfer (cluster 4, Fig. 4). However, the other two sporulation-related proteins (stage V sporulation protein N in cluster 1, and stage V sporulation protein S in cluster 3) showed insignificant changes. The decrease in sporulation in *B. cereus* indicated that the resistance of *B. cereus* to “hunger” conditions got improved by the serial subcultivation, *i.e.,* the evolved *B cereus* was able to resist more severe nutrition insufficiency in the co-culture, as sporulation is usually initiated by nutrient limitation [18]. During the co-culture of *B. cereus* and *K. vulgare*, the production of 2-KGA would inevitably bring down the pH of the environment. As the optimum growth pH of *B. cereus* is around 7.0 [28], at the later stage of the co-culture, *B. cereus* would suffer acid stress. This stress together with the nutrient lack thus results in the occurrence of sporulation of *B. cereus*. We compared the 2-KGA yield of original *K. vulgare* co-cultured with the 0^th^, 50^th^, 100^th^ and 150^th^ *B. cereus* in the same medium as performing the subcultivation. As shown in Fig. 6, compared with the ancestral co-culture, 2-KGA yield was increased by 20.3%, 22.6% and 18.5% in the evolved co-culture (the differences were significant with p<0.05). This experiment on the one hand suggested the subcultured *B. cereus* did contribute to the increased 2-KGA production in the consortium. On the other hand, suggested the resistance of *B. cereus* to product stress was enhanced. The decreased expression of sporulating proteins also reflected increased power of *B. cereus* in resisting such stresses. Our previous study showed that the existence of *K. vulgare* promoted the sporulation of *B. megaterium* [29]. Meanwhile, the sporulation of *B. megaterium* in return helped the growth of *K. vulgare*, thus increasing the production of 2-KGA in the co-culture [15]. The current study further revealed that decreased sporulation of *B. cereus* could also promote the growth and 2-KGA production in *K. vulgare*. This result suggested that not only the sporulation process, but the growing state of *B. cereus* before sporulation also matters.

Oligopeptide binding protein (oppA) and oligopeptide transport ATP-binding protein (oppD, oppF) in cluster 2 and 4 belong to the oligopeptide transport system (Opp) [30], which helps the intake of peptides (from 2 to 18 amino acids) from culture media for cell growth and muropeptides recycling [31]. oppA, a cell surface lipoprotein, plays the role of peptide binding in the periplasm. oppD and oppF, intracellular ATPase subunits associated with cell membrane, are responsible for energy supply from ATP hydrolysis [31]–[33]. We previously found that the growth of *B. megaterium* required a large amount of amino acids, which could be supplied by the biodegradation of peptides by *K. vulgare* in the co-culture [29]. Here, we further found that the enhancement of the oligopeptide transport system during experimental evolution, suggesting that the evolved *B. cereus* could assimilate amino acids more efficiently and grow better than the original *B. cereus*. This result was also consistent with the decreased sporulation in the evolved *B. cereus*, both suggesting a good growth state of evolved *B. cereus*. Thus when cell lysis finally occurs, *B. cereus* could better supply materials deficient in *K. vulgare* to enhance each other’s cooperation [34].

**Figure 7:**
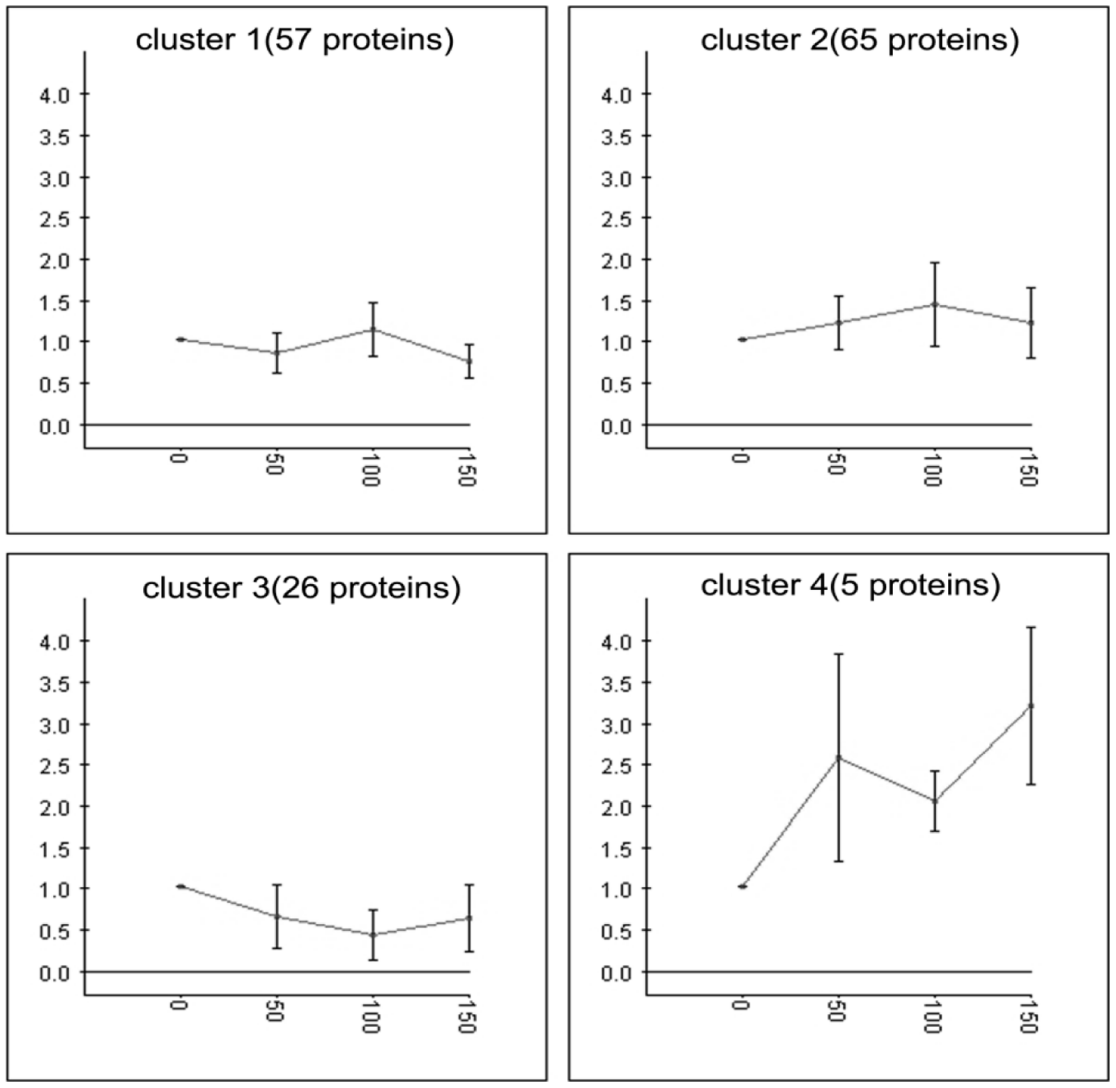
Hierarchy cluster analysis of the co-cultured *K. vulgare*-*B. cereus* proteins: 153 proteins were categorized into 4 clusters based on expression levels using K-means algorithm with the software Expander 4.1.

In the co-culture of *K. vulgare* and *B. cereus*, the growth rate of *B. cereus* exceeded *K. vulgare* at the beginning. With the lysis of *B. cereus* during subcultivation, *K. vulgare* could catch up in growth at the later stage. Most previous research focused on the companion effect of *B. cereus* to the growth of *K. vulgare*. Little attention had been paid to the influence of *K. vulgare* on *B. cereus*, although the interaction between the two bacteria was found to be bi-directional [29]. Here, we found that *K. vulgare* was a dynamic stimulus to *B. cereus* in the evolved co-culture. In the co-culture, proteins of *B. cereus* could be classified into four clusters (Fig. 7). In cluster 4, two of the proteins were immune inhibitor A, which was consistent with that in the mono-cultured of *B. cereus.* As opposed to the mono-cultured *B. cereus*, the levels of many proteins decreased with the progression of serial subcultivation (clusters 1 and 3, Fig. 7), which was probably due to the competition between *K. vulgare* and *B. cereus*, indicating the co-culture is a process both of cooperation and competition.

**Figure 8:**
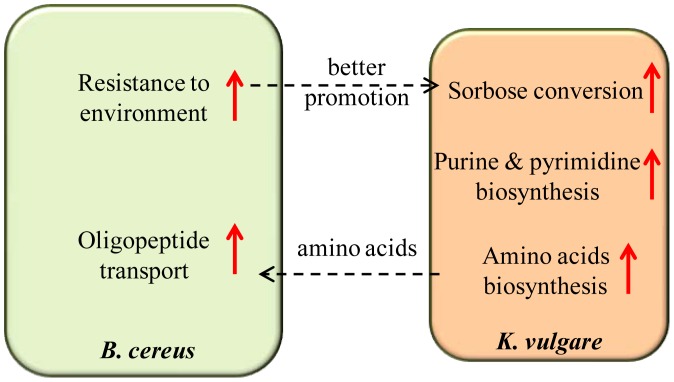
Change of interaction mechanism between *B. cereus* and *K. vulgare* upon experimental evolution. Red arrows indicate up-regulation. Up-regulated amino acids biosynthesis in *K. vulgare* provided more amino acids for *B. cereus*, and corresponding better oligopeptide transport in *B. cereus* was observed; *B. cereus* became more resistant to environmental stresses, and further promoted the growth and 2-KGA production of *K. vulgare*.

It could be concluded that after serial subcultivation of the co-culture, *K. vulgare* acquired enhanced capability of amino acids biosynthesis, which stimulated the transport of oligopeptides into *B. cereus*, leading to a better growth state and increased resistance of *B. cereus* to severe conditions, including nutrient lack and accumulation of product pressure. Thus, the promotion effect of *B. cereus* to *K. vulgare* got improved, and hence enhanced the conversion from sorbose to 2-KGA in *K. vulgare* (Fig. 8). Consequently, the experimental evolution of the co-culture led to a 16% increase in the yield of 2-KGA.

## Conclusions

We used a proteomic profiling approach to investigate the evolutionary dynamics of mono-cultured *K. vulgare*, *B. cereus*, and their co-culture during serial subcultivation. Enhanced expression of proteins involved in purine, pyrimidine and amino acid biosynthesis was observed in evolved *K. vulgare*, which could offer more amino acids for the growth of *B. cereus*. At the same time, enhanced expression of proteins involved in oligopeptide uptake was observed in the evolved *B. cereus* strain. Meanwhile, sporulation of *B. cereus* was decreased upon subcultivation, suggesting an enhanced resistance to unfavorable environments. The better growth state of evolved *B. cereus* in return could supply more deficient materials in *K. vulgare*. Thus, serial subcultivation of the co-culture enabled strengthened cooperation between *K. vulgare* and *B. cereus*, leading to an increased yield of 2-KGA.

## Materials and Methods

### Cell Culture and Growth Conditions

The original strains of *B. cereus* and *K. vulgare* were provided by Prof. Yuezhong Li (Shandong University, China). The experimental evolution procedure of this microbial consortium was conducted as described in our previous study [8]. Briefly, the consortium was cultured in the seed medium consisting of 20 g/L L-sorbose, 1 g/L KH_2_PO_4_, 3 g/L yeast powder, 0.2 g/L MgSO_4_, 3 g/L beef extract, 1 g/L urea, 10 g/L peptone, and 3 g/L corn steep liquor, and was transferred to new medium every day. Cells in the 0^th^, 50^th^, 100^th^ and 150^th^ transfers were obtained for liquid nitrogen storage, which were subjected to comparative proteomic analyses.

### Sampling, Extracting of Proteins and iTRAQ Labeling

Six groups including 24 cultures (with each sample having two biological replicates) of cells were respectively harvested at the exponential phase (about 10 hours after inoculation) for protein extraction, by centrifugation at 5000×g for 5 min at 4°C. The pellets were washed with phosphate buffer solution and subsequently with distilled water. Then the pellets were stored in liquid nitrogen for later protein extraction. The extraction procedure was according to the method we used before [15] [16]. Briefly, the cells were grinded in liquid nitrogen, resuspended in cell lysis buffer (8 M urea, 4% m/v CHAPS, 40 mM Tris-HCl, and 1 mM PMSF), and further broken by pulsed ultrasonication on ice. Then a centrifugation at the speed of 12000×g was applied for 40 min at 4°C, and the supernatant containing intracellular proteins was further measured for protein concentration using Bradford method [35]. 100 µg proteins were further purified by precipitating in 80% acetone at −40°C overnight, ready for the next step of iTRAQ labeling.

Using the method of iTRAQ 4-plex labeling quantification, four generations of *B. cereus*, *K. vulgare*, and the mixture of *B. cereus* & *K. vulgare* could be respectively compared in one group of labeling. Thus, in all, six groups of iTRAQ labeling were conducted. The labeling procedure was conducted following the manufacturer’s instructions (AB Sciex, Foster City, USA) with minor modifications. Briefly, protein pellets were resuspended in TEAB buffer, reduced, digested by trypsin and labeled with isobaric tags. Six groups of samples were labeled respectively, and in each group the 0^th^, 50^th^, 100^th^ and 150^th^ generations of cells were labeled with iTRAQ tags 114, 115, 116 and 117, respectively. Then the labeled samples from each group were pooled, dried on a speedvac and resuspended in the strong cation exchange (SCX) buffer A consisting of 5% acetonitrile in water and 0.1% formic acid, ready for the subsequent LC-MS/MS analysis.

### On-line 2-D Nano-LC and Mass Spectrometry Analysis

Each group of peptides labeled by iTRAQ reagent was analyzed by 2-D nano-LC (Agilent Technologies, USA) tandem mass spectrometry (micro-Q-TOF II, Bruker Daltonics, Germany). A two-dimensional separation method was applied to these peptides. Specifically, the two dimensions consisted of the first dimensional separation on a strong cation exchange column (ZORBAX BIO-SCX II, 3.5 µm, 35×0.3 mm), and the second dimension of separation on a C_18_ column (ZORBAX 300SB-C_18_, 3.5 µm, 150 mm×75 µm). The first dimensional separation was achieved by increasing concentrations of ammonium chloride (NH_4_Cl) solution at the flow rate of 10 µL/min as described before [16]. The second dimension of separation was conducted using a 120 min linear gradient ranging from 5% to 40% mobile phase B (mobile phase A, 5% acetonitrile in water, 0.1% formic acid; mobile phase B, 5% water in acetonitrile, 0.1% formic acid) at a flow rate of 300 nL/min. A mass range of 70–2500 m/z was acquired by micrOTOF Control 3.0 (Bruker Daltonics, Germany), and the five most abundant precursors were further fragmented for MS/MS analysis.

### Protein Identification, Quantification and Data Analysis

Spectra pretreatments were conducted using DataAnalysis (Bruker Daltonics, Germany) as described before [16]. Database searching for protein identification was performed using the Mascot server with the threshold set at *p*<0.05. The database for *K. vulgare* was an in-house protein database with 3179 sequences obtained from our genome sequence results (File S1), and that for *B. cereus* was a database consisted of all the bacillus species from NCBInr database. When performing the group of mix-culture protein identification, a combination of the above two databases was used. The searching parameters were set as follows: trypsin as the cleavage enzyme; one maximum missed cleavage of trypsin; fixed modifications including MMTS modification of cysteine (Methylthio (C)), iTRAQ (N) and iTRAQ (K); variable modifications consisting of the oxidation of methionine and iTRAQ (Y). The mass error tolerance for precursor ions and fragment ions were set to 0.5 Da and 0.1 Da, respectively. The quantification of proteins was conducted using the Mascot server by performing the “iTRAQ 4-plex quantification” and three biological replicates were required. InterPro (http://www.ebi.ac.uk/interpro/) was applied for protein sequence analyses and classification, and the Kyoto Encyclopedia of Genes and Genomes (http://www.genome.jp/kegg/pathway.html) was used to reconstruct major metabolic pathways. Hierarchy cluster analysis (HCA) using the tool of Expander 4.1 was applied to each of the labeling group to further analyze the protein quantification data, giving an overall view of the feature of the dataset and seeking the variation pattern differences.

## Supporting Information

File S1 Protein sequences of *Ketogulonicigenium vulgare*.
(DOCX)
