# Supplementary material for: Comparative Proteomic Analysis of Experimental Evolution of the Bacillus cereus-Ketogulonicigenium vulgare Co-Culture
Source: PLoS One. 2014 Mar 11;9(3):e91789. doi: 10.1371/journal.pone.0091789 (PMC3950281; doi:10.1371/journal.pone.0091789)
Supplement: File S1 — Protein sequences of Ketogulonicigenium vulgare. (DOCX) [file pone.0091789.s001.docx]

>gkv_1|gene_xdhA|xanthine dehydrogenase, small subunit|

MNMEISFLLNGERARVTAEPTRTLLDWLREEQGLKGTKEGCNEGDCGACTVMVTDEAGPRAMNACILFLP

QLEGKAVRTVEGVAAPDGSLSPVQQAMVDHHGSQCGFCTPGFIMSMTAAHLVGARDHDDVLAGNLCRCTG

YAPIIRAAEAVADAPAPDWLHDTPLETVPQSLDAFAAWYEQNPDATLVAGATDVGLWVTKQLRDLPKVAF

LNRLTELQQIELSDHEIRIGAGVTMTRLLPVLRQYHPSYAEMVRRYASHQVRNAATIGGNVANGSPIGDN

PPALIALGATLHLRQGAQRRQMPIEDFFIAYGKQDRRGGEFVEAITIPTKAPALRVYKLSKRFDQDISAV

CAAFNVTVEDGVVSEARIAFGGMAATPKRASHVEGALIGKAWADAAQAAALLAQDYQPLTDMRASAAYRL

QAAEGLFLRYIDDINGKPAHVLEVSA*

>gkv_2|gene_xdhB|xanthine dehydrogenase, molybdopterin binding subunit|

MSVAKSLPHDAATLHVTGKARYTDDIPAPRDALHLAFGLSTIARGSILSMDLTAVRAAPGVVQVITVDDL

PFDNDVSPGWHDEPLLATGRVNHIGQPLFIVVATSHLAARKAARKAKVEYQAEEPILTIEQALAADSRFE

KAAMVWTKGDAPAALQTAPHRLTGSLSMGGQEHFYLEGQVAMAWPQENGDMLVNCSTQHPSEIQHKVAEA

LGTHMHGVRVETRRMGGGFGGKESQGNALAVACAIAARATGRVCRMRYDRDDDMMITGKRHDFRIDYTVG

YDDTGRILAIDVTHYTRCGWAQDLSLAVADRAMLHAENAYLIADMRITSHRLKTNMQSATAYRGFGGPQG

LFGMEQIIAHLSHTTGIDAITLRRRNYYQPMEAAGVDNTTPYGMQVTDFILQDMTDRLMTTSDYASRRAA

VDEWNAQNDRLKKGLAFSPVKFGISFTATQFNQAGSLVHIYQDGSVSLNHGGTEMGQGLFQKVAQIAAKS

LGIDIDRIRITATDTAKVPNTSATAASSGSDLNGAATHIACETLRTRMAEALGPLYQTNPTDVRFVDDQV

HFTGQSITFPEAARICYNQRVALSATGHYKTPDISWDRIKGQGRAFYYFAYGCAVTEVVLDTLTGENRIL

RADILHDTGASLNPVIDIGQIEGAYVQGAGWLTTEELVWDDKGMLRTHAPSTYKIPACSDRPRIFNVDLV

NRPAPLPTIYRSKAVGEPPFMHGISAFLALQDAVCACGPTWPELRAPATAEAILTAIGRTRS*

>gkv_3|gene_xdhC|xanthine dehydrogenase accessory protein XdhC

MSLDLAALTRAVAQHLRLVRVLVLKHAGSVPRETGTSMLVTTNGLEGTIGGGRLEDEAIRAARALLAEDA

APRLQTYPLGPRLGQCCGGSVTLLYEVFTAQNLPSALPYARPIDPAAPPPAQLTPLPDGTPRIEAGWIIE

GPPPQNRPLWLFGAGHVGRAIVSILAPLNDRQITWVDTSADRFPDTPANVTPLIAADPARVVPYAPAHAD

HLIMTYSHDFDFALCHALLSHGFHSAGLIGSDTKWIRFQRRLATLGHSDAQIQRITCPIGDPALGKPPQA

IAIGVVYALLKGKELQSQ*

>gkv_4|gene_NONE|ABC transporter family protein

VSDVLLGLSGLTKAYPGVVANRDISFQIAKGEVHALLGENGAGKSTLVKTIYGLVRPDSGTMQLDGKPYH

PAEPRAARAAGVAMVFQHFSLFEALTVAENVALGMENPPPMAALAQRITEISNAYGLPLSPERTVGDLSA

GERQRVEIIRCLLQDPQLLIMDEPTSVLTPQEVSTLFETLRRLRAEGRSILYISHKLEEIRAICDSATVL

RHGEVVGSCDPRTTSARDMAEMMVGASLQTPSRAPVLAGEIALELKSLSLPSPRAFGTPLHNVSLILRRG

EILGIGGVAGNGQDELLLALSGEMRSPAGMIWLNGSDVGTSRPNDRRKLGLLAAPEERLGHAAAPDMSLS

ENALLTGALREKLVDGGFINWAKTTAFARRIIETFDVRTPGPHVAARALSGGNLQKFVVGREILQNPAVL

VVNQPTWGVDAAAAAAIRQALLDLAARGAAVVVVSQDLDELMEISDRFAALNAGHLSEARDARGLGIDDI

GLMLGGAKAEVAA*

>gkv_5|gene_NONE|branched-chain amino acid transport system / permease component family protein

MIRLEKRPTHSHVWTWATPLIAVLATMIAGGLMFMLLGKDPFATLRTIFWDPLFGQHASYYRGQLLIKAG

PLILIAVGLSFGFRAGIWNIGAEGQYIVGAICGAAVALAFYPAAGWYIFPLMVIAGALGGMAWGLIPGIL

RVRYGASETLVSLMLVYVAERLVAQMALGAMRNPEGMGMPGSRVISRYPAAANTEIFAGTGMHWGVVAAL

IAVIVAYVALSRHMFGFNIRLAGQSPRAAAFSGVQPGKLILICTGLSGALAGLAGMFEVAGPAGRISIDF

GVGYGFTAIIVAFLGRLHPVGILLAGLLLALTYIGGESAQTAIGLPAAAIQMFQGMLLFFLLAFDVLTNY

RLRLARPKAKEVA*

>gkv_6|gene_NONE|branched-chain amino acid transport system / permease component family protein

MDLSAINPVLLIASLMVASTPILLAAIGELVVEKAGVLNLGVEGMMITGAICGFIAAHHSQSVTLGYIGA

AAGGAALALVFALLTQFLLSNQVATGLALTLFGLGFASLAGQGYNGIKAPTSYRLDIPVISDLPVIGPIL

FSHDIMVYVSLLLCLGVWIFLNRTRAGLILRAVGENHDAAHALGYKVVLVRCLAILFGGACAGLGGAYLS

LVRVPQWTDGLTAGAGWIALAIVVFASWRPDRLLIGAYLFGGITVLQINLQLARVPVPVSLLAMAPYIVT

ILALVLISRGRRSSASPAMIGRPFHATQ*

>gkv_7|gene_NONE|basic membrane family protein

LKRRTLLASGAAAAVLAGLGLPARAQAPVKVGFIYVGPIGDGGWTFQHEQGRLALLEYFGTAIETVYQES

VPEGADAERAITQMALAGCKLIFTTSFGYMEATMAVAAQFPDIYFEHCTGYMRAENVATYDARFYEGRAV

MATIAGRMTQTNKIGYIGSFPIPEVIQGINSTYIHAKKVNPDIELVVAWAYSWFDPAVEADAATAMIEQG

VDVILQHTDSTAPQAAAQTKGGIVTFGQASDMSAFAPFPRVSAIIDNWAPYYIKRVQAVIDGTWESTATW

GGIAEGEVGIGEITDAVPAEVKAEAEALRDAIAAGTYHPFTGPLNRQDGSAWLAEGETASDEDLLSMGFY

VEGITAQIPS*

>gkv_8|gene_NONE|bacterial SH3 domain protein

MKTVTSISILMQIAALALCLALPAAAQDSDIPSPPPRPDASAPAQDADADAIAAAVAGAAPVEAPVAAPA

EPPPPPPTPASGTPEPPEPTGPQLGASTNLPVPRYVSLRSNEVNVRRGPASSQRVDWVFHRAGLPVQITG

EYEHWRRIIDRDGEGGWVHYALLSGNRTVIVQAELLPVLAQPEANAPVIAQFENGVIADLDECRPDWCRI

GAGGYRGWVMKSALWGVDPTEIRN*

>gkv_9|gene_NONE|cation diffusion facilitator family transporter family protein

MSKSDDNNRLNLSAGVLSVSVASLLVLGKLWALQATGALSIAATLADSALDLLMSLGGLIAIAYAAKPAD

KDHHFGHTAVEDLTALAQSLVITASAVLIALAAIRRMAAGDTDVIGAQGIGMVIMVLSIVLTLGLVAWQR

HVARRTGSRVVAADSLHYIGDLIPNIGALIALAVSALWGIGAVDSVIALLAAGLMLRGAAKIGKQAWDAL

MDRSAPPDVIATIEGVARDFDGIIGYHDLKTRTSGSRIFVTLHIEMDGNQTLFAAHRTSAALRRAIVRAL

PNADVMIHKDPFGAPPHPDDERQQ*

>gkv_10|gene_NONE|bacterial extracellular solute-binding proteins, family 3 family protein

MMKALFTSLGLALIATAGAAQTLPDLQGREITVATENAYPPLQFVGPDGAAIGWEYDAMDEIAKRLNLTV

TYGNISWDAMIPAVSEGQFDIGMTGITIREDRMEMVDFSDPYLTSQMLMIVRGDEDRFVDAASFAANPDL

LMSAQPGTTPFYVGVYDVLDGDEANPRIVKFETFGAGLAALRTGDVDLALSDSTAAHGYVNTSDGALKII

GEPLGTEDFGFIFPKGSDLVTAINAAIASMEADGTLDALSTKWFFEYGQGE*

>gkv_11|gene_NONE|amino ABC transporter, permease protein, 3-TM region, His/Glu/Gln/Arg/opine family domain protein

MRRAKSDKDFPYWLVILGVTGLWLFWRVATDDVYAGVLNTLSRGLGVTIMVTLIAFSGGAVLGLGLALAQ

LSRSLILRQAARLYVEVMRGVPIMVLLLYVAFVGVPGMVALWNGLTGWTGIEPLRTRDVPLLWRAIIALL

LAYASFLAEVFRAGILSVEVGQIEAAKALGLGRWHIFRHIVWPQAMRNVLPPLGNDFVAMVKDSSLVSVL

GVTDITQLAKLTSASNFRYFETYNVVALIYLTVTIGLSLALRRLEERLRARDQR*

>gkv_13|gene_NONE|peptidase family M3 family protein

MTNPLLGPWRAPYDLPPYDLISDADYAPAIDAALDTARATVAAIADNSAAPTFANTIAALEMADEQLGRV

LSAFYTVAGADSNPAREALQRDLAPKLSAYSSEISSNKALFQRIDQLWTDKDALNLSDEEERVLMLTRRG

FVRSGAGLSGAAEAEMKDVKSRLAVLGTTFTQNLLADEREWFMPVTEADLAPLPAFLTAALRAAGAQKQA

DGPVVTLARSVITPFLQYSPNRALRQRAYEAYVARGANGGATDNRAIAAETLALRQIRARLLGYDTFAAF

KLETEMAGNAQNVRQLLLDIWGPARKAAQSESTALEALLHADGIQGPLEAWDWHYYSEKRRLAEHDLDEA

AIKPYFQLDRMVEAAFAVANRLFGLEFEALDTPFYHPDCRVWKVTRGGEWVAIFVGDYFARGSKRSGAWC

SAMRSQSRVGGVAQRPVVMNVCNFAPPEAGQPALLSYDDARTLFHEFGHALHQMLSDVTYESISGTSVAR

DFVELPSQLYEHWLEVPEVLAEFATHAETGEAMPAALIERLLAARNYGQGFSTVEIVSSALVDLEFHDGP

APQDPMQKQAEVLESIGMPHAIRMRHATPHFAHVFSGDGYSSGYYSYLWSEMMDADAFDAFLESGDPFDP

ATAKSLEENILSKGGSADAAALYTRFRGRLPGVESLLKGRGLI*

>gkv_12|gene_NONE|moeZ/MoeB domain protein

VTDRRFTPDEVARYGRHITLREIGGPGQKALLRARVLIVGAGGLGSPVLQYLGAAGVGQITVVDDDLVEA

TNLQRQVIHTAASVGTPKVASAQAAINAQNPHIHVTTVQTRFSAQNAAELVAQHDLAIDCCDDTATRNAL

NAACVAAGIPMVSAALTTWEGQVSLYDPARGGPCLTCVFPHAPPVIEDCSVVGVFGPLPGIIGTMMAAEV

VKALTGAGEGLRGRLMIYDALYADMRIIRTKADPACPTCHGMGAVK*

>gkv_14|gene_dut|deoxyuridine 5'-triphosphate nucleotidohydrolase (dUTPase) (dUTP pyrophosphatase)|

MTLTIKFTRLDGYDDAVALPSYQTPGAAGADLRANFGADLRGIGLTLPPMGRALVPTGLAIEIPTGFEVQ

IRMRSGLALKQGLMLPNAPGTIDSDYRGHLGIIIMNGGDQPVTIAHGDRIAQMIVAPVVQAQFELVTALT

QTDRGAGGYGSTGVSQ*

>gkv_15|gene_coaBC|phosphopantothenoylcysteine decarboxylase/phosphopantothenate--cysteine ligase|

VLVGKRILMIIGGGIAAYKALELIRLLRGQGVEVVPVLTRAGAEFVTPLSVSALANTAVHQDLFDLTHEA

EIGHIQLSRAADLVVVVPATADLMAKMAQGMANDLASTLLLATDKRVLMAPAMNVRMWDHPATQRNLRTL

EADGVLRIGPDDGAMACGEFGPGRMAEPLAILAAISDALQPADKPLDGNHILVTSGPTHEPIDPVRYIAN

RSSGAQGTAIAQALLDLGAKVTFITGPAEVPPPAGADVVRVETASQMRAAVQAALPADAGVFAAAVADWH

VVGAGPSKIKKVAGQLPQLNFAENPDILAEVGHMTAGRPRLVVGFAAETDDVIAHARAKRLRKGADWIVA

NDVSPATGIMGGAENEITIITAEGETALPRMSKPAVAKELARRIADTLLKDDA*

>gkv_16|gene_NONE|conserved hypothetical protein

MDAKHTLLAERLLKKPASDFSDSDRRVLRHMLDRAPVSQDPALRADTLGARVADKVASFGGSWTFIMLFG

GVLVTWVVLNGVLLASPPDPFPFIFLNLMLSMIAAFQAPIIMMSQSRQAQKDREAAANDYEVNLKSELEI

MRLHEKLDDLRLVALEDQLRLITNKIDAIAAAVNRP*

>gkv_18|gene_NONE|competence protein comM

MLARCYTVAFEGIEPRLIEVQCALAPGIPGFSIVGLPDKSVSESRERVRAALAAMALAMPAKRVTVNLSP

GDMPKEGAHYDLPIAIALLAAMDAISRDAAESVLAMGELALDGRLVPVAGALPAALASASEDKALILPAP

SAPEAAWVAAATVFGAESLGSALAHLAGTTPLAPARPPLSAAPAAVKSTDLASVRGQEGAKRALEIAAAG

SHNILMLGPPGAGKSLLASCLPGILPPQTADEALESAMIQSVAGTRGAGPSPHRPFRSPHHGASMAAIIG

GGRRAAPGEISLAHNGVLFLDELPEFQRPVLDSLRQPIETGEVWIARAEAHLRYPSRFMLVGAANPCRCG

HMADASRACARAPRCGQDYMARLSGPLLDRFDIRIDVPAIPPSDLRGPLGEPSSEVARRVTAARKRQIQR

LEPFGARSNADCPPDVLEEMTALDGDCAAFLQRAAAHFGISARGYSRILRVARTIADLDGAEQITRPHLS

EAIALRGP*

>gkv_17|gene_gshB|glutathione synthase|

MALKVAIQMDPIESINIDGDSTFRIALEAQARGHSLFYYTPDKLAFQEGRITARGWPITLRREKGNHVTK

GDEVEVDLADYDVVWLRQDPPFDMGYITTTHLLDMIHPKTLVVNDPFWVRNYPEKLLVLNFPELTPPTAI

ARDLDTLRAFRAKHGDVILKPLFGNGGAGVFKLTADDSNLASLHEMFTSISREPLIVQKYLPDVRKGDKR

VILVDGEPVGAINRVPASGETRSNMHVGGRPEKVELTARDLEICAKIGPLLREKGQIFVGIDVIGDYLTE

INVTSPTGIQELERFDGTNTAEKIWEAIERRRAA*

>gkv_19|gene_NONE|uncharacterised protein family UPF0102 family protein

MTDLRMTGGLRNHLQGAAAEAAVLRHYEALGARLLHSRWRGRAGEIDLILQQGDLTIFVEVKSAASFDRA

AFSIQPGQAARILRAAEEFMAGRLADLRIDLALVDGQGHVHVIENAFFA*

>gkv_20|gene_NONE|tetrapyrrole (Corrin/Porphyrin) Methylases family protein

MSGAFPLTAGLYLVATPIGAARDITLRALDVLRLADVIAAEDTRTARKLMEIHGVPLNGRRILAFHDHST

EGTTAHLVTQVREGKSVAYVSEAGTPLVADPGYELARGMIAEDLPVTAAPGASAVLTALTIGGLPTDRFL

FNGFLPAAHSARQSELAILRDVPATLVFYESPKRLGSSLVDMAAVLGAGRQAAVCRELTKKFEEVRRGTL

AELAEYYQENDARGEVVVLVDRAGAVVTGAADIESALREAMQTMRIKDAATLVAGALNLPRREVYQIALA

MSADD*

>gkv_21|gene_NONE|ABC-type branched-chain amino acid transport systems periplasmic component-like protein

MRKLMTRRSLGRMAAAVAMAALAACAPTVNTGGGTTPAATGGTVRVALLVPAGSAQATDNLIAQNLENAA

RLAISDLGTSRIDLRVYPTGGSATQAATVARAAVSDGAQIILGPVYAEEANAAGVAVASSGVNVLAFSNN

PTIAGNNVFILGQTFRDTANRLVGYSSSQGINSYVIAHANDLGGNLGRDAISAAITAGGGTVAGVESYAL

SQAATAEGARRVGATVNATGAQAVITTASVNADLPILATVLPEAGVSRETTRLIGLTRWNATSQALTLPG

LQGGIFTLPDQARVTAFEGRYSATYGQPPHPLASLAYDGIAAVGALLQRGGSNPLSRGALTQTQGFEGTS

GIFRFLPDGTTQRGLAVAEIRNNMVSILDPAPRSFGGPQS*

>gkv_22|gene_glnD|protein-P-II uridylyltransferase|

MIRPAHEIFDAPAITARLLAALDGVADPMDMRRATVAILSDAMANGRAVIATAFAANPLESRGVTHAYAW

LTDCIVTLALWVARGPMQPTPAAGDLTMVAVGGYGRGEMAPFSDVDLLFVTRANMSRETEAVIESLLYTL

WDLKLKIGHSSRTVDDCIAMAKQDFTIRTAMVEARYLDGNTTVFQTLRSRLWNELFKNTASEFIEAKLDE

RAQRHVKQGGQRYMVEPNVKEGKGGLRDLQSLYWIGKYVYNVQDAAQLVQKGVFTADEYETFRLAEDFLW

AVRCHLHLIAKREVDLLTFDMQVEVASRMGYRDSRGRRAVEHFMQDYFLHATKVGELTRIFLTVQEDKLI

KPAPMLSRLFARKRPVAPPYALKTNRLTVADPKAFLADKLNLLRIFEESVRMGAPIHPDALRLVAANLNL

IDDDMRESRSANRIFLDLLLRHGNPARILRHMNEIGVLAAFIPEFEPIVAMMQFNMYHHYTVDEHTIQCI

SNLAAIEAGELADDLPLVSDITKAGISRRVIYVALLLHDIGKGREEDHSVLGAQIARRVAPRLGLSKRES

ETVEWLIRHHLMMSDTAQKRDIAEPRTVRAFAKMVESRERLDLLTVLTVCDIRGVGPGTWNNWKAALIRS

LHRSTAEVLASGPDTLSRRTREDEAKRALREALHDWPAADLRAETGRHYGPYWQGLPLSAHVVFANLLRG

INDSEIRIDLTPDPDRDATRASFAMSDHPGLFSRMTGALALVGANVVDARTYTTKDGYATATFWVQDADG

RPFEAARLPRLRQMIDRTMNGEVVPREAMKERDKIKKRERAFTVPTLITFDNEGSDIYTIIEVDTRDRPG

LLHDLVRTLAAQNANIASAVIATYGEQAVDTFYVKDMFGLKFHAEGRRQQLEAKLREAIKQGAERAHS*

>gkv_23|gene_mviN|integral membrane protein MviN

VTTETSRKPSGASSPIRLATNFVTVGVWTFLSRVLGFARDIMMAAYLGTGPVAEAFAVAFTLPNMFRRFF

AEGAFNLAFVPMFAKKLEAGEDATGFARDAYAGMAFILTIFSVIGMLIMPVLVWLMASGWVGDARFSLAT

AYARVTFPYILLISLTALLSGILNAAGRFRAAAAAPALLNLTFIPAIVIGAHFDALPGGGDGVRIGWAMA

WGLPVAGILQLATLWWAARRAGFTMTIKRPRLTPELRQLAIIAMPAVLAGGVVQINLLVGRQVASYFEGA

YNWLSYADRLYQLPLGVVGAAIGVVLLPELSRRLAAGDDKAGKQAMNRATEFALMLSVPAAVALVVIAVP

LISVMYERGAFTSADTAATALALAVYGLGLPAFVMQKVLQPLFYARADTRSPFRFALVSLVVNAVAAVAL

APFIGFIAAAVGTTVAGWGMVLQLWLGSRKMGAAAEVDAQLRTRFWRICAAAAIMGVFLAIAYALLNPML

EPGRMRYLALMILVFGGMGVYFVAGQLLRAFDIREVRAMLRRSKGR*

>gkv_24|gene_NONE|RNA modification enzyme, MiaB family protein

MSQNPPDLRPDLAPRPRFDTAPRPGQPTIGMVSLGCPKALVDSERILTRLRAEGYAISPDYAGADAVIVN

TCGFLDSAKAESLEAIGEALQENGKVIVTGCLGAEPEYITGVHPKVMAVTGPHQYEQVLDAVHVAVPPSP

DPFVDLLPAAGIKLTPRHYSYLKISEGCNHKCKFCIIPDMRGRLVSRPAFAIVREAEKLVQSGVKELLVI

SQDTSAYGVDLKYATERGHRAHITDLARDLGSLGAWVRMHYVYPYPHVRDMIPLMADGLILPYLDIPFQH

AHPDVLRRMARPAAAAKTLDEIAAWRDICPDITLRSTFIVGYPGETEAEFQTLLDWLDEAQLDRVGCFQY

ENVAGARSNLLPDHVPDEVKQDRWNRFMEKAQAISAAKLQAKVGQQMDVIIDEIEDGVATCRTKADAPEI

DGNLFIDEGVERLSVGDIVRVTVDEAEDYDLWGTPV*

>gkv_25|gene_NONE|peptidase family M48 family protein

MTAKALRPRSFIALIAVLLLAACAAPSRDTAPTGRVTTGVSHQGAAPLSPADMSLRAQGFIYAASQVEPV

AERICRERAPQRNCDFQIVIDDQSDIGPNAYQTLDRSGRPIVAITLPLLFMVANSDEIAFVLAHEAAHHI

EGHLERRSQSVAQLGEILGSMVYSKEFELQADALGTLIAAEAGFDPLRGSALFFRIPDPAGRILSSHPAN

AERLDVIRRTYANYMARRS*

>gkv_26|gene_NONE|conserved hypothetical protein

MDMTELAVPGTEIAVRVTPKASRARILRDESGVLRVYVTVVPEDGKANAAVTELLAKALRIPKSKLILKS

GATARDKVFRLL*

>gkv_28|gene_NONE|nitroreductase family protein

MTQSALEFLLQRRSHPARLLSLPVPDADQLQTILTAAARTPDHGKLEPFRFILLRRGALDRLAALAGARG

AALGKEEGPLLKAVTQFSQSPFAIAVVQVPRPTDKVPAIEQTYTAGAVALALLNAAMASGFAANWLTGWT

AYDPTFLAEGLGLQLGESLVGFVHIGTSTSTPPERPRPDLAQIVSELPE*

>gkv_27|gene_NONE|conserved hypothetical protein

MTITSVFVVFAVSWFLTLFIVLPIGLRTQGDVGEIVPGTPASAPADFNAKRTMLLTTIWATVVWAVVCGI

ILSGVIKVRDFDFFHRMNPPIERPEAQVIERRDHTAH*

>gkv_29|gene_aspS|aspartyl-tRNA synthetase|

MHAYRSHTCAALNAANVGQNVRLSGWVHRVRDHGGILFIDLRDHYGITQVLVDPDSPAFSAVEKVRAEWC

IRIDGLVKARDASLVNPKLATGEIEVFVQDIEVLGAAAELPLQVFGEQEYPEETRLRYRYLDLRREDMQR

KMTLRSDVVASIRQRMWGKGFREYQTPIITSSSPEGARDFLIPSRLHPGKFYALPQAPQQFKQLLMVSGF

DKYFQIAPCFRDEDPRADRSPTDFYQLDMEMSFVTQKDVFDTIQPVLSGLFEEFGGGRKVDTEWPQISYK

DAALWYGSDKPDLRNPIKMQVVSEHFAGSGFAVFAKLLEQDGTQIRAIPAPTGGSRKFCDRMNVWAQKEG

LPGMGYIFWRTGDNGEMEAAGPLAKNIGPERTEAIRLQLGLGLGDAAFFLGGKPAVFEKIAGKARNEIGN

ELGLTEKDRFAFAWIVDFPLYQQDAETGAYEFEHNPFSMPQGGLEALNGDPLAVRGYQYDLACNGYELVS

GAIRNHQPEIMFKAFEIAGYGPDEVRRRFGGMVNAFTYGAPPHGGCAAGIDRIVMLLADEQNIREVILFP

MNQRAEDVMMGAPNEPTNEQLRELRLRVLPPEA*

>gkv_30|gene_NONE|acetyltransferase (GNAT) family protein

MRILDGTESDLPAIVAIFNDAVVNTTAIWSDVLATVEARKTWMEQRRAGGFPVFVAKNEADEVVGFASYG

PYRPFDGFRLTVEHSVYVRPDQRGKGVGGKLLDALITHARGAGLHVMVGGITADNAASITLHERRGFQQV

GLLPQVGVKFGRWLDLAFLQLKLDDLPHPKAD*

>gkv_31|gene_NONE|glucans biosynthesis protein G

MTRRNLLKSTALSAMILPLASGVFAQEASGDLPDGPMPFSFDRLSDEMRTLATQPHVVETVDDGFLNTLT

YDDYRAINFLPERARGAAEHLPFQIHSFHMGWLFPSPVRMFEVTGEEARPMIFSSDDFEYRGELAARVPQ

HFDLPGVAGFRLHYALNRPDIMDELIAFLGASYFRALGRGNSYGASARGLAVNTGASEGEEFPTFTRFYI

DRGTDPSRIVVYAALESVSLTGAYRFEINPGEDTVIGVTARLYMRRDVSVLGVAPLTSMFLYSEKNRAEF

DDYRPSVHDSDGLKLIRNGGDVLWRPLNNPPRLSGSFLGEENPRGFGLIQRDRDFENYQDAEAYYERRPS

IMVQPRGDWGKGYVRLLEIPTDLETNDNIVAFWVPEAPARAGDALEFSYDLIWGALPENPDADLAYVHEM

RAGHGGVSGVEHDGASRKFVIDFRGGMMARLPADDQNFEAVVHVTGGETTHMHHERLAANGDWRLVLDVT

PTGDGPVELSAHFAGYDRKLSEVWLYQWVRA*

>gkv_32|gene_NONE|hypothetical protein

MKDLMDVNTMDTTKAEAQFNALPPVAPLAMPRQVLVPTQRSLRETLAEWAQMALRFGKHISGARS*

>gkv_33|gene_NONE|glucans biosynthesis glucosyltransferase H

MDAFRLWLLAAGRGFQILAALVAGGGGFYLFLQFGASDGIDAFDILRAGLIFISTAWLAIGASGAFLGLL

TRAPKQPETSGPLSTRTVVLMPMYNEDPRETFARLAAMDESLQATGYGASFDIAILSDTRDDARAAQEWV

WFHHLLDVRGGHGRMFYRRRDENSGKKAGNIESFIATSGAAYDFAVILDADSLMEGETIVALARRMEAAP

DMGLIQTLPQIVNAKSRFGRGQQFAAAFHSPVFARGQAMLQGRTGPFWGHNAITRVNAFAESCGLPVLPG

KAPFGGHILSHDYVEAALLARNNWTVRLDDDLGGSYEEGPENIVDHAKRDRRWCQGNLQHSKLILAPGLK

GWSRFTFLQGILAYISPLFWLAFIIASILAPLTATNQPNYYPNEYWQFPVLPPDQTSKAIGLAVGVIGLL

IMPKMLVWLKAALTGRTRGFGGAGLAGLSALAELLSSSLMAPVFLMYQTRSVIQVLMGRDGGWPPNNRGD

GRLNLRDAWAASNWISLTGIVGLIAAIWLTPALVPWLLPVGLPMIAAPLIIMWSSRPSRTALFSVPQERA

QPPIVTRHQAICDEWAAHPAQ*

>gkv_34|gene_NONE|conserved hypothetical protein

MADKPIMARATAVWLVDNTTLSFKQIADFTGMHELEIQGIADGDVATGVKGFDPIANNQLAAEEIAKGEA

SPLHKLKLKFNPAAVGEEKRRGPRYTPLSKRQDRPAGILWLVKFHPELADAQIAKLIGTTKPTIQAIRER

THWNIQNIDPIDPVALGLCRQSELDAQVQKANEKKAREGGTMTDDERRKLLSTEQSLSMPAEPRIPSAIA

GLETFSLSESKAEEALDADSFFNLPAGGDDDDEDDDR*

>gkv_35|gene_NONE|ribonuclease . domain protein

MSRPLLRVLMLLMTLALPVQAQQRGTAGDFDYYVMALSWSPTWCALEGDASASPQCDGTHGWVLHGLWPQ

YERGWPQDCHSAYTPPSRAQTAAMADIMGTSGLAWYQWNKHGACAGLPPRDYFALARSAYDSIAKPTAFE

RLQRPIELPASLVEDAFMQENQGLLADQITITCRAGRIQEVRICLTRDLDPRQCGADVIRDCTLDNALMS

PVR*

>gkv_36|gene_NONE|hypothetical protein

MRIAAQLLTGLALAAGLAAPAMAQSQRNTAGVDIGAVCPSGFTSTATAATVPVLAWTDHVPASPGFGFAT

RANVNVPAGSGDLVLTLTRPTLDGGTTQEQFRQQINGQGVSAFAYTFATPDETVAGTWRLVAEAGGAQIY

TAEVTVYTPGANDSLIASCS*

>gkv_37|gene_pckA|phosphoenolpyruvate carboxykinase (ATP)|

MDQGRVNPSMKLEQQGISGLGQVYYNLLEPDLVKAAVQRGEGELGQGGTVLVNTGKFTGRSPKDKHVVAT

PGVEPHIWWDNNRRMEPEAFDRLYADMLAHMKGRDFFVQDLFGGADPAYRLDVRMVTELAWHGLFIRHLL

RRPTSAEVDAFVPEFTIINVPSFRADPERHGCRSDTVIALNFEKKLILIGGTEYAGENKKSVFTLLNYIL

PEMGVMAMHCSANHAIDDPEDSAIFFGLSGTGKTTLSSDPARVLVGDDEHGWSDNGIFNFEGGCYAKTIN

LSAVAEPEIYATTKNFATVIENMVYDPETKELDFTDAKYTENMRCAYPLEQISNASSTGLAGAPKNVIML

TCDAYGVLPPIARLTPAQAMYHFLSGFTSKTPGTERGVVEPEPTFSTCFGAPFMPRRPEVYGKLLQEKIG

QYGATCWLVNTGWTGGSYGTGKRMPIKATRALLTAALDGSLNDVAFRKDPNFGFEVPVAVPGVDDALLDP

RQTWADAAAYDAKAAQLVAMFSKNFEKYLPFIDEDVKAAAIG*

>gkv_38|gene_NONE|hypothetical protein

LVIFYSQTAPFRRKALRQRKVDSRDAQAALDDSPRQLRRD*

>gkv_39|gene_NONE|HPr kinase

MPKPLSMILHASCVAIDGRAVVLTGASGSGKSALALQLIALGASLVADDRTIVTGSAQGLRATCPERLTG

LIEARGVGIMHAPHTPRARVVLAIAMDQAETERLPPPRSVTWCGHMLPLLHNPQASYFPAAIHTYLRMWR

DDRSPLLSN*

>gkv_40|gene_NONE|P-loop ATPase family protein

MNAPAEPITQQDPDPRIVIVTGPSGAGRTTAIRAFEDMGFEVIDNLPLSLLPRLFDGPPPARALALGIDA

RNRDFSVPALFEALRQLGDIPGVQGELLYLDCSEDVLIRRYSETRRRHPLSPAEAPQMGIRRECALLEPV

RAISGTLIDTSALTIHELRAEIEATYGAGVGLSVTVTSFSYKRGLPRAADMVFDCRFLRNPHWNPDLRPL

DGRDGAVADYVRADSRFGEFNTRTRDLLEFLLPAWQAEGKTHLSIAFGCTGGQHRSVMMAEEMRNALEAL

GWPVSISHRELDRQAANARSTNTGQRI*

>gkv_41|gene_NONE|PTS system fructose IIA component family protein

LIGIVIVAHGGLAREYLSAVEHVVGKQDGVVAIAIGPEDDRSVKQDEICAAADAVDTGDGVVMVTDMFGG

SPSNLSMRACNLSNRRMVYGANLPMLIKLAKCRQKPVEDAVTAALTAGRKYINSQMMAADGTLCHCEN*

>gkv_42|gene_NONE|phosphocarrier protein HPr (Histidine-containingprotein) (Protein H)

MLRTLSIVNIKGLHARASARLSETVERFDASALVRKDGLEASGDSIMGLLMLGAAKGTEIEVETSGPEAA

ALMDALEALVANRFGEPD*

>gkv_43|gene_NONE|electron transfer flavoprotein subunit alpha (Alpha-ETF) (Electrontransfer flavoprotein large subunit) (ETFLS)

MAVLVLAEVQSGALVADATARTITGARALGDVTVLVAGPAAAADAAAHIDGVSRVIWAQSPALDHQIAES

MADLIVPLASAYSHILAPATATGKNTLPRVAALLDVMILPDVIGILGADTFLRPVYAGNAVQKVQSRDAI

KVISLRTTAFAPAGAAASAAPVISVDAPAASALSSWVEDRVASGDRPDLSAAGIVVSGGRGFGSEEGFAL

LEALADKLGAAIGASRAAVDSGYAANDLQVGQTGKIVAPRLYIAVGISGAIQHLAGMKDSQVIVAINKDP

EAPIFRVADYGLVADLFTAVPELTAKI*

>gkv_44|gene_NONE|electron transfer flavoprotein subunit beta (Beta-ETF) (Electrontransfer flavoprotein small subunit) (ETFSS)

MKILVPVKRVVDHNVKVRVRPDGTGVELANLKMSINPFDEIAVEEALRLKEAGLAVEVVAVSVGTKASED

VLRKAMGMGADRAILVQLPEGSDAPEPLAVAKLLKSVVLSEGARLVIAGKQAIDDDMNATGQMLAALLGW

GQGTFASAVAVDGDVATITREVDGGLQTVALPLPAVVTADLRLNEPRFTSLPNMMKAKKKPIEAVDAATL

GVDLTPRLSVLETREPAARAAGEIVASVDELVAKLKAAGVI*

>gkv_45|gene_NONE|COQ9 family protein

VEAKRQAMMQAMGQHVLFDGWSEAAFLAAADDAGVAGAQARVLFPRGALDVAVALHKAGDARALADLAAD

PDARFRDRMAQAILLRLHHAGDRHVVRASSSLFALPQHMVEGAALIWGTADAIWTGLGDTSRDFNWYTKR

ASLAVVYSASLLFWLGNEDEAEVAAFVDRRIANVMALQAPPLKTLASTLLAPLRAPTARDDLPGRWG*

>gkv_46|gene_rpsU|ribosomal protein S21

MQVSVRDNNVDQALRALKKKLQREGVFREMKLKQHFEKPSVKKAREQAEAIRRQRKLARKKLQREGLI*

>gkv_47|gene_NONE|bkd operon transcriptional regulator

MQLDATDKRILDVLQKEGRITNAELADRIGLSPSACHRRMQRLEAEGFIRGFVALLDPRKVQRQTTVFVE

ITLSGQADEVLDSFERAVARVPQVLECHLMTGAADYLLKVVAEDTEDFARIHRRFLAKLPGVAQIQSSFA

LRTVVQTTALPL*

>gkv_48|gene_mscL|large conductance mechanosensitive channel protein

MFNEFRAFIARGNVIDLAVGIIIGAAFTAIVNSLVADLINPIIGVVTGGIDFSNLFINLGAGEYTSLAAA

KAAGAPVFAYGSFITAIINFLIIAWVVFLLVKAVNKMTQADKKKAEAAAAAAPKGPTQEELLAQIRDLLA

AKN*

>gkv_49|gene_NONE|glutathione S-transferase, C-terminal domain protein

MADLSAFAINDRWPAQNPDVIQLYSFPTPNGVKVSIALEEMGLDYEAHRITLADADVKSPAFLSLNPNNK

IPAIIDPNGPDGAPIGLFESGAILIYLADKTGQFIGTGAQRFKTIQWLMFQMGGVGPMFGQMGFFSKFAG

AAIEDPRPRERYLNEAARLLQVLDKELADKDWITGEYSIADMAIAPWINSLEFYGTKPLIGWDECKNVQA

YVARFLARPAVQRGLNIPPRG*

>gkv_50|gene_pheT|phenylalanyl-tRNA synthetase, beta subunit|

MKFTLSWLKDHLDTTASVEEITEALTDLGLEVEGVEDKTARLRGFKIGKVLNAVQHPDADKLRVCTVLTA

DGEQQIVCGAPNARTGITVVVASPGTYVPGIDVTIQVGKIRGVESHGMMCSERELELSDEHDGIIELPSG

EVGQEFVDWLAENQPAKVDPVIEIAITPNRPDALAVRGVARDLAARGLGTLRARDVQAIAPSFATDATIA

IAADTLDDAPVFAGQVLRGVKNGPSPVWLQDRLRAIGLRPISFLVDVTNFFSYDLCRPLHVFDLDKVKGT

VQVRRARAGEELLALDGKTYALAEGQVIVADDEGPISVGGIMGGERTGVSDETVNVLVESAWWQPIQIAM

TGRALKINSDARYRFERGVDPAFTLPGLDQAVRMIADIAGGEASQVIVAGAVPDVARAYRLDTDRVQSLV

GMEIAPETQRATLQALGFVLDGDMASVPSWRPDVLGEADLVEEVARIASLTKLVGVPMPRMQAGVPKPIL

TPLQRRLGTARRTVASLGYNECVTYSFIDQKSASLFGGGDDASMLENPISADLSHLRPDLLPGLLRAAAR

NQARGFADLALFEAGHVFSGGEPGDQVLHVTGLLVGRTAPRDVHKAARAVDLFDAKADIEAVLAAVGAPA

RVQIMRNGADWWHPGRHGTICLGPKKVIGTFGELHPRLLKEMDIQGPAVAFTIFLEEVPLPKSTNAARPA

LVLNDLQPISRDFAFVVPNAVEALTMINAALGADKAMIVDARVFDEFAGGNLPEGHKSLALSVRIQPQGA

TLKEAEIEALSAKVVEKVAKATGAVLRG*

>gkv_51|gene_pheS|phenylalanyl-tRNA synthetase, alpha subunit|

MDDLKAKYLGLIADAGDESAIEDLRVQALGKKGEIALLMRGLGAMSPEERQSVGPALNALKDEINSAIAA

RKASLGDAALAERLRSEWLDVTLPARDRRSGTIHPISQVSDEVTAIFADMGFAVAEGPQVESDWYNFDAL

NIPGHHPARAEMDTFYMHRLAGDNRAPSVLRTHTSPVQIRSMEAHGAPLRIICPGRVYRADYDQTHTPMF

HQVEGLAIDRNISMANLKWVLEEFVKSYFEVDSVDLRFRASHFPFTEPSAEVDIRCSWEGGTLKVGEGDD

WLEILGSGMIHPKVLKAGGIDPDQYQGFAFGMGIDRIAMLKYGIPDLRAFFDSDLRWLRHYGFASLDVPT

LQGGLSR*

>gkv_52|gene_rplT|ribosomal protein L20

MSRTKGGTVTHRRHKKVLDAAKGYYDHRRRTFKVAKQAVDKANQYATRDRRVRRRNFRALWIQRINAGVR

AIDQTLNYSKFINGLALAGIEVDRKVLADLAVHEPEAFAAIVEKAKAALA*

>gkv_53|gene_rpmI|ribosomal protein L35

MPKMKTKSAAKKRFKVTANGRVVAGQAGKRHGMIKRTNKFIRDARGTTILSAADEKIVKLYLPYA*

>gkv_54|gene_NONE|putative membrane protein

MQLDFAFVGAALLLGFAVLSLLSGLADGRLSRFGLLLFAAGFAIGGWVVINHPADYGLAQIPAVFIRVLA

ALTRIS*

>gkv_55|gene_pyk|pyruvate kinase|

MKRHRNVKIVATLGPSSSSYAMIRALVEAGADVFRLNMSHGTHDEQRDRYDIIRQVEADLGVPIAVLADL

QGPKLRVGTFADGPVDLSVGAPFRLDLDPTPGDIHRVQLPHHEIFAALRPGATLLVNDGKIRLRVDQCGA

DFADCTVTVGGAISNRKGVNVPDVVLPLAALSEKDLGDLEFACQLGADWIALSFVQRAADVEQARALVGD

RAAILAKIEKPAAVDDFDKILAVSDGIMVARGDLGVELPVAAVPPIQKRLITACRAAAKPVIVATQMLES

MIESPMPTRAEVSDVANAIYEGADAVMLSAESAAGKFPLEAVGTMDSVATEVESDPHYRELLDSTRKAVR

TTIAEGMVAAAREIADTTDVRAICCYSRSGKTANLVARERPRTPILMLTPLVPTARRMCLSWGTICRMTE

TVDEFRSAVVAAVRIAVAENLAEKTDHVVVVAGVPFNVVGSTNVLRVAPCDERLIYGTDMA*

>gkv_56|gene_NONE|N-formylglutamate amidohydrolase family protein

MTVSQDRITESFEIMGAARRSRWLVTCDHASNHVPDWVAGGDLGLPAADMTRHIAYDPGAAGVSRALSDL

LECPAVLSRFSRLVMDPNRGEDDPTLLMRLYDGSVIPANRNAGHAAREERLQRLYRPYHAAIESLAARQD

DTVIVAVHSFTPQLRGRPPRPWHIGILFDPADPDYSQALIDLLRAEGDLSVGANQPYSGHLPGDSIDRHA

LRYHRLNTLIEIRNDLITTPAQQAGWAARLAPLLQRALTLI*

>gkv_57|gene_NONE|conserved hypothetical protein

MDEQTRIQIEAAAFRRLQQHLMQDRPDVQNIDLMNLAGFCRNCLARWMDEAAAEMGVTIGKEPAREMFYG

MPYETWRAQNQTDASPAQLAAFEKAHQG*

>gkv_58|gene_NONE|putative membrane protein

MSAPAQLSDFVRDALAAGIGREVIRNRLQDAGWSAAEAENALAAWADPEGRIPPMPRPQPSYSAREALFY

ALKFLALIVVVWNVQALGSDLIERLAPRDYVPYVSRWSIANLIVFTPVFIGLHLYTLRLTRQDAAKRRSP

VRIWLGNIGQFIAVLTLLGIATTVIGTWLSNALDMQLALNLALLSVISVLVALFFRAELATK*

>gkv_59|gene_NONE|aminotransferase class IV family protein

MTRTVYVNGEYLPETEAKVSIFDRGFVMGDAVYEVTAVVGGKLLDFAGHEARLARSLNEIEIENPFAEGE

LLAIHRELLAKNELVDGGIYLQVTRGNPGDRDFVFPDPAVVKPTVVLYTQLKPGVLQSPTGINGIRVISV

PEMRWHRRDIKTVQLLYPSMAKMMAKKAGVDDAWFVEDGFVTEGSSNNTYIVKDGAIITRPLSQDLLHGI

TRAALLRYANEAQIKIIERPFTIAEAQAADEAFLTSASAFVTPVIEVDGAAIGAGTPGPIAKRLREIYID

EALKAGI*

>gkv_60|gene_NONE|uracil-DNA glycosylase

MDTAPDFYATREMLAWLVEMGADEAIADQPVDRYALPGESPKIAAPAPIAAPEKPKAARLGPLPEVDAPA

EAAALAASAQNLEQLASVMEGFDLCALKQGARSFVFSDGDPAARVMIVGEAPGREEDRAGRPFVGRAGQL

LDRMFGAIGLSRSAPMADQAVYIANVLPWRPPENRTPSDTEIAMMLPFLQRHIELADPEYLVLMGNTPCK

ALLGQAGILRLRGNWAEVTGRPALPMTHPAYLLREPGAKREAWADLLSLKARL*

>gkv_62|gene_moaB|molybdenum cofactor biosynthesis protein B

MTERAFIPLNIAVLTVSDTRALADDRSGDVLVQRLGDAGHTLAARAILPDERAVIADQLRAWCADPAIDV

VISTGGTGLTGRDVTVEAHRDVYEKEIDAFATVFAMISFQKIGTSAVQSRATGGVAQGTYLFALPGSPSA

CKDAWDGILAAQLDYRHKPCNFVEIMPRLDEHLRRKG*

>gkv_61|gene_NONE|FAD dependent oxidoreductase family protein

MASCDVTVMGAGIFGLSCAWALVQRGAKVRVIDPFGPGAGASGGVVGALAPHVPDQWNPKKQFQLQALLM

ADRYWAAVTEAGGAPSSYARTGRIQPLADQAAVTLARQRGLNAVDLWQGQANWQVVPLADNRWGIGSATG

LGVFDSLSARISPHRAVAALVATLRARGVAVVPDAPLQGQVIWATGYAGIKDLTAALGRSAGGGVKGQAA

VLACDLRDQPQLFIETLHAIPHADGTVAIGSTSENQWDDDSKVDAQVEALIARARDLVPVLRDAPVVARW

AGVRPKAKSRAPLLAAYPDRPGHFIANGGFKIGFGVAPMVGEVMADLVLEGRDTIPAGFGF*

>gkv_63|gene_NONE|UPF0209 protein yfcK. domain protein

MDNQHATITWRDGTIPVSDRFDDPYFSLDNGLEETRYVFLRGNDLPARFTGDFHIAELGFGTGLNFLVTL

QEWRASGATGRLTFTTFEAFPMTPTDRAQALAAFPAVADEAAELATALSTGNSVTLAGNVQLNVIIGDAR

ETLPAWQDRADAWYLDGFSPAKNPEMWDAALLAQVARHMTPAGTLATYAAAGFVRANLEAAGLMVTRIKG

YGRKRHMTTARFPL*

>gkv_64|gene_NONE|conserved hypothetical protein

MSTQNTRLGIMLMVMTTMVFSIQDGLSRHLAGEYNVYLVLMIRYVFFAIFALAIVQVSTGSLKKGLRTAY

PILQPLRGVLLIVETALLVYAFTVLGLIESHAVFTSYPLIVAALSGPILGEKVGWRRWTAIGIGFVGVLI

ILQPGGHVFSLTAIIPLAAATIFALYSLLTRYVSRKDSASTSMAWSALVGAICATAVGVFYWEPMAPSDW

VFMLGLCVFGMLSHWMLIKCYEVAEASAVQPFAFLQVGFVSLIGLFFFGETLEQRVIIGGSIILAAGLFT

LLRERAKAKA*

>gkv_65|gene_NONE|hypothetical protein

MATSTCNACAFYEDHVANSASKLQDAGLCRANPPISQPTAETRGFWPVVSASDWCGKFSVGFAAE*

>gkv_66|gene_NONE|heavy-metal-associated domain protein

MKFRIENMTCGGCARAVTAAIHDIDPDAKVDAAPDDRLIVVSSRAGADQISAALADAGFPATPAA*

>gkv_67|gene_cueR|cu(I)-responsive transcriptional regulator

MNIGDAAKATGLSAKMIRYYEQIGLIAPAARSLSGYRHYAEGDIAALTFISRCRRMGFGVEQIRDLLGLW

RDPHRASADVKAVATGHAQTLAAQARELQEMSQALQRLAASCHGDDGPDCAILDQFATLDPAPKPVRRRA

AATRFGPARAQGQLRSKPSVR*

>gkv_68|gene_NONE|bacterial regulatory protein, arsR family protein

MGIDIKIDPETMRCVADEASDLLKSLGNRHRLLILCHLGEGERSVGQLAEFLGIRDSTVSQHLALLRRDR

IIQGRRDGQTIWYRVNSPAAQQVMKVLYDNFSAAQA*

>gkv_69|gene_NONE|major Facilitator Superfamily protein

LAPATELAKAMAHTTQDNLFRHNLIVLTVAQALGAASPPIIVSLGGLIGQQLSDNPALTTLPVSLFNLGL

ALGTLPSAFIMRRYGRQSAYFIGALLAILGGLVAFTGILNHSFVIFCIGTMLAGYYAANVQSYRFAATDA

ASDAQRAKAISWVMVGGLFAAIIGPQTVIWTRNSITDVPFAGSFLAQAGLALLAILVLTRLRSTPPMQVT

ASDAPMRSVGQLLRSPRFALALGSGLVTYGLMVFVMTAAPVAMVGHGHGVDHAALGIQWHVLAMFLPSLV

TGRLMARFGTERITAAGLVIIGLSAIAALGGFGLMNFYLSLILLGVGWNFGFIGATAMLTASHTQAERAR

AQGLNDFVIFGTVALASFFSGALLQASGWEVINWMIFPAIALVLAPLVWSIGRKA*

>gkv_70|gene_NONE|conserved hypothetical protein

MSQTESYDGPAIRQIDETFSAVGQISPADVAGIAALGFKSLVCNRPDQEEPGQPSYAEIADEAAKHGLSV

TYIPVTGGVGPLPAQITAMREFLETAPTPVLGYCRSGTRVGVIYDATK*

>gkv_71|gene_cydD|ABC transporter, CydDC cysteine exporter (CydDC-E) family, permease/ATP-binding protein CydD

MRHSRSVHVQRSRNETLPQAGGADLLPSLSSLLWLPQALLIAMAVGAMASGHFDALPYQAMGVLALGALR

AWLDALGARLSFQAARAALTDLRAAALARLLASSPLDRDRQESGAAASIIAEQAEAVVPYLARFRPIRCK

ATILPLVIVAVVFPLSWAAALALLISLPVIPLFMALIGWRAEATSRAQMVELGQMNAFLLERLRGLATIR

GLGATARTTAAIHTHAETLREKTMAVLRIAFMTSAVLELFSALGVAMVAVYVGFHLLGDLNFGAWGGQLS

LTQGMFILLLAPHFFEPMRELSAVWHDRANGEAALAALQIQIAGAGTQLPSAPPAPASPTAPAVHLDQVS

FRHSGADKPVLQALSLKIAAGEHVAIMAPSGGGKSTLLALIAGLVPPDQGAIYIAGQTPAQMRQHMAWIG

QDPHIFAGTLAQNISLGRDLPPGAAAQAMADAQMQRVAALHGSGPIGEGGYGLSGGEVLRLSLARAMARP

ADHPAMLLLADEPTAHLDPTTAAEVTHSLLRAAKGKTLITATHDPVLAAKMDRIIQLDPIGMEDAA*

>gkv_72|gene_NONE|ABC transporter, CydDC cysteine exporter (CydDC-E) family, permease/ATP-binding protein CydC

MKSGLLRDLRPVLRLFWQHQRRAMLQGAALAALTALAGAALLGLSGWFLTAAALAGLSFAAKAAFDVFMP

SATIRLLALGRTAARYGERLVTHDATLRVLASLRAQLFDSWSRPGAAGRLLMRPARLLFRLTHDIDALDS

LYLRILVPAMAALSVAIGAGLLLGWMNLGLGLGVIAVLLVAGITLPLIAAQRAARATLRRAQMLETCRAQ

TVDLVQGQKDLLMVGQLQAQADAILATDRRLAKTDTALNRIELRTGLGFGLVGAAVLSGVLLVSGLLVQQ

GLLSAPGAALSVLLAFAAIEPFAALRRGAMELPRMRLAARRLNAQLAPPPKTVPLAAPAGALAVHLDGVS

VSGKSDQRLADLALDIAQGETVALIGHSGAGKSSLLALLMQEITPDHGIVQALPAALMTQSNQIFAQSLR

DNLRLAAPEADDAVLLAALARAGLENALPDGLDTRLGEGGLGLSGGQARRLALARMILRDAPLWLLDEPT

EGLDSAVARDVITQLAALTAQRTIVIATHIRREAEIADRLIVMQRGRITARHDRASPGFNAALALLRPD*

>gkv_73|gene_NONE|cytochrome d ubiquinol oxidase subunit 1 (Cytochrome dubiquinol oxidase subunit I)

MEFDIVTLSRFQFALTALYHFLFVPLTLGLSILLAVMETVYVMTGRVIWRQMTKFWGTLFGINFVLGVAT

GIVMEFQFGMNWSYYSNYVGDIFGAPLAIEGLMAFFMEATFVGLFFFGWDKMSKVGHLMATYAVAIGSNF

SALWILIANGWMQHPVGAEFNPDTMRMEIVSFFEVLMNHVAQARFVHTVSAGYVTASVFVLGVSAFYLLR

GRFVELAKRSMTVAASFGLLASVAVVVLGDESGYLVSENQKMKMAAIEGMWETQPAPASFTLVGIPDMDE

RTTHFGVHIPWVMGLIGTRSLTQEIPGIAELLVENEERIRSGLIAYDALQDYRATPTGQSVDPEVAARFD

EYKADMGYALLLMKYVDDPRDATEDQIIAAAWDTVPYVPVLFFAFRGMVGIGFLNVLLMAGFFYLSARRQ

LDTRRWPLKVAVAAMALPWIAAELGWIVAEFGRQPWAIDGVLPTVAAVSHLTVPTVLFTIAGFTAMYSVL

LVIEVRMMLAAIQKGPAPDDAPERPLFAKSGPVVEKRA*

>gkv_74|gene_cydB|cytochrome d ubiquinol oxidase, subunit II|

MILHQLIDYDTLRLIWWVLLGVLLIGFALMDGFDLGVAILLPFAGQTDIERRVIINTIGPVWEGNQVWLV

LGGGAIFAAWPILYAVSFSGFYLAMFAILMALILRPVGFKYRSKRESVRWRRNWDWALFIGGFVPALIFG

VAVGNVLQGVPFRLGTDMRIFYDGSFFGLLNPFALLCGLLSVTMLTMHGANWIVLKTEGVVAARARRFGR

IATLASLALYALGGIALWLFIDGYQVTSTFAPAGPSNPLAKSVEAAGGLWFSNYAAHPWMMIAPVLGFLG

PVLVLVALQFKRELLAMAGSGLAIIGIIASVGLSMFPFILPSSAQPGASLTVWDSSSSHVTLFIMLVSTA

IFLPLICAYTAWAYKLLWGKVRDDDINGSGHAY*

>gkv_75|gene_NONE|cyd operon protein YbgT

MWYFAWILGLPLAVTFAVLNAMWFELVDDAKAKKPEADAPDL*

>gkv_76|gene_NONE|hemolysin-type calcium-binding domain protein

MVTVIGNGGTLVIDQSNVNDVALLDLQLLGSATIIVDGVDFEADSLLLSAGVLSNITWEAKNGANLTVDA

GLIGLNALVDLNFNIYDDSSISYIGATVSLAGVLQGPIDVNYYGAEVGTFLYDPAVLGVVSATTFNVGAM

GPFDQFIVSGVNMTQGAYAGGVLPLTGFSGLLPPILGETIRVNVAMTAAEYGVIQNPPAGSGIGLVVSGG

NSIYTDPCFAQGSLIRTERGDLPVEQLRAGDLVLTADAGYQPIKWAGQSYLSATDLDQRPNMTPVRIAQG

ALGANLPMQDLIVSPQHRMLVNGTAVHGVTGHDEAFVAAKHLVGLDGVVVAKDMTEVTYYHILLDAHAVI

MSNGAPSESFYLGDFTRRTLNPRLMEEIRAIEPKLQDADFLPEMARPALTGAEARAVIADAAGAELLVAG

RELV*

>gkv_77|gene_NONE|ABC-type multidrug efflux pump

MFRWFENRVDPYPLQKPTQPPRGFWAFIIHYSRGLLPWIILLGLTSAIIAVVEVALVGYLGALIDRMAGA

DPAGFWQTEGRMVLLAGLVLVVIAPLVQAVSSLVMNQTLLGNYPQRIRWQAHRWLLGQSLSYFQDEFAGR

VATKVMQTALAVREVAMKIVDVLVYVIVYFSGALILAATSNLWLALPFAIWGIAYGLLLTWVVPRIGRVS

EAQANARAAMTGRIVDAYTNITTVKLFSHSAREEAFARQSMDTFLQTVHAQMRLATIQNVSLAVLNALLT

ASVAGLGIWLWMRGTVPVGALAIAVPLALRMGNMSHWIMWEFAALFENIGTVRDGISALAVERKVLDAPD

AKDLSVPKGEVTFRDISFDYGAAGNVIQHLNLTIKPGERIGLVGRSGAGKSTLVNLLLRFHDLKQGQILI

DGQDIAQVTQESLRANIGVVTQDTSLLHRTIRENIAYGRPDASIEEILAAARLAEAEEFIAKLTDNQGRI

GLDAQVGERGVKLSGGQRQRISIARVALKNAPVLVLDEATSALDSEVEAAIQEQLTHLMEGKTVIAIAHR

LSTIAAMDRLVIMDKGRIVEQGTHAELLASGGIYARLWARQSGGFIAAEDQPDTPEAAASGH*

>gkv_78|gene_NONE|efflux transporter, RND family, MFP subunit

MKPIAPLTRLIAALTAVTLFATAATAQPMGGRPQAGPTEVGVVTAQRETVPLTEIIPGRAVAFQENAVRP

RVNGMITEIIYTPGQTVTPGTPLFRIESDVYEAAVLSAEASLTQATTTRDNATVNVQRLEQLSSNNTGTR

TDLDTARATLATAEAALVTAQNNLDDARRELGWTTVTSPIQGIAGLPAVTIGDIVTANQTTGLVTVTRID

PIYVDLTEPAARLLSVRSRIEQGLLSRTDRVDVTLTLDDGQSSSYRGSLVAPGVSVSQTTASQTIRFQFD

NPGGRILPGMFVRGDITVGTTDAILVPQRATSRANDGSLTVWVVGEGNKSQQLTLTSLGVTRNAWIVTSG

IEEGAQIMVDGLNNMAAGRDVIPVPVTINELGLIEDMIPASAQAGN*

>gkv_79|gene_NONE|acriflavine resistance protein B

MAQFFINRPVFAWVLSIITMLFGVWGLQSLPIAQYPEVAPTTVRISASYSGAPAEAVENAVTTPIENGLS

DVEGLLYMVSQSSQGRATVTLTFDDSMDPDMAQIQVQNRMQSITNQLPDSVQDNGVSVSRSTDSILMVAA

LVSEDGSYTTLQLGDMMEELIEPTVTRVEGVGGIQAFGTSYAMRIWMDPFNLVQYQLTPSDITSAISSRN

STVTVGSLGSQPVVPGQQFSVSMTAQSQLTSPEEFERILLRTNEDGSHVYLGDVARVEIGQQSYGQDSRF

NGKPAAGFGVNLSSGANAVDTAHNVTTALTNLQNALPDGVEIAYAYDTSPFVEQSIEKVYHTLAEAVLLV

FLVILIFLQSWRATLIPTLAVPVVVLGTFGVLAVFDMSINTLTMFALVLAIGLLVDDAIVVVENVERVME

EEGLDARAATRKSMREITPALVGIVTVLSAVFLPMAFMPGSTGVIYRQFSVTIISAMVLSLFVALILTPA

LCATMLKPQHGPRKFPPARWFNNALAGFTNRYASTNRWILKVPVQSIIVLAAITGALWYLFENLPSSFIP

QEDQGVLMAMINLDDGATTAQTQTVLLEVEDYLLTEETETVEAVFANLGFSFGGSGQGSAMLFIKLRPFD

ERSDLTAAALVQRATARFSGHRAGNIFFMQPPAMPALGNSSGFSMYLVDQGANGQDALLTAAAAVNAAAT

ASDELNNIRGGTTRTQVALNLDIDQLKATALGLTVSDVNNMLSTIFTGRSVNDFAFNGTLRPVMVQAEPE

YRMQPDDVMHWHARNSSGEMVPFSAFMTQKWEVVPTSLARFGGTRAVSMSGSPAADVSSGAAMDRMEQIA

AEQPGGYSVAWTGLSYQERQSGSQAPLLFALSALVVFLCLAALYESWSVPFAVLLAAPVGALGALGAALL

FDQSNDVYFKVGLLATIGLASRNAILIVEFAKERFDRGMDLKDAAIEAARLRLRPILMTSIAFMLGVLPL

ATAKGAGAAAQHAIGIGVLGGMIASTIIGIFLIPAFYVVVTRMFGSRRNTLKSEE*

>gkv_80|gene_NONE|N-acylglucosamine 2-epimerase (GlcNAc 2-epimerase) family protein

VTAFDFTHWLSARALPLWRARGFCPQGFGAIEHLDADGQPALDQPRRNRIHTRQAFVFAGAARDLHSDDL

PRAQRLLGFARSLIGPGGWLPTTSDAAGQPAGDAHRLYDLAFYILANAELPAPVIPWDFLLDALARLKAD

RGWWDDRYHSLPRTQNAHMHLFEAAHAAHAATGDPAWAAVIDECRALFADVFFQSDGTIFEFFDADWQPL

QTGQQVEPGHGMEWVYLAYSDPVLRDQVDLDLMFNTAAAAMTPDGFLPDSTLPPSATCRLWPQTELLRAA

LVQQYRGRALPAPLQPTAILDRFAARYLTAEGGWIDACDRAGVIVADHMPSSSFYHIFSAWRAFSALSVD

AARLGSTDRQS*

>gkv_81|gene_NONE|hypothetical protein

VEQSFILQHFTLSPGLYLPGQQIVLRDPWGDLINAAKWRQVGAGPLSVRISGEGQFLLQAYALIGAEAVL

LSSLRVEGGQAVVLDALRDHVVRLSIIALSCVHLRGAVLLGAQPQRRARLAICIPAFGAGRGLPEKLAVL

RDYLRLMPLGAEARVLVIDQGLATPPVPHAGLRVVAQHNLGGAGGFARALREAQCDPWGFTHCLFTDDDA

HFTPEALHRLHAYLSLAQDPQLAVAAAMVTHQQPDQLWESGAIFDGFCRGAFRGTDLMDQVALARIERTS

AQADWQQQPGIYGGWWMFGFALAGVRYAPFPYFLRGDDIGFALANRLRIVTLNGVCAAQDDFAAKEGPLS

LFCDLRGHLIHPMVFPQIPGGRWRVGWRGALFVLRSLARFRYDEAAALLIAWQDVLAGPRAFATHPGAEA

QRHAIAQLPRPCAPLPRKQARRAWPRWYWMLSVNGQLLPFGSRGVVHIAPRRRRDMDAIWGRRAIVTDQC

DARDLRRFYPLFIRLLALWLRYMLGFKALARAYRATYGTVASQQAWDAHFQSSQPR*

>gkv_82|gene_NONE|conserved hypothetical protein

MIYARLVLAALVIALAGCAPRPDDGLLLPAAPENLSPHAQEVTVMTISNRNRTGPDGQFYGTAHSEMHRE

EFVIAVAPAREDTGSLLFDRDLSRDFAVVSRRALDAPDWHRALRAQHRTADQPIVLFVHGYNQTFQESLF

RLAQISAGMASEVQPILFSWPSQASLMGYVADRDSATVARDDLAQLLRDLHRNMPNRDIMIAGHSMGSWL

VMEVLRDLRRGGENRLLRRIQVGLAAPDIDVAVFRSQMAAVGQLSRPLTILVSQDDIALGMSGRLAEQRT

RLGALDASDPRTIALAEELGVRIIDVSAFPAADDFHHERFLALAAFHPQSAMQASIIDQLRFAGAYILDT

SGQVLRREITDDF*

>gkv_84|gene_NONE|conserved hypothetical protein

MPDTGPSSDVLIAVRRLVDGATVRDVAPLAQPAPLVLGADMIVAPLMLTPAQRVDDDMAAESLPELQPQP

EPEQAAVVSIGPFSGRAPQFGAGEEEGAVSPPSVSASLTISEDSLRELIAEVLRTELQGELGERITSNVR

KLVRREVLRSLNARDNE*

>gkv_85|gene_NONE|type I secretion outer membrane protein, TolC family protein

MKKTLRSVIAGFVASIGLAAPMAHAETLADAMAWAYESSGLLTQNRALLRAADEDVAQSVAALRPVLNWS

AQISSSFADATIFNNDPVTTANLAISASLTLYDGGRGRMGVDAAKESVLGTRAGLLAIEQQVLLRVVDAY

MNVQLYQQLVSLRENNVRVITEELRAARDQYEVGQITRTTVAQAEAALATARSTLVSEQGNLTRAREEYR

VATGRLPGALSAATPARITQSLDQARLIAQQGHPSVVQARHTAAAADINVERARTATQPTVTGTASLGRT

HYVTDALQGSAQAGSNTDQLTVGVGVSGVIYSGGSLGSQLRQVMANRDAARAQLLVAADNVSQSVANAYS

LLDVARVSRESFASLVTAYQLAYDGTREEADLGAATTIDVLDAEQDLLDARASQISAQYSEITAGYAVLS

AMGLLTAENLGLNVQIYDPEAYYNLVDDAPSVTSAQGRALDRVLQAIAHE*

>gkv_86|gene_NONE|protein-L-isoaspartate(D-aspartate) O-methyltransferase (PCMT) family protein

MQAVIQSSAESDFAALRVTMVDRQVRTSDVTSYPIIAAMLAIPREDFVPDALRDVAYVGADLPLAPARTM

PEARTLAKMIDGLAIAPDDRALVIGAGLGYGAALVARMAGQVIALEELPHLAAGAAAALARHKSDARIVQ

GKLVEGAPEAGPYDVILIEGGVRAIPPVIAAQLAEGGRIVALFVEGGLGLVRIGTKVAGQIHWRLAFNAL

LPVLPGFTTTDTFAL*

>gkv_87|gene_NONE|conserved hypothetical protein

MIRYTLKCPSGHQFDSWFQSASAFDSLQKAGHLSCATCGAAPVEKALMAPNVAKSGPTAPSNEDQLKALR

TEVEANSEYVGMSFATEARRMHAGDAPTRAIYGEAKLDEARALLEDGVPVMPLPFIPTRNTN*

>gkv_88|gene_NONE|asparate kinase, monofunctional class

MPILVMKFGGTSVANIDRIQRAARRVAAEVAKGYDVIVVVSAMSGETNKLVGYVNDTAPLYDAREYDAIV

SSGENVTAGLLALRLQEMDVPARSWQGWQVPLQTNSAHGAARIAAIPTENINRKFAEGMKVAVVAGFQGI

SPEGRITTLGRGGSDTTAVAFAAAFDAERCDIYTDVDGVYTTDPRICSNARKLDKITFEEMLELASLGAK

VLQTRSVELAMRFKVRLRVLSSFEEPSDTSGTLVCDEEEIMESNVVSGIAFSREEAKMTLVGVADKPGIA

AAIFGPLADAGVNVDMIVQNISDEGQTDMTFSCPVDQVLRAEKAINEARARGDYTFDELVCDSNVAKISV

VGIGMRSHAGVAATMFRALASESVNIKVITTSEIKISVLVDRKYMELAVQALHDAFGLEKKKA*

>gkv_89|gene_ptsP|phosphoenolpyruvate-protein phosphotransferase|

MAARDESESRKLLGRLRAALAESSAGQARLDGIVHLIAGSMQAEVCSIYLFRDTDTLELCATEGLMPSAV

HRTRMRLGEGLVGRVARNGGIINTPNAPSEPGFRYMPETGEERYSSFCGVPIQRLGETLGVLVVQTKTQR

ELTSDEVYALEVVAMVLAEMAELGAFAAPEAQGQSHRKALMIRGVSGQEGTVKGHVFIHEPRVVVTNAVA

EDPVAELSRLRDAVDNLRFQIDEMLDKARGMNTEQAQVLDTYRMLAASRSWLRRMEEDITNGLSAEAAVE

REQTSTRNRMAQVEDAYLRDRLHDLDDLSNRLLRILTGQGKDTGAEMPIDPILVAKNIGPGELLEYGKII

RGIVLEEGSVGSHAAIVARAWAIPLVIHAKGIVANALNGDPIIIDGDQGNVHLRPDDAISRAYTEKIAMQ

ARALERYASLRDLPATTTDGETIALHMNAGLMADLPSLQSSGAEGVGLFRTELQFIIRNKMPTRSELSAL

YARITDAAAGKRVVFRTLDIGSDKVLPYLKPQDEPNPAMGWRAIRVGLDKPGILRMQLQALLRAANGRPL

TIMFPFVAQPAEYWAARAALDKVIAREKILGHNLPAQLEVGVMLETPSLAFAPLAFFKSIDFLSVGGNDL

KQFFFAADRENERVRRRYDLFDPAYLSLIDMIVGKCRAAGTSVSFCGEDAGRPLEALALAAMGFRTLSMR

PASIGPIKHILRRVDLSAVRMLIDEHRMRGENPRAALKEWLQESDVL*

>gkv_90|gene_NONE|acetyltransferase (GNAT) family protein

MFTIAQETPDDWWEVESLYDLCFAPGREALSSYRLRDDVPPIAGLSLVARGTDGSIAGAIRHWPVRICTE

DGICNRSLLLGPVAVHPTHQGEGLGGLLIYQTLGGARALGWERVMLVGDAPYYNRFGFQKLEGVLMPPPT

NPDRVLGVALKPNAWAGVRGQVSRWSPDMALPQN*

>gkv_92|gene_NONE|flavin reductase-like, FMN-binding

MFYTPEAGHSLPHSPLTAIVAPRPIGWISSRGADGCDNLAPYSYFNLVASAPAQVMFSSTAQKADRGDTK

DSVGNIRETGVFCANIVSEALLGAMNLSAANWPRDIDEFEAAGLAKADCQTINCPRVADAPASLECVVSQ

IITLEGAHNFMVIGRITGVHIADTCLDANGAFDPRLFGQASRLGYADYAVVRDTFAMPRPQRIG*

>gkv_91|gene_NONE|cytochrome c family protein

MNRLALPAIATLALIPMGVLAQDFDPDLVERGRYVAVAADCAACHTNHEEGLEWAGGYVLDTPMGQMIST

NITPSVEFGIGGWTEAQFERAVRHGVAPGLHMIYPGMPYADYARISDEDISALYAFIMSEIEPIDQAPAL

QTSLAFPFNIRYVMAGWNLLFGHNQPFDAATTQPGGADRGEYLVTALAHCNTCHSPRNILMAEGGPFLSG

GNVGGWDVPNITSDPISGIGGWSDEEIVQYLRQGNAHGKAQAAGPMAEAVSYSLQYLNDDDLQAIAAFLR

TVPPVRTEGQDVPSFAFRDPDTLALMNPDGTVGAVPAVTPNPEGEQLFLSSCANCHGADARGTDDFYYPS

LVNNAATSGTSAANLVMVISSGLQRETEEDYFFMPAYAGQWTSQQIADVANYVLQSYGNPELVVDAAYVE

LLQAGGEPPLLMRAMPYLLALGGIGAVAVLLLIIYLIRNRRRRARFG*

>gkv_93|gene_NONE|hypothetical protein

VSLYRRKITAEIGAALAVLGLWLLVIISPLHQVSAFARDAGFGTPWTICNVPQEHGSASHDPMMMACPVH

AIGKNHLDLALPPVLAAQILHTTFAYANLTMPARLAWSRIHLEPGQPRAPPAVL*

>gkv_94|gene_NONE|hypothetical protein

VPLGAAKTCMFFKDGRMKSAYFAAFLAATAVPALAEQPVTLNFVAEIGGAAFDCSQSYDHLGATHAQMNV

TDFRLFVQDAALIRADGSQQPITLTANDWQHENVTLLDFENGAGHCAGTGNAPMNTVITGEVPEGDYIGV

SLTLGVPFALNHIDPTLASAPLNTTGMFWTWQNGFRFLRIDFAPAGGTPMAHEGHGGHSDHGNASGWYLH

LGSTQCAAASQTEAPSACVFPNRTTASFTSFDPATQVIVVDPAPVLRDADVTVNAPDTSPGCMSFPGDAD

CDSVIPKLGLAYGDHPAEAQQLFSIR*

>gkv_95|gene_NONE|di-haem cytochrome c peroxidase family protein

MRKQLCLGFGAALAILAAVATAGANGRYIWPMPAWMPPPPVPAGNPMSAEKVDLGRHLFYDARLSRDGTV

ACASCHEQARAFSDGRELAVGIGQTIGIRNAPALANAGYVPQLTWANPHLTTLEAQALVPLFGEDPVEMG

SNGREADIFAALAADPYYTDAFASAFPDRPVIDLFTVTRALAAFQRSLISLDSPYDRFKYGGDADAMTPA

ALRGERLFFDHRFECYHCHGGILFTDSQQTARNPAPMLGNHNNGLYNIGGTGAYPARATGLYEFTGLAAD

MGRFRTPSLRNVAVTAPYFHDGSAASLREVLDHYAAGGRTISDGPHAGIGAANPFKDGILVGFDASDAEI

NDLIAFLESLTDEGFLTNPAYSDPWPADHPARATRMMP*

>gkv_96|gene_NONE|DUF461

MKTLMIAAAALFAASLSTSVAAHEFVLQDIQIIHPAVPASPPGATAAPLYMALSNNTDTADRLLGIETPY

GPVRFRRPVTAADGTTRFEGMAWIDVPPGEIVVLSQGAMHGVFDIPEPLHEGGEIPVTMVFEARGRVEMH

LMIDPVGALDTETSVASGAQAPQAGDVLQVGAALRSALAPTQATIMPIVLHGNHAVAGWSNDTDAARALL

RRVDGNWQVEMWSGASLLLPATFVSMGMSQADAEILIAEVKAYESMIDPAIIARFDAFPGTVLIEGDAQ*

>gkv_97|gene_NONE|conserved hypothetical protein

MKHLLLTMALAIALPGLAMAVTPLSYSTAGGAPVQAGEVEVSARFARATLPNAPVGGAYMTITNKGAEDD

RLLGATTPLGPVTLHAMEMSGSAMTMRPLPDGVPLPAGQTVTLSPSGTHVMISPLTSALVQGETLDLTLE

FEKAGAVTMTFDILALNARTHPEAEPQ*

>gkv_98|gene_NONE|SCO1/SenC family protein

MTRKPKQTPVVSAEEAKRRASLRNIRIAIWGLAAIAGAGAVWLTTTQRTAIPAPMTMQAAYSDIGQGDYS

LQTTDGQPFTAASLVGQPSLVFFGFTHCPDVCPTTLGDITLWKDDLGAVGDDLQVVFITVDPERDTPELL

HDYVSWVPDSVAVTGTPEEIEKTVTAFRIYASKVPLEGDDYTMNHAAYVMLFDENGQFNQIFSYQEDIDR

VTAKMREFLS*

>gkv_99|gene_NONE|sensory box protein

MVANAPHDQQPALTVEEQDLHPQRASLSPFPSEMEFIRYALDSAAIVAMTDVRGTITFVNSKFCEISGYS

RDELIGANHRILNSGIHSTDFFRSMYRRIAGGEVWHGEICNRRKDGSHYWVDTTIVPHVNINGKVDSYTA

IRFDISSRHAAEELLRRIVSVDSLTGIPNRRSFQEYLESVLPPERDNPGQVHLALLDVDTFKEINDTFGH

DVGDTLLKLVSERLSALSSPDVFVARLGGDEFCLVMTNMDRTGVNDLVQTALAHLREPVPLGSVIRRYSA

SIGVASFPEHAGSLDELFKAADMALYHSKALGRDQAQFFIPRLREIAERKSELLHAVEVGLDRGQFHLYY

QPIVPISSPGALSFEGLLRWNHPDRNLITPAAFLTDMDDPGLQAAIGMFVVEQAFRDMRIMLDQNVPLRR

LAINITNADFRSDAFVDRFFELSRETGIPPSKFCIEVTEGVFLGRDFQHLAGRLSQLHAAGVEIALDDFG

TGFASLTHLRRMPIDRIKIDRSFISNITNSIEDLAIVRGVIDIAHSMGKVVTAEGVETRAQVELLHSLRC

DYYQGWYFSKATPIDGLRDAVRNMPPLIAW*

>gkv_101|gene_NONE|thioredoxin family protein

MDLTADLNTKDIIIDGTDAGFMDDVIAASQQVPVIVDFWATWCGPCKTLGPALEAAVKKAGGKVRLVKID

VDKQPQFAAQLRVQSIPTVYAFWQGQPVDGFQGAIPPSQVDEFVNKLSALAGDDGLGAALDAADEMMEQG

AFADATEIYNAVSQEEPENARAYAGLVSAALAQNDLDGAEAVLNGAPAVIAKSPELEAVRARLELARQAA

SAGPVDDLRAAVAADPANLQALFDLATALHASGDVQGAVDSLLDLFRKDREWNDGAAKAQLFTIFDALPP

KDPIALAGRRRLSSLIFA*

>gkv_102|gene_NONE|ATP-dependent protease La (LON) domain protein

MAEAAPDRLPAQLPERIALFPLFGALLLPRAHLPLHIFEPRYLAMVDEVLTSPHRLIGMIQPLAPNEGAR

LHRIGCVGRIVGFAERPDGRMDLTLAGVSRFRLTSELIVSTPWRQAEVSWDGFAHDRNRMAETDPYLDRA

ALFALLARFFAARGLPHDWQNLKSVPDELLINVLSVLCPLPAGDKQALLETPHLPERRETLITLLEFALQ

RGGDDAADDDEIMQ*

>gkv_103|gene_NONE|trm112p-like family protein

MTTPAPRLFDPRMLEALVCPQTQTTLTYDRERGELISRAAGLAFPIRDGIPVMLVDEARKLD*

>gkv_104|gene_xseB|exodeoxyribonuclease VII, small subunit|

MTQKPVADLSFEEALAELEQIVAQLERGDVPLEESISRYQRGAELRARCTAKLNEAQEKIARITIGENGQ

PAGLQPFEA*

>gkv_105|gene_NONE|polyprenyl synthetase family protein

MQSLSTRMAAAAADVQALIAAHLSPLAAASPVASAMIYACTGGKGLRGFLVLESARLHGIAYADALPVAA

AVEAVHAYSLVHDDMPAMDDDDLRRGRPTVHRQWDEATALLAGDALQSLGFELIATAALPDSARVTLLAG

FARAAGIHGMVGGQEADIAAETAARPLSLDQIIALQRGKTGALITWSASAGAVMAGADAAPLRAFGDAIG

LAFQITDDILDVEGSAATMGKAVQKDAAAGKATFVSLLGLQGAKDQALALLQTADAALIPYGATAATLRE

TAQFVITRQN*

>gkv_106|gene_dxs|1-deoxy-D-xylulose-5-phosphate synthase|

MTDIPRPSTPLLDRVSSPADLKRLSDADLVRLAGELRAETISAVSETGGHLGAGLGVVELTVALHAVFDA

PRDKIIWDVSHQSYPHKILTGRRDRIRTLRQKDGLSGFTKRSESPYDPFGAAHSSTSISAALGFAVARDL

GGATPEGAIGDTIAIIGDGSMSAGMAFEAMNNAGDLKKRMFVILNDNEMSIAPPVGALSSYLSRLYAGGP

FQDLKAAAKGAVNMLPEPLREGAKRAKEMLKHMTVGGTMFEELGFSYLGPIDGHDMEQLLAVLRTVHDRA

TGPVLIHAITQKGKGYAPAEAARDKGHGVGKFDVVTGEQAKVKSNAPSYTSVFANALIEQAEKDSRIVAI

TAAMPDGTGLDKFMVRFADRCFDVGIAEQHAVTFAAGLAAGGMKPFCALYSTFLQRGYDQVVHDVAIQRL

PVRFAIDRAGLVGADGATHAGAFDIAFMANLPGMVVMAAADEAELVRMVATAAQHNDGPIAFRFPRGEGV

GVEIPADAQPLEIGKGRITQAGARVAILSFGTRLSEAEAAAELLSARGITPTIADARFAKPLDRDMILRL

AREHEVLITIEEGAIGGFGSHVAQLLAEAGVFDHGLKFRSMVLPDIFIDQANPRDMYDVAGLNANDIANK

VLSALGVALLERRA*

>gkv_107|gene_NONE|major Facilitator Superfamily protein

LSATLVAGRKVPVGIIIFALAMGGFSIGTAEFAAMSLLPYYALNFGISEAEASHAISSYALGVVIGAPIL

AVLGAQMSKRILLVALMAFYAIANLAASVAPTYQLMIVARFFAGLPHGAYFGVAMLLAASLVPYEKRSFA

ISLVITGLTVATVVGVPAANLMGQTIGWRWGIAIVGALALLTAVLILSLAPQDKPNKAANPLSELSALKN

RKVLLALLSGAVGFGGYFATYTYAASTLVEVTQMPERMVPLVFALMGIGMTLGTLVIGKLADKSLIGTGW

TILGASFVLQLIYPSATGNIWSVVLVLFLIGAFSSFSTVMQTWLMNVAGDAQTLAAAMNHASFNMANALG

PLFAGMALAAGWGLPSTGYVAAIMYAVGMVIFAIMIWDLKRDQA*

>gkv_108|gene_NONE|RNA modification enzyme, MiaB family protein

MDQNAPIFATLGCRLNAYETEAMKDLAARAGVSGAVVVNTCAVTSEAVRKARQEIRKLRREHPDKKIIVT

GCAAQTEPETFTAMGEVDFVIGNTEKMKPETWAAMAPDLIGRTEPVQVDDIMSVKETAGHLIDGFGTRAR

AYVQVQNGCDHRCTFCIIPYGRGNSRSVPAGVVVDQIKRLVDRGYNEVVLTGVDLTSWGADLPATPRLGD

LVMRILKLVPDLPRLRISSIDSIEADEMLMQAIATEQRLMPHLHLSLQAGDNMILKRMKRRHMREDAIRF

CEEARVLRPDMTFGADIIAGFPTETEAMFENSLKLVQDCGLTWLHVFPYSPRKGTPAARMPQVNGNAIRE

RAARLRVAGDAQVAVHLAQQQGVQHRVLMENARMGRTAQFTEVIFDSDQPEGQIVNAEVMGIAGNQLTAR

AL*

>gkv_109|gene_dapF|diaminopimelate epimerase|

MQSNSDTGLPFMKMHGLGNDFVVMDERGLPPRVTPALVAALADRNRGVGFDQMAIITDGGNADLHLTFFN

ADGSPSAACGNATRCIARYEMDRTGKTSLTITTDRGVLLAREEGNGLTSVNMGHPMTDWDEIPLAEDVDT

LALPILGAPTATSMGNPHCTFFVDDVMAIDLAAIGPTIEHHPLFPERTNVQFAQVIGEDRIRMRVWERGT

GITLASGSSSCATAVAAYRRGLTRAKVEIVLDGGSLQVEWRDDGVWMTGPTMHAFNGVLTQQFLDSL*

>gkv_111|gene_NONE|acetyltransferase (GNAT) family protein

MISLRKMLEPEYQAYLDYFIPDYAAEIAANYGLTLVDAAAQAQREIAHSLPHGPQTAGHILFCIFDRTAK

GEDHVGYLWCKPDTEAQSVFIYDFGILPAFQGRGLGKAALARFEDDMAALGFKQIKLRVAGDNARAKHVY

ERGGFRVTGINMAKSI*

>gkv_112|gene_NONE|zinc-binding dehydrogenase family protein

MKAVFYTGDKSFELREIARPDPAAGEVEIAVAYNGICGTDLHAYHGAMDARIGHNRVLGHEMSGRVARLG

AGVTGLAVGQPVVIRPLKPCGDCPACNAGLSHICHKLKFLGLDTDGALQDYWSVPAYAVHPLPETVPLDH

AALAEPVAVAAHDVRRSRLAAGEFALVIGGGPIGLLIAMVARHAGARVLISEVNPLRIGIAQEMGFDVVN

PREANVIDTVNAATGQKGADVVFEVSGTVAGVELMTDVAASRGRICMVAIHTTRPQVDLFRFFWRELELI

GARVYEAEDFDTAIDLIATGVVDAGRMITDVQDLSQVGAAFAALDGNAKAMKSLIRISG*

>gkv_113|gene_NONE|bacterial extracellular solute-binding proteins, family 3 family protein

MKINRRIALLAMTAGMSLGLAMPAAADLAEIRERGVIRIAVAMGIPQYSYIDSNMQPAGSDVETARMLAE

DLGVALELVEITNAARVPTIQTDKADLVVSALGITDERRQAIDFSVPYATLALVVAAPADIDISDYADLT

GKRIALTRATTNDQDITANTTGAEILRFEDDATLITSVISGQVDIISSQSAVIGGINERRRGGPLEIKFI

QRETNLGIGLAKGNPELLAWVDEWVVTNFDNGRLREVFTTYQHRDLPDDLTSR*

>gkv_114|gene_NONE|glutamine transport ATP-binding protein glnQ

MSVAVKLDNVVKRYGALEVLKGVSFDIKAGEVVALIGASGSGKSTALRCINRLEQIQGGTIEVCGRAVND

DKVDLRALRQDVGMVFQQYNLFPHMTVGENVILALRRVKKISRADAVQISRKVLDQVGLLEKIDNYPEQL

SGGQQQRVAIARSLAMQPKVMLFDEVTSALDPRLTGEVLRVMEDLAKGGMTMVVVTHEMGFARRAADRVI

FMHHGNIHEEGDAAILNAPTTPELREFLEHDLN*

>gkv_115|gene_NONE|amino ABC transporter, permease protein, 3-TM region, His/Glu/Gln/Arg/opine family domain protein

MQTFGTGHFIYLLNGLWWTIILSALAMSLGSIAGFIVMLGRISRRKWLVRLSGTWVQIIQGTPLLIQMFI

IYFGLGVVGISVPALAAAAIATMIYASAYLGEIWRGCVQSIAKTQFEAAESMGLTRWQALRDVILPQAMR

IATPPTVGFTVQLIKNTSLASVVGFLELTRAAQVINNSLFEPFLVFGIAAALYFAVCYPLSLWSRNLERK

LNVGRR*

>gkv_116|gene_NONE|amino ABC transporter, permease protein, 3-TM region, His/Glu/Gln/Arg/opine family domain protein

MSYQFNFAALVPYWPDFISGAWVTLQLTVGAVIFGMVIGVLCAIARRSRFVALRSVAGVYVETVRNTPFI

VQIFFLFFGMSSVGVRMPIMVAAIFALVVNVGAYTAEIIRAGMDAIPKGQIEAAEALGLSQFQIYRDIIL

MPAIEKVYPALTSQFVLMMLTTSICSQISAEELTGVANNIQSNTFRSFETYIVIGLFYIGITLMMRFGFW

LIGLYAFPRRRVMGGV*

>gkv_117|gene_kduD|2-deoxy-D-gluconate 3-dehydrogenase|

MSANPLFDLTGKVAVVTGANTGIGQGIAIALAGAGADVVLVGRSPMDDTRSQLQGFGTRVHEIRADLSST

APLAGIVDEVVSKLGGVDILVNNAGIIRRADAVDYTEADWDAVIDTNLKSLFFLSQAAGRHMLAKGAGKI

INIASLLSFQGGIRIPAYTASKSGVAGLTRLLACEWAAKGVNVNAIAPGYFVTNNTEALRADADRNAAIL

GRIPAGRWGKPGDIGGAAVFLASQASDYVDGVVLPVDGGWLAR*

>gkv_118|gene_NONE|4-deoxy-L-threo-5-hexosulose-uronate ketol-isomerase (5-keto-4-deoxyuronate isomerase) (DKI isomerase)

MSEAIDIRHNVHPEMAKALDAEGLRRHFLIADLFRMGEVRMTYSHIDRLIVAGAMPADTALALPTPKAVG

QTRFFDNREGGVINIGGAGRVIVDGAAHDLHGETALYIGRGTAEVQFESTDPAHLAQFYMISTPAHKDLP

TRRVTADQANRLNLGEVATANKRTILQFLHPDVIETCQLTMGMTRLEAGSVWNTMPAHTHDRRSEVYLYF

GLPEGQRVFHLMGEGHETRHIVTGNGEAILSPSWSIHAGAGTSAYAFIWAMGGDNKNFTDMDHIKIEDLK

*

>gkv_119|gene_NONE|bacterial regulatory proteins, gntR family protein

MERKARTIGGISEVSRTRAQAPRTADLIYRDLHRDIVSMRLKPNSKLSEKELSEHFGVSRTPLREAIQRL

AEDGLVAVFPQAGTFVAPIPVRLLMESILIRRALEVVIAQTATEMAVAQDIRDIDANLAELEGAVNAGDL

GEFHRIDSEFHRLIGRISGLGTVSNTIEHVRAQIDRYRLMTLPQAGRLTRVITEHRAVRDAARAGEAMAF

HIGQMLDEVEALQHLDRDYFYDDRE*

>gkv_120|gene_NONE|bacterial regulatory helix-turn-helix protein, lysR family protein

VNIRQLEAFHAVMETGSATRAGERLGITQPAISKLMKSLADECGFVLFQRRGGQLVPTREAQLLGLEVAR

LFSGSRRVAEFIQAIRTNQVGEVSLAAPPALATRYLPQILASEIRDLSDLHLQIMSRSSPQIIDLVAAGQ

LDIGLSSMAVDHPDIEVEHVRSFALVCLLPFGHPLGAKPALDIEDLREQPFISLPTGDCTFSNTSRAFKV

NGVSVSRRIEAPHSETAALMVANGIGLTIVPPFAGIEFDAQRVLRRAIRPVEHLDIWMLKRRNRPISMAA

DMIRAQILRTLRAIDEEPLAQPSVSLASVSLAS*

>gkv_121|gene_NONE|periplasmic dipeptide transport protein (Dipeptide-bindingprotein) (DBP)

MKTSFAAAAALLALSSPAFAGTLTVCLEGAPEIFNPQLTSSGTTSTVLGQIYDNLVAVERGGANIQPALA

ESWDVSDDGLTYTFHLRQGVQWQSNDAFTPTRDFNADDVVFTFARMMDEAHPYHSVSGGSYITFNTKLAD

ALAGVEKIDDYTVAFTLTAPLAPFIGIMAHGSIAMTSAEYADVLQAAGTPDAFDREPIGTGPFQLQAYQT

DAIVRLIPFHDTWGEAAGIAENTPMVDAIIMAISADASVRVQRALAGECAISYYPNLADAPLIDASETVE

LVPAQVASSGFITFNQTIDKFQDIRVRQALAHAINMEPLVETVFNGMGHLTGAVVPPAFWGSATDLAPYA

YDPELAKQLLADAGFADGFTTQIWAVPVSRPYMPNGRRAAEMIQADWAAIGVNAEIVTYEWAEYIQRSRA

FEAEVGMFGGIYDFPDPSQIPNNYFTCNAEGTPSPSNIGHWCNAEFNAVMQAAGEITDQSEREALYLQAQ

HILYDDVAAVMFGGADQLMAVSTSVNGFTPAIFGSSRMSGVTVE*

>gkv_122|gene_NONE|FAD dependent oxidoreductase family protein

MRAIVIGAGMFGASTALQLARRGVQVKIFDFAHNGKATMAGAGIVCPWATQIEEPAWYEMYAAGARFYDT

LIADLNGAGETDLGYRKVGALVTSQNADDFKAAGARIARRAATAPEAGDVRLLSPKDAQALFPVLKDGLE

AWFIPGGARVDARLLSAAMVRQAIALGAEFSNDYVTLVQGPEGTAVRDGSGTLHTADEVIVAGGAWASQI

LAPLGVDHPVKPQKGQIVHLRLAGVQTAQWPVLLPMTSHYMLAFDDSRIVVGATREVDSGFDYRVTATGQ

ASVLEAGMAIAPGLAHAEIIETRIGFRPAGPTIKPIFGRVPGAPGLSLANGLGAGGISIGPFAGKLLADV

LTGKQTEVPVAPYAPQN*

>gkv_123|gene_NONE|endoribonuclease L-PSP family protein

MIKHFRKSAHLHGAVQHNDTLYISGHAAHDLSQDMAGQTQEICDKLDKLLADCNSDKTLLLQARIYVTDM

TQKEAMNKVWLAWLDGIDLPTRATIGVNDLGDPRRLIEIATVAAVREA*

>gkv_124|gene_NONE|dipeptide transport system permease protein dppB

MLRFLFRKLIVIVPVLIGITIVSFGFVRILPGDPVMLMAGERGLTPERHAQLMAQFGYDRPILVQYFSYL

GDVLTGNFGTSLVTKKPVLADFAALFPATVELAICAVILAALIGIPLGVLAAVKRGSWFDQIAMSIALTG

YSMPIFWWGLLLIILFSSTLGWTPVSGRIGLMYYFPNPTGFMLWDAATSGQTGAFTSAVRHLILPSIVLA

TIPMAVLARQSRSAMLEILGEDYVRTARAKGLSPLRIVGLHALRNALIPIVTTLGLQIGALLTGAILTET

IFSWPGIGKWMVDSISKRDYPVVQGGLLLIAAIVMAVNLLVDVLYAVINPRIRHK*

>gkv_125|gene_NONE|dipeptide transport system permease protein dppC

MTDTISQPIPKPVGPIRAFWYGFSANKGAVLGLIVFSIVVLTALLAPLVAPHSPIQQYRDAFLVPPAWLE

GGRAEFLLGTDAVGRDILSRLIWGARYSLYVGLLVITIALICGVSLGLLAGYVGGAVDTVIMRVMDVILA

FPSLLLALVLVAILGPGLTNAMIAIALVLQPHFARLTRAAVLSEKGKDYVTALRVSGASPLRLMFVTILP

NCMSPVVVQATLSFSTAILDAAALGFLGMGAQPPTPEWGTMLADARELILRAWWVATFPGLAILVTVLAI

NLVGDGLRDTLDPKLKKG*

>gkv_126|gene_NONE|ABC transporter family protein

MALLEIKNLSVTFPTAAGVLRAVDGIDLRVDQGEILAIVGESGSGKSVSMLAVMGLLPPAAVIRADQILF

NGQDMLRLSARARRKIIGKDISMIFQDPLSSLNPSFTVGFQICEVLRLHLGLSRRDAAARAIALLDAVGI

PDPAARMNSFPHQMSGGQCQRVMIAMAIACKPRLLIADEPTTALDVTIQKQILDLLADLQRETGMGLIMI

THDMGVVAETADRVIVQYKGRKVEEQPVLDLFSSPQQAYTRALLSALPENATGLRLPTVDTLMKGGA*

>gkv_127|gene_NONE|dipeptide transport ATP-binding protein dppF

MGEIVLKAEGLIRDYTSGGLIGGRKVNRALKGVSLQLERGRTLAVVGESGCGKSTLARILTMIDAQTEGD

LTICGEKVDLSQHKPGRDLRRKVQIVFQNPYSSLNPRQKILSALTEQLYLNTDDTPTQRRDKALAMLGRV

GLGPEVAGRYPHMLSGGMRQRVVIARALMVQPEIVVLDEPVSALDLSVQAQVLNLLRDLQDEMGLSYIFI

SHDLSVVRYLADRLIVMKAGEIVEEGAATDIFAAPQHAYTRKLFAATPSASIEAIRTRITARQTAGLRP*

>gkv_128|gene_NONE|FMN-dependent dehydrogenase family protein

MNTDPIDNRYATGERFTTNLGIWRAAREACTDEVWNYLNCGTGDEVTLRANTADFDKWQWKTPLFAGIGR

PDTATQFLGHSLSFPAFIAPFGGGEYMLDAEGHRATGRAARDVGIRQIVPVAAAHSLEDIATASGVAQMF

QVTFVGDVGAVVDMMHRAKAAGYEQIVATYSPIRQWRERMIEDRTRFAPGKAEANFGPGLSDPAALREQI

AFSQPRWGWAEAREAIARAPLPILVKGVMSADEAKQCLDAGAMGLYVSNYGGRSIDRQPSAISALPQVRA

AAGPDVPIIFDSGIRRGSDIAAAVALGANAVALGRAVGFGLAADGEAGVRRVLQILKDEYWTTLGHLGCN

STADLGPHVFI*

>gkv_129|gene_NONE|conserved hypothetical protein

MKTLTRLDLNDARRLIAGAIRHADQIGVPMCIAITDESGNLIAFERMEGGKVTSSTIAIDKAYTAAAARK

ATHEYGAASQPGAPAYGINSAIGGRLMIVAGGLPVTYADAVIGAIGISSGTPAQDLAVAEAALQFWQAAS

*

>gkv_130|gene_NONE|hypothetical protein

MRPVMERGLWRYLRLMVLWVMLLPFAASSLFAAGIMPTRAPSGAIMLVICMGEGGMMEVAVDPQTLLPIE

APDASDMDGGQHCYWAAAHVPFTPPETPPLRLPDAHRDTPAIAAQSTALRAAIATGLPPSTGPPVTL*

>gkv_131|gene_NONE|pepSY-associated TM helix family protein

MTIQTSGGLPPDAARRAKASNLYRAVWRWHFYAGLIILPFMITLAVTGGIYLFKDEVDRIVHADFMRVEP

GGAMLQPSALIAAALAAQPGTAVKYTDPARDDQSTEITVQSDEGAKAVFVNQYTGEVLEVRADRSTFAWT

VRYLHSFRYFGPNPRKIIEIVGGFSILMVLTGVYLWWPRGQKGGVVTLRGTPARRTYWRDVHAVSGIILG

AFIVFLSITGMPWSGVWGGKVNQWANSGNFGYPAGLRVDVPMSHDHLDHIAKTSWSMEQAQIPETAPPIP

DMPSIGIDIAVQIFDEMGLHRGYSVALPNGPTGVFSGSVYPDDLTQQRVVHLDQYTADALIDMSYADYGP

LGRWLEFGINTHMGQTFGLLNQIVLLLVCIGIVVLMVSAAVMWWKRRPSGKLGVPPMPSDRKVFIGLFII

LGIGGVIFPLTGITLAVMIAFDMLWQRISPAA*

>gkv_132|gene_NONE|zinc-binding dehydrogenase family protein

MRSAIHATFGLPEDVLTLGDAPMPEPAAGQVRIKTILSPIHNHDVWTVRGNYGYKPELPAIGGSEAVGIV

DALGEGVTGIAPGTRVAVASVHGTWAEYFLAPAAGLVPVPDAISDEVAAQLIAMPFSALSLLEELDVKAG

DWVIQNTANGAVGKTLAMLATARGVNVINLVRRDAGVAEMAALGIGNVISTAAEGWKDQVRALVGDAPIR

AAVDSIGGTATGDLTGLLGENGLLVVFGSMTGAPMQISSGDVIFKQVRIKGFWGSVVSATMPVDKRRALF

GELLTQAASGKLQLAVGGIYGLDQLRAASLAAQATGKTGKVLLRP*

>gkv_133|gene_NONE|organic hydroperoxide reductase

MKVFYKTRATSTGGRTGHTALDDGSLAFDLASPGSGKEGANPEQLFALGYAACFGSALELIAKQMKLDVT

AKTSVEVGIGQTASGGYALDIDIYAETTGITEDEAHRLIEKAHEVCPYSNATRGNIDVRLHVVVN*

>gkv_134|gene_NONE|bacterial regulatory proteins, tetR family protein

MDMLHLTPKATATRLHILKTGEKLVQSRGFSGLGLQQILQAAGVPKGSFYHYFASKEAFGVAMLQQYMVD

YAARFEVLMRASDSGRALLLRYLDAWITDPAHPDQPGWAEGCLVVKLSAEVADLSEDMRLVLADGITRIT

DRMAALIAAGQADGSVPAKADPQGLAVVLYQLLLGAAVMAKVTRTRAPLDGAATAARLLLACGDQPNT*

>gkv_135|gene_NONE|uncharacterised BCR, COG1937 family protein

MPKHPEDKKRAMTRLNRIRGQCDALERALEAGDPCGPILQQIAAVRGGINGLMAEVMESYIREEFAADAA

SGKADDLLALVRSYLK*

>gkv_136|gene_NONE|S-(hydroxymethyl)glutathione dehydrogenase/class III alcohol dehydrogenase

MKSRAAVAFGPGKPLEIVEIDVAPPKAGEVMIKITHTGVCHTDAFTLSGDDPEGIFPCVLGHEGAGVVVE

VGAGVTSVAVGDHVIPLYTAECGECLFCKSGKTNLCTAVRATQGKGVMPDGTTRFSYNGEPIYHYMGCST

FSEYTVVAEVSLAKINPDANHEHVCLLGCGVTTGIGAVHNTAKVQPGDTVAVFGLGGIGLAVIQGARQAQ

AGRIIAVDTNPTKFDLARQFGATDCVNPKDHDKPIQQVIVEMTTWGVDHSFECIGNVNVMRAALECAHRG

WGQSVIIGVAGAGQEISTRPFQLVTGRKWMGTAFGGVKGRSQLPGMVEQSMKGEIELEPFVTHTMGLDAI

NEAFDLMHEGKSIRSVVHY*

>gkv_137|gene_fghA|S-formylglutathione hydrolase|

MERVELHIAAGGRQEVWRHRAEALDCEVNFAIYLPPQADTAPVPVIYFLSGLTCNEQNFITKAGAQAFAA

QHGVAIVAPETSPRGDGVADDPAYDMGQGAGFYLNATEAPWAAHFQMYDYIRAELPALIEATFPVTDARS

IMGHSMGGHGALVIGLRNPGRYKAISAFSPIVAPTQVPWGEKAFTGYLGADRSTWAAYDATALLAHAAER

LPILIDQGSADNFLETQLKPEIFVAEAQRLGHPVELRMQQGYDHSYYFISSFIGDHIAHHAAALKGT*

>gkv_138|gene_NONE|glyoxalase/Bleomycin resistance protein/Dioxygenase superfamily protein

MAKMVHTMIRVLDEARALAFYDTAFGLRVKDRLDFADFTLVYLVNDETAFELELTVNKGRTEPYDLGNGY

GHLAVVVDDMPALHARLTEAGYAPRKMVDFAPGGEVIAQFFFIADPDGYQIEVIQKAGRYA*

>gkv_139|gene_NONE|ABC transporter family protein

LSAPILALDGVSKTFGSAENPIYATRGVSFGLFPGRSLALVGESGSGKTTVARLLMREYHPDEGQLLFRG

APVARAKGRALRDYRSAVQMVFQDPFSALNPTRSIRHHLERPLRLHRPDLDRAGRAAAMVELLGRVQLDP

ERVIEKFPHELSGGQRQRISIARALAVNPQVIVADEPTSMLDVSVRLGILNLLNDMKSELGLALLYITHD

IATARFVAEDIMVMYAGQVVEWGDVDAVLTNPQHPYTRLLLSAVPDPDKRLDGTPTGAMDDVDAIRARAA

KVQPAVRQIAENHFIRSTE*

>gkv_140|gene_NONE|oligopeptide transport ATP-binding protein oppD. domain protein

MTTLSIRNLSIDYIGPRSDFHAVKDVSFDVGRGEFFGLAGESGCGKSTIAFAISRLHRPPALIRKGSQIL

IEGRDVMALDARALRDFRWREVAMVFQSAMNSLNPVLTIVDQFYDVLKTHAGMSRDQARARAAELLALVD

IPAHRLDAYPHQCSGGMRQRIVIAICLSLNPKLLIMDEPTTALDVVVQHEILQRINKLRKDLGFSVLFIT

HDLGLMVQVSDRIGIMLEGELVEVGEAQSIYRNPQHDYTKRLWAAMPRLHGARLQEAGR*

>gkv_141|gene_NONE|binding-protein-dependent transport system inner membrane component family protein

VAVFKKLWENKKVLVGLSIVAALVLMAIFAPLLTEYSPTRRVGRPHEPPSWDHIMGTTRLGHDVFTRFLY

GARTSLMVGFGAGLLITIIGTTLGIIAGYKGGVVDEVINFFTNMVLVVPNLPLLLVLAAFIGQVSPLVIA

LILGFTSWAWGVRVTRAQTMSIRERDFVKSAEMLGEPQWRIMLFEIFPNLISIVGINFIGSVIFAVITEA

TLEFLGLGNPNTVSWGIMLYNAQNASALVVGAWWDLLAPCFGLAVLGLGLALINFAIDEMANPRLRTGTI

LGRWFGLIRSGEGKL*

>gkv_142|gene_NONE|binding-protein-dependent transport system inner membrane component family protein

MGFLLRRLVFYFAAFLVAATINFFLPRLMPGDPIEIMFSSAGSTLTLDNLNALRLTFGFIDAPLWQQYLT

YLKSVFTGDLGLSIRYFPLPVTDLLGRALIWTLTLVGIATVFSFIFGTLLGVVAAWRRGSKFDSFVSLLS

IFATSIPAVVVALLVLFLFGYTLRWFPNGYAADPMIDPAFSWVYIKSVLYHGTLPMLTLVFVLTGGFVVT

MRNNMINLLGEDYIVMGRAKGLAERDVMLWYAARNALLPTVSNLAIALGTVLSGSLVVEVVFNYPGLGNT

LYQAILARDYPVIQGQLLIMTGAMLVANFLVDLSYVLLDPRLKKG*

>gkv_143|gene_NONE|bacterial extracellular solute-binding proteins, family 5 Middle family protein

VKKRILLAALSATALTTAAPVSAEAILSLNAEAATTWVRNFNPFAQTTSRYTTMDFIYEPLVVFNRLQGG

TPHFRLAESYELSDDLTSITFKLRDGLKWSDGEAFTADDVVFTFDFIKENPALDFISVWGDLTAVEKVDE

TSVRFTLSQPNSLIANTIVEMPIVPQHIWSEVADPVTFANENPVGSGPMTEVTRFTPQVYEQCRNPHYWD

NDSLFVDCIRMPQLADNPQLLAALNAGTVEWSTSFVPNIDASFVATNPEHHKYWFTPSSLVSFQLSFTTP

DENNRKAFTDVNFRRALSMLIDRQTIVDIAGYGYPLINEDPSMLGELYSAYANPAVAEEFGTYGRFDYDA

GTALLDEAGYVDANGDGFRDNPDGTPITIDINVPSGWTDWIDAVQIAMETLTEAGLNVSMSTPDSAVWGA

DLIAGNYAMTLNALASASNPYFPYRQTFNPDDFGRSRFAAPHWSDEHMMELLNTYTTTQDADQQKAIMDE

VQMIVAQNMPVIPVYNSPAFYQYNTTNFTGWFNAENPVASPVVSRVNRTRLLQLLALRPVE*

>gkv_144|gene_NONE|glycosyl hydrolase family 3 N terminal domain protein

MTSTPLALSIGMPGLALDADEIAFLREANPYALFLFKRNLDNPDQIRRLCAQFRDAVGRPDAPVFVDQEG

GRVTRLDNGNWPLFRPLADFGALARKDMDLAKHALRLSTLAMGSMLSDLSMNSGAAPVIDLSRSYTHGVM

GNRLLDSDPDVVAALGRVIVDAFLEVGQMPMMKHIPGYGHAAVDPHMELPVVDASLDDLRASDFRPFKAL

KDTPWAMVAHTLYTQIDAENVATRSAAICNLIREELEYDGVLISDCITMEALSGTWPERIKGVLDAGYDI

ALQCQGELGDYQAAAVAARPLSDATLARIARGDARLGQAHVDARAVHAEVEEIFKTAALA*

>gkv_145|gene_NONE|conserved hypothetical protein

MDMLVNLFSPRIDALGLRAAKALDGSAITIRRAIPPELHILQDWTRAHFSPYWVSEVTVAMAHQPPGCLI

ATEGGSLLGFACYDATARGFFGPTGVAETQRGRGIGLALLHQTLVAMKSQGHAYAIIGAVGPVDFYAEAV

GAMPIPTDSGDIYQGLLRAPAAFKDPGQ*

>gkv_146|gene_NONE|bacterial regulatory proteins, gntR family protein

MPAPTDQLAEAQPLAQRIAAALQNDQSEDPLYRRLAAALRGLIAGGHLRNRDSLPSERRLAEATGLSRVT

VRKALEELVDGGLVERRAGARSHVAQDMDQSLSVLMGFTADMRRRGTVGHSVLLHKIMDMPTPDEVLKLG

ISLGEQVLRLSRVRLADGEPLAVEHAVVPAFAVAGALGDSLYEALRQNGYRPYRALQRLRVALADADEAA

HLLIPPGSPILHIERHTFMENGRPIEVTRSSYRGDRYDFVAELQIDD*

>gkv_147|gene_NONE|dipeptide permease subunit F-like protein

VQYLHQTPLLAMNPRWRIARILAEAGPVDAALQQQIGVLPDWFTRFPHELSGGQLQRVAILRALGAAPRY

LIADEITAALDPLAQVQIWQVLRDLAACGQVGLVAISHDRALLSRLTGNLLTIDPAPQTHSAGQSENWYY

TGL*

>gkv_148|gene_NONE|hypothetical protein

VRGWRHDHLPRLRRIQSRQYPPKGGFPRAGWAAQADDLSLSQMQGQVLKQYPAARGGG*

>gkv_149|gene_NONE|ABC transporter family protein

MLTISALSVGFRHYETLLRQGVAWRLRDFSLTLGAGQIVAVIGASGAGKSVLAHAVLGILPPNAVQTGQV

VSVKAGFIPQQISHLDPLARVGSQLAWAARRQGRAVDIAESLKAVGLSDRVQRLFPHQLSGGMARRVFIA

MALAGQPDLLIADEPTAGLDPENRDLILNILQAHAARGGAVLLITHDLLPALPVADRVVILHDGQMVSIE

QAVHFSGQGDALSAPYARALWRALPENGFIADA*

>gkv_150|gene_NONE|binding-protein-dependent transport system inner membrane component family protein

MNGRVQAIIIAALALGLIGGVAIAAYSLGDLGIRADFSARSLAPGGGHLFGTDQMGRDILARSLHGLALS

LRVGMIAAGLSVLIAAAMALLSGLSRHCDHVAGFVTDAMLAMPHLLLLLLISFALGGGATAVIIAVAISH

WPRLARLLRAEVIQASAAPYIETARALGRSRLFVLWHHILPHLAPQMLVGFLLMFPHAILHEAGLTFIGF

GLEPSRPAIGVMLSDAMRQISAGRWWLAVFPGLMLLAMVLAFEALGSALRRLTNPREAAC*

>gkv_151|gene_NONE|binding-protein-dependent transport system inner membrane component family protein

MDLQLTVAARLIRQVLLLATVALAVFLLMKTSPVDPIDAYLGPAIATAGPEQRAQIAAAWGLDQPAHVQF

LAWAGHVLRGDLGFSTTYHAPVAQVMGDRIGASLALTGLAWLLSGLLGFALGVIAAVRGGWIDRVIRLYC

YVLASTPTFWFAMILLMVFSVTLGWTPICCSGPIGVPPDQVSFVQRLQHLILPLTALTLFGVAQIALHTR

VKLIEVLQSDYVLLARAQGASALDIVWHHGLRNAALPALTVMMASIGELFGGAILAEQVFAWPGLGRASV

EAGMKGDVPLLLAIAVLTALVVSSANMLADWLYQLVDPRVRQ*

>gkv_152|gene_NONE|bacterial extracellular solute-binding proteins, family 5 Middle family protein

MTVLRHAFAALLLSTAALQAEVPDLVLAIGGEPDTGFDPLLGWGAYGNPLFQSTLLKRDANLATAPDLAT

TWSLSEDRKVWTITLRGDARFADGSPVTAADVAFTFNTAKGAAGAVDLAVMVRAEAISSDTVQIGLDRPW

ITFAEAFYTLGIVPAAAYGPDYGRNPLGSGPFRMVSWAEGEQLIVAPNPYYYGAPSPFGQITFLFTGEDA

GLAAAQAGVAHMVSVPAQLADAVPAHFHAVPVQTVDNRGLSLPFQPPHEVDGRNIGNAVTADPAIRRAIN

MGIDRDLLVEVALNGHGTPAFGPADGLPWAGAGDTIAYDLQGALNVLDAAGWQPGADGVRRKGALIAAFP

INYPAGDATRQALAEIAAELLRPLGIAATPVGGSWDAIQRVMHAEPVVFGFGSHSPYQLYSLFAARLGGV

DYMNPSYYANPAVDALFEQAQAAESLEASFPLWAAAAEHYGVAGDQAWAWLVNLDHVYLVSDCLDLGPTQ

IEPHGHGWPITASIANWQWTCN*

>gkv_153|gene_NONE|glycosyl transferase family 8 family protein

MQHVKGDGLLHIWGDGLSPAEVALIAQLQPVNPRLRLEFSALSKAEMAGAKGPDARISGASMGRLLIPKK

LDGRVLYIDGDTRITADPSQIFDLDMQGKPLAAVRDYVVSKWCRNGAPPDKPRVRELRQLMGQEDISHYF

NSGVLLIDTSAVRATPALFDAMTDVIRASASPWGDQDHLNSIFTGNVRLLDPAWNSSWSRTREQRAFIRS

SGALDNELTGLSDVIVHFHGSNKPWLGPRYDFWSRRGRAVMAYRRVQKRFQRAFPLLRF*

>gkv_154|gene_NONE|hypothetical protein

VQASSKLWREICRVKKQTSGLFHYFGGNLRRRRYDIERAQNVTVLEGQCGPQREMAVVLLFQPDGLLDSS

LLTLTELNALGIGCVVVSNAPLSLDDRNALLQHSYLVIERPNVGYDFGGYREGVLTLMARKLPIDALYLM

NDSIWFPVHDAKDTIAACRATNADLYGLHMSTVSRHKSRSYVQSYFLRFSHKILSSKSFMRYWEKLHLID

NKSTVVRYHEWHLAQYFIKRGFSIDALVHSRDLISTIMNITDADEMRLMLEHQSAICPKEAKYIRPVLAR

SKDALEARDRLMDDIKARKIFVTLTTVHPILLLRMKLPFLKKMRRGDFASQRAALIVLGLTKGFNPAVRA

EIANWDSRRPEYRDTVKLASL*

>gkv_155|gene_NONE|hypothetical protein

LPVIDARFAPDVSAALAEIGRSWAHLPLVFGPSQDLRARRFCASNACHGYEAGSLLQARQHALP*

>gkv_156|gene_NONE|ABC transporter transmembrane region family protein

MQLTQKIMSRFVKDDVDGNVVKRLLQYGVATQGRYYAIGILAMVIVATSAGLTAWSMEMIINAMSNPNDR

AQLATVSMMVVGIFALRGIGAYVQAVAMAKAGNGIIADQQRNIFNKLMEQGVDFFNLRESSDTLMRVTQS

AQAARGLIDIIATSAVRDSLTLISLLVVMVYQQPVLTLAAMTVGPMAFLMLRTLVRRVRDVTSRQMMSLA

EILRVLQETSAGIRIVKIFALEDLMRGRMSAAVRAVEKRSNAVTRLESITMPVMDILAGLTIAGILWLST

WNIVGGADGASAGQLMSFITAMLMCYDPARRLSQMRVRAEAMLVGVRMLFDLIDMKTSLNENPDGPALKP

GPAHIRLEDVTFAYGERKVLQNVDMDIPAGQLTAIVGLSGAGKSTVMNLIMRLYDPTEGKVTIDAQDISG

LRASSLRRALAYVGQDTFLFSTSILENIRYARPDASDDDVKAAAEAAFAHEFIIALPQGYQTQVGENGAF

LSGGQRQRISIARAFLKNASILLLDEATSALDAISEEKIRDAVQVLGRGRTRVAITHRLSTIMAADLVYV

MEDGRVIESGSVDALLRADGPFKDLYDKQFG*

>gkv_157|gene_NONE|phosphomannomutase (PMM)

MVPKFGTSGLRGRADALTADCITAYIQAFVAACPIGNGVFVARDLRESAPRIAKDVIAALRQTGITVTDC

GCAMTPALALASSRAGAAAIMVTGSHIPAAYNGLKFYTPMGEITKIDEAAILAALGGPAITRPLGPLRYT

DISAAYRTRYTTAFGTAALVGRRVGVWSHSAVGRDDLIAILRALGAEVVEFGRADHFIAVDTEAVTDTTR

ARLRAAARMYRLDAILSMDGDGDRPLMTDAGGTLIAGDILGQITARALGATSVVTPISSNTGVEALDLHV

LRTRIGSPYVIAGMQAQPRAIGYEANGGVLLGYDAQCAGPLPALMTRDSLLPMLVALIAAAGGTLAARVR

AEPARFTASGLLPDIDPCAAQRLIADLQADSRAFLAPFGLEPAYINRMDGLRITALCGQILHIRPSGNAP

ELRLYTEAASPRSAARLLKAGLQHLKARLVQQVVIGVADL*

>gkv_158|gene_NONE|mannose-1-phosphate guanylyltransferase/mannose-6-phosphate isomerase

MNRNIHPILLCGGAGTRLWPLSRRDYPKQFVRRTGAESLLQGAARRVSGPLFAPPIVVTGHDFRFLVIEQ

LAGVGVQPQRVLIEPEARNTAPAILAAAFALSQSDPDALMLVAPSDHVIPDTEAFRDAVAKAVPRALAGD

LVTFGITPTRAETGYGYLQLAPDAEPRADAPQNLLRFVEKPDATRAAQMIASGDFLWNAGIFLFTARMLI

AAFCAHGPAVLIPVEAAVRAACCDLIFMRLDPVAWATAPDISVDYAIMEKASNLAVMPFSAGWSDLGDWD

AVWQESGPDARGNVASPHSTAIACADTLLYSTSAGVELVGIGLQDIIAVATPDAVLVAHRSQTQRVKEAV

AALQHKGAVQATQFRTAHRPWDENPHGLWAKRIEVAPGTTFNLQPQHHAATHWIILSGVAQMTHRDQVHL

LCKNESLYVAAGGLHRLDNPGNVPLILLAVQTEAEGGVTTNHPQSAVG*

>gkv_160|gene_NONE|xylose isomerase-like TIM barrel family protein

MKTSIATVSISGTFQDKLAAIAAAGFHGIEIFEQDFLASDLSPREAARMVRDHGLDITIFQPFRDFEGLP

APLRQRAFSRAERKFDLMAELGTDLVLFCSSVHPAALGGIDRAADDFRALGEIAATRGIRVGYEALCWGK

HVDDHRDAWEVVRRADHPNIGLILDSFHTLGRKIDPETIRRIPGDKIFFVQLADAPAIPMDLLYWSRHFR

NMPGEGDLDVTAFTRAVLATGYDGPLSLEIFNDQFRAGRPRLVAQSGYRSLMALMDQVRRAEPSLSVPLP

NMPAPAAVRAVEFLEFASSKADAAALDQVLGAAGFSVAGRHRSKPVTLWRQGAVNLLVNTSSADFNLTSW

STHGTTVSEIGLTMPDAKDTATRAMALGAQAHVSAPHATGERDFPGIRHAGGSVLRFLDAGQASIWDSDF

DVTAPAPVGIGIQRVDHIAQTMAYDEMLSWSLFYTTLFEATRAPMVDVIDPDGLVRSQALQSGALRVTLN

GAEARRTLAGQFIEETYGASVQHIAFETQDIFATAAALAARGFPVLQIGDNYYGDLAARFGLPADEVAQL

QALNLLYDEDGAGRFWQVFSRPLAGGLFLEIVQRAGGYSGYGGPNAPFRIAAMKRHLRPAGLPKR*

>gkv_159|gene_NONE|shikimate / quinate 5-dehydrogenase family protein

VIRLGLIGDNIKQSQSPALHRLAGALAGLDVSYDLLIPAAQNRDFDALFAWAHDNGYHGLNITYPYKERV

TPMLQIPQPAVAALGACNTVIFGDTPVGHNTDYSGFAAAFRQRFGGAKPGAVAMAGAGGVGKAVAFALAD

LGAARLAIYDPDTARAQALITALAAYQPSLDLRLAPSITAACEGARGLVNSTPLGMGGINGSAFPEHLLA

GRDWAFDAVYTPVETQFLREAQAAGLDILTGYELFFHQGIDAFRLFTGASVDPAALRRALLAPAA*

>gkv_161|gene_NONE|bacterial regulatory proteins, gntR family protein

MTRAENDGESAAGLTISENAYRRIRSDIIFGRLRPRQKLKLDGLRDSYGISISTLREILNRLSSEDLVVA

EGQRGFEVAPVSVENLKEVAALRLLLETHALEQSFAAGGVEWEGDIVASHHKLATLERRMATGDMSQTEL

WKRYDWEFHQALISACGSQVLMDTHAAIFDKYLRYQMIALSHRGDIAAGEHKILLQAALDRDAPMARAVL

ERHVQGGLDHALATGTIA*

>gkv_162|gene_NONE|bacterial regulatory proteins, gntR family protein

VTDADPLGLTAGESAYQRIRSDIIFGRLAPALRLKLDQARQHYDISVSTLREILYRLCAEGLVQAEGQKG

FTVTPVSQQNFRDIAAMRDFLETYALRQSFQRGDVEWEAEVLAAHHRLSRLEAKMLAGGRDHAADWKRYD

WAFHRALIGGCGSQVLLATHARIFDQYLRYQIIAVIFRGEVAADEHRALLDCALRRDADAAAAILSRHIH

ACVEHTIRNGLLPATPLS*

>gkv_163|gene_NONE|bacterial extracellular solute-binding protein, family 7 family protein

MTHALTRRLLLSAGAALALMTSAPAMAQSNVPLRFSAVFSQQDIRAEMMERFAAALGEGFDFQGYYGATL

FRQGTELVALQRGNLEMGNIAPQDISEQLPEWSVLTSAYLFRDADHLVTFFQSEAGEQMKQLAEDRLNIR

VLGPTYFGVRHVGLRGDRAVNTPADLAGVRLRMPGGDSWQFLGQALGANPTPVAYAEVYTALQTGAIDGQ

DNPMPNVQNMKFYEVMDQIVKTAHLVGFDVLTISKSSWDSLTPEQQAQVQAAADEAIAWSNAQHLQNEAD

LIAFFEGEGLAIVEPDLDAFRTHAQELYQTSGMAARWPEGIVEQINGL*

>gkv_164|gene_NONE|tripartite ATP-independent periplasmic transporters, DctQ component family protein

MRLSAIGGWLVRRAENIQALMLAVMFFAFIIQVVFRYFFNLPTGWTSELTVIMWLWMVLWGAAFVAREDE

EIRFDLLYGSVRKGLQRVFVIATALVLITLFLVSLPASWDYVTFMKIQSSAYLKIRFDWLFSIYIIFVVA

VVCRYLWLLWQAVVVRRTPPATAEQDDITGHSI*

>gkv_165|gene_NONE|TRAP transporter, DctM subunit

MSLTDPFTACIVLLITLAVLGLPVAYAMILASILYLLMAGLDMGTAAEQLLNSMYTSYTMLAVPLFILAA

ELMNSGSMTTRLTNFANALVGRFRGGLAQVNVLQSLLFAGMSGSAIADAGGMGKMMMRMMTQEGKYTPSF

AAALTAVTAVVAPILPPSIPMVIYALVSNVSIGYLFLGGILPGLLISASQMLIVGWSARRNNFPTEAPVP

VRELPMITLRALPALLLPVVLIVGLRGGVMTPTEAAAVAAGYALFVSVVIYRDVGLREFYASLLSAARTT

TSIGMLIAAALVFNYIVTVENIPNSISRFLLGFDLTPLTFLLIVNVILLLIGAVLEGSTIILIIVPVLIP

TAAALGIDPIHFGVVVVFNVMIGLVTPPYGLLLFVVKRVSGASMGAILRDTMPFLLGLIAALLLITLIPD

IVLFMPRMFGYSG*

>gkv_166|gene_NONE|3-dehydroquinate dehydratase (3-dehydroquinase) (Type IIDHQase)

MTNTIYILNGPNLNRLGKREPHIYGHTTLAEVEALCRNTAGADVQIRFHQSNREYELIDWVHEATEEGAA

GIIINPAAFTFTSIAILDALKQFDGPIIELHISNVHRREAIYHNSLVSKVATAVMAGLGPRGYATAVGAM

QDLIAAKAQS*

>gkv_167|gene_NONE|SMP-30/Gluconolaconase/LRE-like region family protein

MTAPVEVVHHFTRDKLGETPLWCDRRQQLLWVDIEQPRLQSFDPATGRHQALGVDCDWLGSHALCADGRR

LIAKDLALHLMDEDSGKMTPFAVIETGVDNRLNDGRVDRWGRLWIGTMDNQLHRPQGALYRVAGSGRVDK

IAGDVIVSNGIAFSPDGRQMHFTDTRRYMSWVYDIDPDDGEITGRRLWADYSATKDRPDGAAMDVDGCLW

AAFFGGGKIVRYRPDGQIDFEIPLPISNPTCLCFGGPDLRTLYITTAFKFLNSTQLQREPLAGALLAIEG

IGQGLPEHRFTL*

>gkv_169|gene_NONE|bacterial extracellular solute-binding protein, family 7 family protein

MLRSLFLAAGLAVAAAAASAQPINLRFADSTTADAPRSRALVEIFAAEIAPDFTFEGYFGGTLYRQGTEI

VAVQRRNLEMALMPPSDFAEQVPEFGILTAAYLVRDADHMRRIFESDVGDEFKAMARERMGVHILAPAYY

GTRNLNLRGTRAVNTPADLAGLRLRMPGGEAWQFLGSSLGANPTAVAYAEVYTALQTGAIDGEDNPLSNN

RIMKFYEVTDQIVLTGHNVGFGLLMINAALFDSLTPTQQQSLQDAANKAFEWSNAEYIREESELLTFFEG

EGIRIHTPDVDAFRAYSNEKYLNSDFSRNWPAGLLDRINAL*

>gkv_168|gene_NONE|bacterial regulatory helix-turn-helix protein, lysR family protein

MDSRHLHYFIALAETLHFGHAAARMNMTQPPFSRQIAQLEASLGAQLVARNSRHVRLTAAGVHFLQDARR

ILADLDKAGRDARLIAAGQKGELRLGFMMHAADSILPDLVRQYRAAHPDVRITLREIIPADIASHVLQGE

IDAGLTFASPNVTGLTRLPLLSDVLQLIVPKDHPLADRADACAHDLQDQDIILAPAHIAGALRDAVMAYF

LQAGLVPRIGLEPGLQHSIVQLVAAGLGVALVPASVSRTARPDIATIPLIAPPRLDIVLLTALRNTNPAV

AGLESIAVTMR*

>gkv_170|gene_NONE|auxin Efflux Carrier

MAPTLVATILTVSVLFAVAIILIEIALQTGGSRRAIFARTGLSLVKNPLLIAPVLGLVFMCAGWTLPQPA

DAFLKLLGGAASPCALIALGLFLAGSAGQKPPQGAGVEWGLIAAKLIGQPLVTWGAAMALGLGAQEAFLV

TLLSALPTGTGPFMLAEFYGRNGLLTGRVVLKTTLLSIVTIAALLALG*

>gkv_171|gene_NONE|alkaline phosphatase family protein

MKTIKALPLALLASTAFVAPALAQEQAKNIILLITDGAGPESWSAGTYYRFGALGHEVYDGFDLKAYMAT

HPLNTSSEPTFSDEGTVTFDPAELWTDTAVDTVYEGALGNYAGYFSGYDYARADYTDSAAAATAIASGQK

SYNNSINWSNNGESLRHIGEYVVDSGRALGVVSSVQMSHATPAGFLAHNVSRNDYAAIGAEIVESGLATV

VMGAGHPLFDAAGQAVATPSDNAYRYVGGRDVWDRLVAGETAYQLIETKDDFEALANGDLALTGDKVFGL

VQNSATLQFNRPGVGLGDWLENSPDLPTMTRAALNVLAADEDGFFLMVEGGAVDWAAHANNLPRLIEEQI

DFNMAVEAAAEWVEANSSWEETMIIVTTDHGNGLLQGPLSDTVAYQPIVNQGQGAMPLVRWHSDTHTREL

VPVFAHGAGSEYFRSIAAEETGLSRYTTDAASQIWFDNTDIFRGALTAMGIDEVAAQ*

>gkv_172|gene_NONE|hypothetical protein

MTLISRRLFLAGVTASVAGLPALAQIRETFLTLTPAGAVAEVTRAQREGEILTVTARFTAFEPDYAGEVI

YENLTPSEVIQGVYLKTGDRDFGVWSEGGTLQLPDALHLAPHSGGPNPEIVGEWTAVFIAPTLDVREITL

LLPGMLPIGHFIIRDR*

>gkv_173|gene_NONE|putative membrane protein

MAAVINAEGEIAALFAPYLAHDRNRRLRAALLIGATAALIAALIAWFFGLVPAMIWGVAIAAFALVVLAA

WVWLRRDAPTALHLARDFDRALGRAATLATGLEHEARAGAQTLFTRRVHAEALALAPRFPLIFKSVVSPA

LHVQRRAAVALAIVAPVALLILISPRAQAVAPPPLANAAALAARVADEAARRGDAGLQSLAQQMDDLVRQ

IAAGTAGPEASQQVQALGAAINRALGGTADGDPGIAVSRTLAQQRLADWAGQEMTFSANAPPPATRAAPR

VTTDLAARGGATAGEGERSRPNRLDASLEVDALDLTTADQAQDSRSASAPGQSTFIPPQLREGAPQPGLA

IDMAGAGDAPRQESMQATGGGGGAAPPGAPPPPPPAATGQSLAIIYSGITPRDTPVHYLTAADRAAGDYA

DVTVGGLPAYARQTLPPAQRQGFDAADARAAARFFARGQEAMGPETLGPEKLGATE*

>gkv_174|gene_NONE|von Willebrand factor type A domain protein

MSFLHPLALLLSIPAIAITLLHGRRQRRVTVSGLGLWQALAGTQQAVSPARVWPRPSWALFWQIVALLAL

VLALARPFWGQVAGDQHWLVIADDSALTARDDLAGALARLDHAIPAGRQVSVITTDSAALPLIVQQSARA

GLLAGLAPQQGEGNPDWAGAFALAQRLDPGRIVILSTHAPTGAPDGASHIPLAAHENNAAPAPEFRFAPP

TFAAADPMWHAAFAAYGAVPADAADVALLRGRDDPARGGLRLILPEGVRPGPHHITYWQDHHPLLRGIDW

PALVLDTAAPFTPAADEITLLANDQGALMAAGPRHLRLGFDPAQSNMAETLPLLLAGRVLDWAGLSGARH

CLIGQSCAMARALIGQVLTSADGQQMPVDAPWVIAKQAGVYGFEGRAVLQISPAPLVAPIAAELPVLRFP

LDLTAWFLGLAALALVAEGWLARRAGAGRGLLIWRGAALGAVALSAAWPVLPLPRMAPSTAELTSSDPMA

LHLAAAAMPHGGALRLTTRALPDDLAQPLARLAASGVTVDIAAPEPPAGDVALTAAYLPQVIYAGDQVVA

RLGITAQRATQATLTLTAGEQQISTMVDVQLGANRVDLPLAMADTGAVDVVIELQAVGDPAPDNNRLVLP

RDTLAAPRIAVVAQDDGPRDAVAGMLIDQGFDAIPLTPGRVPVNPDIWDRYDAALLLDLPAIALEMRQSE

LLASRVQDHGLGLVIAGGPHSFGPGGYLETPLETLSPLSARLPHEGPGIAMVFVLDRSGSMSQTVGDVTR

LDVAKQAVSAAANLLDPQTGSLGVVMFGSEAEVALPLGPLPDAAGIAAALGHLQPGGGTNIYPGLQLAFQ

ALRASDADARHIVVMTDGMSDEADFPGLLAAIRAEGITVSSVAIGSTSETSIAEDIALLGGGRFHNTRDF

GALPSILAQEALMLRGAVIEEGQFPVMAEAAVPYTPGPISGLVRTRLKDEATPLLSAALPDGTALPLLAR

WHYGQGQVVALATAMTGRWSADWQQQGLTPLLLAQALRQVLPAPQPEGIRRAGDTAVVSLLAGQQVMLDG

TALPQRAAAGGLVEVTVPLGPAPQGLMLHDGDGMRRLGLPGIIPTADMAQDYSAALAAEIAARPPTQARA

ATVDLVLRPWLIVLALALFLAEILRRYGYVLPRLTLHRKG*

>gkv_175|gene_NONE|ATPase family associated with various cellular activities (AAA) family protein

MTVSSIPALDDPAAFALVHGKLVAARQMINEIVLGQEAMVTHLLTGLLAGGHVLLQGPPGVGKTMVVRTI

AAATGLDFARIQFTPDLMPADITGSTVLAVDADGRNTLEFQPGPIFTQLLLADEINRATPRTQSALLEAM

QEFTVSAGGKTMRLDRPFFVLATQNPVEMDGTFVLPEAQIDRFLFRLDVDYPDAKTLTRILGGRAEVAVA

RASSVMSAADIVQLQDLAAAMPVASHLLAAIAEFAVATQPSASRDDRVKRYLRMGLSPRGAQAFLAAARA

HALLEGHSHVSFDDLRAVLGAITRHRVQLNFEGQAAGLSVEALARELFDRIARA*

>gkv_176|gene_NONE|conserved hypothetical protein

MQPIAPATLERLRHLRFAPLYAKPGAGVGERRSDQRGAGLEFIDHRPYRPGDDIRDLDHRLMARLGQPYL

RSYAADRQLPVSVVVDGSASMGAARRDVALQLAAMLGFVALAGGEGLRVWRGGVPSPLLSGVQRAPLLLD

WLARAGAAARFTDHLNTLARDLPRGGLVLLISDWQDAAALDHLDMLRRAGHEPVVIRLTTAVEVDPTRLG

SGVLVLADAETGQELTIALTPEMLAQYQALWDARTQRLAKGWFFDLPEGTDPGDVIAQMRIKGLLA*

>gkv_177|gene_NONE|tat (twin-arginine translocation) pathway signal sequence domain protein

MTLTRRQALLAGAALPAAAALPALIPAAAHAQAAAGGPARDHALGNFRVTSLLTGAMPMDNPREIYATDA

SEEDFAAVAEAGFVPVDRSVNSFTPIVVNTGAEVVLFDTGLSPEGIVSALQGAGYTPDDVTHIVLSHMHP

DHIGGVMGADGAPVFGNARYFTGQQEFDFWATQANERFDLNVRPLAERFTFLDDGDTVVGGITATAAFGH

TPGHMAFHLENEGRRLLFIADAATHYVFSLANPEWEVVFDADKAAAAATRRRLLDLAATDRIAIAGYHMP

FPGIGFIETRGTGFGYVPASYQFG*

>gkv_178|gene_NONE|cobW/HypB/UreG, nucleotide-binding domain protein

VTVLSGFLGAGKTTLLNHILNNRDGRRVAVIVNDMSEVNIDADLVRGGTELSRSEEKLVEMTNGCICCTL

RDDLLVEVRRLAAEGRFDYLLIESTGIAEPLPVAATFDFRDALGESLSDVARLDTMVTVVDAVNLTRDFS

SHDFIADRGESLGEGDERTLVDLLTDQMEFADVVVLNKATAAGPQRLDQARKIVRALNPDARVIETDYSR

VDGDAIFDTGLFNFDQAHMHPMWAKELYGFADHVPETEEYGISSFVYRARRPFHPQKIHAVLNGDLPGVI

RAKGHFWIASRPDWAVEFSLAGAMSTVVPLGSWWAAVPQERWPTHPDSLAEMRARWDDVWGDRRQELVFI

GADMDKAALVALLDAALVDTDGFQPKVWAKLPDPFPQWRR*

>gkv_180|gene_NONE|hypothetical protein

MPARRNIATSLRRRADPRPMDQFDRLPPDVRAWLARAALPWSPRSVQKLWRRALRECGGDPARALARMDL

AEARMLAKDCPKIWGRAHPLISAC*

>gkv_179|gene_NONE|DNA alkylation repair enzyme-like protein

MRRITDLDPAYVAALNAGAIPSATLTEGLAIDFAALLQAAVPALPSAACAQMHAAAGEGITRRMAKAAQL

IADHQGLAALPALMDHTSDTVRGWACYLIGGADSLALPARLEMIRPLADDPHFGVREWAWMAVRPHIAAD

PAAAISYLTGWTDDPSARIRRFASEATRPRGVWCAHITSLRQDPGSALSLLQPLRADPAAYVQDSVANWL

NDASKDRPDWVRALCADWLAESPAPETARICKRALRTVNKR*

>gkv_181|gene_NONE|hypothetical protein

MSPSPPEDGSIRIFQANGAQTQVLDDLGFAHGAIMADGRAGHFISMPRFHVYPTTFAAMNDTLDQLEQHF

*

>gkv_183|gene_NONE|conserved hypothetical protein

MWRERLKDFARRLKRDLVALWIAARDRRTPLAARGLAIVVVAYALSPVDLIPDFIPVLGYLDDIILVPLG

LALCIRFIPAVLMQDFRALAAARGRVPASRAGLIAVLLIWALLAAWLVSLLF*

>gkv_182|gene_NONE|helix-turn-helix domain, rpiR family protein

MQDSDLSQRIRNQIGGLPPTMRRVAQYMDRNRPEVLAMSAAELAAALETSDATIIRTAKALGYDGLSDLK

RLLTREMSAGTPVENFRRTVSASHADQRRAALRSLQMTGDVLTNLRGEENLAQLDRMISLLDQAQRIVLF

GIGPTAFLTGYAAHQLARNGRETLLLNRTGRDLADQLLGLRAGDALLMISYSQPYAEALATMEEALMQAL

PIMLITNRTEHQLSAKAMETLVLPRGGAQGTAQNGATFACLEALIIGLSIRDPDQTHQGLNRLEQLRASI

DRLG*

>gkv_184|gene_NONE|ABC transporter family protein

VPDQPILLSVRDLRSDLTAPLSFDLDAGQCVAVTGPSGAGKSLRLRMIADLLPHDGALLLRGVACADMPA

TQWRRQVRYVQSEPGWWAPLIGDHIGPSPLLGALGLPDDILTRRVDSASTGERQRVAILRAVADRPAVLL

LDEPTAALDEAATRAVEGLVRQLMGGGMGIVLVSHDAAQVARLADQVISLAGRG*

>gkv_186|gene_NONE|uncharacterised protein family (UPF0014) family protein

MGVTFSVFDLSVAGLLVICAAAASWALSLGVHRQLLWAAGRLIAQLLLVGLVLRALFASESMLLGLGLVV

LMVGAAIYETAARPQQRMAGRFNAVASAAGIGASVMLIVVLAGATLRHDQDVLQPRVLVPIAGIVLGTAM

TAASLALNTLLDNLRRERLAIEAQLALGVGRFRALAPVVRGALHNGIIPTLNQMAGAGIITLPGLMSGQV

LAGADPVQAAYTQIFLSLLLSVAALISAAGVVGVAILRLTDHRDRLRLDRF*

>gkv_185|gene_rfbA|glucose-1-phosphate thymidylyltransferase|

MTKRKGIILAGGSGTRLYPITVGVSKQLLPVYDKPMIYYPLSVLMLAGIREIAIVTTPQDQDQFQRTLGD

GSQWGLSLTWVVQPSPDGLAQAYILCEDFLDGAPSCMVLGDNIFFGHGLTDLLIAADAQDSGASVFGYHV

ADPERYGVVSFDADGRVESIIEKPEKPGSPYAVTGIYFMDGTAPARAKQVQPSARGELEITTLLETYLHD

GSLSVQRMGRGFAWFDTGTHSSLLDAGNFVRTLQLRQGMQAGSPDEIAFEQGWIDAEGLKARAKLFGKND

YGKYLASLLNN*

>gkv_187|gene_rfbD|dTDP-4-dehydrorhamnose reductase|

MKILVFGRTGQVATELQSLVPEVVFLDRTQADLLDPASCVTAINRHRPDAIINAAAWTAVDKAETEEGSA

ALINGDAPAAMARAAAALDVPFIHISTDYVFNGGGNTPFKPDDPTAPLGAYGRTKRLGEVGVEAAGGRYA

ILRTSWVFSAHGANFVKTMLRLGAQRDRLNVVADQIGGPTSARAIAQACLRMAEHLAAAPNLSGIYHFSG

TPDVSWADFARAIMAAAQLPCTIHDIPSTDYPTPAARPLNSRLDCSSLARFDLTRPDWQQDLLVVLNELG

AVK*

>gkv_188|gene_NONE|hemolysin-type calcium-binding protein

MVLTGARAIGTSSDTYRLVWYQNTGSQSTTDNFLNGQTWAIQTYNAANDPDGNPSVGEDGWTTAYTYSQM

TPHPDLVAGLGSGTGYIVFSGNNGNWFILDINADFNTTAETLYYYGPVTPNSLTFTQVQAVCYLRGTMIM

TDRGEMPIEQLREGDRVVTRFGGLREIKWIGRQQFRGNKTFGNEAIRFAPGAISQNMPQRSLYVSAGHSM

LVGDVLVLAQDLVNGITVTRESSRDTWDYFQLDLGTHDLVLADGAWSEVFADCGTFRSKFDNAEDYRRRF

PTNIAPLLPQFCLPRPNDGAALRNAIATVAQRALDKRGAEVMGRLDGQVEIIASPFRVEGWARDQDFPNQ

PVALEILLDGEVIGATLACLPQHGNASRTRQRFVFEGDAALTEAELRRVVVRRTLDGVVLNSQTADQMGQ

MRGHLDLVSATGVIEGWARDLEFTETPVTLEAWLDDSYLGTVTANKARRDLTANHGDCAFTLRLNRSFTA

AEALRVTLRRVGNDAQLNRSVNTKVPQELAA*

>gkv_189|gene_NONE|bacterial regulatory helix-turn-helix protein, lysR family protein

VANSFVHVHLVALRYFSETVRSGSMRQAGEVLAVSASAINRQIMKLEDQLQCRLFERRAEGVRLTAAGEV

LYQYVRRLDRELERAIGQIDDLRGLRRGHVHIACEAGIGRDFLPAVLADFHASHPGVTYKVEIKSALEIL

EQVATDEIDIGIAMSPPTRPEAAIGGRALMPLGVIAAPGWPLAAKSSLRLQDLGGERYIRAKDGMGGGYG

WQKIIDQGAPQAAILETNSPDIMSVMVKAGLGIGIRSPIGIMSDLSRGELSFVPLDDSLAPHPSLTLFVR

GGRILSSGGAVMLEMLREALPAFSQRVWDLAGAQMPLGGISTGAS*

>gkv_190|gene_NONE|FAD dependent oxidoreductase family protein

MQASTRSLTADFTHFQPADTAPPAPIPQLPDAAARLAALTAQVTADLAMMGYATKPWVLPRAHLGQVVPD

VVIIGAGQSGLALGHMLKRRGVTNVLLLDRNPAGYEGVWDTYARNYEIRSPKTITGLELGIPSLTVQSWF

VALHGQAAWDALTRVPRAHWMDYLRWYRQIADLNIRNDINVMDIAYDADGVTLTLQDGAQVRTRYVVLAT

GMEGGGNWVVPDFIRNALPADRYNHSCEAFDASRFAGKDIGVLGAGASAFDATVAALDAGAASVQTFMRR

PAISVLDLVREFENGGFLDHAHALSDETKWELGLFLSGLSQAPAEHHFYRALAFANFRFHSGAPWLDVRM

DGDRIAVTTPKGSFHFDHLITATGVTTNMMLRPELHRLAADALLWRDRFTPPDGNTASARLNFPYLDDYY

RFQPKTAGAAPGIDRIFAFNALAMPSMGGLAAVSISSHRFGTARLASGLTRALFLDQEAELIPTLAQVDT

PCITLTPYAREMLGMLDCD*

>gkv_191|gene_NONE|creatinine amidohydrolase family protein

MTRKIWWNDFSASDFDAIDPMKTIAILPIAAVEQHGPHLPVGTDVIINTGHLEMLAKAAPADLDIRILPV

QPVGKSNEHIWAKGTVSHEAKTLIDSWVEIGLHVARTGIRKLVIVNSHGGNEEIMGIVGRELRVRCGLFV

VKTSWSRFGAPAGLISDTEARQGIHGGEVETALVLHFRPELVDMAKAGNFTSVAAAEEVDYTYLRPTGTH

AWSWIASDVHPSGAIGNATLGTAEKGAAIAQNHVDRFLDLLAEVLRHPVMPEA*

>gkv_192|gene_NONE|creatinine amidohydrolase family protein

MADYWWNLSTAEFAGRDMSQAVAILPIATVEQHGPHLPVGVDSMINAGIIARVMAQIDPALPVFTLPMIP

VGKSTEHLSYPGTLTLSWELVAKIWFEMGECVRRTGCRKIILFNSHGGQVALSEIVARDLRAKLGMLAVA

ATWFRITPVEGIFSAYEDLHGYHGGEIETSMMLALHPELVDMTKAEDFRQLSQVMVDEAEILRPGLFGWM

AEDLHPAGVSGNAAASDAARGEALVQRAADRLVQLIHETAAFPLSRLSQPADYQVSPQ*

>gkv_193|gene_NONE|ABC transporter family protein

MSRDYLCLQHLNAHYGKSLAAHDISLNISKGELIALLGPSGCGKTTTLRMIAGFVQPTSGEVIIDGKDVT

RLAPHKRNIGVVFQSYALFPHLTVLGNVAFGLSMRAVSKDERNERARAALDLVGLGRFADRYPGQLSGGQ

QQRVALARALVIEPSVLLLDEPLSNLDAHLRGEMRSEIRALQQRLSITTVFVTHDQAEALAMADRVVVMN

AGEIVEIGAPRDLCDNPRHAFTASFLGERAVIEGATAGDIFTAPGFHWQGAPAGSTRAVLRAARLRFDPN

AGPKVLEGTVIASAYLGDAVETDVATASGRVRLLTPSDQPVPPVGASCAVHALPGSVTFI*

>gkv_194|gene_NONE|bacterial extracellular solute-binding family protein

MSIMKRRTFGKLVLGAGVAAPFHFVRSAVAQPQPGDELIVGIWGGAQERIVREFVEPALVDKYGCKVSYV

LGGTGERRARAYAERGRPSFDVIYLNIYESRQAVTDGVTQAPTDAVANAEYLYPLAKQGGYGVAFNPCTI

VYKTDKASSPITSYADLFKDEWKGRFASPTVPGMQGIAALLMLAKTYGGDEFNIDVGFQKLQELKPFAAI

QNSAEAAWQMFEQDIADITIEFGSLANMAKDSVLPGITIADPVEGICAAMNVACITTGTQNQVLAEEWIN

LHLSEPCMQAYMRQTYYSPTVSNVAIPADIADKILTPDQVSRLASFDWEHIASAQAEWSSRFTREIAG*

>gkv_196|gene_NONE|binding-protein-dependent transport system inner membrane component family protein

VISSRNIGRGLATPVTLVLLVAFAVPMAVVVLLSMHAYSDPFGPLLRPPSTAQYAMVLGDFFYLRVLLET

LTLAGGVTALSVIIGYPLALWLVSVPAKWRALAFVVILIPLLTNVVVRSLGIVLLLAPDGILNGVLGWLG

IGPFRNMLYNYGAVCIALAQVFMPYVVLALYDVLQGTSPRVKEAAESLGASPSMVFWTVRFPMALPGLRA

GIVVVFLMASTAYVSATILGGGRVLTSGMLVYREAITNLSYPIAAALTLVMTVASLAFSAVVLLVFRKLT

PWTRAGEGRANSSLPAIPVWLVRGLDLIGPLISRGLLVIAIILLLLPLYLVVMQSFNDVPQASSAKFVGF

TLKWYEIVLQNGNYTAAFLNSVRLAVASTLISLAVSIPAAFALVRYRFPGLNGLAVFWALPLSLPGVAIG

VGMLQLLSIFFRLPPFLGLLAVHVAIVIPFCISLLVASVLQLDRAQEEAAASLGANGFQRFFRIILPGLA

PGMAAASIMAFLTSFGEVTVTSFLTTARMTTLPVRIYADSTFMLEPTVHAVSAMTMLLTLIALFVLNKFL

RLDRLYAR*

>gkv_195|gene_NONE|hypothetical protein

MKQLAPFFARKRTRAPGLPRRAHNFHFFVDRGDESILPCVKPV*

>gkv_197|gene_NONE|putative thiosulfate sulfurtransferase (Rhodanese-likeprotein)

MFLRKLIAVGAFALMAGVANANEGPLVTTEWLEENLGSADIAVIEVSVNPGVYERGHIPGAVNFAWHTDL

VDPVRRDIATREDLQARLQAAGVSDDTTIILYGDTNNWFAAWGAWVFDVYGLEDVKLLDGGRVAWEAEGR

PLDSAVPAPAAGTVTLAEANNDLRAFLPEVVAASDSGSHAIVDIRSANEYSGTIIAPEGFQETAIRAGHV

TGAVNVPWSSAVAEDGRFKSPDELRAIYAAAGVDGSKPVITYCRIGERSSHTWFALSRILGYDVQNYDGS

WTEYGNSVGVPVTNPAGTVWTGL*

>gkv_198|gene_NONE|conserved hypothetical protein

MVTASFAARRNLWVAAGLALLIVVFLVLIPARDSLNRPLPLSMLLGVAFGALLQRSRFCFWCNYNDFLTD

RDPRGLLSILTALAGGTIAYAAVLHGWVPDPFAGRLPPDAFIGPIGLPLVLGATCFGLGMGLTGSCISAH

LYRLGEGSVASIPVLVFVLVGFVLGFLAWNPLYLLFGSAGPVIWLPAIFGHGGALALALAGFAALAALLI

RFGRFPAPEAPAQPVQPVQAIFQHRWPGVTAGVIVALIAIIAYFRVAPLGVTAEVGSIARTYAAGQGWLP

QRLIGLDTLRGCIAVVKETLASRNGVFVLGLVLGAAVAAQIAGQFKPVLPRAVSLPRLAVGGMLLGFGAM

IALGCTVGVLLSGTMSGALSGWVFGLFCLLGGWAGGRLRRAIP*

>gkv_199|gene_NONE|hypothetical protein

VPKTVGVLLAAGLSRRFGPENKLTFAWQGQPLMSYAADALVGAGCESLAAVVSDPDVAALLPAGFTAIAL

PTGLPMASSFVAAVDHAVALGGQRLLICLGDMPGISGARLRELLAMPGSAACTCEGARMPPMVLEAADFT

RARASAKGDRGARVFLQSLSPQQLLPLEAFEAVDIDLRPDSN*

>gkv_200|gene_NONE|xdhC and CoxI family protein

MKQGMAPNFHCEWPAAPPLTTATAAMPTPLSAHPALADPWEAALAFGEGTVIALLTKTEGAAYRNIGTAM

AIAPDGRYAGAITSGCIEADLVLRAEAQRLSNAPTLLRYGEGSPFFDLRLPCGGAAEITLFALRDLPVIA

DLSRARAQRIATGLLLTSDGRLSLAPSGQTGFNADGFLTHFQPPLRFVVFGAGPEAIVFSNLVASMGYGQ

LLLSHADNTLDVARTGGLNVRELGRMADIAALGIDHHTAALLFYHDHDYEPEILQALLQTPAFYIGAQGS

RNTQRTRLGRLHEAGVPEDLLARIHGPIGLIPSTRDPQALAVSVMAEVMQYDSQFSA*

>gkv_201|gene_NONE|membrane-bound aldehyde dehydrogenase [pyrroloquinoline-quinone] (ALDH)

MADTKFQSGGKRRFGIGKAETMELTRRGFLISAGLAGAAFGFPRSGLAAMDPATADGLPVQAAGARFDPT

IWYWIDEAGKVNVHIKQAEMGQHVGTAIARILADELGANWADVSIEHVDTDAKWGFMMTGGSWSVSQSWP

LYRQAGAAGRVALADKAAELWGVDASTVTITNGVATSGDNTATFGELVAAGITRTFTADELTALPLRPLS

ELTLVGKDVEALDIATKVNGQAIYGIDAKIDGMVYGAPLMPPTRYGAEITAIDDTEAKAVRGYQQTLKLD

DPSGIAPGFAVVIADTMWAAKKAAKLVKVTWTPGFGADLGEDDFQAENARLIADPASGGVLDTGNSDVDP

VFASAANVFEQTYSTATVLHFQMEPLNALAFRNDDGIWEVHTGNQAQSLTVPWLQAALGVGDGQVIMRSY

MLGGGFGRRLSGDYAIPAALASQQLDGRPVKLVFSREDDVAFDGPRAPSMSQLRMAFDADNKVVAMDSAY

AAGWPTKANMPAGLATGTNGEPYDPFAVDGGDHWYETGAQRLRAISNDLAVQTFRPGWLRSVGPGWTNFS

LESFMDEAAHHIGADPLQFRLDHLTAEGRNAGGNSPLDVGGATRQANVLRRVAEISNYANASLPEGSALG

LATTFGQSRGMPTWVAAVVQIAVDKEWGEIKVEKIWMVVDCGVVVDPDGARAQLEGGALWGVSMALYEGT

GFENGMVRDRNLASYTPLRLIDTPPEVHIELVESTEAPVGLGEPGVTVIAPAIANALFNATGVRMRHLPM

TADDVVAAIEAEA*

>gkv_202|gene_NONE|isoquinoline 1-oxidoreductase subunit alpha

MFELSINGQKVSVDVDDDTPLLWVVRDELGMTGTKFGCGIGMCGACTVHIDGVARRSCVTTVSQAVGVEI

TTIEGLSADASHPVQEAWRNLRVPQCGYCQSGQIMQAASLLAGNPNPSDSEIDSAMTGNLCRCMTYVRIR

QAVREAVTAINEGAVQNG*

>gkv_203|gene_otsB|trehalose-phosphatase|

LGADIAEPASTVPTWNLDGSVAGITAPEEQTAQMKSALPQTPDGWAFFLDLDGTLLDLASTPDAVQPAPG

LFDALRRLEDETGGALAIVTGRAIEFVDALFTGHHFTVAGLHGAALRVPGDDIQPAERASAAYSSARDFA

QAQAKMLPGVLFEDKSQAFALHYRLAPLSAVAVADVMKRALVLAGPAFMLRPGKSVVELCPAGHDKGSAL

RYLMTRPPFYGRKPLAAGDDLTDEAMFPAASALGGLGVRVGPLIDLPRSGASLGLPTPASFRDWIRRLTR

*

>gkv_204|gene_otsA|alpha,alpha-trehalose-phosphate synthase [UDP-forming]|

MSRLIVVSNRVPNPDRPAAGGLAVAVQAALRDRGGIWMGWSGNSCGERDPGPLRIREEGNITYALTDLSD

RDIAEYYQGFANSVLWPLCHYRIDLTDFARRDAAGYFRVNRQFAERLAPMIRPDDVIWIHDYHLIPLAAE

LRQMGIENQIGFFLHIPWPAPDVYLTLPVSERLLQSMTAFDLLGFQTAADAENFGLCLKRSRVAKPVAGQ

PGLFETPDRQFTVDAFPIGIDVAHFTRTARNAARNPTMRRFKDSLGDQQVIVGVDRLDYTKGIPQRLAGY

RRFLENNPIWAGKVGYLQITPTSREGVAEYDALQREVAELAGRIAGQLGRLDWTPVRYVNRAFGQHILAG

IYRMARVGLVTPLRDGMNLVAKEFIAAQDPADPGVLVLSRFAGAAYELEGGALLVNPYDEEGMANAIATA

VSMSLERRQELHAYALAQIEAHDIFGWCDAFLTRLAPAIPELVADS*

>gkv_206|gene_metH|methionine synthase|

MTALPKSASFARIVDAARQRILVLDGAMGTQIQLLKMGEDEYLGHGSAGCQCHIHSDHPQKGNNDLLNLT

QPEAIEEIHFRYAMAGADIVETNTFSSTTIAQADYALEDQVHALNVQGARLARSGVDRATAIDGRMRFVA

GAVGPTNRTASISPDVNDPGFRAVSFDDLRIAYAQQIRGLIEGGVDLILIETIFDTLNAKAAIFAAEEVF

IEIGERLPVMISGTITDLSGRTLSGQTPTAFWHSVRHAGPFTIGLNCALGANAMRAHLAEISAIADTFVC

VYPNAGLPNAMGDYDETPAFTAQQIEGFARDGLVNIVGGCCGTSPEHIRAMAEAVAKYRPRAIPEHAPLM

RLSGLEPFILTPEIPFVNVGERTNVTGSAKFRKMITAGDFASALQVARDQVENGAQIIDINMDEGLIDSQ

AAMVKFLNLVASEPDIARVPVMIDSSKWDVIEAGLKCVQGKAIVNSISMKEGEAAFLHHARLCRAYGAAV

VVMAFDETGQADTEDRKVEICSRAYKLLTEEVGFPPEDIIFDPNVFAVATGIEEHNNYGVDFINATRRIM

EACPHVHISGGISNLSFSFRGNEPVREAMHAVFLYHAIQVGMDMGIVNAGQLAVYDQIDPELREACEDVV

LNRRDDATERLLDLAERYRGQGGAEKKERDLAWRDWDVAKRLEHALVNGITEFIEGDTEEARLAAQRPLH

VIEGPLMDGMNVVGDLFGAGKMFLPQVVKSARVMKQAVAVLLPYLEEEKAAGGGVGRQSAGKILMATVKG

DVHDIGKNIVGVVLACNNYDIIDLGVMVSSEKILAAAREHDVDAIGLSGLITPSLDEMVHVAAEMERQGF

DIPLLIGGATTSRVHTAVKIAPAYQRGQVVYSVDASRAVGVAQNLLGSRSLAYQAEVRAEYEKVAEGYLR

GEREKQRLPLADARANPVKIDWAAYQAKVPSFLGTKVYDDWDLADLAQYIDWTPFFQSWELKGVYPRILQ

DEKYGETARSLFADAQAMLQQIIDEKWFDPRAVVGFWPANAVGDDIVLFADETRSHILATMHTLRQQLPR

RDGRPNIAMSDFVAPMGQAEYIGGFVVTAGFKELEIAARFEAANDDYNAIMVKALADRFAEAFAERMHQH

VRRELWAYAADEVLPNDALIREEYAGIRPAPGYPAQPDHTEKLTLFRLLDAEAATGVKLTESMAMWPGST

VSGLYIAHPESYYFGVAKVEEDQVADYAARKGMDKAEAERWLAPILNYIPKA*

>gkv_205|gene_NONE|ATPase MipZ family protein

MAHIIVVGNEKGGAGKSTVSMHVATALARMGLRIGVMDLDLRQKSLGRYIENRLAFMAAENIDLPTPVYV

ELPEVDPMTVDPNDNVLDHRFSAAVSALEPQSDFILIDCPGSHTRLAQVAHSLADTLITPLNDSFIDFDL

LARIDSDGEKILGPSVYSEMVWSARQLRAQAGLVPLDWVVLRNRIGAQNMNNKQKMESAVDRLAKRIGFR

TSPGFSERVIFRELFPRGLTLLDLRDVGGGSLNISNLAARQELRELIKSLNLPGVTPDF*

>gkv_207|gene_NONE|putative ATP-dependent RNA helicase rhlE

MDFDMLGLSPRLTKALAELGITEPTPIQAQAIPHAMNGRDVLGLAQTGTGKTAAFGLPMIDALIKDSRRA

QAKGARALVLAPTRELAKQIAENLAAYTKDSHLKTVVVTGGAGIGGQIQRMERGTAILVATPGRLIDLLD

RKAIDLSQTEFLVLDEADQMLDLGFIHALRRIAPLLPANRQTMLFSATMPKQMEELAASFLSNPIRVQVN

PPGQAATKITQSVHFVASRAKTDLLIELLDAHRDELALVFGRTKHGMEKLAKQLENAGYAVAAIHGNKSQ

GQRDRALRDFRAGTLRVLVATDVAARGLDIPDVRYVYNYELPNVPDNYVHRIGRTARAGKDGQAVAFCAP

DEMGDLRDIQKVMKTTIPVASGAPWEVPADGGGAKKGPAGRRPFKGNGGGKPKGDFGRPQGQGQRRGGRP

QAKSAA*

>gkv_208|gene_NONE|ybaK / prolyl-tRNA synthetases associated domain protein

MASTRGTLALSKAGISFSLHPYDYDPKAEHTGLAAAEALGLDPAIMLKTLMIEVDGKPACVAIPAGNSLS

MKRAAAAFGAKQAAMMSVPKAEKLSGYHVGGIGPFGQMRPIRTVFEESAMSGPVIYINAGQRGLIMGIAP

DDAARFLQAQIAPLVA*

>gkv_209|gene_NONE|uncharacterised BCR, YnfA/UPF0060 family protein

MQVLGLYLAAAAAEILGCFAFWMWARLDRSVLWLAPGVVSLAVFAYLLTHAPADTAGRSFAVYGGVYIVA

SLLWMWIAEQTRPDQWDVLGGVICLIGAAIILWAPRAA*

>gkv_210|gene_rpmE|ribosomal protein L31

MKEGIHPDYHFIDVKMTDGTVVQMRSTWGKDGDTMSLDIDPLSHPAWIGGGTRLMDTGGRVSKFKNKYAG

LGF*

>gkv_211|gene_rplS|ribosomal protein L19

MDLIAQLEAEQIAALGKTIPDFKAGDTVRVGFKVTEGSRSRVQNYEGVVISRKNGSGIAGSFTVRKISFG

EGVERVFPLHSTNIESITVVRRGKVRRAKLYYLRDRRGKSARIVEQTNYKAKAEA*

>gkv_212|gene_trmD|tRNA (guanine-N1)-methyltransferase|

LSEPPELAGIWTAQVITLLPQAFPGILGESLIGRALQEGLWQLQTYNLRDFGEGRHLNVDDTPAGGGAGM

VIRPDVMARALNVARGRMRGDAPIIYLTPRGRPMTQGLMRELAAGDGVTLVCGRFEGLDQRAIEQFGMIE

VSLGDFVMTGGEIAAQALIDATVRLIPRVLGNQESIEEESFSDGLLEHHQYTKPALWQGHDIPEVLLSGH

HAKIEAWRRAEAERLTKERRPDLWRAWCAQHGKDPDEDRELSGA*

>gkv_213|gene_rimM|16S rRNA processing protein RimM

MSDRIVLGQIAGSFGVRGDLRLKSFCAIPEDIAIYTPLYTDDGRVFRVVVITGQTNGALVARIEGISSKE

EADALRGQNLSADRDRLPNLPDDEFYHSDLIDLEVLDTGGAILGRVKTVLNHGAGDILEVQLTGKPATAL

LPFTLAIVPTVDLKAGRIIADPPEGLFE*

>gkv_214|gene_rpsP|ribosomal protein S16

MAMKIRLARGGSKKRPHYAVVAADARMPRDGRFIEKLGTYNPLLAKDNEERVQLNAERIQYWLGQGAQPT

DRVARFLEAAGLVAKTERNNPKKAVPGKASTERAAKKAARDAAPAEEAAAE*

>gkv_215|gene_NONE|chorismate mutase

MSQDVVTRAAALLAGHRASIDRLDSILVYTLAERFKHTQSVGLLKAEHALPPADPTREQQQIERLEKLAR

DADLDPEFAKKFLAFIIQEVIKHHETHQS*

>gkv_216|gene_NONE|acetyltransferase (GNAT) family protein

MIPAPTLHTARLTLRRPDARDWVAFRDFMLSPRGATFGITTEGRAFRQFAAELGHWDIYGHGMWTVTLSG

SDAAIGLVGPWTPPDWPETEIGWMIYPASAEGKGYAFEAARAAVDHAYRILRWDTVVSYVAPDNTRSAAL

AIKLGAVLDASAATPDSCKGYQVFRHPRPDALDKGEAA*

>gkv_217|gene_NONE|acetyltransferase (GNAT) family protein

MNLADDIMLTTDRLVLRKPQAQDWPAMRDFYLTDRSYWVGGPKEPEASWRGWASDIGHWEMRGFGGWVAV

RKDTGAAIGRFGPYYPIEWPEKEIGWSLWDANLEGQGYAFEAAKATLAHAFTILGWTTAVSYVHANNAPS

ARLAERLGAVLDGDAARPQRDFEVLVYRHNPAKVLA*

>gkv_218|gene_ffh|signal recognition particle protein

MFENLSERLSGVFDRLTKQGALSEADVESALREVRTALLEADVSLPVVREFVKAVSKKATGQAVTKSITP

GQQVVKIVHDELIAMLAGDTDPGVLKIDSPPAPILMVGLQGSGKTTTTAKLARRLKDREGKRVLLASLDT

NRPAAMEQLAILGTQIGVDTLPIVKGEDPIAIAKRAKTQASLGGYDVYILDTAGRLHIDAELIAQAAAVR

DVVSPRETLLVVDGLTGQDAVNVATEFDAKIGVTGVVLTRMDGDGRGGAALSMRAITGKPIRFIGLGEKT

DALEVFDANRIAGRILGMGDIVALVEKAQATFEAEQAERMVKRFQKGLFNMNDLRGQLEQMLKMGGMEGL

MSMMPGMGKMSKQMSDAGMDDRMLRRQIALINAMTKKERANPDLLQASRKRRIAAGAGLDVSELNKLLKQ

HRQMADMMKKMGKMGKGGMLKQAMSMMGKGGIDPSKMGEADMAAAQQQLAKGLGQGFGGQLPGLGGPRAL

PPGLSGLMKKK*

>gkv_220|gene_NONE|bacterial regulatory helix-turn-helix protein, lysR family protein

MDDWDEIRTAYQVARFGTVSGAAEALGVHHATVIRHIDALEGRLGAKLFQRHARGYTATEAGQDLLRVAQ

VADDQFNQMITRIQSHKEELAGELVITSLQGMTELLLPVLMAFQAEHPDIILRHLSGERLFRLEYGEAHI

AIRAGSIPEDPDHVIQPLAQHPMALVAAESYIARYGLPASEADFAGHRFVGHDAEDYRAPYSRWLATVVP

RKQVVYRTLDLQEMYAAVIAGAGIGFVPRWLLGVNPELREVVPLRDDWISPLWVVTHVDLHRSAKVQGFL

RMLKGAARDWTC*

>gkv_219|gene_NONE|bacterial regulatory protein, arsR family protein

MQHRLDLVFSALADPTRRAILAMLLEDDMAVTDVAAPFSMSLAAISKHLQILAEARLISQEKRGRVKWCK

LEPDALREASIWIQGFGLFEGFDLDAFEAFLTQELPPEAQAEA*

>gkv_221|gene_NONE|ATP synthase F0, subunit I

MSDPDQNDGEKKRLEALEHRLREVRKRDAPQVQPGRDFSQADLAWRMVIELVAGIGIGATIGYGLDVVFG

TKPFLMIIFLFLGLIAGVRTMIRSANEVQMKQQAKAASEEKGEESGREH*

>gkv_222|gene_atpB|ATP synthase F0, A subunit|

VAESTETGFVFHPLDQFQVKSLFGGDIGTLTLTNASLWIMLAVVCVWLLMVVGARKRELIPGRMQSVAEM

TYGFIHKMVEDVAGHDGVKYFPLVMTLFLFVLFANALALIPMSFSPTSHIAVTVVLALIVFVFVTGLGFV

KHGAGFLGLFWIKSAPLAIRPVLAVIEVISYFVRPVSHSIRLAGNLLAGHAVIKVFAAFAASLLIAPVSI

IAISGMFAFEVMVAVIQAYVFTILTCVYLKDALHPAH*

>gkv_223|gene_NONE|ATP synthase C chain (Lipid-binding protein)

MELAQLGQYIGAGLACIGMAGAAIGVGNVAGNYLAGALRNPSAAGGQTAMLFIGMAFAEALGIFSFLVAL

LLLFAV*

>gkv_224|gene_NONE|ATP synthase B/B' CF(0) family protein

MDNPAVEAAMPQLNTAYYGNLIFWTLLGLVAIYFILSRIALPRIGSVLAERAGTVGNDLSAAEELNQKAR

SAEAAYQQALSDARVEAGRIVEQTRATIDTELKAELAKADAQISVKVAESEKVLGEIRDQAVASITSVAK

ETVGDVVALFGVAAEDDALSAAVDAKMKG*

>gkv_225|gene_NONE|ATP synthase B chain (Subunit I)

MRKVLSIAALLVGASVAPALAADGPFFSLRNTDFVVLLAFLLFIGVLIWAKVPALIVRVLDARAETIRAQ

LAEARALRDEAAALLASYEQKQKEVQEQAARIVEVARREAEAAAEKARADIETSVARRLSAAEDQIASAH

KAAIKDVRDRAASVAIAAARDVIAGQMDATKGNKLIDDAIKTVDAQLH*

>gkv_226|gene_NONE|bacterial regulatory proteins, gntR family protein

MPFEKIQAEKLSQSVARQIELLILRGILRPGERLPAERELAERLGVSRPSLREALAELQDRGLLISRANA

GVFVAEDLGAAFSPALAKLFASHDEAVFDYLAFRCDLEGMAAERAATHASDMDLRVINTLYLKMEAAHLK

RNPSEEAELDADFHLSIVEASHNVIMLHMMRAMFQLLRVGVFYNRQAMFKQRPIRQQLLEQHRAINDALQ

TRDPEGARKAVIDHLAFVERSLTHQRKADRNDAVARQRFQHEVTR*

>gkv_227|gene_NONE|inner membrane lipoprotein yiaD

MTFSKISLGVAAASMLALAACQAPIQGQTNDNTRQGAMVGAGLGAVVGALTGDDSNDRWRNAAIGAAVGG

GLGAVGGQALDRQEAELRQQLGGNVGIVNNGQNLTVTMPQDVLFGTNSTAVSIQSQTDLRTVAASLNRYP

NTSISVIGHTDSTGSASYNQDLSVRRAQAVASVLINGGVAPARVYTVGRGASQPIASNATPDGRQLNRRV

KIIITPTN*

>gkv_228|gene_NONE|methylated-DNA-[protein]-cysteine S-methyltransferase family protein

METHEPYHYGLIARAIAALDAGGADMSLDDLAAKLGLSPAHLQRVFTRWAGISPKKYQQFLRANLARDLL

ASRHTTLDTAAEAGLSGTGRLHDLILQWEGMTPGAYAAKGAGVTIRHGLFDTPFGPAIVMATDQGICGIG

FTSHQDPALTYRDLASRWPNADFQQDGRLDSQVQAAFSGGPARLHMIGAPFQIKVWEALLAIPEGQVTTY

GDIARRIGNPAAVRAVGTAVGRNPIAYLIPCHRVLRRDGGMGGYHWGLDSKRHLLTFEAARTEPPVISRV

IG*

>gkv_229|gene_nth|endonuclease III|

MREVFTRFRAASPTPEGELHYTNAYTLVVAVALSAQATDVGVNRATRALFEVADTPQKMLDLGEERLIEH

IKTIGLFRNKAKNVMRLSQILVDEFGGEVPSSRAALESLPGVGRKTANVVLNIWWHFPAQAVDTHIFRIG

NRSGICPGKDVVAVERAIEDNVPAEFQQHAHHWLILHGRYICLARKPRCGDCLIADLCLFEEKTV*

>gkv_230|gene_NONE|pyridoxal-phosphate dependent enzyme family protein

LINAAWDRLQGHVRRTPILSSPALDAIAGRRILVKAECLQVTGSFKARGGWAAVSALTGAQGVIAYSSGN

HAQGVARAATAHGLPSVIIMPADAPKAKIDGTRALGGEVVLYDRETEDRDAIGAEIAAARGLVLIKPFDD

AQVIAGQASVGIEIAEELGDLPADVLVPCGGGGLASGIALALSARAPQMRVRTAEPEGFDDAARSLLAGH

PVKNASLGGSICDAILTPSPGALTFPILQSYAQAGLVVTEDQVLRAMAMALKHLKIVVEPGGAVGLAAAL

FQPQAFDTDTVIAVASGGNVDPAILARAVALM*

>gkv_231|gene_NONE|enoyl-[acyl-carrier-protein] reductase [NADH] (NADH-dependent enoyl-ACP reductase)

MTLDLNGKRGLIMGVANDHSIAWGIAKAMHEAGAELAFTYQGEAFGKRVAPLAASVGSDFLVDVDVTNDE

SLDKAFDLLETRWGKIDFLVHAIAYSNKDELTGRFINTSRENFKTSMVISVYSLIEVARRARPLMSDGGT

ILTLTYQGSNRVTPNYNVMGVAKAGLESAVRYLANDLGPEGIRVNAISPGPMKTLAGSAIGGARATLRHT

EQNSPMRANATLDAIGGTAVWLASAAGRCTTGEVVRVDGGYHVLGMPQQENL*

>gkv_232|gene_NONE|beta-ketoacyl synthase, C-terminal domain protein

MRRVVITGLGIASSIGNNAEEVAAALKAGKSGITANAQMQEHGFRSQIAGDVKLNIADHIEKRTLRFMGP

GAAYAWIAMSQAIADAGLTESEISHERTGLIAGSGGPSTSNLFAAHQTVLTTGSPKRIGPLMVPRGMSST

VSANLSTAFKIKGINYSITSACSTSLHCIGAAAEQIQFGKQDVMFAGGGEELDWTLSCLFDAMGAMSSKY

NDTPERASRAFDAGRDGFVISAGGAIVVLEELEHALARGAKIYAEVTGFAATSDGHDMVAPSGEGGERAM

RLALNTLPEGRKVSYINAHGTSTPVGDVGEVEAVRRVFGDTHPVISSTKSMTGHSQGATGAQEAIYCLLM

LRDDFIAPSINVETLDPAINPGEIALTTVLDAGLDTVMTNSFGFGGTNGSMLLSRFVK*

>gkv_233|gene_NONE|fabA-like domain protein

MLMMDRITDISGDGGLHGKGHVVAEFDITPDLWFFACHFPGNPVMPGCLGLDGLWQLTGFNLGWRGWQGQ

GFALGVGEVNLKGMVRPDRKMIRYFVDFTRVIDRKLKMGVADGRVEADGELIYTVKDMKVGLAAPAA*

>gkv_234|gene_NONE|response regulator

MGEAQTERGITWLCGAGLRPTRQRVALATHLVGDGLDRHVTAESLFDAVTASGEQVSLATVYNTLRAFCE

AGLLREITVDGSKGYFDTRLDDHPHFYWEDSATLSDAPADQLEIRRLPQAPAGVEVAAVDVIIRLRRK*

>gkv_236|gene_NONE|glyoxalase/bleomycin resistance protein/dioxygenase

VFFHDPSGQAGAVIGNAGPKTNARDPKDAIMARIDHLKTEARALRAQNPHLTHSAALESVAQSHGYRDWN

TASADAQRPLELGAPVAGHYLGHAFTGRLLDVQTLDASGRQRLTVQFDTPIDVVESQHFSAYRQRATATV

EANGVSPARLGNGTPQMQIWRDSRRM*

>gkv_237|gene_kdsA|3-deoxy-8-phosphooctulonate synthase|

MQDVAIRGLSVGNNQPLTLIAGPCQLESADHAQMIAGTLAEICQRTGAQFIFKGSYDKANRTSLSGTRGL

GMEAGLKILEGVRAAFGCPVLTDIHEREQCAEVASVVDVLQIPAFLCRQTDLLIAAGETGAAINIKKGQF

LAPWDMANVASKVASTGNNRIMLTERGVSFGYNTLVTDMRSLPTMARTGYPVVMDATHSVQQPGGQGNSS

GGQREFAPVMARTAVSLGIAAVFIETHQDPDNAPSDGPNMIYLDQMDRLMTSLMAFDALAKADPLRP*

>gkv_238|gene_NONE|capsule polysaccharide export protein-like

VTGSTDPNVIAQEGLSARQLRMARRVAQRHGITFTTDFDAVAQLRARGVDPFGGNSLLDIIPDDGQGQNG

TANSPLTENLPVKRAKTEPQLRPAAPVGRPTVAGTNATLIDAAADRSKEILALQRDIARRRRRKLALLIA

RLSFFVFLPTLLAGWYFYVIATPMYATESEFSIDQPASAMMGSGTSTASMLSASGSVGIIQDSVTVQSYL

TSRTAMIRLDEDLGYRAHFNQPNIDPLQRLPDDAGYEDAYKLFERNVKVGFDPTEGILRMEVIAADPATS

EAFAKALVGYAEEQIDGMTLRLREAQMADAEQSYRDAELRRADALSRWLAIQQEMQQIDPTSELQVRMQQ

IASLETERQQLQISLRSLEVTSRPVESQLQGLRSRIATIDGLVAELRTSLAASDGGSSQAVRNTELRIAE

ENYNFQVEMVGQSLAQMEAARLEASRQVRYIRMGVEPVAPDRPTYPRAFENTLVALLVFAGIYLMLSITA

SVLREQVSS*

>gkv_239|gene_NONE|ABC transporter family protein

MLEFHDVSKSFWTGTQRKVILNHASFRVELGQSLGILAPNGTGKSTLINMIAGLEKPDEGKIIRNCRISF

PLGFMGGVVNKMSGKENARYIANIYGLDDDYVEAFSRWLCGLGEYFDMPLGTYSSGMRARFTLSLMLALD

FDMYLIDEGMPSTTDVEFNRKAGAILRERLEKSTVIIVSHLPNVLEKFAERAAVLRDGTLHMFDTLEEAK

QLYDYTA*

>gkv_240|gene_lpdA|dihydrolipoyl dehydrogenase|

MSSFDLIVIGAGPGGYVCAIRAAQLGLKTAVVEGRETLGGTCLNVGCIPSKALLHATHSLHEAQHNFDKM

GLEGAAPTVNWDKMQAYKGDVVGQNTGGIEFLFKKNKVTWIKGWASIPAAGQVKVGDEVHTAKHIVIASG

SEPASLPGVEIDEEVIVSSTGALALKQIPKRLAVIGAGVIGLELGSVYARLGAEVTVIEYQDKITPGMDA

DVQRTLQRILAKQGLNFVLGAAVQGATTAEGGATLNYKLNKTGDEHSLTVDTVLVATGRKPFTAGLGLDT

LGVALSPRGQIETDSHYATNISGIYAIGDAIAGPMLAHKAEDEGIAIAEILAGQAGHVNYGIIPGVIYTT

PEVASVGATEEALKAEGRAYKVGKFSFMGNARAKAVFQGEGFVKLIADKETDRILGVHLIGPAAGDMVHE

ICVAMEFGASSEDVARTCHAHPTFSEAVREAALACGSGAIHA*

>gkv_241|gene_NONE|inner membrane protein

MTAELIVLVLVALLQVVQIMLAGAAMNQAGLMQWNAGPRDTAPNFPPLVGRLIRAVNNTTEGLVLFAIAV

IVTVITGQSSGLTATLAWIYLAARVLYVPAYAFGWSPWRSAIWAVGLLSTVIMLIAALF*

>gkv_242|gene_sucB|dihydrolipoyllysine-residue succinyltransferase, E2 component of oxoglutarate dehydrogenase (succinyl-transferring) complex|

MSTEVRVPTLGESVTEATVATWFKKPGDAVAVDEMLCELETDKVTVEVPSPAAGTLAEIIAAEGETVGVG

ALLAQITAGAAAPAPAPAAAPIAPPKAEVKPVPEATARKGDDAPSARKLMAENNIEASAVAGSGRDGRVM

KGDVLAALVSAPAAPAPAAAPAAPRAASRADDAPREERVKMTRLRQTIARRLKEAQNTAAMLTTYNEVDM

TAIMDLRNQYKDLFEKKHGVKLGFMSFFAKACAHALAEVPEVNAEIDGDSVIYKRYVHMGVAVGTPNGLV

VPVVRDTDTKSFAQIEKEIAGFGRKARDGKLSIEDMQGGTFTISNGGVYGSLMSSPILNPPQSGILGMHK

IQDRPIALNGQVVIRPMMYLALSYDHRIVDGQGAVTFLVRVKEALEDPRRLLMDL*

>gkv_243|gene_sucA|oxoglutarate dehydrogenase (succinyl-transferring), E1 component|

MNDQSSSQAFRAADMLNGANADYIAHLQASYASDPQSVDESWRALFAALDDNGAAQAEVSGPSWARRDWP

PMAAGDAISALDGQWPGDPIPADQVKTTTQKLQAKAAEVGSKVSEGQLRTAVLDSLRALMLIRAYRIRGH

LAADLDPLGMQAKPYYPELDPASYGFTGADMERPIFIDNVLGLEVATMTQIVDLVRRTYCGTFAMQYMHI

SDPAQSAWLKERIEGYGKEITFTREGRRAILNKLVEAEGFEKFLHVKYMGTKRFGLDGGEALIPAMEQII

KRGGAMGVQQIVMGMPHRGRLSVLANVLSKPYRAIFNEFQGGSFKPEDVDGSGDVKYHLGASSDREFDGN

KVHLSLTANPSHLEAVNPVVLGKVRAKQAQLRDSERTKVLPILLHGDAAFAGQGVVAECFGLSGLVGHKT

GGTIHIVVNNQIGFTTAPSYSRSSPYPTDIALMVEAPIFHVNGDDPEAVVHAAKVATEFRQLFHKDVVID

IFCYRRFGHNEGDEPMFTNPAMYKTIKGHKTTLTLYTDRLVADGLIPEGEIEEMKARFQSHLNDEFEAAK

TFKPNKADWLDGRWAHLGRPDQDNTPRVATAIAAETMAEIGKSLTTAPDGFGLHKTVDRLLEAKRNMFET

GEGFDWSTAEALAFGGLLTEGYPVRLSGQDSTRGTFSQRHSAFIDQQSETRYYPLNNIREGQSHYEVIDS

MLSEYAVLGFEYGYSLAEPNALTLWEAQFGDFANGAQIMFDQFISSGESKWLRMSGLVMLLPHGFEGQGP

EHSSARLERFLQMCGGDNWIVANCTTPANYFHILRRQIHRDFRKPLVLMTPKSLLRHRLATSKASDFTEG

SSFHRVLWDDAQLGSSATELKPDDQIKRVVVCSGKVYYDLLEERDARGIDDIYLMRLEQFYPFPTMTLVR

ELGRFKDAEMIWCQEEPRNQGGWSFVEPNFEWVLNRIEAKHRRLTYVGRAASASPATGLASQHKAQQNAL

VDEALTIKGN*

>gkv_244|gene_sucD|succinyl-CoA ligase [ADP-forming] subunit alpha (Succinyl-CoA synthetase subunit alpha) (SCS-alpha)|

MAILVNAQTRVICQGITGAQGTFHSQQALDYGTQLAGGVTPGKGGTTHLGLPVFNTVHEAIAKTGANATA

IYVPPPFAADSILEAIDAEVPLIVAITEGIPVLDMMRVKRALVGSKSRLIGPNCPGVLTPNACKIGIMPG

SIFSAGSVGVVSRSGTLTYEAVKQTTDVGLGQSTAVGIGGDPIKGSEHIDILEMFLADPATESIIMIGEI

GGTAEEEAAQFLKDEKARGRWKPTAGFIAGRTAPPGRRMGHAGAVISGGTGDAESKIDAMRSAGIVVADS

PATLGQAVMQAIRG*

>gkv_245|gene_sucC|succinyl-CoA synthetase beta chain (SCS-beta)|

MNIHEYQAKGLLRDYQIPVGAGQIILNSRDADQAAAALQGPLWVVKAQIHAGGRGKGHFIEEAAGAGGGV

RLARSASEAAQIARQMLDNTLVTVQTGPKGRKVGRVYLEAGADIARELYLALLIDRKTSRISFVCSTEGG

VDIEHVAATTPEKVITIAVDPATGFQQWHGRAIAFALELSGAQIKECVALTGNLYRAFTERDMEMLEINP

LIVTPAGHLHCLDAKVGFDNNALYRHPEIAALRDEAEEDEKELAASKFDLNYITLDGSIGCMVNGAGLAM

ATMDIIKLYGSAPANFLDVGGGASKEKVTEAFKIITSDPNVQGILVNIFGGIMRCDIIADGIIAAVREVG

LQVPLVVRLEGTNVEIGKDIIRNSGLNVIPADDLADAAQKIVAAVKDATHKAAEA*

>gkv_246|gene_mdh|malate dehydrogenase, NAD-dependent|

MARPKIALIGAGQIGGTLAHLAAIKELGDVILFDISEGTPQGKALDIAQSGPSEGFDAILKGTNDYADIA

GADVCIVTAGVPRKPGMSRDDLLGINLKVMKSVGEGIAKHAPDAFVICITNPLDAMVWALQQFSGLPAHK

VVGMAGVLDSARFRHFLSVEFGVSMKDVTAFVLGGHGDTMVPLARYSTVGGIPLPDLVEMGWTSQETLDG

IIQRTRDGGAEIVGLLKTGSAFYAPATSAIEMAESYLKDQKRVLPCAAYVEGAFGLDGLYVGVPTVIGAG

GIEKVIGIKLSADEQAMFDKSVDAVKGLVAACKTIDPSLV*

>gkv_247|gene_NONE|conserved hypothetical protein

MKAYRLLTADDTSAFCHKVTEALSKGWELYGNPTYAFDAANGVMRCGQAVTKDVDATYDPEIKLGQL*

>gkv_248|gene_sdhC|succinate dehydrogenase, cytochrome b556 subunit

MADMQKGGRPLSPFMLGKYYRIQLTSASSLLTRISGNALIAGAVLLVWWLLGLAMGPDWFGPVHWVVTSW

IGRLIIVGSTWALWYHLLAGARHLIFDQGYGLKIQTAERLGWAAVIGSFVLTVLTVGLFWLI*

>gkv_249|gene_NONE|succinate dehydrogenase, hydrophobic membrane anchor protein

MRYLTDRKRAVGNGASRTGTFDHIFMTSTSYGLLVLVPCLVWVLGNALFLPFDQARAYVGRPFPLIVLGL

TLVVGLRHFAVGAQMMLEDYLQGFAREITVLIARGLSWLLIATGLLALVRLALGQPS*

>gkv_250|gene_sdhA|succinate dehydrogenase, flavoprotein subunit|

VAAYTYETHEYDVVVVGAGGAGLRATLGMAEQGLRTACISKVFPTRSHTVAAQGGIAASLGNMGPDSWQW

HMYDTVKGSDWLGDTDAMEYLAREAPKAVYELEHYGVPFSRTEEGKIYQRPFGGHTTEYGEGPPVQRTCA

AADRTGHAILHTLYGQSLKQQAEFYIEYFALDLIMSEDGVCTGVLAWKLDDGTFHVFSAKMVVLATGGYG

RAYFSATSAHTCTGDGGGMVARAGLPLQDMEFVQFHPTGIYGSGCLITEGARGEGGYLTNAAGERFMERY

APTYKDLASRDVVSRCMTIEIREGRGVGPNKDHIYLHLNHLPPETLHERLPGISESARIFAGVDLTKEPI

PVLPTVHYNMGGIPTNYWGEVLNPTADNPDRVQPGLMAVGEAGCASVHGANRLGSNSLIDLVVFGRAAAI

RAGQIVQAGTPNAAPNLHSIAKAFDRFDALRYANGGTTTADLRLEMQRAMQADAAVFRTDKTLAEGVEKM

TAVAAKVADLKVSDRSLVWNSDLMETLELTNLMPNALATIVAAEARKESRGAHAHEDYPERDDVNWRKHS

LAWVEGNTVSLDYRPVHLDPLTTVEEGGIDLKKIAPKARVY*

>gkv_251|gene_NONE|putative lipoprotein

LKYIVLFTAFAGLAGCIGQDTVTDASRVLAKEAVNNAVAARAPGINVAPVTDCIIDNANGSELLVLATGA

VTGTVSAETSQLVRDIATRRGTQNCLLSNMSNAGLLSVMQGALS*

>gkv_252|gene_NONE|putative lipoprotein

MRAPALALLGFVTLTACGAAPSGNVDIVAATAYCEQRARAAMGPTGGVTVGFNNRSGPSAEVELGVNTDF

LRGRDPMQVYDECVRRRTGFAPYRPPAL*

>gkv_253|gene_NONE|hypothetical protein

MKKFVFAAVLALIAAPAMAQDTSAARALILPMLQEISPGAAGEILADCVLQAATADELATLSAATGPSRE

VGTLITSIVSRPAALNCVNAAG*

>gkv_254|gene_NONE|succinate dehydrogenase iron-sulfur subunit

MVQLTLPKNSRMTVGKTWPKPAGAKNVRKVQIYRWSPDDGANPRLDTYFVDLDQCGPMVLDVLIKIKNEI

DPTLTFRRSCREGICGSCAMNIDGQNTLACIYGLDEIRGDIRIYPLPHMPVIKDLVPDLTHFYAQHASIM

PWLETKTNTPQKEWRQSIEDRKKLDGLYECVMCASCSTACPSYWWNSDRYLGPAALLHAYRWIIDSRDEA

TGERLDELEDPFKLYRCHTIMNCAKTCPKGLNPAEAISHIKKMMVERVA*

>gkv_255|gene_NONE|nitroreductase family protein

MKKTAVTASPILPVLAERWSPRAFNSAALPLSRIASALEAGRWAPSASNRQPWRIYAASKSDSPEGFAKL

LSFLVPFNATWAAEASILIIGAAQIENDEGKPQPGALYDLGLYMGNFATQIAADGLYLHQMTGIDVDAAT

TGLDMPAGWQAVFAGAIGEFGDIAALPEKLAAREVEPRSRKPLSEIFFPQ*

>gkv_256|gene_NONE|hypothetical protein

MRRILGILFAVAAITGGADRLSAQTQEEFDFVQELLGELQHISFQRGAEYCGFVGIDHTGKLVASEATRG

TMASCPLHVPPRSEMTIIASYHTHGAFDEGFINEIPSDIDMRSDQSMGIRGWVSTPGGRLWLVDSRKMVT

RQVCAQGCLPIDPNYYKAQAGDVAKTYTYDELVERLQDW*

>gkv_257|gene_NONE|hypothetical protein

LPQIHKTLEYRRARFNVAGQNLEQLTRQAWGQFATHVDRAVNGSNHSTIAGMRGRDGGADGFYVHGARFN

DGQGVGTIPMVPAAEVDLGERQPNAGENFVNSDFLALIRGNHVICLNCGRNGGALRSYLSALFKKAGLPV

DAQQFELVRVENPKNLAVIEAVGVKSIDLKVSIAEATADEIIDSPAAGGTWKNAVKKIGEAFLGLTEKDA

ELRQLREAEQGSVTVSINVDKRDATSAPHGLDHFASEFVEDEEAEGYIIHLRNNTKITPHEITVRKPVKL

EAHANSVSVIQAWDALREFMGELAENGQLEA*

>gkv_258|gene_NONE|putative membrane protein

VSGVVGYYFQPMVANNTNAVNTVVTIFSILAGFLIAVITLIAEPTLKQAKSWQELQLMKHTVQRKLFRQK

LLFFLYLITLGVALGTFLVPDAQAELRRWLEMVFLGLATFVFLASFDLPGSLMRIQMERYEAELDATKPQ

VLKDAAQAAVDAVKRADAPK*

>gkv_259|gene_NONE|hypothetical protein

MPPENSTDKTSVEERLLASIKLMDASQGCALLRIETDDPEGAVCGLASSDGIIAVVQNGKTMLPQFQFDM

ANGRIFDVVGAILNLRPACVSNLRLCYWLNRGHVDFGCAPTQRFGHEDAAIVAAFRRYIEPVRHG*

>gkv_260|gene_NONE|phosphoesterase/phosphohydrolase-like

MHWFTADPHYSHDRIIGFCDRPFPDVAAMSAHLLAECRERVGPDDDLWILGDFIAGRSTDAQRREVRTIY

HALPGRKHLIRGNHDQDWVCDLPWDSVAETADIVVDKRRLFLCHYPMITWPGARHQGLQLFGHVHQNWRG

SRNSVNVGVDVWDFRPVKLQEIERRAARLPVNAHWDQVEPGRAWPKALCAGCGRILDPALVSGHAVVRQG

RIVMTATNETIVLMGEAMRKWLPEGRRVCPECIGGYLSVSEVTLPAGFSFDETRNRAVPKGK*

>gkv_261|gene_NONE|ATP-dependent Lon protease domain protein

MKLSPIDREWFDKVLRAIAAAEAGPSEADLAHAPLLSDWKAAISPGGHVMLWGEVSDHPLLGNASIHTSQ

LIAIDPEAGWARTASRWYRLGRSIDALAAELADSMNGKAKLAGSVQFTLPGFANIDDPELLQKLLATYIA

RVRGIDAADRAASGEED*

>gkv_262|gene_NONE|ATPase family associated with various cellular activities (AAA) family protein

MSTIPFIEARFFNPAEGRIELETRLMRHLRKLRCKRIPPDLPAPPDEADAPGEISDADLMGIIRFSDDDE

ARIKRRVRRILELRKAASGLEHLKSDDRARLEVLKDGARLISIPSEHHADEFAAMLHAEMPWMAPATEIV

WHAMRRSVREGLPGLKIPPLLLDGPPGIGKSELARRLGELLATPTTVIEATGENASFGVVGSQRGWGGSH

PGRLIETVLQSRIANPVMVVDEVEKAGRAVSTKGQAFGLAEGLLPLLEPLTAKRWSCPYYQVKFDMSWVI

WVLTSNDFQLLPEPLLSRCPPIRLRHLTLPELQAFVRREGAKRALSETSVETICEILSHPTLQRDRLSLR

VAARMLERAADLEQGPTLH*

>gkv_263|gene_NONE|calcineurin-like phosphoesterase family protein

MTILITADLHLDLWVRAGRDPFTGVLPVLRDLDALIIAGDLANNPKRNWPRALSRITRLVSPARIWVIPG

NHDYYGAMLDDNVLARIAVETGANLAQKQVLTFGNYRLLCCTLWTDFALTGDPEAAMARAGMVMPDYGRI

RRLDGDLITPEDTVAIHRDHLEWLTREMAKPWPGQTVIVTHHAPGTAVSGPISGISPAFASDLDGWIEAH

RPDYWFFGHTHRPLSARIRGAPVINVSVGYPDEVPEGGEAELLLRGLIFPGA*

>gkv_264|gene_NONE|hypothetical protein

MLSGHSRNQISVPPPIIMLAKDGLVPTGHRAPDPDLTTGARGQ*

>gkv_265|gene_NONE|IS66 Orf2 like family protein

VISPAGSFRIFLASEPVDFRKGMDGLVAHVANHFELDPFDAAIYVFRSRRADRLKLLAWDGTGLVLTMKR

LNGGRFTWPKPQTGPGVLSKVQFDALFEGIDWRAVTAASVRKPSFL*

>gkv_266|gene_NONE|his Kinase A (phosphoacceptor) domain protein

MPEHFWAARMDWPHSEPEEVERMTEKRQGLQDTLALVGVAVIVAVTFVGDVISPVIYNVWLAYFLALVLS

FSTRYIWLPIVVVGIVAPLMWMGIVLTDSTSIPYVTVLNRSLAVGVMLVLAGVGALTVRARRMVNAHDWE

QRTQMHLARRLLGDLTRQEIGQRSVNLLALALGAKAGVAYELRGNRLEFLAGFGLAADRPPVHGQGHLWR

AVHDDQVIHLKVAGEHALGWGTGLFAGKSAQSLIIPLHEGGRVNGILEFGLDVMRRNLSTEFLEQAARKI

GIELRSASYREEMEALLEETRRQAETLHSHTEELAASNEELEEQSRALIENEARMREQQSELEEQNAQLE

QQTSQLEEQRDALAASRQQLADQAGDLARESRYKSEFVANMSHELRTPLNALLIMARLLSENRPHNLSPE

QIQWAETIESSGRDLLGLINDILDLSRIEAGKVDVDPEVTQPKDVAARMMRAFAVQASESGLKLVSEVAD

DVPEITTDVGKLEQILRNFLSNALKFTQKGSVTLSVARSGAETVTFSVKDTGIGIAAEQHDAVFEAFRQA

DGSISRRFGGTGLGLSISRDLAGILGGDVMLESTPGKGSTFILTLPLHLTAPVPEADINPRPARSDQAMP

VIDGPLGGISGVSDDRGASLDHPEEHTLLIVEDDAAFAQIMRDLARELGFHAIVVGTADDAVRAAQRHRL

NGIVLDVGLPDHSGLTVLDRLKRDPKTRHIPVHIVSAQDRLREALAQGAVSYLHKPVAREALAKVLEGIS

KQLDRRPRQLLIVEDDPTQLSGLKALLGSDDVITRGAATTAEAIAACRETVFDCIVLDMTLPDGSGFDLL

EKLSADENAAFPPVIVYTARALTEAEEQHLRRYSRSIIIKGAKSPERLIDEVTLFLHQVVSDLPQRQREM

LAASLNRDAQLEGRRILVVEDDVRNIYALTGVFEPHGVHVQIARNGREALEALGRSADSASPKIDLVLMD

VMMPEMDGLTATREIRRIPQWKNLPIIMLTAKAMADDQAQCLAAGANDYLSKPLDVDKLLSLTRVWMPR*

>gkv_267|gene_NONE|cheR methyltransferase, all-alpha domain protein

MKARADAEADAADIELDLFLEALNRRYHYDFRSYSRASLTRRANVARERLDCVTLSELQGRLLRDPDVLH

EVIDAMTVQVSDLFRDPAYYRALREQVIPHLRTFPSLKVWVAGCANGEELYSLAILFEEEGLLDRTMFYA

TEINRRALGRASAGIYDIGRVPGFTANYQNAGGRGSLSDYYTAAYGSAAFNRRLRQRTLFSEHDLATDEV

FSEAHLISCRNVLIYFDNTLQDRVIGLFAQSLVRGGFLGLGAHETLRFSSHSDAFQPFNENERIWRRAAY

PQSTVPETSDA*

>gkv_268|gene_NONE|sensory box protein

MPNRNIRFLLVDDIRDNMVALEALLRRDGLEVDMAGSAPEALEMMLLRDYSLAFVDVHMPDINGYELAEL

MRGTERTREIPIIFVTAAERNETRRFRGYEAGGVDYIFKPIDPVMLKSKAEVFYRIARQAKELEAQRDEL

RGIARDRDLAIASLRAHANNSPLALVECDADLSVRGWSEGAERLFGIKQDDVIGRTLNCAGCFDQSALDM

LHNWVASLDHHARHTAEILATSPGGPLNCEIYGSVLTDPALGRTSLSLQILDVTERHRAEEVRSLLVGEL

NHRIKNTLANVQAIMRQTLRTSGDLKEFGGRFSGRLQALARAHSILSNVTWSSASMDEVIDDQIKAGTLD

NDSLHREGPKIALSPENTLRLALVLHELGTNATKYGALSTPDGHVTLKWQIEGEDLVLEWRESGGPPVSQ

PQGKGFGSSLIATGFGDSRASVDWRPEGVVWTIKVARGFERMHSPRAIAVPHQQPAPTRDAAIAGLRVLV

VEDEPLVAMDISLGLEEGGATIVAVARSLDEAVRSAQLADVDVALLDGNLAGDPVDQVARILHDRGVPFC

FVSGYGREHLPTGYPDAPILAKPASPDMLLSTLRKITSKKVLANAVSE*

>gkv_269|gene_NONE|protein hipA

LDVYMNARLVGQFARQSGGAHLFTYAQSWLDWPGRLPISRALPLQGARHQGAAVIAVFDNLLPDHPQIRD

RLAARVGAGGADAFSLLAEIGRDCVGALQFLPAGEVPDAQGLQADPLSAAQIGALLSNLSAAPLGLDRTR

DFRISVAGAQEKTALLRQGDQWFAPRGATPTTHILKPAIGLLPSGVDLRNAPQNEHFCLRLLAGFGLRVA

DTQVADFNGLSTLVVTRFDRLWTADGRLIRLPQEDCCQALGVPSSLKYQSDGGPGIREISALLQASDQPV

QDRLDFFKANILFWLMGATDGHAKNFSLSMAPGGRFRLAPLYDVISLQPALAAGQMQPGDMRLAMRMGAS

RYYRIGDITGRHILESGLDAGLSRVQVLAICAEIAESAAAAFEAASDTVQDQEMIAAIRAGFDQRLPRLM

TMT*

>gkv_270|gene_NONE|helix-turn-helix family protein

MDHIIRSPKDIGHVLRQARKAGGLSQADLAAKAGLWQRTVSTLETGAADARLETLFDLLAALDLELQVVP

RSRTDPDSFADLF*

>gkv_271|gene_NONE|bacterial extracellular solute-binding proteins, family 5 Middle family protein

MVSYVKAVAGASAIASLLPALAAYAQPAPTTGGTYYHALEAAVTCIDPPTQIFHVALNVGRQLVDSLVDQ

DPQTGAIVPWIAERWDISEDAQTFTFHLRQGSHFADGAPIDAAAVQANIDRIAALGPRAVGAAPLLIGYT

GTEVIDPQTVRITFDRPAVQFLQALSGAWFGLISPNDLGKSADALCAGDFAGSGPFITASYQGDTEIVLQ

RRDGYDSHSSLAGHTGDAYVDALHLVVIPEAGNRAGAVQTGEVHSASNIAFQHVPLLEQLGLTLLSPHIP

GMTESLIFNRETAIGQDDAVRQAIQHAINSEELIRTVWGPSWTPRTAVLSESTPGWADFSDLLTFDEGRA

NAILDEAGWLRGEDGFRSRDGQRLSVRIIMSSPASDQLVKQQLAAVGIDYNIERLDSATSTARVQAGEYD

IYKWQMTRADPAILNAVWNSNRTSQGVARSPASELDDLLLVQESAIDTAARAAAAADVQRYLIENALVVP

LVDRAWTYAIHPSGHGLRLDGETKLVFFDVFTDR*

>gkv_272|gene_NONE|binding-protein-dependent transport system inner membrane component family protein

MAILKFWAARLGQSAFVLWAAFTFSFVILYLLPSDPVSLLLGQDGAAGAPDAQTVAALNAEFGLDQGWIA

QYFQRLGQFLTLDFGNSIQHRRPVTDLVAEVLPATLHLSLLALGLALVLGVALAVAASMTRMGWLRQVLQ

SIPPTILALPGFWIGLVLLQVFAFQLRWVPSLPNRGALSMILPVVTLALPTAAIIGQILTKSLLGTWRQP

FVQVARARGLSQWDILHRHVARNAILPALTMAAIIFGNLLTGTIITETVFSRGGLGRLIERAVTQQDIPL

VQFMVVFSAFVFVVINLLVDAAYPALDPRIRRPRAV*

>gkv_273|gene_NONE|glutathione transport system permease protein gsiD

MSDLSMPRARNACLPGFTQNIGIILSLALVALIALAAVVPSLFTHYDPLYADTAAALQGPSAAHWFGTDQ

FGRDLFARVIYGAGPSLAATALAVAIALVVGSLIGLISGYLGGWADAVIMRLVDVLLAIPGLLLSMAVVA

ALGFGATKVAIAVGIASVAVFARLMRAEVVRVAASRYIEAARLGGASRLTILFRHILPNAAGTVLTLAAL

EFGGAILAISALSFLGYGAQPPQPEWGSLISGGRDFIAVAPWLTLSPGLVIISTVLAANRISRALDPDQS

LVK*

>gkv_274|gene_NONE|ABC transporter family protein

MNDLALDLNGLTLSYRTKTGESRTILNDINLQIGAGEAVSLVGESGSGKTSLGMAVLGLLAPNASATYRS

LTIGGEKITSWRDPLWRQILGNQLALVPQDPNVSLNPVRKIGAQMVENLILHRLATPPEARARAADLLDR

VGIDRPLQRLNQFPHELSGGQQQRVLIAMAFSSNPKLIVADEPTSGLDVTVQRIVLDQLDKLRQEFNTAV

LLITHDIGVAADRTDRSIVMQLGKMVEQGPVRQVLTRPSHAYTQALIAAAPALHGARRAPSAGLSPAAPG

AALVQAQALTKRFGSHPVLHGVSLNIARHSTVAIVGESGSGKSTLARAITGLERIDSGILTFDGVPVPQQ

GHRALRQHFRRVQMVYQNPYSALDPRFTVAQSIAEPLANFDRPTRATLHRRTSEALDAVALPQSYLTRLP

SELSGGERQRVAIARGLILRPDLLVLDEPVSALDVSVQGQILQLLVDLQAEFQLTYLFISHDLAVVRQIS

DEVHVLNQGRIVSSGPTAQVFDASTDPYTRRLLAAIPGQSLSGQSLDLERI*

>gkv_275|gene_NONE|ketopantoate reductase PanE/ApbA C terminal family protein

LTHRYVIIGGGAIGMQLAGRLTLAGLNALLITRGAQYQALSTAPLHLHHPQGTDQVQIAVANSPEAARLT

RNDILVVATKTQDVEQVVTQWAWQPLEGDTSGAPGALAADLPIVILQNGTAAEDIARRRFSQVISVASIV

PVSYLEPGHVTSRAAPKNGYFQLGRLTGASLPTDDTLAQIAGDFEAAGFVTRIRDNITARKYEKLLFNAT

NAVDVFEGTADERRALTAALTAETRAILSAAGVNLDLGAPDVDIAVELGTNRVPAGNTNSAAQRSTWQSF

ARGKPSEVDYLNGDIVLLARQHGLDAPLNARVQRLLGLAQTLGEAPQTRHIRDVLLKD*

>gkv_276|gene_NONE|luciferase-like monooxygenase family protein

MMTTTIPRLRLGFLTNGAFSEHDAGGAAQGHRDAVDLFRLGEQLGYDAGWIRNRHFDNYVSSPLTVLAAA

SAVTQHITLGTAIIPMGYEHPIRLAEDVSTLDLLSGGRVELGIAGGIPSFDAIFNGTDGPGWAKATPKKV

VDFLDAIRGTSYGTTPQGDDLHLRPLSPGLADRIWYGAGTPDSAQRAAGLGLDLVLSAIAPSLGLPFDAA

QKAVVDAHRAAWTRTDRAPRFTTARTFFPALNDRQRALYQGYGDLRQSQGPAASRPKGALTPAEPAGRAN

STGGLMSPEIVGSPAEVIDYLLADVAVREAGELLIFLPPGFSHAENVELMENIATHVAPALGWRPAR*

>gkv_277|gene_NONE|3-mercaptopyruvate sulfurtransferase (Rhodanese-likeprotein) (MST)

MTDPLISPEKLAISLHDVVLLDATYFMPSDPVAARAAYDALRLPGARYFEIDKVADHSSDLPHMMPDAVT

FGAAMAALGIDGSKPVVVYDRSVNHFSAPRVWFTLTAYGVPDVRVLDGGLLYWQKLGLPLEEGAPTNTAT

VPVKDWVLDTRRVLSGTEMAEVVAAGTRPIIDARGAPRFQGTAPEPRAGLTSGHMPGATNVPFDQLTDTT

TGQFASVDQLETLFAEKSGTDTVLTCGSGMTAATLTLGLARIGQTGTIYDGSWTQWGRGTLGPIVTGA*

>gkv_278|gene_NONE|bacterial regulatory proteins, gntR family protein

MSKAPDKLEPSILKRGAGVSYVYESLRNEIIELKLAPGTPIDEVQLAERFALSRTPIREALVRLAAEGLI

TTLTNRATIVSQIDFLNLPDFFDALTLMYRVTTRLAAANHRPEDIPAIRALQDEFVAAVEAKNVMALILT

NRDFHLAIARAGGNQYYIDLFTRLLDEGRRILRLYYSSFNDNLPRQYVNEHEDMIAAIIARDVTRADELA

RAHADQIVRQIQSYISADNRQNAQMGL*

>gkv_279|gene_NONE|FAD dependent oxidoreductase family protein

MRIGVIGAGIIGATSALALARAGHEVVIFDRKGVAAETSRGNAGAFAFADIIPLATPGILRKAPKWLLDP

LGPLSLPPAYALRIAPWLLRFWRASWRDRYATAVAAQVALMQVSQAALADLLRDYHLQDLIRTDGQLQLY

EGEAAFAAAMPGLRVRQQHGIAVDLLTAPAAIAAIQPGLVPRFTHAAFTPAWSNVTDPAIWTEAVLIEAQ

RLRAKLKIAAVTAITPSDAGVTLATPTGPDRFDRIVLATGAFSNPLLQDMNLRLPLDTERGYNTTLPAGA

FDLRTHVTFADHGFVVTRIGDGVRVGGAVELGGMDLPPNMARADALLAKAKRFMPDLVTSGGTQWMGFRP

SMPDSLPVIGAHPDDPRIVLAFGHGHLGLTQSAGTARLVADLIAGAPPQIDLTPYRPTRFFGYRA*

>gkv_280|gene_NONE|proline racemase family protein

MTNHTFQCLDGHTCGNPVRLVASGGPQLQGANMIEKRAHFLREFDWVRTGLMFEPRGHDIMSGSILYPPT

RPDCDVAVLFIETSGCLPMCGHGTIGTITMAIENGLITPRTPGRLSIEAPAGRVDIEYRQDGRFVEEVRL

TNVPSFLHAEGLTAEVEGLGEIVVDVAYGGNFYAIVEPQKNFRDMADYTASDLVTLSPRLRRALNAKYEF

IHPETPAINGLSHIQWTGAPTVDGAHARNAVFYGDKAIDRSPCGTGTSARMAQLAAKGKLSVGDAFWHES

IIGSIFKGRVEAAAEVAGQPAIIPSIGGWARQTGFNTIFIDDRDPYAHGFVLI*

>gkv_281|gene_NONE|dihydrodipicolinate synthetase family protein

MSETIFTGCIPALMTPCKPDRSPDFDALVRKGQQLIAAGMSAVVYCGSMGDWPLLSDAERMEGVARLAGA

GVPVIVGTGAVNTKSAVAIAAHAASVGAKGLMVIPRVLSRGPIVAAQKAHFQAVLAAAPDLPAVIYNSLY

YGFATRADLFFALRAEHPNLIGFKEFGGGADLTYAAQNITSGDAGLTLMVGVDTQVCHGFINCGAKGAIT

GIGNALPREVLHLVALSQAAAAGDAEARRKAYELEQALLVLSSFDEGPDLVLYYKYLLVLNGNAEYALHF

NESDALTPSQQAFVTTQYQLFKDWYANWAA*

>gkv_282|gene_NONE|hypothetical protein

MGADTPEMGLNGRALKAARNVFSFGGARFLNSICRQDK*

>gkv_283|gene_NONE|hypothetical protein

VLPDLIRKRPQPSRSSISAGTSKCPLTAVRLKVFIQAVVPRGIGKADLSDGKWRAL*

>gkv_284|gene_NONE|asp/Glu/Hydantoin racemase family protein

MGRILVVNPNSSVDVTSGIIAALAPFGDHFEVVGMDEGPATVATEEHVARAGLAFAELAARRPDAAAFVT

ACFSDPGLDLARARVPQPVFGIQEAGILSALAVADRFGIVALSPASVQRHLRKLRLMGVDRRLAGELALP

GVSAVASGHDPRVFDMLVDLVGQLKDRGAGAVVLGCAGMAPIRTRLEDRTGVAIIDPVIATGAMALGSLV

ARQAV*

>gkv_285|gene_hydA|dihydropyrimidinase|

MTQFDTVIHSGQIATADQVFAGDIGIKDGRIIAVAEKIAGGERRIDAGGRIVMPGGIEGHAHIAQESSAG

VMTADDYLSGSISAAYGGNSSFIPFAAQHRGQSIDHVLETYGARARRSVIDYSWHLIISDPTEAVLQDQL

PRAFQQGITSFKVFMTYDLMNLGDGGMLDILTVARAHGAITMVHAENNDMVKWMNRQLAARGLTAPRYHA

ISRPELAEEEAINRAIQLAKLADAALFIVHVSTAGGAAIVRREKFNGAKLFAETCPQYLALTRDDLDRPG

TEGAKFMCSPPLRGRETQDALWHHMAMGTFESVSSDHAPYRADETGKFTAGFDVAYPKIANGMPGIAMRL

PWLFSEGVVKGRITLEQFVALSATNAAKTFGCTTKGRIAPGMDADIAIWNPDVTRTVTLADQHDNMDYTP

FEGLQITGRPEHVLTRGASVIADGKLVGTEGQGQFFGRAPVDLRARPGVALPEFDPAQNFGTELR*

>gkv_286|gene_NONE|carbon-nitrogen hydrolase family protein

MTRMIIGGAQMGGIQKAETREQVVQRMLALMDQAHAAGVSYLAYPEMTLTTFFPRYYAESRADFDHWFET

EMPNPQVQPLFDRARAYGMGFSFGFCELTPEGQHFNTAIVVSPTGDIVLTYRKTHLPGHAEFEPERTHQH

LEKRYFLPGDTGFNVAQSQGVQMGLAICNDRRWPESWRVLGLQGVELVSIGYNTPSQNNLSRDEGPEKRL

YHHELSVCAGAYQNATYAIAVAKCGLEDGYHMIGGSMIVDPDGFVIARATGEGDELITAEADFDKCAFGR

STVFNFAQHRRIEHYTRIATQTGVEVPQ*

>gkv_287|gene_NONE|amino acid ABC transporter, atp-binding protein

MEQQPQVLRVRVLDKHLGDVTVLKSVDLTLKKDKQVCVIGASGAERAPMRPCCDRAEAPISGQIYIHGSR

IAHKTSRSCDPYSRRLSSNRAAKAEFGVPKPEQLKALRRCIAA*

>gkv_288|gene_NONE|transcriptional regulatory protein tctD

MRCLLVEDDEQLAEWLVKALRAQGILADWEERGLLAVNRVLSDDYDALLLDLGLPDIDGGEVLRRIRAAG

ASLPVIILTARDDLSERVNLLHIGADDFMMKPFAVAELEARLSALLRRRAGHAQGIFRCGSLTYHQHGQR

FELGGAPLHLSPREHALLRLLIQRAGDPLSKAQILARLVGDDKDLNPEAVEVMVYRLRRKLEGSDTAIQT

IRGLGYMLDAANG*

>gkv_289|gene_NONE|his Kinase A (phosphoacceptor) domain protein

MARGRSLRLALALWMLPLSLFVFLSSLWVTHSNIAALADSAYDRSLAGAVRAIEENISTTNGGLGVQLPY

TLLATLQAASTSVIYVRVSTDDKLVQIGDIGLPPPPAIGSDRPHFYTITYLGKELRGAALRVPLAQPLYG

ADAPQSLIVEVAETTESRALFLRRVSDVAFVRDLSISLIGLGLLVVGIGVSLRPLTRLKEMFDGRAPHDL

SPIPTASIPTEARPLIDSFNTLLQRHADQYAAQRRFLDDASHQLRTPISVLQLQVDYALSTPSPEAQREA

IAAMGPVIARATQMTRQMLTLARAENLSATHVQNWKQIDLTALLPEVLRLHLAPARAATVALDLDLPDGP

VMIAGDDTLIFEGLSNLLDNAIRHSPAGGTVAVSLCSGALAEITIRDHGRGAPPQILAHLGEAFLTIRSD

RSSAGTGLGLSLAQTVARAHGGDLVAENPADGGFQITMRLARMDS*

>gkv_290|gene_NONE|bordetella uptake gene (bug) product family protein

MNLDRAVTRFARIFCLSLATALTALPLALPAQDLPDRIDCLVPAQPQGGFDMTCQLLAETLPSAGLGITA

VTRSYLPGGVGAAAYSAVSHGADARADQLIAFADGSIYNLALGQMPATSDQAPQWVAVLARDYGAVVVRG

DASWQGLEALMREAAQDPHRIGFGGGGLLWGPDRMRLSLTARAYGIPSGNLRFLAFEGSGSCLEALSAGF

VQACLNDAAAAQTAIDQGTDLRILAVYAPTRLPGALSDTPTAREQGVPLDWPVARGVYLAPGVDPAITAA

WQARIGALMHTPAYAAALARLNLQPDPLTGDALRAAIAGIATTAQAHARALGLVCTPQPLWPQRQITCVR

TDPT*

>gkv_291|gene_NONE|iron(III) dicitrate-binding periplasmic protein

MRLTLTLACSMALLAPAAFADPITVNDGTHEITLPDTPTRVVALEFSFVDALASVAVSPVGVADDNDITR

LPQAIRDITGPWTSVGLRGQPSIEDIAALSPDLIIADLDRHAAAYDALSAIAPTLLLPSRGEDYADSLKS

ARLIGEAVGKTPQMDARLTQHAAIMQDYATQMQALVTPGTTVIFGAAREDSLSLHGPDSYDGSVLAALGL

TVPAIRDGGDAYEFASIEQLLALDPDYLLVGHYRRPSIIDQWSSAPLWNVLKAAQAEGHVVSVDSNVWAR

NRGIMAAERIADDALHILQGTFAEVE*

>gkv_292|gene_NONE|iron(III) dicitrate transport system permease protein fecC

MPRGLIMTTGTTLLGVVLFAWSLIAFSTFPLSITQALPLIGWGESSTMTQIIAQIRVPRALCAVLIGGMM

GLCGALMQGITRNRLASPALMGVTGGASLGLALVSSGVIALPIAPPLAAATGGAVAWALVMTLGASWSPD

AARVRLILAGMTMAALCAGLTRLVVLLAEERALGVLNWLAGSLANTGYADVRLLAVTALAAAVLAAFVAA

RLNIVALGDEAARALGISLVPLRLAIFAGGCLMVGVTVTVTGPIAFIGLIAPNVARLMVGADYRLVTPLS

ALLGANLLLASDIAARWVAFPSETPAGAITALIGAPFFLFLARRPL*

>gkv_293|gene_NONE|iron(III) dicitrate transport system permease protein fecD

MIRAFSLCALIGALFLLSLCLGAHRLSPLAVAQLLAAHDAQDFLVWNHRLPRALIALILGGGIGLAGALV

QGVIRNPLASPDLLGVTQGAGMALAGAILLFPGLAVNLLPLIAIAGGAFGAAVLMLYNAGHFSPLRFALS

GVALSAGFAGVTEFLLLTHPVAINTALMSLTGSLWARGWEQMPLVLLVLPIALLALPLAKSLDLIGLGDE

TAHALGVDLGRVQVRALGLAVVLTGTAVAVLGPVGFIGLVAPHIARLATPGRALVILPASAAFGGAIMMA

ADVLGRAMAPPLEVPVGVMTAVIGAPYFLWLLFRMR*

>gkv_294|gene_NONE|iron(III) dicitrate transport ATP-binding protein fecE

MTIAIELKDLSLRYGATPVLNGLNLHIPTGKFTVLVGPNGCGKSSLLKALIRALPLARGEITLDGKAQSQ

IPPRKLARLMSLLPQTLNAPDGVTVRQLVAYGRSPHTNLWNRLDGSDRAVVDSAMTRLNIAAFADRPVAE

LSGGQRQRAWLAMVLAQQTPVILLDEPTSYLDIAHQVEVLRLCRELADEGRTVVAVLHDLNQAFRYGDQV

IVLQNGQCMAAGAPQEVAREDLLHAAFDIRARMITDPEALTPMMILRK*

>gkv_295|gene_NONE|hypothetical protein

MNDRSRALPAGHHNARRMTRLSTGVTCIFTQDAGRPNAHILEARRDISGF*

>gkv_296|gene_NONE|helix-turn-helix family protein

VDDTKPLPTAMIPPAARRKAAHSIAARLRALRKEAGLTLSDLAMRSGLAASTLSKIENEQMSPTYDTILS

LAEGLGVDITHLVTGTQGKSVNGRKAVTRKGEGIVHRTQQYDYEMLCNDIANRQFVPLLAEVKARSLHTF

DGLLRHPGEEFIFVLEGRVELHTEFYAPSLLEVGDSGYFDSTMGHALINPDDRPARVLWVCSRVVGPLAQ

*

>gkv_297|gene_NONE|malate/L-lactate dehydrogenase family protein

MAQRLSFDELVIVLEDILQKAGTSPDVAAIIAQNCAACERDGSKSHGIFRMRGYVSTLASGWVDGAAQPV

VEDAAPSFLRVDAQNGFAQPALAAARPLLLDKLRENGAAVLAIRNSHHLSALWPDIEPFADEGYIALSVV

NSMAVTVPFGAKRAVFGTNPIAFAAPVAGDLPFVFDMATSAWSHGDVQIARAEGRLLPEGVGVDAGGAPT

RDPNLVLEGGALLPFGGHKGSAISMMIELLGAALTGGKFSTEVDWSRHPGAVTPHTGQFILLIDPARGGG

LPFDLRAAEFTQTMREAGLDHLPGVRRHKNRAKALQSGMTLDADAWANLDKLRQFGIAGLNG*

>gkv_298|gene_NONE|hydantoinase/oxoprolinase family protein

MATRIGVDIGGTFTDLVYFDEATGKTVEGKVPTVPSAPEEGVVAAITGHVPQEIIEKAEFFLHGTTVGLN

ALLERRGSKVGLITTQGFRDVLEIRRGDRAEMYNLFWKQTEPLVPRSLRLEVTGRMLGTGAEYIPLDEDT

VRAAVAQLIAAKVDAIAVSLINAYANPAHELRVAEIIAEAGFTGGVSLSHKISGEYREYERTSTTCIDAF

VRGRMANYLRRLDGKLRELGFKGTSLITRSGSGSMTFAEAEDRPFETIMSGPVGGAQGAAELAKILNIKA

LVTADVGGTSFDTALVIDGKPQVLFEGVIDNMPIQSPWVDVRSIGSGGGSIAHIDPGGLMRVGPRSAAAV

PGPACYGKGGVEPAMTDAAAWLGMLGPGDLASGITLDIGKAKAALDSVGQHIGQDAEHTAAGVMRISSAA

MANAMREISLDQGLDPRTMTLLPFGGAGPLMGTLLADELGMNQIIIPPLAGNFSAWGLLGADMVQSTART

RVMDFAAGAAGAINATLTDLFSALETRSAAHADEAVKSARLDLRYKGQEHTLSIEVATQGGALAEAEADI

LNRFVSEYARTFGGTMNQDVELVSIRANTTVPLPQRQLSYTPKRSDGADDRVMDVYSFERQTRLPFRIIP

RGRITGKITGPAIVTEDTTTTYVDADWTITNGSAGEIILERIA*

>gkv_299|gene_NONE|hydantoinase B/oxoprolinase family protein

MKKTYDPIIHNAGKFGRTTEAKADPVTTEVIRHALNSAANQMKRALIRTSFSPIIYEVLDFAVAIYDAEI

RMLSQAPSLPMFMGTLSFCIDEAVKAIGGPENLEPGDALIYNWPFGTGSHPQDMVIIMPVFYQDTTLIGY

TAIKGHWLDIAAKDPYCTDTTDVFQEGVIFPGIKIYKKGVLNDDIFRMIMANTRVPHMVRGDLDAQVTGC

RVGVKSLLEVVERFGLETYRAAVDDMFDHGERIVRSYFEKIPDGRYVGKGEMDNNGVTKDRIPFEVAIEV

KGSDVTIDFSGVPDEQAGPTNSPLPTTISVCRVAITMLAGYGEAPHEGHFRPIKAITRPGSMFHPLPPAP

SFIYGWPGLQAIEVIYNAISNAMPKAVPAQSGGCICSIVAWGQREETREPWADGTPLPTGQGAWDGGDGG

IMLHISESATRFTPAEVWEHRNPWIIEQLAMAQDSVGPGKWRGGPGLNLDIRTTEDTLVTTVFERTLNNP

WGLQGGGEARPNNCLATMPDGEVHAIPKATHFLLPKGAVLQMRTGGGGGYGDPADRPVEAVKRDIEEGYI

SESFARQHYPHAFG*

>gkv_301|gene_NONE|amidohydrolase family protein

MPLLNSTAANLAEVTGWRHDFHAHPEILYEVHRTAARVAELLTSFGIDEVATGLGRTGVVGIIHGNRPGK

MIALRAEMDALPMPEETGLPHASTIPGAMHACGHDGHSAMLLGAAKHLAETRDFAGSVALIFQPAEEGGK

GALAMAEDGLFDRWPISACYAMHNMPGLPLGTFASVAGGIMASADKFNMTLKATGGHAAWPHTTPDPIVC

AGQIIGAVQTIVSREANPLLASVISITQVHGGSAHNVIPAEVTLAGTLRALDPGLRQRNAARLEQIAKGI

AATMGVEAQVEVQFGSGVVMNDPRELALCMDVARDLMGPDMVDAHMVPLMGGDDFCFLAERRPSCYVFVG

NGESAGLHTTKYDFNDDLIPIGISYWINLVQRATAS*

>gkv_300|gene_NONE|high-affinity nickel-transport family protein

MRALAVSVSILAVIALSVALALGLPDVIARQAVGMQREAQDALAGALRALRSGQSGAVIGFLTLCFMHGF

LHAIGPGHGKAVIAAYGAASGAGVRWMAGLAALSSLGQALVAIALVYGAVWLLDGARDRIEELAAYIEPF

SFALVAGLGLMLVWRGLRRLRPAKVAHHHHHHDHDCSCGHSHAPDPQALAAAKDWREVAVLVLGVALRPC

TSALFLLILTWRLGLDALGIIGALVMGVGTMAVTGTAGIGAALMRRGVLLSLPEGGAALRPITAMIELLF

GFAIAVIAGSVLIRLL*

>gkv_302|gene_NONE|polyphosphate kinase domain protein

MLRCYLITLSLCAPLAAGAHPHEFIDTSLVLHFDAAGQIATVDVRWVWDDFTSMLMLADQGMDPDADGAL

TAAEADAMAARLSLWPDDFLGDLYFTADGQPVSLQSPQDVAIDYQDGRLIMSYWRALAVPLQPDAGVITV

QAYDPTYYAFYDLAGPPELQGRADCDVAVAQADLNAAQQLYDQLLSELTEEDIADFGMYPEVGGAFADMV

NVTCAPLQ*

>gkv_303|gene_NONE|bacterial transcriptional regulator family protein

MSALDNGIAILDCFSFEEHALSQARIGQITGLPKASLSRAMKTLRESGILAYDESTRLYRPSQRLFQLGQ

IYRIHLSFLDTVQKLLSRACETCGHTGYITVFDGYHMSVLWVARGSSPLAIASTPAARAWAFATSNGRAM

LALMPGDEWLKRVPNPLPFVTPTAPQDMADLAQRIADVRATGRSMSVNNSYEGVSSQAVAVRDTDSREVI

GIVISYPTNLATEALKAQIGALLDDLRADLARAVE*

>gkv_304|gene_NONE|hydantoinase B/oxoprolinase family protein

MQPKTTSFRAGIDIGGTFTDFILFDGAESAIRLHKCLTTPDDPSRGLLGGLAELVEAAGIAVADLEEIVH

GTTLVTNAVIERKGAPIGMITTTGFRDVLEIGTEQRYDIYDLHLTFPDPLVARAHRLEVPGRLGADGGEI

TPLDEAAVITAVDRLAEARVEAIAVCLMNAYANPAHERRVRDLIAARHPDISVSISSEVVAEISEYQRFV

TACANAYVQPLMDRYLRKVQAALDSLGFTGALRLMHSAGGLVSLDEARAFPIRLLESGPAGGALATAWFG

KAAGEENVIAFDMGGTTAKACMIENGMAHIASYLEAGRVRRFKNGSGLPIKSPVVDMIEIGAGGGSIAAI

DAVGLLQVGPHSAGAAPGPACYGQGGDKPTVTDASVALGYYDPNFFLGGRMTLDMDAANAALNSIAAPLN

LSAVEAAWGIHQVVGENMASAARIHLVEKGKDPRAYAMIGFGGAGPAFAARVARILGVGEVIIPQASGAA

SAFGFLTAPLSFDLVKSAPTALAADFDTTAITSAFAALETDGRSKLAAAGVADSDVIIARTADMRLIGQV

HEINVPIPAGTFGPATYAEILASFTAVYTARYTKIPEGAELEVLSYRVRASGPTPSLTIEQAGQQSSGKD

ALKGHRDCYFGEGFVKTAIYDRYLLTPGAEIAGPAIVEERESTAIIPPGDRFSVDAAGNLRIKIASAQTG

MVIATPDMSLAEVKARIAADPISLEIMWSRLVTVAEEMWHTVIRTAYSLIISESQDFGCSILDAKGEILA

HSSRVMPAFNFTLPIVVKALLERFPVDTLKPGDVLVTNDPWLCAGHLFDIAVATPIFHNGKVVAIVGSVG

HVGDIGGTLNGMKAEQVYEEGLQIPPMKLVRAGVENEDLFTIMAENIRDAQQVLGDVRSLVAANETGGRR

LVSLLQEYGLPDFEALAETVQSLSEKAMRAAITAIPDGVYAGKSTYNPLGTKMDIPITITVAGDTIEADY

AGAPPEVPLGGINCTFSYARAQVVYPLKCMLTPNVRGNAGCLRPITVKAPKGSVMNCNKPAPVSLRTRTG

WFVSPAIFRALAGAAPDRVVAHTGLPSLLTVHGKTPEGEVFYDHLLSGGGQGGSAGKDGKSSILWPTSAA

TSSIELLESRSPIVILEKSFVTDSGGPGRHRGGMASRIRITKRHDDGKQLTVFVSPEGVDIPVAGLYDGQ

SGMGTHGYKRNSSTGDVVQDIGTGGLMTLDSPAHLVELQMGGGSGYGDPMTRPIADVEADLRDERITPET

AATVYGLGRR*

>gkv_305|gene_NONE|bacterial extracellular solute-binding proteins, family 5 Middle family protein

MTLTKRQLLKSLLGGVALTVLPLSAMAQARKIMVVASTVDIPNFDPHVATGYAPQWLFRNVYDPLVRVTG

TPPVATPGLAASWEMSEDGMTYTFHLDPTARFTDGSAVTAADVVYSFQRLLRLGTGPAWMVAGILDGDSV

VATDDATVTMNLLKPFAPLLSVLPWMFIVNAAVVEANLGTDDAQAYLLQNIAGSGAFTINRALPGELYEL

LRVENDWHQGGNIDAVFWRIVREAATTRMLLQRGEVHFALDLFTEDMEALEGVPGVVRIMEPDYRSFSIK

MNTAHGPLMDKNLRKAISYAFNYQSMLDAAQPAALMVGPLPPGMFGANPDLEVYRQDMDKAREYLAASDY

ADGGFTLSIHYAAGYEQQRRWCLILLEALATLNIQLDIRPMVWPDVVAMARSPETVTDFFSIFQSSNYAD

PDNTAYAAYHSSRNGQWQNPTYSNPEVDRLIEEARAATTPEARAELYGQFQEVVVEDAPDIFGVLELRKF

AMRDNVQGYEFCPVGATSPEIWPLSLA*

>gkv_306|gene_NONE|putative D,D-dipeptide transport system permease protein ddpB

MIQMILRRLLLLIPTLIGLTLLLFVISRLLPGDPVGLAAGPNASAELIARMRAEFGFDDPLWLRYWHYLT

GLLSGDWGTSVFTRRPVFQDVLTYLPATLELVFSALLIAVVIGIPLGLLTAVYRNGPLDYFIRTLALGGV

AMPRFFLGLLLQLAFVAWLGWLPLSGRFPFLEIPPQSVTGFYTIDALLAGDIRAFGIAVSHLILPATAMA

LSPLATIMRMMRASTIEVLGQDYILNARALGLSQRLIIGKYVLKNAMSATLTVIGLYVSWLLGGTVLVET

VFDWPGLGLFATQAILTQDFMPVIGVTLVIAIIYLLTMLVVDILYGVLNPKVRL*

>gkv_307|gene_NONE|putative D,D-dipeptide transport system permease protein ddpC

MTDAVTSSPRSARAESLSRALYRFSQSWLSVIGLAIVVLLIIVAILAPWIVPHPEHALGVTNTAARFQPP

SATAWFGTNAIGQDIFSLVVIGSRVSLFSGIAVVIISIIVGTFLGAIAGYCGGWIDEVIMRLTDLMLTIP

SLILAMAVAAALGSGTFNMIVAIALTWWPSYARLVRGEVISRKEDQFVTAARALGAGWQRILFRHILPNI

TSPIVVKASLDMGFAILTVASLGFVGIGVKPPTPEWGTLLSGARSYMPTYWWTAVAPGMAIFFAVFAFNL

LGDGLRDVLDPKARR*

>gkv_308|gene_NONE|oligopeptide transport ATP-binding protein oppD. domain protein

MSLLHINELRLAMQSYRKETEVLHGISLTVERGQIWGLVGETGSGKSLTGLSTMRLVPSPPGRYLGGQIL

FEGEDLLQADERRMRALRGRRMGMIFQDPTTNLNPVFRIGTQMVDAALHAAGEDPSLLGLAPGAGRRDMR

RAARETAIQMLAKVGIPNPESRIDDYAHQFSGGMRQRVLIAMAMIGKPDLLIADEPTTALDVSIQAQILR

LIHDLVLERNIGCLLITHNLGVVAQVCTHVAVMFRGRVLESGPVAQVLKQPEHAYTRALMGAIPTADTPR

GGLRGLAGVDF*

>gkv_309|gene_oppF|oppF

MTLEITHVTKQFGSFTALKDVSLSVRKGSSLGLVGESGSGKSTLARCILRLDTLTAGQITYAGTDIHAIS

AAEMRALRARLQIVFQDPYASLNQRMSVHDIIAEPLIIHKARFPMTASQRTARVLELLVQVGLGPEHLYR

FPHEFSGGQRQRIGIARALACEPEFLILDEPTSALDVSVQADILNLLQGLQDRLGLTYLFISHDLAVVRY

MCDEVAVIYHGEIVEHGPASLVLDAPQHDYTRMLLAAMPDPDPDKSPFLHRI*

>gkv_310|gene_NONE|helix-turn-helix family protein

MAVAPTSVGDALRARRIALGITQRVVADQAGLTTGFISQVERGLTAPSLSSLHAISKVLGLSPLQFIDTP

PAPQQLTRAGARQTYGIAPNVARYERLTGHFPGSVLRSVICHEDPGQRHAPIRHEGEELFYIIAGALTVE

VGGTPHVLHPGDTIHFSSGDVHSTWNHTSSPTTFLHTCTMDVFDDAQAVETTKEISA*

>gkv_311|gene_NONE|hydantoinase/oxoprolinase family protein

MTLRVATDVGGTFTDLVAFETLPDGSTKITTAKSDTTPPNFEQGVLAVLAKAGIQPSDVDFMAHGTTVVI

NALTERKGVKVGLITTRGFRDSLEIARGNRPDFFNLAYEKPAPFVPRYLRRELEGRMSYKGEEVDPLDLS

PLAGIVADFKAEGVEAVAISFLHAYANPAHEEAALAELRRIWPEVASVAAHQIAREWREYERTNTAVLSA

YVQPAAERYLRKLESGLMSMGLKSPPYIMQSNCGVDSLDATARIPITMVESGPASGFWGAAELGRLIGEP

NVLALDIGGTTAKCSLIEDGQVRIMTDYWIERDQRSSGYPIMVPVVDLVEIGNGGGSIAWVDEFSKLHVG

PQSAGAMPGPAAYGRGGQNATTTDANLWLGRINKDYFVGGEVTADMAATEAALQKLADKLALTPDDVARG

IIRIANNNMVNALKLVSLNRGHDPRDFTLVAFGGGGAMHAAALGAELGVKKVVIPTGASVFSAWGMMMSD

LRRDYFVTHLSDLNDGAAATLETAFTAAEDQARAQFSAEGVSADKVKLQRFGKFRYQNQEHTVEVPLSGP

VTAADIARISADFQEAYEREYTYRLKAPVEMVGLHLVATAEVGKLSMQAAPLGAPDATVAVKSSRDVDYA

EGGIHRATIYDGTLLRPGMEFTGPAVIEDPGTTIVIHPGNQVSVDGFGNLHIAL*

>gkv_312|gene_NONE|hydantoinase B/oxoprolinase family protein

MSVDPITQEIIQNSLQAAADEMFAAFRKTAMSSIIYEVLDMGTGILDARGEIACSGAGIPSFVGVLDKAV

KVIIAENGDDPIRPGDIFATNDPYYGGVTHLNDIIVAMPVFADGQVIAWTANIAHNSDLGGKAPGSLSGD

ATEIFHEGLRLPAIKIIDQGTPIAPVFKIIKVNSRMPDVLEGDVWAAIASARVGARRLEDIARKYGADAF

RTAMSNFMDYGEQVSRKALAALPKGTFSFAEDQDDGSVYKCTITITDDVFEVDLRDNPAQVAGPTNAVRD

GVMIAAQMLFKNLTDPWSPANEGSFRPIRLLTTPGTIADAVEPAAIGFYYEVEVRLYDLLWRCLAPHMPE

QLSSGHFSSICGTFIGGTHPDTGRQYTIIEPQIGGWGARKGADGNSAIFSGFHGETYNCPAEINEARNGV

WVDQMALNLAPGGEGEFTGGRGIVMDYRVRAQNGYLTANYTRSKYPAWGSQGGADGSGNIIEVRKVGETP

YRQAFISELPTSPDDVIRVITGNGGGYGNPKDRDPAAVALDIKNGLISADHAREIYGYQG*

>gkv_313|gene_NONE|metallopeptidase family M24 family protein

MSIETRLSALRAKMTDTGADLVALGPGPNMHWVLGFHPHPDERPCLLLVTATGAGFLMPALNAADARARC

DLPMWTWSDATGPQAALDQAIAALNASTTRKAAVDEAMRADFALLLLDALPQAKHAFAAETVGALRLVKD

AAEMEELRMNSAIADEAMEAAFAALTPGMRESDLAQVVKDVFAKHGASPLFTIIGGNENGAYPHHSTSDR

PLTQGDAIVIDIGARKGDFSSDITRMAVIGTPAPDYDKVHAVVEAAVQAALAAARPGVAAKVVDQAARQV

ITDAGYGEYFVHRTGHGMGLEGHEAPFITETSDTTLEAGMVFSIEPGIYLTDRFGIRLEEIVILHEDGPE

VLSKLPRDTYRRS*

>gkv_314|gene_NONE|bacterial extracellular solute-binding proteins, family 5 Middle family protein

MTIKHLMMASALALSSAAALAAPAAAERVLRVADSPIGEIDPHLGNDLTDTVLAVNIYDTLVFPRADGPG

VVPHLASEWAIDGAVYTFTLRDDVTFHSGNPLTADDIVFSFNRFMDMGQGLSSMFAGQVETVEAVDPHTV

RFTLTAPFAPFLSNLVRLHIVDSQLVLANLADGTYGEMGDYGAAFLNGHDAGSGAYMVTSQNPQTETVLA

KFDDYFGGFIENAADTVRYRYGIEASTMRALLARGEHDISDEFLPPEVIAALANDPNLHLISQGGSTGDY

IKLNTQRAPLDDVHCRRALSYAFDYENTLRILQINGEFSQGRPMRGPIPSALAGYNPDAPVMAQDMDRAR

EELALCQYDPAQYPLDIAWVAEVPARERVAMLMQATFSQLGFRVNVSKTPWALLTEQVSNAETAPHAAEI

GVSANSPDTDSLLYAMYHSSVAPTWMSAEHLKNDDVDALLDAARLETDEDKRVEIYSQLNTLLIDLAPSI

FAYESVGVYVARNSIDVPDMLNDDTRRIEMYNMNWNNISVADEQ*

>gkv_315|gene_NONE|asp/Glu/Hydantoin racemase family protein

MGARILWVNTVGWEAYDQPIADVLSAIKAKDTEVEVVSLSLAGRLTHVEYRAYEALTYPAIVGLARDAGQ

RDFDAMVVGCFYDPAVKEAREVSGRTHVIGPMLAAVQLATTVANRFSVLVTRRKCIDQMTDRIREYGAAH

RLASMRDLQIGVESLQKDPNATARAIIEQGRRAIDEDGAEAILLGCTCEFGFHEEAQQILGVPVIDAVSA

PFKLAEHLAGLKRQLGWVPSRVGSCEPPPEDEIDRFGLFQGPVSVGNRILLT*

>gkv_316|gene_NONE|binding-protein-dependent transport system inner membrane component family protein

MQNAYISRLVSALVALLGVSIVIFSIARIMPGDPARIALGPNATAAQVEAMREARHLNDPLPMQYIEYVK

SVAQGDLGHSLYTNRPVTTDIAQFLPPTLELILISALLMVLLGLPLGVISARYQGKWPDNAGRILSLIAI

CTPAFVWGVVLQLSLGYIWPVFPLEGQIATSMRPAVVTGFMLIDTWIAGDPRAFLNALYHMVLPALALAL

SGIGQCARLTRSNMIETYQRPYTEMVRAYGVKPGRIAWRYAFRPAFIPTLTILGLEFAALLGNAFLVEKV

FGWPGLSRYGVEVILRKDLDAIVGTVLIIAAAFLIMNILVDLLVTLVNPRIRLSPRRA*

>gkv_317|gene_NONE|binding-protein-dependent transport system inner membrane component family protein

MQLSNITRSPGWTAFFASPMSVIGLIIVVVIVALAALANIITPYPTHVGPIGDFAAMNQAPSAAHWAGTD

PMGRDQVTRIIFGFRLALIMAAVVLVTSVPVGIAVGLLAGYKGGWTEYLLMRLTDIFLAVPPLVFAMAIM

GFMEPTLLNGMIAITALWWTWYARLIYSVTRAEAQEGYVLAAETIGASTFHILFREILPNCLPTIITKMT

IDVGFVILMAASLSFLGLGVQPPTPDLGAMVADGAKYMPDSWWLSLFPGIAILIVVLGFNLLGDGLREAF

DAGK*

>gkv_318|gene_NONE|putative peptide ABC transporter ATP-binding protein y4tR

MQDPVLTIRDLRVAFGPRKRAAQVLHGISMHVRPGEKVALVGESGSGKSVTARLVMGLLQESRDTHASGS

IRFDGIEAVQGGAQIAARRGNRVAMIFQDPTSALNPTFKIRGIFRDVIRTKDPKITDAAADAKAEAALAE

VQIPDPTRALDSYSFQLSGGMNQRVMIAMALINQPSLLIADEPGTALDVTVQAQTLKLMGDLAASRGTAV

MLISHNLGVVREFSDRVYVIYRGNMVEHGRNDQIFGQPQHAYTQALLSAIPKISGGGLPDLPERSPAFEQ

PLIQHPGCAEPLEA*

>gkv_319|gene_oppF|oppF

MNDIILETIGLGKSFETASGPVHALQNVAQKFTRGGCHAIVGESGSGKTTLANLILGLMQKTTGEIVFNN

QTLPEHRIKSQRRAIQLVQQNPLSALNPRLSIGASVRLPLDVHDIGPRAGRDKRVADLLEEVGLGAEFAT

RSPRGLSGGQRQRVAIARALACEPDLIVLDEPTSALDVLVQARVLRLLDNLRRERGLTYLFITHDLAVVR

AISTTVSVFQRGRMVESGTVEQIFTNPQADYTRQLIGAVPVVTDDELALRDAIRAGAPY*

>gkv_320|gene_NONE|hypothetical protein

MKPDLSHALQLAAGEDAAARVFDYARAGVTAIAPSGLTTASVYDLPNMRTRRVFSENPEAYGTGNFKRVD

RNVYYDTVILGQRPFVSNTVAEFAAAFFDWQKIEDLGFSANMNIPAIADGRVIGTMNLLSTAGTYAPEVV

AGAMEWQPVVTLAFLLLHLEGAEHATFHGKGASIDLTPNLEGL*

>gkv_321|gene_NONE|hydrolase CocE/NonD family family protein

MTIETIEHIWIPLADGTRLAARIFLPAGARSKAVPAILEYIPYRKRDGTRGRDAPMHGYFAQNGYAAVRV

DMRGAGDSDGHMADEYLLQEQDDALEVIAWIAAQDWCDGNVGMMGKSWSGFNCLQVAARRPPALKAILTA

YSTDDRFRDDIHYMGGNLLNDNLWWGSIMLAYQARPLDPETAGAGWRDAWLKRIEDMPFFPAIWAQHQRY

DDYWKHGSVQEDWSAIQVPVMVIGGWADSYTNSVPRLLANLQVPSRGIIGPWGHIYPHDGVPGPAIGFLQ

EAVRWWDHWLKGADTGVMDEPQLRAFINDAVVPVGTRTDQPGKWVGEAAWPSDQIADKLLHLGAGGRLTE

TAGVAAQLPVKSPQDHGRAAGEWMGTGCVGEMPTDQRLDDGGSLNFDTDVLEDAVEILGAPVVRLKLSAD

APVAQIVVRLSDVLPSGEVLRVSYQVLNLTHRDSHENPQALVPGQDYMVPVTLSACGHRFAPGHKIRVSI

GTAYWPMIWPAPYAATVTVDTGGSAISLPVRMGGDGAVAFDAPAHGPAVPITQVDPGMTARSFSFDAITG

LATYVTEGRGGLFGEGVVRFDEIGTTIAHNLRRELTIDPRDPLSARYVLRQSYDLGREGWQTRSEITTEM

TCDLENFYIAGALVAFENGTEVARRTWHETIARDLM*

>gkv_322|gene_NONE|ABC transporter family protein

MSAHILDIRDLTVEAPNGAVLLHGVSLDLAPGEVLGLIGESGAGKSTIGLAAMGYGRGGCRITGGQINLV

GTEMRGATRKTREALRGARIAYVAQSAAAAFNPAMRLEQQILEVPLLHGLMDKSAARAWMIELFTALQLP

SPETFGDRYPHQVSGGQLQRAMIAMAMAGKPDVIVLDEPTTALDVTTQIEVLALLREVIRRYGTAALYIT

HDLAVIAQVADRLMVLRHGREVETGATAQILAAPQADYTRQLVAERQGRFTRSAGHQIGADVLAISNVSA

SYGSTPVLHDVSLTLGRGETLAVVGESGSGKSTLARLIVGLLTPTSGDVRLKGRALPATFQARSKDDLRR

IQLIYQLPDVALNPRQTVAEAIGRPLTLLRGLKGAAKAAEVARLLDLIGLPRDFADRLPSALSGGQKQRV

CIARALAAEPELVICDEVTSALDPLVAEEILTLLRKLQDELGVTYLFITHDLSVVERLADRVMVMQHGRV

VEVGETAALFATPQAAYTRKLLDAVPQLRTDWLDEVMAKRQP*

>gkv_323|gene_NONE|binding-protein-dependent transport system inner membrane component family protein

MKKLTFSAVLGMAIVAIAAFGFLFGPMLAPYGLEQVVGMPYDVPSAAHPFGLDQNGRDMLSRLLHGARMS

IGVSLSAVVISFCIGVPLGFLAALRGGWPDILGARLVDVVMSIPVLISALVVLQALGASLPVLIGTIALL

DSTRVFRLARVVAQGVVVMDYTEVARLRGESTWWLLRREILPNALPPLMAEFGMRFCFTFLFVAGLSYLG

LGVQPPFADWGGMVRDNQQGVLYGLYAPLFPAGAIALVTIGVNLVVDWALAGRTTVQGDNR*

>gkv_324|gene_NONE|binding-protein-dependent transport system inner membrane component family protein

MILMILQRLGLGVLTLVLVSVLVFAGTEILPGDVAAAILGQNATPESLAALRADLGLNAGPFVRYFAWAG

SVLQGDLGQSLANRQPVADLLWPRFWNTMALAAYAAVIAVPLAVGLGILSAAWRGSLFDRAANVIALATV

SLPEYFLGLLLILFLSVQFGLLPSLADTYAGMGFPAWLRATTLPMLVLVLVTVAQIMRMTRTTVLAVMDQ

PYIETAFLKGLKNGRVVLRHAAPNAIAPIVNVVAFNMAYLIAGVVLVEAIFNYNGLGRFMVDAVSKRDLP

MVQAAAMVFAAVYVVLNMIADIAAIALNPRLRHPRVKG*

>gkv_325|gene_NONE|bacterial extracellular solute-binding proteins, family 5 Middle family protein

MTNSNNLLIGRRGLLAGAAGLSALSLLPSRLRAQETPRSGGVLRMGIGGGSTTDDFDIRKLNDWVPVNQA

YMVMNGLVEIDADNIAQPELFESWEAAEGAAEWIFNVRQGVTFHNGKVLSADDVIYSLNLHRGDSTSAAR

SIASPITGLEKLSDFQIKITLASGNADLPYMLSDYHFLVVPEGFDDWSNPIGTGAFKLESIEPGVRGRFT

RNVDYWKPNTAHVDAVEVIVINDISARTNALMSGQVHAINSLDFKTVNLLGRNPNLNIIRSSGGQHFTFL

MDTRIAPFADVNVRRAIKYGIDREQLLQTALLGFGQLGNDHPIPRTDRFYNSELPQRAFDPDQAKFYLRE

AGMESLSLTLQTSDAAFPGAVDAAAIYRTSAAGAGIDVNIQREPADGYWDNVWMKAPFSMGYWGGRPTAD

QMLTIAYSSASAQNDSHWANERFDSLLVEARALLDEDKRREIYWELQQIVSDDGGVMIPMFGDYLDGVNK

AVKGVTPHPMFNLMGARMAEKVWLDA*

>gkv_326|gene_NONE|bacterial regulatory helix-turn-helix protein, lysR family protein

MSRMKVPPIAALVAFQTVARCGSISSAAGMMGLTQSGVSRQIARLEDFVGAALFDRTASGVVLNAFGQDY

ALQVGRVMDALGTLEDTVQGARDRSQVVLACSQGVADLWVLPRLQQLRHDLPWLVLKLRVDENIALLRPD

EYDLALYHRPARMADFVMEPLGPERMVPVMAPGQPPLIQQAAPLLLTMEESFKEWTDWGNWLYSAGVELP

AGAMRWKMGSYRLAIEAARQGIGVAMGWTWVVQDLLDAGALVEAHPHHLEGPGHYYLLRSAQRHQRSSAK

KLADWIQAKNRVLDRPAETTVTGV*

>gkv_327|gene_NONE|putative membrane protein

MGMPDFIGLLGAVFYLSAYAMLQLGKLKLEDNAYAGLNILGAIAILTSLIWSFNLGALVTQSAWLVFTVL

GVIRSRMRRAALPPNPAGHA*

>gkv_328|gene_NONE|glucose / Sorbosone dehydrogenase family protein

MSQRNLRKALATSALALAFAGAASAQDFNEAPPNDPNQSPAFEGQTRAPVIADDIALSKDVFAGGLVNPW

GMDQLPDGSWLVTERPGRLRHVGADGTLSEPITGIPEVDSRGQGGLLDVTVADDFDSTRRIWFSFAEPRG

GETNATAVGTGILSEDNTALSDVEVIFQQQPAWESTLHFGSRLVFAPDGALFVTTGERSLPEPRELSQDV

TTHLGKVIRITPEGGAADGNPAIEGGLPEIWSYGHRNLQGATIGPDGELWTIEHGPRGGDELNRPEAGLN

YGWPVITYGINYSGEAINTGITAQEGMEQPLYFWDPSIATSNMVFYEGDLFPDWQNSLLIGSLAGQSLVR

LTLDGTAVTGEARYFAGEGRIRDVDVGADGAIYLLIDAEDGQMLRVTPAE*

>gkv_329|gene_NONE|conserved hypothetical protein

MGTRYYIRWYLALTLIGVLPIISVTLAGLIATVNGCVLHEGGVSPCLIMGHDFGGALHTMTVLGWLMLIT

NFALLAGVLGLVWEGVKAVFRAIFNR*

>gkv_330|gene_NONE|hypothetical protein

VSALQPLPQDAWDAWTPDQLAKRLACFTGDWYVVGGWALDLWHGAQTRAHEDLEFAVLADHEQPCRQALK

DLSFFTAHDGTLAYLPLSAPLPADIWQQWGADMNTGLWRVDMMVDRGTPDLWIYKRDPALTMPRTDAIRQ

TPCGIRYLAPHLALLFKAKYARDKDNTDFQTALPRLTRAETADLTLWLDRFHPQHPWITALQNR*

>gkv_331|gene_NONE|cytochrome c family protein

MTHRFMTTLAVSMLLAAPALAQDADQLAHGRSLVETNNCTGCHTQNLGGQFFGGWYVPNISSDEASGVGS

WTADELVAYLRDGTSAKGQAAGSMAGVVGSITRHMPDADLQAIAAYLKSSDPVVSFERTTEAAAPGSFVD

AKPASLASLDPVQSTDPAAHVDLSVTDGATLYISACATCHMPNGEGIEGQFYPALTGNTTTGTYVPNNLV

QVILYGVTRDSNRGMPVHMPAFGNDLSDEQIAAVANYVFDRFGNDDLSVSGDDVALLRARQNLPGQQ*

>gkv_332|gene_gdhAlpha|FAD dependent oxidoreductase family protein

MADFDADVIVIGSGAVGSNAAYELAKQGLSVILMEAGPRLPRWKILQNFRASPRKGNHNDPYPNLPYASN

SFTDGYLENTGSFDLRPGMLRLLGGTTWHWAAATWRFTPEDMKLNSLYGVGRDWPIGYDELEPFYGLAEV

ELGVAGSDTQDQSGQGRDLAYPPRSTPYPAPPEADTYYFARLRDRIGAEGYNFVHEPNARPTVTYDGRPA

CTGNNNCMPVCPIGAMYSGNMTAQKAEDEGVQIITEATAYNLEKGEGGKIVAVHYKTPDAESHRLTAKYF

MVAANGFETPKLLMISDVANSSDQVGRNLMDHSGMGLQFLADEALWPGRGPVQQGGIFNWREGEFRREHS

AIKHALSNNVPNKMIAERLLAQGIVGTELDEKIRDMSARFVDVSTVFEMLPHPENRLQPHATRKDALGIP

TLSINYDIDDYVKAAVPVVKEDYANFVRIMGGEVIEDDTGFQNRDHIMGTVIMGDDPATSVVNGECRTWD

HDNLFLATTGVIPASGLINPTLTAVALAIRSAQIIAKEI*

>gkv_333|gene_NONE|tat (twin-arginine translocation) pathway signal sequence domain protein

MDIKTTPFDMRLSRRSLLQRAAFASVIAFGASVAGTAVFAQGATALNFVTLSQVLTMRDALDPEIGARAL

DNLTADDADFPAKAQALADAIAAEGFDDMDAFGTFIAGHEDLRETAMKIISAWYLGYTGTMSGNSFNDTA

RFVTYRGALMYEPTMAETVIPTYSRGAPNYWAEAPASVARD*

>gkv_334|gene_NONE|bacterial regulatory proteins, lacI family protein

MPRRTHDHTTFEAIAAAAGVSLSSVDRVLNERGSVSEKTRQKVLAAAREMGINRNLPEAWHRVQHVDIIL

PRNQAMHWQVLDQTFQALGVNVPRWLSLHRQRLPQNDFKALRDALLAPPHQRAGLIIAADAAEDIAPALR

EVMARGEKLVTLTTEVPGLPAHGFSGINNITSGRTAGYLMRRLGSLTDPGAKVLIMQGNARRLEHFQRVE

GFRAGLGDIARQIFMHVDETEAGAAERALRRVLADGERISGLYCTGTYSEELGPLMAAMGGARPIWITHD

KTPAHEALMQRGLLDFVLDQDSAAQAAWALALMITMLSGEPWTGAPQLAPELRLFCCENLSN*

>gkv_335|gene_NONE|hypothetical protein

MQSMQIRKLVGRGSATRKYDLLTVLGTYALSQDRSLQRQVLRLICLLTARYNWQSDQLSVGQAEIARLWS

VDPRTVKREMAAFRDMGWLVEKRPAARGRVTLYGLGMARIITDTRGTWEKVGPDLVARMDDNLPEVPGAD

SGYVMGGGAQGAEVIPFPQSLPDEPAAQLWADMARQLHAENAGTYQAWFAALRPAEQPGYLVLHAPGGYH

ATYIRTHLMARLEAALARVAPGLGVMIAG*

>gkv_336|gene_NONE|cobQ/CobB/MinD/ParA nucleotide binding domain protein

MYTHEDLAALQAQSLKMQSFIRKQTFSPEHEKQLRRFSSWEVSELIFKINQSTFRGRLAQDPDLPGGEVE

DDGRQRWFSLNEINELRRKIKINRHSLMPPRPAGKRAIRVAISNFKGGAGKSTVALHMAHAAALDGYRVL

VVDFDPQATLTHSMGLHDVSEEFTVWGIIARDLVRETDRMNAAPRAAESGTALPQRRLPAAVRDLGLGEL

RVTDFIKTTAWPTIDIVPSCANAAFVEFASAQYRHLNPDWSFFAAVSRYLDAVPRDSYDLIIFDCPPAIG

YQSMNAVFAADVLYIPSGPGYWEYDSTTSFIGQLAEALADLSAGFAGTFPAGKMRLPKTFLDIRFLMTRY

EPGNELHRAMLEAFRKVFGDTVAKEPIEMTRAVEQSGRFLSSVYEIDYRTMTRETWRRARASFDRGYDEF

KECFLNAWDKMEVEE*

>gkv_337|gene_NONE|parB domain protein nuclease

MKKRRIFDITLPDEDDEIFPAGKIEPEKTEQRRSPMATAISENAGALRERSAIEAEIRAENDALAAEHVR

MKQLGLMVDLIPLDQIETYKLVRDRRLGDDLELAELTASIQAIGLSNAIRVEQREDGKYELIQGLRRLSA

YRALLKESGDAEKWGRIPAGILPRGEDLEHLYRRMVDENLVRKDISFAEMARLALDYAADPGTKQSDPDR

VVAELFQSAGYQKRSYIRQFIRIMDVLGLDLQFAPHIPRALGLKLSTLLDERPEVAHQIRTVLRAMPNRS

VADELNLLRQITDGQPAPSDEASDAPVRATTGGVAAPVSRRAKTTFQVPSHMGQARCTAANGRLEIKLDC

DFSALDRRKLELALTKLLSDLE*

>gkv_338|gene_NONE|phospholipase D Active site motif family protein

MTQHTDILIPHKTCWRIAPAGRFAFIDDGAAYFAAARQAMLQARHSILLIGWDFDANITLDRTPDGAEDE

GPARLGDFIIWLADRTPGLQIRLLRWDTGAIKSMLRPSELATVIRWKMHPRITLRLDGAHPPAASHHQKI

VAIDDALAFAGGIDMTMQRWDTPEHLDDDPRRTTPRGRPLPPWHDATSAFDGPAARAIGDLARARWLAAT

DEALPPCPAPHDCWPKDLAPTFTDVSLGIARTLPKMDGQDPVHEIEAAWLAMIASAKRMIYAESQYFASR

KIAHAIALRLTEDDPPEIVIVTPHSADGWLEPIAMDTARAKLIEALARVDHKKRLRLYHPVTAAEAPIYV

HAKVTVVDDDLLRVGSSNFNNRSMRLDTECDVILSHPRAADLRATLLAEHLGTTPEVVTSTLAETGSLIA

TVEALRGPGRSLVPYVLPELSSLAEWLAENEILDPNGPDEIFESLSKRGLYKGWQHLLRGRFRRAPR*

>gkv_339|gene_NONE|major Facilitator Superfamily protein

MAHKPSLSQRLAARGIHYGWVVAAATFLTMMVGASAVGAPGVLMLPLQAEFGWSLADISAAFALRLMLFG

AVGPFAAALLARFGTKHVSLVALAMISVGVVGSFFMSSLLELFVLWGLIVGFGTGLTAMVLGATVATRWF

AARRGLIMGLLSASVATGQLVFLPAFAALTQALGWRMALGLLLAMLALATVIVLTLMRDDPADIGLRPYG

ATGDAPTPSAAPTASLGATLMVPIHVLRQNLNSGTFWILFGTFFVCGASTTGLIQTHMIAICADFGIQPV

AAAGVLAVIGGANFIGTIISGWLTDRFDSRWLLFWYYGLRGLSLIYLPFTEFGMYQILLFAVFYGLDWLA

TVPPTVRLANDRFGRDATIIFGWVFFGHQLGAAMAAWGAGIARGFYASYLSSFYAAGFICIIAAVSVFYI

RRRTLPT*

>gkv_340|gene_NONE|marR family protein

MSDCHCTTLRAATRRIGALYDGALAPFGVSGAQFALLRRVRALDGPSLSQLAQNLELDRSTISRNTRVLQ

RAGLLQLGPCAHDKREQTATLTAAGAALLHDAAPVWQSCQDEIATRLGPARTAVLHDLLDLI*

>gkv_341|gene_NONE|bacterial regulatory proteins, tetR family protein

VVETAAKLFREKGFDGVGVSSLMQAAGMTHGGFYKQFTAKEELIAEAMGAAVAQSRARLAAAAPDDPARF

AQIVRMYLSPAHRDAPGEGCALAALAAESGRHGAGLQAAAEAGVQDYLDGLTAVLGDPARAAAVLAQMVG

ALVLARAAGQGALSDQILAQNIAALLEE*

>gkv_342|gene_NONE|short chain dehydrogenase family protein

MNMSLPKVLITGASSGIGATYADRFAHRGHDLVLVARSAGKLADLAARLRAEAGVAVEVLVADLAVPEGQ

IAVEAKLRQDAQIGILVNNAGASIAGAFTDQDIDVATDLVNLNTISLMRLTHAILPRLKAAGQGAVINIG

SVVGMSPEFGMAVYGATKGFVLSFSQALQVSLQGSGVYVQAVLPAATRTDIWAGADPAHLPPMMAVDDLV

DAALAGFDAREAVSIPHLHDLDRWTAFEGARIALLQDVGNITPAPRYAAAD*

>gkv_344|gene_NONE|protein yhdH

MQFKALLATAADHAAVVDFDADDLMAGDVTVRIDYSTVNYKDALALGGGRIIQRFPLIASIDFAGVVEAS

GSADFQIGDRVVLNGWGLSQTHHGGYAQVARVPSEWLVKLPDSISTFDAMGIGTAGYTAMLSVLALEHGG

LTPDRGDILVTGANGGVGSVAIALLSGLGYRVIASTGRVDQGDYLRALGAADVIDRAELSSPGAPIGPER

WAGAVDSVGSHTLANVLAQTAYRGVVTACGLAQGLDLPGSVAPFILRNITLSGIDSVNAPKAVRQTAWAR

LARDLDLAKLGRAVSVIGLNEVRDTAAAVLQGRVQGRTVVDVNA*

>gkv_343|gene_NONE|molecular chaperone, DnaK

MQNPVLAVDFGTSNTAAAVYSGGAPRRIPLEEGADTLPTAVFFPVNGGPMLIGSEATRALIDGHEGRFMR

ALKSILGTALFHEQRLIGGKRRTLANIVTDFLIALRQRAEARTGLTFTAVLSGRPVHFHSADPLRDARAE

DDLRACYHAAGFTQVDFLNEPEAAAHAAPEAEGLGLIVDIGGGTSDFTVFRRNGAQIETLASHGIRLGGT

DFDQSVSLAHVMPLLGMGGTLKRDFGPGLLPVPQAIYVELATWAKIPFLYTPETRRAIADMQRHASNPAA

MAHLATVIQDELGHSTAFAVERGKIDANDGRDGRIALGFIARGLTAGVTPGSLHVALSDYAPRMQAAMEE

TLARAAVSADQIDHIVYVGGSSLMGIVTQSASAIAPAARALRAEAFTAVVDGLAIASAKAA*

>gkv_345|gene_NONE|pca regulon regulatory protein

MRDTMGSLAKGLTVIEAFSADHPRLSITEAAARTGLDRATARRCLLTLVESGYATHDGKFFTLTPRVLRL

GVACLATMPLPQIVQPYLDRMTDAIGESSSVSILDGADIVYIARASTQKVMSIALMPGSRLPAYCTSMGR

VLLAALPADQRPPLTDLPARTPYTLTDPAALTAVLAQVRAAGYATIDQEVEIGLRSIALPLMNGHGRVVA

AVNIGTAASRTTMADLTTRLLPALGALQTELRGLIK*

>gkv_346|gene_NONE|3-oxoadipate CoA-transferase subunit A (Beta-ketoadipate:succinyl-CoA transferase subunit A)

MDKQVSDLAAAVAGIESGAVVMIGGFGGAGAPIELIHALIDRFHATGAPVDLTVVNNNAGNGHVGLAALI

EAGMVKKLICSFPRSADPVVFTTKYLAGEIELELVPQGTLAERIRAGGAGIPAFYTPTSFGTDLAAGKPT

AEFEGRAYVQERWLRADYALIKAEVADVQGNLTYRATSRNFGPIMCTAARVAIVQARAVVPAGQIDPEAV

VTPGIFVQRVVQVANPAQEEDLNRANAAYPLEA*

>gkv_347|gene_NONE|3-oxoadipate CoA-transferase subunit B (Beta-ketoadipate:succinyl-CoA transferase subunit B)

MKLSNNQIAWRAAQDIEDGSYVNLGIGFPEKVAQFQPAGRQAIFHTENGILNFGESPAAGAEDWDLINAG

KRAVTLNPGAAFFHHADSFAMVRGGHLDVAILGAYEVAENGDLANWSTGPKGVPAVGGAMDLVHGAKRVA

VITDHVTKDGRPKLLERCALPLTGVGCVTRVYTSLAVIDIEGGRFVLREKLAGMSFDELQAVTGATLHVV

GEVADLIVPEV*

>gkv_348|gene_pcaF|beta-ketoadipyl CoA thiolase|

MRDVYICDYIRTPIGRFGGALAMVRPDDLGAVPLRALMARNPGVDWAAVDDVIFGCANQAGEDNRNVARM

SLLLAGLPVEVTGTTINRLCGSGMDAVIAASRAIAAGEADLMIAGGVESMSRAPFVMPKAETAFSRAAEI

HDTTIGWRFVNPAMEKAYGVDSMPQTGQNVADDFGISRADQDAMALRSQANAARAQGDGRLAREITAVVI

PQRKGDPKVVSQDEHPRATSPEALAGLRPIFAGGSVTAGNASGVNDGAAALILASAEAAAKHGLTPIARV

MGGAVAGVLPRIMGFGPAPAAKKLMARLGLSADDFGVIELNEAFAAQGLATLRDLGIADDDPRVNPNGGA

IALGHPLGMSGARITGTAALELQGGAARYALSMMCIGVGQGIAIALERV*

>gkv_349|gene_pcaD|3-oxoadipate enol-lactonase|

MQVLARPWGAMHYRIDGPADGPVVVFANSLGTDLRLWDGVVARLPGFRCVRFDLPGHGLSDLAAEVSISA

LAEDVAALIATVASAPVVLVGLSIGGMIAQELAVKRPAMLAGIVLSNTATKMGSADAWAARIAAVEAGGL

ASIADQVMERWFAPAFRARATLPLWRNMLLSTRADGYIVACRALAAADLTGQAAEIDLPAVVIAGEKDGA

SPPDLVAASAARIAGARLHNMAGVGHLPPAEDAAGMVAIIAPFLKDVLK*

>gkv_350|gene_pcaC|4-carboxymuconolactone decarboxylase|

MTDRFAAGIATRRKILGDAHVDRATQHATPFDQPFQVMITETAWGNLWSRGTIPARERSMMTIALLAGLG

NYEELSLHLRATVNTGASPADVVEALLHVAVYAGVPRANHAFKIAKEVFTELGIDTSQIPSAK*

>gkv_351|gene_pcaH|protocatechuate 3,4-dioxygenase, beta subunit|

MTTTDQGPLYFRDRDWHPAAYTPGYKTSMTRAPFRPLVSLGSTLSEETGPAFGQAMIGELDNNLIMNFTG

QPAIGERIILHGRVLDENGRGVPGVLVEIWQANAGGRYRHKKDGYLAPLDPNFGGCGRTLTDASGGYEFL

TVRPGAYPWPNRVNDWRPMHIHLSVFGSGFGQRLITQNYFEGDPLIARCPIVQTIGQQKAIDVLVAPLDM

NRAIPMDCLAYKFDIVLRGRRQSYFENRKEGL*

>gkv_352|gene_pcaG|protocatechuate 3,4-dioxygenase, alpha subunit|

MQRLDYLKETPSQTAGPYVHIGLVPNALGIPGIYPEDLGRAPVTDAAKGQRISITGRVIDGAGMVLRDAL

FETWQADAAGIYPVNDPRGPADPGVTGWARVAADFDTGIWRIDTVKPGRVPYPDGRLMAPHIAVWLVARG

VNLGLQTRIYFDDEADANEACPVLGRIEHRERVATLLARHQGNGQYQIDFRLQGEGETVFFDM*

>gkv_353|gene_NONE|lyase family protein

MPASIFDSAIYRGLFGDAEVEALFSDSAVVRAMLLVEGALAEVQGELGLIPLDSALFITRASREVQIDPS

GFARETAVNGVPVPALVAAFRKAMEAPEHAQYIHWGATSQDIMDTGLALRLRRAVAIIDARLGSLIGRLG

ALAADHADLPMAARTYGQFATPTSFGALIASWGRPVLRHRARIAAADVAVVSLGGAAGTLSAMEGQGPAV

RAGLAARLGLSDPGVSWHTERDGQGALMDALVGLLVSLGKMGEDLILLTQSGVDEVVLAGAGGSSTMPQK

QNPVGPSVLVALARYSVAVGGAFQGAGLHRQQRDGAAWFTEWMVLPQIVIAAARALALAEDLARGLRPDA

AAMARNMLSGGGLIAAESYSFALARQMPRPAAQAKVKALCAEVVQSGVPLADLMARDFPGLQADLGLGEA

PAEARAFADEAHG*

>gkv_355|gene_pcaQ|pca operon transcription factor PcaQ

MAPRVIDQRIKLRHLTCFWEVARLRSVGKAADFLNISQPAVSKTIKELEDILSQALFDRTRRRLTLSPFG

EVFYGYAATSIAALRQGIAAAQHEVQRPILRIGALPTVSAQILPETVKALGAAHPGLRMRILTGPNDYLL

GLLRTGDADLMIGRMARPAQMLGLSFEHLYFEQVVMAVRPGHPLLAEQGFEIAMIEPYQLMLPTPDSLIR

RLVDQMLMANGVTEPRDEIETISNAFGRAYVMQSDAIWLISEGVVKRDLAAGTLAALPADMVDTMGPVGF

TTRTDALPNFEAQLFMQAVRAAAAQLRG*

>gkv_354|gene_NONE|regulatory protein

LDNPTMDQIPSYALYGEATQRPEWFHAETIPARSRRHGFTIAPHRHSHLFQVLILTAGRADMVLDGETHQ

LHAPAVAVLPALSVHGYTFSRDVDGHVLSLLTSALPRDILPAAAVLRDAAVIAAAHDLMHEPADPYAQQA

RATLLLIAINRATQTPPAPGTHLSAFRVLIEQHFRTPRPIKTYASALGISQTHLARLTRAGTGQSPLEMI

EQRIALEIKRNLLFTNRSMKQIAADLGYDDPAYFSRVAARLLGQSARAYRANSVIRATAPPPPAPPA*

>gkv_356|gene_pobA|4-hydroxybenzoate 3-monooxygenase|

MRHQVVIIGAGPAGLMLGRLLEIAGVDAVILEQRSPEYVLGRIRAGVLEQGSVDLLRRVGAGGRMDAEGH

LHAGASFEFDGDALRIDFAELVGRQVMVYGQTEVTRDLMALRQAPTIYGAEDVALHDFGGAAPYVTYRKD

GVEQRIDCDFIAGCDGFHGVSRASVPAGAITEFQRVYPFGWLGVLVDQPPAADELVYAHHARGFALCSQR

SPSRSRYYVQVGADEKLADWSDDRFWDELRARLSPKVAAAVQTGAAIEKSIAPLRSFVAEPLRFGRLFLA

GDAAHIVPPTGAKGLNLAMSDVEMLGDALIEALVERSDAGIDAYSSRVLRRIWKAERFSWWMTNLLHVFP

DGGDFGRRIQRAEFDYLAGSRAAQQSLAENYTGAF*

>gkv_357|gene_NONE|high-affinity branched-chain amino acid transport ATP-binding proteinlivF (LIV-I protein F)

MSAASLNITGLTAGYSQTRILEGIDLSVPAGGKIAILGRNGMGKTTLFATIAGQTRRHAGQIMMDDTDLT

ALDGAARARAGLGLVPQNRAIFRSLTVEENLATGLKDRPRSAIDEAYALFPRLAERRRNMGGQLSGGEQQ

MLTTARTILGQPRLLMLDEPLEGLAPVICDALMEAFTQLAASGQMTILLVEQRLKAAFDFADHIVILERG

QVAWQGTSDALQSDPATVERLLGVGH*

>gkv_358|gene_NONE|ABC transporter family protein

MSILEVRNLSKAFGGLQVTDDVSLTLNPGDRVALIGPNGAGKTTFVNLVTGHLKPNAGQVLIDGQDMTRA

SPTARVHAGLVRSFQVTRLFPDMTPEEHVALAILQRMGRAERIFADYRAMPDVMDEVFDILQLLQLSPLA

RTAVRDIAYGQQRLLEIALAMALRPRVLLLDEPAAGVPASDTVLIEKALDQLPPALAVLMIEHDMDFVFR

FARRVVVLAAGRLIFDGTPAEVAADPQVREAYLGSYAE*

>gkv_359|gene_NONE|branched-chain amino acid transport system / permease component family protein

MNTISLWRRGFKGDLITIAVMVTLAVICFHFMPSNLSFFTRIIAIMLLVLAIDLVTGFTGIATLGHAALY

GAGAYAAGIAAAKFGINDPVLMLLVGIVAGALMGLLSSLVILRGHGLSQLVLSIAVVQLAREAANRFSTW

TGGSDGLAGISPAPLFGMWRFDLWGRTSYWLAVALLIVTFITLRQIIRSPFGKLCQAIRQDPVRVRAMGA

PVYLTQVKMYVISGAVAGLGGALSAVATKVVGLDSLSFELSAEAVVMLVLGGTANLYGALIGSAVFMWFE

HTMSTINPYHWLTIVGALLVAIVLFAPRGLTGLAESLWARLMKGAFK*

>gkv_360|gene_NONE|branched-chain amino acid transport system / permease component family protein

MQTIFSIGADALAYGMVLFVISIGLSVTMGLMKFVNLAHGAFAMLGGYIASYATQSLGWHFGFALLAAVA

LCMILAVPLERFLYRRFYGASELRQVLMTIGLTFMMIGIANWLMGPTLKTIALPPSLQGPVDIGFRSFPA

HKIFAAAVGLITALGLWLLIERTFFGVKLRAAVDDAKMAEALGIRTKAVYAISFALAIGLAAMGGVVGAQ

LLPIEPQYALHHMVTFLVVVCVGGAGSIGGALAACLILGMVKTTGAYLWPEFGNFFFYLAVIAIVSMLPN

GLMGRAK*

>gkv_361|gene_NONE|receptor family ligand binding region family protein

MKFLCQTTAILALCAGGAFADTIKVGVIGPFSGGAALQGRNFQAGIEAYFALNGRTVGDHEIEIIYRDLP

AADPGQSAALTQELIVGEGVQYLAGYYYTPDAMAAAPILEEANVPMVVFNAATSAIVNASPLVVRTSFTT

FQTSTPMATVARERGIERVITVVSDYGPGVDSETAFVRAFGAAGGTVVESVRMPMSTVDFSPIMQRIRDS

GAEAVFAFLPAGPQTFGFMTAFVENGLKADGVQLLAPGDLTQESDLPVLGPNAAGMLTTFHYAVSHNSPE

NIAFTEAATAWLGNPAELSFPAVGAFDGMHVIAHMIAATNGQQDAAAAVDSVKGLEWISPRGPVSINPDN

RHITQNIYLREVAEVEGQYINHEIQVFENQGDPGWVAP*

>gkv_362|gene_NONE|sorbose/sorbosone dehydrogenase(SSDH)

MQRSLLAQALGLAALLALPASAQIADYTPLDEQTLRNPSPGDWLQWRNTDNGWGYSALDQITPDNVGALR

MVWGWAMEPGQQETGPLIYNGTMFLANPGGLIQALNAETGDLLWEYKREFGDTVRPGAITRGLAVFGDHI

YYAAPDAVMVALDARTGQIAWETVVADTQVGHYFTAAPIVAEGMVVAGYQGCNRFREDKCAIVGIDAASG

EEMWRVDTIEPESEGDSWGGTPHLLRGGGDIWTSGTYDAENGLVLIGVSQAKPWARASRGHDGATLYTNA

VLAIEPATGEVAWYRQYIPGDSNDMDEAFEHMLIEIEGSDRYVNMGKLGILWQGDPADGAATAAYDLGWQ

NQIDLQGAAFADYRDGMVPDVNVPIAMCPSTAGFRSWRAMAFSPETRAVYVPISMNCDAAMIYREVEMVE

GGGGNGQAGSTRTMHPDSPENMGRFVAMNVDTGDVLWQHDMRAPANTSTLTTAGGVMFAGDWDRNLYAFD

QATGEVLWQTRLPQAAQGYLAAYAVNGRQYIAVPIGVGGASWSTSLPITLLPEIRRPSTGNGLLVFALPD

VN*

>gkv_363|gene_NONE|hypothetical protein

MARQIAYGMMLALLATAAQAQQPYIPPEFYDARRPDFGQSLPLCLLPDSVTAAIDDEMARLIADRLLLQP

ATVPMQVNMGAMDEDGIWPDLFVHLTEVCVGMLGAQLITGAPYPDWLIISRPYYSAPYLLVSLDPAIRSL

ADLPEGSFIGSPIYTPLDTELARLIGAGAAPLNRLPYDDIDQVHRFLADGTMAAAIIWGPYTRALPPDGL

HIGTTLAPLHTTTRSLGIVMRSRDRALMGMIDSAIASLADDGLLPTFTPTGD*

>gkv_364|gene_NONE|glycosyl hydrolase (secreted protein) domain protein

MTFRTLPRLAAAGAIALTCTITAPANAQSITAIANGVCQGLDPTCYNDWGGRDPAANGYSVLIYSRVGET

VTNPHANIAYGVQRLTELLEGADIKVTTSSDLADVQGPRQLRAYDTVIFFNTQRDTLDSAAQMALRIYVE

SGGGFVGIHNAFGTQYNWEWYRGLLGGTQLFDHGPNQPATVTVVNAADAATEGLPASFTVTDEFYNVFPD

PQAVADINVLLAVDDSTRIAGTGGYNGHPGLYDGLHPLSWCHYYDGGRAFLTTLGHTEDIFDDANFRAHL

LGGIESTMGRKPFCQGG*

>gkv_365|gene_NONE|periplasmic binding proteins and sugar binding domain of the LacI family protein

MSYRSRITALDVAQLAGVSRSAVSRTFTEGASVSPQVRARVLAAAETLGYRVNLLAKGLKDQRTSIVGLV

VSDMHHSLRAQLVDALARQLMQAGYRPMLLPVEGEDGMAHALHMMLDYNVAGAILTSDAPPASIAAECAG

HNLPLVVLNRDSAGATYAAIRHDAEAAGALAAETLARAGCKRVIAVRQSRPSFTITWRARAFAARASALG

LDVDELIAGTRHDYDGGRAAGLAFLPTAARFDGVHCGNDFSALGFLDALRGHISVPGDLAVIGCDDIAEG

AWRAYNLTTIRHSMRDLASAAVATLTAQLTGAPPQRQQILPVSLVPRGTTPPL*

>gkv_366|gene_NONE|tat (twin-arginine translocation) pathway signal sequence domain protein

LWQLCEELSCRALWAVAKPPLHTLSKIFRVCFKSLSQSGGKRRPSDQNRKIILMNRRSFLLRSAAVGAAA

TLPRMAFAQDGTLYVYSSSDSNVVDFWTNVIVPRFAATNPGVQVRVVDAGDNAGLRAIGERALAALSSGA

DPQAEVFESFRPEELTGTIEAGLWVDMTDAGLTNWSRVNPAAMEGNYAAPWRGSQVLLAYDATRLNPEDA

PKTWDALATWIKANPGQFIYNRPDKGGAGGNFVRRAVHEANGRDPSAFTVANYTDAYAADALAKAWDVLL

DIAPSLYDGGSYSAGNTQSIQMLAQGVVTMTPVWSDQVLSAIAQGVLPETTGLVQLQDLALCGGFSRATV

LANGAQKEAALKLVDFLLSEDIQSAVLTELGGFPGVSWDYVDPALRAQFADVIPTSIPTFPGGAWEVAIN

DGWYRTVAPNVARD*

>gkv_367|gene_NONE|binding-protein-dependent transport system inner membrane component family protein

MPKTAGGRGLIGLLLVAVPVGLLAWLIIFPIFNALWRTMVVDGGVSVETYAFFFTDAYSLRNLWLTIWVT

AVCAALLMAICLPIALYLRFAKSRVAAVVQGLALFPLFVPSVILAYALIRTIGPNGAVDILLVNMGLPKI

VSPYLTPWGPVIGLVWDNIPLTVLLLVAGLGGVSDNAVEAARDAGAGKIAVFRHVIIPQIGNSVLVVLSF

TVLGIFSAFTFPYVLGGASPEMMGPFMQRTFGQLFDTRMAMTQAVITFAICAVFGAFYVRSIARGQKERR

*

>gkv_368|gene_NONE|binding-protein-dependent transport system inner membrane component family protein

MQKRFDWLGALIATALAVVIVVPLLVVAIWAFTEVWRYPSLLPQQWGLRFWNQTLARADVWEAIWLSVRL

SLTVTFLSAVICLPAAYAFARMDFPGKNIFFLSFLASHAFPKFGLLVAIAGIFLGLGLISNFWGVVLIQL

VGTLMMMIWIPVAAFQNIDRRMEEAARDAGAGPIRVFLQVSLPQAAPTISAAVLLTFVGTFYETEGAWLI

GAPQIRTMPVLMITFINNQIVVQYGAVLSVLLWIPSFVALMFARKLVGSSSFARGFGV*

>gkv_369|gene_NONE|ABC transporter family protein

MSSLTLQGLRKGFGGQDVVKSVSLEVADGELVCLLGPSGSGKSTILRMIGGFEAPDGGAVLIDGADMTRV

PPEKRPTGMVFQSHALWSHMDVAGNIAFGLRLRGMTRADIAQKVDEVLVLVGLEGYGKRRVWQLSGGQQQ

RVALARSLALEPKILLLDEPFASLDQHLRERLREELRDIQQRLGITTVFVTHGQDEALALADRIVVLKDG

AIEQADTPARIYRQPATRFVAGFIGTMNFVPTHFAANASTHPLFQHCAELPDGPAMLAIRPEAMQLHPGA

GGRVHRVTDFGTHLLAEVGLDDGPRVKVICPPDAPWQAGDVVRPAAAEIAVFHG*

>gkv_371|gene_NONE|glycerophosphoryl diester phosphodiesterase

MAEPLFIDHAKGRTWLKWHRGRRQAGDMEFAPARILQGMRAGARVEVDLVRHAGGGFAVLHDETLDRGTD

GTGRVDQASADQIRALRRRDDAGGVTDVGVALLGDLCAALAGSLPDTALLQLDMKENADQIGDDDVAAFA

AAVQPVARHIIVSGGDAAMVARLQAAAPAVMTGYDPCHFGALTRLRESRDYAGFVAQALADAGQAQMIYL

DYHAVLQADYDRFDLIAAFGAAGRRVDAYTLQAVTPQTVAIARHLMALGVDQITTDDPLGLWAACQA*

>gkv_370|gene_NONE|inositol-1-monophosphatase (IMPase) (Inositol-1-phosphatase) (I-1-Pase)

MMTDPFIPSARLAQLIAAAHAGGAIARAALRRRDASEVVHKAVRDYQTEADVAVERAIVAHLAPHFPDHA

IAGEEGADDRAGSAGRIVIDPIDGTTNFMWGMPHFGVVITLVEGGETVAGVTYDPMMDETFAAERGAGAY

LNGQRLQLSHSLDAINAVFGAGLPIPGQVKSVTEARYHAALRRLMDTSAGVRRLGSSALSIAWVAAGRLD

GFFEDGLSLHDYGASVLILREAGGIVTDFAGGDIKNPGAILAGRPGLHDWLLEGFKA*

>gkv_372|gene_oppF|oppF

MTTPLLSVKNLSKTYRGPGGKDVLAVRGVSFDLAPREILGLVGESGSGKSTTGRLVLRLEDATTGEITFN

GQRIDTLPQRALRPVRREMQVVFQDPYAALNPRMTVGKFVSEPMEIHKITAGRTETRDRVADLFKQVGLD

PSFMDRYPHEFSGGQRQRINIARAISISPKLIVADEPITALDVSIQAQIVNLFSDLQEQLGLAYLFVAHD

LAMVRFLCHRVAVMLRGRIVEIAPTESLFQNAQHPYTQALISAIPIPDPARERARRALSYTPDEAALSGE

LREVSPGHFVLQA*

>gkv_373|gene_NONE|oligopeptide transport ATP-binding protein oppD

MTNRTPLLEVRNLSVAFDTPAGTVHAVNDVSYTLHKGETLGVVGESGSGKSVHAMSMIGLIARPPGRITS

GEVLFRGRDLLKLSEAELRDIRGREIGFIFQDPMTSLNPVLTVERQLIETLTRHLGLTGAQARTRAVELL

KMVRIPDAERRIKSYPHQFSGGMRQRVMIAMGIACGPELVIADEATTALDVTVQAQILDLMKDLRREHDT

TLIWITHDMGVVAGLADTVQVMYGGRILERGPVDAVFASPRNAYTWGLLRSLPTEGGAQKTRLYQIPGSP

PDLTKPPAGDPFAPRNEFATPRCAREMPPLLPAVGSDPNHLVAAWYDLPALIAAKEAE*

>gkv_374|gene_NONE|dipeptide transport system permease protein dppC

MMSFLRALLRSKGGLIGAIILLVLVAVAIGAPLLDLSNPIRGDLRARLVGPTWEGLFAPGAHPFGTDPNG

RDILARVIYGARISLSIALAAVLLGALIGVTLGIVAGFTGGWVDAILMRLVDMQLAFPLVLFALLVVAAL

GPSLTNLIIVLGITSWTQYARIVRGQVLALREREFVLSARAAGAGSLRIMARHILPNVMTPVLVVGTLEL

ARIIVMDAALSFLGLGVQPPTPSWGRMLADGRVYITSAWWVVTFPGLAIALTVLSVNLLGDWLRDYFDPK

ARQ*

>gkv_375|gene_NONE|binding-protein-dependent transport system inner membrane component family protein

MSRYLLRRLIEAVISVWGVLTIVFFVGRLLGDPVSLLVPIGASVADMERLRAGLGLDLPLWQQYLTYMGQ

VLQGDFGTSFVFNQPAMQVVLERMPATAQLAGAALAIGVLIGGTAGIIAALNKGRWPETLVLGFAMIGQA

TPTFWLGIMAILFFAVQLGWVPTGGYGTWQHLLLPAFTVAIFISASIARLLRSSMLDTLREDYVRTARAK

GLMPRTILMWHTLRNALIPVITMIGILVGELLGGAVVTETVFAWPGVGRLIFQAIDQKDFPVIQAGVVLV

ATIFVVANFLVDLLYAVLDPRIKEGRA*

>gkv_376|gene_NONE|oxidoreductase

MMRDISLAALEARLAQDLDRLNFTGKDWVPPTVIDGARVRDVVVIGAGMCGLVTSVALQKHGILNHVVYD

KAPAGLEGPWLTIARMETLRSPKQLTGPAYGLPSLTFRAWYEASYGTEAWDALFRISLEDWMAYLVWYRR

VMHVPVVNGAEMTGVSAGPDGLIALDMVIDGVQTQVLTRKLVLATGRDGLGGPFLPQVARDLPASLRAHS

ADIIDFDALRGKVVGVVGAGASAMDNAATALEHGAKAVHMFIRRTDIPRLNKGMGIGSPGMTHGYLDLDD

MWKWRIQHHLNTSQTPPPRPSTLRVSRHANAYFHLGAPLTSLREDAGRLSLDTPKGHFDLDFMIFATGFG

VDYTQRPELVAVTPHIKLWSDTGFPDTLPADLKDNALASAPDLGPAFELREKTPGACPILNSIYSFNFPS

TLTHGKLSGDIPAVSEGADRLVRGITSALFTADIEQHWTLLETYATPELLGDEWQDADTKEAP*

>gkv_377|gene_NONE|alpha/beta hydrolase fold family protein

MPQVTLDGCTFNYEVAGPEGAPVIFTLHGGRGAGELGNDFRTWGAALSDKFRVISYDQRGHGKTTDTLPF

TFNQLADDIETLRKHFCGDAQCIVIGGSFGGFIALTYALRHPGSYSKLILRGTAPSYHMEAEAIEIMKAR

AHLVPSLTPAMIEKLFSNRVESDLEFRLLWLAMQPLYADDPATFDAQKAFERTRDMPVHLQAHNDLYDDD

QWLAYDVRERLHEITAPTFICVGEDDWICPVSQSRLMAEKIPNATLLVVEGANHSVHAQAPDVVMPAVRE

FLSA*

>gkv_378|gene_NONE|bacterial extracellular solute-binding proteins, family 5 Middle family protein

MTRLWASALSSLALAAAFAAPAAAQDRTLVMALSGDVSTLDPHMTASIGSDLSVASHIYPSLVLRGPDMA

LQANAATSWEATDDYTWVFHLNPEAKFINGEVLDAAAVKANIERVTNPELNSRIASWFTNISEVNVIDAQ

TVEMKTAIPYPALADQLSMFFLLPPQWMNDVNPALETTSGGPYVMTNRVPGSSITLEANADYWGAAPAFD

RVEIRIIPEDAARVAALRAGEIDFADKIPVSEVEGLDADANLTAGSVPATRTTFLKINTEKEPMESLALR

QALNYAVDKEVITEALFNGMASVANCQLLTDQYFGYNPDLEPYPYDPERAIALLEESGIDTSQTIELEVP

VGTYLQGDEVAQAVQMMFTDIGLNIEIVEMSFGAYQDKHIKAHDVGRLSLLSYAWPTIDADGLLGLAEGG

NPYAFWHNDEFDGYLAAGRASTDPAVRQEAYNQATALMCEQAPFVFLYEQPVTYATSDEVTWQARGDDWK

RAMDFVPN*

>gkv_379|gene_NONE|bacterial regulatory helix-turn-helix protein, lysR family protein

MAGNSLFDLRQLEAYAAVISTGSVTGAAKVIGKSQPVVTRLIQDLEADVGFPLFARHGRRITPTQNGSLF

YREVERLLADAHRTRQRAADLSHRQIGSVALAATATMAASIVPRALKGLRDQGYAPKEIVLRSQTSEEVI

QSVAARQADIGIASLPLDHPALEVQWIAEASCVCVLHEDDPLAALDVIDLSALAGRPIITLLNPYRVLGR

ISAALHDIEPESVIRTNSSTTALNMVSAGLGVAILEPVSPIGQRFANTVVRPLSVSIPYYWGIVTPVGLP

ESPIIQPLVEALITASQMDMPGFRIRDVRETESILKNIFTAPE*

>gkv_380|gene_NONE|hypothetical protein

MADLAFPEVPQRASFRDSTDACRASVIQPRDDLGLAPQLRAGLAARMCVLIGQAEMAAAYRAGLDGLALQ

IAQGALPSDLGDAGAAAMARHCDHITSHPADATRGDIDALVAAGLSVPQIIALSELVAFINYETRIRQGH

ALLEGF*

>gkv_381|gene_NONE|uncharacterized peroxidase-related enzyme family protein

MAAFTVKALAWQPYLEPLAMEDATPAQLEAMQVTPSGGKVSPYVRTLAHDPESYAARTVLYNQIMYDEGG

LSNEWRELGALVASAVNKCVYCASVHARRYDRLSGRNDVVKELYTRGMDGTFEPKIRAVIDFSRALTQTP

AGATRAQVQALLDHGFSTSDVSDLIHAVSIFGWANRLMHVLGHAVPAGTATK*

>gkv_382|gene_NONE|hydantoin utilization protein A (ORF2)

MMADKIPSLRVGADTGGTFTDISIYDPATGKLSVWKVSSTNADPSEAIATGIAEGLAAYGRTPDEVAYVG

HGTTVATNALITGKFAKTAMLTTQGFRDVIEIRRQMRPDLYDLQQVKTPPLAPRDLRLELSERVNFDGSV

RILLDEAEVRARAATLRAEGVESVAIGFLFAYLYPDHEETVARILAEELPGVFIASSADVAPEYREFERF

STTVVNAALGPVMKRYLDRLRPRLQAIGVQPEPRLTQSNGGVISAAEAAMYPVRTVLSGPAAGVMGALEI

AGAAGFPDVITFDMGGTSSDVALIDKGRPQLVTDAVVHGHPIKVPMLDIHAVGAGGGSIAWIDAGGHLKV

GPQSAGAVPGPVCYGKGNTEPTVTDANVVLGVLHPTHLLDGRLPIDRQAAVDAIQALADRLGMGLMETAQ

GIIAVATANMAKAIRVVSVERGYDPRDYALMAFGGAGPLHAARLARELDMPRLLVPRNPGIMCSMGLLLT

DLKTHFALGRRIRLDAEGLGAVAESFATLEARAEGWFAQQGIADADRATQRSLDMRYRGQGFELPVDCPA

GPITDATVAALREGFAEAHRQVYGYAFPDEPIDIIALRLAATGHVPRARLSAEEPATGPASAAITGTRDV

WLPERGTFAALPLYDRAQLQPGHVVKGPAILNQMDSTTLVLDGQTVTIDPYLNLVIAEDADEA*

>gkv_383|gene_NONE|hydantoinase B/oxoprolinase family protein

MKLDPITVEVIGNALSTVVEEMGRALMRASYSANIKERGDCSAAVFDTRGRLIAQAQQIPLHMGSLLGIA

EAVLSRTDLSTVVEGDVFIGNDAYTGGGTHLNDIVFFEPVFAQGAIVAWVANIAHHSDFVDRGHAHIFQE

GPRIPPIRLYRAGVIQQDILDFLLLNCQVPEERVSDFRAQMASNRLGVQRMQALYTRYGAATVDAASAQL

LDYSERMARAGISAIPDGRYAFSHDFDTSLWPELLNLSVAIEIKGDEALFDFTGCPPQTRSGMNMVFTAL

QACVYFVVKTLIDPATPANAGFHRALSITAPAGSVVNATAPAAVYSRHDISQRLIDMMFAALAPVLADRV

PAGSTGVTVQTVSGTNPRNGKFYVYNESMGGGMGARQRLDGLDGVHVNSTNSANIPVEALESEYPLSVDV

YELVQDSGGAGQYRGGMAIRRRISPTGHQATVNLGGPLNRIPAWGLDGGAPGGLARIELGEGVKPLSARN

GMLEDGQKAAAVTSGGGGFGDPRQRDRDLVRRDLREGRISEAAARDIYGLDI*

>gkv_384|gene_NONE|peptidase family M20/M25/M40 family protein

MTTTVTTVLNDPRFKAAEAALQQGYTRFVDELITLTEIPAPPFKEQRRAAAYLKMMIDAGLDDAFIDDIG

NVCGVLRGGHNAGHVAVAAHLDTVFPEGTDVTVRREGTRLFAPGVGDDTRGLATNLNMIRALIAAGIRPA

RSILFVGDVGEEGKGDLRGIRHLFEQGPFKGQIEAFVGVDGDQTARIVNQAVGSLRYRVTFRGPGGHSFG

AFGVVNPAYAMAAVLTGLSQLHLPTEPKTTATPSVFGGGTSVNATPEAVWFELDLRSVSATELTRIDRAF

RALLDQAVAAENARGQTHAGVISVELQNIGNRPAGLCDPASDIVAASLEAARAYGFDAELRASSTDANIP

ISLGVPAVCMGHGADDNTRAHSLEENIDVAEDKVMRSQSAILATILAVAGIAE*

>gkv_385|gene_NONE|bacterial regulatory proteins, luxR family protein

MSNRPTAVYFLTAGQSPRPDLIADVTRALPLPIEAHEIGALDGLSPAAIAALAPTEDEAQIMTFDAGGQW

ITLSKPRLAARMAAALAQVPVDQRPLVVILSTGLLGDFDTPFPTVNAQRALESTISALAEMGEQIGIIQP

LPHQAAYEAIPALSSYKVEKIAARMGDRTALEQAGRALSHCAFIVLNAVSYSAADADIVRAASGRRVLLA

RKIVASALQLLLSTRRAPGSDLAPDLLSGLTPRQRQVLLLMPEGLSSKLIARRLGISPKTVEIHRSQIMR

RLNVRSAHELIFLIAAQRSAEGGI*

>gkv_386|gene_NONE|conserved hypothetical protein

MTRLFTADEIDPLAMGAWILGTGGGGNPYLAQLNLHQLYRHGKTVSLIDPSELADDDMVAVVSKMGAPLV

GQERLVDPEHLARAVRAMEEYLGKTFAAVMSVEIGGGNALSPFLAAAHLGIPVVDADAMGRAYPEAQMTS

FAIGDLKMYPLSLVDCRGSEAIVTKVPTWKWMERISRAMVTEVGSTSATCKAPRTGAEVKEWGVLNTVTK

AIGLGRAVMAAQAAHTDPVQAVLDYEGGIRMFTGKIADIDRTTTGGFLRGAAKIEGLDGDAGDVMELAFQ

NEWTVAYRNGTPVASTPDLLCLLDTVSGEAIGTETVRYGQRVTVVALPAPAVLCTERGLQNVGPRAFGYD

MDFKSVFDT*

>gkv_387|gene_NONE|hydantoinase/oxoprolinase family protein

MKRIGIDVGGTNTDAVLITGERVLSFIKQPTTADVMTGVVNAIKAVMAADPEPHIAIDAVMIGTTHFTNA

VVERARLERAAAVRIAMPASASLPPMVDWPQDLFDAVAPLRFMVEGGHEYDGRPLVPFNREQMFDAAYQI

RDAGITSVGITGLFSPLTAEGEAEAAAVIRTVIPDARITLSHTLGRIGLLERENVTMLNAALQGLGASTV

DAFRKALVECGITARFYLTQNDGTVVLADVAAANPVYSFASGPTNSMRGAAFLTGRLDGMVVDVGGTTAD

IGYLQGGFPRQANNVVKVGGVRTLFRMPDLLPIALGGGTIINPETLAIGPRSVGYNLLRDAMVFGGSILT

ATDIAVAAGLAEIGDRDRVKGLDKALVDGALAGMRKLLEENVDRMKTSAEGTPLLAVGGGAFLIPDELRG

CSEVVRVEHAGVANAIGAAMAQVSGEVDQVFSGLDRDAALAEAERLARAQAVAAGAGEETISVIDAEDIP

IAYLPGKARRVRLRVVGDIDFGGK*

>gkv_388|gene_NONE|hlyD family secretion family protein

MKRSVILPTAIVGALGLAGVLAVLFAWHLPPFSPALPSTENAYLRGKVTSLAPQLSGYISEVPVTDFQEV

HAGDPIAVIDDRTYRERLAQAEATRAGADAALSVAQQNVRSAEATLHAKEAALVAAQIAVDTAQSARDRT

SELRTRGVATDASIEQADLSLQNAVAQYSQAEATVAVQREQLAGAIAQISTAEANIASAVAAVALAQLDL

EHTVIRAPADGYLGQVSARVGQYVSAGTALVPHVGKDLWVIANFNEGNLSAIHLGQHVTFSVDAMGGQNF

AGTVESFSPAAASEFSLMQGTNATGNFTKIPQRVPVRITIDPDQAGTQNLVPGLSVNAKIQPL*

>gkv_389|gene_NONE|major facilitator superfamily MFS_1

MPAIFAGMSGKAAIPYLIAGVLIALAQGLGQGFITANLPQIAGDLGVSTTDATWLMVAYMTPRAALPLLL

IKIRTQYGLRRFTEVSVIAFALVAVAALFTDDMDSAMLVQFLSGCAAAPLSTLAFLYMIEPFSPMFKLKL

GMPLAMTMIMIGPNFARVVSPSLIGDGGLFGIHLASLGLALMCLAVVWFLPLQPQPREKVLKTLDFVSFA

LISAGFTGLISCATLGPTYFWDQAAWIGWVLAGSVGLLAAAVMVELTRDDPMIDFRWIASPAILHLTITL

LLFRLILSEQSAGAPRMFQVLGVGPGQMVDLFSVICWATLLGGLIAIIWIKPKQEAAMHATALVLIACAA

FLDSQSTIDTRPAQFMFSQALIAIGSMLFMPPAMMMGLMSALARGPQYILSFIIVFIATQSLGAVMGSGL

FTTLINHRQAFHLATLNEQLVPTDPQVQAAIAAGARMFAATSADQAANQSNAVAQLATQANQQAYVLAYN

DAYFIIGLIACAALAALILHSLRDWIWRKIDPSVIPAGPPGPPSGPPTPPAAAPADAPTPSRT*

>gkv_390|gene_NONE|marR family protein

MPSDPEILDQLLRLMRSLRRNFDIAAAELGLTSARARVISALSHMEGATQAELAQKLEIEAPTLKRQVDA

LEDLGFIERRGVDGDARKRALFLTDLARSSSITRLVREMRRGVLTGIPPEDRDTLSRALDIMAQNAARIS

EDGIK*

>gkv_391|gene_NONE|carboxylesterase family protein

MSKTSVIAQTEAGQIVGYFKTPDVIHFRGVPYAAAPYGDLRFKAPQRHPGWEGFRDATNTGPTSPQNEPS

FRGPGFNYRAVFSPGWVRGTEILNLNIWTRDMNARAPVMVYIHGGAFDHGNGAVPMYDGTRFAEDGVVLV

TINYRLGLEGFLKLEGGDANNAIRDQIAALEWVQRNIANFGGDAGNVTIFGESAGAASVNLLLTAPAAKD

LFHKAISQSGLAPSAPKAALADAVAREVSSALGIAPTVDAFRDISQQALLDVVAEVARNASDPLIASEGT

LIARPYGDGDVLPLDVRGAFSAGASVGKQVIWGFNTDEATLFTVPNGLHAKATEADVTAFAARVSKNPES

LLAFVKKTLGAHATPGQIRDRMQTWSMFGGGTVWAAGEHAALGGQGWLYEFTWKSPQLGGMIGASHLVEL

PFVFDILNHPNIPAMLGDAAPQSLADDMHSRWVGFAKTGDPGWPTFDRADALSIRFDAPAQLDSHRHDEE

AAYWPQG*

>gkv_392|gene_NONE|glutathione import ATP-binding protein gsiA . domain protein

MSDTLLQVDNLVVEYPGSGFFAKPARALHGVSLDIQRGETVGLVGESGSGKTTLGRAILGLADVHSGTIS

YGGKDISRASRGQRRALSRDIQVIFQDPYSSLNPVMSIEDILIEPLRVHRIGTAEDARAKAARLLERVNM

PRSALSRLPREFSGGQRQRIAIARSLMVDPKLIICDEPVSALDVTTQERVTSLLADIQRETGVAYLFIAH

DLALVRQISRRVAVMYHGEIVEWGDAEVIGARPQNPYTKRLLLAAPVPDPARQRIRRARRLDLAAQA*

>gkv_393|gene_NONE|ABC transporter family protein

MTITTTNTTERPIIKRKSYLRDVLTRPTAIIGLSWIVIVAVAAVFATQLSPYSPIENNLRATFQLPTLAH

PLGTDQLGRDILSRLMHGAAEALIGSLVAVTVAVMIGLPLGLLAGYYGRWVNLITSRLADLLLTIPTIIV

LLAVLAVFGNNMYFAMIALGTMLSAGFMRLATSTTQGVARELYVDAARVFGVRDLRILIRHVLPNMIGPI

VVQTSMMLGIALLLQSGLSFLGLGARPPFPSWGQMVAEASLQVYTQPWMMVPAGLAIALTTLALNFIGDA

ARDALPQAQRGNLLASSASAPRPAQDQTPKADDIALSVRDLHVGFPDGKGGTHPLVRGVSFDVKRGTTLG

LVGESGSGKTITALSILGLLPTPLCITQGSIILDQANLAGADEAGYKGIRGRRIALVSQEPMNALDPCFK

VKTHLRGPLMRFRGLSRRAADAEALELLKTVGMRDPKKVYESYPHQLSGGMAQRVSIAWALAGQPDILIA

DEPTTALDVTVEAGILDLLRELQSTFDMSIVLVTHDLGVVADICTEVVVMRDGQIMEHAPVDQIFTAPQH

PYTQSLLQHARALERDLGQD*

>gkv_394|gene_NONE|binding-protein-dependent transport system inner membrane component family protein

MLWFVAKRLLTAIPTLLIVATLTFLLVYMLPGDVAQTILGDQATPDQVARLRGELGLDRPFFAQYVSYLG

QLAQLEFGRSLINNQDVLYAIGQRSTVTLTLAIGATVFSAVVGIALGMWAALKGGAVDRSLRTLTSIGMA

IPSFWAGLLLVLLFAVTLMWLPANGYTPITRNPGMWAMSLILPIVAISIAAISTLTRQTRASFQEVLGKD

FIRSLRASGLPVGVIAFKHGLRNAAIPVITVIGLQFVALLGGAVITETVFALPGIGQLVVSAVQQRDLPV

VQGVVIYITVIVLLVNLCLDLAQGWLNPKMRLS*

>gkv_395|gene_NONE|bacterial extracellular solute-binding proteins, family 5 Middle family protein

MRLSLISRRSLMVAALLLGTTALVACKTEDTASADENRTITVGVQRPPNSYDPLLSVWGGQYQMYLLPVY

EPLIRQNTDGTYGPALAVEFGYSDEARREYTLTLREGVVFSDGTTPVTADAVKQNLDRLLTVSGPQTREL

SDSLAGVTAPDARTVVISLNQANPDLERIISQLSGMIVNPAALAEGSDLATNPAGAGPYVLDAANTIVND

TYVYTKNPHYYDPDAYPYATITMKVYGDQNAMLTALQSGVAQLGYGGPDNVEVARRAGLQVAEQPTNVFH

IVLHDRDGALAPALADQRVRQALNFAVDREAILASVYRGEGALTTQIFGPNTEAYDAALNDLYPYDPDRA

RALLRDAGYPDGFTFSVAMFFPQRDGDYAQAIAAYLAEIGVTMQINSLAGTTSDPSIMRQNGGFVNGFGG

QGAFTDSKTLFLSPGTIFNAFGSVDPELNALWEAAANQTSDAARQQGFRDLGRAVVEKAWFLPTTVVNAV

AYYREDALSDVTFTPGVTVPLFYELRNKAE*

>gkv_396|gene_NONE|carbohydrate kinase, thermoresistant glucokinase family protein

MAEPQHFVVFGVAGAGKTTVAHQLADATGRIFADADDFHTPEAVQKMSSGIALTTEDRLPWLARIRDWMD

MQAANGQRTSVACSALRRDYRDILRGKPGDVRFIFLSGTQDLIGSRLAARAHHYMPSSLLASQFQTLEPL

FPDENGVTIDVSVPVTDIVDQLRRSDRLAG*

>gkv_397|gene_NONE|bacterial regulatory proteins, gntR family protein

MIGVNRIWCWEDALSRQTIYDDLLDQLGQRICGGVYAAHEVLRTDDLAAEFDLSRTVIREALKVIESMGL

IRARRSLGLVVMPQADWHVFDPRVIRWRMAGPDRMNQLRSLTQLRQTIEPVATRFAALHASDAQRQQIVE

LADLMMETGEKGDLAAFLAHDIDFHALLLEASGNEMFRAISTSVAAVLKGRTEHKLMPTKPKPESLALHK

LVAVSVANGDSATAEAAMQTLMNEVRTTLDGI*

>gkv_398|gene_eda|KHG/KDPG aldolase|

VTPAEQSAAAAKICALAPVVPVLIVENTADAKPLASALVAGGLPALEVTLRTPAALDVIRAMAEVEGGVV

GAGTLLTPADVKAAKAAGAKFGVSPGATQALIDACAEYELPLLPGAATASEVMFLLEQGFTVQKFFPAEY

AGGVPLLSAWSSPLPQVKFCPTGGVSPSNAVKYLSLPNVICVGGSWVAPKSAVASGNWAEITRLAADARL

IAGV*

>gkv_399|gene_edd|phosphogluconate dehydratase|

MQRPIHDTINRVTDRILQRSEGVRSAYLDKIGKAASAGPARAHLACGNQAHAYAAMGQDKVSLAAGRAPN

IGIITAYNDMLSAHQPFETYPELIRATARTVGATAQVAGGVPAMCDGVTQGQPGMELSLFSRDVIAMAAG

VGLSHNCYDTSLYLGVCDKIVPGLIMAASTFGHIPAVFVPAGPMASGITNSEKAAVRNAFAEGKATREEL

MASEMASYHGPGTCTFYGTANTNQMLMEVMGLHLPGATFINPGQPIRDALTAAAVRRAVEITALGNDFRP

IGEILDARAYVNGIVGLMATGGSTNLVLHLPAMARASGVLLDLQDFADIAAVVPLMARVYPNGLADVNHF

HAAGGLQFLIGELLDAGLLHNDVQTIMGEGLDAYRTEPKMIDGALHWVDGPKTTLNEKILRPAADPFAAT

GGLRQLSGNLGRGVIKISAVAPDRHVIEAPARVFHDQHDVKAAFQRGEFTSDTIVVVRFQGPRANGMPEL

HTLTPVLSVLQDRGLRVALVTDGRMSGASGKVPAAIHISPEAAAGGTLALLQDGDMLRLDATNGTLECLT

DLAGRKPAPVDLEASASGMGRELFAAFRAQVGGAETGAAVVV*

>gkv_400|gene_NONE|conserved hypothetical protein

MKSLLSGASLALVMLAPPLAAQPAYTSNVPSLQITHLQTLPARESPATAEYCGDLQAETSAAAHVAAQGW

APTAEAELGPLTLVSFVGQQVQALSGTCELLEGNVGIFYGDELLGLIYGTDASLPQIGTITRFDENTIRI

WDGDLLPQPVADLHLAEGGALTLQPPAREQSYCDGTATIPLLYNQQISKARQLLMANGWQPVATTNQVDS

FAQTLAAAGIPEVDSCAGTGFGFCAFNYQTQGATAFVVTAGEGSEGGTPIVVRYGVTCTN*

>gkv_401|gene_NONE|substrate binding domain of ABC-type glycine betaine transport system family protein

MTKLVYASVLAIAASAMAAPAFAQDNQECGRVTIAEMNWASAGVAAWVDKIVLEEGFGCQVELVTGDTMP

TFTSMNERGQPDVAPELWINAVKDPLEAAVAEGRLITLSEILTDGGVEGWWVPAYFAEANPEITSVEQAL

QHPDLFPAPENAARGGIYTCPAGWACQITTENLFRALDAEAQGFDLVETGSAAGLDGSIGNAYARGEPWL

GYYWAPTAVLGMYDMVMLPFEVDVDEQHFVECISVVGCPDPQVTAYPVAEVFTVVTQQMAENNPVAVGYL

ETRAWGNDTVNAILAWQMENQATNEDAAWWFFENFPEVWGAWLSDDARTSVESAI*

>gkv_402|gene_NONE|glycine betaine/L-proline transport system permease protein proW

MASSCWGLPDFLCGAPEMSGSAMRAMRRTIDTGFRDMVRGASSYIDAATAPVQSFLNTLEGLFVNTPWIV

VFLVLVGITWGTSRSWKITAGAGVALIGIGWFGLWNDSMITLSMVTVCTLVAVIIGLPIGILTARSDRAQ

RIITPVLDVMQTMPSFVYLIPVVVIFGIGKVPGMIAVVIYAMPPMIRLTNLGIRLVDREAIEAADAFGSS

ERQKLWNVQLPLALPTLMTGVNQTIMMALSMVVVASMVGVGGLGRNVLQAINNQFFTIGFLNGFALVAIA

ITFDRASQAYGKRLQRHKEVSDE*

>gkv_403|gene_NONE|ABC transporter family protein

MSDQFLAPDDEELTPGISIRNLYKIFGPDAAKHVEKVKDGMTKAELNQKHRHSLGLRDINTDLPAGKISV

IMGLSGSGKSTLLRHLNGLIMPTAGEVLIDGQDVAKMTPSELRVFRRNKTAMVFQNFALLPHRTVMDNAI

YGLDIQGIKRADSIARARHWIERVGLKGYEDRYPSQLSGGMQQRVGLARALTNDAPILLMDEAFSALDPL

IRVDMQSVLLDIQKEVSKTIVFITHDLDEALRLGDKIVILRAGEVSQQGKGEEIVLHPANDYVNAFVREV

NRGRVIRLRSVLKPKPAERDWPQLQLPGNTTLEEAALLLLPAPESFATVLGRNGQPRGIVNMDDIMHGML

VRSA*

>gkv_404|gene_NONE|peptidase family M20/M25/M40 family protein

MDKIFDYIDAREAEYVTRVMDYVRHPSISAHNIGIRHVAGLLVDMLDGLGFQTQLIETAGHPFVFGEYLV

DPSLPTVLLYGHYDVQPPDPLEKWISPPFEPTIRDGRIWARGIGDNKGQHFAQLMGIEALLAVTGTLPCN

VKFLLEGEEEIGSPQIADFVAAHREMLACDLVITSDGPLHDTGLPQVTYGVRGMASFELRAKTADRDSHS

GNYGGTMPNAVWTLVNLLATMKTPDGEITIAGLHDPIIPATNAERAAVGALPLDVDGFMAELGLKHLDAP

ANVPFYDRLMFRPTLTINGLHGGYGGEGTKSVIPSEAFAKCDIRLVEAMTPDQVFDCVRAHVAIHAPDVE

FIAHGGMLPSKTSLESPYARAITDGIRAARGVEPLHIPSAGGSLPDYVFTKILGVPAFVVPYANHDEANH

APNENLKLDLFHAGIRTGAAMLTKIGGQ*

>gkv_405|gene_NONE|hydantoinase/oxoprolinase family protein

MNSSDLATDYTVAVDIGGTFTDISLLNRVTGQRWRAKTPSVPSDPSEAFLRGITIALEQAGAPAAALGRV

LHGTTVATNMILEGKGAPIAFLTTEGFRHVLAIGRQDIPRKANLYTWVKPKPPVPASRIFEVRERLAPGG

DVLVALDEETVVAAARAIAKLPVQAVGVSLLHAFANPAHERRVAEILREHLPDLYITISSDVLPVVREYE

RGLTTVLNASVMPGVTTYIARLEDRLTSANAPAPLMLMQSNGGIAGAAKIRTAPALTALSGPAAGVVGAR

VMAEACGIRDIITCDIGGTSADICLIKDGHIGLTQSGAVGDWPLALPIVDMITIGAGGGSIAKVDAGRLS

VGPQSAGARPGPAGYGHGGTLPTVTDAHIVLGHLPASLLGGTMHLDREAAQRVIRDSVATPLGLSMEQAA

RGILTIADNNMVGAMRVISVERGHDPRSFALVPFGGAGPLHGCSLAETLGTDTVVIPPSPGVLCADGLMV

ADLKSEFSRALGRPGAVDPAECDPIIAELSAQADAWFATENVAPDLRNARTVALMRFKGQGGEIAVPYTS

DGKVTEAAFIAAHTEMYGFALQAPVELVTLRVECTGTTIQPPESLLPAGTEVPVDQLSPVWIDGAEVQVP

VISRTRLGAGAVFDGPAILTQLDTTTYVKPGWRGTVDASGALILRKA*

>gkv_406|gene_NONE|hydantoinase B/oxoprolinase family protein

MTDQHTLIDIDVPAGTCDPITLEIVRGAIVATQKEMEALIERTAISAFIREKKDFYTALFDDKGKMAVGS

MVPIFGDLTTPVIEKFPRASMRPGDLYWYNDCYGSKGAVTHSNDQVLLAPVFHDNRLCAFVMCWAHFADI

GGIYPGSISPDATSIYQEGIIVPPTKLVDAGVVNEMALAIFHRNSRFPAQSEGDLSALMAAVALGSVRVA

EIVALRGADVVQDALAQLLERNRLMVRGKLAETFDYGTYKFTDSIDTDGHGNGPFRITFSLTRERDADGA

DIFTFDASESDDQAPGPVNLLMNKGVPGMALGLYYLGGDPSQVCNAGGPLSLDNVIYREGSIVQPRFPAP

LGMRGLTMMRTLAVINGLVNVAGGGAPASHSAYVINIMRGTYTNPAGESEPFLLADGIGVGYGARPNADG

IDAVYFVAQENYPVEFLEMGYPVRLLRYGVLPDSGGPGKFRGGVGIVREYEILADEAQIAIRIDGVQNPP

WGIGGGMSGGVGAATVNPGQANERKLPPLSEGTKLHYGDILRIETGGGGGYGHPHDRAPADVLEDVLGGF

VTAEAAEKYYGVKITDDAVDDAATAALRAMRPATRAFHRKEYLDELV*

>gkv_407|gene_NONE|oligopeptide/dipeptide ABC transporter, ATP-binding protein, C-terminal domain protein

MTTQSDTLVSVENVSKSFTLSRSLSDVFNGRHPKVDAVSEISFTLKRGETIGIVGESGCGKTTLGRMLLK

LITPTGGAIRFDGADINALSGESELAFRRRAQLVFQNPFDALNPHFTIRRSLYEPLLNTRVPAAEHDRMV

GEAMVRVQLDRMLHLLDSYPHQLSGGQLQRVVLARALVLQPDFIVADEPVSMLDVSVRAGILNVLREVRD

TMGLAAVFISHDLALVRYVCERTITMYLGAVVEDGPTKDVIAHPLHPYTRALVQAVPVPNVDQSHDPLPL

IGAMPDARNPPAGCRFSDRCPLADDGCRAARPPLRPVGPGRRVACFKVPAFTTAQVQ*

>gkv_408|gene_NONE|dipeptide transport ATP-binding protein dppD

MLLSVKDLKIHYQSPRGTVQAVDGISFDVPEGKVVGVVGESGCGKTTAVRALTRVMPSVAKFAGGEVLFD

GKDLLSLSESQMNALRWRDISYIPQSAMNALDPVWRVGDQIIEVLVKRGGMKKPAARTRAEELFAMVGLE

RKRLRDYPHQFSGGMRQRASIALALALNPRLVIADEPVTALDVIIQRQVLDTFLRLQRETGISVVMVTHD

ISVVAYVCDYVVVMYAGKVVEKGPVRDVLTQPSHAYTIGLYNAFPELSEDAARELSPIEGGPPLLLNPPT

GCRFRARCPFALPVCAQEPPEVQVGAGHTAMCHRAHEAEALRAAARDPKIWQGVVTQ*

>gkv_409|gene_NONE|binding-protein-dependent transport system inner membrane component family protein

LQPAIENAPRKALPDLSQGKLNSGLWPRFMRGINTFLSVPRQNAYAMIGLIIYAIFILTAVFADVLMTHR

PLEILFTDSYQLARNIAPGPEHLLGTTYGGRDIYSQLVIGTRSALLVGVTAAVCVVAIGTVVGLISGYFG

GWADNLLMRLSDIALGIPFLPFVIVLASFLGASQMNVIIGIALLLWPNSARVIRSQVMSLRERAFIEAAR

VTGAGQWKILFVHIAPNVLSLAFLYTSVAVGWAILTEASVSFLGFGPSNTVSWGYMLQDAYASQALGRGQ

YNWFVPPGLCIVLVVMAGFFISRGFEEVLFPKLKG*

>gkv_410|gene_NONE|binding-protein-dependent transport system inner membrane component family protein

MKYMLSRLFYSLVVLWIVATILFFMFRLMPGTPLAAFIDNTTTKEQQDAIIAQFGLDRSLFEQYLSFLAN

LAQGDLGQSFFHKRPVMDVVMEALPNTIILTMTGLVVAYIFGVLAGAYLAWKRGSVAEAIGIPITLATRA

APEFWLGMVLLAIFSFQLGWFPAGGANSVGMTYDNQWQRVFSWDFLRHLALPALTLAIYLQGLPLLLMRS

NMIEILQEEFITMGKMKGLTPRTIVLNHAARNALLPVATAFALGVGGAIGGNVVVETIFSWPGIGRLLVN

AVSASDYPLAQGAFLLITFVLIAMNFIADMTYHLLDPRIRLAASN*

>gkv_411|gene_NONE|tat (twin-arginine translocation) pathway signal sequence domain protein

MSITRRALLRGSAYAALGAGLSTTLPRIALAQDTSAIRPITLYSRAQAANPQQYQSAELIAQAWSDLGLQ

VTVNGLPQNQLSDIVWYNRNSWDVTMWQMVGRPERSDPDDFVMNLFHSSNIETGYNFVGYNNPDYDAAAE

QQRLAVDRDARHALIIKAQELVNADQPYSFLVYPTKSYAFDKTVLDGATMVNQPGLGIRNFLSYVAVEPL

GAQRDLITNTANELNAINPFYISGGGDSWITECIWDRVMRIGEDGLPQPWAAESVVWDEAGTTATITLRD

GMTFHDGQPVRIEDVIFSLEAPQGENVAPMYRPFVTPITAMAKVDDRTMTLTLDQPNAAFETSTLSKMNI

VPEHVWSPLLERLSAGETAESVLEESRIGSGPFKFDRWNQSEVVLSSVKDHFAAPKMDRVIMRVVLNVEA

ALGMLRSGELNFLTDYTGDPQLLLDAAAADGDIEVVDVVDMGFQFLGYNLRRAPFSDPAFRRALSFAINR

RLILGAAYNGFGVPANSHVSPALPFWYDTRTEEIPVGPEVAIQMLEEAGYSIVNGRLHYPAGQTETLQ*

>gkv_412|gene_NONE|hypothetical protein

MLDKEFSIIEQVVPEHAWDLVTTFAGRERWRPEDVNSAGELIGARLRALGLPVVIHRPIVALSIPLSASV

TLDGEEMFAKPPSSSKSCPEGVSAELFYIPANKSSLRSYNKKAIAFFGEGVGTDDEMRARVAGKILITEG

FGNPALAAIAMEWGAVGLIAVNPGVDTHWGTCTTVWGSPDLDDFGRKPTIPVIAVNKLVGGKLIERAKAG

ATTATIKTEMQEGWFEQAIPTVEIPGTVEAEKFVFLHGHYDSWDVGVGDNATGDAAMLEIARALWENRDK

LYRSVRIAWWPGHSTGRYAGSTWYSDAFALDLDENCVMQINCDSPGCRWATSYHATTAMPETFALVKDVI

EKVAGQTPIFDRPHQAGDYSFNNIGISSFFMLSSTMPDDLRAEKGYYAVSGCGGNIAWHTENDTMDIADK

DILMTDIKIYLDAIWRVANTPLLPVDWRETTKEFAASAATYTKAAGDAVDLSAVVSSIKALDDQLAAFYA

AIAAGAIATPDANEVLMRLARILIPVNYTTAPRFTHDPAITRAPLPLIEKVIDLPHVPEALYPITVNQII

RGANRTAGAMREATRLIKTVM*

>gkv_413|gene_NONE|hydantoinase B/oxoprolinase family protein

MIDDIRLQVIWSRLISVADEIATTLERTAFSLIVRDNQDYACALYDSRGVMLAQSSQCTPGQAGSTPTVI

AEMLATYPPETLEDGDILICNDPWVGAGHAPDVFVATPVFHRGALIGFACTCAHHADMGGRLGATDAREV

YEEGVIIPVSKLYRAGIKNDELHRLLARNVRMAEKVLGDINAQIAANRVGAKGLCQLLDDFALPDLHALA

DLITNRTEDMFRRALARLPEGSADSEVFHELRDASGARLRINLRMQVHAGAVHLDFTGTSAQVDMPVNAV

LNITRAYCLFPFIATLCPDLPMNAGAFRPVVLHVPEGTVLNPTFPAPGMYRSLLSYFTVEALMGALYKIA

PDLAMAPSGTYPLWTEKFSGTADDGTKFLSHYNAQGGQGAFRDRDGVSAVVFPGNIATTSVELFELEAPF

RILSRELRPDSGGPGKYRGGLGQETVMECLNATAVQVAFSGGRLVEPALGREGADAGAKGVIRIGDEAPF

ERSGRGVMQKGDIVTFSQPGGGGFGPPDQRDPAAIAHDIAMGYVTEAAAKAVYTYQPE*

>gkv_414|gene_NONE|hydantoinase/oxoprolinase family protein

MTTPDYRIGCDVGGSFTDFILFDAATGALETLKVATTPAAPEQGIMQGLSQLAARHPDLAAHLNTFIHGT

TLVINAILERKGAKTALLTTEGFRDIIETRREIRYDIYDIRQTYPSPIVPRNLRRPVPERMGHDGQIVAP

LDTAKVRAALMELANEGVESVAVCLINAYANPAHEREIAKIAQDLPLTLSLSLSADILPEVREFERFSTT

ALNAYVKPKVDRYLGALEENLRGDGFAVPVYLMQSGGGIVTAATARAAPVRLAESGPVGGVLAARDLALA

AGYHDAIAFDMGGTTAKTCLIRKGEMPVTRAYEVDRVHRFKRGSGTPLAVPTVDLIEIGAGGGSIAHIDG

LGRLCVGPESAVADPGPACFGRGGALPTVTDANLLLGYLDPAEFAAGGIRLDRAAAEAAVAALAVPLGLS

VLGTAAAMIEVVNENMSQAARIYAAENGGDLTRSTMVAFGGGGPLHGAEVARRLRVPRILVPEAAGVFSA

MGFLMATPRYEVARSHPRRLSATTAPELQAILDDLLAQARSVVAAAAPDAPQGASLFADLRYFGQGHQLR

VPLDDLSHGAITAAFRAAYLQSYGYAYDDMEVELVTLRAEVHASSTGPTFRPLAGIGAVRPTRLAWDPIA

RAMVPHQVVAFATMKGQITGPALINQPGATIHVSQGATATRNPAGWLDISLPQEVPHD*

>gkv_415|gene_NONE|peptidase family M20/M25/M40 family protein

MTDDTLLDAIRHWVEVETPTGHVAGLTRLVQMVAADYAAIGAETEIIAGTGGQGPHLIARLNAGTSNAGI

LILSHLDTVHPVGTLADFPFQIKGDRAFGPGIYDMKAGAYIAMQAAGAAARSGTLSLPITHLFVSDEEIG

SPTSQALIEDLARRSKYVLVTEPAREGGQIVIARKGVFRYRADAFGRPAHSGARHQDGRSAIAEIARLTL

AFEALTDYDSGTTVNVGMIGGGTAANVVPAHAYAEIDLRVDNLAAARAVEDFVAGYRPHDPDVRLQITGG

LNRPPYETSPAIQDLFTTAATIAQDIGFTLKGLKTGGGSDGNFTAALAPTLDGLGADGAGGHTLEEYIRV

SSLTERLRLLQGLMERLT*

>gkv_416|gene_NONE|peptidase family M20/M25/M40 family protein

MTPSRAAAVLAAQTLADDGRFLETLRARVAIATESQNAERLPDLYRYLHDEIGPAVAKMGFDYRVVDNPI

AGGGPFLIATRIEDAALPTILGYGHGDVVRGIPAQWREGLDPWTITVEGERWYGRGTADNKSQHAIWLAA

LGEVLAARGSLGFNAKFIVETSEEIGSVGLDLLLEAEAEALSCDLLLASDGPRMLRDKVDIKLGNRGAYA

FDLSVKLREGSRHSGHWGGVLEDPGLILAHALASITTPRGRILIDDWLPKSVPPRVTAALQAIHVDPSDP

TLIMPEDWGEARLSRGEKMYGWTSFIVLAYVTGTPEKPINGVQPEAYARCQLRYTVDVDEARFMPALREH

LDRHGFHQVEITNLPLNRFPAWRTDPDNPWVDRVLDSIAATIGYAPTLMPNSSGGLPSEIFARHLNCPVI

WIPHSYGGCKQHGPDEHVLQPLMREGLGIMAGLFWDLGARDDG*

>gkv_417|gene_NONE|bacterial transcriptional regulator family protein

LSYTISAVDRALQLLEALADSPESGISELAERTGFTKSLIFRLLYTLEERGFVSKDPVRRTYSLSWQAVL

LGAKARRQSRLISAATPHMVALQNATECNVLVQVRDDLHSVTVAMLHTRASHSVFGDVGRQGPLHAGAGP

KILLAYAPEAIRTRVLASALPRYTENTVIDPERLNASLDIIRRDGWVITEGELDHSTCSVAVPLFNGAGE

AIATMAVNGPVALLPPEKRGAVLDALRDAARQIAGLIGNYGSPPERDEI*

>gkv_418|gene_NONE|hypothetical protein

MLSSNELNRVTDIAWTLVNQFASQDREVPVEANRGAQIIADHLRAAGLPVQMHQPELYLSLPKTAQVQIV

GGKTLRAKPPAFTLHCPQGITGDLIYLPDAGGGTPLDRSPAALGALAAARGKIAVIEGFALPNFIAGLEA

AGAIGAIVVNPGVDIHWGTVSTIWGTPELPDLARLPKIPSVAVNQPDGQALIALAQAGGQVVIRTELETG

WFPQYLPVVEIPGKDSEDFVLLHGHYDSWREGVGDNGTGNACMLAVALALWQQRGTLKRGVRIAWWPGHS

TGRYGGSAWFADTFARDLDRHCVAHLNCDSPGCRWATSYAEIACTAEAAPYVSQVVADVAGQVACGKRPQ

RNSDYTFNNIGVTGLFNASSNLPPEVLAEKGYYVVGGCGGNIAWHTENDTIEIADRDVLQKDIALYFAAV

AGLAQADQLPFDWRLSAAEYVETIAQYQAACAFDLTPAAQAAGTLAAAIDALYAQDLPPAVFNDKVMALS

RLLIPAHFTRGPRFSHDPALNVPPLPAIADAMLWHQIPADQQGFLRAQLMRGQNRLVDVFARAAQAVAL*

>gkv_420|gene_NONE|zinc-binding dehydrogenase family protein

VMRAVGYRRLGPAAEVLHLEIRPIPEAPGAGEVIIRLHASGINPHDTKARAGWTGGTPPEDFFIPHSDGA

GVVTAVGAGVDLAIGARVWVFGAPHGAGTAADYLRIPAWRVLPLPDALDFAEGASLGVPLLTAWLAVLAD

GPVMDQVLLVQGGGGAVGEAAVALGVAFGARVVATARSADSAARAAARGALAVRAPDDPALSQFIADMTG

GAGVPRVVEVDFGANQTRNLAMLADHGTLASYSATSDKFPTLDYYGFARKGARISFVQGMKLTPAHIVRA

RADLLPLLAQGQLRPTIAARFALDEAAAAHEMVERGAGGNVVLMLN*

>gkv_419|gene_NONE|uracil-DNA glycosylase

VTYAVRLPTRDVFAAWRSAARRAISHRIAPADLDWAPQDGLFSARALPDDDGPHQARVSKDFLRFAKSVL

WHRAPERFDLMYQALWRLDSGAGDPTSQADPLGRRLQLMAKSVGRDIHKMHAFVRFRELPAPGPRRRFAA

WFEPEHLTLEPASSFFAKRFADMDWAIFTPDLSAHFTDGALDFHQGQPRPDLPDDASEALWGTYFTNIFN

PARIKLQAMRSEMPKKYWHNMPETRLIPAMLRDAERRVRQMHEAAGSMPDLGAQNISARYRAAMPAAPDL

PETLEQAEAAAAQCRRCNLCEAATQTVWGRGAPDAALMIVGEQPGDHEDLAGVPFVGPAGQVLHRAMAEA

RLATDQVWLTNAVKHFKFAPRGKQRMHKSPDGPEIAQCRWWLGLELAFIKPRLTVALGATAAAALTGDTS

PLAARRGAIETGLHGGPVLIAWHPSYILRLPHVADKDRVYSELSHDLQHAHQLIQH*

>gkv_421|gene_NONE|radical SAM superfamily protein

MSLNEKLAILSDAAKYDASCASSGGEKRDAKKGGLGSSGGAGICHAYTPDGRCISLLKILMTNFCIFDCA

YCINRVSSNVQRARFSVEEVVTLTLEFYRRNYIEGLFLSSGIIRSPDQTMADMVRIARTLRQDHGFRGYI

HLKTIPDAAPELIHEAGLWADRLSINVELPQDSTLRQLAPEKRPETIRTAMAQVRLAGEAAKDKTHKGRA

ARRFAPAGQSTQMIIGADGANDVTILNSASRLYTGYHLRRVYYSAFSPIPDASAALPLVQPPLLREHRLY

QADWLLRFYGFTAEEIATGATGGHLDLDLDPKLAWALQHRGLFPLDVNRASREMLLRVPGFGTRTVDRII

ATRRTHTLRYEDLVRMGALMKKAQAFVSLPGWSPRALTDQADLRARFAPPPQQLQLL*

>gkv_422|gene_NONE|ABC transporter family protein

MTPLLRLDHISKSFGPTAAVHDLSLDLHAGEILALIGPSGCGKTTTLRLIAGFEVPGSGQILRAGQPITA

LPPERRGIGIVFQDYALFPHLSVAQNILFGAPDADLAALCALVGLTGLEQRFPDQLSGGQQQRVALARTL

AVKPDIILLDEPFSNLDASLRQRARAEMRRLLKETGCAILLVTHDQEEALGFADRVAVMQAGRLHQIDTA

RAVYDHPISAIAARALGPVMLIDGIADGDACATPLGPCRLASPAQGPVTLALRPHHILPDPNGTPARLIT

STFAGGQCHHSLQVADIDLQMTLSAAYPLPDPLFIALDQMHRFSPLSPDQYQGN*

>gkv_423|gene_NONE|binding-protein-dependent transport system inner membrane component family protein

VRKRRPPLILVLPAVILAVGTCLPVIYLLIRAFGAGPDQIAQIILRPRILLLLGNTLQLVALVLLLGTLI

ALPMAWLVTQTNLRGRRIATWLMVLPLAVPGYVMAYALIGMSGYYGFLSHWFGITLPPLRGLWGAGLALT

LYTFPYIFLHLRAAFLGMDGSLVESARSLGLSPLQAFLRIILPQLWPALVSSWLIVGLYVIGDFGAIALM

RYEVFSYAIYTQYAGAFDRTTAAWLALILLGLTLIALWAQARVTRNRRFSSGTLRRAQALRLGRWQASAW

GAIALIALCSLGLPLLVLGHWMRLGLPDFNAALLVRAMAATALLAAPAAAIAVALALPVSLLALRYPGWL

PRLTGRLAYLGYATPPLPLALAMVFMVLALVPMLYQSHAVLIFAYVISFAAIALGPLRLALMQIPARQEE

AARALGKTAPRAFARVTLPRLRRPALAAGVLVFVMIVKDLPLAYMLAPTGTRTLAMSIFAWTSEGMMANA

APFALLLLIFCACFVGLFLKYESGAARR*

>gkv_424|gene_NONE|bacterial extracellular solute-binding family protein

MMKNAYLAGVAFALGFASVATMASAQSLTIYSGRGEALIGPLVAQFEAETGIDAEVRYGSTAEMAALLME

EGENTPADLFWAQDAGALGALAPHFSDLAAGVNETVLPVFRDPSNKWVATSGRTRVLVYSTDRVEEGALP

AAITDLTDEAYRGRVAWAPTNGSFQAFVTAFRLTHGDDAAKAWLEGMIANETKVYRNNGTQIEGIANGEV

DFGLVNNYYLGRYTAADAEYPVDQTHFGAGDIGNLVLVAGAGVIDVSDNKDNAQAFIDFLLSPAAQQYIT

LSGNEYPVREGIIPQATLEPLATVQEISPAVNVNDIGDLEGTLSLLRDVGLL*

>gkv_425|gene_NONE|response regulator

MIRVLIVDDHPIFRSGLSLSLGESPDLQICGEADTAAAALTLARDLRPDVVLLDLSLPGGGLNVLPDLVA

LDGVNVAVLTASEACDDVMAAINTGARGYILKGIGGAPLIDAVRSIAAGEGYITPTLAARILTEIRVEER

LSPQRSASLDLLTPREAEVLNMVAAGHSNKEIARLGDMQEKTVKHHMTRILHKLGARNRTEAALLLRDGT

RSDQSRI*

>gkv_426|gene_NONE|histidine kinase-, DNA gyrase B-, and HSP90-like ATPase family protein

VQETLNNGAHHAPGARQSLRAAAAKGGLQVDTLDDGPGFDLNAATDGLGLRGLRERVLGLGGRFTLHSTA

KGGTSVSMWLPQMQKGAE*

>gkv_427|gene_NONE|two-component sensor histidine kinase

LRRGWLTIRRDARHDRPNRERPIMRAKNDNSSPLMRQFLLLAGVVLTLGMTGTGLWVSAQIERIVVTNSG

AITALYVDAMIAPVAQKLAQVDDLTPEDQTRLDQIVRQGGLSRAVSTFNLWDRHGRILYSTRPERIGQIR

QDNPRLNTALSGRVYASLRNVTQTDGTREQLAEVYSPIHSSRTGQIIAVAEFYTDSADLREDLWQSRLAS

WIVVALVTLAMFAALCTIVLRGDRTIRQQRARLADQVQVLSHSLAANAALTQQIEQANRRIAEINDLTLQ

RLSAELHDGPAQHLAFAAMRLDGVAGQEPVALAVNEALRELRYICRGLVLPELGDLDGETIIHRATATHL

ARTDAPVDVQIAGPLPPLACTRRTACIVSCKKR*

>gkv_428|gene_NONE|mmgE/PrpD family protein

MTTVIQNIAAWCGAQTDFSAAAKRLAAEAITDTLACIVAGRADSATLAVRRAMESQIGASGARIVGGGRA

APAVAALINGTAAHALDFDDNFRPGMSHASAVIVPALLAIADLVDASGEDFVKAYLIALQAQAYVGWGVG

YNHYVAGWHGTSTIGSVGTAAGTAWLLGLDTDGIARALTLGVSMASGVKGQFGTSAKPFHAGMAARNAVE

AALLAQAGLSGKMDILEGEQGLRELYSGGIITDFWDKTPIDGPHVIETTGVVPKRHPCCGSTHMVIDAIL

DLQAKHGFTADDIARVDTLVGVANWRNLAYPAPVDEMQARFSMQYCVARALRKGVLSLSDFTQSAVDQYA

QDPLMAVITMEHYGEENSKKALHPHKVTLWLKNGGQLEGARSVAKGNLGDAFNDADRMEKFVDCCTGLTN

ASVDGLFVTCETIALQRDLKAIDPLFNA*

>gkv_429|gene_NONE|beta-lactamase family protein

MTPCTNPNGQWQSDDSAFSPAAKAAITAVADSQNTDAALVIIGGKIVYAYGDTTRKYLCHSIRKSFLAAL

MGQDVADGTIDLNATMKDLNIDDNDGLSDVELQAQVYDLLTARSGVYHAAGYETKWMQRIKEKRHSHAPG

TFWCYNNWDFNALGTVFVQQTGLSVADAFQQRIAGPVGMEDFSLAGDTPDAWRESFQQSRHDAYPFRMSS

RDLARFGQLYLQGGRWSDQQILPEGWAEECVMLYSHAGARGGYGYMFWLERDGVFAPGVKTPKGSYSANG

AGGHYCMVVPEHDMVIIHRVDTETPGTELSKFGFGKFLKAVFAATAA*

>gkv_430|gene_oppF|oppF

MSDQNFVNVTGMTISFGTGSKRRQVVRDVSFQIPKGGAYGLIGESGCGKSTILRALAGLNTDFSGTFQFD

GETVGNKRGKPFFSRVQMVFQDPYASLHPRKLVQDVLTEPLKVHGFDNIHQRIDDVLRAVGLGPQFRFRY

PHELSGGQRQRIAIARALIIEPTLMLLDEPTSALDVSVQAEILNLLKTLREERGLTYLMVSHDLAVIAHI

CERAGIMQHGRVLEEMTADDIRAGRATADYTRTFLQASLDAIPSQE*

>gkv_431|gene_NONE|putative peptide ABC transporter ATP-binding protein y4tR

MSDNLLHVRNLRVSFKTARGPVDVVRGISFDIGREKVGIVGESGSGKSLTGRSMLKLLPKTATITADKLE

FMGEDLQGASERRMRQIRGRHISMILQDPKFSLNPLVKIGPQIVEAYRVHHKVAGAAAKARAIEMLESVH

IRDPEHVFNLYPHEVSGGMGQRVMIAMMLIPEPDLIIADEPTSALDVTVRRQVLSILDELVTRRGTGLMF

ISHDLNMVASFCDRVLVMYAGRIMEDLPARDLHKAQHPYTRALLQSLPRLDKPTDRLVVPTRDPAWLTEP

TLQTKFEPSA*

>gkv_432|gene_NONE|dipeptide transport system permease protein dppC

MTSTPDTSNPTGLRAWLHAPVPQTARQARAQHIWRKIKQMLANPSGMIGLGVLLVIIMAALLAPMIAPYS

PFSQNLQARLLPPSAAHWLGTDELGRDVLSRLLYGSRITLYMAFLTAAIVGPVGLIIGTVSGYFGGLVDT

VLMRVVDLFMAFPSLILAMAFSAALGPGIENAVIAISLAAWPPIARLARAETLMIRNSDYVAAVRLQATP

AWRIIIGHIAPMCIPSVIVRVTLNMAAIILTAAGLGFLGLGAQPPSPEWGAMLSVGRDFMFTHWWIAAIP

GCAILITSLAFNLLGDGLRDVLDPRNV*

>gkv_433|gene_NONE|dipeptide transport system permease protein dppB

MTDLPVTTDDERRADRRAFVTRALRLIISVAVTLLGLITLTFFIGRLLPLDPVLAILGDNVSQEAYDRMR

TQLGLDQPIWVQYWMYLTKALTLDFGMSLTSSRPVVEDIARVFPATIELATVAIFIGTFLGIPAGVLAAM

YRGSIFDHMIRLFSLVIYSMPNFWLGLMGLMVFYAGLGWVAGPGRIGFVYEFSVAPGTGFLLIDTALQGN

WDAFKNVFSHIILPASLLGFGAMAYISRMTRSFMTEQLSQEYIITARVKGLSWARTVWGHAFRNIAVQLA

TVVALSYAFLLEGAVLIETVFVWPGFGRYLTTALMAGDMNAVVGCTLVVGIIFVVINLICDLLYRVLDPR

TR*

>gkv_434|gene_NONE|bacterial extracellular solute-binding proteins, family 5 Middle family protein

MKKTSFLRNSALTLALAATVSAPALAQTPPNALVMAWNIDAISTFDPAQIAEVVTDEIYRNACESLVDFD

VADESNYVPALAESWETSEDGLTMTFHLREGLTFRDGSPATAGDLAWSMQRVVRLGFGNAATLIEYGFTA

ANIEQTITAPDDRTLVLTLDRPYPASLILGSIAANRVASLLDRKVVEANATGDDLGNAYLATRTECVGPY

NLVRWNPGEVVMLQANETAGGSRTPGLDQILIRHVAETGSQRLLIERGDVDVARNLNAEDLADLEQNENI

KIETVLKPSMFYLAMNMDHPALNDSRVRLAMRYLIDYQGLGDTVMRGVGVPRASYVQYPAVGALDLEEGQ

PFSLDLDKARELLTEAGYPDGFTATLLIGSHPYGSPIGQALQETAAQVGITLNMERMSNSQLFSRTRGRE

FETALLGFGASIPDAHMMSSRMVFNPDNAAEANLGQYPSWRSAYFDEDANARVEEAMMERDPARRIELYH

ALQRDQMENGPLAFIMQTMDPAALRTTITAYPRNGFRVYYDAVTK*

>gkv_435|gene_NONE|aminobenzoyl-glutamate utilization protein B

MKNSDIIWDYVDAKGAEFSEFSDVIWDMPEIAYTEYRSVAEHKKMLEAQGFSITENVADIPTAIMGEAGT

EGPVIAILGEYDALPGLSQEAGVAEYRPIPGNGHGHGCGHNMLGSAALLAATAIKDWLATSGFKGRVRYY

GCPAEEGGAAKSFMARAGAFADVDIAISWHPASFTRVDEALSLANTRMDFAFTGRASHAAAAPHLGRSAL

DAVELMNVGVNYLREHVPADSRIHYAMLDSGGIAPNVVQAKAKVRYAIRSTTLPAMFHLLERVKKVAKGA

AMMTETEVEITTISAVSNLLGNGPLEQAMQGCMDRLGGVPFDEADRAFAREIQATLLDQDIENDFLRVGI

ETDPALPLHEGVIPFDQRGEPMIGSTDLGDISWVVPTVQARVATHAIGTPGHSWQITAQGKMPAAHKGMI

YAAKVMAGTGVEMLLDADQLAAAKADHARRLAKTPYTCPIPTEVNPPLQPRPADLG*

>gkv_436|gene_NONE|prolyl oligopeptidase family protein

VADTVIKTDTPPGMTSFGITALTAMREARAEAVSTDGASLVYLSNASGTNQIWSQSLAGGPALRLTDLAE

RVSSFAFNPKSNDILIVTDTGGDERFQFLLLRAGADAPVALTAAPTVVHQWGAWSPDGSQIAYSSNARAP

HLMDVHLMDVASGAVTTLLEGNGFIEAIAFTPDGSALILRDSARGMGDQDLLLLDIATRACAAIMPHDGP

ARYMNARLRKDGATVFLLCDQGSDFHQVQQRDLASGALVSCIAADGHDIDAYALTPDQQQAACAINIDGA

TQLVLSDLNGDNRIEVALPFIGCVNSLRFTPDGAALLMSLDSTTHSCDVWQYTLASGTFTQLTDAPKGGI

SAASLIAPVLERFTSFDGLSVPALVYRPAGTPPAKGWPVLFLVHGGPEGQWSHNWRPDVQHHLSQGVMVV

APNVRGSTGYGRSYHASDDREKRYDSVADLNAIADAIAARPDVDASRIGVQGQSYGGFMVLAALTTRPDL

WKCGIDLYGISNFTTMMQTTGPWRKVLRAVEYGTDAALLDSLSPIHKMDQIRAPLLLVHCHEDPRVAMEQ

SEQVYSTLRGLGKPVEILRVAAEGHGFARRENRIHAFSTIAAFVQRNL*

>gkv_437|gene_NONE|bacterial regulatory helix-turn-helix protein, lysR family protein

MESKWLEDFLALALHKNFCRAAEARNITQSALSRRIKLLEGWMGAPLIERRTNPISLTEAGEKFLPRAEE

MYNMILSVRDELRAPYMAAQEVLTVCMMSTLSITTFPRLVAQLEQRGEQFRFRFADSRTTMPDWVELLRS

GNADFLLTYAHSSVAVLNTLNEFEYLTVGTERCIPVSVPGTGGRPRYDLTQQDRPVDYLSYRNHSFFANA

LPGIIKRGGFRLNTVYENALSAALLAAVRVGLGVAWIPEKLLEDDLATGRLLRAATPEYDLLVDVRLYRP

MDDGSRIKNRFWRKLGELSPLQ*

>gkv_438|gene_NONE|beta-lactamase family protein

MAMLNWDAAAEMARNAAGWEADQPGGAIILFDTAGLRDSAVAGVESLATMSPISVDSIMRYASVTKHVFA

SFVLAHPAVISLDDPLSKHLPQLNAVTGAVTVGRALDMSGGIPDTREALSLLGLSMFNQTRAPQLLDFHA

AMPRLNYETGTEVHYSNGGYRLVEEAMRSHGLLFDDFLRATLREKHDLPLHASEMWTDPVRNLSTGYWHD

GTGWKMGLQGMHLSAAGSLSGSGRALAEWGRLLLRGEGDFAGRLAALSAPRYLTDGRPTGYGLGLRQQAV

GALTLVGHGGSQPGYRSYLLLDPATGTGCAIVANRDDVNPTCIATDIMAALLGEKSQSPESTLTPGLYVA

SEGADWLAVSGNSVTRLDDAVSVYPDGTGGVDSLSPTSRLQLRMEGDDIVGLVGHASKRYRPVAASPAPS

GLAGIWRSHAFGAVLEIAGNAVIMGTGPLRRAMPLHHLGGGRFLFTLEDGPSQRKICLHYRGDGRIDLAL

ARARMIEYEKAPA*

>gkv_439|gene_NONE|NAD-dependent malic enzyme (NAD-ME)

MQDEAAQAARQAALNYHEFPRPGKLEIRATKPLANGRDLSRAYSPGVAEACLEIRDDPSTASRYTSRGNL

VAVVSNGTAVLGLGNIGPLASKPVMEGKAVLFKKFANIDCFDIELNESDPEKLADIVCALEPTFGAINLE

DIKAPDCFIVEKLCRERMNIPVFHDDQHGTAIVVGAAATNALLVAGKAFDQIKVVSTGGGAAGIACLEML

VKLGVKRENIYLCDLEGLVYNGRTAQMTPQKQAFAQGDSPATLGDVITGADLFLGLSGPGVLTGEMVAKM

APAPIIFALANPTPEILPDLARAAAPNAIIATGRSDFPNQVNNVLCFPFIFRGALDVGATEINDEMELAC

IAGIAALARATTSAEAAAAYQGEQLTFGPDYLIPKPFDPRLMGVVASAVAQAAMETGVATRPLPDIAAYR

AQLDSSVFKSALLMRPVFEAARQTTRRIVFAEGEDERVLRTAAAMLEETTDVPILIGRPDVVEARCERYG

LPIRPGRDFHIVNPQDDPRYRDYWGTYHELMARRGVSPDTARSIMRTNTTAIAAVMVHRDEADSMICGTY

GQYHQHLDFIRQILARGQLRPVAAMSLMIMEDGPLFIADTQVHSDPTPDQIKETVLGAARHVRRFGLTPK

IALCTQSQFGNMDTDAGRRMRGALEMLDDIAPDFLYEGEMNVDSALDQSLRERQLPNARFEGSANVLVFA

STDAASGVRNILKAKAGGLEVGPILIGMGNRAHVVTPSITTRGLLNMSAIAGTPVGHYG*

>gkv_440|gene_NONE|DEAD/DEAH box helicase family protein

LTHQNIAAPLAAALSQRGYETLTAVQQAVLAPEADGRDLLVSAQTGSGKTVAFGIAVAPDLLGDDNILPL

NTPPVALFIAPTRELALQVAQELTWLYANAGAQIATCVGGMDYRTERRALARLPQIVVGTPGRLRDHIDR

GGLDLSELRVTVLDEADEMLDLGFRDDLQYILQAAPEDRRTLMFSATVPREIEKLARDFQNDALRLETRG

EAKQHNDISYQALSVTMRDRENAIFNMLRFYESRTAIIFCKTRANVNDLLSRMSGRGFRVVALSGELSQQ

ERTNALQALRDGRANVCIATDVAARGIDLPGLELVIHYDLPTNAETLLHRSGRTGRAGAKGVSALIVTPG

DFKKAQRLLSFAKVTAEWGKAPSAEEVSARDDLRMVEHPALTTKLDDMTVANDLLARFGAEQISAAFVQL

WREGRPAPEALTVVNTPTPGAAPAPREPRTSPREFGASVWFSLSAGHVDRAEARWLLPKICDAGGITRDA

IGAIRVRESETFVQIAANEAGRFANVSEIEPGLTLTQIDGEPNLEQRSSGPKKFGAGKPGKFAKGGESWG

DKKQTGWVERKNDGWDDKPAPKKHRKGEFEAGMADAPAAAPRKPRFDDAPAATKTARIKPRWTEEQPSFK

AKRPSAAGAPAKPRDGAKPAFKGKPYAANTTGGAPRKSKFKA*

>gkv_441|gene_NONE|quinoprotein glucose dehydrogenase B (Glucosedehydrogenase B [pyrroloquinoline-quinone]) (Soluble glucosedehydrogenase) (s-GDH)

MLPKSLKHKNGAMRLVAASTLALMIGAGAHAQVNPVEVPVGANETFTSRVLTTGLSNPWEITWGPDNMLW

VTERSSGEVTRVDPNTGEQQVLLTLTDFSVDVQHQGLLGLALHPEFMQESGNDYVYIVYTYNTGTEEAPD

PHQKLVRYAYDAAAQQLVDPVDLVAGIPAGNDHNGGRIKFAPDGQHIFYTLGEQGANFGGNFRRPNHAQL

LPTQEQVDAGDWVAYSGKILRVNLDGTIPEDNPEIEGVRSHIFTYGHRNPQGITFGPDGTIYATEHGPDT

DDELNIIAGGGNYGWPNVAGYRDGKSYVYADWSQAPADQRYTGRAGIPDTVPQFPELEFAPEMVDPLTTY

WTVDNDYDFTANCGWICNPTIAPSSAYYYAAGESGIAAWDNSILIPTLKHGGIYVQHLSDDGQSVDGLPE

LWFSTQNRYRDIEISPDNHVFVATDNFGTSAQKYGETGFTNVLHNPGAILVFSYVGEDAAGQTGMMTAPA

PQTQYTQVPAEGAGAGATEVADVDYDTLFTEGQTLYGSACAACHGAAGQGAQGPTFVGVPDVTGDKDYLA

RTIIHGFGYMPSFATRLDDEEVAAIATFIRNSWGNDEGILTPAEAAATR*

>gkv_442|gene_NONE|PRC-barrel domain protein

MSNPVTSYNPAETSNLLSGKDEVKGTKVYSPAGDDLGHIDDVMIDAASGKVVYGVLEFGGFLGLGSDYHL

IPFGKLRYDHARHGYVTDLTKAQLEGAPAYSDDWRTNRDWQQRNYDHYGLPPYWI*

>gkv_443|gene_NONE|PRC-barrel domain protein

MKNLMLTSAIVLMTAGTAAYAQDATPAPADPAMPAPTDPVTPAPADPTIPAPADPVTPAPADPTIPAPAD

PAIPAPADPMVPAEPPTTAAPMAEGGAVATAEELTGANVQDLEGTSIGSISDLQLNGDAVTGVIINVGGF

LGLGAKPVLIPVEQIDVIRDADGTVLHVEVGMSREQLEAMPEHVTQ*

>gkv_444|gene_NONE|conserved hypothetical protein

VALIAAALGFTGIAGTASSIAQVLFFIFIVFFVVAMIARALRGRPPM*

>gkv_445|gene_NONE|response regulator

MSEPEHTTSTSREAPRVENSMAMRVAAELPFLRRYARALTGSQESGDKYAAVTIEALLEDRSLLDPALSP

RIVLFRTFHSVWQSSGRILADAATTMAEKRALARLAGLTSNTREALLLFTVEEFSREDIAQIMQIDADQA

DALLKTAYVETLDTIKGRVMIIEDEPLIAMDIRAIVEEMGHEVVGVATTRAEAEALGRDAQPDLILSDIN

LADKSSGIDAVNTLLAELGAIPTIFITAFPEKLLTGERPEPAFLITKPFTEERVRSAVSQAMFFASTETL

SS*

>gkv_446|gene_NONE|hypothetical protein

VFWFAHTGTLLFKNLYFHWNQTPVAALGSQEIIQFYCERPMRPTGTVSAQHIE*

>gkv_447|gene_NONE|hypothetical protein

LTDRDAIDHAAALDPALQRQVDENLQLLYRSKLNDDLPDSLQALVKKLLEDGRPS*

>gkv_448|gene_NONE|RNA polymerase sigma factor, sigma-70 family protein

LNAIAKPPQPPKSDVPPLPDPRQEILLHLPALRAFALTLSRDSVLADDLVQETLMKAWSKFHLFTPGTNL

RSWLFTVLRNNMRSMLRKRSREVADVDEKMAARLASKPNQESNLALKEVELALDKLPAEQREVLILVGAM

GFSIEEAAETCGCAPGTIKSRANRGRLALARLLGLAPGEKIDISDQATLAVMSQRP*

>gkv_449|gene_NONE|mgtC family protein

VSPDLQNLWQSEFGDPFLALSIEVVLLRLAFAALLGAAIGFERELHASSAGLRTHMMIALAACLFAIIAD

EIIIRQLDSGQQLNMDPLRLIEAVTAGVAFLAAGSIITSGGRITGLTTGAGMWMAGAIGLACGTGLIPLA

ILASVLALIVLWFLRRLSHAIARGGNQASEENGSEQ*

>gkv_450|gene_NONE|H-NS histone family protein

MTDINLTALSLVELKKLEKDVGKAIASFEDRQIAQARLEAEAVARKFGYSLEVLAGTGSAKKSTPVAPKY

QHPENHALTWSGRGRKPGWLVSLLESGKSIEDFAI*

>gkv_451|gene_NONE|NRAMP family transporter

MSEKSTDTPKWSIVGPGLVVAATGVGAADLIATTVAGSLYGYALLWSVIIGCIMKVVLVEGAGRYTLATG

NTIFEGWASLGKWTTVYFGPYIVIWGFVYGAAAMAGTGLALYSLFPQLSVAVWGIISGILGLIMVWSGRY

DRFEKILTFFVLLMFVTMVIAAAFTLPNLGEVLSGLVPIIPEGSMINILSVAGGVGGTITLAAYGYWLRE

KGWETPKFMRVMRIDNQVAYLVTGIFVVATLIVGAELLYSANIAIGGGDQGMVDLANVLASRYGDFMGKL

FLFGFWAAAFSSLIGVWNGVSLMFADFAGQMRKLPHGHIERGSKGKAYKAYILWLTFPPMVMLFLGQPVY

LILAYGVLGALFMPFMSVTLLWILNTDRVPREWRNGPITNILLLLCTLAFAALAINQVWSAIARVF*

>gkv_452|gene_NONE|hypothetical protein

MKNLFAIATATVLAAAVGTSAMAANDFGHIYVSPKIEAGKPLNIDLINVSSPAQLEIYGARGQLVSTQDL

NAGVTTDLRIQSTQVPGGALTAVLKVDGQTVDTQSITRK*

>gkv_453|gene_NONE|uncharacterised protein family (UPF0160) family protein

MTISYLVTHSGSFHADELMSSVVLTRLFPDATILRTRDAQAITPADDRIIYDVGRAYDADQRIFDHHQPD

APRREDDQPYSSFGLIWKHFGADYLRAMAVPEGDIEAIHLSMDRHFALPVDLVDNGALDPATAGALVGLT

LPVLLESLKPVFDNDDPAAEDQAFHAALAIARAFFEASVGRKAAKARAESMVLDAIATAGEGKVLELPRG

MPFRSAIDQAGADHLLFVITPRGTDWSLAGIRKKPDGFEQRADLPAAWAGLNDAALEAASGVKGAKFCHN

GRFIAVADSREAIVAMAEIAVREAAAQV*

>gkv_454|gene_NONE|bacterial regulatory proteins, lacI family protein

MSPKRPTLEDIAKIAGVSRATVSLVVRGSPLVAEATRARVEQIMAQQDYVRDIGAARLRNNSSNTVGVIV

PNLVNAFFTEFLSGVEQVMGTHDRVVLLANSKDSVARQTEILQRFRGHGVDGVILCAAEGTTPDLPERIR

GWGMPVVQALREVGTDVSDYAGADYSEGVCIAMRHLVAMGHRRIAFLSVRARTSAREDRLRGFARGLAET

GAENAGIVEADLAWTGAAAAADAVLALDTKPTAILCFNDVLAAGLMLGLRRAGVSPGKDLAVVGLDDLPL

AEMTYPPLTSIAVSPATIGAGAARLLARRLSQPDAPFERFINTPLLVIRQSVRALTV*

>gkv_455|gene_NONE|amino ABC transporter, permease protein, 3-TM region, His/Glu/Gln/Arg/opine family domain protein

MYQFNFRPVFDNLDMLFYGAWLTVQLSFSAMVLGLIVSILGAVAKTSGGRVLRFIVDIYVEAIRNTPFLI

QIFFIYFGLPAIGISMSPNTAALVALVINVGAYGTEIIRAGIESVPQGQVEAGRALALNKVQIFRYVILK

PALRNIYPSLTSQFIYLMLTSSVVSIISANDLAAAGADLSARTFANFEIYLALTIIYFLLAFGFSTLFGA

LRRTFFNYPASR*

>gkv_456|gene_NONE|amino ABC transporter, permease protein, 3-TM region, His/Glu/Gln/Arg/opine family domain protein

MIREFSQADIFFIVSAIRWTLLLSLIAFVGGAIGGMLIALARTSRNVVLNRFARAFIEVFQGTPLLMQLF

LVYFGLAVVGLPINPLLAAAVALTLHASAYLGEIWRGAIEAVPQGQSEAATALSLSYPDRMRHVILPQAM

RVATAPTVGFLVQLIKGTSLASIIGFTELTRAGQIVNNATFQPFLVFGTVAALYFILCWPLSLLARHMEA

RMRRAITR*

>gkv_457|gene_NONE|bacterial extracellular solute-binding proteins, family 3 family protein

MKTTRRLFTALSAAALAFGMTAPANAADLEAIKNSGTLRVGMMVDFPPFGILDASGQPGGYDADVAKALA

DYLDVTVQIVPVTGPNRIPYLLSGQVDVLVASLGITAERAERVDFSTPYAGIAIGVYGATDVAVTEAADL

SGVTIAVARASTQDTGVTAVAPADAQIRRFDDDASAVQALMSRQVQTIGLSNVVFSQISGVAGGRFDKKF

DLSSQLQGIAVSPDSDALLAEINSFVTHSRTDGTFDALYQTWLGEPLPDFVKNAE*

>gkv_458|gene_NONE|glutamate/glutamine/aspartate/asparagine transport ATP-binding proteinbztD

MSQHAITMRGVNKFYNTYHALVDIDLTVAPGERIVICGPSGSGKSTLIRTINQLEAIQSGEIVVDGVALT

DENVAKVRQEVGMVFQSFNLFPHMTVLENCILAPMKSRKVSRAEAEETARAYLARVRIPEQADKYPAQLS

GGQQQRVAIARALCMKPRIMLFDEPTSALDPEMVKEVLDTMIDLAREGMTMICVTHEMDFARAVADRVIF

MDKGAIVEQNPPEEFFGNPQNERLQTFLGQIA*

>gkv_459|gene_NONE|D-isomer specific 2-hydroxyacid dehydrogenase, NAD binding domain protein

MVLALAALKPADMAQKLHDTYDVVTDIAAASQAQIVLTSGAVGLSPAQMDQLPALRLIAVSGVGVDAIDL

PAAVARGIRVTTTPGVLSLAVAEMALGLALAAGRRIAEGDRFVRAGDWASGRKLALGRSVLAGRAGILGY

GRIGRQLADLLRGLGMPVAYTARSEKNDSPDTYHPDAVTLAQHSDVLFVTAAGGAETRGLVNADVLAALG

PDSILVNVARGPVVDSAALAAALQAGHIAGAGLDVFDDEPNVPQALLDAPNCVLTPHVGSATDEARRAMS

ALVLDNIAAFVAGGPLPSPYGE*

>gkv_460|gene_NONE|2-dehydro-3-deoxygluconokinase (2-keto-3-deoxygluconokinase) (3-deoxy-2-oxo-D-gluconate kinase) (KDG kinase)

MKFLSIGEPLAEFNNPLDAPDQFNRNAGGDTLNTAIYLSRLTPAGSVGYLSRLGDDKMSAFLRGVIVDEG

ITDLCATEIGGRPGLSFITTDANGERSFTYWRDQAPARKLFQSPEDLAVLEQADVLFLSGITLAVLYPEG

RANLLAALAQRKAAGAQVVLDTNYRPRLWPNAETAAAVIGQAAGIATLVLPSLDDMEACFGQPEAEGAMA

LLQGLTDAEIVLTTGGDDVLYRAAGSADVRAIPLPPRRPARDTTGAGDSFNAGWLSSRAAGLSVEAAIAR

AAAVAAEVVTYPGAIMPRAAMPTFEAQN*

>gkv_461|gene_NONE|hydratase/decarboxylase family protein

LTSSFDPALAASLLIAARAGGPRPAALPSVPPDIDAAYQVQHLVLGDGAPAWKMALLRGTARECAAIPAS

DVHVSGAEIALDSDGAIEVETAFILGRAITPGCTPAAALDAVAEVRLAFEFINARLLDRLARDPMEAMAD

SFCSAAIVLGDPIADWAEVLQQPLEIRLDLDGRAVAASEQPQPLAETGPFLVWLADHAAKYGLPLGAGSV

IISGARIGPLQVNGATHAQARIAGATVAAQMSAPPRRS*

>gkv_462|gene_NONE|hypothetical protein

MPMLPHIPIIPGETILSWADRSAQAQAGHCLPELLRVVGFPQKDVEPVKDLAVLARLTDLFGGEAPALEA

SAIFRHDGNMRRFRHEVFKPSMLCRRVTAFCPMCLLDDGSFQHSRVSWQFSSTVCCDRHDIALTTTKAEI

HFDALVPSVAFPDPAALQELCDAATPMTPGPLQRYVEDRLSGSTCEPLWPDGCRLDQVMGISRCLGNAMR

DHTTGDLGDACRTGFEALRGGKASILKALDDILCQRGQKARVNTPTRALGRLYDAVRLNEDLSPLRPLVR

SFLLDNVPLAAGDDIFGEVVPARRRHDLATLSGMCGMSKLQVRDVFAALGLLSTDNGRPLNALTFDAVES

ETVACELRGSVPANALSAHLGCDMTTAAAVLNAGLVERVLGDRIIVDRDADALKRVSLDSIHVLLALLFE

NATTIAVVPVQFATLSAAARHAHWQVDRIIRLVLSGRLDLYGLVGRTDFDGLLIDEPQLLRILEAPPMTA

AMSKEDAANELGVEVQILENIMRIPDESGAPIIRRVVPLTGPLKRRHQVAKPDFEQFRLDHISIRELAEA

EGATLAEMHASLQKRGILPILSGMLLATQVFRRSDI*

>gkv_463|gene_NONE|conserved hypothetical protein

MMNTDTQTRYIAIAPLMAQLRARHVVTERDREVQAQLCRLLHVDRTGQITAEPVRFTAGLESRGIILIEP

AGGGKTTAIRNVLQEFPALGTHPDTGAPRVLEVQVESPATLRSILRALLREMGLTKVSSRTSAWELLDLV

KNRIAVTGATTIWLDEAQDLFLSRSTREIDDMLKMIKGLMKGPNSVVVILSGTERLIEIAGYDPQVNRRF

TKIIPGDLQIGADNAGLTEVICDYCAAAGLTFDGSGSVVSRLIYGSRHRFGRAVETTINAIERALFEGDT

ILGRQHFAEAWGMQEMCAWDRNVFVSPDWHKFELDAQAAEFDASRTARQRRALRQG*

>gkv_464|gene_NONE|integrase core domain protein

MTAIDRHLPQFIIGKHDRITFPTFGMLPGMPGKSMRLVYATDEGVVLQPADGAGPSETFSYAQLRRMNAA

REIHHEPEFYLPVAQRSATLRNELDGIAPAMSDAERRRVDARYALVMAFDVLSDNDAGAARIKRTDESIA

ANMDLIRDTAAEYLSKTGPTPDFVERVREYRENGGRKPQGGQTAARPEAVAPRTLRKWSAAMKVGGKWAL

RDKCCNRGNKNGHFTTDEQGLLSNVIREFYLDLNRPSVAQTVMEVRRVFLEKNAVRREQGLTEMRIPSRD

AVRSFIKGIDELTGLFERYGHREALKRMKPVTRGLEVSRPLERVEMDECKIDLITIMAQAGLLQLFTPEE

LEKMGLNNKKRRWWLVFAIDCRTRMILGMKLTNEPKTTAASECLRMVLEDKGEFADAVGALTPWSHRGKP

ESLVTDNGVFRSIEFTDTCANVGIGLLRTVAGAPGMRGMIERLFRTAIMSLFSRLSGRVFSNVLERGDHP

SEERACLTIEQLAYAIVRWVVDIYHNTPHEGLGGLTPLQQWGRDMEDGNYPLHALPTRATTRIAFGVPIV

RQLSKTGITVGGIRYHDEVLAHRHLSYGDHDVEVRWDHTDIGRIAVCVENRWIEARSVHRNGLLGQALDG

LSAAEWDASVRALRASDPKRQAFDEGVVHAAIKDIQAMNHRQQLEYQVINQDWSKERVLAREEVMMSFDV

RPDTPRTHQTADDYGRTIQPRAPMENVGSEAAALPKPDRGPKFDGKAKG*

>gkv_465|gene_NONE|hypothetical protein

VALPAPSRAGRDIAIASNRHCTAHDVLGHGAGFRIQCESALERDHAYIINARPQTHDLREQVVFPYPFAD

SKTRHIFDFYVTELDGRRIACTVKPDPLTRKRGEGQMPGQDFITHMQTVSFWVQERNFADEVRLLTEKDI

DPVELHNARILAAFREPDPDADAAARELTRYLLGGRSLRDLTRDLGLAERGYRALLRLVRMGALVGQPGH

QLTPDILIFRKGTIQ*

>gkv_466|gene_NONE|hypothetical protein

VDGPLNGPGGPGTACLVTGDSWSFPAINGDIRLSNTLRPIQMDQCIRRKAARAI*

>gkv_467|gene_NONE|hypothetical protein

MANSTNMAVALAAIFGQTLTSVEGLSRALKQAGLRTDGGKGRGASNMVGQDTFNLAIVLAAGVGMTAAPA

FVRNVIDMELKSGVLLQGEQVVWAPGSQPPRGSTLGLVKQDLLPGVEVTSTLGAFMGNWIDGLFDATLPW

KDEGIANLEVGLSGPWADFTLEWAGNRLELGFAAQGVDPRAEPDWERRIILRGNLFRKLVQIVEA*

>gkv_468|gene_NONE|conserved hypothetical protein

MIDRIHLLRNVGQFDNVSPQQQFTPLTLIYGENGRGKTTVAEILRSLATNDPVLVTERQRLGSPHPPHVV

ISHAAGNAIFQNGVWNRPLPDITIFDDAFVSANVCSGIEIQAAHRQGLHELILGAQGVALSTALQAHVLR

IEGHNTALRELADAIPAAARGPYKVDPFCALPPDEAIDAKIQEAERRLAAARASDAIRQRPGFQEFGLPD

FDLDAIDQILGRGLPGLEADAAARVRAHIAKLGQGGESWVADGMPRIERASQGQDGEICPFCAQDLAGSD

IIAHYRAYFSQAYEDLKAVIRQTGIGVRDTHGGDIPSAFERNIRTAAQTHEFWKDFTELPEIEIDTAAIA

RDWSAARDTVLGQLREKAAAPLDQTRLTPEARQAVQSYRARIAEVAALSASVVGANARLDIVKEQAQADD

LAALTSDLAKLNAQKVRFNPAVAQCCDAYLAEKEAKGATELLRTQARTALDQYREQIFPAYEAAINDFLR

RFGASFRLGEVQSVNMRSGSSASYCVVINQQNVNLTAEAGPSFRNTLSAGDRNTLALAFFFASLEQDPNL

ANKIVVIDDPMTSLDEHRTLRTREEILAMGRRVQQVIVLSHSKAFLCHLWEQADRNAAVALRINRAAIGS

DIAGWDVRNDSISEHDKRHELVRGYLRAADPNQERAVATALRPILEAFMRIAYPEYFPPGTLLGPFIRTC

EQRVGGANEILSAADITELNRLKDYANRFHHDSNPAWQVAAINDAELTDFVERTLLFASRR*

>gkv_469|gene_NONE|type III restriction enzyme, res subunit

MSDLHHEKHLESYIVQKLAAQGWLVGDSAGYDPDYALYPDDLEAWLKTTQGPKWDKLAAMNGDKTREVVM

KRLEAALEKDGLMHVLRRGFSIAGCGHLDLSEAAPEDQRNADVLHRYASNRLRVVPQLKYHPGRELAIDL

GFFLNGLPLATVELKTDFTQSIEHAKNQYRNDRLPVDPVSKRKHPLLTFKRGAVVHFAMSDSEIWMTTKL

AGENTFFLPFNQGFDGHGGNPPRPDGEYPVAYFWERICRPDNFLRIFHSFVYVEKKNVVDLKGNWSVKET

LIFPRYHQFDAVNSMIADARAKGPGQAYLCEHSAGSGKTSTIAWTAHDLIKLRKEDGMPIFDAAIIVTDR

NVLDGQLQDAVQQIDHQNGLIAAIDREKSSKSKSDQLTEALTRGTPIIVVTIQTFPFAMEAILTKKSLRD

KNFAVIIDEAHTSQTGNTASKLQATLALSSKKDMADMTVEDILMEIQSSRKRPSNVSHFAFTATPKHSTM

MLFARPADPTRPASDDNLPMAFHKYEMRQAIDEGFILDVLEGYVPYKTAFNLGKEMVDEKRVDGKAAKRA

LAAWMALHPTNVTQKVQFIMEHFSKNVAHRLDGKAKAMVVTSSRAAAVRYKKAFDAFIEVNPAYQEIRAL

VAFSGKLTGKEVMHPNDDLLSNDIFTVDEDVEFTENNMNPGIGGQDLRIAFDRPEYRVMLVANKFQTGFD

QPKLVAMYIDKKIANAVEIVQTLSRLNRTFPGKDQVFIIDFVNDPSSIQAAFAQYDSGAKIEQVQDLNVI

YDIKDRLDAEGVYDNGHVLNFMTARYQTAAAFNTGGQTEHRAMFAATQEPTDTFNERLRGLRDVAQAAEN

AFERASLDGDDAGMKKADHDRARVAEAIGQMMEFKKGLGRFARTYSYIAQLIDLGDPELENFASFAKLLE

NRLNGIPPENVDLRGITLTGYDIKTRPPEGTDGEDGGSGDKKGLILKPEGSGGAARPGSVPVYIQEIISK

LNSIFGEATPLNDQFSLVNQIVAIVRENAVVMAQVERNDKTSAMKGNLPGAVEAAIARAMSSHATLATLL

LKSDRQAIKLLIPMIYDLLKQGGQIELGQ*

>gkv_470|gene_NONE|conserv

MSTAESFGRTIQLFLVDGKPTGLRKATIHGWTGLLFVSGASAFGDLTKRIEVDRTGVYILAGPDTDNLGV

TRVYIGSGNAVSERIEQSAKKRDFWETAIAVTTSDDDLSKGHAEYLECRLIALAAQAGRVVLDNGTQPAS

GRRRLPEADVANMEQFLANLRIILPVIGVDMLKPQPKAVTQMATPPEARTEAEVHFEIRHKSGVRATAVE

EEGEFVVLEGSEALTGTGYVQQSYGGLKEKLIADGVLVPSGEGRMRFSCPWSFNSPSAAAAVVLDRNSNG

RLEWKVRGSSQNYHDWQQAQAAVQGESA*

>gkv_471|gene_NONE|restriction modification system DNA specificity domain

MPRAEWGFVGSIKVPTPPLEEQTAIAIFLDRETARIDGLIKKKGRFIELLKEKRAALITHAVTKGIDAGV

PMKDSGQDWLGQIPEHWDTVPPTALFTESKERAHEGDQMLSATQKYGVIPLEEFEALEQRQVTMAVTNLD

KRKHTEIGDFVISMRSMDGGLERARAVGSVRSSYSVLRCGPEVEGRFFGYLLKSSLYIQALRLTTSFIRD

GQDMNFSHFRKVKLPRVPVDEQIRIADHIDRETARIDGLVAKTDRSIELLKEKRSTLITAAVTGKIDVRN

AA*

>gkv_472|gene_NONE|N-6 DNA Methylase family protein

LSETQVKNTSLADFIWKNADDLWGNFKHVEFGKIILPFTLLRRLECVLEPTREQVRETVKSLKDSGIDLD

VILRQQTGFPFYNTSNYSLASLGATRTRQNLEDYIAQFSENARVIFEQFDFANTIARMDRAGVLYKICLN

FSAIDLHPDAVPERVMSNVYEHLIRRFGAEVNEAAEDFMTPRDVVHLAIELLLDPDDQLFIENPGLIRTL

YDPTCGTGGFLSDGMEHVRSLQDRYSIAPVIVPYGQELEPETHAVCLAGMLLKTLESDPGRDLSKNIKLG

STLSADKHRGEKFHYCVSNPPFGKKWEMDADAVTREHLEQGFEGRFGPKLPRVSDGSMLFLLHLLSKLED

PIKGGGRAAIVLSGSPLFNGNAGQGESEIRRYLLEQDVVEAIIALPTEIFFRTGIGTYIWILSNKKPKHR

KGMVQLINATGLYEPMRKSEGNKRRRVGEDQTAEIVRMYSEFVQTKESLILQATDFGYRRIRVLRPLRKK

MIISEEGIAALADEKAWEKRSAGQQAGWLGLFRENLGRTESWHWIESFAKNAAKCDDDLGKVDVGLIKAF

QKAFAVHDPDMDPVTDKKGNVIPDDDLTDYENVPLTTDIHDYLASEVLPHAEDAYIDETYRDETDGDIGI

VGYEINFNRHFYEYQPPRKLEDIDAELKAVEAEIAGMLAEVTA*

>gkv_473|gene_NONE|hypothetical protein

MRVGDRIESQNEIVATFGYSLVTVLRTLRDMEDEHIIYRQVGRGSFLVRLPWAESYLRIGWFYNRDRIPG

GIFSNVLYSHVIASLENTIVSDGHAFVLGSFTDRKMPVELWDRLDAVLLFGAPSDMAKEHLPETTSLIAT

MDMMLAQVHIDSHGIDMRPAFDGMLNHLGPGPQKVLYLDGRIDLRYPAIRDQELRSAAAARGHCIETLMV

DMEHPDAAYSALRQAITRFKPDAVCGFLREVWTQELTQQYPDLPIYPVVTRPDARGFSVDARGWTRALAD

RTYARLADRTLPPLDTRFPVRFLR*

>gkv_474|gene_proA|glutamate-5-semialdehyde dehydrogenase|

MQDMQQDYTALIADMGTKARAAARVLATASPTAKAEALTTAAKLLRARTAEIIAANAKDLEFGREKGLSP

AMMDRLALDADRIAAMAAGLDSVAAQKDPVGEVIADWDVPSGLNIRRVRTPIGVIGVIYESRPNVTADAG

ALCLKSGNAVILRGGSESLHSAAAILACLRAGLAAASLPEDAIQMVPVRDRDAVAAMLGAVDFIDVIVPR

GGKGLVGLVQREARVPVFAHLEGICHVYIDGAADPDMARAVLLNAKTRRTGICGSAECVLIDTAFIAKHG

KGLVQDLLDAGVEVRADETLATVPGTVAAKPDDFGQEFLDMIIAARVVSGVDEAIAHIRRYGSGHTESIV

TADPIAVAAFFNQLDSAILMHNASTQFADGAEFGMGAEIGIATGKLHARGPVGAEQLTSFKYLVEGQGTL

RP*

>gkv_475|gene_proB|glutamate 5-kinase (Gamma-glutamyl kinase) (GK)|

LGTLIGARRLVVKIGSALLVDRDTGQLRRAWLESLAADVAALRARGTQVILVSSGSIALGRGVLGLPAGS

LALEQSQAAAAVGQIRLAGAYEEVLAPHGIVTAQVLVTLEDSENRRRYLNSRATMETLLSLGVTPIVNEN

DTVATDEIRYGDNDRLAAQVAVTTGADKLVLLSDVDGFYDDNPHLNPDAKFFPVIEIITPEIEAMAGDGV

SGVSKGGMITKLMAARVATDGGCDMAITLGAPLNPLQKLEEGARATWFLALEDPQIARKRWIGAMKPKGE

VSIDAGAVAALQEGRSLLPAGIRLVNGRFGRGDPVAIVGPAGNRIGIGLTRYTAEEAHMIRGHRSAEIEA

MLGYKGRAAFIHRDDMVL*

>gkv_476|gene_NONE|GTP-binding protein Obg/CgtA

MKFLDLAKVYIRSGAGGNGCVSFRRDKFVEYGGPDGGDGGKGGDVIIEAVEGLNTLIDFRYQQHFFAKNG

QPGMGSQRTGAHGADIILRVPVGTEIIDEDEETLIADLSEVGQRITVAQGGNGGFGNLFFKTSTNQAPRR

ANPGQPAIERTLWLRLKLIADAGLLGMPNAGKSTFLAATSNARPKIADYPFTTLVPNLGVVGVDDVEFVM

ADIPGLIEGASEGRGLGDQFLAHVERCSVLLHLVDGTSEDPVSDYHTIIEELTNYSEDLDSDLINRPRIT

ALNKIDALDEEERAALQAEMEEAVGGPVMLMSGVTREGVVDVLRALRAEIDENRLRFRIAQEPEAPRWEP

*

>gkv_477|gene_NONE|acetyltransferase (GNAT) family protein

MNIATINAQPEIRTSRFTLRPLRMSDTGLISLYAGDLRVATGTRSIPHPYPPGAAAQLVTRANGVKRDED

FWALDASEQGGAEFMGLISLDRMDRGQSEVRYWIAPAFWNTGMASEAVRAIINANPHGATRIFAEAFQDN

PGAARVLTNCGFEYLGDAEAWSVARGGAVPTWTYTLKTGL*

>gkv_478|gene_rpmA|ribosomal protein L27

MATKKAGGSSRNGRDSAGRRLGVKKFGGEAVIAGNIIMRQRGTKMWPGAGVGMGKDHTIFATVDGAVKFH

TGLKGRTFISVLPVAEAAE*

>gkv_479|gene_rplU|ribosomal protein L21

MFAVIKTGGKQYKVQSGDVLRVEKLDAAAGETIQFNTILVVGDQIGAPVVAGAAVQAEVIDQIKGEKTIH

FVRRRRKHSSKRTKGHRQQLTLVRIKDILATGADATGVKAAVGASSKE*

>gkv_480|gene_NONE|hypothetical protein

MWRNLFAATAFVLAAQSASAEVDQAKADALFEALLFPQITDVMARESTDFGLELSEGMTGLPPSAKWQEA

VAAIHDRDWMMSQVRGDWYAALDGVDLDALLTFATTAPSREMLQLELAAREAMLADDVRDEAIEYAAVTI

ARKTPRAELIADLVARGDLVEMNVASALNSNLAFYRGMAEGEGDVTDDIILSEVMAQADDLRAGTVEWLY

AFMLMAYAPVSDEDVQAFVAFGETDAGKALSRATTAAFDPMFEEISFNLGRAAAQLLASEAL*

>gkv_481|gene_uvrA|excinuclease ABC, A subunit

MEQKFIEVRGAREHNLKNVSLDIPRNQLVVMTGLSGSGKSSIAFDTIYAEGQRRYVESLSAYARQFLDMM

EKPDVDHITGLSPAISIEQKTTSKNPRSTVGTVTEIYDYLRLLFARAGTPYSPATGLPIEAQQVQDMVDR

TMAMPEGTRGYLLAPVVRDRKGEYKKEMLELRKQGFQRVKVDGEFYELDTPPTLDKKFRHDIDVVVDRIV

VRAGMETRLAESFRTALNLADGIAVLETAPSEGDPERITFSEKFACPVSGFTIPEIEPRLFSFNTPLGAC

PDCDGLGVELFFDERLIVPDLTLSLLKGAVAPWGKTKSPFWLQTIEGLAKHYAFDAKTPWKDLPESVKQV

VLHGSGTEELAFRYDDGGRSYNVTRTFEGVVPNLQRRYRETDSNWSREDMEQYQNNRPCQTCGGYRLRPE

ALAVRVGDMHVGQVVAQSVKEAYAWVQTVPEALSQQKGQIAVAIIKEIRERLGFLNNVGLEYLTLSRASG

TLSGGESQRIRLASQIGSGLTGVLYVLDEPSIGLHQRDNDRLLDALRNLRDQGNTVLVVEHDEDAIRTAD

YVFDVGPGAGVHGGQIVSQGTPAQIEADPNSLTGQYLSGQRRISVPQKRRKGTGKKLTVVKASGNNLHDV

TVDFPLGKFVCVTGVSGGGKSTLTIETLYKNAAMKLNGAREVPAPCETIKGFEHLDKVIDIDQRAIGRTP

RSNPATYTGAFDQIRQWFAGLPEAKARGYGPGRFSFNVKGGRCEACQGDGLLKIEMHFLADVYVTCETCH

GARYNRETLEVKFKDKSIANVLDMTIEEAADFFSAVPPIRDKMNALCHVGLGYVKVGQQATTLSGGEAQR

VKLAKELSRRSTGRTLYILDEPTTGLHFEDVRKLLDVLHELVDQGNTVVVIEHNLDVVKTADWVIDIGPE

GGTGGGYIVATGTPEQVAEVAESHTGHYLKPLLERGHALEAGHVA*

>gkv_482|gene_NONE|glutathione-dependent formaldehyde-activating enzyme family protein

MTTAHYTGSCQCGAIAYTVDADLDSTVTCNCSRCKRLGSVMTFVPASAFHLTKDGPVTSYKFNKLHIEHT

FCPTCGIQVYARGDSPDGPVVAVNCNTMDDVDPRALKSHFYDGAAM*

>gkv_483|gene_lpdA|dihydrolipoyl dehydrogenase|

MQKFDMIVIGAGPGGYVAAIRGAQLGLKVAVVERAHLGGICLNWGCIPTKALLRSAEVFHLMHRAKEFGL

KAEGLGFDLDAVVQRSRGVAKQLSGGVGHLLKKNKVTVIMGEATIPAKGQVRVTTDKGTEDLTAPAIVLA

TGARARTLPGLEADGDLVWSYREALVAKRMPKNLLVIGSGAIGIEFASFFNTLGAKTTVVEVMDRILPVE

DAEISAFAKKQFVKQGMTIMEKATVKQLDRGKGKVTAHIEANGKVEQLEFDTVISAVGIVGNVEGLGLEA

LGVKIDRTHVVVDEFCRTGVDGLYAIGDIAGAPWLAHKASHEGVMVAELVAGQHPHAVRPESIAGCTYCY

PQVASVGLTEAKAKEKGYEVKVGRFPFIGNGKAIALGEPDGLVKTIFDAKTGELLGAHMVGAEVTELIQG

YVIGRTLETTEAELMETVFPHPTLSEMMHESVLDAYGRAIHF*

>gkv_484|gene_NONE|major Facilitator Superfamily protein

MFRVLIGSWAIFVGIFMLMIGNGMQGTLLGLRGELEGFSTFSMAMVTSAYFVGFMLASTIAPKMIRQVGH

VRVFAALASMISAIFVLYSLWPSPIAWMIERAMIGFCFCGAYIVVESWLNSTVSNENRAQALSLYMWMQM

AGIIMSQVLAASGDVAGFQLFGIASIIISLCFAPMLLTTSRVTPTFDQTKSLPLRRLVQASPLACAGMFL

LGSVFAAQFGMAAIYGVRVGMPVEQITLMIGLTYLAALILQFPVGWLADRMDRRILIGTLAAIGGAAALL

AFLIPGQIWLILVAAAVVGGTSNTVYPVIIAHANDYLEADEMPAASGGLLFINGMGSISGPLILGWMLDN

VGPQGFWLVIAVVMLALGLYAAWRLRRVPNKEIVGEPAAHQHLLAGSSAVAVQVVAEAVAEAASGDAAEN

GLTSN*

>gkv_485|gene_queA|S-adenosylmethionine:tRNA ribosyltransferase-isomerase|

MKLSDFDFTLPEDLIALRPAVPRTAAKLLVARGDTISDLTVAELAAQFNPGDRLVLNDTKVIPARLSGLR

HRGEATARIEVTLLQPTADGAWMALIKPLRKLHEGEDIVFTSRLSATLIGRDGDQAKLRFNLTGDDFDAA

LAEAGQMPLPPYIAARRAADAQDLQDYQTIWADRPGAVAAPTASLHFDAAVMAALAARGVTLTRVTLHVG

AGTFLPVKVDDIAQHKMHAEWGEVTATAAAEIAATKAAGGRVIPVGTTALRLLETAARDTGAIAPWVGET

DIFITPGFNFRVADGLVTNFHLPKSTLMMLVSGLMGADRIRAIYDHAIAQRYRFFSYGDASLLLPKG*

>gkv_486|gene_NONE|hypothetical protein

VAFSLCLLLLGYAIGRDVAAPRWLRDEVASIASEALGEGRITFGGLSVHMQRDLHPVVTISDATVFDGAD

RRIAHLPAVNLHLSPRGLVLRRELLVQRITSAAAEVDLTRNADGALAIDFGMVPQANGLNYTEFASVFQQ

FDEIFEAPIFEALREVDIAGVVLRYTDLRGERSLTLDGGTLRMVLDAQNTRLTADAAVLSGRDYATRLAL

TYQSPRHSPAAVAQMRVTDAAAADLGQEIPGLGVLSLLDAKLSGELQIEMGESGAMTAMSAQLNVPGGAL

LPDVGPPGARFDNLDLALSYDPAAQRVALQHMQVSAPWISYTARGTTYLQDFQAGLPQQIVSQLEMDDIT

VSPPGFYDEPAQLAAAAADFRVTLDPLRIEIAGLSLRDESGGLLQADGTVAISPDGWSLMVDAQMDRLPT

ERLVGLWPQSMRAAPHRWMAANLSGGEAVSPHLSLRKLDDGPFTWALSTGFRALDIRGLPQLPLIEDSEG

FVEITAGRLGIGVTKGTATAPTGGPLDMAGSTFTIADMHGPAVGARLDLQAEGTIPAMLSVLDLPPFGYM

TKANVPVDLADGRAQMQGWVTFPLHTPMPAGSVDFAFSAAMQDVTSDQIVPGKHLTSNVLSVDVDRAHVA

VSGPVQLNGADATARWQHNFGTPGSQVTADVAVSDALLQALAIELPLTTSGAAPGQLQVDIIPGAPPAFS

LTSDLAGLAINAGAFGWQKGAASRGNLQVTGTLSTPAQVSALTLSAPGLDLRGQLAMAEGGGLAEARFDR

IQLNGWLNSAMTLTGQGQGRPLRTALRGGSVRLSGLQSLGGATTGNAGPLALRLDQFEVFDGLVLTDLSG

DFNPTGGYSGRFTARINGGVAVEGAVFTQNGRQGVQITGADAGALLQSLRLSGAARGEGFTLTLVPDGAS

DYLGQFSARGLRILDAPALAQVLDAASVLGLIQQISGQGVVFDTVDATFRVSPGGVTLLSSAAVGPGLGI

SLDGAYSTRDAVLDFQGVVSPVYFLNGIGQVMTRPGEGVFGMTFTLGGTAGQVQVAANPLSLLAPGFLRE

IFRRPVPDMTRESG*

>gkv_487|gene_NONE|putative peroxiredoxin bcp (Thioredoxin reductase)(Bacterioferritin comigratory protein)

MPKDQPMTRLQAGDTAPNFTLPGHDGKTYHLSDFRGQRVVLFFYPADNTPTCTTENAEFATHAEAFAAAG

VQLIGINRDSLAKHTKFAAKLALPFPLLTDEDGAVSGAYDVWQEKSTFGKTYMGILRTTFLIGTDGRLEM

VWPVTRLAGHVDSVLTSCLSKN*

>gkv_488|gene_NONE|peptidase family M23 family protein

LPERRLFIRSDTQTRFIRLRPVTQIIAITGCSLLVGWSVIATSIVLMDAISAGNYRAQAERDKMLYEARL

EALSSERDSRAVEAIAAQERFQAALTQVSQMQTDLLTLEDQRVELESGLAAVHATLRRTVSERDSARSQS

ANLLAQIDGAQDVTSGPSDEELTSTLDMMSNALAETAAHRDASDATAQTAAINAADLELELRLMQERNGE

IFSQLEEAMQVSVEPLSRMFRAAGLDPDDLINQVRRGYSGQGGPLTPLQYSTSGSAINPDVERANRILGA

LDNINMYRTAVDLVPVAQPFRSGAARLTSNFGTRWNRAHEGLDFGMPIGTPVYATAEGTVTFAGWQSGYG

RIVKIRHQFGFETRYAHLNEINVRVGQRVSRGDHIADSGNTGRSTGPHLHYEVRVNGAAQNPLNYIRAGR

DVF*

>gkv_489|gene_NONE|conserved hypothetical protein

MFSKSKINEPGNKTPEQAATPSQPAAATTPAPSYSAAPAVKPKPPASVLSSDLHITGNIRTSGDVQIEGQ

VDGDIRAHLLIIGEGATVRGELIADDIVINGRIVGRVRGLKVRLTSTARVEGDIIHKTIAIESGAHFEGS

VQRHEDPITGESKQKALAKPVDDQGV*

>gkv_490|gene_NONE|hypothetical protein

MPNDYFQSMSGGLESPAAQAVAITTSNTADLSVFPRALYATTAGTVRVTMMEGGAIVTLPILVGVPLPVR

VRRVWTTGTTASGIVGVW*

>gkv_491|gene_NONE|metallo-beta-lactamase superfamily protein

MTTLSPSPEVTVFHDKRTGSLQYVVADPAAKTCVIIDPVYDYDEKSGQTWTENADRILGFVAERGYSVAW

ILDTHPHADHFSAAPYLKEKLGAPMATGAYVTGVQKLWADFYNWPDFPQDGSQWDHLFHAGDTFSVGGLT

GYVMHSPGHTLASITYVIGDAAFIHDTIFQPDSGTARADFPGGSAAALWESMQAILALPPETRLFTGHDY

MPAGRDVTWQSSIAEQLATNVHLSRYKTREDFIAAREARDATLPMPKLILHALQVNINGGRLPAPEANGR

RYLKIPVDLLSRGSGE*

>gkv_492|gene_NONE|lipase

MGIIPLDQVAEDARDMVRLYREAAPTPYADMPLPDARRAYMQSCALNGLPHVPLPQVVDHRIAVSGAEIT

IREYRPIIAGVLPAVLFLHGGGWVLGGLDTHDTICRHLAAQSGAAVFAVDYRLAPEHPFPIPYDDSVAAL

NWLIAQADALAIDPARLAFAGDSAGGNLAAALTNSRIAKPLAQVLLYPVTDLAQRAPSYTRVATGFSLAA

AGMEWFIDSYAPAPQDRSDPRLSPLRGDIAAVPMFILTCGLDPLADEGIAYAEAAAQAGAEVEHIHLPHH

AHGLFTSAGRITTGAVMLERVAQYLAARLAA*

>gkv_493|gene_NONE|cyclophilin type peptidyl-prolyl cis-trans isomerase/CLD family protein

MANPLVLMRTEEGDITLRIYLDKAPISAGNFLQYVDSGALNNQTLFRIVTPHNEEAPRDHYIQALHWGWR

TKGEHDPQPFAKIPLETTRDTGLHHIRGTLAMGRYEPGNSGAEFFIMMNEDPDMDFGGKRQPDGLGFAAF

GQVESGWDVMDRLYARAEADQTHLRTPIAISEVKRIAG*

>gkv_494|gene_NONE|lrgB-like family protein

MIDLFWLTLTVGVFAGAQSLARRSGFHPVVNPVLISIAVIVAILLVTGTDYQTYLDGAQMIAYLLGPATV

AIAVSLFRARRLIRQQALPVLGALAVGAPVGAASGWLIASAMGVDPAMALSFVPKSITAGIAVGVSEAIG

GLPSLTVALAIITGVLGAVVAGPLMNRIGLRDPAARGFAMGVSAHGIATARAMQVSAVAGAFAGLGMALN

GLATAVVVPLVFTLLG*

>gkv_495|gene_NONE|lrgA family protein

VILACQLVGEIVIRLTGWPLSAPVIGIVLLFALLLIQARRTGDAAVEQSETVKVADRLLSVLGLFFVPGG

VGIIVYFDAIAPQLWPMVAALVGSTLITLLVTAAVFAWLSKRGRA*

>gkv_496|gene_mraY|phospho-N-acetylmuramoyl-pentapeptide-transferas e|

MLYWLTALSDGSGPFNLFRYITFRAGGAFFTALLIGFIFGRPLINHLRRVQGKGQPIREDGPAGHFVKAG

TPTMGGLLIIGGVVAATLLWARWDNAFIWVVLFVTLGYAAIGFVDDYAKVSKQTTAGLSGRVRLLLGFLI

AAIAGFWASYYQPDAMANHLAVPFFKSLLLNLGILFIPFAAIVIVGAANAVNLTDGLDGLAIMPVMIAAG

TFGIIAYFVGRADFSTYLDVHYVPGSAEIVVFCAALIGAGLGFLWYNAPPAAVFMGDTGSLALGGALGAI

AIVVKHELVLAVVGGLFVVEAMSVIIQVLYFKRTGKRIFLMAPIHHHFEKKGWSESQIVIRFWIIALVLA

LIGLSTLKLR*

>gkv_497|gene_murF|UDP-N-acetylmuramoyl-tripeptide--D-alanyl-D-alanine ligase family protein|

MSALWTGVEAAAATGGRLTRDFAVTGVSIDTRTLQPGDLFVALAAARDGHDFVASALEKGAAAALVSRVP

EGLPANAPLLIVDDVLAALEAMGRAARARTKAKIVAVTGSVGKTSTKEMLRTVLAAAGRTHASEASYNNH

WGVPLTLARMPADTEYGVLELGMSHPGEIAPLSRMVQPHVGVITTVAAAHFEAFGSIEGIAHEKAAIFEG

LLPRGTAVVNGDLAVSPILIAAAKDKGARVITFGETPRNHHRLTDLRVRAGVTVASGRAWRTPFHMKVAS

AGRHFAVNALAVIAAADALGMDRARALVALAEWAPPAGRGMRQIISLDPLREGRTFELIDDAYNANPTSM

AAALDVLALSEPVDGVGRLARGRRVAVLGDMLELGPDEMALHAALAEDHALTAADLIVCVGTRMKALHAA

LPAERAYWLATPEEILPHITRIADAGDVVLVKGSKGSRVSIIVDALRKLGCAPPETLE*

>gkv_498|gene_murE|UDP-N-acetylmuramyl-tripeptide synthetases family protein|

MPNGQITKSLAELGLHGATGARGSARRVSGIAIDSRDLRPGMLFAALPGTQVHGASFVERALKAGATAIV

TDPAGAAIIGDTLIGQEVGLVVMRDVRAAVAGAAALWFGVQPAMMAAVTGTNGKTSVTSFLRQIWQYAGH

KAINIGTTGVDGDWHFPLKHTTPDALTLQRILSEAAEAGVTHAAMEASSHGIEQRRLEGVMLSVAGFTNF

SQDHLDYHKTFDAYFNAKAGLFSRLLPEDGIAVINIDDPKGADIARVAEDRGQEILGVGKNPGARMRLVA

QRFDATGQDLRFEWRGQAYQQRLDLIGGFQAENVLLAAGLAIASGDDPAHVFAAFPHLTTVRGRMQLAAR

RGNGASVFVDFAHTPDAIETVLQAIRPHVMGRVVAIVGAGGDRDRTKRPLMGAAAARFADMVIVTDDNPR

TEDAATIRDAVMAGAVAEGGCDVREVGDRAEAILRGVDMLEAGDALIICGKGHESGQIVGHDVLPFDDVE

QASIAVAALEGLI*

>gkv_499|gene_NONE|penicillin binding protein transpeptidase domain protein

MIRKPLRPLARILQARESGESTDAIQRENMRLRQEEERDHGRQRAELRLLVLGAAFVMGFVMVGGRMALL

ASTDPGEPTMSSGAGAVITSQRGDIVDRNGRLLATNIETASVYVHPHQLIDPDRAASELARIFPELDEAR

LHQDFNSERRFMWIRRQISPEQRQAVHDIGDPGILFGARETRLYPNGPVAAHIMGGAGFGSEGVASAEVI

GVAGVERFFDARLRDPAMAAEPLQLSIDLTVQAAVEEVLYGGMTMLNAKGASGIIMDAYTGEIIAMASLP

DFDPNDRPRVLTTGDQSDSPLFNRAVQGVYELGSVFKIFAAAQAIELGLVNQDTVLDTRSPLVVGRFRIS

DFDNYGPTNSVHRIIEKSSNVGSARLAMLIGPERQRNFLGSLGFLQPTALELSEAATGVPLLPRTWGELT

SITASYGHGFSSSPVHLAAGYATMVNGGHLVRPTLLHTEEHVQGVPIISPATSQDVREMLRAVVTSGTAS

FADVPGYEVGGKTGSADKPRPTGGYYDDKVIATFAGAFPMNDPRYVFVITLDEASEYIAGMTRRTAGWTT

VPVTAEIIRRIAPLLGVRPAVELLASSGVTAIAR*

>gkv_500|gene_NONE|conserved hypothetical protein

MRALLNIAIALFVMGLAFWAYRENYQTQAAQREVNSLRNQIAATHSRNTMLRAEWAYLNRPDRLSELVAL

NFGELGLLPMMPETFGRIDTIPFPPPTVQPTPQPGGFDILPEANVTEAILP*

>gkv_501|gene_mraW|S-adenosyl-methyltransferase MraW|

MANDAPHVSVLIKPLVRAVAPVQGVWVDGTFGAGGYTRDLLAAGADKVIGIDRDPSVFPLSADWRAAMPD

RVDLRLGTFSEMDEIVGEMVDGVVLDLGVSSMQIDQAERGFSFQKEGPLDMRMGDSGPTAADLCNTLPEE

ELANILYLFGEERASRRIARAIVQARPITRTLELAEIVSSCLPRQKPGQSHPATRSFQALRIAVNDEYRQ

LYDGLAAAERILKPGGYLAVVTFHSVEDRMVKRFMQLRAGAVGGGSRYAPEVQSAPPAFTLVTRKAIQPD

DDEVAANPRSRSALLRVAKRTDAPAEMLDPGEIGMPTLEPAKPRGGRR*

>gkv_502|gene_NONE|mraZ family protein

LVSFTGEYVQKIDGKGRMSVPADFRRVLESHDPDWAAGTNPGLYLLYGDHLKNCLRVYTVAAFRQIADDI

QKMPQGSPGRRIASRLILGQSVRLEVDKDGRTVMPSDQRAKLGLAEGELRFTGAGDHFEIWENQTFADTI

TAEVEAFLATQGDDFDPLSLLQG*

>gkv_503|gene_NONE|hypothetical protein

LRKRRVRHLGGWPNGLAAFPFARFLDWWGIGESLRLV*

>gkv_504|gene_NONE|septum formation inhibitor-activating ATPase-like protein

MALTKDVILEALRQVALPDGGDLITRDLVRALALVEGEVRFVIEVADAAMAERFAAVPAAAEAVLAALPG

VQKVNVVLTAARGAPTLKVGRHPTAQPAGPQPVAGIKSIIAIGSGKGGVGKSTVTANLAVALARAGRRVG

LLDADIYGPSQPLMMGEHRKPASPDGKTMIPVQAHGVTMMSLGLLVAPGKAVAWRGPMLMGALQQMLGQV

AWGELDVLLVDLPPGTGDVQMTLSQRTRLTGAIVVSTPQDVALIDARKAIDMFNTLHTPVLGLIENMSHF

TCPNCGHESHIFGHGGVAAEAQNLGLPLLAQLPIDLETRLAGDAGRPVALGDGVMADAFAQLAAGLVRGG

MA*

>gkv_505|gene_NONE|hypothetical protein

VNAQLDSILFLENPQERFASKIRRLRPMHPAHGGLVKASIVRAVNSFTYGLKQGR*

>gkv_506|gene_NONE|benzoate transporter family protein

MRASLFPPAILAALIGYGSTIALVLAAAQALGATPEQTQSWVFALCLGKAAGSVILSVWTRIPTVLAWST

PGAALIAATEGITMAEGVGAFILVGLLIALTGIVRPLGRLIAMIPDSIAGAMLAGVLLPFCLQVTGALSA

APLVVGVMLVVYLIMRLINAASAVLVALVAGLLAAALSGQITLPTQGFALPHLVFITPSFNLGTVFGLAL

PLYIVTMAAQNLPGFAVQRAAGYEPQVGRSLLVTGAGSAITGLFGAHTHNMAAITAAICMDPATHPDPAQ

RWKVALPYGALWLILALSGPWLITLLQGLPPQLLAAVVALGLLAPLAGALGTAMAKADERLAATVTIVVA

ASGVSFFGVGAAFWGLIAGLIVHASDVTTRKTRK*

>gkv_507|gene_NONE|lysE type translocator family protein

MTIAVFFAVLAFAVVNSITPGPNNLMVMASGANFGLKRTWPHLWGITLGFAVMVFTLGIALAGITAIMPT

LHVILKVAAVAYMVYLAWKIANAAPVDDTVSTAQPLTFLQAAAFQWVNPKAWAGAVTAVAAYAPSGSFIS

TFWVGIAFIIAALPSVTLWCVAGQEMRRLLNTPARLRAFNITMAVLLLLSLIPILML*

>gkv_508|gene_uvrD|DNA helicase II|

MGLSSHGALGYGAAMTENSPNISLSQRAMGVRPSPYLEGLNPAQRAAVETLDGPVLMLAGAGTGKTRALT

ARIVHILNTQRAYPRQILAVTFTNKAAREMRLRINAMLGDQAEGMAWLGTFHSVCVKLLRRHAELVGLRP

DFTILDSDDQLRLMKQLIIAANIDEKRWPARALANVIDGWKNKAIAPDSIPTSDASFYDGRAMRLYAEYQ

ERLRQLNAVDFGDLLMHMVRIFQKYPDVLQKYQQDFKYILVDEYQDTNVAQYLWLRLLAGGHSNICCVGD

DDQSIYGWRGAEVGNILRFERDFPGATVIRLEQNYRSTGHILAAASGVIAKNGGRLGKTLWTQSYMGEKV

RLIGHWDNEEEARWVGEEIEALQRGTRGIGPFELDDMAILVRASHQMRGFEDRFLAIGLPYRVIGGPRFY

ERLEIRDAMAYFRLAVSSTDDLAFERIVNTPKRGLGEKAVQTIQRTARERGMSLLDAAGVVVEQRLLGGK

ALASLGGLVQQFYRWHDAVLAGRSHIEIAEEILDGSGYTTMWQNEKTPDAEGRLENLKELVKSLDNFENM

QGFLEHVSLVAENESDDSQPQISIMTLHAAKGLEFPVVFLPGWEDGIFPSQRSLDESGLAALEEERRLAY

VGITRAERVAIISFAATRFFHGQRQSMLPSRFIDELPHDDVEVLTPPTLNNGHYGAASSSPAQVEERAAK

ADVYNSPGWQRMQSRSVQRGAGGMKSARNVVIDAVASPVFELGQRVFHQKFGYGAVTALDAGKVMVAFDK

AGEKHLMAHFLVPAAQADDIPF*

>gkv_509|gene_pyc|pyruvate carboxylase|

MAEFQKILVANRGEIAIRVMRAANEMGKKTVAVYAEEDKLGLHRFKADEAYRIGEGLGPVQAYLSIPEII

RVAKMSGADAIHPGYGLLSENPEFVEACDAAGITFIGPKAETMRALGDKASARKVAVAAGVPVIPATEVL

GEDIDAVRDEAAAIGYPLMLKASWGGGGRGMRPINGPEELAEKVREGRREAEAAFGNGEGYLEKMILRAR

HVEVQILGDKHGQIYHLWERDCSVQRRNQKVVERAPAPYLTQEQREEICDLGRRICQHVNYECAGTVEFL

MDMDSGKFYFIEVNPRVQVEHTVTEEVTGIDIVRAQILIAEGKSLADATGAASQADVQLKGHALQCRVTT

EDPLNNFIPDYGRIQMYRSATGAGIRLDGGTAYSGAVITRYYDSLLTKVTARGQTPEMAIARMDRALREF

RIRGVSTNIDFVINLLKHPVFLGNEYTTKFIDTTPDLYHFKKRKDRATKLLTYIADITVNGHPETKGRPR

PADIKLPRVPKVSIDAPPAGTRQLLDSEGPKAVADWMLKQKRLLITDTTMRDGHQSLLATRMRSIDMIRI

APTYAHKMSELFSVECWGGATFDVAFRFLQEDPWQRLRDIRAKMPNVLTQMLLRASNGVGYTNYPDNVVQ

FFVKQAAETGVDVFRVFDSLNWVENMRVAMDAVVDSNKILEASICYTGDMLDSARPKYDLKYYVAMAKEL

EAAGAHVLGIKDMAGLLKPAAAAQLVRVLKEEVSLPIHLHTHDTSGASIATIMAASAAGVDAVDAAMDAF

SGNTSQPTLGSIVEAMRGTERDTALDVEAIRDISNYWEQVRAQYAAFESGLQAPASEVYLHEMPGGQFTN

LKAQARSLGLEERWHEVAKTYAEVNRMFGDIVKVTPSSKVVGDMALMMVAQGLTRTQVEDPAVEVAFPDS

VVDMMRGNLGQPPGGWPVELQAKILKGEAPMLDRPGASMPPVDLEGVRAKLSAELEGRKIDDEDLNSYLM

YPKVFLDYMGRHRNYGPVRTLPTETFFYGMRPGDEISVEIDPGKTLEVRLQAVAETTEEGDVKVFFELNG

LPRTVRIADRTKVSTLIARPKATAGNPNQIGAPMPGVVATIAAQVGKPVKAGDLLLTIEAMKMETGIHAE

RDGVIKAVHVGPGSQIDAKDLLIELE*

>gkv_510|gene_acsA|acetate--CoA ligase|

MIIKTYPPSQDLADNAHANKATYDQMYAASVSDPDTFWGHQAQRLDWIKPFSRVKNTSFAPRAVNIEWFG

DGTLNVAANCIDRHLVDKADQTAIIWVPDDPGADAQHITYRDLFENVGRMSNVLKGLGVKRGDRVILYMP

MVPEAAYAMLACARIGAIHSVVFAGFSPDSLGARVNGSGAKLVVTTDYAPRGGRKTPLKSNADAALLHCD

DDVKMLVVKRTGDQISWLEGRDYDYNALAASASPYCTPAEMGAEDPLFILYTSGSTGQPKGVVHTTGGYL

TYAAMTHEYTFDYKPGEVYWCTADVGWVTGHSYLVYGPLANGATTLMFEGIPTYPDAGRSWQIVQDHKVS

IYYTAPTAIRALMAHGDGPVQGYDLSSLRILGTVGEPINPEAWNWFNETIGKGNCPIVDTWWQTETGGHL

ITPLPGATPTKPGSATLPFFGVQPVILDPTSGAEQTDVAAEGVLCIKDSWPGQMRTIWGDHARFEKTYFS

DYKGYYFTGDGCRRDEDGYYWITGRVDDVINVSGHRMGTAEVESALVAHPAVAETAVVGYPHAIKGQGIY

AYVTLMNGEEPSDDLRKDLIKWVRAEIGPIATPDLIQWAPGLPKTRSGKIMRRILRKIAENDFGSLGDTS

TLADPSVVEDLIENRMNRG*

>gkv_511|gene_recN|DNA repair protein RecN

MLRALEIRDMVIIDRLDLAFQPGLNVLTGETGAGKSILLDALGFVLGWRGRAEVVRQGADRAEVTALFDL

PAGHPARAILTEAGWDDTDELILRRTASPDGRKQAWVNDRRAGAEVLRALADHLIELHGQQDDRGLLDVK

MHRQLLDQFAVAEPLLAEVRAAWTDLRRLSQRAASLAAERAALAAEEDFLRHAVAELQKLEPQTGEEQEL

DTARRLMQAAARIGDDISRARQAISSDGAEGLVANAIRWLDDAAGQAEGRLDSALDALARALDALGEAEQ

GIETCLDALSFDPMRLEQIEERLFAIRALARKHNVLADELPVLAETLAGRLNDLDSAEAQDAELAAALST

AQKAYDAAATRLTALRQREGARLATMIEAELAPLRMERARFFVEIAPASRGPDGMDEVTFIAATNPGAPA

GPLSKVASGGELSRFLLALKVCLTKDQTGLTMIFDEIDRGVGGGTADAVGRRLRALAEGGQVLVVTHSPQ

VAALGAHHWRVEKHVTDGVTTSRVVPLDEDARVEEIARMLSREPVSEAMRIAARELMAAGR*

>gkv_512|gene_yfiO|conserved hypothetical protein

MTMAGLIKATGGRGMFLVAALSATLLAACSSNESSVLRQPGALDAYSAEQVFDLGEQQLNENRLDDAAFF

FGEIERLYPYSSWARRGLIMQAFAYHRARDYENSRSAAQRYVDFYPTDEDAAYAQYLLALSYYDQIDDIG

RDQGVTFRALQELRRVIELYPDSEYATAAVQKFDLAFDHLAGKEMEVGRYYLSRGNFTAAISRFRVVVED

FQTTTYTPEALMRLVEAYMALGLTDEARSAAAILGHNYQSTPFYADAYALLTGRGLSPEASGDNWLVAIY

RQVVRGEWM*

>gkv_513|gene_lpxC|UDP-3-O-[3-hydroxymyristoyl] N-acetylglucosamine deacetylase|

VQTTLQQPATFTGVGLHSGQQVSLTVLPAGADHGIVFRRTDAAGGGAVIPALWNHAVQTPLCTQLEDAAG

HTISTIEHVMAALAGCGIHNAMIEIDGAEVPILDGSSRPFVMGFLRAGIAQLRAPIRAIEILHEVSVTHN

GATARLSPATSMQIDFEIDFPDAAIGHQSMLLNMSRDAFQRELADARTFCRLSDVEDMRTHGLALGGTME

NAVVVDGDRIVTPGGLRYADEPVRHKMLDALGDLYTAGAPIIGRYTGLKAGHALTNRLLRALFADPTAWR

RVSCNRAMAARLPGQGGLLHATARVA*

>gkv_514|gene_ftsZ|cell division protein FtsZ

MPGHDELKPRITVFGVGGAGGNAVNNMIEQELEGVEFVVANTDAQALVASKAALRIQIGLEVTQGLGAGA

RPAVGAAAAEESLDQIIDHLAGSHMCFITAGMGGGTGTGAAPIIAQAAREMGVLTVGVVTKPFMFEGAKR

MRQAEEGVAALQKVVDTLIIIPNQNLFRIASEKTTFTEAFMMADDVLYQGVKGVTDLMVRPGLINLDFAD

VRSVMDEMGKAMMGTGEAEGPTRAIDAAKKAISNPLLDEISLNGARGVLINITGGYDMTLFELDEAANHI

REVVDPEANIIVGSTLDPDMVGKIRVSVVATGIDAAEKAPEMPVPRRSVQQPLVQQQPLQQPVMQPAVEA

VVRAEAQRNEQFRTEQYRDPAPQPRQEYAPQPEPVRQEPVRQELVRQEPRQTYAPTPRPAAEPYEEVPPM

YTPRPQPTMSQPAAPAHQPDPRYIAPQRPATSAAEALQRLSNAVDHNPVADQRRAPEPQPRFGIGNIIGR

MAGGNQDGQGAAPARPQPSASREEPSSDPERERVEIPAFLRRQAN*

>gkv_515|gene_NONE|hypothetical protein

MPRHRETRDIHALTARIFWHLCSPPRGLARIGLLLPWVWCPFRRF*

>gkv_516|gene_ftsA|cell division protein FtsA

MTDLYQNQRAMRHMRTVALQRGYVAVLDIGTSKIACLILKIGAPRRGDLSDSVGALGGQNAIRVIGAATT

RSRGVRFGEINALAETEKAIRTVITSAQTEAQMRVDHVIACFSGAQPRSYGLEGAVDLAGKTVDEGDVGR

VLAACEVPDFGDGRNVLHAQPVNFSLDHRSGLSDPRGQAGSKLATDMHVVTVDGAVVDSVLRAISRCHLE

CAGIASSAYVAGLASLVEDEQELGGACIDMGGGSTGISIFMRKHMIYADAVRMGGDHVTGDIAMGLQVSM

AQAERIKTVHGGVVATGRDDREMIDIGGETGDWDRDSRRISRSDLIGIMRPRLEEILEEVRARLDAAWFD

SLPSQSIVLTGGASQIPGLDGLATRILGQQVRLGRPLRVQGLPQGATGPDFSASVGLALFAAHPQDECWD

FEIPAERYPARSLRRAMRWFQDNW*

>gkv_517|gene_NONE|cell division protein FtsQ family protein

MRPVDKKPVDRKIERETRYLRRDPAPSRWSYRYQRLMLTPAFRAGVRLGTPVIIIALAVAVVFGRADSRD

WIMGHYNAAIAAVTQRPEFMVGSFAITGASPDLALAIEGLVDIPFPISTFNLDLQDLRTNIAALSPVRNV

NVQAGGGVLQIVIEERQPVAVWRHVDGLRLMDGEGIATGMILNRADRPELPLIAGDGAQAAIPEAMELFR

IASPLGARVLALVRMGERRWDLVLDREQIVQLPAVDAVAALQRVIAQEEAQQLLSRDVAVVDMRNDARQT

IRMTQRARDALRSMPGRSAGRG*

>gkv_518|gene_NONE|D-alanine--D-alanine ligase B (D-alanylalanine synthetaseB) (D-Ala-D-Ala ligase B)

MGGFSAEREVSLSSGRECAKALVEAGYDVVEVDADATLVDRLAHVKPDVVFNALHGRWGEDGCVQGILEW

LRIPYTHSGVLASALAMDKQRTKSTYQAAGLPVVPSLLAARDEVSQRHMMDAPYVVKPANEGSSVGVYIV

HAGANRPPQLSPEMPDVVMVEAYAAGRELTTAVLGDRALTVTDIITDGWYDYNAKYSVGGSRHEIPANIP

VQIYDACMDYALRAHRALGCRGLSRTDFRWDETRGLDGLILLETNTQPGMTPTSLAPEQAAWAGISFPEL

CRWLVEDASCGR*

>gkv_519|gene_murB|UDP-N-acetylenolpyruvoylglucosamine reductase|

MTDMPSVRGTLTADRPLGDLTWMRVGGPADWLFQPADHDDLADFLSALPADVPVFTMGVGSNLIVRDGGM

RGVVIRMGRGFNGITIEEGGVVRAGAAALDSFVARKAADAGLDLTFLRTIPGTIGGAVAMNAGCYGQYVA

DRLIEVTVITRTGERQVIAAKGLGLAYRQSALPDGAVVVEAVFNAPAAAPEDLHATMDAQLKKRDETQPS

KDRTAGSTFRNPAGFSSTGKADDVHDLKAWKLIDDAGMRGATLGGAIMNPKHANFLTNAGGASAADLEGL

GERVRKAVYDASGITLEWEIKRVGEPIVNS*

>gkv_520|gene_murC|UDP-N-acetylmuramate--alanine ligase|

MRMPKDVGPLHFVGIGGIGMSGIAEVLLTLGYQVQGSDLKSSAITERLAGLGATIFTGQRAENIDGAGVL

VISSAIKAGNPELDAARARGIPVVRRADMLAELMRLKSNVSVAGTHGKTTTTTMVATLLDAGGIDPTVVN

GGIIHAYGSNARVGQGEWMVVEADESDGTFIRLPTTIAIVTNIDPEHMEHWGTIEALRKGFTDFVTQIPF

YGLAVLCTDHPEVRALAARVTDRRVVTFGFNEDADIRAVNLTYEKGQAHFDIALPDGVIAGCTLPMPGDH

NVSNALAAVAVARHLGMDHDAISAALAGFAGVGRRFTKVAEVDGITIIDDYGHHPVEIAAVMRAARQATS

GKVVAVHQPHRYSRLAHLFDEFSACFDLADYVGITDVYAAGEDPIPGASRDDLVAGITAHGHPHAFAVTS

EADLADVVRKYMAPGDMLVCLGAGTISAWAYNLPKALAHD*

>gkv_521|gene_NONE|glycosyltransferase family 28 C-terminal domain protein

LIIAAGGTGGHMFPAQALAEAMLARGWRVRLTTDARGARYVGGFPSAVEIVQLKSGTFARKGVLGKLLAP

FAIGAGVISAVAQMIAQRPAVVVGFGGYPSIPAMAAAWLLRLPRMIHEQNGVLGRVNRVFAPRVNAVACG

TWPTDLPKGVEGIHTGNPVRASVLLRAGAPYIQPGDYPMSVLIIGGSQGARILADVVPPAIAALPMPLRH

NIRVSQQARPEDIDRVTALYAAEAIPAEIAPFFNDIPTRMSEAQIVITRAGASTIADLTVIGRPAILVPF

AAATGDHQTANAAGLVQQGAAALLPERSFEVDALSQLLEGILSDPPKALAMARATLAQGRPDATDRLVEL

VEGLAKPAKYAVSEAKE*

>gkv_522|gene_ftsW|cell division protein FtsW

MTDMVYRAAAVQQVGDPVLPRWWRTIDKLTFGCILVLFALGLLLGLAASAPLAQRLELDSFYFVVRQAVF

GIAAITLMLMVSMLSPRMIRRLGVVGFVFAFASLAALPFVGTDFGKGAVRWFSLGVASFQPSEVLKPFFI

VVTAWLLVAGGQEGGPPGKRISLLLTAVIVIMLALQPDFGQAALIIFTWTVMYFVAGAPMVLLMGLAGIV

AGGGVIAYNSSDHFARRINSFLAEEFDPRSQIGFATNAIREGGFFGVGVGQGQVKWSLPDAHTDFIIAVA

AEEYGLLLVGAIILLFLVIVVRSLFRLMRERDPFIRLAGTGLATVFGVQAMINMGVAVRLLPAKGMTLPF

VSYGGSSLMAAGLSVGMLMALTRARPQGLIGDILLRQGRR*

>gkv_523|gene_NONE|hypothetical protein

LTARAAQEAALHWLLRWIDGPVRAILTGQTRKTGGFEAFSGGER*

>gkv_524|gene_NONE|pyridine nucleotide-disulphide oxidoreductase family protein

MQVNTLIIGAGAAGLYCAAHAGPGTLLVDHARAPGEKIRISGGGRCNFTNIHAAPQNYISQNPHFVKSAL

SRHSAADFLALVEAHRIPWHEKTLGQLFCDRSAKDIIAMLTSMVERAGAALWLETSVVDVAHDGTHFRVT

LEQAGERTQVTAQNLVLASGGKSIPKMGATGFAYRVAEDFGLPVTETTPGLVPLTFSDNQMAPLAGVATP

VIARAARGPAFPEALLFTHRGLSGPTILQISSYWQAGEDIAINLLPGAPVIDQLRAQRKGAGAKSIASVL

GGLLPRKLVDVLLEDWGLGGNYADLSDKALQDMAQRLGDWQLRPTGSEGYRTAEVTIGGIDTNALSSRTM

EAKAVPGLYCIGECVDVTGWLGGYNFQWAWSSAHAAAMAISGR*

>gkv_525|gene_NONE|UPF0335 protein ORF1

MSDITSTSVAADEIRSIVERYETLEAEKKYVAEQLKEVMAEAKGRGYDTKVLRKIIALRKRDQNDLAEEE

AILDLYKQALGM*

>gkv_527|gene_NONE|putative membrane protein

MQSLIWIGAIVTLSGLAGVVWTLVQVLGARRRGASPEALQEVVKKVAPVNLGAFFLSFIGLMLVVVGLIL

R*

>gkv_526|gene_NONE|bacterial regulatory protein, arsR family protein

MEIQSEHIDEKSRYCADMAALAAVAEGASDVFRVLSNPQRLRILCALNSGACCVSQLEDNLGASQAYVSG

QLARMRSEGVVTCARAGRQMVYSIADPRLQAVLAGLAQAVAQIAASQMAVSAQDQADNHQHQTDK*

>gkv_528|gene_NONE|surface presentation of antigens (SPOA) family protein

VHMAGGLKTMLGRGRPHIPMGGAQPQRVLRQSFARALTEVGGLRATLALPDVGEGVLDDILPALPDGMIL

FSLVCDGDIVGLMAFDAQLRMAVVEAQTLGEPYHASMPERPRTSVDEVLCQPVADRCITDLIAEALAAPT

SGWSVQAWVEGVVCDRPFSGGRDAAFALSHGAYRQMRIGVGISAERAGSLVLLLPVEGARAAVAQRRKLA

EDAPARAVEASRSRHAGWSLSINRNVMGAAVDLQAVLHRMRLPLSQIEGWAAGDVVMMPGASLAAVRLVG

VADTPVGTGQLGKAGGMRAIRMKEGAAPLALEDMEALGGKGWENRASLAG*

>gkv_529|gene_dgt|deoxyguanosinetriphosphate triphosphohydrolase, family protein|

MTTSWPEGSYGTLASYAADPARTRGRLYPEEESIHRSPFQRDRDRIIHSSAFRRLKHKTQVFLEHEGDYF

RTRLTHSIEVAQVARTLAGSFGLNQELAEAVALAHDLGHTPFGHTGEDALDALMKPYGGFDHNAQAIKIV

TSLERHYADFDGLNLTWETLEGIAKHNGPVPAPVAFALADYDARHPLDLHTYASAEAQAAALADDIAYNH

HDLHDGLRAELFSTDELAELPILQDCFAEVDRKYPGLNYYRRRHEALRRFFGVLVEDVIHVTRTNLAELQ

PQTADDVRHAGRAMVRFSDGLWADLKVIRGFLFTRMYRAPGVVMMRQEATKVIETLFPLFMEQPDELPRQ

WRADVEKAQDQQALARIVSDYIAGMTDRFAIETYARLVDEDMTDRTRALRIL*

>gkv_530|gene_NONE|iron-sulfur cluster assembly accessory family protein

MFTLPPTVTPRAFERLAQIGAAKQGKALRVAVEGGGCSGFQYQITLDAPQGDDLVLEGAGEKVVVDQVSL

PFLASAQIDFSDALIGARFVIENPNASTSCGCGTSFSI*

>gkv_532|gene_xth|exodeoxyribonuclease III|

MRIATYNINGIKARLETVTEWLTRESPDVAIFQEIKSVDEGFPRSAIEDLGYNLETHGQKGFNGVAIASK

LPMEDVTRGLPGDDNDEQSRWIEATVIGKTAVRICGLYLPNGNPAPGPKYDYKLAWMERLHKRAEALLAS

EEPAFMAGDYNVIPQAEDAKNPAEWLEDALYLPQTRDAFRRILNLGFTDAFRAITQGPGHYSFWDFQAGA

WQRDNGIRIDHFLLTPQCADLLQNAWIEKDARGREKPSDHVPVWVELDA*

>gkv_531|gene_NONE|FAD binding domain protein

MSDDRSVAVIGGGIAGTCAALAFAQRGARVTLYERAPQIREFGAGLQITPNGARGLQALGIDLSAGLTAQ

AVAPHDGVTGRAIARFDLTQLSGPPYRFFHRGTLIGLLADACTRAGVDIRTGQQVLDATPDGAVVTAASP

AHYDLVIGADGVHSIVRQRLAPSTPRFSGQVAWRGVIAGDHPPEARIWMLPGHHAVTYPLPDGQINIVAV

RERKAWAPEGWSHPDATENLRAAFCDAAPELAMMLGQIAQPMLWGLFLHPVAPVWRADRLVMIGDAAHPT

LPFLAQGANLAIEDAVTLGAADDLAAWEAARILRTSRAIAAANRNAKNYHLRGPARALAHSGLGMIGRFA

PARFLQSMDWLYGFDPSQASSSTQTGT*

>gkv_533|gene_dksA|RNA polymerase-binding protein DksA

MKAEVFLPDDYRPAEDEPFMNDLQLEYFRRKLLGWKADLLSDSRDTIEGMKDQTRNIPDLADRASEETDR

ALELRTRDRQRKLVSKIDAALRRIDEGEYGYCEITGEPISLKRLDARPTATMTLEAQERHERREKVHRDD

*

>gkv_534|gene_NONE|putative membrane protein

MRQIATLYADKTMLLPLAGLILGAALGAWRASRRGGKALDLAQWAAVHGVFGFIIGVTALIVITRMAS*

>gkv_536|gene_NONE|peptidase family M48 family protein

VLRLLPFVLLIAWPLVSYWLAVRRTRQMLDRQSTPLVEPRLNAMAKQLGSALDLPTVKVHILEIAAINGL

AAPDGRIFITRGFLKAYAAGEITAEEIASVIAHELGHVALGHGKRRIFDFAGQNALRTALMVLFGRFLPG

VGPWLAGLAASAFAARLSRSDEYEADAYATALLGRAGIGVQHQISMFKKLDGMTGNLGAQPAWLISHPRP

DQRIAAIEANAAKWGLRVE*

>gkv_535|gene_NONE|hypothetical protein

MSESPRVVVDTCVLFPTVLRELTLGAAARGLFRPLWSERILGEWQRAVIKLGPAAVAQVEGEIAMARVHF

PKALVSYQPALEERLYLPDPNDRHVLAAAIAGSADAILTMNASDFPRNILSEEGVLRVDPDGFLTDLAQK

HPDQMRAVADEVLARANHFAPKDAAPWRLRALLKKGRLNRLGKLLDP*

>gkv_537|gene_NONE|conserved hypothetical protein

MTEHTPDPATTYATVRLSPKADARAIRHGFPWVYSDELVLDRRTKALTPGTFAVLEDAERRPMAIVTVNP

ASRIAARVMDQNIEAVIDTAWLAAKLTRALAHRTRLYDQPFYRLVHAEADGLPGVVIDRFGDTAVIQPNA

AWSERMVENLADALAEVTGVTTIIKNGEGRARSLEGLPEEMAVLRGTAPTAPIAVPMNGATYMADVMGGQ

KTGLFYDQRPNHAFAASLSRGVKVLDVFSHVGGFALAALAGGAASALAVDGSENALTLASAGAAAMGVED

RFATRKGDAFATLEALAAEGETFDIVICDPPAFAPNKNALEAGLRAYERIARLAAPLVAEGGYLVLCSCS

HAADLTKFRNASARGIGRAGRRGQIINTGYAGPDHPLLPHLAESGYLKSVFFRL*

>gkv_538|gene_NONE|glutamate-ammonia ligase adenylyltransferase family protein

MTHDAPLANHLTRCPTAFDPARGADVVRLLPDLPADIASLIAGTAGSSPYLARLIEREHDWLAPALDAPR

RAVDQVIAALGDIAPDILGAGLRQAKRRVALIVALADLGGVWPLEDVTGALTRLADAATDAAMKAALMAE

VARGKLPGMSADDVQSGAGMVALAMGKMGAGELNYSSDIDLICLFDDSRFDRDAYPDARASYVRATRKMT

ALLSDITAEGYVFRTDLRLRPDASVTPVCVAMSVAEQYYEAEGRTWERAAYIKARAAAGDLQAGARFLRA

LRPFVWRRHLDFAAIQDAHDMRLRIRDHKGLNGRLNLLGHNMKLGRGGIREIEFFTQTRQLIAGGRDDSL

RLRGTVPGLMRLSETGWITPEVAEDLTDHYRAHRLVEHRLQMLNDAQTHDLPVTEEGFARLAAFMGTSTA

DLKADLTRRIKAVEALTEDFFAPDDSATAREADFGAEVIARWRSYPALRSVRAGQSFRRLRPRILSRLQQ

AAKPEEALAVFDQFLSGLPAGVQLFALFEANENLLNLLIDICATAPALAQYLSRNAGVLDAVIGGSFFAQ

WPDDLPAELHTAIARAPDYEAKLDAARRWMKEWHFRIGVHFLQGLTDAEGAGTQYAALADSVLQGIWPVV

SAEFARRHGAAPGRGAIVVGMGSLGAGRLHAQSDLDLILIYDAQGVDASDGPRPLAVSSYFARLTQALVT

ALSAPMAEGRLYEVDMRLRPSGRQGPVATGWGAFRAYQTDEAWTWEHLAQTRARPIVGDAGLMAEFEGFR

RDLLALKAGGAKVRADVLDMRARISAAKPPQGDWDAKIGPGRLQDIELFAETMALLASNPARTVQDQLAV

ADLPEGERQTLQIGARLFWQLQSAQRLLTAGPLSADALGAGGRAFLLRETGQPSVVALNAAIEQVAARAA

GVIETHLSADGQSA*

>gkv_539|gene_NONE|magnesium chelatase, subunit ChlI family protein

MSNDDTLLAEVEALQTQLIAARASIARRVIGQERVVDLALGAVLAGGHALLVGLPGLGKTRLVGTIAQVL

GLNSSRVQFTPDLMPADILGSEVLETAADGTRSFRFIEGPVFCQLLMADEINRASPRTQSALLQAMQEGE

VTIAGQHRPLGRPFHVLATQNPLEQEGTYPLPEAQLDRFLVMIDVPYPSRETEREILLATTGTDEAEATA

IFTPDQLLAAQTLLRRVPVGQTVVDAIIDLVRACRPDDPTAPDYIRENITWGPGPRAAQALMLLARAEAL

LNGRLAPNMADVAALAGPVLGHRMAASFAARARGETVAALVARLVTETTGLDAAA*

>gkv_540|gene_NONE|conserved hypothetical protein

MSDQTAHSADLHTGLRADSEKLAHALPALLAAARALAATITPGTHGRRRAGLGDTFWEYRPAEAWDDASR

IDWRRSARGDTAFVQDREWQLAQSLSLWVDDSAAMRYAMDANHPSKAQIAQRLALALAVLLERGGERIGY

LTADLPPRRGRLQLERLTAALLHQGDHDYASAPPAMAPPRGQVVIFSDFFGDLAALEAYIASATGRGCTG

LLVQVLDPTEESFPFTGRTLFRSMSGAIRHDTLKADALRETYRARLAARKDAVAGLAASAGWRFHTLHTD

TPASQSLLWLYGALDGVSA*

>gkv_541|gene_NONE|N-terminal double-transmembrane domain protein

MSGLIFTSPWLLLGLIALPALWWLLRAVPPAPLTRRFPGVALLLGIPDPEAESARTPWWLLLLRALAVAA

LILGFAGPVLNSTTRVTGGGPLLILVDGSWAQAADWDATRARIATIAGEADRPTAIEVLTAPTPDGPLFR

NPADLQGMIEGLQPRAWQPDALPAWLETIDGTFDTIWLSDGLARSSRAELANALQQRGTLQVIEATAPTF

AILPPVFEDGGVDLTVQRSTAGPAFSLPVLAQGTDPNGIARTLATASADFAADATAADVRFELPPELLAR

MTRFDLGTAPSAGATALADDRLRRREVALIAPAGGSEAVNLLSPTHYLRQALAPSADLIETDLPTAMRAG

PDVIVLVDIANLTPDETATLETWVDQGGLLVRFAGPRLAASDMGRGAVDPLLPVRLRAGGRSVGGALSWG

EPKTLAAFTEGSPFFGLTVPDDVEVTAQVVAQPDPTLAARVIAQLSDGTPLVTREMLGQGQIVLFHVTAT

PEWSNLPLSGLFVQMLERLSLASGTALDEASLAGTRWAPDQVLDAFGALHSSDSLAPITGEELAVARAAA

ATPPGLYAGQGRRIAVNAMSAGDTLAPAIWPAGVQVLGSDGAAAVALRPWLLMAAIVLLLVDTLASAALS

GRLRGRGLAALLLIALAPLPRPASAQDFDAGFAISATSQVVLAHVSSGDPEVDSIVSAGLTGLSRALALR

STIVTSRPIQLDLDTDELAFFPMIYWPVTAQSPLPSREAYQRLNRYLRTGGMIVFDTRDAEIGAFGATPA

SRRLQEIALPLDIPALEPMPADHILTRSFYLLQSAPGRFTSDLWVEAAPPEELAEGMPFRNLNDNVTPVV

IGANDWAGAWATRPDGAPLLPVGRGLAGDRQREMALRFGVNLVMHVLTGNYKSDQVHVPELLNRLGN*

>gkv_543|gene_NONE|putative membrane protein

MEGSVIFAPLLSWPLVWAALALALVIGLLALWRGLGGWGWRALAALALALALANPSLMRETRDALSDIVL

VVVDETASQRIGTREAQTASALDQITRQIEALPNTEMRIIPVRNGEDDNGTRLLTALSNALAEEPAARIA

GIILLTDGRAHDLAVTPELPAPTHLLLTGEPDDWDRRLIVRNAPAFAILDEPVTLTLRIEDQGAAPESGP

VPLTIAIDGGDPMTFTVPVGRDIELPLTLPHGGMNVLQFQTPTADGELTDRNNTAVVQINGVRDRLRVML

VSGEPHAGERTWRNLLKSDPAVDLVHFTILRPSDAVDSTPSEELALIAFPTRELFVDKINDFDLIIFDRD

RRRGLIPDDYMMNVVNYVQGGGAVLVSTGPEFAGAESIARSPLGFILPARPTGQVFDQAFRPQLTELGQR

HPVTAGLPELSGVDEDGNPLWGRWLRLLAVTPDPNAEIVMADESDNPLLILNRVGEGRVALMSSDQAWLW

TRGFDGGGPQNELLRRLAHWLMQEPDLEEEALWAEPQGQTLRIIRRTMAEDAPQVTITQPDGSTVTVQPT

EVAPGRFEALFRAPEAGLYRLTDGELDAVIAAGPAQPREFEETIATADILSPATTATGGGVVHLSDGTPD

IRTARAGQSAFGEGWIGITPRDVSRVTALDINALLPPWGWLLIAAALMLTGWLREGRRANA*

>gkv_542|gene_NONE|conserved hypothetical protein

MNLIELLIIIGWKDLAVLAMIAVIWLVLGFVIEHPSARRPSVSRLMEGYRRAWMEDFSKRENRVFDATII

SSLRQSTSFFVSTCLLAVGGLLALMGNIDQLSGVAEQLTHQNESQLLWQLRLLPATVFLIIAVLKFIWSN

RLFGYCSILMGSVPIDKDSPQGPARAARAAELNIRAAISFNRGLRAMYFALATLAWVMGDALLVISVLAV

TGFLWSREFASRSRDVMLDGADDH*

>gkv_544|gene_NONE|trypsin family protein

MPKVLPVFIAALLSAAPALAQDHAREGAVGRIEIEDAGHCTAVLIAPDQVLTAAHCLFDADTGARIPAQD

IVFQAGFTNGRAYAYRQIRRIVIHPDHQPDTRASGAAARSRADLALLELYQPITPLAGITPLPIASVIPL

EGALNVIAYGTGPAGAHQTQDECRRIAQDGGVYIFGCALQVGGSGAPILANRGGTPQIVALVSAIGQMRG

RDVVVGVNLPQQIGALRAAFDASASSGAEPRR*

>gkv_545|gene_NONE|hsp20/alpha crystallin family protein

MRTFNLAPLHRATVGFDQIADMLDRTLSADAGQTNYPPYNIEKTADDAWRISVAVAGFADADLDVELRDH

ALIVTGRKADDTTTRKFLHRGIATRAFERRFQLADHVRVQGASHENGMLHIDLIREVPEALKPRRIEITS

NAPTLVEAKSVN*

>gkv_546|gene_NONE|acetyltransferase (GNAT) family protein

MITYRDAIPTDAAGMSRVLQDIITLTGRQRASDEAFCLRSYIENPANVKCTVAVDEAGQVMGFQSLTIAG

PDNAYGTPEGWGSIGTHVSPAAHGRGIGRTLFQHTRAAAEGAGVEQIDASIGADNPEGQGYYGAIGFATW

REANGRVQKVFRIAK*

>gkv_547|gene_polA|DNA polymerase I (POL I)|

MTGKETAPFGKGSHLHLIDGSAYIFRAYHALPPLTRKSDGLPIGAVAGFCAMLQRYVENNNGPEAATHIA

VIFDKGSITFRNDIYPEYKAHRPPLPEDLRPQFPLTRAATAAFNIAYKEIEGFEADDIIATLAREAAEAG

GRVTVISSDKDLMQLVGPAVCMFDPMKNKLIDADGVIEKFGVAPDRVVDVQALAGDSVDNVPGAPGIGIK

TAAQLIGEYGDLDNLLANAAQIKQPKRRETLINFADQIRVSRRLVQLDAQVPLDFTLADLVLRAPEPEVL

LGFLTEMEFRNISKRVADKMGVDAPMIAAATAKGIAEKSAPKTVAPPPPDAPIDHAAYVNINTPEMLADW

IDAIRAQGYVAFDTETTSLDEMQAELVGVSLALVPGRAAYIPLGHRMAAGDDLFADNRLTEGQLPLEVVL

NALRPVLEDPAVLKIGQNIKYDVKIMARYGVHLAPVDDTMLLSYALHAGLHNHGMDELSERYLNHRPIPI

KELIGSGKSAVTFDHVPVEKAVAYAAEDADITLRLWQHFKPQLPQSRVTTVYETLERPLIPVLTRMEMAG

VQVDRDTLSRMSNAFAQKMVQLEEEIHAAAGRSFNVGSPKQLGEILFDEMGFQGGVKGKNGAWGTGADVL

EDLATEHALPGLILDWRQVAKLKSTYTDALQDHINPDSGRVHTSYSIAGANTGRLASTDPNLQNIPVRTE

EGRRIRGAFVAPAGKVLVSLDYSQIELRILAQIAQIDALKEAFQQGLDIHAATASEMFNVPLDQMTPEIR

RQAKAINFGVIYGISAFGLARNLRIPRDRAQGFIDRYFERFPGIRAYMDQTIAFAKENGHVRTLFGRRIN

TPEINAKGPHAGFARRAAINAPIQGTAADIIRRAMIQMDPAIAHLPARMLLQVHDELLFEVEENAADELT

AIAKRIMEGAADPAVHLDIKLVVDAGRGQNWAEAH*

>gkv_548|gene_NONE|chain A, Solution Nmr Structure Of Q5lls5 From Silicibacter Pomeroyi. Northeast Structural Genomics Consortium Target Sir90

MNSLTQMQARALTRPAPVTREVATTRIACDGLTENAALGHPRVWLTIPADSDFIDCPYCDARYIRAPGAA

EGH*

>gkv_549|gene_NONE|HIT-like protein hinT

VTTTYDPNNIFARILRGEIPNNTVLETDHALAFRDIHPQAPTHVLLIPKGPYVDFGHFVAEASAAEQAGF

NAALDAVLKALELPAGFRVIANAGPDSNQEVPHFHLHILGGRNLGPLLPA*

>gkv_550|gene_NONE|epsK domain protein

MTKIAFILLCHKDPKAIINQAVQLTSGGDCVAIHFDGRASGEDFDEIQRGLSANPGVTFARRRLKCGWGE

WSLVEATIEAIRAAEKAFPDATHFYMVSGDCMAVKSAIYTHKFLAAENVDYIECFDFFESDWIKTGFKEE

RLIYRHIFNERTNRKLFYWSFDLQRKFRLTRDVPHDLQVMIGSQWWCLRRSTITAILKFIDKRPDVMRFF

RTTWIPDETFFQTLVPHLVPEKEIRRRTLTFLMFSEYGMPVVFYNDHYDLLVSQDYLFARKISPEAKDLR

LRLGQLYGSERDDFKISNEGRNLFKFQTNRGRTGQRFSPRFWEEITSVGRGREILVVVCKKWHVAKRLVQ

EASRLTGLPSLEYMFNEDRAPVPDLGGIQTTVEKRTRHRRALLRMLFEYFDSDRMIVCLDPSNVELMSDF

DADRSTMRVLEIECEMSDDFLRGHATRVGLASDHSSGDLLERLLPAVRADLRLESDRLRDMGFEHFHRIR

ENGHEGENIAALVEFFGIKPDLAASINNSSSIFAD*

>gkv_551|gene_NONE|conserved hypothetical protein

MSFPGTWMTESESMIYRVVPKCACSTIGQIMYYSDHGRFFDGDIHDAGQGLHKWNRPESQDLISKAVSDH

QTYVFTCVRNPYTRILSSFFDKIAGIQRNGRRYRGNLVPALIQKYGIEVGSPEDNFEFDQIKSFRRFLLF

ARDSIRFRKPMEPDIHWSAMSGHISTFIANGGRYDKIFWTEKFNEGMADVLRSTKTPQPVDIKEIPRFNE

SEGHGPKRLHPVEDYFDDLSMHLVFDMYKRDFRLFKYDFENPANKNPIAEIDLDEVHKRLAK*

>gkv_552|gene_thyX|thymidylate synthase, flavin-dependent|

MPLSPEQHQEIEAQRGQSATTLRAVSPGMEAHLYKAHQVLDHGFVRVVDYMGDDSAIVQAARVSYGRGTK

SVSNDEGLIRYLMRHWHSTPFEMCEVKLHVKLPVFVARQWIRHRTANVNEYSGRYSILDREFYIPSAEHL

AAQSVVNNQGRGEALSGEEAARVLAWLKDDAGRAYDHYEAMISQEGQQGLARELARMNLPANIYTQWYWK

VDLHNLLHFLRLRADAHAQYEIRVYADALCEIVKDWVPFAYKAFEDYRLGAVSMSTQAVDVIKRRLAGEA

VTQETSGMSKGEWREFVAIWG*

>gkv_553|gene_NONE|conserved hypothetical protein

MEFQMTPVAVGVLAVSMSVDAFVASVGKGAGAQRVKFGTALRTGLIFGVVEMITPLIGWALGIAASQYID

AVDHWVAFALLAAVGAHMKWQALQHQPDAPRGDTTLWATVLTAIGTSIDAMAIGVSLAFLEVNILLIALA

IGTATMLMSTGGMLAGRLLGQRFGRVAEALGGIALIGLGSLILFEHLTAAAG*

>gkv_554|gene_NONE|conserved hypothetical protein

MNALDRIAAALERMAPAPVAAPDFGAAAFVWHTSPDRLAPVDHVSRVDVDLLVGINRARDTLLANTCQFA

AGLPANNVLLWGARGMGKSSLVKAVHGAVSADLPSLKIVEVQREDLPSISRCLNLLRGRPERFILFCDDL

SFSHDDQHYKALKAILDGGIEGRPENVVLYATSNRRHLMPRDMIENERQAAVHESEAIEEKVSLSDRFGL

WLGFHAADQDEYLQMIRGYCDAYGIEIDDATLRAEAIEWQATRGSRSGRVAWQYFTDLAGRRGVTI*

>gkv_555|gene_tatC|twin arginine-targeting protein translocase TatC

MSKTNEIEDTAAPLIEHLAELRNRLIWSVLAFVVAMTICFFFATPVFNVLTGPLCVELAKRGQDCQLIFI

SPQEGFFVAVRISMLGGLILSFPVISYQLWRFVAPGLYKQEKMAFLPFLLASPFMFFLGAAFSFFIVMPM

AYAFFLGFQQFAPDGSAVVDPNIAPMASVVFQGSAQEYLSLTMALIVAFGLCFQLPVLLTLLGRVGIVTA

AGLKSVRKYAIVGILVLAALVTPPDVMSQIILFAAVYPLYEISIFLVQRYEKKREAELRAEGLWVDDEEL

DDQEHAVGTDLAKKP*

>gkv_556|gene_NONE|sec-independent translocase protein tatB homolog

MAIIGIVALIVVGPKDLPILFRNIGNFVGKAQGMAREFSRAMNAAANDSGLNEINQTLRSAGQSLNTATT

AAKKPTAYGAQKLREAAGLTGATPEETAAKAAADQSLRDTGAAAMTRNTETASLAGVGRPNLVPTAPVTA

PVAAPVTPPVTSVETAAASTPVAEPAAAPVTTPVSAPISAPEKSQ*

>gkv_557|gene_NONE|mttA/Hcf106 family protein

MFNNIGPMGILLIAVVVLVLFGRGKISNLMGEVGKGITAFKRGVDDGKKELEVSATELRDVTPAAPVATP

VAEEAPKDRV*

>gkv_558|gene_NONE|HTH domain protein

MPSPRNQRLFQIMQILRSAPGGALLSAHDIATQTGVSDRTIYRDMATLIDSGLPVAGTPGQGYHITAAIT

LPPLNLSLDEIEALHIGLSILGEADDIGLRAAAQSLSNKVDAALSADLSDADRRWALSGPGVNAAVAEAA

RGFHFLPVLRSAIARRQKLRLSLSASASPPSERIVRPLRIDYWGRIWSLQCWCETTGGMEQLRTDHIDSV

NVLPQLFTPPPQ*

>gkv_559|gene_NONE|hypothetical protein

MIGVTGGMEATSGGLFSQSLMVYRGVDLTVTDGVALGDSLTFAAGLLPDDYYTLDREAAPARLSFAVAET

GRLMIAPGSSIGTAGHSLHVDSVLLLLDRNGRVAEVLLLVEEKGGIAIAVYALPLSPLNRGFEYRLAAVT

QRNARHRLFGTPMLQFTHDTLLTLADGSRIAASDVQVGQHLMSGDGNMITVNWIGRSQLMAARDMAPVLV

PAGSAGNQSDLILGPRHRIRGRLIRDMIGRSNIRQIEPMVQHYVQILPHRHRSLAVAGVAVDCLMIEAPP

PHTANSAPVAQGLPLPAVAQNDWR*

>gkv_560|gene_prfC|peptide chain release factor 3

MQNQTSPLPAEIARRRTFAIIAHPDAGKTTLTEKFLLFGGAIQMAGQVRAKGEARRTRSDFMKMEQDRGI

SVSASAMSFDFGQFRFNLVDTPGHSDFSEDTYRTLTAVDAAVMVIDGAKGVESQTRKLFEVCRLRDLPIL

TFCNKMDREARDTFEIIDEIQENLAIDVSPASWPIGSGRDFLGCYDLLHDRLELMDRADRNRVAETVSIS

GLDDPKLAEHIPTDMLKKLREEIEMARELMPAFDRERFLEGSMTPIWFGSAINSFGVKELMTGIGEFGPE

PQPQKAAERMVPAGEGKVAGFVFKVQANMDARHRDRVAFIRLASGHFERGMKLIHVRSKKPMAVTNPVLF

LAADRELAEEAWAGDIIGIPNHGQLRIGDALTEGEMLHFTGIPSFAPELLQNIRAGDPMKAKHLEKALMQ

FAEEGAAKVFKPSIGSGFIVGVVGALQFEVLASRIEVEYGLPVRLESSQFTSARWVSGDKDAVEAFVSAN

KQHIATDNDGDLVFLTRLQWDIDRVARDYPKVSLTATKEMMVS*

>gkv_561|gene_NONE|hypothetical protein

MHMIQTRMISSLAVGLALFAAAAPLAAQQVTPGRLAVEANAKEDVNGACRVSFVVGNGLEENLYKLSFEA

VVMDTAGEAAQFTLLEFTEVPQGAVRVKQFDLRGRACDDVGQVIVNRTLTCEAVGLDPQACAQNLDVASR

ISTPDVTEGQISVELNRLESFEGSCRLTFVTRNGLSQPLGGMMAETALFDTNGGLSRLTVFELGDVAAGQ

TRVRRYEVTGSACDGLGNVLVNDWQSCTVEGTEAGACTAAINVTSRTAIGLLQ*

>gkv_562|gene_NONE|hmuS protein

MAAADTHAQIPTPAEIRQARADNPKARDRDLAESLGVSEAALVAAYVGHGVTRIAANPDQLMPLIPALGE

VMALTRNEACVHEKVGTYSEYHANPHAGSVLNPNIDLRTFPKHWVHGFVLEKETETGTRRSIQVFDSAGD

AVHKIFLREGSNVEALEAVKDALRLPEQSDVVETTARPAVQGPKADPSKADALRADWQAMTDTHQFLRMV

SGLGMNRLGAYHTVGAPYARLLDKTAFQAMLDGVVAQEIGIMIFVGNRGMIQIHTGPIYKLMPMGPWQNI

MDPGFNLHLRADKIAEVWAVTKPTSRGDAISIEAFDAEGDIILQVFGVQKPGMEHRPMWNALVEALPSAQ

VEEVA*

>gkv_563|gene_NONE|hmuT protein

MRRLLLTSAIAIAVATGAQAQDRILSLGSSVTEILFAIGAEDKVIARDLTSTYPAAAEALPDVGYVRALS

PEGVLSVNADMIIAEPDAGPVETIDVLKAASIPWVTVPAGWDAAQIVEKINLIGEATGHAAEAAALAATV

TAELETAATAAAEIPEDQRKRVLFIISTNGGRVMAAGSETGGNAIIELAGAVNAVQGVEGYKPLTDEAIT

AAAPDFILMMDRGHNLDAANDELWAMPVLASTPAGQNQAVIRMDGIYLLGFGPRTGAAALELHNALYAGN

*

>gkv_564|gene_NONE|hmuU protein

MTFALSHAASVDNADPREVRARRLTLLLIAALVVTCGASVMFGASGTSVTKVLGQLWRGEEIALIDQIVL

LQVRIPRMVLGVLVGASLAVSGAVMQGLFRNPLADPGLVGVSAGASLGAITAIVLGGFLPAAALAFVGGW

LVPAAAFVGGWGATMALYAVATRSGRTSIATMLLAGIALGALTGAISGILVYRANDNQLRDLTFWGMGSL

AGANWPKVLSAAPLIVIALAVAPFLARSLNALALGEAAAAHMGIPVQKMKSVAILTVAGATGAAVAVSGG

IGFIGIVVPHLLRLAAGPDHRHLLVNAGLLGAIVLLLADMISRTIVAPAELPLGIVTAVLGGPVFLWVLL

RQRGVVDL*

>gkv_565|gene_NONE|ABC transporter family protein

MTVTARQISVKLGRKQILEGVDFTAAGGRLTAIVGPNGSGKTTLLKALTAEIGNGDGVEINGRAINALKP

WQLAAMRAVMPQATSLAFPFTAIEVVRLGLQAGVHAADRTLARRALERVGLQDKAEQHYQQMSGGEQSRV

HLARTLCQVWEPMAHGKPSWLFLDEPVSALDIGHQLLVMDITRDFARAGGGVVAVMHDLNLTALYADHVV

LMRDGAILAAGAVQDVMTSENLSRAYGCALRVNHAPTADHTFLLPHAASSHAA*

>gkv_566|gene_NONE|phosphate ABC transporter, periplasmic phosphate-binding protein

MSLKTVCVSAIAIAAVAGAAQARDNIQIAGSSTVLPYASIVAESFGENFPEFPVPVVESGGSSGGLQRFC

AGIGENQTDIANSSRPIRAGEIETCAANGVTDIIEVRVGYDGIVFASALNGPEFAFTPADWYKALAAEVV

VDGEIVPNPYTTWDQVNPALPAQQILAFIPGTRHGTREVFDEKVLVAGCEESGAAEVLSAARGDEAACVA

LRTDGVSVDIDGDYTETLARIAANPQALGVFGLSFYENNTDTLRVATMSDIEPTVEAIATGTYPVSRPLY

FYIKKAHIGVIPGLKEYAEFFMSDDMAGPAGPLAQYGLVSDPELAETQALIANETVMASN*

>gkv_567|gene_pstC|phosphate ABC transporter, permease protein PstC

MPLLWTLIVILAIAAIGYWLGRSRAMQSAGHSTRALHSLPGYYGWNVAIWAMAPAFLLILVWLVIQPAYV

NHIALQALGDAAQNSGSASLLLADVHRMADQLAAGSAISAEGPTAAAAQLSFAANTAGRMWMSIAAIALA

LAGTAYAWSRTNAQFRARPRVEAAVRTLLIASASIAILTTVGIVVALIFNTIAFFQAYPALDFFFGLTWS

PSAGGVNSRLGILPLLWGTLYISFIALLVAVPLGLFSAIYLSEYASPRIRAIGKPMLEVLAGIPSIVYGL

FALIVVGPLLMSWFSPTGMLGLGWMRGGTAVITAGVVMGIMIIPFVSSLSDDIINAVPQSLRDGSLGLGA

TRSETIRQVVLPAALPGIAGAILLAASRAIGETMIVVMGAGAAGVLSLNPFDAMTTVTAKIVSQLTGDAD

FSSPEALVAFSLGMTLFVITLGLNVLAMAIVRKYREQYE*

>gkv_568|gene_pstA|phosphate ABC transporter, permease protein PstA

MTDMNTTPKQSLLAPDARTKKRNAAEARFRAYGIGALLIAMGFLIALMWSIFGNGIGAFTQTFVKIQVPL

EESVLDPNGNREPADLARATTLRYGSLLQGAMASTLEREGITTDMEPAALAQILSSSAAAEVRNRVLAEP

SLIGQTVELEVLASSRVDGYMKGRVSRESLARDRNLSPAALNVVDQMRDAGVIERRFNWNFIFGSDASDQ

RPEQAGIGVSMLGSLAMMLVVLALSLPIGVAASIYLEEFAPQNRFTDLIEVNISNLAAVPSIVFGILGLA

VFIQIMHLPQSAPVVGGLVLTLMTLPTIIISTRAALKAVPPSIRDAALGIGASRMQAVFHHVLPLAMPGI

LTGTILGLAQALGETAPLLLIGMVGFIASNYPGSVDAAFNAPNSAMPAQIYEWAKRADPAFYERAWGGII

LLLVFLLLMNFLAVLLRRRFERKW*

>gkv_569|gene_pstB|phosphate ABC transporter, ATP-binding protein|

MNNPKIMARNVQVYYGDTHAIKDVNVDIDDRTVTAFIGPSGCGKSTFLRTLNRMNDTIASARVEGEILLD

AENIYDPKVDPVQLRAKVGMVFQKPNPFPKSIYDNVAYGPRIHGLAKNKADLDDIVERALRRGAIWNEVK

DRLHSPGTGLSGGQQQRLCIARAVATEPEVLLMDEPCSALDPIATAQVEELIDELRATYSVVIVTHSMQQ

AARVSQKTAFFHLGNLVEFDDTTKIFTNPEDPRTESYISGRIG*

>gkv_570|gene_phoU|phosphate transport system regulatory protein PhoU

MSEQHIVSAYDRDLETIQALIFKMSGLVEDAIGRSIEALSTRDVELAEQIRAADKQIDALEEKINDEAAR

TIALRAPVSKDLRIILSVLRISSSLERIGDYAKNIAKRVTVLAEQRAITESDATLRRMAREVERMLKDTL

DAFVQRDATLAQEIIGRDTEIDQMYNALFREFFTHMLEDPRNITACMHLHFVAKNLERMGDIVTNIAEQV

IYVTTGNRPEEPRTKEDETPFIGKVD*

>gkv_571|gene_phoB|phosphate regulon transcriptional regulatory protein PhoB

MASQLPHILVIEDEPAQREVLAYNFEAEGYRVSTAPNGDSALLQLAEEPPDLIVLDWMLPGVSGIEICRQ

IKARAETRAIPVIMLSARSEEGDKVRGLETGADDYVTKPYSITELLARARAQLRRTRPATIGGVLRFEDI

TLDGETHRVTRDGNELRLGPTEFRLLTTLMERPGRVWSREQLLDRVWGRDIYVDSRTVDVHVGRLRKALM

IHGGTDPLRTVRGAGYALG*

>gkv_574|gene_NONE|major Facilitator Superfamily protein

MTDDPAGQPPRAPAENPILVVGLTTAIQAMTSYGLLSLPVASVFYAADFGLPAWIVGVQISGIYCVALFS

SLIASNMVRRLGGGRTSQIALLAMALGVACIASGLGALLLPGLVLMGLSYGLPNPAASHLLRRFTPPARR

NLLFSIKQAGVPIGGAMGGIVTAWIAHHVNWQAALCLPALLSLTLGVVLQLVHKGWDDDRQRDQRVLQAP

LRDLIRVFGFPRFKAIFASGMLLAAAQLCVSTFIVLLLVVDLQIDPITAGAGLSLLQIAGILGRISTGAL

ADFFRSGLRVLIWLALALAATTLVLVLTPAPSALLLTALLIVIGLLSSGWSGVLIAEADRCAPPAYASAA

TAALMCGTFFGVMISTTAFAGIVQLFGTYRTPFAMIAMGCIVAAGLLRIAYRADINDKEV*

>gkv_575|gene_NONE|uncharacterized protein

MTSRNGVEIDYCPTCRGVWLDRGELDKIVERSEQVVAAAPAPAAAPQPDRHRDDDRGGRYRDDRGRGDRY

DDDDDDRRDGRRGRRRESFLGDLFDF*

>gkv_576|gene_NONE|nucleoside 2-deoxyribosyltransferase family protein

VSHIRKKVYIAGPDVFFRNASEVMRKKGEIALEYGFDPSTLAEDDLDPVGKTARQFGISIGLANERKMDE

ADFIIANLTPFRGISADVGTAFEVGYMRAQGKPVFAYTNTDRDYFTRLSADYYAGAQPEVIDGVARGADG

LMIENHDMVDNLMLDTAAEESGGAFVVGAVAADADLLGDLQAFRDCLAAARAYWDARTS*

>gkv_577|gene_NONE|conserved hypothetical protein

MAIKVQQPPDPWQRTTTQHGFAADEVISCIQKSLRRGLLENAILLGWEMFLTSPEMEEMLWSRLCVIAVE

DVGLGNPGLPSIIETLYQQHMRYPRPAGDRFLFAAHAIRMIAGSVKERTSDDLVNWARRSVELGERMPEI

LDIALDMHTGRGQEMGRDYRFFMEEASVVIPEMEGKDQTWKNWIIKALDEGKLT*

>gkv_578|gene_NONE|binding-protein-dependent transport system inner membrane component family protein

MTFLSRNWPILLLLAIWQLGVSLSGLNSIVLPAPLPVLMDMLGNPGLYAVNTLQTLWTAFAGLLIGSALG

VLVACLAYASRFLAGVLTPFGLIFSSVPVVALIPIIARILGYGSTTVIAIVAIAAFFPTFVFVGKGLQQL

PRGADDLMRVLGAGRLKRFSRLVLPSAVPDMMIALRLIVPESVLAAILAEYLMGRSGLGYVFAQATSRFA

MERAFGVSLVVTLTAVLCFFLAHRAERAVKARWS*

>gkv_579|gene_NONE|binding-protein-dependent transport system inner membrane component family protein

MTRLTKLLPGILGIVIFAAGWELIGQYRLAGLTWPPLSTVLGFLGNPANHALLQRAATASFVAVGLGYVF

GVGIGFALAALVRVWRVTRPGIDRFVALIHATPGIALAPVFMVLLQRDQIPVAIAALAVFYLVYVATTSG

LEAAHRAHHDLFSVLGAKPQTRFFRLEIPAALPAIVSGLKLAVPVAFMGGIVGEWFGASRGLGLLMISAM

QNFQIPLLWSAVLLVMVPSLALYLLMTQAERLVARRFA*

>gkv_580|gene_NONE|aliphatic sulfonates import ATP-binding protein ssuB

VTKDPAISLCGVSKSFKLEGGRQLTALEGIDLSLAPGEFVALLGPSGCGKSTILRLVAGLDTATTGSVSI

EGRSPAALSKAHRLGVAFQDHALLPWLSIAQNIALPFQVAGQSVDHARVAELIALVGLTGFEHARPSQLS

GGMRQRASIARALVLQPDVLLLDEPFGALDAVTRRHMNVELQRIWSTRTLTTLLVTHAVDEALFLADRIL

VMSGRPGRVIRDLRVPFGRPRDPSVMRDPEFHRLVDELTEALEPSGAQ*

>gkv_581|gene_NONE|NMT1/THI5 like family protein

MPKIFTGMLKTMTTALPLSRRSLLALGGAAAAGFSLPRMAIAQSMPVVSTALGWIPNAEYAGLWVAIEKG

YFAEEGIEIAYTPGGPNAPGVLVRLAAGQADFAGGDWIPLFETLNRDNDFVVLGAAFPANPAALMSLSAN

PVLTPADLVGKRILSQMPADKNTIDFILTKAGLPLDYEMVPTGFSPEPLLAGDGDVYMAFATNQPITFEK

MGMVAGQDFHVTLLRDLGYDVPAGPIVAKRDYVAQNRPLVVGYLRALLRGWIENGANPAYGAELGATKYG

VDFGLDLDQQIRQSELGQPLTAVAGAPGPFWFDPALYETNILPVAAAAGLTGLPAASDLIDLGPLEEAIA

SL*

>gkv_582|gene_NONE|helix-turn-helix domain, rpiR family protein

MNHGNRLFSGLPAESTVARAIQNALPAMSEAQRRFAALVQAEPLRVARLSINDAVSGADVSVATANRFAT

ALGYAGYPEFRADLIRAFEDFFVPVERLKRRQAEKRSAMDIAQAAFAEDLESIGATASSLDSASLEAAVQ

QIIAARRVFVAGFDLSAHLGGMLAIGLVMTGCDAQTVPSGGGAVGAVRTLTRMGPQDLVITIAFPHYYRD

TIDMAGFAKGAGIPVLAITDSPRSPLVPLAQVALYVTAKQELNAPSPSSAAILSLIEALVATVASQRPEA

AEASERFASSAYPWMTNR*

>gkv_584|gene_glf|UDP-galactopyranose mutase|

MKKCLVVGAGLSGAVIARQLADAGQFITVADSRAHIAGNCHTARDADTGIMVHTYGPHIFHTDDREVWNY

VNAFATFMPYQNRVKTTTRGAVYALPVNLHTINQLFNTALRPDEARAFIAAKADITITDPQSFEEQALQM

VGREIYEAFFKGYTNKQWGCPPSALPAAILKRLPLRFSYDDNYFCQQFQGIPKDGYTAMVARILDHPRIA

VRLNTHVRRDEIKGYDHIFYSGPIDAWFGYTLGRLTYRTLEFERFYHDGDYQGCAVMNYADLEIPYTRIT

EHKHFAPWEQHARSVLYREFSRACGPGDIPFYPTRQSRDKDLLHSYAALAAQETHVTFIGRLGTYRYLDM

DQTIREALDCSRAWLKGAGQRPTAFHLDPA*

>gkv_583|gene_NONE|hypothetical protein

MIRRLDQDGGFAGAFDHQSPLMHADATATAYENVICNFGAI*

>gkv_585|gene_NONE|hemolysin-type calcium-binding region

MPTFTLQIRTPAAGYDVVDNDQGKLSTYSDAIIVNTDSNVFSSAAYRSALDVYREQTAGTNPSYDQYLTY

LIDLLPQFGVNTTAEDFTQQHGPTFPAGSFVFFGHTWSSSPGGKDFDVMEVLLLDPEKLTSQINYVAPVY

GFIGDIPDAGEPFQVMHNTHQAGYLPDKPPISFDISRYMLAPCFTAGTFIETDRGDIAIEALRIGDLVKT

IDNGLQPIRWIGSSRICSNALSSNTKLRPIQISADALGAGVPAHDLVVSPQHRVLIRSKISDRMFGAAEV

LVPAVKLTALPGIFTDNSCDPVEYFHILFDQHEMVRSNGAITETLHTGPIALRSLSSAARAEIFAIFPEL

AALGVPRPLARSTPAGKDAAALIARHLKNQKPVQIGL*

>gkv_586|gene_NONE|hypothetical protein

LHGRLRYLGLRSHQPISTRDRGLNASRSEHKCEVANPRSLGYDSGARHAPTAIGACAFDS*

>gkv_587|gene_NONE|conserved hypothetical protein

MNALRVDFVTGAVAYRLRSGGGKSQPIARALGFRAGQDMNVVDATAGLGRDSFLFASLGANVTMIERSAQ

MYALLRAGMDEARAAGPEFADIINRMTLLHGDAMQLLPGLSPDVIFVDPMHPPRRSSALVKLELRQVREI

VGFDEDAADLMRVALAHAKKRVVLKWPRKGDAMAGIPAPSHQILGKSTRYDVFITKRGPGV*

>gkv_588|gene_NONE|hypothetical protein

MPAVPTTNKFRNRSVSDCARDSLLIAMQCTGCKQQRYYWASDLVKVLEPFHEAHVPPWPCAACRTGEWMV

MRWMFPYPELMQRIGKIRRPVGRVTKWI*

>gkv_589|gene_NONE|chain C, X-Ray Crystal Structure Of Protein Atu5096 From Agrobacterium Tumefaciens. Northeast Structural Genomics Consortium Target Atr63. domain protein

MARLFKPNEVVDRLGNYEPQPEIYPDQLAPIVRAEGDQIILQTARCGLPTPETYLEGHAVDRGVTNIRNT

SSPHWRRWLGTAHRCLVPLTSFAEPAGKGKGNVWFHLADYRPAMFAGLYVPD*

>gkv_590|gene_NONE|chain C, X-Ray Crystal Structure Of Protein Atu5096 From Agrobacterium Tumefaciens. Northeast Structural Genomics Consortium Target Atr63. domain protein

VRKKADGETTDNLFAALTCDPNATIVRIHPKAMPVILTQRAALRTWLRAGLNEARALQNPIKDEF*

>gkv_591|gene_NONE|hemolysin-type calcium-binding region

MPYLTAVNGAVVNLSLPTPILTLPSTNILNAFGTSTLFSQYNVDGVGDGSTPETVQGGDYLAPIIGGSPV

PGTYAGSGTFQTAGLTVGNAFLGATVRLNPVDVDYFVDENDQLYIISDAPLDAANLTVTITVNALGTSTP

LTLPLTDLLTNPIVAPVLGLLGGPNAVNNILNQVINSQTFDPNGTMTIPPGEINDIVCFVAGTMILTPDG

YRMVETLQVGDLVMTKDNGAKPVKWVGVRKLSAAEIIVNQHLRPIRIKAGALGVNIPSQDLMVSPQHRVL

VRSKIAQKMIQSDEVLVAAKQLLQLGGIDIATDLTEVEYHHFLFDQHEIVFSNGAETESLYTGAQALKGV

GAEARREIFALFPNLLDQEKAPIEARPMLTGRKGRRLAMRHMQANRPLVV*

>gkv_592|gene_NONE|putative lipoprotein

MRRSLMMLTWLIALSALASCGERIEAGCAGWRQIQVAGATVDYLADQDPQALRALIGHQDFGVASGCW*

>gkv_593|gene_NONE|putative membrane protein

MTAVWARIIMRYLSGALVSAGFISADFGAQLATDAELHGVLVMALGAVLAVIAEWAYRLAKRFGWAT*

>gkv_594|gene_NONE|carboxypeptidase

VLGTAYWETNRTMLPVEEAYWLSDAWREKNLRYYPWHGRGFVQLTWKANYQKASAKIGVDLIGDPSRAME

PDAAAQILVHGMIGGWFTGKKLADYIDGARVDFVGARAIVNGKDKAAEIAAIATAYLAALPEDQGSIWLR

IFKAFWGIITGKKQ*

>gkv_595|gene_NONE|type I secretion target repeat protein

MLMVFSQNPLNANQIAALSFQAVAFDTLTGQAVVAPCFTRGTLIMAMSGMVPIEDLRAGDLVDTIDNGLQ

PIRWIGSSTVSGAALKATPKLRPIHISAGALGEGLPSQDLRVSPQHRILVRSKIAVRMFGAAEILVPAVK

LVALPGIYSVDECDSVEYFHMLFDRHEIVLSNGAESESMHTGPVALRSLSSQARAEILSIFPELEQIGAA

RELARPVPSGKDLAAMFDRHVKNTQPIQRPLV*

>gkv_596|gene_NONE|hypothetical protein

MSDQSPLRSPEFWGGVAVALIVKVRTTQQLGAWQVISTLIVAVGAAWLATDWVSAMTNTPKAVAAAMLTL

TAEGIMRWILIAVNDPKQAIELWKAWRK*

>gkv_597|gene_NONE|hypothetical protein

MLALGFGLTALAALAGARAGAVPVISLASSSGYAGAELVSSVEGQWYADGVAIPGAWGGALMITPDLEGA

AIFLRVDPHIWVSDGDPGYAGSSYQSSVFGQWQADGVDIPGAVGLRWQMTPAYEGAAISLDYRPIIRIVQ

GAGYAGSRLRSNLPGQWFADGVAIPGSIGRDLIISPALEGAAISQDAEVTIMQSNTIQMWVPERALTAPQ

KANGGLWLGEDRTTVERNGADYVTAWRDKFGVRDMTQPTAANQPRAGTFRGMPAVIWDKSPAYQYLQPPA

AFAPMWWLILAEFATGVEIISGTSTGSAIYTQILGNGQAALARVSFQQPDAVQSGTSAIRLNAADTEVSS

GIFPMPMGSMSFGVSASASWCIGRGFDANNNRQWVGPILGAIALGVVPDLATRHLIEAYMHWRHGLEERL

PANHPYRNAPPRVQ*

>gkv_598|gene_NONE|hypothetical protein

MADAFQHHAVGMDSPASNAASITPSNTDDLPHVPRALYALGEGNVRVTMRGGGDPVVLPILVGVPLPVRV

SRVWASGTTATGLVGIW*

>gkv_599|gene_NONE|hypothetical protein

MTQKYWTGAEAAIIAAEAAATALVTGLPEYRDGQEVAPEARVTARWAEPRETATPGTFAIPAYPGMDVPE

GCAEADGVSLPKVMEDELG*

>gkv_600|gene_NONE|hypothetical protein

MIELDFAKIRALRIAGRDVTELRRGAVLMWAKPPEISLASGSGYAGSVYAATQPGGQWFADGVPIHSATD

QTWVMTDAYEGAVIQYDIAIQPQSVEISITSGAGFAGSVYSASRGGGQWYADGLPIPGARGQTWTMTIAL

EGAAISYITFTAPRSNRIQMWTPTVLAAALKEGWWSMRRGVQLAADDRVAAIADSFGLRDMLQTSASLQP

RTVVQMGRRVMTFAQEEQTYLLAASSHYGRYVYAVAQYKTGVETIFASYATLWGMIGSGAGRVRGNRDTN

GLQQTPLVRMNGHAPTAAVLPMAGAVLGTAPHRNSEALRAWGIGAGQSASFGWDGLIAEVIALSNEPSAD

DHDRLSGYLAHSWGQADSLPAAHPYKSLGPRID*

>gkv_601|gene_NONE|hypothetical protein

MAITTVTVTGELRDIAGAPQNLSLIRFTPRGWDKSGAAIITGAPIDVTVTGGAFSASLFRQDLGQGVVYD

VAYVLPRERITTIGSIFIDGPGPFALADLLGVPVPFGVTVTLVEGGSWPPPADPNPLHWYARVK*

>gkv_602|gene_NONE|galactoside O-acetyltransferase (GAT) (Thiogalactosideacetyltransferase)

MNELQKAAAGLLYDANYDPALLAKRRAAKRILFEINNLHPDEDEKRTQLLKGLLGKTGQNITFDGQFHCD

YGFNIEVGENFYANVNLVILDGAKVTIGNNCFIAPNVGIYTAGHPLDAERRNKGLEYAHPITIGDDVWIG

AGVTVLPGASIGSGSVIAAGSVVRGEVPPNVICGGNPGNVIREINERDSQKYR*

>gkv_603|gene_NONE|hypothetical protein

MQQEVPFTLYRLDTGEIAAFVVADPTISVPAGFGLLQGYWSAATHWVRGMPVELPPRPDDRHVWDPVAWD

WVINPDLDTYQWAQLRMERTRLLAACDYRSQPDYPQSDEARAAWLAYRQALRDLPGNLTDPAQVRWPDLP

G*

>gkv_604|gene_NONE|hypothetical protein

MTRIWTLVLTVVALMSVAAPAAADPVTGWLAVNVFGWAGATIGAFLASAVVSLGTGILGAALTGVAAQQG

QNYDVKFDVEFGDDTALTFTAGDFATAGKRRFIAKWGRETRFITEVIEISSLPQGFAGAWVNDERADLVA

GKVGTVVTGLSLFGPSSIRSLPAHDIGAVPATHTVVGWPLSNMKDDEGEGDLGARIWVKWIDGTQTAADP

FLLWAFGEDADYPWTANHIGTGKTYVIVTTRFDSETLTSYPTYLWEPEPLPLYDPRYDSTAGGQGAQRWG

QRATYQPSRNAAVISYNIARGIYWGDEWLFGGKNMAQWRLPLAEWMAAMNACDMPVALAEGGTEPAYRAG

LQITVSDEPLGVMEEIGKGANMRYAEVGGMLKPVVGLPAAPVFAITDADIVISEGQSLTPFAPASQTFNA

ITATFPDPTAKWASRDAPEYISEAGVAADGGRHLPTSLSYPAVPYPHQVQRLMRAQLEDYRRDTIVEFSL

HPGAYALEPLVDTISWTSARNGYDAKQFVVEQVTKLPGMNVTVRLREVDPADYDWSPSFELPYDSVAPVP

EIPWVQAIDGWTAVGDEVADDAGVGRVAAIRVGCAGDAIGIAQARIQARRLGASEPTFDVMRPYDRPYQW

RITGVAPASVYEVRGALMSELTGGYVWSGWITVTTPAIQMQEGDLPDGFVARIEEMAAAQGIQPVDALPD

AGARADQLVMIRTTGEIWRWDAAAGVWTQNVFAGVSAASLDKTKFAAGLTVPEVVDVLPATGSVGDMLVL

TTDQKIYRWDSELGAWSNKTDGGDIVVNTLTGAAFMAGAVGAREIATGALRAHHVLITGGSLVPDYLYQD

LGTPVGQGGRSWFWNAAQGVVFQQRFVDTQNGNNYGPQGVGIQLNTAPSTVTGNPWAWVLSGEVFPIKSA

TSYSFELGYWVSGGSRTLFRITYLDRDGNYVGELGHIALHGAAWINRFSVSGTSPATAKTAKLEVYIDPS

YGRPVLNIGSAQLLERNAVLLIVEGGIQTQHLTSQIVTADKMAANSVTAANGAIADLAVNTLQIAGNAVT

VPAYAYWEPSSPTFVTNSADYPLLELTVDRRGLATMITANAQLDGSSTDMRIVVWLLRNGQQVGGSYGYG

GAWRQSSVINFVDWDTGQGPTTYTLMARTVVHASNVYQRYLSAHQFRR*

>gkv_605|gene_NONE|hypothetical protein

MVQKGSLTRLPDWRARLSHALDIQRDHPFEWGRHDCGLGLAAGAVEAITGEDLRPAWANYKTPTGALRVL

RKAGYESLGDAMAALLPEVHPAFAQIGDLALLDGEGQIGALGVIDISSVIVLDKTGHARVPRDQIKRAFK

VG*

>gkv_606|gene_NONE|hypothetical protein

MRTYDAQFAASLTAARDGGIAPVYFFWVLARPRAGGAEVPIGLWSGDEDITLTLTQPDGSNVSRHYFGGV

NLKVEDLTYVGDLTDNPVAVSTSQGVDAAQLLARGYDLRLAYCEIHATTMTGGAFTAAPQLQWVGIIDAG

PINTPGENGEGGITYQIRSEIMWQLTARNPAKSSDAHQKRRNLIDRFCEFAAVIGSRSVQWYKKDR*

>gkv_607|gene_NONE|hypothetical protein

MIDGFYSVEFETVLGSGGGVVVLEDGSLRGGDSKRYFLGSYRIEDQKLLADVHVGTHMDKLDIPPVFGVN

ELDLKITGKLTASAAIEGTARSPQRPDSVMVFNMKRISG*

>gkv_608|gene_NONE|hypothetical protein

MTLPDLYPLAFLGDLIATRAEVSLKLQRYDEMSGGGDGRRWAAELAPPLWTASFDLNNLSRDCIGRARST

DARFRALGTNRAFLWADPTYSGPAIGAPAHLVNADVRVAGFSADRTRITFSGLPAGFEFAAGDRFSAPWG

TGRYYLGEIADGGAVAGGSVQVAVYPYPPLSLQVGAQIELLRPVCRMFVPDDGYTPYSYRRGSAATGASV

TMLEKR*

>gkv_609|gene_NONE|tape measure domain protein

LSEKQFTQSIVRLEGRLAKMERDATTKFQRQNATLTRSFKDMETQIGRSLNNIKARAGSILAPLAAALGG

RQLIAMTSAWTDITSRVNNAAGSMDRGTDVMERVSDMARRTYSDLRMTADSYISFASVLGDLGVATNTQL

DFVESLNNALVVSGAKGDVASRVMNALSNAMALGTLQGDNLNTVIASGGRVAQALAASMGVTTLELRKMG

RDGKIGRAELLGITSQMAKLRDEAAAMPATIQDGFMLLNNALLEYIGRGDDAVGISARIADALTIIADNF

DTVADKGLKVAAVLAGAVLGRSVAALAVRFGTGAVEIMKYVTAMRAASSVGGLAAALGGMSAAAGPLGIL

LGGVVAGGLVMYSDAALKAEGRTQRVSDELERLGLVAPPAASAIDAVGSALDGLDGRLANLRDLREELEK

VSTVDLQSIVDRAGSAGGLFGGWGRGGEEKDALSAVRDLAVEFGALRISADDVIRSLESIDTSSFTEDGR

EIISLLANHVRATSAMRDAIAQSGMSDILSDQLEAVRELDRYLKGLQYDDIASETIAQLMAVRVALQEGT

MSAYEAQQALQDIGEADPNVAPFLGRIAQMTGALADLIRTADRASAAAALASDPSLAARAAAATEYGASR

SEGQRIEREAADYVTEANRRNGLTREALALENEIASVRRQSLSDEVQLTEAQIRHIAAGNLAAQDARREQ

GRSGRRRDDTPYTDAVKSIREETQAFQIEAAAILSVADGTRDYGDALEFARKRAELLHAAQQQGRAMTPE

LMAEIDALAQAYVTAGLGAEEAAEKLGKIKEASDTGRATLEDFFGSIIDGSKSAREAVADLLMQIAKVQM

VKGMMGLIGSTSWGGGLINAIGSGLSYDTGGYTGPGGRYQPAGIVHKGEVVWSQDDVSRVGGPAAAEMLR

QTAGIPGYSEGGLVGMRLPAMSSLTTADHAGPQSMSVAITVDVSGAQGDAAIEERAAVGARAAMTQVLSD

YDKRFNVKVRNAMVERRKTR*

>gkv_610|gene_NONE|hypothetical protein

MGFAPEQVQRMSLWEFHSAFAAWKRFNGIKTEGGGEVTLDRLKALGVK*

>gkv_611|gene_NONE|hypothetical protein

MAEAQVINWTCGEHAFRLRIGEAEALDDLTPQGIADFRFRCRQGIERGSLGFSPVRVREVIDCIRLGLIG

GGMEGDAARALALRAMEEADFAELVKICYGIVTGFFSGKDHDQPEKPVAAEMTDENG*

>gkv_612|gene_NONE|hypothetical protein

VRVVPQCHLRAAARTKPLTTLRSPDRLHFCLQHEEIAKMAAPFHEEYHELVFEFSEDNGTTWARNCVIMG

ADVTRTASTSETETVDDCDDESKPNNVSVRVQSLAVSFSGTGNWTQGGYDTFLKKFYAGDSTEMLARIGN

LNAGAGEIEYETGPIIITSLGQSRVKGAVVSASVEARFAKTPTRSLKVGS*

>gkv_613|gene_NONE|hypothetical protein

MSSPATELQDAIEAAVRADAALMAIIAGIYDRVPDRPWGAANGYISFGPWDSVSQEGGCQAIEDVSLQLD

AWSNRTGRAHCEEIMQRLRRIMGQVQTDRHPIVARGDPFSQVLRDPNGLTLHGVLRYEFQMERYDG*

>gkv_614|gene_NONE|TP901-1 ORF40-like family protein

VRRKLRTHAKAAIEAGRSQARKEGEEIASLARAFAGASDGDLAASIRVEEADAVMTSQGRSGFIGVVVRA

GNDATIVTNKQGERFQNAKLQEVGTQSMPAKPFFNPAKRLRRKQAMAAIRRAVRKAWKEGG*

>gkv_615|gene_NONE|conserved hypothetical protein

MSTAGKLIERVQFESPDMRTGIWTHEYECRAEFIYARGGEAVYAARLEGRSVFKIKIRQCAAAREILQSW

RMIDMRRATYEGDVPQTGVYNIREVDPISDRAWIYLLVEAGVAYG*

>gkv_616|gene_NONE|uncharacterized phage protein

MDIQQTSEATGVIITLEDAKKHCRADDFGDDDALISLYIDAAVDWVQSVCQTRLERAEFTATGSRFDLGF

AGYPDPEITSVGYVDDLGAAVTLDASRYALRDGRLIVYGAENVASARVVFVAGLGPGNVPARLVQAMRML

VAHWYLNREAVGAGLAQVPLGVRDMVATYRSFAFG*

>gkv_617|gene_NONE|chain G, Bacteriophage Hk97 Expansion Intermediate Iv

MDLEIKEALDNASKTLAEVKKAQVDLSDQLKVLDQKKASGEDITDIKGHVEDSRKGLTELGDQVADLTKK

LSHRGADLEGKSLGRIIAEHDEFKTLKDDRKARFEVKDVTTGSFGTIDLPGGVRRGSRGLIQPVNQALFL

RDIIPTAATTAAVIEYLQESGYTNNAGVVAEGAQKPQSELDFEPKAAPMVKMAHFFRVTEETLDDVDGME

AYINQRGLYGLQLKEEGELLNGPGTTGRIDGLLANSTTYDSGLVPGITPVNAMDDIRIAIAQVAEADLLA

SAIVMNHLDAAALDLAKDADGRYLHPAFAGNTAWGLPVVRTKGLPQGKFIVGGFIGNTLIWQRKGIEVRR

STEDRDNFVKNLVTILLEERLQLETLRPEGIVYGDLTAAGGE*

>gkv_618|gene_NONE|phage prohead protease, HK97 family

MAMEFKLARLDAKAATEEGVFVGYASRFNVVDQGGDMVRPGAYAKSIAGQKSVKLLWQHDAAQPIGVWTS

ISEDEKGLRVEGRLALETVKGRETHALMKMGAIEGLSIGYRTKDADIIDGIRHLKEIDLWEISVVTFPME

DGSGVDAVKSSVQIMRAAKDGDFAPLKKSVEVALRDAGLPVWLRKAIAARAPEALGDGQRDASASETAKA

IKDAFKF*

>gkv_619|gene_NONE|phage portal family protein

MRIAEGVASLPITVGKLGHDRLGRAIRTPIRAGELTERLTLAPNDWMTPTEFVETLTMWAVFRGVGRAYI

HRGYRGRIRALIPINDGGVTIRRDYDTGKVWYDANIPGLGFLSNLTRRDFIEVTCPRWNEIEGLNITAEI

GKVLRLALTLEDRQMEDGRKKAVPGYITTDQQLSKDSAKMVKEALKDKLPNTPVFDSGTKYNSIIPTQAE

LQLLETRRFIIEEVARAYGIHPIFLAHDAAGQSLTRISDAMDYHVTITLGPWVKRWEEAIRFSLLGADEY

VDFDETQFYRMDLAARADYAAKSLGNNAAWETPNSILEWLGKNPVEGGDALPAAGGAPITE*

>gkv_620|gene_NONE|phage Terminase family protein

MSSSDLTTDYANRVLSGDIIAGKFVRAACQRHLNDLREGAARGLNFDIDEAARAFRFFPAMFTVTAGAKA

GEPFHLLEWMQFVVGSLFGWRNADGTRRFRQAWIETGKGQAKSPLMGAIGLYMIGFNGVPRAEAYAIAND

KDQAKVLFSDAVALCRAPIPGKDGATLESVAKVKIRGVGDNAWKIEVPESGAKFLPVASADSISGPKPIA

VFADEVHEMRTDKAIQLWKAAIDKMPGDPLMILGTNTPAADQAVGTDYSEFYQRVALGMIEDDSAFSYIA

RVDVDDDPFSDESCWVKALPALGVTYPIDNVRRRVETAKHMPSERLATERLYFGRPVGSSGFWLADEAAW

RAVLGPVAEDDMRGTPCVLALDLSKKNDLTALSAVWRDDDDQLTAKLWYWTTEGGLERREAEDRTPYKSY

IAGGDLIAVRGEVIDYTFVAQQVKELCVAQEVEALVVDPAYVSDFIAACEQISFEVWRYMGPDEPEGTGL

KIVTHAQGTRIAFEGRQLCMPHSITHMTDKILKREILIAENKMTHVCAANTVLISDGQGNQAFDKQRQRG

RIDGMVALAMGVGATKADRKGRRSYMEGGVLFT*

>gkv_621|gene_NONE|hypothetical protein

MAAKKPQMPRYDAVFRGDEARVSLAKLLWKEVIAALEEVSALSRVNLARADRYVRAKVEFEALYPEAAEA

GPVTRGPNGGEVFSFTWSAVEKLNDRMLKLERAMFGEAQARPPAEKPKKGSAPADEFLGSYNGLRQ*

>gkv_622|gene_NONE|hypothetical protein

MVYGFVGYTRSEVDYVTTGAINLDETVSLDGVTAGFGGEYAFNDSWALRGEYAYSGYGKEELIAPAGGRV

TNASIELHTISVGVTYSF*

>gkv_623|gene_NONE|prokaryotic transcription elongation factor, GreA/GreB, C-terminal domain protein

VSKTKLPRVVISDATMAVLERLAEGLERRNPELAEHFVDELSRAKVVKSAALPVDTVDLGSTVTFRDETT

GKSQTVTLALPENADINEGRISVATPIGVALIGLSAGAKFSWLANTGTKHELLVEDVHR*

>gkv_624|gene_NONE|putative membrane protein

VQKFFISSFAMLINIGVVVGAIFVVIGAISAFAQTGNLFAPIAMLGVGFVGIVGGAGTLYVLLGIYDSTR

ATYELLAEQARQK*

>gkv_625|gene_NONE|hypothetical protein

MPERVCIVSVRRSDGADLVAGHAGMSLGEGQEYVRADLLNVAIIRAESAGARIRAALVKGELGE*

>gkv_626|gene_NONE|hypothetical protein

LDLGDDQGLDVGQGDQITAGFWADLLQALNIDPMKLPARWAGKQARADVAAWLGMGFSPDQVIEVAKASR

QQMPQAPNGPKALDARMAAAASAKPAGGRASMDEMAAFWASKITAPGYIAPSAISPALARHMLQREMIAP

DQLRARGISF*

>gkv_627|gene_NONE|endodeoxyribonuclease RusA family protein

MTHLGTIELAWPVNALSPNARPHWSDLAKAKKFARMDAWLLCRAAKIPAQRADASLHLRFTFHPKVKRAR

DLDNLLASMKAAIDGIRDALGVDDSRFSFAMCMGPVVRGGKVVVDIIAMKRADETRRVPPPDHPVLGYPG

*

>gkv_628|gene_NONE|hypothetical protein

MAWLSPTPEELDACVAEHRILDREIQQLRRQAAATGPAPVWLRSQEEMV*

>gkv_629|gene_NONE|UPF0335 protein ORF1

LSIPMKNTEADINVAEKVAGVAADEIRSIVARYEALEAEKKYVAEQMKEVMAEAKGRGYDTKVLRKVIAL

RKRDQNDVAEEEAIFDLYKQAMGM*

>gkv_630|gene_NONE|hypothetical protein

MTKDFTITINVTNKPKIRSAIYIAGAQVGVIGIGIVTQSVAMQWVGFCFLILWLIGATKAWQTKNSDLTI

DQAFVRLSQIQREADN*

>gkv_631|gene_NONE|MT-A70 family protein

MTAFPIEQYDLIMADPPWSFATYSAKGQGKSADAHYATRDLRWISELPVASIAAPDCLLWLWATNPMLPQ

ALTMLRYWGFTFKTAGHWSKRNLVTGKLAFGTGYILRCAGEPFLIGTRGNPKTTRSVRSVIEGARRQHSR

KPEEAFTAAERLMPSARRIELFSRQERAGWDVWGDQTDRFPAPAAMSQPEGVA*

>gkv_632|gene_NONE|hypothetical protein

MSALPTRQDDELILRALAMRVRGISLSEVGDILGVGKSTIGMATQAVFEADLRESGERAAVVDRGYRWPK

HKGARR*

>gkv_633|gene_NONE|hypothetical protein

MSILYAHPDGLMSAQDLRETLVRDHIRASRVTLARPRGFFDISLHRDCEVDGAALADWALRRPANGPSAP

AASLAS*

>gkv_634|gene_NONE|acyl carrier protein (ACP)

MNGFTNTEARLIFLIAKDFHIAAEILPSTSLRGDLRMDSLDLVDLCVKAEDLFDIEINDAEATEATSIAD

LARLIDHVLAEEAAA*

>gkv_635|gene_NONE|hypothetical protein

MSRVSFALSIAPMILGIALLAWHAWPTIQWARALSPAAGPHMTCATDPVINCRLPSAETGQ*

>gkv_636|gene_NONE|hypothetical protein

MSAAMIHELESLIARSKRLGTWSHTASSVYGSEMTKPHALILAAPVELPHYDQAPPFAVAIAGSLVAQPD

QLLSEIADQLNAVPVIAQYCLDLLREKESGR*

>gkv_637|gene_NONE|acyl carrier protein (ACP)

MTVSDAQRGLADILEELFGLLPERTAPTAHLRHDLGLDSLHLIELFMEIEVRFGIEISDAAIESVQTVAG

LSCVIEHLVTQKRNAA*

>gkv_638|gene_dnaN|DNA polymerase III, beta subunit|

MTDTVSAIIDRHALRKAMTSLQRTVQQWAAIPALKYISISSGLGEVTLRATDLDNLLTIKLEAETTGTVP

FLVSAEVLQKFASLAAGPVTVTRTPDIEGKDALITITDQETTLRLRERIQHDDFPTVPAWDMKDAARFTG

SGAELSRILDLSRHCVSTEETRYYLNGIYLITAPERSTLRAVATDGHRMAVIDSNIEAPNLAGVIFPRFA

LDVFRGLLDPKSNTPIKMQFEENRGIIEGDDWILHSKMIDGTFPDYTRVIPKHETNCEAHFTRAIVTKAH

RLSQAVRGHIAAAATITSDGKMHLMHEGEDESVSVPVGASPNFDLGRHGFNVKYLNKQAQVTPEFTLRAS

TERPNDPATIVSDDPDACWILMPMRAA*

>gkv_639|gene_NONE|hypothetical protein

MMRDHIFDALTPNATSERNLREILRGGYCTRWHANADMAHIRETLAEHHARVAQIILALHPSPSAALLDA

ALHHDAGEPRVGDVPWPAKRDNPALAQAIDEVERAARERLGIHINLSTIDKAYLKLSDRLAGYMHVQHTA

PHLLAQRDWLEDRLAIELQAAQLGVAPAVSRLIEGAS*

>gkv_640|gene_ssb|single-stranded DNA-binding protein (SSB) (Helix-destabilizingprotein)

MSGSVNKVFLIGNLGRDPEVRSFPNGGKVVNLNIATSETWRDKNTGERKERTEWHKVAIFNDGLANVAEK

YLRKGSKVYIEGKLETRKWQDQSGTDRYTTEIALRPFFGQLTLLDGRSQDDNRHDGEGYSSGSYQRPDDT

AQRAARDLGDDEIPF*

>gkv_641|gene_NONE|hypothetical protein

MTSRPFLAEAHACRIHGLIKQTGGAITVAQLSDRLGIAPITVRDIVQARGWTRRIGRKAPIIHLVDPEVD

VVELHFD*

>gkv_642|gene_NONE|phage integrase family protein

MSEYRVGRLRGGYCVTWTDPDGGRRRYKLDATTPTEAEAEARSIFHQAAAARALTVSDIWSAYRKDKAGR

RIDHHMIDTGKTILPVFGALEPHQITTQDCRDYVEKRRAIGRKDGTIRTELGHLRTSLSWAEKHRMIEHA

PHIERPAMPSPKERYLSRAEIDRLLSVDGDPHIRLAILLMLTTAGRVGAILELTWDRVDMIRGQINLRLE

GEGPRKGRAVVPINNTLRAALVAAKAHAMSEFVVEYAGGQIGSIKTGFRNACAKAGLKGVTPHVLRHTAA

VHMVEAGVPILEVAQYLGHSNPSVTFSTYGRFSPDHLRKAADALEFGKLRSVQ*

>gkv_644|gene_NONE|hypothetical protein

MTQTKPPASSRDARLKAALKSNIARRKAQMKARAASAEEDEAPEPTSEDQPDQ*

>gkv_645|gene_NONE|conserved hypothetical protein

MTEDASFADGDERPLRLIAHETADLGVISALVQDAILPASEMVWQPRQRRFALLLNRFRWEDPLNANGRR

RRAERVQSVLHFDDVVKVRSQGLDPAQKDLVLSLLALDFTPGADGTGTLTLTLAGDGVIALDLEALNITL

QDVSRPYRAPSGKVPRHAD*

>gkv_646|gene_hisD|histidinol dehydrogenase|

MPLHLNTLDADFEQAFTALLGMKREDSPDVDAIVAGIISDVRQRGDAALIELTARFDRLSLTPETIRFSE

AEIDALIAKVPPIEAEALELAAERIRTYHAAQMPQDARWMDDAGAELGWRWTPIAAAGLYVPGGLASYPS

SLLMNAIPAKVAGVKRLAVTVPTPDGVANPLVLLAARLAGVDEVYRVGGAQAVAALAYGTATIPAVDKIT

GPGNAFVAAAKRRVFGKVGIDMIAGPSEILVIADGDNNPDWIALDLMSQAEHDASAQSILVTDSPAFAAA

VETSVAKLLTTLARAEIAGESWRDFGAIIIVRDMAEAVQLSNRIAPEHLEICTADPEALLTQIDHAGAIF

LGQWTPEAIGDYIGGPNHVLPTARSARFSSGLSVMDFIKRTTIARMTPEALRAIGPAAEVLAASESLQAH

GLSVTARLKALNEE*

>gkv_647|gene_NONE|uncharacterised protein family (UPF0262) family protein

MNRLIAITLAEGGHASHQALQERQVAIRDLLEENAFAIPPRPDYPLPAGPYRLHLALLDRRLHFTVTSES

HDPTVAFQLALGPFRQVFKDYAQICGSYHSAVKSMPPARIEAIDMARRGIHNEGASILQERLHGKAELDA

ATARRLFTLVSALCEDVL*

>gkv_648|gene_NONE|low molecular weight phosphotyrosine protein phosphatase family protein

MKAPLPASILFCCDQNSVRSPMAEGIMKKLYGRDCYVQSAGVRSDLEIDGFAIAACSEIGVELSRHRVRS

FDEMQDWGDDLSSFDLVVALSPASEARVLELSQYFHMNVEYWPIIDPTNAGESRETRLAAYRDARDMIIQ

ALTDRWGSPAMAEQPL*

>gkv_649|gene_infA|translation initiation factor IF-1

MAKEELLEFPGVVKELLPNATFRVELENGHEIIAHTAGKLRKNRIRVLAGDKVQVEMTPYDLTKGRINYR

FR*

>gkv_650|gene_maf|septum formation protein Maf

MMGLRLILGSASPRRAELLGQLGIAADAITPADIDETPAKLEMPRDYVARMSREKLAALPARDDAVTLCA

DTTVTMGRRIMGKPENAAEAASFLYAMSGRRHRVITAISVGRGADSWHRVVESVVKFKVLSDAEVNAYIA

SGEWAGKAGGYAIQGRAAAFIPWISGSFSAIVGLPLSETAALLHTAGYRAADTIIANGE*

>gkv_651|gene_NONE|ribonuclease, Rne/Rng family

MKGRRIVLDHYQGREAAALLVDGRLDDLLIDSDQVRTGAIFRAICDRPLKGQGGMMLRIPGGTAFLRQGK

GLHPGQAMLVQVTGIAEDGKAVPVTDRVLFKSRFAIITPGAPGRNISRQIDDEEERDRLAAIAHEAMEGA

DEGTFGLIIRSSAAGADEDDIYDDIREMLDLAVAVMADAEGTEPEALTEGDGPHDLAWREWSAPDIETIP

GGFAREGVDQMIEDLAETRVEIGEGTMYVETTRALVAIDVNTGGDTSPAAALKANLAAARALPRALRMRG

LGGQIAVDFAPMSKAHRKQVEQSLRASFKLDPIETSLVGWTTMGLFELQRKRERLPLAQLLEGANGQ*

>gkv_653|gene_NONE|UPF0243 zinc-binding protein CC_2340

VAEYKPFCSARCADVDLGRWLGGNYRIASEDNDEQEEALQELEKLIDSEFPPDGTTRH*

>gkv_652|gene_NONE|AMP-binding enzyme family protein

MVAFASVADRAAIEAHTPWPPADFPATSWQMVQRAADQFGARRGVTFQLLSDPRARAETLTWSQVRDKVG

QTANLLRSLGVSEGDTVALLLPNCTEMVLSYFAAQTAGIVCPINPLLEPEQIASILRETGAKVLVTLKSM

PSSDVAQKAALALVLAPEVETVLEVDLARYLPFFKRIIAGFMRPKLDVQHQAKVVDFTRAIAAQPVDPVF

EVGTHDRVASYFHTGGTTGLPKVAQQRFSGITYNAWVGAHVLFTERDVMMCPLPLFHVFGSVVVLGMATA

SGAELVLPTPAGYRGKGVFDNFWKLIARYRATFLITVPTAISALMQRPVDADISSLKLAISGSAALPVEL

YKRFEDAAGLTICEGYGMTEATCLVAVNPPNGPKKIGSVGIAVPHTKIRVIDPVTQFPCAVGEVGEICVQ

SPGVFPGHTYTEDARNADLFYPGADGQPLWLRTGDLGKLDADGYIFITGRSKDLIIRGGHNIDPAEIEEA

LAAHPEVAFVGAVGQPDPHAGELPCAYVELVRDATITPTELTAFARKHIAERAAVPRHIEVLDELPKTAV

GKIFKPALRKLAIQRVLDEAFKHAGLAATVALVEEDRKRGLVTYIARGDGFDEDAFRALMGRYALVWDWA

PAS*

>gkv_654|gene_NONE|ABC transporter transmembrane region family protein

MARGAPASDRPASRDMGALRALWPFLRPYRSQLLAALGALVLTALVSLALPIAVRHVVDGFSAENVAVLD

GYFLLALGVAGLLAVGSAARFYLVNRLGERVISDIRKSAFARMISMSPTFYERILTGEVLSRITTDTTLI

QSVIGGSVSSALRNLIMLAGGLVLLFITAAKLTLLVMLVVPLVVVPIVLLGRRLRVLARENQELIARSAG

QASEQLLAAQTVQTFTHESASRDSFNTTTEAAFQSARNRVSTRAAMTAVVMFLIFAGVVCVLWIGAHDVR

AGRMSVGELVQFVVLAIMVAGAAGALTEVWGELQRAAGATERLVELLETEDPVVDPVQPVSAPDLRDVPI

HFDDVTFSYPARPDAPSLNQISFTIAPGETVALVGPSGAGKTTIIQMLERFYDPQSGAIRLGDTDLRDMA

RADFRAQMALVPQDPAIFAATARENIRFGRPTATDAEVEAAARAAAAHDFLMALPEGYDSYVGERGIMLS

GGQRQRIAIARAILRDAPVLLLDEATSALDAESEAAVQKAVEQLARGRTTLIVAHRLATVKRADRILVFE

QGRLVAQGTHDSLVAEGGLYARLARLQFIAADAA*

>gkv_655|gene_glyS|glycyl-tRNA synthetase, beta subunit|

MADLLIELFSEEIPARMQTRAADDLRRLVTDGLVEAGLTYEGAGAFSTPRRLVLTLSGLTAQSRAVREER

KGPRTNAPEAALQGFLRATGLTVDQLDVRGDTYFAVTEKPGRAAPEIIAEVLTSAVRNFPWPKSMRWGSG

NLRWVRPLHSIICLLDDQVVPVDIDGIVAGNTTRGHRFMAPGVITVTGFADYEEKLQAARVMLRADQRAD

TIWTDATNAAFAKGLEVVEDKGLLAEVAGLVEWPVVLMGPIGEAFLGLPPEVLQTSMREHQKFFSLRNPA

TGRIEAFITVANILTADDGATILQGNGKVLAARLSDARFFWENDLRTVSRAGLEGMAAGLANVTFHNRLG

SQTDRIARIEAMARALAPVTGADADEAALAARVVKADLRSEMVGEFPELQGTMGGYYARAAGLSDAVANA

CKGHYQPLGPDDAVPHEAVSATVALADKLDTLTGFWAMDEKPTGSKDPFALRRAALGVIRLVLGNGLRLP

LRDTLAAPLAANFAASGTAGETAPVAENLLGFFHDRLKVFLKDEGLSHDVIDACLAMPGNDDLDLLVRRA

RALQAFLKTDDGENLVQGFKRANNILTQAEAKDGVEYSFGADPAFAEDEVERALFAALTRAEGQITPAIA

AEDFAGAMAAMAALRAPIDAFFAGVQVNAENAIVRRNRLNLLGQIRTICLQVADLSRLVG*

>gkv_656|gene_NONE|signal transduction histidine kinase regulating citrate/malate metabolism

MKSQTMARLMLGGMLAFALIFGAGMYYTQVYAYYNRLTPEEIGPVTLLRNDGTAAELSVTALQAIDSNSS

PLRFRACFAATTPPVEIAANFAPYPGATPLVGPGWFDCFNAREIGEALEAGAAQAYLWQENVHYGIDRVV

AVMPDGRAYAWNQMNRCGEVVYDGEAAPEDCPPAPEV*

>gkv_657|gene_glyQ|glycyl-tRNA synthetase alpha subunit (Glycine--tRNAligase alpha subunit) (GlyRS)|

MTDTRAPRSFQEIILRLQNYWAAQGCAVLQPYDMEVGAGTFHPATTLRALGSKPWAAAYVQPSRRPTDGR

YGENPNRLQHYYQYQVLVKPSPPNLQELYLGSLDAIGIDTRLHDVRFVEDDWESPTLGAWGLGWEVWCDG

MEVSQFTYFQQVGGHDCKPVAGELTYGLERLAMYVLGVDHVMDMPFNDPDAPIALTYGDIFHQTEEEYSR

HNFDAANTEKLLKHFEDAEAECKALLDQPAIDPKTGKRIIMVHPAYDQCIKASHLFNLLDARGVISVTER

QAYIGRVRTLAKACADAFVLTGAAGYVPEEAAQ*

>gkv_658|gene_NONE|putative peptidoglycan binding domain protein

MLPRLRVALHHLVFAAMLCISAGSATAQDSTRAYIQVEAQPTLAMAQQRVRAYAGALPDVSGWALPSGWY

AIVLGPYSRTDAAIYLDQLMTEAQAPNDSYIVDGAQFRQQFWPIGLGVASTPLPIPMNSGRPVNVPPPGP

APVTPLPVTPQPINPQPAPTAQAETVEQALAAEGALSNGDKRLLQSAMRDAGVYGGAIDGLFGRGTRDAM

AAWQAQNGYSPLTGVLTTDQRAALMAQYNAVLDDLGIERTVYDMAGIEIDLPTAVVNFQEVTPPFARWQS

DDGAYAVLLISQPGDRARLASLAATLQSLPFVPAGADVQVSATGLSITGASGDSRIRIEAGLHDGAIKGY

GLIWPAADPAFDRLFARMQQSFRAIPGVLAAPAAAAQADTALTAGLSLDAPRLVQAGIYIDAQGLVLTAA

APLAQCSRIALNGDTPARITQTAGEMALLQPELRIAPTAVARFATPQDGAAITGAGYPYGHTLAQASTTP

GQITALDGLTGTADHLRLTMQATAGDIGGPLLDAGGDVVGMLLPQTAGLPQTVQIAASSGAIARLINAPA

PLAVISAPIAPEAVSTRARLITAQISCW*

>gkv_659|gene_mtgA|monofunctional biosynthetic peptidoglycan transglycosylase|

MSAQKAKRPARKSAAARPARGKLPWRAWLVRGAKYAAMGFGGLVALFLLLVLLFAFVRPPTTPYMVAESF

RQGGVRHEWVAMDRIAPAMALAAVAAEDANFCTHWGLDLDAIRFAIDSRLGGASTISQQVTKNVYLWHGR

SWLRKSLEALMTPVIELFWSKRRILEVYLNIAEFDRGVFGVEAAAQHYFGTSAANLTNRQAALLAAILPN

PKERSPLDPSAFVNRRASSIMDGAATIRGTARAACFS*

>gkv_660|gene_NONE|glutathione S-transferase, N-terminal domain protein

MTRLYHYPLSPFCRKVRLCLGEKRIEVELVEERYWEQSPDLMRRNPAGKVPVLKLENRFLTESTAICEYL

EDIVPTPALMPQGAEAKYEVRRLIGWFDDKFFNEVTNKLLTERIFKKVQGGGYPDSTRIKEGARAVKYHL

DYMNWLLESRRWLAGNDMTLADFTAAAHLSCLDYISDVDWNRSDLVRDWYAKIKSRPAFRSLLADQIPSF

LPAPHYADLDF*

>gkv_661|gene_NONE|conserved hypothetical protein

MKARLKAAALDAGFVAMGICRPDSIRKDGDGLRAFVADGMHGEMQWLEDRIDWRADPTALWPQARSVIML

AESYAPTYNPLDALEDPARGAISVYAQGKDYHDLVKKRLKRVGRWLIEQAPGSEIKVFVDTAPVMERPLA

REAGLGWTGKHGCILSRDFGNWVFLGCIFTTLDLEPDQPVRPSCGSCTACLDICPTQAFIGPGRLDPRKC

VSYLTIEHSGPVPLDLRGKLGNRIYGCDDCLAICPWNKFAVEASDIRYHGDVGNPPLDELAGLDDTSFRA

RFSGNPIKRIGVNRLLRNVMYAIGNSGKPHLRASAQRHLNAEDPVLRDAAEWAVARLS*

>gkv_662|gene_nspC|carboxynorspermidine decarboxylase

VIETPFYLIDRTKLSRNLQIINRLRDLSGAKTLLALKCFATWPVFDQLAEVMDGTTSSSLYELRLGHEKF

GKETHAYSVGWSDAEIDEAVSYADKIIFNSIGQLTRFEAASSKVARGLRLNPRFSTSGFDLADPARAFSR

LGEWDVDKIRAVLPLITGVMIHYNCENGDFDLFDRQLTRIEDEFGEILRQLDWVSLGGGIHFTGEGYPLE

KLAARLKAFAANMGVQVYLEPGEATITNTTSLEVTVLDILNNGKDLAIVDSSVEAHMLDLLIYRTTAKME

TSGDYPYQIAGKSCLAGDIFGDFTFPKPLQVGDRLSIADAAGYTMVKKNWFNGVKMPSIVIRELDGSMTV

AREFGYADYASSLG*

>gkv_664|gene_NONE|saccharopine dehydrogenase family protein

VKKNVLIIGAGGVAQVVAHKCAQNNDRLGDLHIASRTKSKCDAIIQSVADKGAMKVAGSFTAHSVDAMDT

AAVADLIRATGAQIVINVGSAFVNMYVLEACIQTGAAYLDTAIHEDPAKICEIPPWYGNYEWKRRADCAA

AGVTAILGVGFDPGVVNAYARLAADDYLDTVESIDIVDINAGSHGRWFSTNFDPEINFREFTGTVYSWQN

GAWQSNKMFEVGKEWDMPVVGTQKAYLTGHDEVHSLSARYPDADVRFWMGFGDHYINVFTVLNNLGLLSE

KPVKTAEGLEVVPLKLVKAVLPDPSSLAPDYTGKTCIGDFVRGTKDGAPAEVFIYNVADHEDAYAETGAQ

GISYTAGVPPVAAALLVADGTWDVKTMANVEELDPKPFLNLLNRMGLPNRIKDANGDRALEF*

>gkv_663|gene_NONE|hypothetical protein

VFPPSLTRAISFPICTGKMQKIMGAGQRKRPPKPGAFVA*

>gkv_665|gene_NONE|conserved hypothetical protein

MFGVAAAGFSRGRIDPPTLVGMSAILMWSATVGLYRNISEIFGPIGGSALIFTVSGIVALIHAGPKAFRG

HSPRYLLIGGAMFVTYEIALALAVGFAQTRAQSVEVGLINYLWPSFTIALAIWAGHARAGIMVIPGILIC

LLGVFWAATGSANFSLAAMVHNIGSNPLPYILAFIAAITWPLYTILTKGMAQGRSAVPLFLLATAALLWV

YYGVSDQPTLVYNSKGAMMVLTFGVLTTLAYSAWTYGVNHGNLTLMATASYFSPLLSVMLSSVLLSMVPG

LNFWLGASLVTAGSLICLAATKR*

>gkv_666|gene_NONE|RNA methylase family UPF0020 family protein

MIPVDEIFLVTAPGLEDVLADEARALGLNVTGVIPGGVTVAGGWPAVWRANVKLRGAVRVLARIASFRAM

HLAQLDKRARKIAWDQLLRADVPVRVEATTVKSKIYHAGAAAQRVETAIRETLGAPIAQDAPITIKVRIE

DDLVTISVDTTGESLHKRGHKLAVNKAPIRETLAALFLRQCGYSGQMPVLDPMCGSGTLVIEAAEVAMGL

PAGRARDFAFEQLAQVDRAALAGLKAGGRSTDLRFYGSDRDAGAIRMSRDNAARAGVGEITMFDVKPISE

ITPPEGPAGLVIINPPYGARIGEKKTLFGLYGAMGKVLTERFKGWQVGIITADSGLAKATGLPFLPAGPV

VANGGLKVQLFRTDPL*

>gkv_667|gene_NONE|endoribonuclease L-PSP family protein

MKALEPTSIRPPFGQYSSGVSVAAPQQWVVTSGQLGVGPDDVAPQSVLEQARICFANCGAILAEAGLGPQ

DVVRVAGFVTAREDFAAYMQARDEWLADAPVKPTSTLLIVTGFTRAEFKVEVEVMAAR*

>gkv_668|gene_NONE|creatinine amidohydrolase family protein

MPHQYWSDLRATDFNASLHDAVALLPVGATEQHGPHLPLNTDSLLAEEMARLSAAHAASATVLILPTIAV

AKSDEHIHFPGTLTLDGATLLAVLEQIGASVARAGIRRLVCINAHGGNVPVLQMLVRSLRIKHDMLAVTA

GWIGMGFPEGAVSPREQAEGIHGGLVETAAMLHFRPDLVDMTQAQHFVPASSAVAAQNTVLRMMGNVTTG

WCAEDLHPAGAAGDAASATAALGAELVTHSAQRFGKLLDEVAAHPLPKGAA*

>gkv_669|gene_NONE|amidohydrolase family protein

MNRVLKNARIPAALAEGLSGRDLGNGLIELDLPIAGGKIAPASLGAPQDLGGKIVLPCFVDAHVHLDKTY

TAQRAGVSRTGLDEAVGLAMQDAPNRTAADLDTRMERAAEAAYRAGTVALRSHIDSMQAPNDNPGWQALV

RLQQRWAGRLTIEPVALMRIERAIEDSFADRCAQIAATGGLAGGYISGQGCDPAMIDRFFEQAAGAGLGV

DFHVDETADASARGIESVLDAMTRTGFAGRVTLSHCCKLSAQSPSVAQPIVERMAALGVHVISLPLSNAF

LMGRAPGQMSPLRGMTRVQELAAAGVPVSFASDNVQDPFYAYGAYDMFEVMRAGLLIAQLEGDAGHWLQA

ITRTPASAMGLQAGVIGYGRPADLIIFDAYDWADLFSRAHENRTVLRAGAPLPQRTGQ*

>gkv_670|gene_NONE|FAD binding domain protein

MNLDAFRAAIDGIEVEDRPHIVRQKSRDFFWYSPILKRQLDGFVGDLVVSPKDEDEVIRILAAAYAHDVP

VTVRGGGTGNYGQAMPLQGGVVLHTNKLTGITALHEDSICVRAGTVIEHIEHHLRENGREIRLFPSTTAS

ASIGGFIAGGSSGVGAIRWGGLRNPANIRRVRLVTMEAAPRILDLTGDDIHKAAHAYGVNGVMTEIELPI

DPAVDWVDVMITTPDFISANTLAISLGEDEGVLLRMLSTFAAPIPELFFQRFRPFVGVGRGVIAAMVRRD

DLPAFESHLQNWPTAELVYRADLADPALRLPAVFEMAWNHTTLRAIKTDPSLTYLQMMFPRDAMAQTITA

LDQHFGDEIMLHFEYTRFSGVVSPVAMPIVRFTTEERLEALIAELERDFGITVFNPHRVTLEEGGMKRTD

ISQLEFKRQTDPKGLMNPGKMIAWTHPDWQPEAGKSFLFQE*

>gkv_671|gene_NONE|flavodoxin-like fold family protein

MKVHVIYAHPQEDSFNAALHRTVVDSLIEAGHEVDDLDLYKDGFNPVLSLADRESYHDVPANRALVDDYV

RRLESCDALVLCHPVWSFGWPAILKGYIDRVFIPDVSFKLRDGQMGPGLMNIKKLATVTTYGSKKWRAWF

LGDPPRKNGTRFLRVVCNPLVKVSYHALYDMNNVTRADTDAYLGKVRQAMLKF*

>gkv_672|gene_NONE|ABC transporter family protein

VTHSATDTEKPDLATLALRFDDVSMQYPDGTIALEGIDLTIRKGEFVSVVGPSGCGKSTLLKLASGLEAH

TGGQIRVDRSNLGYTFQDATLLPWRTVLPNVELLMELRGIPPEERRRVALEQIELVGLKGFENHYPKRLS

GGMRMRASLARSLALNPAVFMFDEPFGALDEITRERLNDELIALYLRNGFTGMFITHSIPEAVYMSSRVI

VMSRRPGRIIADFPIPFAYPRQPELRYDPEFSRIAGEVSVALRHAIEE*

>gkv_673|gene_NONE|binding-protein-dependent transport system inner membrane component family protein

MTEIAPKIRTEAYAPTPVAPPRTPAQKFAATFLPPLIMGVLVVLLYWVVRESLPAHRQFLMPSASGMWDK

ALSQPAVWAELGSRSLTTLTIALTGLAFSIPIGMALGIIMFRFFVMERAVYPFLVALQSIPIMAIIPLIQ

SALGFGFMPKVLIVILFTFFAIPTTLLLGLKSLDQGVLNLFRLQGASWWTMLRKAGLPSSAPALFAGFRI

STSMAVIAAVTSELFFMAGRGGLGQMLVNAKTDFKYEQMYAALIASATLSISIFVVFTLVGNRIFASWYE

TAERKS*

>gkv_674|gene_NONE|hypothetical protein

MNRILTALTASVAAFAGTTASAGEFATAHYTTPLADVCPSPFYIQKDWLAQAEHGGLYQMIGAGGTMESG

AYRGPLGATGIELAILEGGGGIGLGDGETAYSALFNGNSKAGVIPHLGFQELDNAYIFSNLFPVVGVFVP

LDIAPSGLIWDTGTYPDGFHSVDDLKAFGESGAGMIYVSTITRTFGLWLVEQGVSRDAFVEGYRGDLENF

VANNGTWLNQGFVTTEVFNLSNGMNWAKPVDAVTVNELGYPTITGMVSVAQPRLEELAPCLELLVPIMQQ

AAVDYINDPAEVNQLIADFTAGGFSASWWRATPELNAYSAAAQRDRGIVGNGNNATIGDFDLDRAAAMLE

LVKPMLDDRANPDVTVDDVVTNRFINPEIGL*

>gkv_675|gene_NONE|tetrahydrofolate dehydrogenase/cyclohydrolase, catalytic domain protein

MALLLDGDALAAKLRQQMTERVAASGIRPVMATVLVGDNPASESYVARKHKDCREIGIEALRIRLPAGAS

PEQVLAEVARLNDDPSVDGFFVQFPLPEGHDEQAIAAAIRPDKDIDGLHPENLGRLITGKGGIPPCTPMA

VLSLLRGYNVPLAGKHVVIIGRGLLVGRPLAMVLSAPGVDASVTLLHSQTPDIAAFTRNADVVIAAAGHP

ELIRADMIRAGATVVGVGITYGDDGAMVSDIAADVSAIAGAVTPAHGSVGSLTRAMLLQNLINLALEKHS

HARN*

>gkv_676|gene_NONE|bacterial transcriptional regulator family protein

MPATDENRALFEDDKEISLTLARGLDLIEAFAGDERRLSIPELAARTGMNRTVVRRLVRTLEKKGYASAD

RGQYELTPHILRLIRGFIEGRSLPQIVHPLLRAAAEDIGESVSFAMLDDTEAVYVAHAFLPARFTLNMVT

VGSRAPLLPTAVGRVIVAFLPDIERSAILSRLSPQAHTPQTETDAARLDAIFADCRRLDYCMADGEYVEG

VASLAVPVFDGMRRVTGALSIIFPTHGHDATEIAEKLAPRMQATASALGSALQ*

>gkv_677|gene_NONE|tetrahydrofolate dehydrogenase/cyclohydrolase, NAD(P)-binding domain protein

VTTIFTGFDLAADILQGVRADIATLGRAPVCVTLFDDSSAPARAYLNRQITLARGAGIDLRPMGYADAQL

AQLAADARVDAIATLYPLPSGLTPMGAAQAIGGGKDIDGQHPNHAGPLLLGDGTLRPAATAQASLICARA

ILGDLAGAEIVLIGASRLIGRPLAMLLLDAGATVTTCHIQTRDLARHTRAADLVISAAGVPALLTADNIA

KGGRILDLAIIPKDGSLVGDADLPSLMGHAALVSAVPDGVGPVTTACLFANIAAAAKSRAMNLPLLQQD*

>gkv_678|gene_NONE|hypothetical protein

MKKLIASFAIAASIAAPVFADTEVRPGVFFAGTVETAGSDRAVMQDLIFDLATAWAVCDRDAMANAITDD

VSFSYPTSAVNGREAIMADLEAFCGAATDTSLYFPADAFYIDVDTGRIAAEVQFRTFQRGNRQVVNDVWI

ATVTDGKVSVIKEYLDGRVKDLQAQGVLQLEESPDFLTPWPPRTEAWASCFPIVRAAPTNDCVQ*

>gkv_679|gene_NONE|conserved hypothetical protein

MKPATALDDQTSAAEMRQGMTWVLLDMALVSAMTVMVKKGGVDFPAVQMVFFRSLVGLVAVLPLVLRHWR

VIRQTRNVKRNVFRVTCNAVALSCNWGALTILPLATANAIGFLRPLIVMVMAIFLLSERVTGWRWAGAAL

GLMGVGVMLLPSLTGMGEAQDHLLGYAFAGGAILFGAMATIQTRALKGENTTVMMVFYTVGLTLFTAIPA

FFVWQPVALHHLPHLLGIGIIAQVAQYCYLRGYQLAPASKLAPLGYLSLIFATVMGYVFFDEVPTVYTAG

GAIVIIIGLIVARRA*

>gkv_680|gene_NONE|glcNAc-PI de-N-acetylase family protein

MLTDRNRLFRRIADPRMVRLARALGRLGSTVTMMNTGAHPDDEQTELLAWFSFGRNMRVVIACSTRGEGG

QNALGPERGAALGLVRSRELEESARVIDADIAWLGHGPVDPVHDFGFSKDGKDTLERWGRARVIDRLVRA

YREYRPDIVLPTFLDVPGQHGHHRAMTEAAEAALALAADPTYEVDGLAPWRVAKYYLPAWSGGGSTYDDE

LPPPPATVDVRVEGFDPVSGMRYDQIGEASRGYHASQGMGTWRATPRRHWALHGTAPEGDILDGLPATLG

ALADVAGAPAELALAASEIAKARAAFPDDQAMITALVAAHKAFSAPMGADFDALHGHRIRQKIAEVEAAL

ALAAGVDVAAWLQDPLVPGRSATLAVWVNAGHATLRGIAAAASDGIAQKGGSEERDGLHLLTLAVPADLS

PASRYLPGWARLGGNGILAASVTVEVGGITFALPVDLEEEPLLQPAASVTPSADAVLVNLNDPQPVHFAM

TGTASAASLGLGSIDGLTVENADGHVTLTASGLAPGKTRLPISVAGAAGWQAKPINYGHIGRLAQVVPAG

VDILALDLKIPDGRVGYIGGGADRVGLWLERMGVDVVDLDAEAFDAARANGFAGFDTLVVGIFTFGLRPD

LAAATADLRAWVHAGGNLVTLYHRPWDNWKPDETTPAHMVVGSPSLRWRVTRPGAPVTILEPDHDLLAGP

NTITHADFDGWDKERGLYFLSSWDQVYQPLLAMSDPDEQPLLGSLVTGRIGKGRHTHTALVLHHQMDRLV

PGAFRLMANLIQPA*

>gkv_681|gene_NONE|ROK family protein

MVSAMTTAEGATEKRKRAIGANPERNRAHNRSLVLNLLREHGQIGRAAMARHTRLTQQAVGNIIDELLLE

GMVIETGRLRVGRGQPARQFALNPCGPVSLGVEIAAGHLAIVFQALTGAIRARSIVPLADTAPAPVIAAL

VAQIEKLKSEAGAPEIIGMGVVMPGPFEIEGISAVGPATLKGWAGLDPAALIADATGIGTVVYENDATAA

AVFESLHGVGRGLRDFCHVYFGVGLGLGLIHDGRPLRGAFGNAGEIGQIAVPPRGGGAAAALEDRASVFA

LRDFLRETRGAPDDLDLLASLDPAEDPALQDWIARAADQLSPVLAILENIFDPETITLGGLMPRPIIEAM

IDCLQPLPVTVSSRSARGLPRLMLAQTGPYTAALGAAAMPFMDQNTTATLRQ*

>gkv_682|gene_NONE|bacterial extracellular solute-binding family protein

MRKLSKIAALASGVSFLSVGAASAVEIEYWQYVFDTRVAAVDQLITNFQAANPDITVKHVTFPYADYPTR

VVAGTMAGQGPDVVQFFYGWLDNFIAQDLLQPLDPAVFPAAEIEADFFPIVSAMSRDGSYYGLPTAVRSL

ALFYNKDRMTAFGLDPNSPPTTLDELIAQAAASTERDGAGNYTSIGLTVEMGGQDHHWWREALVRQFGGV

PYDENGDVAYTSDEGKAAFRWYTELNTAQHVGAPGFMDEGQAAFRAGLATFTIDGTFRLGGFAANPFEWG

VTELPANAAGERGNYSSYFANGIGADVTGEELEAAQKFLAYLSSEEAMTVWLDVVGELPARRTTALSEEN

LANPIYGPFLRGLEYAHTTRFYDESGQRQIMLDAANRVFLEGLSPEEALDIAGQAEQAIINDLR*

>gkv_683|gene_NONE|binding-protein-dependent transport system inner membrane component family protein

MSVTDPNGAAEGRPGLWNRLGIRTKHVLWAWAFLAIPVLFYVVIRFYPTFDAFWLSLTDGNIRRGPSFIG

LENYARMYADPVFWKVFGNTFLYLLIGTPVSLVISFTIAYYLDRVRFMHGLIRALYFLPYLTTAAAMGWV

WRFLYQPVPIGMINSFLTSIGLEQQPFLRSTDQALMAATIPAIWAGLGFQIIIFMAGLRAIPSSFYEAAR

IDGLGEWAILRKITLPLLKPTTIFLVVLSSIGFLRIFDQVQSLTANDPGGPLNATKPLVMLIYQTAFSSF

RMGYASAQTVILFLVLLLISLLQLWLLRDKK*

>gkv_684|gene_NONE|binding-protein-dependent transport system inner membrane component family protein

MSASTELAANRRNIRPGRVIAWTLLILGGFLMALPILYMFSTSLKPASDTFDLRLIPAAPTLANYIDILQ

DGRFIRWFYNSMIIAVAVTASNVFFDSLVGYTLAKFDFRGKNIVFIAILSTLMIPTEMLVIPWYMMSAKL

GWLDSHWGIMFPGMMTAFGTFLMKQFFEGVPNDFLEAARVDGLNEFTIWWKIAMPMVLPAISALAIFTFL

GNWTAFLWPLISTTSPDLYTLPVGLNSFAVGEAVRWERIMTGAALATIPTLLVFLALQRFIVRGVMLAGL

KG*

>gkv_685|gene_argH|argininosuccinate lyase|

MSNPNDPRLTDGSVFPDPVYKETVLRPLFDGAKTHHVAAFGAIDRAHLVMLAETGILPAADAGKIAVARA

ALDTEIDPATLTYTGEVEDYFFLIEKELKARVGAELGGRLHTARSRNDIDHTLFKLGLRARLNLLIEQAI

ALHGAIVAKAEAESATLIVAYTHGQPAQPSTLGHYLSAMAEILARDIQRLFEAYRIVNLSPMGAAAITTS

GFPINRERVAELLGFAAPLQNSYSCIASVDYITSTYSAMELMFLHLGRPIQDLQFWTSFEVGQIYVPNAL

VQISSIMPQKRNPVPIEHLRHLASQTVGRAHSMLTIMHNTPFTDMNDSEGETQETGYQAFEVAGRVLTLL

AALVAQIKVDPARVASNIRRSCITITELADSTVRREGLSFREGHEIAAAVARAVVAAEGDLTTDGYAPFV

TAFKHATGRDPQIDAAAFAQITSPEYFVAVRDRTGGPAPEALAQAISGYKTQNAGFAAQLATLIATQSAA

DADLATAFNLLKESA*

>gkv_686|gene_NONE|ABC transporter family protein

MAKIELEGLVKDYGKVRAVHGIDLQIEDGEFVVFVGPSGCGKSTTLRMIAGLEDISGGALKIGGKVVNQL

EPKQRNIAMVFQNYAIYPHMTVGQNIAFGLYTSKLPKAEKDRLVREAGETLGLTPYLDRRPAALSGGQRQ

RVAIGRAMVRSPSAFLFDEPLSNLDAQLRGQMRIEIKRLHQRLGTTIVYVTHDQVEAMTMADKIVVMRDG

RILQVGSPLDLYENPVDVFTARFIGSPSMNVIEGESDGVNLRLGNSTLPGFGANLPAGKVMVGLRPHDLK

VGVPGDATLEAVVTAIEPLGAETLVHMEVAGQPLVGSAPGRVLPVVGSTVTASVTRGVLYVFDAQTEKAL

GRA*

>gkv_687|gene_NONE|hypothetical protein

MTSKNNGEGVLSLGRIYADLAFAELDAPPPPAARSMPKALA*

>gkv_688|gene_NONE|pfkB family carbohydrate kinase family protein

VITAAHLVAAGRPAHLLARLGTDPIAVAIASELTALDLDLTYVERAADAGPQLTVAIVTPEDRAFITRRS

PRGMPSQAAAALHGAGLRHLHIAEYATLAENPALIVTAKMAGLTISLDPSWDESLIHGPALLAASSGVDV

FFPNMDEATALTGKTAPAAALDILAQHFPVVALKCGSAGAMLAVGSTRFSVTAPKTVVVDTIGAGDSFNA

GFLDAWLSGLAPEEVLRRAVQRGSQSVMAAGGTGCLSQMKSAS*

>gkv_689|gene_NONE|cation transport family protein

VKTAVKGWAARFLSLPPPLVVAGIYIATITMGASLMMLPMAQAMPMRWSDAFFMATSAVTVTGLAVVDVG

SHLSLLGQAVLVTLVQLGGLGLMTFAVLILEIVGRPVGLMGEAYLREDLKQNALWRVGRLVRRIAVVVFA

IEAVGIAILCLSFIPDLGFWPGLWAAIFHGIGAFNNAGFSIFRTGLMEYVADPIVNLVIPALFITGGIGY

FVLHDLIYKRRWRYWSLNTRIMLAGTAVLIPWSVLMFAALEWTNPATLGGLDGIWPRIAASWFQGVTPRT

AGFNTLDISGIHDSTAMLFISLMLIGGGATSTAGGIKVTTFVVMILATIAFFRRQTQLHIFGRGIGPDEV

LKVMAIVAVSLVLVFCGVFLLSLSHDGHFLDIAFEVASAFSTTGLSRNYTPELNDFGRCVIMVIMFIGRL

GPLTLGFFLATQLSPRVRYPQERIHIG*

>gkv_690|gene_NONE|trkA-N domain protein

MARTEQSFVVIGLGAFGAAVASELARFGNRVMGIDLDERRVAQMVSVLPTALILDATDEIALREAGVDRY

DVALVAIGQNIEASILATMNLRLLGLETVWVKAASRVHHRILVKIGADRVILPEQEMGRHIAQMLNNPVV

QDYVSLGNGFNVVSIELPKALDGATPKSLGLVGREEPRLMAAMRGTQQLDIANPDLRFAPNDKLILLGRR

VVLQAFSDGL*

>gkv_691|gene_NONE|hypothetical protein

MIATSAFAQAPAMPDMDEAVAAANNQLGVLEYCAAEGHIESTAVEVQERLLQVLPPASDPTAVEAAYAAG

KEGTIAVSGTEMSLADAATGQGTDVAALCQQLGSMVEQAGASLPN*

>gkv_692|gene_NONE|hypothetical protein

MSLINQTVGLGWWSNIAMVRYVEGSMFLRATMDVPHGRRIYQSGMIRLPS*

>gkv_694|gene_NONE|putative membrane protein

MNTAPFVRIALRYIAGGLVSYGILTPEGATAFASDPQVIAQASIVLGAATAALTEGFYALAKRWGWRT*

>gkv_695|gene_NONE|carboxypeptidase

MAIDLNLGETGRILAVCRAQALDAAQTAYVLATAYWETNRTMEPVEEAYWLSDTWRRRNLRYYPWHSRGF

VQLTWEANYRKAGARLGLDLLSDPDLARDPQIAAAILVRGMVEGWFTGKKLGEYVHGTRHGFYEARRVVN

GLDRAADIAVIAECYLRAVNPAPVPWIVKIFQYLTQRKSA*

>gkv_696|gene_tyrS|tyrosyl-tRNA synthetase|

MTYTPKSEFLAVMIERGYIADCTNYQALDAALMSQVVPAYIGYDATAASLHVGHLLNIMMLRWLQKTGHQ

PITLMGGGTTKVGDPSFRSDERPLLTPAKIDENIAGMQQVFARFLRYDDSANGAKMLNNAEWLDSLNYLE

FLRDIGRHFSVNRMLSFESVKSRLDREQSLSFLEFNYMILQAYDFLELNRRYGTLLQMGGSDQWGNIVNG

IDLTRRVIDKEIYGLTSPLLTTSDGKKMGKSANGAIWLRGDMLSPYEFWQFWRNSTDADVARFLKLYTEL

PVAECDRLGNLGGSEINAAKIILANEVTTLLHGAEAAAAAEATAREVFENGGVGDDLPQLALSVDEVGDG

ISLAQLVVRSGLAKTGKDGKRLIAEGGLKVNDETTVDAGRLFTAADLEVPVKLSAGKKRHALVSIS*

>gkv_697|gene_NONE|ferric uptake regulator

MAHSILGRCEAQGLRLTDQRRTIARILEGADDHPDVAELHARAVAVDSRISIATVYRTVKLLEEAGILER

HEFGDGRARYEDADRDHHDHLIDMHSGEVVEFIDPEIEALQEAIARKLGYRLVGHRLELYGLRDSQSPEK

KT*

>gkv_698|gene_NONE|conserved hypothetical protein

LNNFRAAILLTLAMAGFAVEDVMFKLANQSLPQGTILTIVGFSGALVLAIAATIRRENVFSRDFFHPFVI

LRNVTEMIGTACFVMAITTVPLTLASAVAQALPLAVMAGAAIFLGEKVGWRRWAAVGVGFIGVMVIVRPG

LEGFNVSTLWAVAAVMAMAVRDVITRRVPDHIASLPVASWGFMFAGFAGLVIAAAQGELPVPQGMQWLPV

MTSMTAGIGAYVALIISSRIGEISAVIPFRYTRLLFAMILGALVFGERPDTWTLVGSALIVGSGLYAIYR

ERKRVREARS*

>gkv_699|gene_eno|phosphopyruvate hydratase|

MSSIVDIQAREILDSRGNPTVEVDVLLESGAFGRAAVPSGASTGAHEAVEKRDGDKSRYAGKGVLEAVAA

VNGELAEELVGLDATEQEDIDALMIEIDGTPNKGRLGANAILGVSLAVAKAAAEFTNQPLYRYIGGTSAR

TLPVPMMNIINGGEHADNPIDFQEFMIMPVAADNIRDAIRMGAEVFHTLKKELHQAGLSTGIGDEGGFAP

NIASTREALDFILRAIEKTGYTPGRDIYLALDCASTEYFKDGNYVLSGENLVLTPEQNADYLAKLVADYP

IISIEDGMAEDDWAGWKLLTDKIGDKVQLVGDDLFVTNPLRLTDGIEQGVGNSMLVKVNQIGTLSETLRA

VDIAHRARYTTVMSHRSGETEDTTIADLAVATNCGQIKTGSLSRSDRLAKYNQLIRIEEMLGAGAVFAGR

SILR*

>gkv_701|gene_NONE|conserved hypothetical protein

MTADAIIAKLGLQPHPEGGWFRQTWRSDAEDAGNRPSGTAIYFLLKGGEVSHWHKVDAAEIWLFHAGAPL

VLSIAATTQGPAVDHTLGADLLAGEAPQLIVPAHHWQAARSTGDYTLVSCTVSPGFQFENWELAAPDFDI

PR*

>gkv_700|gene_NONE|comEC/Rec2-related domain protein

VRGTAFRNALVRVIDDQRGALLPWIPVLLGAGMAAWLALRAEPGLAIYAAVVMLLIGGAALRNVPGGAVV

LVALGFLLAALRGYTLEAPVLSRDYYGPVMGRIVAVDANQAGAMRLTMDQVVLNRVAPHQTPAQVRVSLS

GRQGFFDQTPGTVVRMTARLTPPNGPVEPGGFDFRRYAWFEQLGAVGNTANPVVDAMPTAAQDLFIDRLR

MRMSGAIRDHIAGDAGGFVAAVLTGDRQGLSPAVNQWMRDTSLYHLVSISGVHMALLAAFVFALVRGVVA

LVPPVALRVSSKKLAALVALPVAAFYLALAGRDIATERAFITVAVSLIAVLLDRRAISLNTVALAATLVL

VLRPEAVLNAGFQMSFAAVVGLVVMFDTLRLARARWPGLRRWRALGWLLLPMGVSLVAGLATGPYAAASF

NRIAHYSLPANMLAEPAMSFLVMPAGILALILWPVGLGPAALWVAEQGTRWILWVAEVIAAWPYAVSAAV

TPQPAVMPLLTLGAIWIVLWRGWGRWLGAPVVVLALTLWGATQRPALLISSDGAAVAVLGPEGRVFSKPR

GAGYAASNWLLSDGEMVAQAEAFERPGFTRQDGRLVAEVGGQRILHLTTAAAVRAAADCGGADIVIAAHD

LPPITGCRVFDRRALRDSGAVAGWPTAQGLRLESVAESSGQRLWIR*

>gkv_702|gene_gltX|glutamyl-tRNA synthetase|

MSLSKPVVTRFAPSPTGALHIGGARTALFNWLFARNQGGKFLLRIEDTDRARSTPENTAEILAGLTWLGI

DWDGEPIHQFERAPRHAEVARELLAQDKAYKCFSTQEEIEAFREKARAEGTSTLFRSPWRDVPSSQHPDL

PFVIRIKAPQTGAQTVHDAVQGDVTVSNEQMDDMVMLRSDGTPVYMLAVVVDDHDMGVTHVIRGDDHLNN

AFRQKMIYDAMGWDFPVMAHIPLIFGPDGKKLSKRHGATAASEYQALGYPAAGMRNYLTRLGWSHGDDEF

FTDAQAKEWFNLEGIGRAPARFDTKKLEHLSGQHIARTEDTDLFAQLTAFLAKTGAPALDPAQEDLLLRS

MYTLKERAKTFPELLEKAHFALTCRPITPDEKAAAALDPVSRGILNELTEGLPGVSWEREALEHLVTEVA

EKHGTKLGKLAAPLRSALAGRTVSPSVFDMMLILGRDESIARLKDASI*

>gkv_703|gene_gltA|citrate (Si)-synthase|

MADQKTAKLTIDDQSFDVPILSPTNGPDVLDIRKLYGQAGVFTYDPGFTSTAACDSAITFIDGDQGILTH

RGYPIEQLATQSRFLEVAYLLLYGELPTPKQLTDFETLVTRHTMIHEQMQYFFRGFRRDAHPMATMVGVV

GALSAFYHDSTDINDPQHREVASIRLIAKMPTIAAMAYKYSIGQPFVYPRNDLDYAANFLHMCFAVPAEP

YHVDPIIARAMDRIFTLHADHEQNASTSTVRLASSSGANPFACIAAGIACLWGPAHGGANQACLEMLREI

GTVERIPEYIAKAKDKSDPFRLMGFGHRVYKNFDPRAKIMQQTADEVLALLGIENNPTLQVAKELERIAL

QDEYFIEKKLYPNVDFYSGIILEAIGFPTSMFTPIFALARTVGWISQWKEQLADPQLKIGRPRQLYMGES

LRDYVSIDKR*

>gkv_704|gene_NONE|cytochrome c-type biogenesis protein ccmH (Cytochrome c-typebiogenesis protein cycL)

MKRLLAALGLALTLGLSAAPPVYAVQPDEVLSDAGLEARARAISVNLRCPVCQNESIDESHAEIARDLRL

LVRERLVAGDTDEQAVDFIVARYGEYVLLNPRASGMNAILWGAAPAFFLVALGVAIIAIRRRSRAEVAAP

LTDEEKRRLDDLLGKD*

>gkv_705|gene_ccmF|cytochrome c-type biogenesis protein CcmF

MTVELGHFALILAFAVAILQMIVPMYGAQKGWRGWMAFGAPAATAQAALVAFSFGALTWAFVTSDFSLKL

AVVNSHTDKPLIYKISGVWGNHEGSMLLWLFILAIFGAAVAWFGGDLPERLRARVLAVQASISVAFYAFT

LFTSNPFERMVIAPLNGQDLNPLLQDPGLAYHPPFLYLGYVGFSVAFSFAIAALIEGKVDAAWGRWVRPW

TLAAWSFLTVGIALGSWWAYYELGWGGFWFWDPVENASFMPWLIGAALLHSAIVVEKREALKSWTVLLAI

LAFGFSLLGTFIVRSGTLTSVHAFASDPARGSVMLAIMGFYLGGGLTLYAARASAMEAKGVFSLVSRESA

LVMNNVLLAVAAFVVLIGTIWPLVTEVIGRTVSVGPPFFNAAFTPFMIALSIILPLGSVLAWKRGTLGKA

LQQMWGAAVLAFALMALAWVMTGGSTILAPIGVLLGTWVVAASAVDVWARTGRGDIRGRLSRLTRLPRAD

WGKAVSHIGFGATILGVALSLSLVKEDIRVMNIGDTFELGNYTFNLTAVNELQGPNYLTTMADVTISRDG

QEVASLHPEKRFYPFAGMPTTEAGISSNLLRDVYVVLGDPQVGGGYAMRSYIKPFVNWIWIGAGLMAIGG

GISLSDRRLRVAAGAAKKATRAAGVPAE*

>gkv_706|gene_NONE|cytochrome c-type biogenesis protein ccmE (Cytochrome c maturationprotein E) (Heme chaperone ccmE)

MKGLKKQRRIQIIAVAAVALALSTGLIGYAMRDGISFFRSPTQVLAEPPHENELFRIGGLVEEGSIVRGQ

GTSIVFNVTDGGGTVPVAYTGIVPDLFAEGQGVVAQGRYIDGRFEAVEILAKHDESYMPKEVVDALKEQG

VYRDDAES*

>gkv_707|gene_argC|N-acetyl-gamma-glutamyl-phosphate reductase|

MPFNVAILGASGYTGAELVRIIATHPELRIAALSADRKAGMAMADVYPHLRHLDLPRLVQTSEIDFAGID

LVFCALPHGLSQALVRDLPQHLKIVDLGADFRLRDPAEYEKWYGAPHVATDLQQEAVYGLSEFYRDDIKS

ARLVAGTGCNAATVQFALRPLIEAGVIDLDEIICDLKNGISGAGRSLKENMLFAERSEDVAGYSQGGKHR

HLSEFDQEFSAIAGRPVRIVFTPHLVPINRGILATIYLKGDAQAVYDALASRYADEPFIVVLPFGQLPAM

SHVQGSNFCHIGVTADRISGRVLVVSTLDNLNKGSSGQAVQNANLMLGLDETMGLMLAPVFP*

>gkv_708|gene_murI|glutamate racemase|

MAVGVFDSGLGGLTVLDAVAKRLPDLPLVYFGDNAHAPYGVRTSDDIYNLTVSATQKLFDNGCDLVILAC

NTASAAALKRMQENWVPEGKRVLGVFVPLIEALTERQWGDNTAPREVAVKHVALFATPATVASRAFQREL

AFRAIGVDVEAQACGGVVDAIEEGDLILAEALVRSHVEALKRKMPRPDAAILGCTHYPLMEKVFADALGD

GVQVFSQANLVAESLADYLQRHPEMVGPGMESKFLTTGDPARVSSHAIQFLHRPINFQKA*

>gkv_709|gene_NONE|bacterial regulatory helix-turn-helix protein, lysR family protein

MDWDKLRIFHAVADAGSLTHAGDTLHLSQSAVSRQIRALEEALDTTLFHRHARGLILTEQGELLFEATRS

MNKRLEAAAARIRDSEDGVFGTLKVTTTTGFGTMWLAPRLGKLYDQYPDLKIDLILEERILDLPMREADV

AIRLKEPSQADVIRKRLMAVRMRLYATPEYLQNSPPINRLEDIAQHRLISQSLNSPQPVAAINLSQRLMT

FDMASALTVNTYYGVLQGVLAGIGIGILPDYVTEDSPNLCRVLPYLESSEIPVFIAYAEELRQSRRIAAF

RDFIQDELIAHRRKLRESAPPADAVPD*

>gkv_710|gene_purL|phosphoribosylformylglycinamidine synthase II|

MTTETAFREPQITADLIASHGLKPDEYQRIEEIIGRTPSFTELGIFSAMWNEHCSYKSSKKWLRTLHTTG

KQVICGPGENAGVIDIGDGQALVFKMESHNHPSYIEPYQGAATGVGGILRDVFTMGARPMAAMNALSFGM

PDHPKTRQLVEGVVAGIGGYGNAFGVPTVAGEVRFDPAYNGNCLVNAFAAGLADADRIFYSAASGVGMPV

VYLGAKTGRDGVGGATMASAEFDDTIEDKRPTVQVGDPFTEKRLMEACMELMASDAVISIQDMGAAGLTC

SAVEMGDKGGLGIRLDLELVPVRETAMTAYEMMLSESQERMLMVLKPEGEAEARAIFEKWDLDFAIVGET

IAEDRFLIMLNGEIKADLPLSKLASSAPEYDRPWVATPPAAPLEGVPSITPIAGLTALISSPNHAAKQWV

WSQYDHQVGADTIRVPGHGAGIVRVHGTNKAVAFTSDVTPRYVKANPIEGGKQAVAEAYRNLIAVGATPL

ATTDNLNFGNPEKPQIMGQLVGAIEGIGAACRALDFPIVSGNVSLYNETDGNGILPTPTIGGVGLIADLG

DMIAGPAQAGDALIMIGRVGSHLGQSALLLEAFGRADGDAPGVDLEEEALHGQFILTNRMRIRACTDLSD

GGLALAAFEMAEAAGTGVSVAAEDTAQLFGEDQGRYLLAVPREQVAALVDAAAGEGIAAVIYGEFGGDTV

AFGADKAPLATLRATYRDSFAGKLGL*

>gkv_711|gene_NONE|bolA-like family protein

MAIEAQEIEDLIRASFPAARITITDLAGDGNHWAAEVIDESFRGLNRVQQQRAVYAALKDQMEGANGALH

ALALTTKAPE*

>gkv_712|gene_NONE|glutaredoxin family protein

MSDVALNQIRETIAGNDVVLFMKGTKMMPQCGFSSRIASVLNFMAVDFADVDVLADADIRQGIKDFSDWP

TIPQLYVKGEFVGGCDIVTEMVLSGELDTLFTNEGIAFNKESADKIREANA*

>gkv_713|gene_NONE|conserved hypothetical protein

MPQINVMIGNRQFEVACNPGEEQFVTGAAARLDTEAQAFAAQLGRMPESRMLLMAGLMLADRTGALEEEL

TALRHHVGTLEARLAQTPTRVEVEVERVVEVPVEVPVEVQVEVERLVEVPVEVISEEVVAAYEAMVERVE

ALMRAAETRFGIEQK*

>gkv_714|gene_NONE|hypothetical protein

MTQDSQQFAPLGADRIADAAARVEAALSRIAGVLQASASGPLHAGHAADTSEQLAAAEGRFAVLEQQLAA

QGSRLAEIDDELYRLRQSNAELRAVAEEMRGSLAANVADPRLINRAMEAEIAALQASRAADLAEVSAVIA

GLRPLVANSARPSHQFRAADFEEQE*

>gkv_715|gene_tkt|transketolase|

LDIAALRAAHPDHWMKAAAIRTLTLDAVAAANSGHSGMPMGMADVATVLFEKHLRFDPKAPRWADRDRFI

LSAGHGSMLVYALMYLTGYEDITLQQIKDFRQWGARTAGHPEYGHAAGIETTTGPLGQGIANSVGFAIAE

EHLRARFGTSLINHYTYVIAGDGCLMEGVSQEAIGLAGKQELSHLIVFWDNNGITIDGKVDIADVTNQPA

RFAASGWHVQEIDGHDPVAINTAIEAAKKDKRPSMIACKTHIALGSSAQDTSKGHGALTDAKLIADAKAA

YGWTAGAFEVPAQIKAGWEAMGQRGAAAHAEWTARFNALSDTKKADFERIFRAEAPKGLPAKIRALKKEI

SEKQPKVATRKSSEMVLDVVNPLMKETIGGSADLTGSNNTLTKDLGTFGVENRKGRYVYFGIREHGMAAA

MNGMVLHGGVRPYGGTFMAFTDYARGAMRLSALMGAPVIYVMTHDSIGLGEDGPTHQPVEHLSMLRATPN

TYVFRPADTVETAEAWEIALTSTSTPSVLALSRQNLPTVRKTHTNTNLTAKGAYVLAEATGKRQVILMAS

GSEVEIALAARDQLEAAGIGTRVVSVPSMELFRAQDEAYRKRVLPAGPARIAIEAGVRQSWDWLLLGERG

REAKAAFVGMEGFGASAPAEVLYEKFGITAANVAQKAKGLIG*

>gkv_716|gene_NONE|conserved hypothetical protein

MSGLLALLDDVAAIAKVAAASIDDIATQTVKAGSKAAGLVIDDAAVTPKYVTGFKADRELPMVWRIARGS

IFNKLVILLPILLALNYFLPWIITPLLMLGGLYLCYEGAEKVIHYIRPQDPVAHAEHETGDPAKLEESRV

AGAIKTDFILSAEIMVVSLSVIEVGGFWMEAVTLAVVGLAITALVYGGVALIVKADDIGAAMHQNGRLGA

TRAFGRGLVVGMPPFLAVLSFVGTLAMLWVGGNILTHGASTLGWPLVYDGIHHWAVDAGNMLSGARGFVE

WFVTAALDGIVGLLIGLIVVLVVTPFMRGKAH*

>gkv_717|gene_NONE|hypothetical protein

MTNQIALTFALLLLLLVGADMLFNHGAALLFLARKFLDLLNWIQFWR*

>gkv_718|gene_gap|glyceraldehyde-3-phosphate dehydrogenase, type I|

MAVKVAINGFGRIGRNVLRAIIESGRTDIEVVAINDLGPVETNAHLLRYDSVHGRFPGTVTTTADTIDAG

RGPIRVTAIRNPADLPWGDVDIALECTGIFTDADKAKIHLENGSKRVLVSAPSTGADKTIVFGVNDDTLT

AADMIVSNASCTTNCLSPVAKVLHDAIGITKGFMTTIHSYTGDQPTLDTMHKDLYRARAAALSMIPTSTG

AAKAVGLVLPELKGKLDGVSIRVPTPNVSVVDLTFEAARPTTVEEVNAAIIAAADGPLKGILGYTHEPLV

SSDFNHDSHSSVFALDQTKVLDGNMVRILSWYDNEWGFSNRMSDTAVALGKLI*

>gkv_719|gene_coaD|pantetheine-phosphate adenylyltransferase|

MRIGLYPGTFDPVTNGHLDIIRRGAALVDRLVIGVAINAGKGPLFGVDERVALLEVEVAAIPADIAVVPF

ETLLVDFAGKVGAQVIIRGLRGAADFEYEFPMTGMNRTLAPDIETVFLMAEARHQAIASRLVKEVSRLGG

DVSAFVPPGVQQALADRWK*

>gkv_720|gene_NONE|hemolysin-type calcium-binding region domain protein

MAEVTLNADGEGSYLLPVYFTGEDDTVAVNVTQGFRGSIVVSGHYNDGEIESLSLSLPDGWRLALRERGR

AAALEVEAWVAYDIFDAGGALRGPITISANYISVPCFTRDTWIETAGGPVLIQNLTPGDLVMTRDHGARP

IRWIGKRNLDADELETFPSLRPVRIKAGALGRGVPQDDLLVSQQHRMLVRSRIARRLFESAEVLIAAKQL

LQVEGIDIAEDVSAVEYYHMMFDQHEVVSANGAPTESLYTGAESMRAISPAAQEEIFTLFPELRDYDDTP

PPARPLASSRAGRRLVVRHLKNNLPLLSP*

>gkv_721|gene_NONE|FAD binding domain protein

MKAIIIGAGMGGLCAGIALQRIGHEVAVYERVREIRPVGAALSLWSNGVKCLNFLGLEAQVRALGGQMDS

MAYVEGHSGRTMTAFDLAPVYETAGQRAYPVARAELQNMLMDACGRENITLGAELVEVWEDESQVHARFA

DGSVASGDYLIGADGAHSLVRSYVLGEKLPRDYSGYVNFNGLVAIDPAIAPADRWTTFVADGKRASVMPV

ADGRFYFFFDVPGPAGQTVERADFKDTLRQHFADFAAPVQRLIDAIEPERTNRVEIFDITPFHTWTRGRV

ALLGDAAHNTSPDIGQGGCMAMEDAVVLGIALQVNTLGVQDALIRYQNRRAPRAGELVLRARRRAAETHG

FDMAETQAWYDGLWTEDGSRIMRGLLSNIEGNPLD*

>gkv_722|gene_nusB|transcription antitermination factor NusB

MRSAARLYAVQALFQMEAAGVTVTRVTREFEDFRFGASIDGEELVEGDVTLFRTLMQDAVNNQAQIDQMT

DRALVSKWPIARIDPTLRALFRAAGAEFMLKETPAKVVIVEFMDIASAFSADPKEPKFVNAVLDHMAREA

NPADFAE*

>gkv_723|gene_NONE|hypothetical protein

MSGIGHNGGPGNGDGFLRHCWRMARRELIGGGGTLPIEVVRARVARAKALGLDYKTYAGVRATTGRDLVA

FLYSSNALGVFRPGQVIADAPLLRLQASAAQPHLGLSGRALPGLLDQIAAKSELALPRFGTNWGEMRDDL

KAWLMAQGLPGDAVLMIGETDHERELMTAGGLAGFISGQSFLGAGAGR*

>gkv_724|gene_NONE|conserved hypothetical protein

MLKIVKWVFIVALALLVGGVLHYNLPRHDIVRIIGTENRRVTPGWNSMFYSNAEPGSAAGQVRDVFFINT

ALPNGRERVFRNEDTGWGWPPYFKFSAYDIQAQVANLASTAEAPRWVAVRHYGWRNQFLTIFPNAVKVWE

VPGPDTRIIPWFNIIFLTVSVALVWAIAARVIRWRHRRLQPALDRLDQRIDARRAGISRWFKGK*

>gkv_725|gene_NONE|conserved hypothetical protein

MSFAQPAVSRRFALAALGGFSLMAACAPVPEVAAPEPVVGGVPLSQIVEGYGLIEDEGYRLPPVSPQYLQ

GVNRRVLMRYPGELRPGTIEIDPNAKFLYWIMPDGMAWRYSIGVGIEGVGLRGTTIIQRKAKWPGWTPTA

NMLRRDPALYGPFRGGVPGGLASPLGARALYLYRNGSDTYYRIHGTNDLESIGNSGSAGCIRLFNHDMIH

LYDLVPNSTRVVIRTYADSVRIEGSAMANRGIELAPYLITPEQLFNGEEGGRADELASVSGRG*

>gkv_726|gene_NONE|cytidine and deoxycytidylate deaminase zinc-binding region family protein

MQFRSYMIHALEEAKLAAMRGEVPVGAVVIGPSGQIIARAGNRTRELHDPTAHAEVLAIRAACAVIGSER

LIDHDLYVTLEPCPICAGTIAAARIRRLYFGAEDPKSGGVLHGARVFNHPQCHHRPEVYGDLGAPEAETL

LRDFFAAKRL*

>gkv_727|gene_NONE|conserved hypothetical protein

MNQDPTTPPHEDAAPAATGERIAKVLARAGIASRRDVERMILDGRVSVNGKVINSPAVNVADRDRVSVDG

QPVAAAEPPRLWLYHKPSGLVTTNKDELGRETVFDALPEDLPRVMTVGRLDLTSEGLLLLTNDGEVKRKL

ELPATGWLRRYRVRVNGEVTEPELDKLRAGITVDDIEYQPMDVELDRQQGANAWLTIGLREGKNREIRRV

MEALGFVVNRLIRISYGPFQLGALEAGEVQEVRRKVLRDQLGLDQDAPDLQRSRRIIRNEKIVDETPKRG

RPAKPGFKPGAKPSGDRPSYGDRPSFGDKPRFGDKPRGDRPSFGDKPRFGDKPRGDRPSFGDKPRGDRPY

GDKPRGDRPYGDRPSGDRPSFGDKPRSFKPRTEGDAPRGDRPSFGDKPRFGDKPRGDRPSFGDKPRGDRP

YGDKPRGDRPYGDKPRGDRPYGDRPSGDRPSFGDKPRSFKPRTEGDAPRGERKSFGDKPRFGDKPRGDRP

SFGDKPRGDRPYGDKPRGDRPYGDKPRGDRPYGDRPSGDRPSFGDKPRSFKPRTEGDAPRGERKSFGDKP

RFGDKPRGDRPSFGDKPRGDRPYGDKPRGDRPYGDKPRGDRPSFGDKPRSFGDKPRFGDKPRGDRPSGDR

RPSGAPQGGNRRPPRRDDK*

>gkv_728|gene_NONE|putative membrane protein

MAERATLPHDMRRAHATLFAPHLLWLADRFALRHRVWLDRGLNMARAFGGKFSPRGKGESDAARAAIRDN

REEERKLSKGGKRARILMIPGFLAAGLSLFRGAEELAFGLLGGGGVILAAWLLREGLRAEAAYEARAIAR

RPAIPRKIFAAVLFGIGTLFMAVSADTSFEEGAIYGVIATVLSLIAFGLDPMRDKRAEGIDTFQQDRVAR

IVDEAESYLATISQQIATLNLRSLSAKAEGVIASARRMIRTVEEDPRDLTAARKFLGVYLMGARDATVKF

VDVYRRTSDEGARNDYETLLDDLQGQFAATTTRMLEGGRTDMDIEIKVLRERLQREGIPTE*

>gkv_729|gene_NONE|tellurite resistance protein

MSETIREAATAALKDVEKVTATVLPTPTPAAEIVPLAQADTVKSTEIRQRIAEIDMTNTQSIIGFGSRAQ

SELQVISQSMLQGVKNKDVGPAGDSLRDMVTTIRGFSVSELDVRRDQSWWEKLLGRAAPMAKFVARFEEV

QSQIDKISDDLLKHEHVLLKDIQSLDLLYEKTLDFYDELAIYIAAGEEKLAELDSTTIPAKEAEAAGAPE

DQAIIKAQELRDLRAARDDLERRVHDLKLTRQVTMQSLPSIRLVQENDKSLVTKINSTLVNTVPLWETQL

AQAVTIQRSSEAAKAVKAANDLTNDLLTKNAENLREANKAIRTEIERGVFDIEAVKAANANLIATINESL

QIADEGKAKRAAAEADLQRLESDLRDTLVSAKAREMPVAG*

>gkv_730|gene_NONE|conserved hypothetical protein

MMRPTRHRLMTWTAGLCALSLLIGCAEEQPVPEAEAPRAISPRERPVQAEVVALPSATSQALAQYYVRLQ

NDLRGRGLLRTDDGHDAPFTDTVLARNFIRVALFDEYTDLGHRFAARASESHLRRWEQPIRMQTVFGATV

PLEQRQTDRAEVTAYAARLAEISGLSITPNASDPNYLVIFAGEDDRSSFAPMLRQFIPGISDSAMLALMN

PDRSTLCLVVAFGETGQSNTYTRAIALIRGEHPQLIRSACINEELAQGLGLANDDMMVRPSIFNDSEEFA

LLTTHDALLLKMLYDPRLRPGMTIDEATPIVNQIATELMTVPPA*

>gkv_731|gene_NONE|antifreeze protein, type I

MGILDFLSGQFIDVIHWTDDTRDTMVWRFEREGHEIKYGAKLTVREGQSAVFVHEGQLADVFGPGLYMLE

TNNMPIMTSLQHWDHGFKSPFKSEVYFVNTTRFNNLKWGTKNPIMCRDPEFGPVRLRAFGTFSMRVSDPA

AFMREIVGTDGEFTTEEISLQIRNVVVQAVSRILAASDVPVLDMAANTADLGKLITTAIAPIIAEYGIII

PEFYIENISLPAEVEKVLDKRTSMGIVGDLNRYAQFSAAEAMTAAAQNPGAAGAGMGMGIGLGLGGAAGP

WGAAPTPAAAAPPPPPPADGVWHIAKDGQTSGPFSRADLGRMAQTGTLTRDSHVWTPGQDGWKRADEVNA

LAQLFTVQPPPPPPGV*

>gkv_732|gene_NONE|NADH pyrophosphatase zinc ribbon domain protein

MTDLPNIPRRRRASAADTAEEHRFPCEQCGANLTFAPGQTELRCDHCGHVQHISLRGAMSDALRELDYNL

VLANLVRDAEYETTRFIQCPNCGAKSEFQEGTHAAACPFCATPVVTDTGAHRQIKPQALVPFTLDERAAR

DAMTKWLGSLWLAPNGLQEYARKGRSMSGIYVPYWTFDSNSRSEYTGQRGDIYYETRTVMRDGKRHTEQV

QRIRWTRVSGQTARVFDDVLVLAENALPRDYTDALEPWDLSQLLPYDPQFLSGFRAEGYALGPEEGFDIG

QQKMRAVIEGDVRRQIGGDRQQIDRLHVEHNDVTLKHVLLPIWVAAYKYRDKSYRFVVNAQTGKVKGERP

WSAWKIFFLVLAVGALAAGVAFLGYNG*

>gkv_733|gene_NONE|fructokinase

MDKTMILCCGEALIDMLPSSDMLGRKAYVPVTGGAALNTAIALGRLGTPVGLFTGLSSDPFGQMIISVAA

DEGVDTAPSAIRALPTTLAFVHLLDGRASYSFHDENSAGRMLAVSDIPDTSAACYLFGGISLAAEPCGAV

YEAFQARASESSLTMLDLNIRPALINDEAAYRARLDRMITRADIVKISDEDLDWLVGPGDMADQAQKLLD

MGPKLVLLTMDSRGARGFGRCGTRVSVAALHVPHVVDTVGAGDTFNAGTLAALYDAGLLTKDAIATLSAE

DLHAAMLQGTRAAGYCVAQAGANGPTRAQLCAL*

>gkv_735|gene_dtd|D-tyrosyl-tRNA(Tyr) deacylase|

MRALIQRVSEASVRVDGAVVGEIGSGLLILVCAMKDDDDTAPARMAVKLSKMRVFRDAQDKMNLSLMDTG

GSALIVSQFTLAATTKGNRPGFSDAAPPERGRQLYESFTSEVAALGIPHANGIFGADMKVALVNDGPVTF

WVEV*

>gkv_734|gene_NONE|tetrahydrofolate dehydrogenase/cyclohydrolase, catalytic domain protein

MAAVIIDGKAFAAKVRAQVGAAVTALKAQGITPGLAVVLVGEDPASQVYVRNKHRQTIEVGMNSFEHRLP

AETPEADLLALIAQLNADTAVDGILVQLPLPAHLNAELVINAIDPTKDVDGFHIDNVGKLVTGQKSMVPC

TPLGSLMLLRDSLGDLNGLEAVVIGRSNIVGKPMANLLLQQGCTVTIAHSRTRDLPAVVRRADIVVAAVG

RPEMVKGEWLKPGATVIDVGINRIQPAGADKAKLVGDCDFDSCAAVAGAITPVPGGVGPMTIACLLANTL

TATCRRYGLPEPVGLTA*

>gkv_736|gene_hflB|metalloprotease|

LGNARNIGFIVVIFLLLLGLFQVFSGNDNSNAANNPRYSDFVAAVDAGNVTSVTLNGEQVVYRTTDGRQI

STVRPADAQTTQMLLERNIPFEARSQEQSTLQAFLLSMLPFVLLIGVWIYFMNRMQGGGKGGAMGFGKSR

AKMLTEKQGRVTFEDVAGIDEAQEELEEIVEFLRNPQKFSRLGGKIPKGALLVGPPGTGKTLLARAIAGE

AGVPFFTISGSDFVEMFVGVGASRVRDMFEQAKKNAPCILFIDEIDAVGRARGVGYGGGNDEREQTLNQL

LVEMDGFEANEGIIIIAATNRRDVLDPALLRPGRFDRTIHVSNPDIRGREKILSVHARKVPLGPDVDLRI

IARGTPGFSGAELMNLVNEAALLAARANRRFVGMVDFENAKDKVMMGVERRSMAMTQEQKEKTAYHEAGH

AIVGMKLPKCDPVYKATIVPRGGALGMVVSLPEIDKLNYHKEEAEQKIAMTMAGKAAEIIKYGLENVSSG

PAGDIQQASSLARAMVMRWGMSDKVGNIDYAEAHAGYQGGGSAGGLSISAHTKGLIEEEVKRLIDEGYDH

AFKIIQDNYEEFDRLAQGLLEHETLTGEEIGRVMRGQPIGGGNDDDIEPPAPSVTSVPKTRPRREAPGME

PEPSA*

>gkv_737|gene_tilS|tRNA(Ile)-lysidine synthetase|

VPDLSKDPRAIARAFVTQVTGRIGVAVSGGSDSLAALLLLHEAAPGRIWAATVDHNLRADSAAEAEMVQR

FCAARDIAHYTLPWQPADMAGNLPHRARIARYSLLSTWAREAFLAGVVIAHNREDQAETYLMNLGRSAGI

DGLSAMRDRWRVEGVPFYRPFMALSRKALRDVLRAGAISWVDDPTNDDPHYQRVRLRRLLPQLAEAGIET

TDITRAALHLSQVSEALDWALTAAVGDPTTPAGEIVLPVAQYRSLPTAMRYRLLLRALDWIGGPAYPPRG

PELTGLDQALFGASRRTLGGCLFTSGARHLRITREVAAVGAAVPAGVLFDGRWQLAGQLPQGALIGRLGG

AIAEFPDWRASGYSRASLMAGPAIWQAQRLIAAPLLQPDAKWQAEVVRKFNMTPLSH*

>gkv_738|gene_ygbF|tol-pal system protein YbgF

MIRKAALMIGVALLPQFAVAQDAATLADIRQQLTTLYADVSNLRAELTASGGLSGGVAGATPLDRLNSIE

VVLQQITAKTEELEYRISRVVSDGTNRIGDLEFRLCELEPNCDIGSLGNTPTLGGGTGSSSAAAPAPSVT

TPPTSGGSSQMALGEQEDFARASEALAQGDFRSAADGFATYLQTYPGGALSAEAMLRRGEAQEGLGQISE

AARSFLESFAGAPDGAYAPQALLKLGLALGQMGQDTDACLTLGEVALRFPADPAVAEAATAMRNLNCA*

>gkv_739|gene_pal|peptidoglycan-associated lipoprotein

MHKFLIAGLLVSAVALSACSRPDRSATGDSNSPYGAGGYGQGVGAGGAGSASDPTSQAYFNQTIGDRILF

AVDQSSVSSEASQILNGQAQWLQTNSQYGIVIEGHADEQGTREYNVALGARRANAVREYLISRGISANRI

RTVSYGKERPIAVCSAESCYTQNRRAVTVISVGGIS*

>gkv_740|gene_tolB|tol-Pal system beta propeller repeat protein TolB

MNKFMALVLTAALAGPFAAMPALAQQGPLRITVTQGVIEPLPIAVPTFQAETAGAAEVANQISRLVAQDL

TGSGLFREVPASSFISNHAMFDQAVAYADWRAINVQGLVTGAVSVSGNQLTVKFRLYDIYTGAELGQGLQ

FNGSVQGWRRMAHKVSDAVYSRITGESGYFDSRVVFVAESGPKDNRLKRVAIMDYDGANVQYLTDANSLV

LAPRFSPTGAQILYTSYQSGAPRINLLDVASASARQLPTEVGEMAFSPRFSRDGGSVIYSLANGGNTDIY

RTDIRTGGHTRLTNAPSIETSPSLSPDGSQIVFESDRSGTTQLYVMGANGGEARRISFGDGRYGTPVWSP

RGDMIAFTKQQGGRFHIGVMRTDGSEERLLTSSFLDEGPTWAPNGRMLMFTRETQGETGGPSIYSVDISG

RNLRQVATGSFASDPSWGPLQP*

>gkv_741|gene_NONE|similar to telomeric repeat binding factor (NIMA-interacting) 1

VERLGLNTIGAKVSAIGHVALIVWMIFGLSLVSDPLEFEFPDVSVISGAQFDELAANTSPQVQTDVTATD

AASAEAPPEVAPMPPERPVQQQTPTPAETPVETPPAPEAAPTPVPVPDVAPVIEAPPAPVPDAAPSAPVV

APQVVEAQPAPQAPDAGAPDAAVAPRPVPRPADRIAPEAAPAPPLQAQEAPTAQAAVSPDADTPDVADEA

QEQTAPPEATTQTVTEADRPAAAPTNVPRPQSRPQRQAAAPTQSATPQTPPASEAAASTPAANNNDDILA

ALAAAQDTPSASTSAPQVQMSGAEQDAFRIAVQGCWAVDPGAAAAQVTVVVGFELDTSGRVIGNNVRMVS

SNGSGAAEQTAFEAARRAVLRCQGDGYPLSQAVLASGQSFEIEFDPSQMRLR*

>gkv_742|gene_tolR|protein TolR

MGAGVMKSGGGGGRRKRVRRSQAISEINITPFVDVMLVLLIVFMVAAPLMTVGVPVELPKTEASALPIDQ

EEPLSVTITADGLVMIQTTEVPPAELVTRLQAIAGERASDRIYLRADGANSWDMVAQVMGALNAGGFSNI

GLVTDTGGSTFAPQPQ*

>gkv_743|gene_tolQ|protein TolQ

MDITHDLTMWALFARATLIVKIVMILLVLASVWCWAIVVDKYIQYRRAKAEAEQFDRAFWSGEPLDLLYQ

QIGPNPDGASQRVFAAGMGEWRRSHRTDGALIPGALSRIERAMDVVIGKESERLQKGLPVLATVGSVAPF

VGLFGTVWGIMNAFVEIAAQQNTNLAVVAPGIAEALFATGLGLVAAIPAVIFYNKFNTDADRITASHEVF

ADEFSTILSRQLDA*

>gkv_744|gene_ybgC|tol-pal system-associated acyl-CoA thioesterase|

MTHQFALRVYYEDTDLAGIVYYANYLKYIERARSEWVRALGISQTALKTDSGVVFAVRRVEADYLTPARF

EDDLIVETILHAMTPARLVLDQRVLRGAEVCFAARVTLVAISASGAPVRLPAALRDALC*

>gkv_745|gene_NONE|hypothetical protein

MLDVTPYFTRPDGTYTFARWRRPLVPVIFGLADESLPIFKGAIEAVAAIARHGFAETDPEQGANLMIFVL

RDWAELEAAPEIAELIPEITAALPRLIEQDARSYRLFRFETDGAIRACFAFLRVTGADDEPAAEDLALDQ

AVRAMLTFGAGAAPQVLASSGGGTVVHPDIAAILRAAYDPVLPDAAGDASHALRLSARAAR*

>gkv_746|gene_ruvB|holliday junction DNA helicase RuvB|

MIESDPTLRADPLPEDNPDRALRPQGLDEFVGQAEARANLRIFIESARRRGEAMDHTLFHGPPGLGKTTL

AQIMARELGVGFRMTSGPVLAKPGDLAAILTNLEPRDVLFIDEIHRLSPVVEEVLYPAMEDFALDLVIGE

GPAARTVRIDLQPFTLVGATTRLGLLTTPLRDRFGIPTRLEFYTIEELNLIVRRGARLLGVPTDDDGARE

IARRARGTPRIAGRLLRRVVDFALVEGDGRISHALADMALTRLGVDDLGLDGADRRYLNMIGDTFAGGPV

GIETIAAALSESRDAIEEVIEPYLLQQGLIQRSPRGRVLTLAAWRHIGLEPPRPQSDLFG*

>gkv_747|gene_ruvA|holliday junction DNA helicase RuvA|

MIGWLSGRIIEKSGDQILLDVRGVGYIIHVSDRVMASLPGVGEAVALYTDLLVREDLMQLFGFTSRTEKE

WHKLLMGVQGVGAKAALAIMGALGGDGLSRALALGDWSSIAKAKGIGPKTAQRVVMELKGKAPTVMAMSG

GAESLDAVIEDDGPAPKPAARRARPVSNAQPDALSALGNLGYSPSDAAAAVAQAAGEAPDADASALILAA

LKLLAPKA*

>gkv_748|gene_ruvC|crossover junction endodeoxyribonuclease RuvC|

MRVLGIDPGLRNMGWGVIDVQGTRISHVANGICHSAGDDLAARLLSLHGQLTRVFETYAPDTAAVENTFV

NRDAVATLKLGQARAIALLVPAQAGLSVGEYAPNAVKKAIVGVGHADKGQVEHMVRLHLPGVKLVGVDAA

DALAIAICHAHHSQSAGHLQAAIARADQRINVRARG*

>gkv_749|gene_NONE|conserved hypothetical protein

MATYELNTTAYANTNGNFFARLLQRVATWNEMRQTRIALMSLSDHELADIGLTHADIDAIVSGKLAR*

>gkv_750|gene_hisG|ATP phosphoribosyltransferase|

MPLKLGVPSKGRLMEKTFDWFGARGLRLRRAGSDRDYAAVAEGLGGVEVVLLSAGEIPLELAAGRIHLGV

TGTDVLREKVANWPAQVEELAPLGFGHADLIIAVPNHWVDVETLDDLDAAAAAFRRDHGFRLRIATKYHR

LVREYMQAHDIADYQLVDSQGATEGTVKNETAEAIADITSTGETLRANGLKILGDGPILASQATLFRARG

AEWSDDVQDTYARLRQMLAL*

>gkv_751|gene_NONE|tRNA synthetase class II core domain (G, H, P, S and T) family protein

MIPTASKAAARAEAARILALFTAAGAEIVEADILQPADVLLDLYGEDIRARAYVTNDPLRGEMMLRPDFT

VPLVQMHMAAGAGPGRYAYAGEVFRRQEVSAGPAEFIQVGFELFTEQAEADADAEVYATITAALGDLNVQ

AAMGDIGILVSAVRALDTSDARKAALLRHVWRPRRFRLLLDRFGGRQPLPAPRADVLADAPPAFGLRSMD

EVAARLDAMREDAATPPIPPAQMALFDEILAVAAPAPQALRELRDIASRYAGIAPAVARLSLRMEALTAR

GIDLAEVAFETTFGRTALEYYDGFVFGLYAAGRHFPPVASGGRYDALTRVLGQGRAVPAVGGVIRPEIVV

GLKGA*

>gkv_752|gene_NONE|anticodon binding domain protein

MAKDKTRARPRAETPKGFRDYFGAEVTERAEMLHQIAGVYHAYGFDALETSAVETVEALGKFLPDVDRPN

EGVFGWQDEDADWLALRYDLTAPLARVYSQFRNDLPTPYRRYAMGPVWRNEKPGPGRFRQFYQCDADTVG

APSVAADAEICAMLSDTLEVVGIPRGDYIVKVNNRKVLNGVMEVAGVLDPSDPDKFVTERGIVLRAIDKF

DRLGDAGVRALLGAGRKDESGDFTKGAGLSAEQADIVMGFMFARRDTGVQTAARLRELVTGSAIGATGVD

ELETIAELLEAQGYGADRVMIDPAVVRGLGYYTGPVFEAELTFEILDEKGRPRQFGSVSGGGRYDDLVKR

FTGQEVPATGVSIGVDRLLAALRAKGRMGQQTAQGPVVVTVMDRDRMADYQSIVAELRRAGIRAEVYLGN

PKQFGNQLKYADKRNAPIAIIEGGDEKARGVVQIKDLVLGAQIAASASLEEWKDRPSQYEVARADMVARV

KAIIAGQA*

>gkv_753|gene_NONE|slyX protein

MSTDNMQTLLEEKIAHLTRMVEDLSDVIHRQDQELVLLTRRVQFLIERDANREAELMDSPAADQRPPHW*

>gkv_754|gene_dnaE|DNA polymerase III subunit alpha|

MKVGQALPVPQAAGYGWSMTAKGAAMADPKFIHLRVHTEYSLLEGAIHAGDLPKLAKAAGMPAVAVTDTN

NLFCALEFSEKASKSGIQPIIGCQVDLTYMKVEAGKRAEDPAPIVLLAQSEVGYANLMALNSCAYLDKGG

ALPQVTTADLAAHAEGLICLTGGPDGPLGRMIRAGQDNAARLLLERLAAIYPNRLYVELQRHPIEGGLPD

AEAQTERPFIEWAYALNLPLVATNDAYFKDEGFYEAHDALICIAEGAYVDQQEPRRKLTPQHYFKSQAEM

ATLFADLPEALENTVEIAKRCAFKAYKRDPILPRFADDEVEELRRQSKAGLQARLDVIPHAAELHVYWDR

LEFEMNIIEKMGFPGYFLIVADFIKWAKDEGIPVGPGRGSGAGSLVAYALTITDLDPIRYSLLFERFLNP

ERVSMPDFDIDFCMDRREEVIQYVQGKYGRDRVAQIITFGALLSKAAVRDVGRVLQMPYGQVDRLSKMIP

VEGVKPVSIAQALKDEPRLREEADREPVVARLLDYAQQVEGLLRNASTHAAGVVIGDRPLDHLVPLYQDP

RSDMPATQFNMKWVEQAGLVKFDFLGLKTLTVIQNAVSLIRKSGRHLHEAADGTVLYTPPKGAEDDIGAI

PLDDAKSYQLYSDAKTVAVFQVESTGMMDALKRMKPTCIEDIVALVALYRPGPMENIPTYCEVKNGQREI

TSIHPTIDFILAETQGIIVYQEQVMQIAQVMAGYSLGGADLLRRAMGKKIAEEMAKERPKFTTGAKETHN

IDEKKAGEVFDLLEKFANYGFNKSHAAAYAVVSYQTAWLKANHAVEFMAGVMNCDIHLTDKLAVYFQEVR

RGLKLGTVPPCVNRSLATFDVVDGKLVYALGALKNVGVDAMRLVVEARAGKPFVTLFDFARRVDLKRVGK

RPLEMMARAGAFDVLDSNRRRVLESLDALVAYSVAVHEQRSSNQVSLFGEGGDDLPEPRLPNVPDWLAAE

RLGEEFKAIGFYLSGHPLDDHMPGLRRKGVLTLSEVEGKAQNGAFLARMAGVVSGRQERKSARGNRFAFA

QLSDPSGQYEVTIFSDVLEAARPLLEAGTQVILQAEATLEADQLKLLARSFSAVGEMDTGVEGYRIYIDR

EEALAQVSSVLENAIAAKVRAPRGHIAFEIIVPDVGEVKIEAPRTYPLNPQIRAALRAVNGVQEVIEV*

>gkv_755|gene_NONE|conserved hypothetical protein

MTYYSGTLIAVPTASKEIYTDYARTSWPLFQRLGAVRMVETWGADIQAGDTTDFQRATLSTPDESIVFSW

IEWPDEATADKAWAEMETLDPSELPGEMPFDGARMMFGGFAPVVSSGTDKGADYIQGFVLAVPASNKIAY

IAEAERGWAEMFSPLGCLGLFENWGVDVPHGELTDFYRATKAEDGEVILFSWTAWADKATCDAAARQMEA

DYDPESHHAEMPFDGARMIWGGFDVIYDSDRHSLDRS*

>gkv_756|gene_NONE|glyoxalase/Bleomycin resistance protein/Dioxygenase superfamily protein

MGYQGTPCWYELGTEDIRAAGQFYRKIFGWQMIDGGMEEMDYHLGKSGEEMVAGFMSTADQEDPPPPNWL

IYFAADDCDKTAADIKAAGGQIYRGPDDIPGTGRYAIAADPQGAVFGILQADMSEMSVDDIAKVDAGDGA

FNQNKAGHGNWNELMTTDPIAAFDFYAGLFPWSKSQAMDMGDMGKYQLFSHKGADIGAFQGLGNAPVPAW

LPYFGVDGSVGAVVQTIQEAGGLVHHGPSEVPGPAYIAIAQDPQGAWFAIVGPDK*

>gkv_758|gene_NONE|domain of unknown function (306) family protein

MTQKIALAILLAASTALPAMAQETRTITGEIAYLQRIALPEVAQVRAEVRGPHDVLLAQYNQPTGGAQVP

LPFTLEIPDDVTARLTVSIAFEGQVRWLAPSVDLPLGGDDIALGTLQAAPFVAAGFTSSFNCEGEIISAG

FVNDTIVITREDGSQRIMPQVIAASGAKFADPDNPDQTFFWNKGDNATMRMDGILSECAVVADAQAAPWR

ASGFEPGWLIEITGDNYTLTRMDEDDVIGVLPEPTWQQGAVVWAISNPEMQLRAAPEVCYDNATGMPHPE

TVSLTLGDGTVLQGCGGNPASLLQGETWRVTDLNDLGVPSDGDGLIRFAADGSVSGQSFCNNFIGSYEIG

GEAISMGHLASTLKICGAQADYREEGFLKALRDTVMFSFDASGGLELRDLAGTVIIRASR*

>gkv_759|gene_NONE|calcineurin-like phosphoesterase family protein

MLARFLSGAAVLAISATAAQADFTLTILHTNDFHARYEPISSSDSTCSAAANTEGSCFGGTARLVNAVAD

ARARAENSILVDGGDQFQGTLFYTYYKGAVAAEFMNQLGYTAMTVGNHEFDDGPEVLRDFVAAVDFPILM

SNADISQEPELAGSIQKSTIVEVGGERIGLIGLTPHDTHELASPGPNVIFTDPAESVQEQVDQLTAEGIN

KIVLLSHSGLNVDMDVAARVTGIDVIVGGHDNSLLSNTIEGAKGPYPVMVGNTAIVQAYAYGKFLGELNV

TFNDAGEIVTAVGEPLLINNEVPEDETVVARIAELAIPLDEIRNEIVAQSGGEINGDRNVCRLQECSMGN

LVADAQLARVAGQGVTISLANSGGLRASIDAGEVTMGEVLTVLPFQNTLATFTTTGQGIVDALENGASQM

EDVAGRFLQVAGLKYTVDPAAPAGSRISDVLVQEADEWVPIDLAATYGAVTNNYVRNGGDGFAAFVTAEN

AYDFGPDLADVVVEYLVASGPYQPYVDGRITVK*

>gkv_760|gene_NONE|conserved hypothetical protein

MLIYKILRSDEWAALQAAGETLGAPVDLADGYIHFSTAETVAATAAKYFTDETGLWLLAVDVDACGAALK

WEPARDTLFPHLYRSLSLAEVVWAKPLPLGDDGHIFPEL*

>gkv_761|gene_pyrD|dihydroorotate oxidase|

MRALETLGLGLMRQCDPELAHRAALVALQLGLGPKGGPVTTPRLATTVAGLPLPNPLGLAAGMDKNATAL

APLSQSAFGFLEVGAITPRAQPGNDKPRLFRLTEDSAAINRFGFNNEGMEAAAQRLAHRPSGAVIGLNLG

ANKDSDDRASDFARVLTHCGAHLDFATVNVSSPNTEKLRDLQGRAALSALLHGVMAANAGLARPLPIFLK

IAPDLTEDELADIATVAGEVGLSAIIATNTTLSREGLKSRHAAEKGGLSGAPLFEKSTRVLARLSTMTDL

PLIGVGGVATGAQAYAKIRAGASAVQLYTALVYGGISMVADIARDLDALLARDGFANVADAVGTGRAEWL

*

>gkv_762|gene_NONE|thioesterase superfamily protein

MSHWLDEALVAVPYAQFLGVRLMWQDAAPIGLLPYREELIGVPTPPALHGGVSLAFLEVTALATLMQATA

GQMAETTGAQPLEWPRTIDLSVDYLRPGLQLDAYARARITRAGRRYATLSVAAWQEDESRPFAQAIGHFL

MPES*

>gkv_763|gene_NONE|uncharacterized domain 1 domain protein

MAAADATTDFIAMAQNFVTSLPQAAALQLQIEAIGHGMAQMRLPYAAHLARDAASGVLHNGPIATLIDTC

CGVASFSDPRIGGVTATLDLRLDYLRDATPGQDITVEARVLQVTRSVAFVEARAFDADKSVPVTTAKAVF

TAVMREGGK*

>gkv_764|gene_NONE|merR family regulatory family protein

MNAIIERLNLKEMCAAFAVTPRTLRYYEYIELLSPEKQGRNRFYGAREQARMKLIMRGRKFGFSLEDIRR

WLLLYDQEGIEAQLRKWYEAADSQLAALEERRKQLESTIRELRTLRNRSQTMLKP*

>gkv_765|gene_NONE|merR family regulatory family protein

MTETMTIREMCEAFDVTPRTLRFYEAKELLFPIRQGTKRLFTRSDRARLKLILKGKKFGFSLEEIRQLLE

LYALNDNQMTQLTRARELALVHLAEMEAQRATLDAAIAELRGEIAWGEGEIAARSTANAA*

>gkv_766|gene_NONE|hypothetical protein

MTALKQYSRLESFGLWRPALDAPARSVVVSFGNATLVLSDDSGRPLTHWSLPAIRALPRTDDATVSYSPD

LTGDEQLTLDDALMIEALAKVMAALDAPQKRRLPLRLFVLGSLVTLAVAVMVFLPILVRNQTLSVVPLAK

RAEIGATILGHMQAESGTSCRDPFGVRALDQLRRNALGPNWRGQIVVLPGPPFAPAVLPGRTIVLSQQAI

TRAADPAELAALVIATATAPTSADALRPLLEDAGFAATASLLTTGDLPQAPMVNYAHKLLEQSLPPVTAA

GSTPVAGDVLNDNDWVGLLGICNN*

>gkv_767|gene_NONE|sporulation related domain protein

MARRAILALLLTLPLSAAAEGAPPRDMITPEGCVLMRATLGADVIWAEVLGPDGQPWCPAPTIVAPVAPA

ATPRRPAVVPPQVQARAGDRFVQVGTFAAPENATRAAAQLRAQGWPVAFSDITVNGVPMRGVAAGPFAGQ

SQVQAALETARRIGFPGAIPQ*

>gkv_768|gene_upp|uracil phosphoribosyltransferase|

VVNHPLVQHKLTLMREKETSTASFRRLLREISLLLAYEITRELPMTLRRIETPIQEMDAPTIDGKKLALI

SILRAGNGLMDGILELIPAARVGFVGLYRDPETLQPVQYYYKVPQQLEDRVTIVVDPMLATGNSTAAAID

LLKASGAKDIRFLCLLASPEGIARLQEVHPDVSIVTAAIDEKLDDHGYIVPGLGDAGDRMFGTK*

>gkv_769|gene_add|adenosine deaminase|

VTASLEFRDLPKIELHLHLEGAAPPAFIRGLAREKSVDIGGIFDENGNYTYRDFDHFLKVYDAACTTLQG

PEDFHRLTLAVLEESASHGVIYTESFLSPDFCGGGDLAAWRDYLAAIEAAAAEAETRFGITMRGIITAIR

HLGPDQSRNTARVAAETAGDFITGFGLAGAELMGRPGDYTYAFDAAREAGLELTAHAGEWGGPDMVADTI

RDLRVSRIGHGINAIHDLALVDVLAETGTVLEVCPGSNVFLNAVKDWPSHPIARLRDRGVKVTVSTDDPP

YFLTTMSDEYANLARHFAFGPDDFAELNRTALDAAFCDAKTKERLAARLEKA*

>gkv_770|gene_deoB|phosphopentomutase|

MPRAFLLVMDSAGIGGAPDAGQFFNGAVPDTGANTIGHIAQTRGLQVPVMASLGLGAAIKAASGLLVDLP

PPTLGAYGAATEISPGKDTPSGHWEMAGVPVPWSWTYFPDIVPAFPGAVTARICELAGTAGILGNCHASG

TQVIDDLAEEHIRTGWPICYTSADSVLQIAAHEAHFGRARLIDICAALAPMLHAMRVGRVIARPFIGDAA

QGWQRTPYRRDFAIAPPAPTILDRAKDAGHPVHAIGKIGDIFSMRGIDDCAKGRDADLMQALQARVADAA

DGALVFANFVEFDTDYGHRRDVEGYARHLEWFDAALGRVIAALRPDDLLIVTADHGNDPTWVGSDHTRER

VPVLVHGAGVRDLGPLAFVDMAASIAAHLGLPPNGIGRSFL*

>gkv_771|gene_pdp|thymidine phosphorylase (TdRPase)|

LDARGLIVALRDGQGASPDAIRAFARGLADGTVSDAQAGAFAMAVLLRGLGDAGRVALTAGMRDSGDVLR

WDLDRPVLDKHSTGGLGDCVSLILAPALAAAGVAVPMISGRGLGHSGGTLDKLEAIPGLSVAGDEARLRQ

LIRDVGCAIVGASGRIAPADKRLYAIRDVSGTVESLDLITASILSKKLAAGLQGLVLDVKVGSGAFMRDL

AQAKALARALVETANGAGCPTSALITDMNEPLAPAMGNALEVRAALDVLQGTGGRLRDVTLALAQRLGVD

VTAALDTGRALEVFAQMIAAQGGPANFVPRVDHYLPKAAVIRDLPAPRAGYLGGWAGVDLGLAVVNMGGG

RRVEGDVIDPAVGLSDILPIGTPLAAGQAVLRIHARDEASCDITARAVLAAMQITDAPPAARPLILAEVL

*

>gkv_772|gene_NONE|cytidine deaminase (Cytidine aminohydrolase) (CDA)

MSLVDDARAIREKAYAPYSNFKVGAAIRATSGKVYVGVNVENVAYPEGTCAEAGAIAAMIAGGDTRIAEV

AVIADSPAPVPCCGGCRQKLAEFGDADVPVTLATVAGQSLATTIGQILPGVFNISHMQKA*

>gkv_773|gene_NONE|hypothetical protein

MRGVLGFVTTYIAPAVLLLAVGVGTSWLMTQSAGVAAPVLPDAGLAVVVDGCLGAPARAPLPASGDNGGE

FMALSAKDIARALDQCGGVSGALPWDLGAL*

>gkv_774|gene_NONE|ATP-NAD kinase family protein

MSQPLLHFAASRAPLAQEALAELTALYGQAPLDQAEAIVALGGDGFMLSTLHAPRPDGLPVYGMNRGTVG

FLMNDYRPDDLHARIRAAEVEVINPLHMRATDTSGTVREALAINEVSLLRTGAQAAKLRVFVDGRLRMPE

LVCDGALVSTPAGSTAYNYSAMGPILPIGSEVLALTAIAPYRPRRWRGAVLPRAAVIRIEVNDPAMRPVM

ANADSQPFADIAVVEVRSEPAVSHRILFDPGHGLEERLISEQFA*

>gkv_775|gene_NONE|hypothetical protein

VSVIGANPYVGGACDGRVLPQRQRRIEGKIGAGRGRMLAF*

>gkv_776|gene_NONE|serine hydroxymethyltransferase (Serine methylase)(SHMT)

MTDTTAFFTQDLAQRDSAVFDAITLELGRQRDEIELIASENIASLAVIQAQGTILTNKYAEGYPGKRYYG

GCQYVDIVETLAIDRAKQLFDVGYVNVQPNSGSQMNQAVFLALLQPGDTFMGLDLNSGGHLTHGSPVNMS

GKWFNVVSYGVRQQDQYLDMDDIRAKALEHKPKLIVAGGTAYSRVWDWAAFRAIADEVGAYLMVDMAHIA

GLVAGGQHPSPVPHAHVVTTTTHKSLRGPRGGMIMTNDEAIAKKINSAVFPGLQGGPLMHVIAAKAVAFG

EALEPSFKDYAAQVVKNAKAMADELQKGGIDIVSGGTDNHLMLADLRPKSVTGKAAEAALGRAHITTNKN

GVPFDPEKPFVTSGIRLGTPAGTTRGFKEDEFRQIARWIVAVVDGLAANGDEGNGEIESRVKAEVEALCQ

RFPIYPNL*

>gkv_777|gene_NONE|hypothetical protein

MNQDHLHNQTEEIRQLLREKHGLRGSSLQRQLERGGRLLPRAVRSEAWYLAQIDELSKNPKLLKMVDFEH

ADRAASVVRSHLESINPLDRLWGRMLSFFATMALAVLVLTGLFVLWHLTRG*

>gkv_778|gene_NONE|alpha/beta hydrolase fold family protein

MLNTITHGEPGTRRPILIAHGLFGSGRNWGVIARRLAAEGRQVIAVDMRNHGSSPWYPDHNYFAMAQDLA

QVIEDKLGSRADVIGHSMGGKAAMMLALTRPELVDRLVVADIAPVTYGHSHIASINAMQAVDLDAVSTRA

EAAAALGMDSDTTGLLLQSLDLPHKRWKLNLETLGREVRTISGFPQTGLTYGGHTLFLRGALSDYVLPEH

RDTIRAMFTRPHFAKLPGAGHWLQAEKPTEFFNAVSAFLCKDFEPAMQPAPAMA*

>gkv_779|gene_lepA|GTP-binding protein LepA

MTELDHIRNFSIVAHIDHGKSTLADRLIQETGTVAGRDMKEQLLDSMDIERERGITIKANTVRIDYKADN

GENYVLNLIDTPGHVDFAYEVSRSMRAVEGSLLVVDSTQGVEAQTLANVYQALDANHEIVPVFNKIDLPA

SEPARVAAQVEDVIGIDSSDAILVSAKTGVGIHEVLEAIVHRLPAPKGDPNAPLKAMLVDSWYDSYLGVV

VLIRVMDGTIKKGDRIRMMATNAVYGVDRLAVLKPQMTDIAQLGPGEIGVFTASIKQVRDTRVGDTITHE

KKGTTAALPGFKPAQPVVFCGLFPVDSAEFEDLRDAIEKLALNDASFSYEMETSAALGFGFRCGFLGLLH

LEVIRDRLEREYNIDLITTAPSVVYHLFMRDGTQMDLHNPADMPDLTLVDHIEEPRIKATIMVPDDYLGD

ILKLCQDRRGVQLDLSYAGNRAMVVYDLPLNEVVFDFYDRLKSVTKGYASFDYQLSGYQEDSLVKMSILV

NEEPVDALSMMVHRDRADMRGRAMVEKLKELIPRHMFKIPIQAAIGSKVIARETLSAMRKDVTAKCYGGD

ATRKRKLLDKQKAGKKKMRQFGKVDIPQEAFISALKMDS*

>gkv_780|gene_NONE|conserved hypothetical protein

LRHNGGKIGEWGQAMQKKTVTTWGLAAILALGLTACGPRGAISEACMTGGRSAANYQLCSCVQNVADQTL

SGSEQRRAADFFGEPDRAQAMRTSSSQRDRDFWTRYRAFTNTAASRCG*

>gkv_781|gene_NONE|glyoxalase/Bleomycin resistance protein/Dioxygenase superfamily protein

LDADTAPRFTDCSPILRMFDVDKAREFYLDFLGFGVTFEHRFHASAPLYMGIMRAGLTLHLSEHHGDASP

GTTIFVKMQNIRAFHTELTDKRYRYNRPGLEQAPWGLTVEVHDPFGNRIRFCEQP*

>gkv_782|gene_rpmB|ribosomal protein L28

MSRRCELTGKGPMVGNNVSHANNKTKRRFLPNLQDVTLQSDVLGRSFTLRVSAAGLRSVDHRGGLDAYLA

RAKDVELSDNALKLKKEIAKATAAVVAA*

>gkv_783|gene_NONE|hypothetical protein

MKTALKGKLQLLPETVFDGAHLHATHFAGAAGPNGQQLMVVFDYRKEGRAGFSAATHSSSYARQGYGQLS

IRTAQNDWYLNGDTAALEAALPAIAARYTRVHLLGYSMGGFGALRFARALGAKRAVVISPQFSLDPGVAP

FEGRYPPLAVTDDLGRHGTADLQGMILFDPFVPEDRAHAALIGRAFPGLSRLLLPFGGHPAIRTLRATRA

QWWVQRAAALGWDDAPSIRGAHRDARRLSQGYWLRLAAQAARLGHSALGDVARARAAALLPVAGDAEGGE

ST*

>gkv_784|gene_hrpB|ATP-dependent helicase HrpB|

LPIDPLLPDLIAALKAAGRAVLQAPPGAGKTTRVPLALLEANLSAGRIIMLEPRRLATRAAAERMAETLG

ESVGQTVGYRMRGESRTSAATRIEVVTEGILTRMIQTDPELAGIGAVIFDEFHERSLQADLGLALVQEVR

AALRPDLLLIVMSATLDAAPVAALLEDAPILTAEGRSFPVDHIYLDRPLAPKARLENALADLIARAAAET

TGDILVFLPGEAEIRRLESQLARLGDGFAIRPLFGNMPFAAQRAALTPDAARRRVVLATSIAETSLTIPG

ITVVVDGGRARRARFDPGSGMARLVTERVTRAEAVQRAGRAGRTAPGRAYRLWTRGEDGALLPFPPPEIE

SADLTGLALELALWGDDRLPFLTPPPEGTLAEARALLQGLGALDGRAMTAHGRQLAALPLHPRLGHMLLR

AGPASAPLAALMAERDPMRGAGSDLLPRLAAIQRGDPRADPAVIARIRTEAKRLAALAGPQADLSHAQMA

ALAYPDRIAQRRAGDQPRYLLSGGKGAVMADDDALASQPLLVITDTDGNPREARIRAALSITESEIRDLL

ADQITLHRITEWSPREGRVLARQREMLGAIALSDRAWPDAPPDAIARAMLAGVRDLGLNWSDAAQRLRAR

VQLLPDLPAMDDDTLLAEAEAWLLPHLSGLRTAADWRRFDMLNALRGRLDWGQMQRLDQAAPAQFETPLG

RKVPIDYAGDFPSIEIRLQEMFGTTRHPMIGVKPLRITLLSPGGKPVQVTTDLPGFWTNSYADVRKDMRG

RYPRHPWPEDPRAADPTLRAKPRGT*

>gkv_785|gene_NONE|NUDIX domain protein

MAVQMPPPKGPIAQRKEQLEQRLVTLRGVKTTTKGWGKLAAHADPLYHQIGALCWQPTPAGPQVLLVSAT

SGRWIIPKGWALPGKSPIDAALAEAWEEAGVKKAKAQPRSIGHYMGTKRTLGGDEVLGAVQVYALRVRKL

LDDYPEADRRQRQWFSAAEAAALVDEDGLRDLLIAFAAT*

>gkv_786|gene_NONE|exopolysaccharide synthesis, ExoD family protein

MTASASQTPPDPMNPPPRHGLTAVLRGIAMNGTDERISIRMLLEAMDGRAFGALMLLFALPNVIPTPPGT

SAILGVPLVYLTLQMMLGHNPWLPKVIADRSLARKDFVALVMRMNPWLEKAERLTSPRLQFLLNGKMERV

IGGICLVLAITLALPIPLGNMLPALAIAIIALGVLERDGLWILGGIITGIISMIVVSGVVYAMFRAAVFV

ITRTFGL*

>gkv_787|gene_argF|ornithine carbamoyltransferase|

MQHFLDINTTPADQLRGMIDKALAMKQARAGLPKGAGDADQPLADRMVALIFEKPSTRTRVSFDVGVRQM

GGQTMVLSGADMQLGHGETIADTARVLSRYVDMIMIRTFEEATLLEMAEYATVPVINGLTNRTHPCQIMA

DILTYEEHRGPITGKKVVWSGDGNNVFHSFVHAAQKFNFDLTFTGPQPLDPEAGIVAEARAAGANIVIER

DPMKAVEGADLVVTDTWVSMHDPASAKERRHNQLRGYQVNDTLMARAKSDALFMHCLPAHREDEVTSSVM

DGPHSVIFDEAENRLHAQKAVMRWCLGV*

>gkv_788|gene_argD|acetylornithine/succinyldiaminopimelate aminotransferase (ACOAT) (Succinyldiaminopimelate transferase)(DapATase)|

MISPILPTYNRAPLTFERGEGSWLITTDGDRYLDFAAGIAVNVLGHANPVLVEALTAQANKMWHVSNLYH

IAEQEALAERLVEHSFADTCFFTNSGTEAAELAVKMVRKFWTTEGQPERFRILTFEGSFHGRSSAAIAAA

GSEKMVKGFGPLLPGFTQLPWGDVAAIREHIFADDIAAVMFEPVLGEGGIRPVPDADLKAIRALCDEAGV

LLVLDEVQCGIGRTGKLFAYEWAGISPDIMMVGKGIGGGFPLGALLATENAAKGMTAGSHGSTYGGNPLA

CAVGNAVMDVVATPKFLGEVNRKAGLLRQGLEALVASHPDVFEEVRGAGLMMGLKCKPPAGELVNAGYAA

NVLTVGAADNVVRLLPALNIPDADIAEGLQRLDAAATALGGK*

>gkv_789|gene_NONE|putative membrane protein

VQPFEARNPVASARGASYGSAMTYAYVILFTLPRRRR*

>gkv_790|gene_NONE|gcrA cell cycle regulator family protein

MSWTDERVETLKKMWNEGLSASQIAKELGGVTRNAVIGKVHRLGLSNRTAGEETTAAPAAPDVTPEPPRA

SAPPPRPAAAAPQARPAAPARPVRPAPAAMDDDLSDIDAVDADDDQDDIAARDDDNDADDLDEIPGAAIR

RAIVPAGQPLPPQPSANEISAEALAKVSEVEKVARKLTLMELTEKTCKWPVGDPSTPHFWFCGLPVQSGK

PYCEAHVGVAFQPMNTRRDRRR*

>gkv_791|gene_NONE|NLPA lipofamily protein

MPIIRAKEKLLSVALIGAMGLAAMPAQALTIGVIPGMIADSIEAAAEVARAEGLAVEVVEFLDWTTPNVA

LSAGDLDANYFQHVPFLQDVNAATGFDLQVVDIGVLSRLGLYSNRFDSIDAIPEGAQVGLASDPTNQGRG

LRLLAEAGLITLSKDTYDVTPDDVVENPRALRFVEVDGPQLIRSMDDLDVVQAYPSLLVNAGLADKANQP

LILSAPDEDQFAIHFVARAENSADAELLRFIEIFQTAPEVRAAIDAAYVGYRDLYVLSWEEGAE*

>gkv_792|gene_NONE|csbD-like family protein

MNWDIVKGKWAQLTGDVKAKWGELTDDDLTQIDGEREKLAGKIQERYGLAKDEVEREIDDYFKNK*

>gkv_793|gene_NONE|hypothetical protein

MMGEMNADYLMFDHMEDRGQTAQTSHDALETVAPGAHGFLTATRNDRLVSLSRLHVQSPSFAGLNSFPDT

FEAVN*

>gkv_794|gene_aspA|aspartate ammonia-lyase|

MTDTILTADTRLEHDLLGDRAVPQSAYWGIHTLRAVENFPITGQRLSETPDLIVALAMIKQAAAEANATL

GLLSPEIADAIIAACLEIRDGKLHDQFVVDLIQGGAGTSTNMNANEVIANRALELLGHRRGDYARLHPNE

HVNLGQSTNDVYPTALKLAAWTGIGRLVQAMDGLRLAFADKSAEFSDVLKMGRTQLQDAVPMTLGQEFGT

YALMLAEDQSRLHEASDLIREINLGATAIGTGITAHPDYAALVRERLSLISGEDLITAPDLVEATQDCGA

FVQLSGVLKRVAVKLSKTCNDLRLLSSGPRAGLNEINLPARQAGSSIMPGKVNPVIPEVVNQIAFEVIGN

DMTITMAAEAGQLQLNAFEPIIFYSLHRSLSHLTAGCQTLEAHCIRGITANRDQLRMTVENSIGIVTALN

PYIGYRNATEVALEAHRSGRGVAEIVLERQLLTREQLDRLLRPENLTRPQPLLSKL*

>gkv_795|gene_NONE|acetyltransferase (GNAT) family protein

MTDVTTRSYQPADHDACLALFDGNVPHFFDPSERADYVAFLADQVLRRPYIVLEQAGRIIACGGLQVLAD

QRASFLSWGMVARDLQGQGIGRHLTMARIALARATEGVDKITLNTSQHTQGFYARFGFTPVKVTLDGYGP

GLDRWDMVLDLRLGAAKNTSGPS*

>gkv_796|gene_NONE|major Facilitator Superfamily protein

MQSMSHQIAALGTVPMALLHSATARVVAALSVTQFIGWGSMFWLPAVIGPAMAADLQMPLPMIMAGPTVM

LVLMAVTSWPLAPIFERRGARSVMIPGALLAVAGLVTLAFAQGPLSFLAAWILIGLAGAGMLTTPAQIAV

TEVAGDKARQALGILILTGGLTPTIVWPLTGLLQGIWGWRGAVLVFAALVLLVCVPLHIYALARKPRASK

TVQADTAPSRIDPLRLILLSSSFAANGFVTWGFALTIIILFEAGGLTPAEAMLAAAVIGIAQWGGRVLDF

FAGRYIPPLIMGLTGAALFPLSFFFLIANAGFSATMGFAVLYGVAGGITAVARATLPLDVFPAGAYARAS

AQMAVPLNLAFASAPPIVAAILTAAGPQAALWLALVISVFAFCALLGLWHLRRKDRALANIV*

>gkv_798|gene_exbB|tonB-system energizer ExbB

MLQSLAQTTRHFALLIAVMMVAPAAAQETAPARIEPAQIEPAQIEPALDATRAPEPAASIAHDLSPVGMF

MAADWVVKGVMIGLALATVTVLVLWLGKTLELWGARRRARNGLKLLAASTRLEDAIIAFQGERGAIALMV

RAAEDEARLSLGALDYAGSDGMKDRIASRLQRLSAGAGRRLQRGTGILATVGAVAPFVGLFGTVWGIMNS

FVSIAETQTTNLAVVAPGIAEALLATAIGLVAAIPAVVIYNGFARAVTGYRQILTDAGATIERLVSRDLD

WRKVPAAPEAE*

>gkv_799|gene_exbD|tonB system transport protein ExbD

MAGGIREHSGDDEDMDVSHEINVTPFIDVMLVLLIIFMVAAPLSTVDVNVDLPTANAPPSAREDQPIFVS

LRSDLTLAIGNDDLPRETLGTALDTATGSQRDRRIFLRADQSVPYGDLMAVMNLMRSAGYVQIALVAQEG

AMAQSAPPSP*

>gkv_800|gene_NONE|C-terminal TonB protein

MTQPPLNLSRRAVLDSAQGPLLLRPAANIISFARRSGELIGPQHLRTSLWAEGLRWGVSTLAICALIGGA

VFAATRAPRTPDGPAAPPPAIMMEFAPEIAAPEVEAPGTDLAPETPDQAPQQEIDAPVPDEPIPDEIREV

PPEILPEAPPEDLPLEPPPEVPPEPLDEEPPEELPPAAPPPTPIRSRLKNCPRSKPPRWPSARRRGRKTC

APRRPRRHLSSGRWPRPNPHRARSRRPAPTRQPRRRT*

>gkv_801|gene_NONE|tonB family C-terminal domain protein

VTSQQVPNISPQQWQDRVNTHVNRFITYPAEARAARQQGTPLIRITLDDAGNVISASLARSSGYTLLDQA

AVAMAYRASPLPVPPASVPQRSIVIPAEYILR*

>gkv_802|gene_NONE|tonB-dependent Receptor Plug domain protein

LLRQTALRLSMAMMVGAAFAPAAHAQQMVDYTVAPGPLQVALTDFSLQAGVQILFDSELTAGLSSLGLQG

PRAVDGALAQLLAGTGLTWRYSSAGAIVILRDTVVLDLGPDELMIGAVRAVSTVTGAAAPYATAGATSHI

AQENIQNFRGSAVSDIFRGTAGVMSGDARNGGAGMDVNVRGMQGFGRVATTIDGAENSISVYQGYQGSAN

RSYVDPDFIGGIDITKGSDVASRGIAGTVAMRTLRADDILAEGASVGHRLRIELGTNTASPEDGAVAGYA

WPGLYSTPVAVPSAQGLDRPAALRPTQGSLSFMTAVRDGDFDLLLGYAYRRQGNYFAGENGGQGAEPVDI

GRVESCNAYGYCQVWDPYIDNLGSTNYRLGEEVLNSELETHSILINGTRRFGADATLQLGYNGYFSEAGD

RLASRLNGDRVQDVQSTRTTGTDVHGVTARYRWNPADNDMIDLRANFWLTRLAQRQVSRSLVPSGVIGEL

QQRYRIGWDVTKYGGDISNESQFRFQNGQDLTLSYGLSYISEDVDPSLYGPILNSYARLEGEREEASAYI

RAAWKPLDWLTVNGGLRYTHADLHDGGDYETQTTAYFDRDPFVSGGGFSPSVGVTFDLPGETQLYANWSS

TLRYASLYESAMVSSFTLAPIGVEPERANNWEVGVNHIRDGLFTDSDSAMVKLSYFNWNVEDYIGRQYSY

HLQPNGSTYQGMQILNFDSATFEGLELSARYQNGGFTADFAASYFLDMGFCLTAGVCDSATLYSDFATNH

VPPEYTLDLTLSQQLMDERLTLGGRVSRVGPRAIDHGQVTAQGMSQFISLVNWEPYTLVDLFAEYRLNSD

VTLSFRVENATDQYYIDPLGLITTPGPGRSFYFGLTNSFGAGSDLPALLSGHVSDRSNGRDWAGWHAGLF

TGFNSTENDTSFTALDGSTNSITANEGFVADFNGALVGGQVGYNWQFANGLILGVQGEFSRPDQRADIEI

LSNEGTLAGTANLAAGYHQQIDWNAALNLRAGYAFSNDFNMYGLAGVARQEETWFRDSYTADYASRYMPN

GNGTTFAGLEEIDVSRNGINIGFGAEYALNDNWSIWGQFNRTFFRKTEVQFTEAREGTSLDYTMPVQVGT

ETVEIPAYPELCAILPDYCVPQYFENPIYEYVPVEGTYTNVNGRTGISDMTNDSFRIGFTYRF*

>gkv_803|gene_NONE|hypothetical protein

VALIASGHATADDARRLHDWRAQSAAHEAAFVAATRAWRTLGPALDQVAPVPAPQMSRRGVLTGGVIAAL

SLGGVGVATAALLHDGAAFVVATGGREMVTLADGSHVALDGGTRMTPQMAAGRRGLRLMAGASVVQVAAQ

EASPFALRTDGWQANIQPGSEVAVTLGPRSDCVECLSGALDLGGQALHAGQRLLRGSDGSVAVADVPAHL

MASWRDGLLVFQDRQLADVIADLNRHRAGRVVIMNPAMAGRLVSGVFHLASPDDVLAQIEMALDLEVMHL

GRMAVIT*

>gkv_804|gene_NONE|RNA polymerase sigma factor, sigma-70 family protein

MNSDETDLAQSFLRARPMLLRYARGRTGEASEGEDIVQDAWLRVVRHAPAVLAAPVPYMLRVVRNLAIDH

GRAQSRRLRHDEVDALLDVPSASPGPEQSAIAKSELQHFVRILRDLTPRRRDILIAARLRREPYASIAKR

HGVSTRTVEYEVKMALEECLSRMQDVGGGLGVLPGRRG*

>gkv_805|gene_NONE|proline dehydrogenase transcriptional activator

VTIETVDLDGYDRKILDALAVDGRIPLTALADRIGLSKTPTQARVKRLEESGVIRGYRALIDPVRLGLDH

VAFVEVKLTDTREAALARFNATVAKVPEIEECHLIAGSFDYLLKVRTHDMREYRRVLAEAISTLPHVAST

STYVAMQAVKDEGIAGAPA*

>gkv_806|gene_NONE|bifunctional protein putA

LVNPRYHIAIDRQIDFSGSDNKDRQLIPEIAMQSQFPALRAAIDAANLTDEAVAITALVAAADLTPEGRA

QISADAADLVRQIRAQKNPGLMETIMAEYGLGSDEGLALMSMAEALLRIPDAATADALIADKIAPANWGS

HAGKSPSLLVNSATLGLMLTGTVLNDTDKGAIRGAIRRLGAPVIRAATGVAMREMGKQFVLGETIDQGLK

NAEAFEKLGFTNSYDMLGEAARTAADADKYFREYEAGIAAIATRCTHGSIKTNPGISIKLSALHPRYEVA

QRDRVMAELAPRVLELCRQAKAANMGLNIDAEEADRLQLSLDIIEWVIAQPELAGWDGFGIVVQAYGKRC

GATIDFLYALAKAYDRKIMIRLVKGAYWDAEIKRTQVEGLADFPVFTRKHHTDISYIANTRKLFAMVDHV

YPQFAGHNAHTVSAVLFLARAAGLAVTDWEFQRLHGMGEQLHKLLRSTHGTSCRTYAPVGKHVDLLAYLV

RRLLENGANSSFVNQIMDEAISPEAVATDPFGLHQPGLALPAGPALFGAGRVNSTGFDLTDIPTLQAVEA

ARAPFATHHWQAAPRLATGAGTGVIAPVLSPATLEQVGTVTSADAATCSAAFDLAQNWDAPVATRAAVLR

RAADLYEENFGEFFALLAREAGKTLPDAVAELREAVDFLRYYATEAEAAGTRAARGTWVCISPWNFPLAI

FSGQVAAALAAGNAVIAKPAPQTPLIADKAVALLLQAGVPAHALQLMPGGAETGAALTASPRLAGVAFTG

STATAQAIRKAMAENAAPGTPLIAETGGINAMIVDSTALPEAAVRDIINGAFRSAGQRCSALRVLYVQED

IADRVTDMIRGAMDLLSLDDPWDLDSDVGPLIDGMSRDKIAAHVDTARATGTLLHQGAAPAGGHFLAPAM

IKVPGITAIEREVFGPVLHIATFAAKDLDQVVDDINASGYGLTFGLHTRLQSRVADLSHRIHAGNIYVNR

NQIGAVVGSQPFGGEGLSGTGPKAGGPNYLPRFAAPETRATRAGFMPGPTGETNELILSPRGKILCAGPT

PEDVAAQTAAVRALGGVAMTGAADYTSADIAAVLWWGDETRAREIERALAQRKGAIVPLITSQPDIAHIM

HERHICIDTTAAGGNAALLAEVGKAA*

>gkv_807|gene_NONE|inositol-1-monophosphatase (IMPase) (Inositol-1-phosphatase) (I-1-Pase)

MAIGSANLNVMIKVARSAGRALIKDFGEVENLQASMKGPGDFVSRADRRAEETIRNGLMEARPSYGFLGE

ETGMIEGEDPTRRWVVDPLDGTTNFLHGLPHWAVSIALEHKGQIVAGVVYDPVKDECFYAEKGAGAWMNE

QRLRVSSRTKLLESVFATGIPFATQRTLPATLQDLARIMPTCAGVRRMGAAALDLAYVAAGRFEGYWERE

VKIWDIAAGMIIVQEAGGFVGPIRDDHDPLVHGDIVAANGEIFDKFAKLVRARPEAAIPTE*

>gkv_808|gene_NONE|HTH-type transcriptional regulator metR

MHIEFRHLRTIKAIHDAGGLAKAADLLNMTQSALSHQVKNIEDQAGVELFVRRSKPLKLSAAGKRLLVAA

ESILPQIAALEAEFKSLAGGSAGRLHIAIECHACFEWLFPVMEQFRKLWPDVDVDIRPGLAFGALPALRR

EEVDVVISSDPEASDDVTFLPLFDYAPTFVASCQSPLAAKPYVTAEDFRDQVLITYPVERARLDVFSQLL

TPAKVEPAMIRQVELTAMILLLVASNRGVAVLPDWVVHQQRYKADYVMRPLTEGGISRSLYAAVREGDAE

RPFVQDLVRLAQAQAVMLQNGQANGF*

>gkv_809|gene_metF|5,10-methylenetetrahydrofolate reductase|

MSAPIISFEFFPPRNLEAAFRLWDTVKALAPLRPAFVSVTYGAGGTTRQLTQEAVVAIHKATGLNVAAHL

TCVNATRDETLAIADDYAAAGVTQIVALRGDPPKGAGKFTPQPDGFQNSVELIAALAATGKFKLRVGAYP

ERHPDAANTAQDVEWLKRKFDAGADSALTQFFFDTEAYFRFLDLCEKAGITGKIIPGILPVENWTGARRF

AESCGAHIPPAVIAAFEAAPNDATAVAIDLATQMSQQLMQAGADHLHYYTLNKPDLSLAIAANLGIRP*

>gkv_810|gene_valS|valyl-tRNA synthetase|

MPMEKTFDAASAEARLYQTWESAGAFRAGANASRPETFSIVIPPPNVTGSLHMGHAFNNTLQDILTRWHR

MRGFDTLWQPGQDHAGIATQMVVERELAKAGLPGRREMGREAFLDKVWEWKEQSGGTIIQQLKRLGASCD

WDRNAFTMDEHFQKAVIKVFVEMYAKGLIYRGKRLVNWDPHFETAISDLEVENVEVAGHMWHFKYKLAGG

ESYEYVEKDEDGTVTFREMRDWISIATTRPETMLGDGAVAVHPSDARYAAIIGKMIELPLTGRLIPIIAD

EYPDPTFGSGAVKITGAHDFNDYQVAKRHNLPLYRLMDTKAALRADGLPYAEAAARAAEIAAGAATNEEE

IDSLNLVPEKYRGMDRFEARKAIIADIGALGLAVTRLVKTIDKETGAEDLSREAVVDSKPIMQPFGDRSK

VVIEPMLTDQWFVDTSKIVGPAIDAVRNGDTQILPERDAKVYFHWLENIEPWCISRQLWWGHQIPVWYGL

DLGASNYRDDENDGALDEVEIMRLLSSDRLVHAGAVYHCAADFSGVVENFRDEIADTPMPLSHARIVEVA

DRAAAIEALAAGLAQYNLTQDPTVLVYPVWRDADVLDTWFSSGLWPIGTLGWPEQTPELAKYFPTNVLVT

GFDIIFFWVARMMMMQLAVVDDVPFKTVYVHALVRDEKGKKMSKSLGNVLDPLVLIDEYGADAVRFTLTA

MAAMGRDLKLSTQRIAGYRNFGTKLWNAHRFAEMNDVFAARPADGQAPRASQTVNRWIIGETARVREEVD

AALDSYRFDVAANALYAFVWGKVCDWYVEFSKPLLSGEDIAARDETRATMAWVLDQCLVLLHPIMPFVTE

ELWQTTGQRDRLLALSDWPTYAAADLVDAGADAEMSWVISVIEGVRSVRAQMNVPAGLQVPVLQLEADEV

ARGAWGRNEVLIKRLARIDSLTEAAEVPKGAITVPTAGATFALPLADIIDVAAEKARIGKALDKLAKELG

GLRGRLNNPNFVASAPEEVVEEVRENLSLREAEEAQMRAAYDRLAEIG*

>gkv_812|gene_NONE|aminotransferase class I and II family protein

MSDARLTSLAASLPATVPFVGPEAQERAAGRPFAARIGANESVFGPSPRAIAAMAAASEHVWQYADPENH

DLRAAIAAYHGVPPENIAIDAGIDTLLGLLVRLTVAAGDKVVTSAGAYPTFNFHVAGFGGELVTVPYNDD

HEDPAALIAKAAEVQAKLIYIANPDNPMGTWHSADVIQRMIDAVPAGSLLVLDEAYIELAPEGTAPDLHP

EEERVIRMRTFSKAYGMAGARVAYAIGPAGLIAAFDRVRNHFGMNRTAQIGALAAIQDQAWIDHVRARVT

VARNRIADIAADNGLSVLPSATNFVALDCGQDGAFARRVLDSLIKDGIFVRMPGVSPLNRCIRVSAGTTA

DLDAFAAALPKAIAAANQGA*

>gkv_811|gene_NONE|aminotransferase class-V family protein

MFANGRTHLAIPGPSVTPDEVLRAMHRTSTDIYGGALLDMTVSIYPDLQKLARTASALPVIYLGNGHAAW

EGAIANTLARGDRVLVLATGNFGIGWANQARAMGAEVVVVDFGLTTGIDLSRTEEALRADLASGTPYKAV

LMSHVDTATSLCNPVLPVRQVMDAVGHPGLLMVDCIASLGCDRFEMDAWGVDVMVATSQKGLMVPPGISF

VYFGAKALAAREGVGFVPAYWDLKPRANPPAFYGLFGGTPPVQHLYGLRAALDMIMAEGLENVWARHAGL

AQAVWAAFDAWSAGGPVSLCLSDPSLRSHAVTAAYAGPGNGDALRGWVTKFTGVTLGVSLGRDPAADYFR

VGHMGHVNAHDVFGVLGAMDAGLKALDIPHGEGALAAATKVIAAAAKKAA*

>gkv_813|gene_NONE|hypothetical protein

VIICNAVAMFVKFYVAVMNRASHLVENLAVACPWVIDVLKNLSHG*

>gkv_814|gene_NONE|protein tyrosine/serine phosphatase

MIDTGRVTCPSGWRDTLDFLFLDHAVLRYTWCNQAKVCDDVWRSNHAGFGRLRRLRDQGIKAILTLRGSS

PSAANRFEAAACHTLGLHLYSVSLEARRAPKRDEVLRLFNTFRALPRPFLMHCKSGADRAGLAGALYLLG

MQGATIEQARKQLSLRYLHIRASQTGVLDHVLDLYENDFRRYPIGIEEWFATKYDRDAATESFRR*

>gkv_815|gene_NONE|ABC transporter transmembrane region family protein

MTIPAPDSAQQTPDMQPHLFRWYWQEFLGRYWPLLLIAIILMAIEGATLASFAVMMQPMFDSIFEGGSRA

ALWGIGLAMLAIFTLRAFASLGQKILVTRVNELVGARLRARLLSHLMTLDGSFHQQHPPGQLIERVQGDV

NGITSITAMILISLGRDLAAVLALFAVALVTDWQWTLIALVGIPLLVAPSLVVQRLVRRLTRKSREVEGT

LSTRLDEVFHGINPVKLNNLEDYQSRRYRDLLGQKVRLATSSAAGKAAIPSLVDIMTGLGFMGVMVLGGG

EILSGEKTMGQFMTFFTAMASVFGPLRRLAGLSGSWQAGQVALERLKSVLDIAASITPTDRPQPIPQAPP

EIRLQDVRLDYGTTEVLHGVTFTAAAGKTTALVGASGAGKSSVFNLLPRLIDPSSGAVLINGISNKDFAL

HDLRDLFSVVSQDSALFDESLRDNILLDRPAPEDSMLEDVLAAAHVTDFLKALPEGLDSPVGPRGSALSG

GQRQRVAIARALLRDTPVLLLDEATSALDTRSEVLVQQALEQLSHGRTTLVIAHRLSTIRNADSIVVMDA

GRVVDQGTHDELLARGGIYADLYAMQFRSSAESETDE*

>gkv_817|gene_NONE|aminomethyltransferase folate-binding domain protein

MTHARKVFAITGTDRLPFLQNLVTNDVKRAEGALVYTALLTPQGKFIADFFLHEDGSRLLLDVDAGAAAA

LIPRLSMYRLRADVQIAETDLVVSRGTGDAPAGALADPRDPRLGWRLYGAADVSDATDWDALRVDLLVPE

MGAELTGESYILENGFERLHGVDFRKGCYVGQEVTARMKHKTELRKGLARVQVVGDAAPGTVIMAGDREA

GQLLTRAGDQAIAYLRFDRAGGEMTAGSARVTRLPD*

>gkv_816|gene_NONE|inositol monophosphatase family protein

MHVTPQQERDLIAIMQDAARAEILPRFRNLHADEISTKSHPGDLVTAADILSERRMTAAIPAVMPGAVVL

GEEAISVDPILRDQIGLAATSVILDPVDGTWNFTKGVALFGMILAVAHREVPDFGVLFDPVAQDWVVAHA

GQPTRFVTADGFSQSVHTSDERDPAKMTGFVPFEIMHRHHRQTVTAAMDGFASATALRCSCHEYRMIARG

QAEFLISHHKPNPWDHAAGVVAVKGAGGVARFIDGEDYNVARPHGYLVSAASEEIWQMVADRFRFLQADA

V*

>gkv_818|gene_efp|translation elongation factor P

MAKINANEIKPGFILDHDGGLWAAVKVDHVKPGKGGAFAQVELKNLRDGRKLNERFRSEDKVDSVRLERK

DQQFLYETDGILTFMDTETFEQTELSSDLLGDRRPFLQDGMTIMIEYFGDEALNVALPPKVTCRIAETEP

VMKGQTAAKSFKPAILDNGVRVMVPPFVGPDELIVVNTETMEYSERA*

>gkv_819|gene_NONE|hypothetical protein

MKEFVDSTAYNNEQGNRARKLFAAVVLAALDDAIADDKKYGNGPEQIARWARSRDGREVLSCAGIDPNER

VVSGLMDFVAKGVRTSVALSREESERRHAAEEEARAA*

>gkv_820|gene_NONE|hypothetical protein

MEAVMRRLLIIWVPLLLLGLFCGYWLVAQAQIKRAVTVAMERLPAYGWQANLDTLHLRGFPFRFDVMATD

VSAENLAGTLAWQAPALNIHALSYQPNRIVAALPPQQQVTLYGQRIDVQSADMRVSTHVGLSPDLPLDEA

VLEARDLALTSEFGWQAQVSHLLSALRAAPDVPADSPPTYDAYTRAQNLVLPEAIRAALDPSGVLPAAVA

SVEFDTRLSFDAPIDRYLTPQVAPTSVNLRRIHLQWGQMQLTATGTLQPGSNGLAEGEIALSLNGWQQLV

TLAENAGALVPDRAQQLRFLLGAAAGGGDRLSLTLTVSQGVLSYGPVPLMPLPRMLQPQTP*

>gkv_821|gene_NONE|motA/TolQ/ExbB proton channel family

MELKSTATKDQFSQPLRQLLSMLVVLALVVVGGWIVHTTIEGIFWTNPYLNGLIAGVFVLGVVACFMQVF

ALARAISWIETFVRLPQGVPERTPPMLAPLAALLKSSRNQRLHLSPSSSRTILETVGQRIDEAREFSRYL

ANTLIFLGLLGTFYGLATAVPALVDTIRSLNPQGEETGAAIFARLQAGLEAQLGGMGTAFSSSLLGLAGS

LIIGLLELFAGRGQNRFYNELEDWLSSFTRVTYSGAGGGEGDGGTETSVLALVMGQLSDQLAKLQGQMTD

AQDISAQTATQMQTVASGLTAMMGRMESDNAALARIATGQEQLVELLARREGVADGFDPESRMRLRSIDG

QMNRVIEELTASRQESMAELRADIAHLTRTLRDVAS*

>gkv_822|gene_NONE|ompA family protein

MRRSTERFNTNVWPGFVDAMTGLLLVLLFVLSIFTVVQFVLREEVTGQEQQLGQLSTDLAALNAALGSER

SRAAALEGQNAERQSLIDTLSQSLAERDSALATAGDQITDYEAQVAALIAGRTQMQGEIDTAAEEARLAE

TRREALQALIASLRADADSAAGQISDLEAQALLDAAAAEALRARLENADAELTAMTLSLESERARAEETL

TLLAAARAAQAELEGARDTALTDAEAQAALLALAQQQLSEQQEMSSQAQLQVEALNAQAAALRNQLGDLQ

GLLDASAVADSVAQVQIESLGSQLNAALARVAAEERRARTLEEAERIRLEEEARRLAEEAESLEQYRSEF

FGSLRALLDGQDGVQIVGDRFVFSSEVLFEAGSATLSAAGDAEIANVAALLRRIASEIPSQIDWVIRVDG

HTDNTPLSGTGEFANNWELSQARALSVVLYMIQNEGIPPNRLAANGFGEYQPLNPANTPEARAQNRRIEL

KLTER*

>gkv_823|gene_NONE|glutathione S-transferase, C-terminal domain protein

MPELYGVYKSRATRNVWLALEAGLKLPLVRTIPSSRIADPLAADAPFNTKSPDFLALNPMATVPVLKDGD

LVLTQSLAINLYLARVYGDKLGPQNAMETGAMSNWALFAATELEPHTLRIQVLVGKGATDEALAEAAEAY

ARPLKALEVHFAKHAYLVGERFTVADINLCEVLRYATALKGAFDAYPAVKAYLATQQARPAFQEMWALRE

AEA*

>gkv_824|gene_parE|DNA topoisomerase IV, B subunit|

MADDLLAGSGAQGNEYNAASIEVLEGLEPVRKRPGMYIGGTDERALHHMVAEILDNSMDEAVAGHANRIE

VELHEDYSVTVRDNGRGIPVDPHPKFPDKSALEVILCTLHAGGKFSNKAYSTSGGLNGVGSSVVNALSDL

MRVEVARNRELYVQSFSRGVPQGPVQKVGAAPNRKGTSVTFHPDAEIFGSLKLKPARLFKMVRSKAYLFS

GVEIRWKTAIDDGETPAEATFKFPGGLADYLADVMKDTQTYADKPFAGKVSFEEKFNVPGSVEWAINWSA

MRDGFILSYTNTVPTPEGGTHEAGFWAAVLKGVKTYGERVNNRKAKDITREDLLTGGCALLSIFIREPSF

VGQTKDRLSTEEAAKWVELAVRDHFDNWLAADPKSAREILDFLVLRAEERLRRRQEKETQRKSATKRLRL

PGKLTDCSAKIRDNTELFLVEGDSAGGSAKGARSRETQAILPLRGKVLNVMGAAAGKLNQNQELSDICEA

LGVQMGAKFNTEDLRYQRIIIMTDADVDGAHIASLLMTFFFTQMRPLIEKGHLFLACPPLYRLTQGANRL

YVADDAEKEYWLQKGLGGRGKIDVQRFKGLGEMDAKDLKETTMDPKTRKLIRVTINDDEPGETSDLVERL

MGKKPELRFQYIQENARFVEELDV*

>gkv_825|gene_NONE|glyoxalase I/dioxygenase superfamily protein

MSSLALDHIAVCARTLDEGCDWVEARLGIRPRPGGKHARYGTHNCLLGMGDGFYFEVIAPDPEADHSGPR

WFGLDHPPEVSRIGNWICRAEGLAEFQTPVDAGVVTPLERGALRWNITVPEDGSLPLDGAFPTLIEWTAG

QHPSTVLADSGLRLQNLHISHPQVALVRDWLIGHLGDPRVTLTEGPLALTASFIGPHGTVNLA*

>gkv_826|gene_NONE|dnaJ domain protein

MSIWSRISEALAALLHGEGLFAALERLRNPPEHSVAFTIAVIALGAKIAKADGQVTRDEVSAFRDVFEIS

DADCGHVARVYDLARQDVAGFDAYARHVADMFRDHPDTLKDLLEGLFHIAIADGDYHPAENMFLKEVARI

FGFTEAEFACIRARALPADMPGAENDPYHVLGVAPDAPIDDIKAAWRHLVRENHPDRLTARGVPAEAIKL

AEKRLIAVNNAWSAISARGALQPA*

>gkv_827|gene_nadE|glutamine-dependent NAD(+) synthetase (NAD(+) synthase[glutamine-hydrolyzing])|

MGETFRLTLAQLNPVVGDIEANAARARRVFHDARAAGSDFIVLPEMFLIGYQAQDLVLRPAFQRDAAAAL

AQLARDTDGGPALGIGLPWREGDELFNAYAILSGGKVQAMVRKHELPNYGVFDERRYFAAGPISGPVAFE

GGPRIGLPICEDAWFPDVAEAMAESGAEILVVPNGSPYSRGKYDVRVQRMVSRVTENDLPLVYLNMVGGQ

DDQVFDGGSFVLGHGGGLAAQLPFFTETVSHIDFTRCETGGWQPQRSTLAHVPDEHALDYQAMTTALRDY

VRKSGFSRVLLGLSGGIDSALVAAIAVDALGAENVRGVMLPSRYTSDHSLEDAEVCARALGIKLDTVEIA

GPVAAAEAALAPLFAGHDADLTEENIQSRMRGLLLMAQSNKFGEMLLTTGNKSEMCVGYATIYGDMNGGY

NPIKDLYKTRVFAVAAWRNANHFDWMAGPAGEVIPTRIITKPPSAELRPDQKDEDSLPPYPVLDAILDLM

VDRDASVADCVAAGYNRETVKRVEHLVAISEYKRFQSAPGVKLSRRALWLDRRYPIVNRWRDKA*

>gkv_828|gene_NONE|MORN repeat family protein

LQHLVQGFTRSRAEGKRDQFSPIPARALLEKLSGQREADTVAAMPLMTEIQMPRPALRILTLIGGLVGGA

VAMQAVAQGVEVKQYANGGIYEGQFLNGRQHGQGSYRLPNGYEYTGQWFEGEIRGIGRATFPGGDIYEGS

FSAGKPEGTGTITYADGSSYTGEWVDGRLHGTGTLTYADGSRYEGSFQNNMPSGEGTLTMPDGFSYAGDW

VNGVRDGAGRITYADGATYEGGVDAGLPDGEGTLTQADGTTYTGDWRAGAMTGQGQMQLANGDSYTGGFA

SGFFDGEGALTYANGDSYTGGFRAGQRSGQGLFTGANGYRAEGVWADGALSGVATVTYADGAVLVAGFEN

GQASGSGKITYADGASYDGEWANGTMSGQGTVTFANGDSYVGAFADGRMHGTGRMTAADGASYDGAWANG

LRDGEGTARYADGSVYMGGFAGGVRNGQGTLTMADGFNYTGYWSEGEMDGQGTATYPGGEVYEGNFRAGR

RDGHGRLTYPDGTVETGEWQDGAMVEPLVVPPAADAPAVSE*

>gkv_829|gene_leuA|2-isopropylmalate synthase|

MPQNRIQNNFASDKNRVLIFDTTLRDGEQSPGATMTHAEKLEIAHLLDEMGVDIIEAGFPIASEGDFQAV

SEIAKITKNATICGLSRANIRDIDRAWEAVKHAASPRIHTFIGTSPQHRAIPNLTMDQMAERIHETVTHA

RNLCDNVQWSPMDATRTEHDYLCRVVEIAIKAGATTINIPDTVGYTAPRESADLIRMLLERVPGADEIIF

ATHCHNDLGMATANSLAAVEAGARQIECTINGLGERAGNTALEEVVMAMRVRHDIMPFDTKIDTTKIMHI

SRRVATVSGFPVQFNKAIVGKNAFAHESGIHQDGMLKSADTFEIMRPADIGLAGTSLPLGKHSGRAALRA

KMRELGIEMADNQLNDLFVRFKALADRKKEVYDDDLIALVQDADTNEVHDTLQVKRLRVICGTEGPAEAL

LTLTVDGVDKAADATGDGPVDAAFKAIKTIFPHDAVLHVYQVHAVTEGTDAQATVSVRIEEDGRIATGMS

ADTDTITASVKAYVNGLNRLIERRKKTAPGVDHRDVSYKMSAEG*

>gkv_830|gene_NONE|hypothetical protein

MMTRRTAAALGLALALSMGSVFAAKAQDESPTAAAIALWQEDRTQIFDAAGLEMAELAYIARLLVIFADS

PAQPQFQRQLELLAEDPEALALRDVMVITDTTPAEANAIRRQLRPRGFSLVLVEKDGRVELRKPDSWSLR

EIIRSIDRMPLRVQEINNALGRG*

>gkv_831|gene_NONE|putative lipoprotein

MKNIVKFVGAAAIAVTLAACGDTDIERGATGALIGGAAAAVTGESVMNGVLIGGAVGAVSCSVAPGAPNC

YR*

>gkv_832|gene_NONE|hypothetical protein

MIKHRGFPGRLQGTDFQFVIRRAHKDGATKLTARERFSDRRPADREADRGFLLALWENFGEEPFERGNLD

AGRLSWVLGREVVPAEPEAFDPESYEALLRVDANKLRANFPGLID*

>gkv_833|gene_NONE|hypothetical protein

MRRIALALAIGMASGASAQQVIAPSGVEVQLYDVRFDEDAARFRFVAPVLAGVAEVPDSWTADATWLCTY

LALPALSANDVTPAQVVVSVSGAEVPFGEATPDVPQFFEGFAVADGACQWGLF*

>gkv_834|gene_NONE|conserved hypothetical protein

MPQLPIPVQTIGLLLLSNIFMTFAWYGHLKFKSAPLLAVIAISWGLALFEYMLQVPANRIGHGYFNAAQL

KTIQEVLTLGIFILFSIFYLGEPLRWNQLVGFGLILVGAWFVFQTFPGQQG*

>gkv_835|gene_NONE|conserved hypothetical protein

MAGHSKWANIQHRKGKQDGIRSKLFSKLAKEITVAAKMGDPDPDKNPRLRLAVKEAKSNSVPKDVIDRAI

KKSQGGDAETYDAIRYEGYGPNGVAVIVEALTDNRNRTASNVRSFFTKTGGNLGESNSVAFMFDRKGEIA

YKAEAGDADTVMMAALEAGADDVESDDEGHWIYCGDTALNEVSTALEAALGESETAKFVWQPQTTAPVTD

LETLQKVMRLVELLEDDDDVQNVTTNMEVSDELMAEYAES*

>gkv_836|gene_NONE|calcineurin-like phosphoesterase family protein

MKILFLGDVVGRAGRRAVTENLPRLREAWRLDFVVVNGENASGGMGLTGAHCKTFLEAGADVVTLGDHAF

DQKDMLQYIETEPRVIRPLNFSKVAPGKGARVFEARGGRKVLVAQVLGQVFMKRPFDDPFSALETVLKTH

PLGGLVQASLIDVHCEATSEKMGLGHYCDGRASVVVGTHTHVTTGDAQILPGGTAYMTDAGMCGDYNSVI

GMDKAEPLRRFITGMPRDRFTPAEEEATLSGLYVETDDRTGRATRVEMVRQGGRLQQSGPL*

>gkv_837|gene_NONE|5-formyltetrahydrofolate cyclo-ligase

MTIDDEKIACRARAAARRALAHAAADDRASARLAQVLGEYAGQVISGYVAIRTEIDPMPALRIAAGQGSR

ICLPVIDAPATPLRFRAYTPGDALADGALNTLEPAHGDFLRPDVVVLPMLAFARDGRRLGYGGGYYDRTL

QALRETGPVVAIGFAYQAQMDDDLPTDPYDQPLDLMVTDQQIIDFRR*

>gkv_838|gene_mgtE|magnesium transporter

MDQHDEKIIDAEESYGLSTRLFDEVLDAIEADDAPAIDAALEPLHAADIADLIEQLNTRDRRALLQLWPN

GVDGEILSELDENLRDEVIAQLNPDQLAEAVRDLESDDVVDLVEYLDAPQQEAVLDALDASDRVAVEKAL

AYPEESAGRLMQVEVAKAPEHWTVGEAIDFLRSDIALPDQFYHLVLVDPRMRPIGQATLGRILSSARATP

LKDILEETFITFNVNDDEGDVARAFNQYHLISSPVVDSDDRLVGVITIDDAMIVLDEEHTEDMLRLAGVG

ENATISDSVFETVKQRFPWLFVNLITVNIAALIVGLFDQTIAAYVALAALMPVVASMGGSAGTQSLTVAV

RAIATNDLTASNAWRVIRREMFVGLLNGLVFAVVMGAVGTFGYGSALLGLTLALAMLINLVVAALAGVLV

PVVLDKLKLDPALASGTFVTTTTDMVGFFAFLGLATVILL*

>gkv_839|gene_guaD|guanine deaminase|

MTKQLLLGQVLSFDGNPFETEWTAVAHHRRRGAVLLEGGVIAAVDEADSLRARHPDAEVTDYGDALISAG

FIDTHMHYPQTGIIASWGKRLIDWLNTYTFPEEARFADPAYACAMANRTLDLALAHGTTTLTSFGTIHPG

SVDAFFEAAAQRGMRVVAGKTCMDRPETTPDFLRDTAQSAYDDSKALLDKWHGVGRALYAITPRFSPTST

EAQLRALGDLWRERPEALMQTHLSEQVDEVEWVKGLYPQSRDYLDTYEAFGLLGEGAIYGHAIHLTGREI

ARLRDAGASLAHCPTSNTVLGSGLFDLPGLAGKIRVGIATDTAGGNSFSMLRVMAATYEIAQLRGTAIHP

AQLMWLATEGGARALRQEGRIGHLGVGAEADLVILDLASTPAIAQRSARAADFWEALFPTLLMGDDRAVR

DVWVNGARMAARAG*

>gkv_840|gene_NONE|asnC family protein

MESVMSVDHQETDSIRQIRHRPADLDAIDRKILGALAVDASSSYAELSRVVNLSAPAVHDRVKRLKRDGV

IKSTVAVLDGCKLGRTLLTFLVVDTSSYSATRKLLKFTDRPEVEELHTVAGDGCVLIKVRAVDTEGLESF

LMEIQSLEGVRSVRSYITLSTFIERGPAPE*

>gkv_841|gene_NONE|inner membrane transport protein ydhP

MPVALFALAIGAFGIGLTEFVVAGILPQIAADFGVDIPQAGLMATTYALGVFVGAPILTVLGARVPRKAL

LIGLALIFTLGNIVTALAPTLPMALAGRILTAFNHGTFFGVGSIIAASLVARDKQASAIAFMFSGLTLAN

LVGVPAGTWLAQAFDWRLVFWLSAAIGVVTMTSIALWVPRIAGGKAIALRSELRAFIDPQVLLAMGITVF

GPAAFFTSITYIAPMMIHEAGFSDAGVTRIMVLFGLGLAVGNWLGGRFADRSLFGTLFVTLAAQALVLLV

FWLNVGSGVIASASVFFMAAFGFATVAPIQKLVMDRASAAGAPTMAASVNIGMFNLGNAMGAWVGGATIA

AGFGFAAPNWAGAILSLIALGLAFIAWASSATGRMAQQVR*

>gkv_843|gene_dgt|deoxyguanosinetriphosphate triphosphohydrolase, family protein|

MDWIARRSGFGKREGDYRTDGDIDYGRIVHSAAFRRLQGKTQILSLGDSDFYRTRLTHSLEVAQIATGLM

RTLNAHDPDHPAVPHLPDQSMIMAISSAHDLGHPPFGHGGEVALNYAMRDAGGFEGNGQTLRILTRLESF

SQSHGADLTRRTLLGVLKYPAALPVLQNPLLVPALKSGPTLFKTLDIAACKPPKGYFAAEQEAVDWVLAP

LSDSDRATFGDWVPKPGGHGKTLHKSLDCSIMDLADDIAYGIHDFEDAIALHLVTPELLRSAIRAELWQD

FMAGRRDADKKEKSNDPYGDLIAQLFGAGNTRKKIIGALVGYLVRPIAFVEKAAFTAPLLRWNVGLPDAQ

SKLLAALKDFVFQQVIDSPNVQHLEFKGQQMVVTVCEALQSDPKRLLPPDAYARYRAAADDPRVICDHVA

AMTDLHLMKTYERLFSPRMGSVFDRL*

>gkv_842|gene_hisN|histidinol-phosphate phosphatase HisN family protein

MTRVSPALAADLRRVAHLLADAARPVTLQHFRRGIAADNKGPAKGVAFDPVTIADRESEMAMRAILARER

PDDAILGEEFGHQPGNSGLTWVLDPIDGTRAYIAGAPTWGVLVAVSDDQGPLFGLIDQPYIGERFAGGFG

RAECVGPQGTSALQTRAARALSDAIVMTTYPEVGTPTEAAAFHTVAAQARLTRYGMDCYAYAMLAAGQID

LVIEAGLQSYDVQGPIAVIEGAGGIVTDWQGGPAWNGGRVLAAANAEVHAEALAILSQTL*

>gkv_844|gene_NONE|helix-turn-helix family protein

MAHTVDVYVGKRIRQRRWLIGMTQQQLAEHVGIKFQQIQKYETGANRVSASRLWEIAHALGTSISYFYEG

MNAAATEDSTLNEELWGREAMELLRSYYAIPEDQRRHIFELARVLSDAA*

>gkv_845|gene_NONE|bacterial regulatory helix-turn-helix protein, lysR family protein

VSRTLDLTALRSFVAVAENGGVTRAAGLLNLTQSAVSMQLKRLEESIGVALFERAGRGLALTGAGDQLLS

YARRMMVLNDEVYARLNARGYEGEVTLGVPQDVIYPVIPRILQQFARDFPLVQVHLISSFTLMLKEQFRR

GEIDVMLTTEDELGEGGETLAQRELIWVGAPGGAAWRKRPLPLAFERACIFRSFVQRRLDEAGIDWQMVV

NSESTRTIEATVSADLAIHTYIAGAEPPHLERIPHDGGLPDLRAFNINLYHAVHGESASVMAMVDLIRRA

YRAM*

>gkv_846|gene_NONE|conserved hypothetical protein

MSRLVALTSPVTARVSLIARLAFALRVRQERRHLVQLDDHLLKDLGLSRDEVQAEAGRKLWDTPKR*

>gkv_847|gene_NONE|uncharacterised protein family UPF0005 family protein

MAQFDTIRSSAGVRAGAIDQGLKAHMNKVYGTMSIGMVITALAAWAVSGLAVTTDPNAAAAQMGSRYLTD

FGYALYASPLKWVIMLLPIGMVFAFGALINRVSAAGAQLFFYVYAAAMGLSLSSIFLIYTGTSIVQVFLI

TAIAFAALSLYGYTTKRDISGWGTFLIMGLVGLLVAMIVNIFIGSGPLAMAISVLGVLLFAGLTAYDTQN

IKNTYIQHAVTGDSEWLGKAAIMGALSLYLDFINMFVMLLQLFGNRQE*

>gkv_848|gene_NONE|NADPH-dependent FMN reductase family protein

MTRILAIAGSLRAASFNAALLRAALAETPEGTTLDIGDMHGVPLYDGDLEEAEGLPAQVVALQGQLAAAD

GLLLVSPEYNGSIPGVLKNTLDWMSRGKGLAAFKGKPVAVIGASPGGFGTVLAQAHWAPVMRSLGMRPWH

DARLMVSRAGNVFDADLNLTDDAVRKQLADYLAGYAASLR*

>gkv_849|gene_NONE|biotin/lipoate A/B protein ligase family protein

MHGEYKVPGGKLVIADVEQVDGKLADVRISGDFFLEPAEALDDILAALNGLPADLSPAETTAAITAGLRP

GTQLVGFTAADITTAVRRALGLSKTWRDFDWQIIDGAPESPAMHLALDEVLAREVAAGRRAPTLRFWQWD

RPAIIIGNFQSLSNEVDLEAAQSHGIQTVRRVTGGGAMFVEPGSAITYSLYAPESLVADMDFAASYAFLD

MWVIKALNELGVDAVYKPLNDIASSKGKIGGAAQKRFAGGVVLHHATMSYDVDVNKMFQVLRIGREKLSD

KGITSAQKRVDPVRSQTGLPRDEVITRMKATFTDLNGGSTGAITADERRAAEALASSKFEDPQWLRHVP*

>gkv_851|gene_NONE|nitrogen regulatory protein P-II (PII signal transducing protein)

MKKIEAIIKPFKLDEVKEALQDIGVQGLSVAEVKGFGRQKGHTELYRGAEYVVDFLPKVKIEVVLPDEQV

EAAIGAIITAAKTDKIGDGKIFVSPVEQAIRIRTGESGDDAL*

>gkv_852|gene_glnA|glutamine synthetase, type I|

MTTYKLLELIKEEDAAYVDIRFTDPKGKLQHVTVVADLVDEDFIEEGFMFDGSSIAGWKSIDQSDMKLMA

DSDSAYVDPFYAEKTIAVHCNVVEPDTGEAYARDPRSIALKAEAYLKSSGIGDAFYCGPEAEFFLFDDVR

YSVSMNKVSFEVDGVDAAWNSDTKYEAGNMGHRPGVKGGYFPVNPIDDAQDLRSEMLSTMKRIGIKVDKH

HHEVASSQHELGMIFGGLTEQADNIQKYKYIIHNVAHAYGKSATFMPKPIAGDNGSGMHVNMSIWKDGKP

LFAGDKYADLSQEALYFIGGILKHAKALNAITNPSTNSYKRLIPGFEAPVLRAYSARNRSGCVRIPWSES

PKAKRVEARFPDPSANPYLAFAALLMAGLDGIKNKIDPGAASDKDLYDLPPEELAEIPTVCGSLREALTE

LAADMDFLTAGDVFTKDQLEAYIELKWQEVYAYEHTPHPVEYKMYYSC*

>gkv_853|gene_purB|adenylosuccinate lyase|

MIPRYSRPEMVAIWSPETKFKIWYEIEAYACDAQAELGVIPKANAEAVWRAKDVEFDVARIDEIEAVTKH

DVIAFLTHLAEHIGADDARFVHQGMTSSDVLDTTLNVQLVRAADILLADMDKVLAALKRRAFEHKDTVRI

GRSHGIHAEPTTMGLTFARFYAEMDRGRSRLLAARAEIATGAISGAVGTFANIDPFVEEYVCEKLGLAPE

PISTQVIPRDRHAMFFATLGVIASSIENIAIEIRHMQRTEVLEAEEFFSPGQKGSSAMPHKRNPVLTENL

TGLARLVRMSVTPALENVALWHERDISHSSVERAIGPDTTITLDFALNRLAGVVEKLVIYPENMLRNMNQ

FKGLVMSQRVLLALTQAGVSREDSYRLVQRNAMKVWEQGADFKTELLGDAEVTAALTPAQIEEKFDLGYH

TKHVDTIFARVFGAA*

>gkv_854|gene_NONE|fliG C-terminal domain protein

VQHIDHYSRATKAAIVVQALLREGQTPPLARLPEDVQVRLTHEISNLRLVDRATMNAVVSEFIAELEGLG

LAAPADMEGTLASVAQHLSPGAAARLRSEAAARVGSDPWAQIRQMKPDELAAPLSRESIEIAAVILSRLP

VNRAAEALSRIPGERARRVALAFSRANHVSPLALHRIGQAIVRDYCAIEAPAFDRPPDQRVGAILNASTQ

RTRDTVLEGLGEDDPAFADQVRRAIFTYAHIPTRLYPQDVPKILRKIDPPDLATVIAATQGQETDDGATT

AYLLANIPQRLGDTLREEAAAMDRPRRADVEKAMGVIVATLREAADQGEVNLMNTAPDDEA*

>gkv_856|gene_NONE|bacterial lipid A biosynthesis acyltransferase family protein

MAQTSPPADALDGSFADLVTDRALRGLLALAMALPYERRVAMMGAITRKVIGPLAGYRKRAEVNLAMIDP

QLTALRRRQIAGYVLDNMGRTLIENYSNKDLGARLATTEITGEGLDALAEAKAAGRPVLFLTAHFGNYEV

PRHILHRMGYVIGGIYRPMRNPYFNSHYIRTMEDVSGPVFPQGRRGTMGFVKHLRAGGMATLLFDVHDVG

GAPISYLGQPALTSLSAAEMALKFNALLIPYFGTRSGDGLSFTPTLEAPIPHSDPLTMMQEATRRLEARV

AANPEQWFWVHRRWKAPKTA*

>gkv_855|gene_NONE|hypothetical protein

MQRLISMLLNMFMGSKGANKSPQMRQARKVTKVLRRFTRF*

>gkv_857|gene_scpB|segregation and condensation protein B

MSDLTQAARMLEAVLFASRKPLNPRQLAARLPEGVDIQGALAALLTHYAGRGVELVRVGEAYAFRTAGDL

AHLMREEVEETRKLSRAGIETLAIIAYHQPVTRAEIEDIRGVSVNKGTLDQLIELEWVRLGRRRLTPGRP

VTYVVTETFLDQFGLETARDLPDLKEMRAAGLLESRLPPVVTVKSHETEALDPDSVAQDED*

>gkv_858|gene_NONE|scpA/B family protein

MADPVLDSVGERLEAEALIVDVGGFEGPLDLLLTLSRTQKVDLRQVSILALAQQYLAFIDAAKALRLELA

ADYLVMAAWLAYLKSRLLLPADPKAEGPSGEELAAHLAFQLERLDAMREAAAKLMARDQLGRERFTRGDP

GEMVRNRTLRWTASLTDLMQAYARLRTREEFRPFVLDRDAIMTLDQALERMRNLIGFAGDWTEISGYLPD

GWREAPEKWRSATAATFASALQLVKEGRAELRQDGTFAPIEIRKRGENV*

>gkv_859|gene_NONE|glycosyl hydrolase family 3 N terminal domain protein

MHHSAVIFGCAGPVLLPDEAAFFRAAQPWGFILFGRNVEDPAQLRRLTADLRAAVGWDAPILIDQEGGRV

QRLRPPHWRSFIPALDQMERARDPLRAQWLRNRLIADDLHRVGIDVNCAPLADLVEAETHSSLRGRLYGE

TVDGVVAGARAAADGLLAGGVLPVLKHIPGYGRATVDSHLELPSVTQPLEELLARDFAPFMQLNDMAMGM

TAHVVYTAIDPDLPATVSPAMIALIRQTIGFDGLLMSDDVSMNALSGTIAERAAATVAAGVDLALHCNGD

MIEMQAVAAAVPQMTVAATARATRALGQRRPPGDFDAGAAWADLQDQLNG*

>gkv_860|gene_NONE|sporulation related domain protein

MASITEGRFGGDDRQPPGGKGPRNVLQMVWLCVSLGLMVAVGVWGYRLLLREAAGIPTVHASTGPVRTAP

EVPGGSTAQNIGLSVNAVLARNPDSMMESDVVILAPQGAALSREDLSRQSSYLSAAGAMPSGISDDVLSA

GEVLQIAGADDPVESIDDLLSQLINPPVIAAAPEAAAPSSSGIPRPRTRPTNGAVTALAVAAAQAPVASA

PQSISADVPAGTPLVQLGAFPSTEGAANEWTRLSRNFADFLRGKTPVIQQATNGGNVIYRLRASGFTDLE

DARRFCALMVAENGACIPVSAS*

>gkv_861|gene_argS|arginyl-tRNA synthetase|

MNLFTQMRALVIACLDQLATEGVIPAGLPTDNVAVEPPRDAGHGDMATNAAMVLAKPAGVQPRVIADALA

AKLVLDPRVASAEVAGPGFLNLRLSADIWQGVVKAVLSDAGFGRSTLGAGKRVNVEYVSANPTGPMHVGH

TRGAVFGDALASLLAYAGYDVTREYYINDGGAQVDVLARSAYERYREAHGQQPDIREGLYPGDYLIEVGQ

ALKEKYGDTLLDKGEQYWLAEIREFATLQMMEMIRGDLAMLNVKMDVFSSEKALYGTGKIEAAIAKLEEQ

GLIYEGVLEPPKGKLPEDWEPREQTLFRSTLHGDDVDRPIKKSDGQWTYFAPDIAYHYDKVQRGFDLLID

VFGADHGGYVKRMKAAVSALSGGTVPLDIKLIQLVKLFKNGEPFKMSKRAGTFVTLRDVVEQAGADVTRF

VMLTRKNDAPLDFDFDKVLEQSKDNPVFYVQYAHARVHSVLRRAQAAGLDVSDAALIGADLTRVGHEAEL

KVMRQLAEWPRLIEIAARGNEPHRIAFYLYELASDFHGLWNRGNDDTALRFLQEGDAETSQAKIALIRAV

AVVISEGLGILGVTPVEEMR*

>gkv_862|gene_NONE|DEAD/DEAH box helicase family protein

MTEFSDLNLDPKVLQAVAEAGYTTPTPIQAGAIPEALAGRDVLGIAQTGTGKTASFTLPMIQLLSRGRAR

ARMPRSLVLCPTRELAAQVAENFDMYAKYTKLTKALLIGGVSFKDQDTLIDRGVDVLIATPGRLLDHFER

GKLLLTGVQIMVVDEADRMLDMGFIPDIERIFQMTPFTRQTLFFSATMAPEIERITNTFLSAPARIEVAR

AATTNQNITQAVVQFKGSSKEREPSEKRAVLRALIEAESATLTNAIIFCNRKIDVDIVAKSLKKYGYNAE

PIHGDLDQSQRMRTLDGFRDGSVKFLVASDVAARGLDIPSVSHVFNFDVPSHAEDYVHRIGRTGRAGRTG

STFMICVPRDEKNLAAIEALVQSTITRVESPLKELPPARKPRAEKPAEDKQTERPRREKPRLARNRPEAA

PVEDVVEAVAAPVAAAPAPAEKSRRRKPRRDEPQAEVAVAETPIAPREERHERHDHKEGRKDDRKHRRDD

GGVAGWGADVPDFIRLSFAERRKKVSA*

>gkv_863|gene_NONE|putative membrane protein

MLRLGLISSALLALPLALILATPAAAQDQTCTAHVNEQHLIIVEGTVTDRSSGWRERMTNAWNGRGRDGD

YPVCDSTVTIAFIGRMMGLEDTADYCLTRADEDSAWLLAPGARNYRGECRRTTCEYVNMAADASGDILRR

GAEIATGQEINDVGDGVTAVAGTAGTVMLTGQGGAIMNALGAGAQALTAAVSAPALLTAGAVTVVGVGGA

VYLCRD*

>gkv_864|gene_NONE|metallo-beta-lactamase superfamily protein

MSQERLIYLPLGGAGEIGMNAYVYGYGKPGEERLIVVDLGVAFPDMDTTPGVDLIFADVAWLEARRDRIE

GIIITHAHEDHIGAIGHLWPRLRAPIYARKFTGLIAAGKLEDTGAPIKSVLNIVEPWPAQTITMGPFSVG

FLPISHSIPESAGLIIDTPKGRILHTGDFKIDVNPVVGEAFDEALWSRAAEGGVKALMCDSTNIFSPHAG

RSESTLSTEIENLIASADGMVVATTFASNVARLKTIANAATAAGRRICLLGRAMRRMVEAALETGILTDF

PAVISPEEAADMPRHKLLLLVTGSQGERRAASAQLANGSYLGLKLKEGDLFLFSSKTIPGNERGVIRIMN

MLSEKGVDIVDDAGGRYHVSGHANRPDLQRMHRIVNPKLVVPMHGEHRHLREHVKLAEESNIPGFLAVNG

MMLDLTGDRPQVAEYIDTGRTYLDGSVQIGAMDGVVRDRIRMALNGHVTVTLIIDEHDEPLGDPWCETMG

LAELGRGNVPVVEAIEAELAKLLRRLDDKTLLDDDKLEKELRKSTRNAVNAEIGHKPEVTVIISRLV*

>gkv_865|gene_NONE|biotin--[acetyl-CoA-carboxylase] synthetase (Biotin--protein ligase)

LFTDPDWPAGVDRIIFDSIDSTMLEARRQLALGLTRPLWILAHDQTAGHGRRGRAWTHPAGNFAGTVVFR

PGGTPHSAGLRSFLMSNALRTALSAFVDPARLGLKWPNDVLLDGGKIAGILLESSAQGAAVDWLAIGVGV

NLAMAPDSDGAAFAPVALGAHPPHPEVFLTHLARAFAAQEAIFATDGFAPIRNEWLKHAVMRGKTITARL

PNVTYEGRFDGIDDDGNLMLTCADGPRTIAAAEVHFGPAPHQP*

>gkv_866|gene_NONE|NADH-quinone oxidoreductase subunit N (NADHdehydrogenase I subunit N) (NDH-1 subunit N)

MAADLALIWPEIALVLFAVLALMWGALRGQDREGPLLNTISAAVLAVIGVAVAFRGDGAGFNGLLQIDAF

ARFAQAVILLSAAAVLATGQSWLAGQGLMRFEYPVLVVLAVVGMMVMVSATDLITLYMGLELQSLALYIL

AAIRREDEASSEAGLKYFVLGALSSGLLLYGASLTYGFAGTTSLAGIAEAAASANIGLLFGLVFLLAGAA

FKISAVPFHMWTPDVYEGAPTPVTAFMAAAPKVAAMAMLARLVHTGFAPVLPDWQQIIAVLSVLSMFLGA

IAGIAQRDIKRLMAYSSIAHMGYALIGLTAGTTLGVEAMLAYMAIYVTTSVGAFAFILSMQRDGQPISSL

SALQGLGWAQPGRALAVMALMISLAGVPPFVGFLGKIYVLRAAVDAGLVWLAVAGVVASVIGAYYYLRIV

YLMYFTPAEGALETGKGWAAPVVLALAALAMVAGAVNLLGLESLAAMAAQSLVY*

>gkv_867|gene_NONE|NADH-quinone oxidoreductase chain 13 (NADH dehydrogenaseI, chain 13) (NDH-1, chain 13)

MLLSLVTFLPALGALVLALFTRGESPTVQQNARWVALGVTIATFAASLFILAGFDPAYDGFQFVEDRAWV

MGMSYRLGVDGISVLFVLLTTFLMPLVILTAWPVQDRTRTLMIAWLVLETLMIGVFTALDLVLFYLFFEA

GLIPMFLIIGVWGGKNRVYAAFKFFLYTFLGSVLMLVAMLVMYWESGTTCIAACGEGAIELIAYPFPVAG

GMQVLLFLAFFASFAVKMPMWPVHTWLPDAHVQAPTSGSVILAAVLLKMGGYGFLRFSLPMFPVASDMLA

PLMLWLSAIAVVYASLVALVQSDMKKLIAYSSVAHMGFVTMGMFALNRQGVDGAIFQMISHGFISAALFL

LVGVIYDRMHTREISAFGGLLNRMPAYGALFLLFTMANIGLPGTSGFVGEFLTLLGTYQANTWVALVAGT

GIIFSAGYGLWLFRRVIMGPILHKPLEAIQDITPREAAIFVPLVVMTILLGVYPSLVTDITGPSVQRLVD

HVAAELAAANLSANGGF*

>gkv_868|gene_NONE|NADH-quinone oxidoreductase chain 12 (NADH dehydrogenaseI, chain 12) (NDH-1, chain 12)

MEQAILLAPLAGAVLAGFGWRTIGQQAATILASALLVLAAVLSWIIFLGFDGDTRVIQLLRLIDVGTLHV

DWAIRVDRLSAIMLVVINSVSALVHIYSLGYMAHDDNFAHGESYRPRFFAYLSLFTFAMLLLVTADNLVQ

LFVGWEGVGLASFLLIGFYFRKDSANAAAMKAFVVNRVGDLGLYLAMMALFLSTGSLNFDVIFTRAPALA

EGGIPFLWREWTGAELVALLLFVGAMGKSAQLFLHTWLPDAMEGPTPVSALIHAATMVTAGVFLLCRMSP

ILEYAPLATGFVVVLGAATAFYAATVALVQNDIKRVIAYSTCSQLGYMFVAVGVGAYGVAMFHLFTHAFF

KALLFLGAGSVIHATHHEQDMRHYGGLRRDIPLTFALMMIGTLAITGVGLPLTTYGFAGFLSKDAVIESA

FAGAGFWPFAVLVLSAALTSFYSWRLIFLTFFGKPRYDEHHHHPHESPAVMWVPMAVLAVGAVLAGMVWY

SDFFGHADQVGAFFGIPVHDAGDHHYEFVGLPGEGALFIAAGNHVLEAAHEVPTWVKLAPFFAMLIGLAV

SVVFYLIRTDLPGKLAARLPGVHAFLMNRWYFDEAYDRLLVRPTLCLGRILWHKGDEGAIDGSINGIAMG

LIPRATRALSRAQSGYLFHYAFAMVVGVVGLLLWLFLFGGAR*

>gkv_869|gene_NONE|NADH-quinone oxidoreductase subunit K (NADHdehydrogenase I subunit K) (NDH-1 subunit K)

MMITQGHYLAVAGILFVIGVFGLFLNRRNVIILLMSVELILLAVNINLVTFSTSLGDLTGQVFTLFVLTV

AAAESAIGLAIMVTFFRKRGAIGVEDANVLKG*

>gkv_870|gene_NONE|NADH-quinone oxidoreductase chain 10 (NADH dehydrogenaseI, chain 10) (NDH-1, chain 10)

MAFIFYVFAAGTLIGGLMTVTARNPVHAVLWLISAFLSAAGLFVLQGAEFVAMILMIVYVGAVAVLFLFV

VMMLDVDFAALRAKSRAFVPVGVVIGAVLAAQLVMVFGDWSAAPGASARLATPTPAGVDNTHALALILYD

RYFIAFQLAGLVLLVAMIGAIVLTMRHRQDVKRQNVLEQMSRDPAKAIRMANPGSGQGLDRSKS*

>gkv_871|gene_NONE|NADH-quinone oxidoreductase subunit I (NADHdehydrogenase I subunit I) (NDH-1 subunit I)

MSQIDYTRAAKYFLLKDVWDGFRLGMKYFFRPRPTVNYPHEKVPLSPRFRGEHALRRYPSGEERCIACKL

CEAICPAQAITIDAEPRDDGSRRTTRYDIDMTKCIYCGFCQEACPVDAIVEGPNFEFATESREELFYDKD

KLLANGDKWEAEIARNLAADAPYR*

>gkv_872|gene_NONE|NADH-quinone oxidoreductase subunit H (NADHdehydrogenase I subunit H) (NDH-1 subunit H)

VLLILGQSLLVMVCILLALAFLMYADRKVWAAVQLRRGPNVVGPWGLLQSFADFLKYIVKEVIVPAGADR

FVFFLAPLLSFVLAMAGWAVIPFSDGWVLADINVAILYIFAVSSLEVYGVIMGGWASNSKYAFLGALRSA

AQMISYEVSLGLIIIGIIISTGSMNLSAIVHAQDGAYGIFSWYWVAHFPMLFLFFISALAETNRPPFDLP

EAESELVAGYQVEYSSTPFLLFMIGELTAVVLMCALVTLLFLGGWLSPIPGVADGIFWFILKVLACFFIF

ALVKATVPRYRHDQLMRIGWKVFLPLSLGWVVLVAFLAKFEILGGTWARWAIGG*

>gkv_873|gene_nuoG|NADH dehydrogenase (quinone), G subunit|

MQDLRKISIDGRVVEVPAAMTLIQACEAAGVEIPRFCYHERLSIAGNCRMCLVEVVGGPPKPTASCAMQV

RDLRPGAEGQPPVVLTQSPMVQKARSGVMEFLLINHPLDCPICDQGGECDLQDQAVAYGKATSRFTLPKR

ASDDLDLGPLVETHMTRCISCTRCVRFTTEVAGITQMGQTGRGEDAEVTSYLGQTLQSNLQGNIIDLCPV

GALTSKPYAFNARPWELTRTPSVDVMDALGSNIRVDTRGRDVMRILPRNHDGVNEEWISDKTRFIWDGLR

RQRLDRPYIREDGRLRAVAWDEALALAGTKLAGGKVAGLVGDLAPAEAAYALKLLVESLGGRVESRLDGA

ALPLQRGGYVGTASINDIDVARQITLIGTNPRVDAPVLNARIRKSWIKGADVTLIGPAVDLTYAYTHAGT

GRAALAALPREPVDGALFIIGASALTGTDGAAVLAEVFAAAAAQGGKVLVLHTAAARVGALDVGAATEGG

VQAALTGADVVYALGVDEIDIPEGPFVIYQGSHGDRGAHRADLILPAACYTEESGLFVNTEGRPQLALRA

AFPPGEARENWAVLRAVSGAAGRVLPWDSLAGLRQHLVQAHPHLARLDVVPENPAPDLPLGRLGSGDFAL

PDVDFYLTNPIARASTLMGQLAGQARKRAIAAE*

>gkv_874|gene_NONE|hypothetical protein

MRHILAAITIFATSLSLPAMAQNRPPLGQVPQVTEPLVVAAMVYEIDRVCGALNIRLLRGIGFLNGIKST

ARSLGYSNAEIDAFVDDRTEKTRLEEVARSRLRERGAIPDQPETYCALGRAEISAGSEVGRLLR*

>gkv_875|gene_nuoF|NADH oxidoreductase (quinone), F subunit|

MLSDQDRIFTNIYGQHDRSLAGAQMRGHFDGTAGIIAKGRDWIVNEMKASGLRGRGGAGFSTGLKWSFMP

KDSDRPAYLVVNADESEPGTCKDREILRHEPFALIEGCLVASFAINAHACYIYIRGEYIREREALQAAID

ACYDAGLLGANAAGSGWDFDLYLHHGAGAYICGEETALLESLEGKKGMPRMKPPFPAGAGLYGCPTTVNN

VESIAVAPTILRRGASWFSGFGRPNNAGTKLFAISGHVNRPCIVEEEMSIPFRELIDRHCGGIRGGWDNL

KGVIPGGSSVPVLPASVMGEAIMDFDWLRAQQSGLGTAAVIVMDQSVDIVKAIWRLSKFYKHESCGQCTP

CREGTGWMMRVMERLVAGTADPAEIDMLIEVTKQVEGHTICALGDAAAWPIQGLVRHFRPEIEARLHQGR

IAAE*

>gkv_876|gene_NONE|putative membrane protein

MNDSDKDQRVAAQGRRLAIGIVIVGVYWIAATWIGGAMGLTNRVRALLDLIALAGFGWVLWGAFQLWRAR

RDDSKG*

>gkv_877|gene_nuoE|NADH-quinone oxidoreductase chain 2 (NADH dehydrogenaseI, chain 2) (NDH-1, chain 2)|

MLRRLHPDQPASFAFTAANAAWAQLQIAKYPAGRQASAIIPLLWRAQEQEGWLTRAAIEHVANMLEMPFI

RALEVATFYFMFQLQPVGAVAHLQICGTLSCMLCGAEDLVSVCRQKIAAQPHSLSDDGKLSWEEVECLGA

CTNAPMAQIGKDYYEDLTAEGLSDLIDALRAGDVPQPGPQNGRFSAEPLGGATVLQNTPGDKQALNASLT

LATTINDTIKRIDGTEVPLRHPRGETTL*

>gkv_878|gene_NONE|putative lipoprotein

MVIRFVKPLALLVVGAALAGCTADMFPSMTRAQGPAGTAAPVAPAPQTPPASNLPPADRLVASIESEGCL

LRQNNVASVLLRANLTQQELMDLAPQLAASGRAEVTSDGSVRVISPRCA*

>gkv_879|gene_nuoD|NADH-quinone oxidoreductase chain 4 (NADH dehydrogenaseI, chain 4) (NDH-1, chain 4)|

VRNFNINFGPQHPAAHGVLRLVLELDGEIVERCDPHVGLLHRGTEKLMESRTYLQNLPYLDRLDYVAPMN

QEHAWCLAIERLAGIEVPRRASLIRVLFCEIGRVLNHLMNVTTGAMDVGALTPPLWGFEEREKLMLFYER

ACGARLHAAYFRPGGVHQDLPPDLIADIDAWAIGFPQFLDELSGLLVESRIFKQRLVDIGNISADEAIAM

GFSGVMVRGSGLEWDLRRAQPYECYDEFEFDIPVGTKGDCYDRFLCRVAEMHESTKIIRQACEKLRNCPG

EVMAYGKLAPPKRAEMKTSMEALIHHFKLYSEGFHLPAGEVYAAVEAPKGEFGVYLVSDGSNKPYRAKLR

APGFAHLQAMDHMTKGHLLADVTAIIATLDIVFGEVDR*

>gkv_880|gene_nuoC|NADH-quinone oxidoreductase subunit C (NADHdehydrogenase I subunit C) (NDH-1 subunit C)|

MQDLATYLRDMAPGAPTAQLAYGELTLTSTLTDLPALLMFLRDDVQCRFSTLVDITAVDYPARIKRFDLV

YHFLSMHLNHRLRLKVAVADGEMPPSAIPAYPAADWFEREVFDMFGILFTGHPDLRRLLTDYGFSGHPLR

KDFPTTGYTEVLYDEVEKRVVHAPVQLVQAYRQFDFLSPWEGMGNTLPGDQTGKGDAT*

>gkv_881|gene_NONE|NADH-quinone oxidoreductase subunit B (NADHdehydrogenase I subunit B) (NDH-1 subunit B)

VQDKGFLVTSTADIINWARTGSLHWMTFGLACCAVEMMQLSMPRYDLERFGTAPRASPRQADLMIVAGTL

TNKMAPALRKVYDQMPEPRYVISMGSCANGGGYYHYSYSVVRGCDRIVPVDVYVPGCPPTAEALLYGIMQ

LQRKIRRTGTIVR*

>gkv_882|gene_NONE|NADH-quinone oxidoreductase chain 7 (NADH dehydrogenaseI, chain 7) (NDH-1, chain 7)

LLQDYLPILIFLGLATVLGIILILAAVVVAVRNPDPEKVSAYECGFNAFDDARMKFDVRFYLVSILFIIF

DLEVAFLFPWAVSFAGMSMVSFWSMMVFLAVLTIGFVYEWRKGALEWN*

>gkv_883|gene_NONE|HAD-superfamily hydrolase, subfamily IA, variant 3 family protein

MRSVIFDLDGTLADTSLDMINAGNAALEGIGIAGSLGPDDAGTAFGGGMALMNLGFQRAGRKDVPVKSIH

YPLFIKAYWGALSSHTVLYPGVADAIEGLRSAGYAVGICTNKPAAPADELLRRLGVRDLFGSLVGADTLP

VKKPDPAPLFEAIRRLGADLSRGCLVGDTITDFKTARAAGVPSVLVTFGPGAKIVADLAPDATISSYVEL

TQVIDRLNL*

>gkv_884|gene_glmU|UDP-N-acetylglucosamine diphosphorylase/glucosamine-1-phosphate N-acetyltransferase|

MPSNFVILAAGKGTRMESDLPKVLHEVGGAPLIAHALRLADSLLPDRVIVVTGYEGAAVAKSAAHWNPEA

IIVEQAEQLGTGHAAQMAMPALTGTSGDVFVTFGDTPFIRTATLEAMQDARKTRDLVVLGFEAADPDARY

GRLVMDGEDLQAIVEYKDADAATRAIRLCNSGVICTSAQRLPALLAQLKNENAAAEYYLTDIVAIARAAG

LSAGVVTCPEEETLGINTRTELARAEVIFQARARRDALEDGVQLVSPDTVHFAFDTLLGRDAVVEPYVVF

ATGVTVESGARIRSFSHLEGAHVSRGAVVGPYARLRPGAELAEHAHVGNFVEVKNATIGEGTKASHLSYI

GDADVGAGTNIGAGTITVNYDGVFKHRTTIGDRAFIGSNSTLIAPITIGNEGFTAAGSVITDDVAPGALA

IGRARQVEKPGLARALMARLRAKKDKT*

>gkv_885|gene_NONE|hypothetical protein

MSLPDDLQCRLVTLEEELRVEGWDPTPAEMIDWLARELRPANELPARLELAARLWAFGTQDARILAAKLL

TQARMRPDDAIWAALTGWIAELGAGAEEWALIESVSRALDRRIEAAPERLAEITPWVSHENAALRAAFAL

VARPNLRLKMPKAADLARRDELLVLLAPLAADRDQMVQRALASALRDLAKHDAPRATTFLLAHGNAMVPW

ARRESIGRLPIALDDRGRITGAPDNH*

>gkv_886|gene_NONE|hypothetical protein

MGINNERDIEANIQIGPTDRGMVRIFIEAGTVEIPMDFDPEEAEEIAEEILAAAAAARAANGGGKGKKKR

*

>gkv_888|gene_NONE|hypothetical protein

MTDVTTCLICAAPMRYYFTKTYNDLPAALPYAGFSADYHRCDACGFVASKTHQDMSDDAFGALNNAAHAY

FEALPLAQRGFNQPPYAEQALALAILGRNGIVDLGRTLDYAAGHGTLAGIMARYFDHHIATYDKYIASDG

HDPARPPQPARLVINSAMFEHVTTRAALDALNDLVTSDGVLMLHSVICDRVPADPDWFYLRPIVHCAFHT

NASMEVLMQQWGYAASIYAPKAKSWFLFKAGYPDLEHLPAQIAALNTEFQTEMFIYKSGFVDFWKGF*

>gkv_889|gene_NONE|conserved hypothetical protein

MLKYLLPVAVMLCAAPAVAQYVGPAGAPEGVSPGYPLTTVAEIKADPRDDANVTLEGFLIRQIDRETYVF

RDDTGEIEVEIDEDDFPRQPVSETTRVRIEGEVDTHRLRDTDIDADRVMILE*

>gkv_890|gene_katG|catalase/peroxidase HPI|

MDGNTTSQGKCPVAHGAMTETGKSVMEWWPNALNLDILHQHDTKTNPLGADFNYRAALKDLDVDALKADL

RALMNDSQDWWPADWGSYVGMFARVAWHAAGSYRTADGRGGANTGNQRFAPLNSWPDNVNTDKGRRLLWP

IKKKYGNKISWADLIVLSGTIAYEVAGLKTFGFAFGRQDIWAPEKDTYWGNEKEWLAPSDERYANVEEPA

SLANPLAAVQMGLIYVNPEGVNGNSDPLKTAAMMRETFARMGMDDAETVALTAGGHTIGKTHGNGSAADL

SADPEAAAPEFQGLGWMNTKGRGIGRNTVVSGLEGAWTTEPTKWDNGFFTMLFNHEWHLVKSPAGAMQWE

PITIADADKPVDVEDDAIRHNPMMTDADMALKVDPIYREISLRFMNDFDAFSDAFARAWFKLTHRDMGPK

SRYVGPDIPAEDLIWQDPIPAGATGWDVAAVKAKIAASGLSVQDLVATAWDSARTYRGSDMRGGANGARL

RLAPQREWEGNEPARLARVLTVLEDIAASSGASIADVIVLAGGVGIEQAAKAAGFDVTVPFTAGRGDATE

AQTDVDSFDVLEPVADGFRNWQKADYIVSPEEMLLDRAQLLGLTAAEMTVLVGGFRALDVNYGGAKTGVF

TDRAGSLTADFFVNLTDMAYQWVPTGKDLYEIRDRRTGAVCWTASRVDLVFGSNSILRAYAEVYAQDDNA

GKFVQDFIAAWVKVMNADRFDIAA*

>gkv_891|gene_NONE|bacterial regulatory protein LysR, HTH motif:LysR substrate binding domain

MKNLTIKHLRYFTALARHGHFGRAAEVCAISQPALSLQIKELETITGVPLIERGARQTHLTPLGEEFAAR

AQSILQAMDELGDLARAASGDISGRLRLGVIPTVTPYLLPRLVKTLSARYPALDLQPRETVTRKLLDDLL

AGSLDAAIVALPTSEPSLHEEVLFDEDFVLVRPATDANRPVPSADMLREMRLLLLEEGHCFRDQALSFCS

IDKMRARDLMEASSLATLVQMVSAGIGVTLIPEMALEVETRGAKVTVSRLAPPQPTRRIGMVWRRSNPLG

RQLTQIATLVREVWSQSERPQH*

>gkv_892|gene_NONE|tonB-dependent Receptor Plug domain protein

MVPAVASAQSTATDLGLIVIGSASGVATTLQDAPAAVTVIDSETIAAEGARDLNDILRTIPGLNLTQGNS

GIGEISMRGLPANRTLTLVNGRRVSTGGTVVRDYLGDLTRIPLDAIDRIEVVRGPMSTLYGSDAMGGVVN

IILKEPTDVWSGSVTAEYSSGPADTTADNAQISAYFSGRLTQHLTAAFWGKVYEREATEDYSYTTDAGAA

ATVTSGEGSRTKELGANLIWSPNDTIEWGIELTASDERYLSYQAHDTRRLQTYDLTLTNEWQLGAGQLSS

YLSYQNSQNAPWDTTNTTWKDATEYNTINFETRYSNATQIAGRYLEYTLGLNLSHDELSDTNTNSSGALI

EGEMSTAALYAEARYDMTDSLRLTYGLRADSSEEYGTHVTPRVYANYDFGNGLVLKAGFAQAFNAPDLRS

LNPNYLLTSRGNGCKPYPGPCYITGNPDLVPETSDSYEIGLNYQGSDVSWELTAFYNELTNMFGAAKTGE

TSSNGYAIFERTNIDEGTTAGLEGGLSWNISPDLTWTNSFTYLAKSEFYYEFLDTPFPMATTPELNITTA

LNWQANDALTLGASVTYVGKQVGYITEEELSSEEARAVPAGQNSDPYMLVDLSLAYDLSDNARINFGIDN

VFDAQPDDTVSYRETGRLFRIGITTSF*

>gkv_893|gene_NONE|hypothetical protein

MLCWLSLANAAEVADFTYKVQGIYGIPQISNEFCQEYVATASHISITFP*

>gkv_894|gene_NONE|NLPA lipofamily protein

MRSLTLATRLGAGLAAFAWFGPAAALTIGVIPGAASDSIEAAAVDARAAGMEIEVIEFSDWTIPNVALTS

GDLDLNYFQHGPFLAAQVEATGADLVSVGSGYQPRIGLYSNRYDSLDALPDGATVGVASDPVNQGRGLQL

LQAAGLVTLAEGKGYLVSVDDIIQNPRNLNIVEIEGTQLVHAMQDLDLVQGLPAQIVNAGLVEKAGQAIV

FSDQEVADFSIQFVTRGEKADDAEIQQFIAIYYNSEAARAAIHQAFASNENLYSLRWLQD*

>gkv_895|gene_NONE|rhodanese-like domain protein

MSSRHSEYLISTADLQAALADPDLVILDATTTLVPNAARTFDIVTGQADFEAAHIPGAQFVDLERDLSRP

APGLLFTLAEVADFAAAATRLGIGAASRVVVYSSAQPGWAVRLWLTLRAYGFSQVKVLDGGLMAWRAEDR

PVDTGPARPRPLPVQPFAWRDDRAAFFTDTARVEQAVTSGDAILVNALGRDYFAGTAAITYGRKGNIPGS

RNLPTSALVGADGRFLSADDLAQLYAAEGIAADQATITYCGAGVAASNVAFGRLLLGLDDTRVYDGSLME

WAQDPARPLVP*

>gkv_896|gene_NONE|ABC transporter family protein

MPSASPPSAVRFDGLGKIYHRGGQDVTALSDITLDIAKGEIFGMIGRSGAGKSSLLRTINRLEQPTSGRV

LVDGQDIAALDEGGLVALRRRIGMVFQHFNLLSAKTVRENVGLPLKVAGVPAREIATRVDEVLALVGLEG

KGDSYPARLSGGQKQRVGIARALISRPDILLCDEATSALDPETTLSILSLLGDLHRQLGLTIILITHEMS

VIREICQRVVVLDQGRIAEEGPVWRVFGAPAHPATQSLLQPLARALPQDIAARLRSAPQRGDHVVIELRF

DGGQQVQMDAIASALPGARILGANLEQIGAHLVGQVVLALPQQEQPVQIPSFAATTKVIGYVAAND*

>gkv_897|gene_NONE|D-methionine transport system permease protein metI

MSPQMINRLWQAFFDTLFMVGTSAVITVVVGIPLAVFLVISAPGGIIAAPLANRIIGLVVNGFRAVPFIV

LMVALIPFTRMLVGTTIGVWAAIVPLSIAAIPFFARIVEVSLREVDPGLIEAAQSIGCRRSHIIRHVLLP

EALPGIVGGLTITVVTMIGASAMAGAVGAGGLGDVAIRYGYQRFDTTVMVIVIVILIALVSLLQFLGDYT

VRRLRNR*

>gkv_898|gene_NONE|hypothetical protein

MPSERPARAGRDNEKRPRERVARGAVTFADGPPPTAHRSRNR*

>gkv_899|gene_NONE|conserved hypothetical protein

MNSEFTFSLKSIRFDEDYHPATNTRATTNFANLARGAHRQENLRNALTMINNRFNALADWDNPDSDRYGL

ALDIISVEIAGGADAFPLIEILKTTIIDHHRNTRIDGIAGNNFSSYVRDYDFSVLLPTLKKRAPDAALPD

TFGDLHGKLFQCFVNAPIYRDTFAKAPVICLSASSSRTYQRTDNWHPILGVEYRQDAPSLTDQYFAKMGL

RVRFFMPKGSVAPLAFYCAGDVLGDYSNLELIGTISTMETFQKIYRPEIYNANAAAGKVYQPSLTHRDYA

TTQITYDRDERSRLGIAQGKFAEEHFIKPNQFTLAQWSAAQSF*

>gkv_900|gene_NONE|metE

MTKLLQTSTAGSLPKPAWLAQPETLWSPWKLEGEGLVEGKQDALRLSLADQQHAGIDIVSDGEQTRQHFV

TTFIEHLSGVDFQKRETVRIRNRYDASVPTVVGAVERQKPVFVEDAKFLRAQTKQPIKWALPGPMTMIDT

LYDAHYKSREKLAWEFAVILNQEAKELEAAGVDIIQFDEPAFNVFFDEVNDWGVATLERAIEGLKCQTAV

HICYGYGIKANTDWKKTLGAEWRQYEDVFPKLQKSNIDMISLECQNSHVPMDLIELIRGKKVMVGAIDVA

SNTVETPEEVANTLRKALQFVDAENLFPCTNCGMAPLSRAVATGKLAALSAGAEIVRRELSA*

>gkv_901|gene_NONE|glutathione import ATP-binding protein gsiA

MTPVLSLRDLRVAFSVDGDWREVLHGISLDVMPGETVAIVGESGSGKSVTSLSIMGLLPKTSARVSGSIT

LNGRELVGLPQTQMQKLRGGDMAMIFQEPMTSLNPVFTVGQQLAEAISAHRKLSAAEIRAEAIRLLEKVR

IPNAEARLKAYPHEFSGGMRQRVMIAMALASKPKLLIADEPTTALDVTIQGQILDLIKTLQEEEGMSVLF

ITHDMGVVAEVADRTIVMFRGDAVESGPTAQIFTQQQHPYTRALLAAVPSMGAMEGQAHPLRFGIVDKET

GLASPEVPLDTPVDTSAPILSLRNLVTRFDVKGGWLGRKTGAVHAVENVSFDLFPRETLSLVGESGCGKS

TIGRSIMRLTDSIAGEILIDGEDMRTMPKSRLNSLRRQAQMIFQDPFASLNPRMTIAEALTEPFLTHKMG

SRAEARAKAEHLMEQVGLSPAMLSRFPHEFSGGQRQRISIARALVLDPKIIIADESVSALDVSIKAQVVN

LLMDLQDRLGLSYLFISHDMAVVERVSHRVAVMYLGEIVEIAPREALFANPQHAYTKKLIAAVPVPDPAR

RGLRRAISNDEIKSPIRAVDYVPPKRHYREVGAGHFVMEA*

>gkv_902|gene_NONE|gamma-glutamyltranspeptidase family protein

MTKGAISSTHALATQAGADILAAGGNAFDAAVAAGLVLQVVEPHLNGPGGDLPAIFWKDGKAQVLCAQGP

TPAGATIAHYKAEGLDLIPGDGLLATVIPGAFDGWMLLLRDHGTMELADVMRAAIRLADEGHPILDRAVD

TVAKLGDFFTQHWPGSAALWLQGGVPPQKGGLFRNADLANTWRRIVDLAQGETREARIDAARDAFYRGFV

AEAIGEFCAGFKAMDESGTAHSGVLTAADMAGYRAGYEAPLTYDYNGWTLAKTGPWGQGPVLAQALSLLP

ADVMAGIDTTSADFVHLVIEAQKLAFADRDTYYADPAVVDVPMDALLSDAYASARRAQISDKADNTLRPG

TAPGFAAQVAAMADALERLSRPEGAVYEPTMAHLATDKGTIPRGDTVHLDVIDQWGNIVSATPSGGWLQS

SPTIPGLGFCLNSRAQMFWLEPDLPGSLAPGKRPRTTLSPSLAWHQDGRILSFGTPGGDQQDQWQLLFFV

RFAAQGLDLQAALDAPMFHSTHMPASFYPRSREPGGLVAEGDLPDETLEALRAKGHVLRVVPKGSVGRLT

AALRHPDGRLEAAATYRATYGGAKVLV*

>gkv_903|gene_NONE|glutathione transport system permease protein gsiD

VTDTVVPTAADVPPRRQNRTWRKFKRNRAALVGAIVVLFFAALALLAPLLPIPNPTATDWMLVRKPPSAA

HWFGTDELGRDILSRMIWGARASLQAGVISVGIAVAFGVPLGLLAGYFGGWLDQIIARITDAMLAMPFLI

LAIALAAFLGPSLQNAMIAIGISAMPVFVRLARGQALSVKTEDYVEAARSIGISNLRIMGRYILPNIFPP

ILVQATLTIATAIIAEASLSFLGLGQQAPEPSWGAMLATAKNFLTQAPFMAVAPGIAIFLVVMGFNLLGD

GLRDALDPRDNG*

>gkv_904|gene_NONE|dipeptide transport system permease protein dppB

MMRFVLNRVLVAIPTLILVSIFVFMLQKLLPGDPILVMAGESRDPATIEMLRERYHMNDPVLMQYFYWLG

DVLRGDLGRSLRTGLPVSDLILQKLPVTLQLAVMSMIFAMLIGVPAGVIAAVKRGTLWDYLANGVALSGL

SVPNFWLGIMLILLVSVNLGWLPASGYESPFVDPWRSLQTTIMPAIVLGTAIAGTLMRHTRSAMLGVLQA

DYVRTARAKGLSEKIVVLRHAFRNALLPIVTLSAVMFGELLAGAVLTEQIFTIPGFGKMIVDAVFNRDYA

VVQGVVLVTATSFIVINLLADVLYVMLNPRMRATL*

>gkv_905|gene_NONE|bacterial extracellular solute-binding proteins, family 5 Middle family protein

MKKSLLMATLFASVSLAGGAFAQDLRIGLNEDPDSLDPAQSRTFVSSLVYEQLCSRLFNTDQNMQIIPEL

ATSHSWSEDGMTLVLQLREGVTHHDGTPFNAASAVRVFERNMNLPVSNRKAELGSVESVEATGDHELTIH

LKSADVTLLAQLAHHAGRMYSPDAADAAGENFGQNPVCSGPYSFVSRSEGDRIVLEKFADYYDADEFHYD

RVIFMPIPDTTVRLANVRAGDLDIVERTAPADVPSVQSDSRLQLFQIPNIGYQGITINVGNGARAEGPLG

SNPLVRQALSLAIDREAFNQVVFEGLYVPGNQWAAPGSGWYDERFPVPARDVDRARALLAEAGVELPLRV

ELQTGNSPVAMQAGQVIQAMASEAGFEISLRATEFATLLSENVAGNFDMSLQGWSGRIDPDANIHPFVHT

TGSNNDEHYANAEIDQILDQARQEPDPAVRKELYDRTTEILQNDLPLLYLYHIQYFFTLRDGIEGFAPYA

DGIIRLRGVQG*

>gkv_906|gene_NONE|tonB-dependent Receptor Plug domain protein

MGQVKSRLRLVKALMLTSVAAATLALGAPVLAQDGPVLALQVPAQDLGVALTAVGDQAGVSIFFPSGIVA

GRQSPALSGDYSVEQALVQLLAQTGLTYQFTSPTSVVISQPLADVDSDTLVLGPVRVVASSAAASAVEAA

AERVQDTYRGVAASAHLSAEDIAAARGASPGDMLRGIAGVMNAENRNSGALDVNIRGLQGQGRSPVVLDG

ALQESTVYRGYSGMAGRTYIDPDLIGGLSIEKGPSMGADATGAVGGVVRARTLSPHDILAPDGTWALRLN

AGLTGNNDTPPTATTVGGSEPAVRNYDRPDFLDLNGHNASAAFAYRTQTADFLAAYAQRENGNYYTGERG

IPVSEWNGGAHPFENGEQVINSSIETTSYLLRGVFRPVYGHTFDLSYLRYEGLSAEMKPSQLMYGDTPYQ

TVTDVSVDTYTARYRYNTGNPLVDLRADLWATRVDSFNIDPVRLDYGSFQYNGDMFAATLSERWGATVHN

TSVFEGAPGRLSLNYGFAYDHEDFGKSDDWERLNALYPGRSWDPVRTGWRDQTSAFINATYNPADWATFT

LGARYIHNEVTDSNTGSSWVLGGISNHDAAEGIAPAFSALIEPVQGLQFYGRYAEALRAASPFEATEGFS

GSVNPYWDLKMEHAHNTELGINVNRFAVFRPDDAFSAKLSWFNNEITDYITLGQERLTAPNGNSTEIQVR

TNIPEVSMRGWELSARYAVGSSYLALGATEYTDISSCYRSTASQPISCYDGMPQTSASWFVNHIPPERTF

SATVGTALMDERLNLGLRYNRVVRAPAYELFDLFGSYQINDRTSLSFAVDNLFDIYYVDALSLGEGVAVL

PAPGRTLSLNFTTTLGDGTPERSSSAAVRNYMAAQADARSSAVQPFDGNWGGAYAGLTFGAARFAAEGET

YAGDGSYNANAAIERTDTAASSAVAGLQFGYRKQQDSGLVLGVEASVDFARGRAQQYMIDETLDVDRWGE

NNTRAADYTHSWGATAMLRGSVGQTFGRAHLFATAGLGMMEEHQTRTQYRLVSGIVNYPSFSETDSQLRT

GVVVGAGMDYAFSNALSLRGEYVYGYYPEKNFNFDRASNGTTAAGLLDQIGRQAGSELHTHALRIGLNYR

F*

>gkv_907|gene_NONE|fecR family protein

MPRSFPDTALSDEALDWIIRLHAGGATEADWQAWALWRAQGDMFDAAAREAEALWHGLGAAGHQVKRGAL

TRRTVLGGGAAVVLGAGLLQTGALLPDYSTMAAERRDIVLADGTAVALNARTALNLDARGVAMLRGQATF

DAARDLLLTVPGGEVHAAAARFDVDLLAGGLALTCLEGAVTLHFGGRSARLQGGERLFTAGWQRARVNPA

DALAWQRGKLILDQRPLAELAASLERYRRGRIVIWGDQVADLRVSGVFNMADGDLILQSLAGPLPITFTQ

LPMFSVIRRA*

>gkv_908|gene_NONE|RNA polymerase sigma factor, sigma-70 family protein

MPHEILDDDAHRMMDLYLQRWDLLHRALRKRVGSHDLADEAMQETWFRLRRVVQRREAVRDPKAYILMVA

ANISIDLIRRERRHIIGHDSDAAVIGAVADEMPTAEAVLIGRSQLRQLVQVLMGLKPKAREVLIMNRCAG

MTHREIAAQMKISDRMVAEYMTQALRHCRDAFRALED*

>gkv_909|gene_NONE|acetyltransferase (GNAT) family protein

MTAPYRLPTTFQTSRYHLRQATPDDAQAIFDAYGTDRNVTRYLAWRPHESVSDTAAFLQIAADQWDRGTG

FAVVARPLAQPDQIIGMFHPQLFGHRLSYGYVLRASAWGQGCASEVMRWLVDHALSHPAIHRAEAFCDVD

NPASARVMEKAGMQREGLLRRYFVHPNISDAPRDCLIYAKVR*

>gkv_911|gene_NONE|conserved hypothetical protein

MPKAEWGTKRLCPTTGKRFYDLNANPIVSPYTGLVVSIDTARGRSIMADAADGAKKLKGFDEDDDLLLDD

DVEGDDADLADEVLEDDDEDNVSFDDLGDVAANDDE*

>gkv_912|gene_NONE|conserved hypothetical protein

MSHPQPARNAHRASLKTQVKTLPMAPTPAQFTIDADGPIAVQLRRNARAKRLTLRVSQHDGAVTLTLPPR

ASLDEALRFAQSRAEWIRRHLPMGGPRRVMIGDSLPLRGTAHVITAGTGRGVRAEGGALIVPGAASLGPR

LAAYLKVQARDDISRAADHYARLARRPITRLTLRDTRSRWGSCTSDGALMFSWRLVMAPPPVLHYVAAHE

VAHLLHMDHSPAFWAEVARLFPDHASARQWLRQHGSHLQRIDFTAPE*

>gkv_913|gene_NONE|bacterial regulatory proteins, gntR family protein

MVTHPVSAPPEATARPTADATIAAHERVYKQLRLMIVHGELPPGEALTLRGIGAQFGVSMTPAREAVRRL

IAEGALVMSSSGRVATPELSNERLEELASLRALLEPELASRALPRAHFALIDRMEVINQGISQAIARQDP

VGYIRMNLEFHRALYLRAQAPAFLAMTETVWLQLGPTMRKLYGRLRRTEPPQHHRLILAALRAGDEPSLR

LAVRADVTQGLRMLKN*

>gkv_914|gene_NONE|hypothetical protein

MAIFAPVQPEPAEGQAWSSLRSELMTLIQDFHLTDDISLERMFDRIEETETLQ*

>gkv_915|gene_NONE|hypothetical protein

MMAAPLSPEITKALVAEQPTYDARELTGKNGQARIILGDQVYNLRITRAGKLILTK*

>gkv_916|gene_NONE|FAD dependent oxidoreductase family protein

MQDAYDVVIIGGAMTGSSAAFWLTRNPDFTGRVLVVERDPTYAWAATTHTNSCIRLQYGSEVNVRISRFA

GDFIHQFKEYMQDDAAPDIVLQNFGYLYLANTPAFLDVLRDNAQMQRALGAETQILTPDEIAALFPFYNL

DGILGGSFNTKDEGYFDGGTMFDWLRIKARAQGATFLTDTVTAISADNRITLASGRTISAGHIINAAGTR

GAEVAAMAGLRIPIEARRRFTFIAEAARPLPVDLPLTIDPVGVHMRSDGRYYMIGCPPDLDVAVDPDDFT

MDHAIWEDHVWPTIAARIPAFEELRLINTWVGHYDYNTLDQNAIIGPHPERRNFLFANGFSGHGLQQAPA

VGRALSELVIYCATRTLDLGALGYERIASGTPLRERAII*

>gkv_917|gene_NONE|short chain dehydrogenase family protein

MRKIVLTGARGALGTSLRGPLSKMCDQLVSTDIQPGPEGLYDNETFHIADIAKFDEIAPLLEGADMVVHF

GAIVDEKPFEELLGPNFVGAYNIWEAAHRHGVRRVVYASSIHAVGLEHTNSGADTTVAHNPDTFYGLAKC

FAEDLGRMYWQKRGVESVCLRILSCTPEPQNIRALGTWLSHRDMVQLVTRAIDSPVVGFTVIYGVSNNTR

SPVDNAKAAFLGYRPVDNAEDWAHSLFAKAGIPDPQDKALTRLGGPFAVVPLGESGVAAIQKMSEGQKP*

>gkv_918|gene_deoC|deoxyribose-phosphate aldolase|

MTSLTRNPGMALDLDWVLGARVNTPAITRRCASLPARRSVKKDYQAAWLLKAVSLIDLTTLSGDDTADRV

RRLCAKARQPIAPEVLDRLGMSGLTTGAVCVYHDMIGAAVDALQGSDIPVAAVSTGFPAGLSPWHLRLQE

IRESVAAGAQEIDIVISRRHVLSGNWQALYDEMLAMREACGEAHIKAILATGELGTLENVARASLICMMA

GADFIKTSTGKEAVNATLPVSLTMIRQIRDYHDRTGIYVGYKPAGGISKAKDALTYLSLMREELGLRWLQ

PDLFRFGASSLLGDIERQLDHYVTGGYSAAYRHALA*

>gkv_919|gene_NONE|aldehyde dehydrogenase 5, mitochondrial

MPTLSEIFETMEYGPAPESASEALAWIATHEGRFGHFINGTFTAPAATFVTVNPATDAELAQVSQGSAAD

IAAAVAAARAAQPAWEKAGGPARARVLYAIARLLQKHARLFAVLETLDNGKPIRESRDADIPLAQRHFYY

HAGLAQLFEAQNPNARAHGVCGQIIPWNFPLLMLAWKVAPALAAGNTVVLKPAEYTPLTALLFAEVCQQA

GVPAGVVNIVTGDGDTGAALVAADVNKIAFTGSTEVGRIIRQATAGSGKALTLELGGKSPFIVFDDADLD

AAVEGVVDSIWFNQGQVCCAGSRLLLHEPIAARFIAKLKRRMESLRIGDPLDKSIDIGAIVHPVQLQRIT

ALVGAHTAGEVHTAPCPLPARGSFYPPTLITGLSPSDALMQQEIFGPVLVSTTFRTPAEAVELANNTRYG

LAASVFSENINTALAIAPQLEAGVIWVNGANMLDAAAPFGGMRESGYGREGGPEGLAAYTRMTNHAAPAL

KSKPVALPVLEQVDRTAKLYIGGKQARPDSGYSIALHDTTGALAGHVGAGSRKDLRNAVEAMNAAKGWAK

TTGHNRAQILYFIAENLSARAAEFAALIDSFGGAGAAEVSAAIDALFTAAAVADKYDGRVAGVPFGGLAL

ALQTPVGKIAALCPEDSALLAPVALIAGALALGSRITLVPSARYALVATALYQVLDTSDVPGGVVNIITG

VQSDLAAPIAQHADLDAAWVYGDQALVAEVERLSASNLKRVSSGPSAPDMAQILRAASEVKTIWIPFGI*

>gkv_920|gene_NONE|putative lipoprotein

MKALIPFAALMALAACSSPTLYTAEAPVPLDQRASIAFSTVEVAELSLPRYASGPDIYSEGAGGALTAMS

GVNWADDPAPAMTASLVQVLTGMTGARIAAEPWPFRSFPQVRVEVRVTTMVARENAGFVMIGQYYIAGQE

DGLRERARPFAVQIPMRDGYDAADIAAARGLAVAELGRQIIAGGLS*

>gkv_921|gene_NONE|mce related family protein

MSDPIPDVPVKPARRSLTSRLSIVWLVPIIALIIAVGLAWQNYNDRGPLIEIVFDDASGVRANETELRYR

DVGVGIVESVGFTDDLDQVVVSVRIDKAVAPYVDTGAQFWVVRPEVTTQGVTGLDTVLSGVYLQGLWDRT

PGESENRFEGLPSAPLLATGQQGLRVLLRSSDQTLTGNSSIIYKGVEVGRVGPATVAADGQTVQAEAVIF

APHDALVTEATRFWNSSGFSVSLGPTGAALNFGSLATLISGGVTFDTFVSGAPLAQDGTAFDVFGDNSAA

RSSLFTRQDGQPVNLVAVFEGNVTGLAVGAAVELEGLRVGEVSGINGYFDTDRFGDDNLRLQAVLSIQPS

RLGLEGDSSAMETLDYLQQQVREDGLRARLVTGSLLTGGLKVQLLRDADAVAAEIDMNAQPYPEVPATQS

QITDAATTVEGTLARVNDLPIEELMQSAIGFLDNASTLVGSAQTQAIPGEVAALLGDVRNLTGAPEVQAL

PAQLGATMASITGAVEDLRAVMSDLRDADAAGRILEAVDQVQAVVTDVGTGLEGLPELLATVDELAQSWR

DLPLEGVVTQAESFLATADSVLGDPRTRQLPADISSALDSLRAVLDEARDGSLIANANSALASASRAADQ

LATATDGLPALSAQVNALVVQAGGTLSSFDENASLMRDLRLALRQVNSAATAINDLARALERRPNSILFG

R*

>gkv_922|gene_NONE|paraquat-inducible protein A family protein

MADPDTIPKTARSEGLVGCQSCGRVWRMGTERCGRCGNHLKSRDTRSLSRVWAWWLAGVMLYIPANLYPM

LETRVLFSTSSDTIIAGAVHMFHMGSAGVAFIILLASVGIPLAKFITIAWLALSVGRRGSRVNPAHRQVL

HEVVDFIGRWSMIDVFVVAITSALVQLSFAVTIVPGPAALSFALSVIFTMLSAQAFDTRLIWDSIPVDAD

KTQTPQAKGPVHE*

>gkv_923|gene_NONE|paraquat-inducible protein A family protein

VPPHYTDAELADLVACPRCDALYHARIPEKGERAVCARCHTVLIAPKRKAGMIIIMLAVTVVILVIGALV

FPFMSISASGFSNQTTLIEVALSFQSGLLVAVSLIFIAAVILLPLTRAALILYVLGPVVWDRPPLRWALP

AFRLSEELRPWAMSEIFVIGCAVALVKVTDLASVSIGPAFWMFAVLCVVTTVQDLFMSRWMVWQAMEDLS

RKAKAAGNPTTHPVETAAHG*

>gkv_924|gene_NONE|imidazoleglycerol-phosphate dehydratase (IGPD)

MRSATITRKTAETDISVTVNLDGTGIYDNATGVGFFDHMLDQLSRHALIDMTVRCTGDLHIDDHHSVEDT

GIALGQAIAQAMGDKRGIRRYGECHLPMDDAQVRAALDLSGRPYLVWNVAFPTGKIGSFDTELVREFFQA

LATHGGITLHIDQLHGFNSHHIAEAAFKAVARALRDALEVDPRKADAIPSTKGAL*

>gkv_925|gene_hisH|imidazole glycerol phosphate synthase, glutamine amidotransferase subunit|

MLTALIDYDSGNLHSAQKAFERMARETDGGDVIVTSDPDVVARADRIVLPGDGAFPACRTALQDVAGLQE

AIFEAVTTRARPFLGICVGMQMLALRGHEYQLTDGFGWIDGEVTRIAPADPTFKVPHMGWNDLVIDAPHP

ILDGIKTGDHAYFVHSWAMRVNNPAQLLAHVDYAGAITAIVGRDNIVGAQFHPEKSQATGLRLIANFLHW

AP*

>gkv_926|gene_hisA|phosphoribosylformimino-5-aminoimidazole carboxamide ribotide isomerase|

MILYPAIDLKDGQAVRLVHGEMDQATVFNDNPAAQALAFQAAGAKWLHLVDLNGAFAGAPVNGGAVDEIL

AQVNIPAQLGGGIRDLATIEAWLKKGLQRVILGTVAVENPDLVREAARAFPGHVAVGIDARNGRVATRGW

AEETDVLVTDLARSFEDAGVAAIIYTDILRDGAMKGPNVQATADLARAVSIPVIASGGVSQLSDLITLRD

TGVIAGAISGRALYDGAIDLTAALAALEE*

>gkv_927|gene_hisF|imidazole glycerol phosphate synthase subunit hisF|

MLKTRVIPCLDVADGRVVKGVNFVDLIDAGDPVDAAKAYDAAGADELCFLDINATHENRGTMYDMVTRTA

EACFMPLTVGGGVRTHHDVRALLLAGADKVSFNSAAVVNPDVVTEAALRFGSQCIVVAIDAKTVAPGRWE

IFTHGGRKSTGIDAVEFARTVAAKGAGEILLTSMDRDGTKDGYNLGLTRAVSDAVDIPVIASGGVGKLDD

FVDGVTKGGASALLAASVFHFGTFTIAEVKAHLDQAGIPVRF*

>gkv_928|gene_hisE|phosphoribosyl-ATP pyrophosphatase (PRA-PH)|

MSLQNLAATILARKSADPDSSWTAKLLSRGPEKCAQKFGEEAVEAIVEAVKGDRDALIGEAADTIYHLLV

MCAARDVTLADIEAELSRREAQSGLAEKAARPKG*

>gkv_929|gene_NONE|coA binding domain protein

MSTEKHAPEKKALEKARRIAIVGFSANPARPSHSVARFLQAAGYDLVLVNPGLAGQDHLGTKVVATLAEA

GRIDLVDVFRNSHAIPALVDELLALPYAPHTLWLQLGITNLATLAASEAGIAVVEDRCTAIEYQRHFGAS

RINP*

>gkv_930|gene_NONE|RNA 2'-O ribose methyltransferase substrate binding family protein

MKKPKWVIEKEQARRVSAQETVWLFGLHAVRDALLNPERTRLRLVVTQNAADKLADAIAASGMEVELSDP

RKFAAPIDPESVHQGAALEVKPLDWGSLADVALSLKDNGQPPRLVLLDRVTDPHNVGAILRSSEVFGALA

VVGVQRHSAPETGALAKTASGALERQPYIRVRNLGDAMDELRAMGYLLLGLDGEGEQTIEQAIEGRRDRP

VAIVMGAEGPGLREKTRELCDHLVRIPAAGGFASLNVSNAAAVALYAVSVRD*

>gkv_931|gene_NONE|hypothetical protein

MAAKYLPSYGASTGKTLRHRPDHAFCHLSLRQMRENTR*

>gkv_932|gene_NONE|conserved hypothetical protein

MPLSPKAAAAAATILWGFTYILTTTMLPHNPWFIAAVRALGGGLPLLLFARALPPAGWWGKMIVLGTLNN

GLFFGLLFVAAIRLPGGVAATFQALGPLFMVLLALPLLGVRPAGGKLIAVAAGVVGVAMVVLQGGAALDL

IGVLAALGAALSVALGGTLYSKWKPPVSVVTMAAWQMIIAGIELAIIAAILGDIPPAITATNVLGLAILA

LAITALPFVLWFTGIKGVGPAAVAPMLLLTPITAFVLDALVRGIVPSAVQTLGIAIVIGSLLYGQYVDRN

TAKG*

>gkv_933|gene_NONE|bacterial regulatory proteins, tetR family protein

MTETPALTARGLARQQALIAAATTLFLAKGYASVTVDEVVAIAGGSKTNIYRQFGGKEGLFAAVVDTLCA

ELLSPLVQLQLGDTPRAAGLMILGRTLLRQLLTPRHIAFQRMVTAASDQFPALMARWYEVGPRQSQRIIA

GFLGGGPGCAAAAVLFHDMLVTEAVSRAMMGTPMPPDAVAQHLQSAVALLLPGLAEICAPLE*

>gkv_934|gene_NONE|luciferase-like monooxygenase

MEVGVFIPIGNNGWLISETAPQYKPSFELNKAITLKAESYGFDFALSMIKLRGFGGKTEFWDYNLESFTL

MAGLAAVTSKIKLFGTAATLVMPPAIVARMATTIDSISGGRFGVNLVTGWQRPEYSQMGLWPGDDYFGDR

YGYLTEYTTVLKDLLTTGQSDFKGDFFQMEDCHMKPVPQGDVKLICAGSSDKGLAFSAQHADYSFCFGVG

VNTPKGFAPTNERLLAASAKTGRDLKSFVLTMVIAEETSEAAWAKWELYKSGVDEEAIKWLGLQSAADTK

SGSDTNVRHMSNPVSAVNINMGTLIGSYAEVAAMLDEMADVPGTGGVMLTFDDFLEGIEKFGQFVQPLMK

SRQHIMVEAAE*

>gkv_935|gene_NONE|isochorismatase family protein

MSATVDTTGGRAGRKVTLPSRPEALTINADDTAIVVVDMQNAYSTEGGYVDIAGFDISGAQSVIENIRLT

LDAARAAKVTVIYFQNGWDADYVEAGGEGSPNFHKSNALKHMRAHPETQGQLLAKGTWDYAIVDQLTPQP

GDIVVAKTRYSGFFNSTMDSTLRARGIRNLVFVGIATNVCVESSLRDAFHLEYFGVVLEDATHHLGPKVM

QEAAIYNIEKFFGWVSNTADFCGAISQIAPDQAEV*

>gkv_936|gene_NONE|endoribonuclease L-PSP family protein

MPKEIVTPAGSGKPLAPYSPGTKADGIVYVSGTLPFDKDNNVVHLGDAKAQTRHVLEIIKSVIETAGGTM

DDVTMNHIFVTDWANYAAVNEVYATYFPGDKPARYCVQAGLVKPGALVEIATVAHIGKP*

>gkv_938|gene_NONE|alpha/beta hydrolase fold family protein

VIYDVLDGPAQDAETIILSSGLGGVAGYWGPQLDALRARYRVITYDQRGCGRTGGDLPDGLTIGDMADDL

LAVLDASGTARAHIMGHALGGLIGLDLALRASDRIGKLVLINAWSRADPHSGRCFDTRLALLDHVGVEAF

LHAQPLFLYPAAWMAENAERLAAEEAHGLAHFQGAANIKRRIHALRSFDIDARLSDVTAPTLVIASRDDL

LVPWQRSARLSAGIAGSALVLMTEGGHAMNVTQPAPFNRAVLDFLAA*

>gkv_937|gene_NONE|tonB family C-terminal domain protein

MAARRPMTEEVARLTAGEVSLWAGASATVIAAVLGGIWLVPQLLPPREAMAGSLNPVSVELAQFDASPMT

ETLDVAEGELSAASEAAPEVAPELVDETAEPDPEPEVTPEELPPPEEVQPEETPPEEVQPEELPPEELPP

EELPPEEVLPEELPPEDLPPPPPEEPIDIAPEIDVPDPAVALAPVEVPPDEVLEEVPEEVAAAVPSPPRR

PNPPPPPPREEPRRAPPPASASSASAPPPTQRQAEQAVSQQAAVGVGASQSEISRWQSRLRTHIERRRGS

NNRLRERGDVGLSIRVDRAGTLQAAQVIQSSGNAELDQHALATVQRIGGMPAPPEGITDAGLQVTFVLSY

TR*

>gkv_939|gene_exbD|tonB system transport protein ExbD

MAGGIKENSEDDMDVAHEINVTPFIDVMLVLLIIFMVAAPLATVDVNVDLPVSNATPAPRPEAPLFVSLK

DDLSLLVGVNPVTAEGLAAALDEHTGGDREARVFLRADQAVAYGDVMTTMNNLRAAGYVRVALVGLESAN

SVAIPAPEAQ*

>gkv_940|gene_exbB|tonB-system energizer ExbB

MTLGKVFAMRLILALVLGLFASVTGGALLAQEAPAPDAAVPEVTVPEAPVPEAAAPEAVTPQTTAPAPAQ

AEPVQIETGLGGHDMSPMGMYNQADVVVKSVMIGLAAAAIVTWIVLVAKWLQLTGARGRARRTLRLLEQS

RSLSDASAALAGRSGAPALLVRAASDEIAASQDVLGQVDGEGVKERVASRMNRIEIAAGRRIGRGVGLLA

TIGSVGPFVGLFGTVWGIMNSFIGIAETQTTNLAVVAPGIAEALLATALGLVAAIPAVVIYNGFARAITG

YRQLLGDVSASIGRLVSRDLDRIAVSMGQGR*

>gkv_942|gene_NONE|glycosyl transferases group 1 family protein

MSMLFDEVFYRKQTRLGLRATEEHFNTAGDAKGFDPTPYFSTSYYKARYPDWQAGGAQTAIEDMVNRMRR

GEARQPHPLIDPAYYRETYPDLRSLNADAYFHFVKHGDHEQRSPSADFDAHFYANTYLLPEQPRPFLHYV

TIGKALGYLPRPQIRSFDESRAASAAKTQGLKKPILILVHSAQATGVPILARDLALYFKAEGWDPVFFLM

QAGPLLPFFEQIGPVFIGAEGWDPIGLRTGMPQMVPALITTAAAAHFGADLAAQGSPCVILIHEMAQYIH

AQNLMPSLQDAQRAGATLIGSIPRQAAGLADALGALPTIQPGITLPQTTMQSFRDAQRQFGAAPMFIGAG

TGEYRKGLDLFIDAARDITANLPDATFVWLGQLQQAGRIMVQQAQDDGIKLITPGFVSDSLAWYRAADAY

LLTSRQDPGPTTVIQAAAVGTRFVGYAADIGLIGVADSLGTFITPGDQAGFVRAALAQAQANTPALRRAT

RKMVAQHTSFKTYGAAVLQRLTSRPASSAT*

>gkv_941|gene_NONE|bacterial transferase hexapeptide (three repeats) family protein

MMKSTAEYDEVVVFGTRGNALLMLNEAELLWQGRVRVVAMVDELSNGHLHPVLGVPVLSRDERLKLYPDV

PVLIMAGGIPLRKRIFHDLVAEGATIGNISTLGMEGVDKGTIFSHGTICGPRTRIGPNTRFGIGTLIEAT

LVGHDVTVGDFAVLSAECLVLSHIDIGAEVNIAPGAVIMNGTPQRPIRIGEGAVIGVGAVVLRDVPAGAK

MIGNPAMPIRKWLKLQKMLDEM*

>gkv_943|gene_NONE|degT/DnrJ/EryC1/StrS aminotransferase family protein

MSASAIITVGAPMLPDLPRYTALLSEAIDAAWLTNGGVLHQRLEMALAADLPGRTVGLAASGTTALMMAL

QLGDLPPGAEVITPAISFAATAQVIRWCGFQPVFVDVQPDSLNICPDAVRAAITPRTAAIMPVHLLGQPC

DTDALADIARQHRLWLVYDAAHAYGVTWQGQPIGNFGDATAFSLHATKLLHTGEGGYIVTHPENGAAMRR

MRNFGLDQGRPVGFGINGKLSEAQAAMGLALLPDLPAEIAARRDLRCRYDAAFATLPDVSIQAARGGASD

NLTYYALRLPPARRAGLFNALAAQHVFARDHFPLLCGPGTAFPDAPVITAAPHPIAPARAGEVICLPFHG

RLSTADQAKIIQVTTGFIKGDL*

>gkv_944|gene_NONE|rb115

VFDDNLFIHISVNALRGNGRSWGDTQFAEGLVRAIGRIPGCGAALLFRGEVPDVTPGRDVVLRIVGPYLE

EPLPDVPNILWMITPPNLATAGMLRRYQHIFTGAQLMTDYFTSFGIPTTPLMQATEPSHFHPSKREDGAA

DLPIIFVGSYAPRAERPLVVEAVQSGFDVKIWGMGWEGIVPDRYIQGTRVNYHELAALYARARVVLNSHA

PYMAGYGIMSNRSYDALSAGAHVVSDLIPGYEVPDLPELFQARGRIEMVLHLNTLLTSPPITPAARLDMH

ARVQAGYSFDRRAEQFVQVARRLLAENNVPPCAVRRAPARALDLSSPGVGGETQSQGMLSAAREITAIAG

QIGNMSQPAPPAEVYGGVIHPLMADLRQVQRFARTLSDPVQLEQIAAGARRLQEVTDDAANPLPLRVADF

ERDAMLTRCLRNMPLWAHQPQDYVTETRKRHLVLQPRREAPAPARPIGVFLHLYYQELAPVFAKRLAQIP

LPLSLYVSTDTAEKAAQIERALPQAQVRVLPNRGRDIFPKLYGFGDAYADHDIVLHLHGKKSLHSSMLDE

WLSHILDCLLGDPADVNRILSLFDSVPRLGIVMPVVHRSVLNAAHWGFNRDIGAELAYRMGMATPLPEND

ALQFPAGSMFWARTAALQPILDLALEASHFPPEAGQVDGTLAHAVERMLGVVCRAGGYYMLPVAGSSMRY

YPRYQQKLGNNRAVAEALAQGAFDVC*

>gkv_945|gene_NONE|conserved hypothetical protein

MLTRLWNSPQILLTLASLFWAGNFVVGRAILTEAPPVAMAFWRWALAIIPVVLVARGRVDVKHELALVRR

HWLIIVVLGVLGISCFNTFVYLGLRETASINALLMQSAMPLLILLACFVLYRERPLPQQIIGVVLSLGGV

IFIAARGHLETLTSLGFNTGDLWVLAAVVAYTFYSALLRKKPAMHPLSFLVALFIVGALALLPPYLTEHA

SGQVMRLTPQMVAALAYLVIFPSFLSYLCFNRGVELLGAGRAGLFIHLLPVFGSALAVIFLGERFESFHL

IGAVLIGVGLLVAARRGRSARRKMS*

>gkv_946|gene_NONE|inosine-uridine preferring nucleoside hydrolase family protein

MKGQAMRLIIDTDTAGDDCFSMLLALHQPGVTVEAVTICGGNIDFDQQVENALYTLEVAGKGGKVPVYPG

CRRPMMRKPIDAEYVFGEDGMSDAHYPRAHQRPEASHAVQALIDIVMSNPGEITILAQAPLTNIAAAVVQ

EPRFAGAVKHLWIMGGTDNSLGNVTPAAEFNFYVDPEAAQIVMNAGFACTLSTWTLSVQDSGILAAELAD

IAALDTPLSRFFTQVNQASVDFSMARYGVGDSLHPDALTCAIMLDESLILESGDCVVDVETQGRLTRGYS

SVSSPRLPAQEVADPELGSATPANARVIRRADRAAFVQMMRRALGA*

>gkv_947|gene_NONE|ABC branched chain amino acid transporter, substrate binding protein

MKRLATLSTLSALAVVASAGAALAQDACPIRVGVLHSLSGSMAISETTLKDAMLMLVAQQNAAGGLLGCE

IETVVVDPASDWPLFAEQARDLLTVNEVDVIFGAWTSVSRKAVLPVLEELNGLMFYPVQYEGEESSRNVF

YTGAAPNQQAIPAVDYFLEELGVTSFALLGTDYVYPRTTNNILEAYLIDKGIAPENIFVNYTPFSHSDWA

TIVSDVVALGAGGGQVGVISTINGDANIGFYTELAAQGVSADDIPVVAFSVGEEELSGLDTAGLVGHLAA

WNYFMSADTPENAAFIEAWHEFIGSDTRVTNDPMEAHYIGFNMWVNAVEQVGTTDVDAVIDALPGQTFPN

LTGGIAEMLPNHHLTKPVLIGEIRADGQFDIISQTEPVPGDAWTDFLPESAILEADWVDLKCGMYNTQTQ

TCVQLTSNY*

>gkv_948|gene_urtB|urea ABC transporter, permease protein UrtB

MIRVLLIALALCFSFGAPKLVAAQTLQDALQVDPGAIAQPSRRTVGETLDRLLATEDPALPLLLERWQGR

ALYQRAADGLFFYAEQTADGFNLFDITTLEAAGTAAPREVAALIPNAGVRGVIASALIEFQLTDPDPARR

ASAVAAIARDPSADYLEPLRETLAEEDDPGLYARKERVLQMLIARFDTDIDARVAAISALGTDVSTETRA

LLNQLLRSTTAVGATVPEGVNIAAVLEPGSRALSRDAAYGMLISAGLAPAPVTPAQIREALIANIDGGFV

GGMPVAQMNTDAARTSAYDALAASGTVAPRVTPADMDAALAAHSFWSQYETSNVQITDAAAAALASATTR

VGLFQVADISLDALSLASIYFLAAIGLAITFGVMGVINMAHGEFIMMGAYTAYVVQLFIPDYTVALIVAL

PAAFLVAAIAGIALERLVIRHLANRPLETLLATFGISIALQQLTKIIFGTQARPVTAPSWLSGAWIYNDV

LGISWIRIAIFVLALVFLALLLVILNRTRLGLEIRAVTQNPGMAASMGINPDRVKMLTFGLGSGIAGIAG

VGIGLYAQVTSEMGSNYIVQSFMAVVVGGVGNVWGTLAGATLIGVMQKGIEWFNPSNTLAAQTYMILFII

IFIQFRPRGIVALKGRAAGV*

>gkv_949|gene_urtC|urea ABC transporter, permease protein UrtC

MQGGFLAKNPSVLWFLLVLAVFTVVATLLSQIGIGAVSTSMVKVLGRTLCLALVAVAMDVIWGYCGILSL

GHMAFFGIGGYAIGQWLMYARTEMIVAGTLAASPLPPTALEVKNAVASQIFGVVGTSELPWVWGFAHSLP

LQLAMVVLVPGLVALVFGWLAFRSRVTGVYLSILTQAMTLALSLWLFQNDSGLRGNNGLSGLQNLPGLDH

WGQDFIAVVFLWASALALGLGYVFFAFITSGKMGSVIRAIRDDEARVRFLGYGVEGFKLFIFTVTACVAA

IAGALYYPQAGIINPAEVAPIASIYLAVWVAIGGRGRLYGAVIGAVAVSLLSSWFTGGAAPPINLGFYTI

RWTEWWLVLLGASFVLVTLFFPKGVGGLFDLIPSRKERKE*

>gkv_950|gene_urtD|urea ABC transporter, ATP-binding protein UrtD

MNALLEVSGISVTFDGFRAINNLSISVAERELRAIIGPNGAGKTTFMDIVTGKTRPDTGRVLWGEMAVNL

VGMNEAKIARMGLGRKFQRPTVFEAQTVRDNLLMALKNPRAPFAVLLSRPTAADILRMEEIAVEVGLSAH

LHRISGELSHGQKQWLEIGMLLAQEPRLLLVDEPAAGMTPAEREHTTDLLKRMAQTRAVVVVEHDMEFIR

RLDCRVTVLHEGRVLAEGSLDHVTANQEVIDVYLGR*

>gkv_952|gene_urtE|urea ABC transporter, ATP-binding protein UrtE

MLDVENLTLKYGQSEILHGISLTARMGQVTAVMGTNGVGKTSLLKAIAGRHPFDKGTIRLDGRDMGHYSA

NGAARAGIAYVPQGREVFPMMSVTENLMTGFACLPRADHKIPDRIYELFPVLKDMAHRRGGDLSGGQQQQ

LAIARALITQPRVLLLDEPTEGIQPNIIKDIGRVIAGLRDEGKMAIVLVEQFFDFAYGLADEFVALNRGD

VVLSAPRADVTREGLLEKVSI*

>gkv_951|gene_NONE|glyoxalase/Bleomycin resistance protein/Dioxygenase superfamily protein

MKRRSFVTALIAAPFVITRPAVAQVPAVAIAFPLQTPIHVTKSGLRARDAEALSLWYQTHVGLQELSREG

ATIHLGAGGITLLEITEVEGIVLAPMRVAGLYHNAYLLPSRHDLARWVLNASNLGMRIDGAADHLVSEAI

YLTDPEGNGVEIYADRPADEWEWNNGQVEMASLQIDFQGMVAELQGQETRWFGAPPGTCLGHVHMKVGDT

ARASAFWRETMGFDAVRERMGASFLSTGGYHHTIAVNEWNSFGAGPRDPIHVGLDFVELGGSAVTAAASY

QDDWGNSIRMAV*

>gkv_953|gene_NONE|hypothetical protein

MICFALIAAAMLVALTPAVARGVAAAPCAATQVCYMPGTAPVKVALPDQCPACLPLRAFETAMPPRAAQV

AVRSADMPQPRGLTAGGQWRPPRGVI*

>gkv_954|gene_NONE|bacterial regulatory helix-turn-helix protein, lysR family protein

MTQDDPITAIKLRLQFADDAVFGPGKAALLEHIHAEGSIAAAGRLMGMSYKRAWSLVEEMNRSFTTPLVL

SARGGASGGTAQVTPTGAEVLAAYRALEKVLHSKGAAPLSKIRAHLRTGGDA*

>gkv_955|gene_modA|molybdate ABC transporter, periplasmic molybdate-binding protein

MNRKIAFAATLIGGLMASSAMAEDITVFAAASMKDSLDAVIANWTAETGNTVTVSYEGSSALARQIEQGA

PAAMFISAAIDWMDYVEGLGLIEDGTRSDLLGNSLVIVSHEAPAAPVTIDANLDLIGLLGDEKLAMALVD

SVPAGVYGKEALTNLGLWDSVAPNVAQADNVRAALALVGLGEAPYGIVYSTDAAADPNVAIYGTFPADSH

GPITYPVALIKEYSSDTAHAFLDYLKTPAASDVFTEFGFVTLQ*

>gkv_956|gene_modB|molybdate ABC transporter, permease protein

MIDWLAGFNLSAAELAALKLSVQVSLVACLCSLPLGIFIAYALARWRFPGREVLNGLVHLPLILPPVVTG

YLLLQTFGRRAPLGAFLEQTLGLTFSFRWTGAALAAAIMAFPLMVRAIRLSFEAADPKLEQAASTLGAGR

LAVFFTVTLPLALPGILTGTILSFAKAMGEFGATITFVSNIPGQTQTLPSAIYAFLQSPTGGDAAMRLVV

ISIIISLSAVLVSEILARRISRMIGQSA*

>gkv_957|gene_modC|molybdate ABC transporter, ATP-binding protein|

MIDVSLRHDFGGFKLDLDFRTGAGVTALFGRSGAGKTSIINAVAGLMHPQSGRIAIGDAVLFDSAKGIFL

PPPRRRIGYVFQEGRLFPHMNVRRNLTYGARFAPKAAGPDFDEVVALLGIDHLLDRPPSALSGGEKQRVA

IGRALLSRPHLLLMDEPLAALDEARKAELLPYLERLRRDSRVPILYVSHSVPEILRLAQDLVLVDQGRVV

AAGPLADVMSSPESVKALGPRAIGAVISARIAAHEPGEVTRMTTPAGDILVPRLQGAPGETRQIRIAAQD

VMIALERPQGLSALNILPAVIRDLQPDDAGGVLIGLQLGEVRALARVTQRSRMALGLQPGLGCHAVLKSV

AVSLG*

>gkv_958|gene_NONE|N-formylglutamate amidohydrolase family protein

MQSEMNQTPLPKGHDLHLPAHGDSPVLFAAGHSGRYYPPDFLAQVRLNPLQIRASEDAYVDQLFGAAPAL

GAKLLVARYPRAYLDLNRAAHDLDPALITDLPRRVAGLNLRVISGLGVIPRVVGPGKPIYSSKIPYAEAA

ARLSHLWQPYHAALAGQMLAAQRQYGRAILFDCHSMPSDAVGLGGPNIVLGDRHGRAADVALRNQTIAAF

RAQGFRVAVNHPFAGAYVTETYGRPADGWHAIQIEVNRALYLDEAQVVPHGGFAPLQARLTRVMAQLIGT

DHARIAAE*

>gkv_960|gene_rpmJ|ribosomal protein L36

MKVANSLRSLKQRHRDCRIVRRKGRVYVINKTQRRFKARQG*

>gkv_961|gene_NONE|bacterial pre-peptidase C-terminal domain protein

MTLKTGLKRVGLGAALAMGTALPAMAQDAICGTMGAGGTWIGGAADTSDLNTLTDPFDQMSLIMSGGRHV

SYFTVSAQTDVRLEAESSDGGDPLVEVYDEAGNFVNSDDDSGGNLSARLETSLAPGTYCMIARTFDGSGT

TAYLRLGRDDTPALTAGMNPDTWSDPDSGYTDPWATSGTCEFATAERPFGEAAIDFSTGPVTISAVPNNS

PYLGFTLAEPTALTVTAANPSADPIIAVYDGNNTWLAENDDFDGLNSRIDFMNTLPAGNYCINLRAYNDG

SLPVDVALSTYDPAAAMRGMIDRAEASPPLDGSHPVTDLGALPARHRADIQATGAAATWFSFEVTDTGVV

AIEAVANGAADPVLVLFDDFGREVAFADDSNGSLDPIMLSRVSPGTYVLALKLYSTDSRALVRMSFESFT

PAGR*

>gkv_962|gene_NONE|putative peptidoglycan binding domain protein

MRHKIYLALATMLLPGAALANDAALLLGVERYETLGRVARGADVANANDGLTALGFRVAVLPNGRAEPTL

TAVQTWLDTIPESERIVAVLSGRFVTDGSRTWFLTAEAGTPSLLGLGDTALSLESLLEVMAARQGRALLV

LAPEAQGGAIDPYLYEGIGALDIPQGVSVVTGDPSAVAGFATEDLTQPGGDLLQLANRRGGLGWQGFVPR

SGFVLMPLEAVPAGPPQPTAEQTAAEEALWQGAQALDSVEAYRNYLARYPMGIYAALAEEAIAAIVAEPN

RADRMIEEAMNLTAAQRRTIQQNLQRLGFDPRGVDGIFGAGTRAAISAWQRGNDFPPTGYVASAQLTRLE

AQAARRTAEAEAEAARQAQAAALADQDFWRETGAQGDAPGLRAYLERYPTGAYAAIATERLAAIDAEIQA

AATARETSAWAAAEAANTPAAYQDYLQVYPQGRFAPEARARITAGTAPTPDAPPTVPPADDAAASAEAAL

NINAMTGRVVEDRLNALGFNPGTVDGVFDDETRAALRRYQEARGLPVTGYLDQQVLVRLLADTLIPGVGQ

*

>gkv_963|gene_NONE|conserved hypothetical protein

MQTRNIFVIAAIAAVTAAGAQAQTSAFANQDRIDDTIEDLRDDIADDFDRDVDAFGNEGRALGFTGSLAA

RANVATGNSESTDVGVGGNFGYFDGINGHAVALSYAYSEDSDAAETNRLMLSYDYTRELSSSIYAYGKGI

YIDDKFGSYTKDTFVGAGLGYRIFNDPAIQWSIAAGPGFRWADVEDGSSIEEVAVSISSDYYYRLSETLA

FTNDTDVIWSESDTYITNDLGLTVSMTDSLALRGSIFTQYHTDPLPGYSSTDNTYGLSVVYSF*

>gkv_964|gene_NONE|short chain dehydrogenase family protein

MRDWQGRTYWLIGASEGIGRELATLVSRAGAEVILSARNAERLEELAATLPGRARVLPMDVRDSAAVKAA

AEAAGQVDGVIFMSGVMSLMRAAGGSAGWSFEAAEVMADTNFTGAVRVISAVLPQMCARGTGHIMLVASL

AAYRGLPGMAVYGASKGGVMQMAEGLRAELQDTGILVQVVNPGFVDTQMTRETGLAMPLRLSAEQAAREM

FEHMNTDTFRRGFPLAMSWSIRLARFLPDWLWMRLIR*

>gkv_965|gene_NONE|hypothetical protein

MGIGQMLKIACAGVIASVAVASAAGAQQGGDYGTYGGAYLDWLSPLAISSYEWPFKHGPISVIAPGANGA

VQTYTLVPCQDGAAVCSGSAIGAVSVDGLTTVVSGLYGRTFYLGMGGHGVIDAGGVQSVLAWDTQGNGPN

R*

>gkv_966|gene_mutL|DNA mismatch repair protein MutL family protein

VSAPPTIQQLGEDIVNRIAAGEVVERPASAVKELVENALDAGARRIDVTIADGGRTLIRVVDDGWGIAPA

QLPLALARHATSKIDGSDLLNIHTFGFRGEALASLGAVGRLSLTSRRAGEEGAQITVNGGATTPTRPAAL

SGGTVVELRDLFFATPARLKFLRSDRAEAQAVADVMRRLAMAEPFVAFTLRDASGGEDRQVLRVNAETGD

LFDALHGRLAAILGREFAENAVRVDAERDGFHMTGYAALPTYSRGAAVAQYLFVNNRPVRDKLLIGALKG

AYADLLSRDRHPAAALFVSCDPQLVDVNVHPAKSEVRFRDPALVRGLLVSAVRHALLTAGHRASTTVADA

TLAAFQPEPAFAPRAYQMPLGGGYGNPSPQALRTAWFAQSPVSSPAAPQGFAEVAQPSARIDDPVGDDNM

PLGAARAQIHENYIIAQTARGMVIVDQHAAHERLVYERLKEQMAHSGVAAQALLIPDIVELGAAGAGRLL

ALANDLSRLGLTIEPFGGGSVAVRETPAILGEVDSPALLRDILDELDDSEQTSLLGARLDAVLSRVACHG

SIRSGRRMRAEEMNALLREMEATPASGQCNHGRPTYVELRLIDIERLFGRRG*

>gkv_967|gene_NONE|insulinase (Peptidase family M16) family protein

MLRFILPLTLALFATTARAEVEIQEVTSPGGVNAWLVEQHEIPFLALEIRVRGGANLDEPGKRGAVNLMT

AVIEEGAGERDAVAFQTAREELAASFSFSVSDDSFGVSARVLTENRDEALALLREALISPRFDQDAIDRV

RAQVISGIQSQTQRPNVIASSTFNAEAFGDHPYGTALDGTIESVSALTRDDLLAAHRNVVTRDRLYVSAV

GDITAEELGTLLDNLLSDLPSNAPALPPHVEPATFGGITVVPFPGPQSTIYFGHEGITRDDPDYITAYIL

NHILGSGGFESRLMQELREKRGLTYGVGTYLVPNDLSELIVGGFSTSNQSVAEAIELVRGEWQRLATEWV

TQAELDRAKTYLTGQYPLRFDSNANIAQIMVGMQMIDLPTDYVLNRNDLVNAVTLQDLNRVASRILNPDA

LHFVVVGEPVGLDANQ*

>gkv_968|gene_NONE|insulinase (Peptidase family M16) family protein

MVHRVVAAGFALALAAMAALSPQIALAQDNVTTHQLENGLDIVVIEDHRAPVVTQMIWYRVGSADEPKGQ

GGIAHFLEHLMFKGTDTMASGAFSAAVAENGGEDNAFTSYDYTAYFQRVAADRLPLMMQMEAGRMRGLLL

TPEEIATERNVILEERNQRTDSNAGALAQEQARAALYLNHPYGLPVIGWRHEIEGLDLPEIRAFYDLYYA

PNNAILVIAGDVNPADVIALAEEYYGPIAPSDNLPPRTRPSEPPQLAARHLDFSDARVAQPYLTRTYIAP

NRISGEQGQAAALTYLAEILGGSSFTSVLGQALAFENPIALNVYAGYGGAAVDSSTFSLSLVPAPGITLA

EAEEDLDGALQRFLDRGVDESQLDRIRTQLRASEIYARDDVFHLANRYGAALASGLSVGDIQSWPEVLQS

VTADEIMQAARDVLDARRSVTLFVTPETPAPEGN*

>gkv_969|gene_NONE|putative lipoprotein

MQKTLLVMGLGLLVTASFAGCSRYNDIAPFQPGALTERELNYTANPPLSAPTSNTLPVPTYSGQ*

>gkv_970|gene_lspA|signal peptidase II|

MRLVLWSAFWVFVVDQVSKYLILHRMNLIWHGSIDIWPPYLRLRMAWNQGVNFGLFHGMDLKWLLIAVAI

VISGVVLWWMRRGDEKPLARVSAGILVGGAIGNVVDRLIYGAVADFLNMSFPGFDNPYAFNVADIAIFAG

AAGLILFSGGASRDPKQGDKQGVTGQGQSGKRPAKTVVAKDKGRK*

>gkv_971|gene_purH|bifunctional purine biosynthesis protein PurH|

MTDIQPLRRALLSVSDKTGLIDLARALEARGVELLSTGGTAAAIRAAGLPVKDVAEITGFPEMMDGRVKT

LHPMVHGGLLALRDNAEHQGAMEQHGISPIDLLVVNLYPFEATVARGADYDEVIENIDIGGPAMIRAAAK

NHAFVNVVVDVADYDALLAELEGNAGETSLAFRQRLAQTAYARTAAYDTAVSTWMAGAIGEAAPRRRSFA

GEIAQTLRYGENPHQSAAFYLDGSNRPGVATAQQLQGKELSYNNINDTDAAFELVAEFDPASGPAVAIIK

HANPCGVARGATLAEAYTKAYDCDRTSAFGGIVALNQPLDAATAEEIVKIFTEVVIAPGASDEARAIFAA

KKNLRLLVTDGLPNPQAPQLAYRQVAGGMLVQDKDVGHIDIADLQVVTKAAPSDAQLADLLFAWTVAKHV

KSNAIVYVKDQATVGIGAGQMSRVDSSRIAARKAEDMAEALGLPESPAKGAVVASDAFFPFADGLLAAAA

AGAVAVVQPGGSMRDADVITAADEAGLAMVLTGMRHFRH*

>gkv_972|gene_NONE|heparinase II/III-like family protein

VTAIPDWTAREAFLNRFYARRAGRGRKVPSFAAFPEPATMGEADRGRQILDGKFVLDGRLVEVLGASIWH

NKLYGAALQECDWLNDLAALGDQKARARAQAWVWDWIARFGRGEGWTPQSTARRLMNWLHHAEFLLQGMQ

GPAQRNFMQSLARQTIYLARAWQFVPVGQGRVSTLAGLNAALLALDGLSRLRPKAEAAFLRALSDVVGRE

GQIASRNPAEAAVLFCDIVGVAVRLHATGQAMPPALQNVIEVMAPLLRGLRHGDGTLARLQGSNGGNPVA

LDAALAQAGVRAPAGARLHMGFARISAGRLVLIADAAPAPAGGHASATAIEISVGRRLLVTSCGDGSPFG

TDWRQAGRGAASHSTLVLEGGAKPAEVMYARTPMDGGTRLELGHDGWRAAHGLVHARQIDVDIAGRVVMA

EEMLMTIDAADQARFDRALVLSGGRGVDFALRFHLHPDVTVVPQDDPLVLNLVLKSGEVWALSHDGQARM

TVEPSVYVENGVASPRSTQQVVLMGRAMSYATRMRWALGRATG*

>gkv_973|gene_NONE|methyltransferase small domain protein

MSDSLIPRRAALNLLNAVTVDHRLLSECTAIFDPLSPPDRARAQRLATSVLRAASRADRWLKPHLKKRPP

VQVQNALRLGVVEIAEGAAPHGVVNDIVTLLADGKRTANFAALANAVLRKGADLPQQWGKLALPQLPDWL

REPLREAWGNSAINAIERAHFRGAPLDLTIPRDTADWAARLGGLVLPTGSVRLDGHGMVSTLPGFAEGAW

WVQDAAAALPVRLLGDLRGQRALDICAAPGGKTMQLASAGAEVTALDLSANRLQRVAENLARTNLNATIV

QGDALEFTTTGWDVIVLDAPCSATGTIRRHPDLPFARDGAEIGSLIALQAQMIDHALRLLNPGGRLLFCT

CSLLPDEGEVQVEEALARHPGLRVIPPELPEIEADWISPEGGVRLRPDYWPSLGGMDGFYMAVLCAP*

>gkv_974|gene_NONE|conserved hypothetical protein

MADTTELPEAAKRALAEAAARRKALDEAAAAMAPELGGRDGLEPVRYGDWEKKGLAIDF*

>gkv_975|gene_NONE|RC150

MTYHALRSAGDEPRLLLTLHGTGGTEEQFHAFGQRLLPGAHITSPRGDVSESGALRYFKRLSEGRYDMDD

LARATDKLGAFIDDERAQTEATRISALGYSNGANILAALSFQRPDLIDDLILLHPLIPFTPPARDFTGRR

VLITAGKTDMICPRERTASLAQYYTQCGADLHLFWHEGGHEISAREGDAVRAFVAG*

>gkv_976|gene_NONE|glyoxalase/Bleomycin resistance protein/Dioxygenase superfamily protein

MLTQIKGLHHISSLASDANRTNAFFTRTLGLRRVKKSVNQDAPDMYHLYYGNALGDQASAMTFFPIPHAR

PGRRGTGEVGVTNFTVPKGSLSAWADRLALGGATDIAFGQSFGAALVAFTAPDGDSFALIEGDDPRAPWT

GSGVGEDMAIRGFHSASLRLKDSGATRELLHFMGYQDLESNGAVTRLHLPDGEHAAIIDIETLPTIERAR

QSAGTVHHIAFAVPDLIAHEEVRKTLEDTGWGVTPSIDRYYFQAAYFRTPGGVLFEISTNGPGFDVDEDS

AQLGQNLILPPKFEAHRDQIAAALPHMEE*

>gkv_977|gene_NONE|aldehyde dehydrogenase family protein

VINPSTEEACAVISLGDQVDTDKAVAAAKAAFPAWAVTPLDLRRTYVERILAQYYIRAEEMAQAISMEMG

APIDFARNSQAPCVSEHIEGFLSALDRMEWAHDMGPEAPGTRIVKHPIGVVGLITPWNWPMNQVTLKVIP

ALLAGCTLVLKPSEEAPLSSMLFAEFVHDAGVPAGVFNLVNGDGAGVGTQMSSHPDIAMISFTGSTRAGK

AISRTAAETLKRVTLELGGKGANVIFADAGEEAVTKGVRRMFNNAGQSCNAPSRMLVERPLYDRALEVAR

KAAEETTVAPAAKSGSHIGPVVNRAQWDKIQDLIQSGIDEGATLLAGGTGLPEGLNKGYFIRPTVFADVR

PGMRIEKEEIFGPVLSMLPFDTEEEAIQIANDTEYGLTNYVQTADKARARRLGLQLNAGMIEMNYRSLGD

GAFFGGVKSSGTAREGGIWGINEFLIEKAISDWN*

>gkv_978|gene_NONE|hypothetical protein

MVARGRRTNPLSANIESALLDHFILLNWRTIAKYPCSGRWLPFGQKLYSITR*

>gkv_979|gene_NONE|ahpC/TSA family protein

MSLRINDVIPDLSVETDQGTIKLHDWIGDAWAVIFSHPKDFTPVCTTEFGAVAQLADEWAKRGTKVLGVS

VDGVEEHVKWKADIETVGGAAPTFPIVADKGLELAKAFDMLPAEAYLPEGRTPNDTATVRAVFIIGPDKK

LKLSMIYPMNVGRNFAEVLRALNALQTSAKNGISTPANWEAGKDVVVPLTISDEDAKARFDNFQTVLPYL

RTAKLRD*

>gkv_980|gene_recF|DNA replication and repair protein recF

MAGLALNALKLSHFRSHKRAELAFDGRPVAFVGSNGAGKTNLIEAISLLSPGRGLRRAVTEDLARRPESV

GWKVQASLTRLHESHEVETAAAPGESRTVRIDDKPAPQVALAAIMPIVWLVPAMDRLWIEAAEGRRRFLD

RMVMSFAPDHAALALEYEKAMRQRNRLLKDGVRDAHWYAAIERIMAKSGAEMTRNRLALIERLRDAQASA

DTAFPAADLTLTSEGPSPVDEAALADALEGSRPRDLLAGRSLVGPHRADLSAIWQAKGMIAADCSTGEQK

ALLISLVLANGRALAEDRGVAPILLLDEVSAHLDAARRAALYDEITAMAGQTFMTGTEVQLFAGLGPRAQ

GFAVEEGPAGSTITAVDLPG*

>gkv_981|gene_dnaN|DNA polymerase III, beta subunit|

MKFSIERSALLKAVAQAQSVVERRNAIPILSNVLIAAEGAAVQFRATDLDIEVQDSAVAMVEQAGAATVN

AAMLNEIVRKLPDGALVAITYDPSAERLTIAAGRSTFTLATLPQQDFPAMASADFEASFTAPAPLLRRLF

DKSKFAISTEETRYYLNGVYFHIATGEEGPVLRAVATDGHRLARIDAPLPDGAAGMPGVIVPRKTVGELR

KLLDDDNALIAVSVSETKIRFATPNITLTSKVIDGTFPDYTRVIPTGNTRRLEVDAAEFAKAVDRVATVS

SERSRAVKLALEDDRLLLSVQSPDTGAAEEELVVAYADERLEIGFNAKYLQEIAAQVDRENAVFMLNGPG

DPALMREGGDTSAVYVVMPMRV*

>gkv_982|gene_dnaA|chromosomal replication initiator protein DnaA

MTKAGWVQVQDNLKAAVGVNNFTNWIAPLELDSLADGVATFSVPTNFIGNYVSTNFGELILYHLNRLGIQ

VQRLGFIAAAARPSLRGGLRAATPRRVGGAARLAEPAAPEAPAAPAPAKSNAIPAAQLDARYTFDRFIVG

KSNELAYAAARRVAQGGPVGFNPLFMYGGVGLGKTHLMHAIAHELRRQQPDLNVLYLSAEQFMYRFVSAL

RDKKALDFKAMLRSVDVLMIDDVQFIAGKESTQEEFFHTFNALVDAQKQVIISSDRAPGEIRDINDRISS

RLQAGLVVDLHPSDFTLRLGVLESRAKTAAAAYPHVVLEEDVLPFLAHRISSNIRVLEGALTRLFAFAGL

VNKSISIALTQDCLADILRASERKVTVEDIQRRVCEHYNIRIADMVGPRRVRAFARPRQVAMYLSKKMTS

RSLPEIGRHFGGRDHTTIMHGVKKIEQLISSDSQMADDLKVLKRALEA*

>gkv_983|gene_rpsT|ribosomal protein S20

MANTPQSAKRARQNAARFAVNKMRRSRIRTYLRKVEEALASGDQAVAAEALKNAQPELMRGVTKGVFHKN

TASRKVSRLAARVKALATA*

>gkv_984|gene_mutM|formamidopyrimidine-DNA glycosylase|

MPELPEVETVRRGLLPVMEGQVIAAADVRRPDLRWPFPPDMAQRLTGKRVLSLRRRSKYILADLDSAETL

LIHLGMSGRMLISGVMIGEYEHARALPEKHDHVVLDMAGGARIAFNDARRFGAMDLMATDAVDQNPLIAV

LGPEPFGNDFSPAYLAARLAGRKTPIKSALLDQHIVAGLGNIYVCEVLFRARIHPARLAGDLTALEADRL

VPLIRDVLDEAIRAGGSSLRDYQKTDGTLGYFQHAFQVYGREGQPCSTPGCIGTVARIVQAGRSSFFCPL

CQAIHR*

>gkv_985|gene_NONE|ubiquinone/menaquinone biosynthesis methyltransferases family protein

MATDDQKTTHFGFQNVPEGEKSGRVQGVFRSVASKYDIMNDVMSVGIHRLWKDAMMDWLAPRAGQRLLDV

AGGTGDISFRFLRRAGGAHATVLDLTEPMLIAGRQRAEAEDMAAQLDWVVGDAMALPFPDNSFDVYTISF

GIRNVTRPEVALAEAYRVLRPGGRLMVLEFSQLPNPALQKAYDLYSFNVIPRMGQIIANDRDSYQYLVES

IRKFPDQDTFLGMIRAAGFENAAYRNLSLGIAALHSGWKI*

>gkv_987|gene_ubiB|2-polyprenylphenol 6-hydroxylase|

LRAVLHIWRLIRTGATFERTGAMSQVLHAVDAPRPLRIALHTLAFPFRWLGYRGDPALPPTTRALTALGP

AYIKFGQIISTRSDVIGFDLAAELRILQDSLPPFALDEARVEIARELGAPVDTLFSEISPAVAAASIAQV

HRAVVRETGETVAIKVLRPGIDKAFRRDIDAFRFCAKLIETLAPKARRLRPSDVVDHFESVVQDELDLRI

EGASASEFRAKTHDDPMFHVPKVHWDLSARRVLTMEWVEGIQAADLQAMDAAGIDRAALAQRLLQQFLRH

ALRDGLFHGDMHQGNLRIGPNGQIIALDFGIMGQLDTYTRRVYAEILYGFVQRDYRRVAEAHFEAGYVPA

DRDIDAFARALRAIGEPIFGMDASRMSMAKLLSQLFDVTERFGMQTRTELILLQRTMVVVEGVTRHIDPR

SNIWNTSGPVIEDYVRDNIGPRAFLADLSEAAKVLARLGPRLPDLAERRVIAFSTPPQTQAPKSRPLLWA

LTGAVIALVGFTLGALLH*

>gkv_986|gene_NONE|flagellar hook capping family protein

MVTTTGVTPTTTANTATTSTQAALSSDFETFLKMLVAQVQNQDPLNPIDSNEYAAQLATFSGVEQQVQTN

QLLNQLIAQTGIGNLGAMSGWIGKDVRVAAAASYEGAPVTLSPNPAALADRAEVVVKSSDGTEVHRFAIP

VSAESVSWDGTTADGGTALEGLYTFEVASYVGDTLILQEQAEVYTTVREVQSASGAQYLIVSGGVAVSPS

AVTALRG*

>gkv_988|gene_NONE|flagellar hook-length control family protein

MGAGMELNIATLLTGTEARSLPAVQVKTLLDEIETAETTFADLFVVIPTPLPAPAPLPPALGAEDLPAEA

PVPEDPAIAPDAAMPSTLSLSAPMEASVTEKSDTEKPENPERPETLAAPAPLKAPLVPTPLAQAVLLLQV

APAPVAADAMTQSLAPVVPAPAPAPPRSRGTLPELAPLAAPLNLPRADGDAPSLVAAAPITTSDDSLMPI

LSTALSTAPSDLDIVAPPIPEARAEVVVEQTRLAQTTRDSAVQTSAPPASPTAAQAVDVARQIEAAISRS

RDGALEIALSPEELGRLRLTLSEGQNGYSLQVVTDRPDTLDLLRRHIDVLAAELRDLGYGTLDLSFQQQG

QGDAHTPYGGQGAENADAAPQIVTLRPRGDGQLDMRM*

>gkv_989|gene_NONE|conserved hypothetical protein

MHIPPLPPTAPQTRLRDAAEALETSFLAEMLKSAGLGRAQAAMGGTGSDEFSTFLLEAQAREMVKAGGIG

LAESLYQALAARGDHDAP*

>gkv_990|gene_NONE|hypothetical protein

MPRNPPAQRILALFESERRGIMAADFTALDVLRTEKEALLAQLEHGMPQLSAADLARISQAAQRNQGLIA

AALRGLRDAHQPKPRPSFSTYSASGDRAAYPAAPPAFERKA*

>gkv_991|gene_NONE|flagellin fljK (25 kDa flagellin)

MSSILTNNGAMVALQTLKATNSALTNVQAEISTGKSVANSKDNAAVWAISKVMESDVQGFTAIQDTLSLG

ESTVSVARQAAESITERLNAIKDNIVNAQTANVDRAKLQTAIDADIEQIRNIVSTAQFSGMNLIDGSTTT

SSFLASLDRRADGTVGVSTIDVAGQNLSTGDYVATAVFGAATDGVTANSDAAAFNLDSGGGTGTIEIDAA

ATYAAGDKISLNIGGKTATYTVSEADVAATTPADLIAVGLKNAVDALGIADLSVDYDSGTPGTLAFTNDG

GNDLTVSAQFKNAGAGGLSLLNTIDVTSDAGALAALNNIDSIVQTAIDASSAFGAVESRISTQADFVGKL

NDSLKSGIGAMVDADMEEASARLQALQVQQQLGIQALSIANQSPQSLLSLFQ*

>gkv_992|gene_NONE|protein flaF

MNAIEMARSFYGNALLGLRDTRSTEQAIFSRITAALIAAEGANFPTKVKALHDNRRLWTWLAADVASAQN

QLPADLRARLFYLAEFTLDHSSKVLREGADLAPLIDINRAMLLGLAPRQKEPA*

>gkv_993|gene_NONE|flagellar protein FlbT family protein

MAGLVLKLAPHERVLINGAVIENGDKRSRLSVVSPKANILRLRDAIAPDQATTPVRRVCYIAQLLLTGDC

DRAEGRRQLLHGIEQLSQALVDPDSRRQLADATAASITEDYYRALRRLRALLPREERLLTPAAEA*

>gkv_994|gene_NONE|conserved hypothetical protein

MSFQPVLPLSGYTGWRFLSQTLERQTAVFAASGEVAMNSTYFRDTIAKIQTPEDLLADRRLLSVALGAFG

LQDDLNAKAFIKRVLVEGTDDKTAFANKLSDKRYAALANAFGFGNEGGARTADAGFADTILARYTAESFE

LAVGEQDNTMRLALNLSGGISDVMQHATSDSAQWFAMMGDSPLRSVFETALGFPTGFGQIDIDQQKQQFM

ARAKATFGVESFADFTDPAVQEKLIRLFMLRSEMNNSSLNSPASVALKLLGG*

>gkv_995|gene_NONE|flagellum-specific ATP synthase

MRAKKPDHDQLVMMNRLTEAMADGPQSKPVGRILRATGGTLGIAGLAAFSRLGDRITVLAHGGPLQAEVI

RVGVEEVTAVPEGVADGVAVDDRVILQGAPRFCPDDSWIGRVIDPDGAALDGRPLLHGLVPRDLRQSPPP

AAARRAMGARLRTGYMVFDTLLPIVQGQRLGLFAGSGVGKSTLLGGLARSIAADVIVIGLVGERGREVRH

FIEEVLGPQGMARAVVVAATSDAAPQQRRRCAWAATTVAEYFRDQGRQVLLMLDSVTRFAEAHRELASAA

GEAAALRGYPASTGPTIAALCERAGPGMGQQGDITAIYSVLVQGSDMEEPVADMLRGTLDGHVILDRAIA

ESGRFPAVDVLRSVSRSLPAAASLPENALIAEARSHMGQYDRAALMIRAGLYVGGSDPIVDAAIAARPGL

ENLLAQSNLPSNAAAFAALRQVLTPLRVVGRG*

>gkv_996|gene_NONE|flagellar basal body rod protein FlgB

MHQNLALFGTAADMAAHASQRINQTAINIANADTPGYRAQTIGGFAEAYAGALPGSALRQTRAHHLAGDG

LAPTNIQRAAVEESPNGNSVSLELETLAAIDAQREHNQALSIYRHGLTMLRTAIGG*

>gkv_997|gene_flgC|flagellar basal-body rod protein FlgC

MADFSTTLSIAASGMQAQASRMRFTSENIANADTPGYHRKLVSFRAAHANDGTVEASRVRLDRAPLAEIY

DPAHPLANDQGNYSGSNVDLLIEIADAREASRSYEANLKVFDQVRQMSTSLLDLLRR*

>gkv_998|gene_NONE|flagellar hook-basal body protein FliE

MDIRSTLASRAYDAVRAQPQVSDSAGSYFSSAAAEFAATLQQGEAAAMGAMTGDYDPHALVTALAQSELA

IETAVTIRNKVVEAYQEILRMAV*

>gkv_999|gene_NONE|flagellar biosynthetic protein fliQ

MLSEAQIYDTLRAGLWAAFLMSTPILVVALVAGLIVGLFQALTSVQEMTLTFVPKLAAIVVVFWLTMGFM

TRTLTAYFHGVLVPLIAGG*

>gkv_1000|gene_NONE|conserved hypothetical protein

MDNTGYASLTRQTGLLHEMRAIANNIANANTTGYRAEGVAFSEYVVRLGPQSQSLSLATARVRQTDFSQG

DIGATGASFDLAIEGEGFFMIDHPEGPMLTRAGAFTLGPDGVLLSADGYPLLDAGEAPVMIPAEAGPVLI

GADGTISAGGDVIGQIGLFTPTDPLSVTRTSGTGFFSDDNMPYLEGQIRQGHLEGSNVNAIAQIARMIEV

QRAYEFGQSLADREDERIRSTVQLMSR*

>gkv_1001|gene_flgG|flagellar basal-body rod protein FlgG

MRILDIAATGMLAQQTRVEVISNNLANMNTTAYNPRRAEFADLHYQQVQRAGTVAATDGTILPTGIQLGM

GVRTTAVSPMLGQGAVQETGGSLDVAIEGQGYFEVALPDGRIGYTRDGALKLTGDGLIVTAGGLEVAPGI

VIPEDAQSVAINADGEVYASFTNAVEQEFLGQLNLAMFVNPRGLEAMGSNLYLETAASGQPMVAVPGEDG

LGTLRQGYLEQSTVDAVREVTDLIAAQRGYELNSKVISAADQMLAAMVQIR*

>gkv_1002|gene_flgA|flagella basal body P-ring formation protein FlgA

LSLIAGTAQADYVVAQRNLPANGIITADLLGLRPGEVPGAITDPSEIIGLEARTPIYAGRPLQASQFGQP

AIVQRNQVIAMQFTRGALTIMAEGRALERAGPGDLIRVMNLQSRTTIMARIGVEGTAYVTP*

>gkv_1003|gene_NONE|flagellar L-ring family protein

MLRPSLLLACAMTLSACGGGTPLVGQPQLTPSLMSDEHLAMVSSDMPMSLGPLNTGASLWSGTRNSLLGD

RRAMQRGDILTVVISINDRAEFSNSSGQSRTASQDMSVGALLGLPERAAEVLHGGATLNPGIQLDSSSNF

NGTGSVSRNERLTLRVAATVIGTLPNGALEIIGSQEIRVNNEIRELTVTGFVRTADINRQNEVSYDRIAS

ARISYGGRGPISVAQSPRYGQQIIDRIAPF*

>gkv_1005|gene_NONE|hypothetical protein

MKKILLPMILTVIGTASGIGAGIITARPAVEAVPTIIPPTHTYVPLDEPFVVPILRDEMTRAIVVAALGI

EIDAAQASIVAPAMPKLRDQFLQVLYDHANTGGFDGLFTAPDVMRRLRDRLKSAAVETLGNGVHDILVTD

LSRQEY*

>gkv_1004|gene_NONE|hypothetical protein

MAANDHHSLAALGQVTALMRAAAEGRLAALRARAQQTEAQRRALDSAAKQVFDPAASADSGAVEQASLHW

QRWVEQRRRALLSEESKLHAEIQNQLPHLAIAVGRDETVARLRADAVARQRREKD*

>gkv_1006|gene_NONE|flhB HrpN YscU SpaS family protein

MSEEAAGADKSHDPTPKKLEDARKKGDHPKSADLTIAAAYGGYYLGLTALGAGLALAAVNQLGGLLYHAD

GLATDAFSAPAHVVLRPLLSITSLAVLPLITVPAMLALLSLFAQRALVFAPSKLVPKLSRISPLAVAKQK

FGLAGIIEFLKSAAKLLLYTGALAWFLSTNFDEIAATPAMPARSVLVFMGQMLAAMLLIALVISLVLGVL

DAVWQQIRHLRDNRMSRQEVMDEMKESEGDPHTRQQRRQRGIAIAMNQVAAEVPKASVIIVNPTHYAVAL

QWQPDSRRAPVCLAKGIDEVALRMRQIAQEHGVPIQSDPLTARALYAQTEVGQEISRQHYAAVAAAIRFA

DDMRRRAARG*

>gkv_1007|gene_NONE|bacterial export proteins, family 1 family protein

MSALHPLAALLEPVGFAAFLVFLRAGAFCALLPAFGEQTLPLRLRLSVAIAMTMITLPAVLPLMPPPPVQ

ISQMGALLLTEIGAGLIFGLSLRLLVWALETAGAIAAQSVGVAQIFGPGMTAEPSSAFSQIFIMAGFALA

CAMGLPVQAVAMMIGSYNVISPGIFPAPDMLAQAILPGFSESFSLAVRLAAPFFIAGLLYNLTLGFVNRA

MPQMMVTFVGAPALTGIGIVLLLLASPVLLGIWVRALGAAFGIPFGVAP*

>gkv_1008|gene_flhA|flagellar biosynthesis protein FlhA

MIMDMRNLFSPTVALALALLTVIIMMVLPMPSWVLDIGLAVSFSLAILIFTVTLFVERPLDFSSFPTVLL

ASLLLRLSLSVSSTKLIIGQGHTGTDAAGHVIEGFANFVMGGSIMLGLVIFCVLLIVNFMVINKGAARMA

EVGARFALDAMPGKQLAIDADMASGAIDHAEARARREREQAETTFFGSLDGASKFVKGDAVAGLLITALT

LVAGLASGVFVQGMAIGQAFETYAILTVGDGLVSQIPAVIISVASALLLARGGANGAVDLALIGQLGRQP

APLFTVAALMLVLALVPGLPTLPFLGAALALGYGGYLSSRALKSRQKAKSQPQIQPAAPPRTMGDVMDID

DIHVEFAPDLVEMVLDPATGLDARIATMRNHVATAFGLLLPEIRLTDNPALPVGSYVVLVQGVEQARAVL

RPGRMLALLPSPEAAAPSGEDVREPVYGAPARWIAPDVQADAALLGMTTVQPAEVLATHLLEVIKRNFAR

LMTHKALRKRLDEMVNLSDTPRADANRKFIDEMVPDRVPVDLLLAVLRLLLDERVSIRNLPLIIESVAEA

RALAANPEAVAEHVRQRLGFQLVAELRRADGTIPLVQLAPEWEEIFTTYQIGADRSVQDVALPPEEFNRL

ATAIADRIGAASDQNSYPALVTSGRRRRFLRTVLQAKGIPNPVLSFEEIGVDARPALVGVVAA*

>gkv_1009|gene_NONE|transglycosylase SLT domain protein

MWRLVFCAALLWSGAALADQCTDAANRAAQRHGVPPPVLIAVSKAETGRHRDGRLEPWAWTINHAGRGYW

LDSRADALTQARNLLAQGETNFDSGCFQINWRWHGQAFSDVSQLFDPDQSADYAARFIRRLYDELGNWSV

AAGAYHSRTPALAAHYRQRFDQILTAMNDPAAGTATRALRVNTFPLLQSSGAPRGLGSLMPGRS*

>gkv_1010|gene_NONE|hypothetical protein

MRAIPFIILALLGPLPVIAQELVPQDITVRAGRHPSFTRLTFPLPADATWQLGRDGASSYLLRINGVAVS

FDTQQVYQRIGTERIAALAPDGSDFGLTLGCACYADAFIAAPNLLAVDIRDGVPPPTARFEAALDPILPL

PDIARPLPLVFLPSTAAAPVVDLGPFERALQESFARAAAQGLVDATVPVMRPAPALPDPIEAPLVAAPSI

RAQPGIAVQTSIDRDSGPPARDRLSRSGAECLPDAAFSLAAWGAGVTLQDAVSTGFSTLYTANEAVTRDG

VATLAQSYLHFGFGREASAIAAVYPLADPGLARLAALGRIIDEVPVATPAFGDQHGCLGAVALWAALEAG

QLPPLRPEDRIAMTTALRDLPEPLRGQMGLRLARLTLDAGDIEGAQAIMDIGSTARAYGAEGVTSLIADS

MAGASAAVSVLQQAQQSGAVVDPASLIALTDAVLQRGEAPPTALFDMVSSARYSLRGTAFAADLARAQMQ

LLLADGQVAQAKAFLAQPDLGLSPPQAATMGDLLLLAQTDRLSDFAFLGEVLALDPNTIGAIAGNSAAKR

LINLGFGEQALALLTPPATGEAMAERRYLRAEAAQMTGRTAVGLAALTGLTDPRAEVLRQALQDPAHAAD

NATDQAWRRGDWAALANTGDAVLQAVATEITQGPTPADSIATLAQAQAVITQSQAARDMASGLLDRFALP

R*

>gkv_1011|gene_NONE|lateral flagellar motor protein MotA

MFGLVGIVVVFVMVFGGYLLSGGKMGIVIAALPFEMMIIFGAAVGAFLLANSIGIVKATLKALPLVFRGA

RWKANDYRDLLVLLFELIRLARANPVALEEHIEQPRASSIFAQYPRILADHEAVSLICDTLRAAGMNYDD

PMQVEEVLEKRLEASRHHALQPVHALQTMADGLPALGIVAAVLGVIKTMSSIDEPPAVLGKMIGGALVGT

FLGVFLAYGFVGPMAARLQSICDEDAHFYQLIREVLVANLYQHAPPLCIEVGRQSTPENLRPGFADLEMV

LKASKEAA*

>gkv_1012|gene_NONE|hypothetical protein

MRTLQVVVGLLAVSGLIRFGLVAGSATAAEGLPPSLDLPASAEEGCGASAAVLAALQSREGQVAAREGAV

ADRLRALDVIEQRVTAQLAALAQAEADLAATMALADQAAGNDLATLAAVYQEMKPEEAAAIFTAMDPVFA

AGFLAMMRPQTSAAILARLEPAQAYGITAIIAGRNTGAPTL*

>gkv_1013|gene_NONE|conserved hypothetical protein

MIADVLMISAALAAAIYCHVLSRRLRAFTNLERGVGGAVATLARQVDELQRALNAARTAGDQSANRLDSA

NRKAEDTTRRIELLLAAMHDLPDSAPAPKPAKPRRLRKTPAPAADLPSFLRADNEVPR*

>gkv_1014|gene_NONE|flagellar basal body-associated protein FliL family protein

MLSMIVAGVLGLAGAAGGYLAVAQGLVRLPGGDVPVEAAFSAPEPVSFIAMEPLIVNLPTASAGRYLRFT

AQLEVPAASAPEVERVMPRITDVLNGYLRAVTPADLADASGLMRMRGMMLRRVQAVAGEGNVRNILVMEL

VVN*

>gkv_1015|gene_fliF|flagellar M-ring protein FliF

VQNLLALWSGFSLRRRIILVAATLGIFLAVIGLARGVGRPDMALLYAGLDPAASAEVVAAVEQQRLPFEV

RGDAIYVPAASRDLLRMTLAGAGLPAPGAQGYELLDNLSGFGTTAQMFDAAYWRAKEGELARTILAVPGL

QAARVHISPPAARVFQQGEPATAAVTVTMASGSASQQQAQAFRSLIAAAVPGLDPGNVAVIDAKAGLIAA

PDNAQLADTRQDELRNRVRSLLEARVGAGNAVVELAVETVTDTESIFERRLDPEGRVVVSNDVSETARRS

QGNADGSVTVASNLPDGAANQEAGSSSSEDRESRNVTNYELSETSREVLRAPGAVRRLTVAVLVNEPTGP

EAAPRAPDELEALRELVASAVGFDAARGDVITLRAMPFVAPPVLGTEAVAAPARLFDPMQLVQFGALALV

ALILGLFVLRPLLRPKPQVDSLPPLLDDSFAMAMPMADGFAALDAPDPVARLTALIADRQEDSVRLLQSW

VEDGRKPDHG*

>gkv_1016|gene_NONE|hypothetical protein

MMMRRPVDLTSFDPAPAVPAGPSADYLAGYADAMAECEQREAARTAHALTELAAHFSALAFTHSDARRTV

LASLTPFFDALCQQLLPGTRGTSLRLYLAEALDQAAAQDLPAHITIDLAPEDHALMTAHAAQLPSYCSLQ

LRTELPPGMVLIGTKAGETCLDVQAIHTCLQDLMQIAAHIIAKPQPEENHVRHSA*

>gkv_1017|gene_NONE|surface presentation of antigens (SPOA) family protein

MSDTLPDAKPLLEGANPALWNVPIEVTIAVGRSRPSVRELLALEENAVLQLDRRIDDPVEIYIGDRLIAR

GELQESEDEPGQLAVRLTEVATTPGEHL*

>gkv_1018|gene_fliP|flagellar biosynthetic protein FliP

MRAIALFSVALTLLAGAAGAQGVDIQSLGLDFGEGSVAGRSVQLLVMVTLISLVPALAIMFTSFPFMVTV

LSILRQGLGTQQSPPNMLIVTLALFLTWYVMAPVFDEAWANGIAPLLEDRLPVDAALRAAMAPFQTFMSG

RVDPTTYQTMASLRPDTVGVAVTDAPLSTLVPSFLLSELSRAFQIGFLIFLPFLIIDLVVAAVLMSMGMM

MVPPAVVALPFKLAFFVVADGWSLLASSLVRNYF*

>gkv_1019|gene_NONE|flagellar P-ring protein (Basal body P-ring protein)

MRAILLAACAAMALVTVPLQAAPTRLKDLVEFDGVRGNDLVGYGLVVGLNGTGDGIRNAPFTEEIMANVL

ERLGVNITGEQFRPRNVAAVMVTARLPPFARAGSPMDVTVSAIGDASSLLGGTLVMTPLNAADGEIYAVA

QGTIIAGGFSAEAQAARVTQGVPTAGVIPAGATIEREVDFDFRTLSNVRLALRTPDFTTAARIETAVNQT

FGRAVAVMQDAGTVIVSIAQTQASSPAHAMSQIENILVEPEIRARVVVDQRSGTIVIGEDVRISRVAVSQ

GRLTVRVEESPMVVQPNPFSNGETVVVPQSTAALSVDPGTGGMAEVNTGTSLSELVAGMNALGVAPHDMI

DILKSIRAAGALHAEFIVY*

>gkv_1020|gene_NONE|hypothetical protein

MAFTPLSDLGRHFANQQANVQMRERLAILTAEVASGEAHDLPAQLGTRANQFAAHDYRLGMIASYSKAGS

LMATQLAAAQSALENIDLQRSTISTQLIAISPQATQVELSAGAEAARNGFAQLISALNTQIGGVTLFAGT

ASGAPATASADVMLSAIETYIGGAVTVSDVQNGIAEWFADIFPTDPAEGYQGNTQNMGRQIDKDLSVALD

VRADDPVVMQLLQDLATAAIADRLGFSGVAQAQLLNKAGEGLITQASGLTEVRARVGLAEQTLAQSQTRL

AAEKTSLGTARNDLAQVDTFEAATALQAVSDQLELHYTLTARLAALTLTNYLR*

>gkv_1021|gene_flgK|flagellar hook-associated protein FlgK

MSLTYSLGNALSGLAAVSRQTEVISSNISNALTEGYARREVALSASSIGGRGAGVTVDGVTRAVNAAITA

DRRAAQTHMSGALALSDGLARLEVAMGSAADAGSIAGRIGALEQALVAAAAEPSSDAGLQAAQQALQAVV

STLNASAAKVQQMRVDADADIATQIKALNTSLTQIAALNTQISRALTTGADAAGLMDQRAILIDQVASLV

PLTEVPRDNGAVALMAPGGVMLVDNSAATFSFTHSHTITADMTLENGLLSGVLVDGRPLDASGGMGKLAG

GTLGAAFALRDTVLTAAQSGLDEIAADLALKFQDSANDPSIAGGLGLFTDSGGLVDPSDATGLSARLRLN

TLIDPAQGGSAQLLRDGLYGGGTAVGSAVQLNRWSSALGQSSALAAGVLNDIAAARVSSDREASYQSALF

NSLYSAEAAQGVDTDREMQKLLLIETSYAANAKVLQAVDGMLQTLLEI*

>gkv_1022|gene_NONE|conserved hypothetical protein

MSISSSMNAGVAGLSANANKLATISDNIANSSTNGYKRAQTEFYAMVIGSSSTKYTAGGVRTTATRVIDE

RGPLVSTSNATDIAISGRGFLPVTTLSAIEAGGALPFQMTTTGSFSPNAAGYLTTASGHVLMGWAADSTG

SIGTQSRDTTSGLTPVRVLTNQVVGSPTTQLTLAANLPATSTEAGATGTVETQVIEYYDNLGKTKTLSVN

YIPTVPATGESNTWTVEIYDDAQGGALVGTYDLVFDDSRTGGGRLLSVTTIAGGPYDPATGLMSIDVASG

PLTINLGVPGQADGMTQLSDGFAPVAVTRDGAPSGTLTGVEVDASGKLYGTYSNGLTRLLYQIPVVDVPN

VNGLQALGSQTYAVSQDSGAFFLWDAGAGPTGEMLGYASEGSAVDVATELTQMIQTQRAYSSNAKVIQTV

DEMLQETTNLKR*

>gkv_1023|gene_NONE|ompA family protein

LHVSLTLQFGGVGMAQTGNQRIIIKRKKVVAGGGHHGGAWKVAYADFVTAMMAFFLLMWIINSITEEQRD

GLANFFATGTPVSTVSGGADGMFGGRDSQSQQVGPAVGSGMDYITGRMPTARGASAAQSSDILALEDIAR

VLSGQSGESLVDDAIQRHIITRMTDEGLVIEVFSTPDAPVFQPGGTVLTPLSLAIIQMIASVAAQVENGI

AVAAHLPQQAGTDQGAWALTAARADTIRAALEGAGVAPVRLRRVTGHADRSPITLDPTVQRNDRVEITVL

RNGR*

>gkv_1024|gene_NONE|glutamate synthase [NADPH] large chain (Glutamate synthase subunit alpha) (NADPH-GOGAT) (GLTS alpha chain)

MSSYDATWVAAEEAKRKWMSENSLYREEDEHSSCGVGLVVAIDGTPSRSVVEKGITALKAIWHRGAVDAD

GKTGDGAGIHIQIPAPFFHDQVRSTGHEPATDKLMAVGQVFLPRTDFGAQERCRTIVEAEVLRMGHYIYG

WRHVPVDTSVLGEKANATRPEIEQILIRCEKNIDAEQFERELYIIRRRIEKAAAAGQVNGLYICSLSCRS

IIYKGMMLAEQVAVFYPDLMDDRFESAFAIYHQRYSTNTFPQWSLAQPFRMLAHNGEINTLKGNINWMKS

HEIRMAHGAFGDMAEDIKPIIPAGTSDSGALDAVFEVMVRAGRNAPMAKTMLVPEAWSKNVGEMPKAWAD

MYAYCNTVMEPWDGPAALAMTDGRWVCGGLDRNGLRPMRYVVTGEGMLIAGSEVGMVPVNERTVVEKGAL

GPGQMIAVDMQTGKLYHDREIKDKLAAAQPFGDWVEKSVSFSSTVVTLPEKRLFSGAALRKRQIAAGYSV

EELEQVLTPMAEDGKEMIASMGDDTPVAVLSKQYRPLSHYFRQNFSQVTNPPIDSLRESRVMSLKTRFGN

LKNVLDESSNQTETLVMDTPFICTGEFEELLRQFDGAGVVTIDCTFDAGSTQDALRGGLERIRAEAEDAV

RSGASHIVLTDEFQSETRVAMPMILATSAVHSWLTRKGLRTFCSLNVRAAECIDPHYFAVLISSGATTVN

AYLAEDSIGDRIERGLLDGTLEDAMRRYREAVNAGLLKIMAKMGISVISSYRGGLNFEAVGLSRSMVDEY

FPGMQSRISGIGLIGIQHKAEQVHALGWKGGQDVLPIGGFYKSRRSGEKHAWEAQTMHMLQAACDRASYE

LWQRYSQAMQANPPIHLRDLLAIKPLGGAVPIEEVESITSIRKRFVTPGMSLGALSPEAHMTLNIAMNRI

GAKSDSGEGGEDPAHSHPLPNGDNPCAKIKQVASGRFGVTAEYLNACEELEIKVAQGAKPGEGGQLPGMK

VTELIARLRHSTKGVTLISPPPHHDIYSIEDLAQLIYDLKQINPRCKVTVKLVSSSGVGTIAAGVAKAKA

DVILVSGHNGGTGASPGTSIKHAGLPWEMGLTEAHQVLTMNNLRDRVTLRTDGGLRTGRDIVIAAMLGAE

EYGIGTAALIAMGCIMVRQCQSNTCPVGVCTQNPELRQKFTGSADKVVNLITFYATEVREILASIGARSL

DEVIGRADLLSQISRGSARLDDLDLNPLLIQVDGSKRHTYDRSRPRNAVPDTLDAEIVKDAQRFLNDGEK

MQLDYAVQNTLRTIGTRVSSHIVTKFGMRNSLQDDHLTLKLTGSAGQSLGAFAVHGLKIEVSGEANDYVG

KGLSGGTIIVRPPMSSPLVAAENTIIGNTVLYGATDGYLFAAGRAGERFAVRNSGAKVVIEGCGTNGCEY

MTGGTAVILGTIGANFGAGMTGGMAYLYDPEGRASAMINMETLVTCPITVTHWEAELRAMIERHAHETGS

RKAIEILANWDAEVPNFLQVCPTEMLASLKHPLTTEVVAVPAE*

>gkv_1025|gene_NONE|hypothetical protein

MTHFRLFSLVPAFAAVATLLPLAAGAQQQRNPNTTFAVPAGCEARLTVQGRACVVSHHFTCSADPEGYQR

RIDINEEGPVYVGMIDAETQWIESRHLRSGLTEQLLPNPADPASLSDLIATGRDDYDFSTTSSAPGQQPF

VMNYRGYDRLTGETLTVDGETLDQTEFSIRAFDAAGQLVWQSEGNEYISREWRHFLSGSSTISTSEDTFT

DDNSPVRISRPGEAGFLSAVPAFGCGDLMSSLPQPPSLKESRHEQL*

>gkv_1026|gene_NONE|glutamate synthase [NADPH] small chain (Glutamatesynthase subunit beta) (NADPH-GOGAT) (GLTS beta chain)

MATEKMLKFVTVPRLMPEKRDADARTQDFDEIYREFAANKAAEQASRCSQCGVPYCQAHCPLSNNIPDWL

RLTAEGRLQEAYEISQATNTFPEICGRICPQDRLCEGNCVIEQSGHGTVTIGSVEKYITDTAWENGWVKA

HLPEIERSESVGIIGAGPAGLAAADVLRRAGVQVTVYDRYDRAGGLMTYGIPGFKLEKDVVMRRINQLEQ

AGVQFVLNCNVGDDISFDALRGKHDAVLIATGVYKTRDLAGDNADAAGIVRALDYLTASNRKNFGDDVAE

YDNGTLNAEGKRVVVIGGGDTAMDCVRTAIRQGATSVKCLYRRDKANMPGSQRETKNAEEEGVEFVWLAA

PAGFTADAGTVTGVNVQRMRLGAPDASGRRSPELIEGADYIEDADLVVKALGFEPEDLPKLWGVDGLEVT

RWGTVRAQFGTGQTNLDGVFAAGDIVRGASLVVWGIRDGRDCATAILDYLGQAASVAAE*

>gkv_1027|gene_uppP|undecaprenyl-diphosphatase UppP|

MVDNNLFLSAFLGVIEGLTEFLPVSSTGHLLIAGHFLGFESPARVFEVAIQLGAILALVVYYFARLYGVA

RRIPHDPLARRFVLSVLIAFLPAVFIGVLAHDLIKRVLFESTATIAVALIVGGVILLVVDRMKITPRYRD

PMDLPLPMALKIGLVQCIAMIPGVSRSGATIVGSLLMGVEKRAAAEFSFFLSIPTMFGAVAYDLFKNRDA

LSGAAWVDIAVGFVATFIVALIVVRWLLDYISRHGYALFGWWRITLGTVTLIALALGFGAN*

>gkv_1028|gene_NONE|short chain dehydrogenase family protein

MSGIVTVFGASGFLGRYVVRRLAQAGWRVRAAVRDPNLALFLRPYGAVGQVEPVACNIRDAASVARVLDG

ADAAINCIGILTELRANTFDAVHHLGAALIARTARAAGVQRLVHVSALGTGGQGSAYFDSKAAGEAAVLA

AFPSAVVVQPAVMFGRDDHFFNRLAGLARLPVLPIVGGDVKMQPVWVDDVAAAIVTALAPDFAAGTYPLA

GPEVMTMQQIAQQVLQVTRRSTRIVDLPLGLARFAAGIAEFGHRASFSILPVPLSRDQIAMLSYTHHLPD

GSGFQPFGITPAPTGVVLPDYLWRFRPAGQFTAIRESAAKLRDAKGAD*

>gkv_1030|gene_NONE|beta-lactamase family protein

MSVFRIWLRRIVLGVIAIAVLTLAGLWVIAPQILVLAREGFPGQVWPTSGTYLEVAGAQTPAAPPAGRAL

PAAAHDRLVNTSGRALLMERAGVLEYEEYTAGLAREDRFNSYSMVKSLIGAMIIRAVADGKIASLDDPLS

DYLGPESPDTTIRAVLTMTSGLELHGLQTKQMEDGDFSAFSGVAELHAFGIERLLPRLHPNAAVAGTFRY

ESSNSAVLGAVLEQVYQQRLPALLSTLIWQPAGAQTASWRAYPMSGGATAYCCLYARPLDWLMVGRYLLD

NGTPEAPFLPQPLWNDLFLPTLSPEQRQDRFYGYHISHNVMDRAGENVAGPFAYFTGHLGQVVYLLPEQN

TVVVRFGAQRQPLQATLYDLF*

>gkv_1031|gene_NONE|metallo-beta-lactamase superfamily protein

MNIIWLGHGSFRIEIGDQILLVDPWLTDNPVFPTAKRDEAVAGTTHILLTHGHSDHVADVLEVAATTGAP

IIAIADLAGYLHKTQGAQTIGINKGGTLRLGNVAVTMVNASHSSTLAGPDGLVAVGSEAGFMIAGEGHVI

YFSGDTDIMADMAWMGELHAPDIGILSAGGHYTMDMSRAAWAAKKYFHFETVIPCHYRTFPLLEQNADAL

KAGLPGVDVIEPDVLVPITIAAK*

>gkv_1032|gene_gatC|glutamyl-tRNA(Gln) and/or aspartyl-tRNA(Asn) amidotransferase, C subunit|

MSIDIETARRVAKLARIRVEPEALPALASEFSAILGFIEQLNEVDVEGVEPMTSVTPMRLKRREDIVTDG

EQQAAVLANAPDAREGFFAVPKVVE*

>gkv_1033|gene_gatA|glutamyl-tRNA(Gln) and/or aspartyl-tRNA(Asn) amidotransferase, A subunit|

MTDISKLTIATARDLLRKGELTSRELTDSYISAIEGADALNAYVHKTPDIARAQADAADARLKAGDAPDM

CGIPLGIKDLFATKGVASQAASGILAGFKPEYESTVTSKLFGDGAVMLGKLNMDEFAMGSANESSVYGPA

VNPWRAAGSDAALTPGGSSGGSAAAVAADLCLAATGTDTGGSIRQPAAFTGVTGIKPTYGRVSRWGIIAY

ASSLDQAGPMTKDVRDAAIMLTAMSGTDSKDSTSADLAVPDFEAALTGDIRGKTIGIPREYRIDGLSPEI

AKLWDDGIAMLKDAGAKIVDISLPHTKYALPAYYVIAPAEASSNLARYDGVRFGHRAQLSAGDGINEMYE

KTRAEGFGPEVQRRIMIGTYVLSAGFYDAYYNRARRVRTLVKQDFEQAFAAGVDAILTPATPSSAFALGM

ESADPVQAYLNDVFTITVNLAGLPGISVPAGQDSKGLPLGLQLIGKPWEEGDLLNIAYSLERAAGFVAKP

ARWW*

>gkv_1034|gene_NONE|conserved hypothetical protein

MDLAMKRILLSMVALSALAACQSSQDYSSNTGSNAGFSAPVAVSSAPLGAAGSSPITSAPADANRTAGVQ

ASPTNTGIASTGISTQEDFETLSSRVSIEEDAALIAQQRAAYQVVQPTALPTNPGNTGPNIVEYALNAPN

QKGQAWYSRFMWASEGRFQRNCAAYSSADEAQRDFLARGGPERDWRGIDPDGDGFACGWDPAPFRAAAGR

*

>gkv_1035|gene_rpmG|ribosomal protein L33

MAKPTTIKIRLNSTAGTGHFYVTKKNARTMTEKMTVNKYDPVVRKHVEYKEGKIK*

>gkv_1036|gene_NONE|hypothetical protein

MQQDGIPRICVPLQADQLWLFDPRDTDAAADLGVDPAWSQASWQGALVEGSERPSWQIADAARRAGADGI

IDPSRQIAGGWHVMLFRWDHVRVAGPGVLLPQ*

>gkv_1037|gene_NONE|integral membrane protein-like protein

LPYEWVKPRAAPEESGAVAVSHPPIAELHLWPYRSLPLRGFVTFMVITLLLIAVPLTVALGSPVLWGVLP

FFMLTIWGLYLAFRRNYRDGHVLERLLVWDDHVQLIRTGTYERRREWEANPHWVRLTLIPEGGPVPNYIT

LRGSNREVEIGAFLSEEERQALAAELRPLFGPRR*

>gkv_1038|gene_ctaD|cytochrome c oxidase, subunit I|

MADAAIHGHDHHEKQGFFTRWFMSTNHKDIGLLYLVAAGVVGFISVLFTVYMRLELMDPGVQYMCLEGAR

LIADASQTCTANGHLWNVMVTYHGILMMFFVVIPALFGGFGNYLMPLQIGAPDMAFPRMNNLSFWLFIAG

TAMGVASLFAPGGDGQLGSGVGWVLYPPLSTREAGYSMDLAIFAVHLSGASSIMGAINMITTFLNMRAPG

MTLHKVPLFSWSIFITAWLILLALPVLAGAITMLLTDRNFGTTFFNPAGGGDPILYQHILWFFGHPEVYI

IILPGFGIISHVVSTFSKKPVFGYLPMVYAMVAIGVLGFVVWAHHMYTVGMSLTQQSYFMLATMVIAVPT

GIKIFSWIATMWGGSVEFKSPMLWAFGFMFLFTVGGVTGIVLAQAGLDRAYHDTYYVVAHFHYVMSLGAI

FAIFAGIYFYMPKFSGRAFPEWAAKLHFWTFFIGANVTFFPQHFLGRQGMPRRYIDYPEAFALWNKVSSY

GAFLAFASFLFFIVIFVYTLVAGRRETRPNPWGEFADTLEWTLPSPPPAHTFETLPKRSDWDKHPSH*

>gkv_1039|gene_NONE|D-alanyl-D-alanine carboxypeptidase family protein

MSRKLNTLIAGLAVLAMGTSSAMAQSFVTSARAAYVLDQTTGTVLLDHNADEVLPPASMSKLMTIYMAFE

AVSNGRLHLTDELRVSQHCMNYGGSSMFLNTQDRPHVEDLLRGVIILSGNDASCVLAEALSPDGTEGGFA

ALMTTRAHELGMSNSHFMNSNGWPAAGHLMSMRDLGTLSRHLIEDFPTFYPIFAETEFHYDGRVPSNSQN

RNPILSLGIGADGLKTGHTSEAGYGLAGSARQGDRRVIFVITGLESEAARRDESERIINWAFRQFALQDL

GKAGDIIPGGTADVWMGAAPRVQLALGQDLQILVPTTSANEMTTEVVYNGPIAAPITAGQELATLVIDRE

GMPQMTVPLVAETDVPAGGFVNRVMGSAMILLGKVGGAADQGA*

>gkv_1040|gene_tmk|thymidylate kinase|

MTCGLFISFEGIDGSGKSTQARVLADALRGRGHDVVLTREPGGSPGAEEIRRLVLTGATDRWSAETETLL

FTAARRDHLEKTILPALAAGQIVITDRFADSTRAYQGATRGDLRGIVDALHALMIGREPDLTFVIDADPA

ASLTRGLARSTDELRFEEFGLPLQQKMRAVYLDIAHAQPQRCIVIDGAGTIDAVAARILTAFDAR*

>gkv_1041|gene_NONE|DNA polymerase III subunit delta'

MSDDTLPDPTAVAGMPHPRESHVLLGQGRAEHEFTDAAASGRMHSGWLLTGPQGIGKATFAYRAAAWLLA

GAPTSGMDLPDDHPDLRLIRAGSHPRLLVIKRGPNEKGDRLESVITVRAMRQLGSFFGLSATDGGRRVVI

VDAADDMNPNAANALLKLLEEPPAGAVLLLIAHQPARLLPTIRSRCRVLPLAPLTGDDLAAILQAEDGAA

LSALAQGSAGAAARIVAHDGLKLYADLVAVMDGMPRFDRPRATQLIASVTARGADGRLALLVDLIDLFLS

RAARAGILGAPLPAASPHETAVLQRLAPHDGAARLFADLQQTLSAKLRQGLAVNLDPAMLILDTLLEIEG

ALRKLP*

>gkv_1042|gene_NONE|hydrolase, TatD family protein

MTAPAAIVDSHCHLDFADFDGQLDEIIARATAAGVTRLVTICTRLDQEPRVRAIAESYPGVYYAAGIHPM

RAAEEPLVTVDQLVALAQHPKFVGIGETGLDYHYSADSAAIQQQSLRIHCAAAAEAGLPLIIHARDADDD

MAAILAEEHRKAPFSCVMHCFSSGAALARAAVDLGFYLSMSGITAFPKSGELRDIFRAMPLDRILLETDS

PYLAPPPHRGKRNEPAFTALTAARAAETFGLDYADFARLTSENFDRLFTKAA*

>gkv_1043|gene_NONE|metallo-beta-lactamase superfamily protein

MQDSADICVRILGCGSSGGVPRLGGEWGDCDPFEPKNTRTRCSILVTRETDQGRTQVLIDTSPDMRQQLL

AADVRTLDGVLYTHPHADHVHGIDDLRAISFNGDQRLDVWMDASTTAAITHRFGYIFKTPEGSPYPPICV

QRLIEGPVTITGAGGPITFEPLRVEHGNISALGFRFADIAYIPDVSLIPEDVWPQLSGLDCWIVDALRYK

PHPSHSHVAQTLGWIDRAAPKIAVLTNMHVDLDYQTLGQELMAGVTPAFDGMVLRFRA*

>gkv_1044|gene_NONE|membrane transport family protein

MLAIIDVILPVFLVLGAGYLVAWRGGMSETAVAGLMKFAQGFALPCILFRGISQLDLAHSFSPSLVVSFY

VPATVCFFVGIAGARLWAKRPWEDSIAIGFACLFSNSMLLGLPINDRAYGPESMDTVLAIVSMHAPFTYF

LGITAMEIARNVGQTPLMVARKVARSIFTNNLVIGVLAGVVVNVLNIPIYHTIGEAMDLIGRAAVPAALF

GLGAVLYRLRPEGDMRLILMVSAISLGLHPVLTYLFGQSFGMTGVALNTMVLTAAMAPGVNAYLFADMYG

VGRRVNASSVLIATTISILSLPIWIIVMG*

>gkv_1045|gene_NONE|hypothetical protein

MTFHHTPIASHAGQRPPRDWAWRLTRSLAPIIIAMTLLFIAVGSLLATAAGSVQQLSVNGAVIEMPRIIQ

RGSAGEAVMIFDGPAADHEIVLGQALMDNIEITRVSPSTVSAADDAGMKLLFPASTESPYILAINLIATG

WGRVHFPLWVDGVQHDITLFIIP*

>gkv_1046|gene_NONE|H-type lectin domain protein

MWAGDGNRSLEVRVSFDRLYAYKPEVLLSLAAVDSAREQNLRYDLRVKDVDTNGFTIHFATWSDTRISLA

TVSWFAFGTAQ*

>gkv_1047|gene_NONE|hypothetical protein

MHGISIHERLKNWRYAALRQAKYRAIYAHAVMVAHLDGHLVADDHPSWTRIDAALDAARAGDAEAISIVL

REITRLRDN*

>gkv_1049|gene_NONE|AAA ATPase containing von Willebrand factor type A (vWA)-like domain

MPNSYLNGRYLMAALASALSLTAAGAAFAQTQPTLVEQLIAQYQAEGFTRVEIEQGRARIEVNAIRGQDD

IEAVYDAITGALLYQTQERVERDDDTRPGVWVERDDDDEDDRPGRGQTGGKDDDRWDDDDRDDWNDRDDD

RDDDRDDRDDRDDDDDDDDDDRYDRD*

>gkv_1050|gene_NONE|hypothetical protein

LGERLERGGGKAEAKGQRAKPLKRQMSQQFFLLSDGVAPAPWLRGLWVK*

>gkv_1051|gene_NONE|aspartic acid-rich protein aspolin2 domain protein

VQTQIVSQLTAQGFTRIEVDQTLLGRLRFRAWSDSGLYREIVIVPATGEILRDYMRSAASGAASIPQLFT

PDDGGSGSAGGLSSDDDDDDDDDDSDDDSDDDDDD*

>gkv_1052|gene_NONE|bacterial regulatory proteins, luxR family protein

MPGRFGIIALLIVQLVCAVLFLWNTLISVLGLPPLAWEVNEFIEIGAACGLLIGVAMGIVTLRDALRRAK

RAESQLRAASGAFMDLLQEKFLQWGLTPAERDVALFAIKGFSLSEIAQMRQTSEGTVKAQTNAIYRKAGV

SGRPQLLSIFIEDLIEAPISPLAAAPTPATLPTVTEK*

>gkv_1053|gene_NONE|putative lipoprotein

MRFLIPLVAALGLTACAIPDVSTRPAPESYPALINVDPMMRDAAALGATNPTATQDDLDARAAALRARAA

ALRAN*

>gkv_1054|gene_NONE|homoserine dehydrogenase (HDH)

MTAPLRLGIAGLGTVGTGIIRIIQEHGQMLAARAGRPVQIVAVSARSNNKDRGVALDGYAWESDPVALAK

RDDIDVFVEVMGGHEGPARAATLAALDSGKDIVTANKALLAIHGNEIAAKAEGAGRVLRFEAAVAGGIPV

IKALTEGLAGNRITRVMGVMNGSCNYILTRMESAGLTYEEVFEEARALGYLEADPQLDVGGIDAGHKLSL

LSAIAFGSKVDFNAVELEGIGAITIEDIRHAADLGFRIKLLGVAQLTGRGLEQRMTPCLVPAHSPLGQLQ

GGTNMVVLEGDAVGQIVLRGPGAGSGPTASAIMSDVIDIARGIRMPTFGIPATSLVAQPAARSAVPAPYY

IRMQLDDKPGALAKVARILGDNGISIDRMRQYGHDGSAAPVLIVTHKTMRSAVELALEALPQTAVVTGET

VALRIESL*

>gkv_1055|gene_glpX|fructose-1,6-bisphosphatase, class II|

MTTAPEFHDRMLSLGLARVSEAAALASAAMVGRGDEKAADKAAVDAMRTQLNMLDIAGVVVIGEGERDEA

PMLYIGEEVGTGTGPAVDIALDPLEGTTLTAKDMPNALTVIAMAPRGTLLHAPDVYMDKLAIGPGFPANT

VSLDMSPAERVYALAKAKGVEAADITVCILDRERHQDMIAEVRGTGAAIRLITDGDVAGVMHCADPETTG

IDMYMGSGGAPEGVLAASALKCMGGEIYGRLLFRNDDERGRAAKAGITDLNRIYTRDDLVTGDVIFAATG

VTDGSLLRGIKREPGFFTTETVLMRSKTGSVRRITYRCPIK*

>gkv_1056|gene_recJ|single-stranded-DNA-specific exonuclease RecJ|

VSFLGVTDSVTGRAWVGPDLETDRLTEALSQQLGLSRPLAALLAQRGIDADGAEGFLTPRLRDLMPDPRS

LRDMEKAAARLVLAVQTRQRIAIFGDYDVDGGASSALLIDWLRNFGLQATLYIPDRIDEGYGPNVPAMAD

LAARHDLIICVDCGTLSHEALGAATAADVIVLDHHLGGETLPPALAVVNPNRQDESGDLTYLCAAGVVFL

ALVEANRQLRAGGLQGPDLTSLLDLVALATVADVAPLVGLNRALVRQGLAVMGRRARPGLVALADVARID

SAPSSYHLGYILGPRVNAGGRIGRADLGARLLATTDPHEAAAMAAQLDALNTERRAIESAVRDAAIAQAE

ARGLEAPLVWAASEGWHPGVVGIVAARIKEATNRPSVVIGFDGGIGKGSGRSVSGVDLGAAIQRVAAEGL

LLKGGGHKMAAGLTVTRENLEPAMARLSELLARQGAGAGGPSDLRIDALLMPGAATVAMIEQLEQAGPFG

ASAPAPRFALPDVVVQYVKPVGSDHLKVTLGDGLGARLDAIAFNAAQSPLGHLLTHSGSQRVHVVGRLEV

NHWQGRASAQLRIEDAAKASS*

>gkv_1060|gene_NONE|hypothetical protein

MSASWSVITVMRNTPYEVMRFVAWYLDMGADQIYIIFHDENDPFIARLQGHPRITCIPFTADLRAQMGID

PDYAGPPQIQAGTYLYPRVTTDWVLRLDCDELVLCENGRLSERLAALPADIQTAIIRPCEQLISDLPAHH

HLFRMRMTDEDAQQIYGDMAELLSKRRGLMSHAFGKSAHRTGIQNIEVREHNAVFIGTNTRTNNLAWMRK

KGVSLLHFNAEVFEKWKAAAARRSRNASFAPALSAKILAAEASETADTELRQIYDTIVHFDTRRTDALLE

TDCGFVLDFDFDVLIDRYFDSAQLMLRAG*

>gkv_1059|gene_NONE|ribbon-helix-helix protein, copG family protein

MHRVTITIETALLDELDAYMARSGASNRSEALRDLVRRGLAQQGAEVGEAQCVGVVSYTLDPSVRALGRR

VPQSRQDRHDHTIAALSVPLDHDSAVEIAVMRGRVAQVSAYAEGLFLERGIRHGKLSLIPVQHDVETHTH

EGGEPHEHSHLRVRESF*

>gkv_1061|gene_NONE|bacterial extracellular solute-binding proteins, family 5 Middle family protein

MKLSHLLSASCVLALTAGMASAQVVLNRGNDTDPSTLDHHRTSTVSESRLMNDLYEGLVTKAADGSTIPG

VAESWDISEDGLVYTFHFRDDAKWSNGDAVTAEDFLYAYRRLMDPATAAPYANMLFPIVNAEAIASGEAE

ADTLGVRAIDATTLEITLSSATPYFLELLTHQTGLPIHAASVEEFGDSFTQAGRMVTNGAFQLVSFTPND

MIVMSKNTNFHDAENVAIDQINYIPFEDRAACLRRFEAGEIQICTDVPTEQMDYLEANLAEELHVVPYLG

TYYLPIKGEEGSPLRDPRVRQAISLVIDRDFIATEVWRDTMLPGYSLVPPGIVNYVDGGVMLPYADEDLL

DREDAAKALLEEAGVAPGTLTVKLRFNTSENHRNTMAAVADMLSNIGITGELDEVEGASYFSYLQQGGMY

DIARAGWIGDYNDPQNFLFLFQSDVQFNYPRWVNADYDAAIDAAATETDLAARAEILAQAETILLDELPI

IPILWYSSRALVSPSISGYEDNLMDDHLSRWLSVN*

>gkv_1062|gene_NONE|oligopeptide transport system permease protein oppB

MLGYTLRRLASAIPTIFIIVTLTFFMIRVAPGGPFNLERPLDPLIMQNLMRAYNLDAPLWEQYVLYIKNL

LHGDLGPSFTRRDFSVNDLFAAGLPVSIMLGGLALLFASIIGTFLGALAALRQNSWLDNLIVALATFGIT

TPNFVIAPLLSMFFGVILGILPAGGWSNANPAYWVLPIITLALPQIAVIARLVRGSTIEALSANHVRTAR

AYGLPTRVVVGVHALRAAMLPAVSYLGPTAAGLLTGSVVVETIFGLPGIGRYFVQGALGRDYTLVMGTVV

VISIFVVIFNLLVDLAYAWLDPRVRYD*

>gkv_1063|gene_NONE|oligopeptide transport system permease protein oppC

MTDATSIPTGGARESRSLWQDAMRRLRRNRAAMASLIVLVAMTLIAIFGPILSPHPPERIYQQYVRVAPS

LEAYPKADNILPGFAREFSRTRLTGDEPVLDGSTLSVNITSDEAIDTRVLRYFERSNLFSAPALELSEDG

RTGTLSVQVERRYFLLGTDNLGRDLMTRIFIGMRISLMIGVLASVMALVLGVTYGAISGYLGGRADNIMM

RVVDILYSLPFIFFVILMLVFFGRSMAIIFIAIGATEWLDMARIVRGQTLSLKRREFVQAAEALGASRAG

ILTRHIIPNALGPVIVFMTLLVPKAILLESLLSFLGLGVQDPLTSLGLLISEGANNMRGAAYQLIFPAAT

LTIILFALNFLGDGLRDSLDPKER*

>gkv_1064|gene_NONE|uncharacterized ABC transporter ATP-binding protein yejF

MSETVLSVRDLRVTFQTDDGDVEAVRGINFDVEAGKTLAIVGESGSGKSQTTMAIMGLLSRNGRATGQAL

YRGQDLIGLGEKQLNNVRGNKITMIFQEPMTSLDPLYTIGTQLAEPLRHHRGLSKSAARPRILELLKLVG

IPEPERRIDSYPHELSGGQRQRVMIAMALANDPDVLIADEPTTALDVTIQDQIMTLLADLQKRLGMAIIF

ITHDLGIVERFADHVCVMRKGEVVEDGPATQVFANAQHPYTQMLLAAEPEGTKAPPPADAPSVLNGQNVA

VHFNIGKSLFKKQVFTAVNDVSLNLRQGQTIGVVGESGSGKSTLGRALLRLLPADGTIAYLGKPLPYGEG

PMRPYRKQLQLVFQDPFGSLSPRMTVGRVITEGLQIHAPELSGRERAARAREALVEVGLDADMINRYPHE

FSGGQRQRIAIARTMVLRPKVIVLDEPTSALDRSVQKQIVTLLRDLQEKHGLTYLFISHDLSVVRALSDY

IVVMKSGKIVEQGETTQVFDAPAEEYTRTLMAAALSKRRFRED*

>gkv_1065|gene_NONE|dioxygenase family protein

MTQQRPDQPDDITRRRIMQRLVAAVPLAAIGASAAKAEPSADIALAAATMGLITTNVCAVKPETTEGPYY

IDPKLVRSDITEGKAGIPLRLSIQVVTADCRPVAGARVDIWHCDAQGNYSGYANQGSDGTLNTEGQTFLR

GTWPADENGIATFDTIYPGWYRGRTTHIHYKIYLDERTVLTSQIFFPDALSEYLFLNAAAYQRSDTRDTV

NKGDGIAAEAGEGAYCAIREQADRYIAALVVGIDPDATWTEGGQGMGGPGRGGPEGGPGGGMPPGPPPEG

SAPPGQMPGQPPEAGDPADRVLFPDGV*

>gkv_1066|gene_NONE|glutathionylspermidine synthase family protein

VHLPISVENSCTLAKNGHLQGEPSVKSRSMHRISLPPRPDWQDKAEAAGFTFHTMYGAPYWAEDVAYRFT

LAQIEDDIEDPSTQLHAMVREAVARITTSEDLMARMGLPFAHWDYIANSWRAAEPELYGRMDLAYDGNGP

AKLLEYNADTPTSLFESASFQWDWLQDQIGLGHLPQGTDQFNRTFEAIAERFAEIFARGTDVHFASVANN

TEDYGTVETLAWAAREAGLGAHYTPMHEIGLTETGQFADNQSRVMGVLFKLYPWENLLAEPFAENLAGAH

LRMIEPPWKALVSNKAILPVLWDMFKGHPNLLPSYFADDAHGADDMPHGTVRKPLFSREGASVTILGPDG

QTIARAEDRSYDQYPEIVQAYHPLPVFDGYRPVLGAWIAGETCVGLGLREDQSPITQNLSRFVPHIIQE*

>gkv_1067|gene_NONE|putative lipoprotein

MPLTIGLTVSAFALAACRPEVTEMDAQLFPDANACYAAADAGGSSILRSDCDTAATEAQAMHEANAPRYD

ALATCEAEYGAGNCTDTAQNSGGGMGSFFMPLLMGYMMGNMLSGGRNASQPIYSQASGGYTTSDRSSTFA

NNSGNTRVNAASFSNAASGQSVRNTGNTSARPAAAPMSQSTVRSTGGFGGSASSGGFGG*

>gkv_1068|gene_NONE|L-lysine 2,3-aminomutase (KAM) (LAM). domain protein

VALQPADLVPDGLISPADAAALRPVTETFRMRITPQMRTAITRADDGVGLQFVPDRRELNVLPSELTDPI

GDGAHSPTKGITHRYPDRVIFHVTQVCEVYCRFCFRREVVGENGVLPAGDVAAALDYIRRTPAINEVILT

GGDPLSLSPRRLHEITTALAAIPHVGLMRIHTRVPVVAPNRITPEMIAALTAPGLQTWLVLHTNHPQEFI

PEAVAALDLLRTAGVPLLSQSVLLRGVNDSVAVLKSLFTTLLRLGVKPYYLHHCDLARGTSHYRTTIAAG

RALMRALRGQISGSALPTYVLDIPGGFGKVPITADYFDGGADGRWQVTDPNGGTHIYHDPE*

>gkv_1069|gene_NONE|ompA-like transmembrane domain protein

MKMNRYFAATAMVALVAANTASAGGLTAEVVEPVVVVPVVAPAAATRFTGAYVGASLGYTFSEDDRVGVH

TPAGTFLGDIGEFELAGPSAAIFGGYRWNSGKWVFGPEVSVRFGDVSDDISYAGPIPAASTGTKELNWEA

ALRATLGYEVTPTTLVYGFLGYSAVEYDYVLNGAITLDETVSQNGLTAGLGVEHALNDTWAVRGLYVYND

YDRENLTGSGGRYTTDTTQLHTVNVGLVYSF*

>gkv_1070|gene_NONE|outer membrane protein

MIKKFIVASAVVAASVGSVNTAVAGGLSQEIVEAPVVIPVAPVLTNRFAGGYVGLGYGHVVSSDDRVGMV

DQTTNLLTGVLGDLDLEGGAAILQAGYRWANGNLVYGPTLRVRGGGIDAELTGTSTGSSTVNWEAALRGN

LGYTVTPSLLLYGFVGYTYAEVEYDVAGAINVAETVNFGGVNAGLGAEYALNDNWSLFGEYEYAGYEGKN

LVDPAINQHTRPTPDFHSVNIGVNFSF*

>gkv_1071|gene_cysC|bifunctional enzyme nodQ|

MTDDVKSHAYEADRLIAEDIDAYLSQHQHKSLLRFITCGSVDDGKSTLIGRLLYDSKMIFEDQLAALESD

SKNVGTQGQDIDFALLVDGLAAEREQGITIDVAYRFFTTDKRKFIVADTPGHEQYTRNMATGASTADLAV

ILIDARKGVLTQTRRHSYLVKLLGIPNVVLAINKMDLVDYSAATFHQIVADYSAFAESIGLNSFVPVPIS

GLKGDNIVEKSSAMPWYQGPTLIAHLETVPLGDTSQEKPFRMPVQWVNRPNLDFRGFTGLIAAGAVRPGD

RVRVQPSGRESTVKEIVTFDGNLDLAVAGQSVCLTLNDEIDCSRGDVISTALAPAETADQFESTIIWMDE

AAMLPGRPYIMKLGTQQAAVTITEPKYEVNVNTMERLAAKTLGLNSIGVCNISTDRQITFAPFGDNKTLG

SFILIDRATNATVGAGLINFSLRRAQNIHWQSVDINAEAHAAQKGQQPKLVWFTGLSGSGKSTIANMVEK

KLFALGKHSFLLDGDNVRHGLNRDLGFSDADRVENIRRVGEVGKLMVESGLIVLTAFISPFRNERQMVRD

MLPAGTFIEVFIDTPLAVAESRDVKGLYRKARAGQLKNFTGIDSPYEAPESPDLHVDTTAISAEDAAEMI

VAHIIG*

>gkv_1072|gene_cysD|sulfate adenylyltransferase subunit 2 (Sulfate adenylatetransferase) (SAT) (ATP-sulfurylase small subunit)|

MTHAQDTAVKPKAETLSAVQLTHLQRLEAESIHIMREVVATAENPVMLYSIGKDSSVLLHLARKAFYPAP

PPFPLLHVDTTWKFRAMYDMREAAAAKAGMTLLVHQNPEAKAKGINPFEHGGLHTDMWKTEGLKQALDHY
[truncated: 822,891 more chars]
